# Comparative proteomic analysis of human embryonic stem cell-derived and primary human retinal pigment epithelium

**DOI:** 10.1038/s41598-017-06233-9

**Published:** 2017-07-20

**Authors:** Heidi Hongisto, Antti Jylhä, Janika Nättinen, Jochen Rieck, Tanja Ilmarinen, Zoltán Veréb, Ulla Aapola, Roger Beuerman, Goran Petrovski, Hannu Uusitalo, Heli Skottman

**Affiliations:** 1 BioMediTech Institute, Faculty of Medicine and Life Sciences, University of Tampere, Tampere, Finland; 2 Department of Ophthalmology, Faculty of Medicine and Life Sciences, University of Tampere, Tampere, Finland; 3 Stem Cells and Eye Research Laboratory, Department of Ophthalmology, Faculty of Medicine, University of Szeged, Szeged, Hungary; 4 Singapore Eye Research Institute and Duke-NUS School of Medicine, Singapore, Singapore; 5 Center for Eye Research, Department of Ophthalmology, Oslo University Hospital and University of Oslo, Oslo, Norway; 6 Tampere University Hospital Eye Center, University of Tampere, Tampere, Finland

## Abstract

Human embryonic stem cell-derived retinal pigment epithelial cells (hESC-RPE) provide an unlimited cell source for retinal cell replacement therapies. Clinical trials using hESC-RPE to treat diseases such as age-related macular degeneration (AMD) are currently underway. Human ESC-RPE cells have been thoroughly characterized at the gene level but their protein expression profile has not been studied at larger scale. In this study, proteomic analysis was used to compare hESC-RPE cells differentiated from two independent hESC lines, to primary human RPE (hRPE) using Isobaric tags for relative quantitation (iTRAQ). 1041 common proteins were present in both hESC-RPE cells and native hRPE with majority of the proteins similarly regulated. The hESC-RPE proteome reflected that of normal hRPE with a large number of metabolic, mitochondrial, cytoskeletal, and transport proteins expressed. No signs of increased stress, apoptosis, immune response, proliferation, or retinal degeneration related changes were noted in hESC-RPE, while important RPE specific proteins involved in key RPE functions such as visual cycle and phagocytosis, could be detected in the hESC-RPE. Overall, the results indicated that the proteome of the hESC-RPE cells closely resembled that of their native counterparts.

## Introduction

The retinal pigment epithelium (RPE) is a multifunctional, polarized epithelial cell layer between the neurosensory retina and the choroid, which plays key roles in photoreceptor function and vision. The RPE cells transport nutrients, waste products, ions and fluid between the choroidal blood supply and the subretinal space. RPE also phagocytizes shed photoreceptor outer segments (POS), absorbs scattered light, secretes many important signalling molecules and functions in the retinoid visual cycle^1^. This highly metabolically active cell type is exposed to constant light stimuli and high oxidative stress making it vulnerable to oxidative damage. Thus, abnormalities in RPE cell function may lead to retinal degeneration and photoreceptor cell death. The RPE is the focal point of many retinal degenerative diseases such as age-related macular degeneration (AMD), the most common cause of blindness in the elderly in western countries. AMD is a multifactorial, age-associated disease characterized by accumulation of insoluble drusen in the retina, degeneration of RPE and photoreceptors in the dry form, and choroidal neovascularization in the exudative, wet form of the disease^2^.

Treatment options for the retinal degenerative diseases such as AMD are currently very limited and mostly only delay disease progression. Cellular transplantation to replace the affected RPE is considered as a promising therapeutic strategy to treat these diseases. Macular translocation and autologous RPE transplantation with peripheral RPE have demonstrated the feasibility and effectiveness of autologous RPE cell replacement therapy in AMD patients, but these surgical procedures carry significant complications^3^. Many cell types have been tested as a source for RPE transplantation tissue including foetal RPE^4^ and RPE cell lines^5, 6^. Issues related to scarce tissue availability and characteristics of immortalized adult human cell lines, and the fact that they only weakly mimic some of the native RPE characteristics after *in vitro* passaging, make these cells suboptimal for treatment of the large population of patients^7, 8^.

Human pluripotent stem cells (hPSC), including both human embryonic stem cells (hESC) and human induced pluripotent stem cells (hiPSC) can be differentiated to retinal cells, including photoreceptors and mature and functional RPE cells^9^. Their high capacity to self-renew and wide differentiation potential makes them an excellent cell source for both cellular models for research purposes as well as cell replacement therapy approaches. Encouraging results have shown that transplanted hESC-derived RPE cells (hESC-RPE) can mediate functional photoreceptor rescue in the Royal College of Surgeons (RCS) rat model of retinal degeneration^10–12^. Moreover, ongoing phase I/II clinical studies have recently demonstrated that it is possible to safely implant hESC-RPE to end stage patients with AMD and other retinal degenerative diseases^13^. Similar studies with autologous hiPSC-RPE have also been initiated in Japan^14^, although suspended later for one of the two patients due to safety concerns regarding genomic stability of the patient’s hiPSCs^15^.

Our research group, along with many others, has shown that the hESC-RPE structure, function, and physiology closely resembles that of their native counterparts with a high rate of pigmentation, polygonal, cuboidal epithelial cell morphology, cellular fine structure, and expression of many RPE signature genes and proteins^16–22^. In addition the cells show epithelial integrity and functionality with the ability to phagocytose POS and secretion of growth factors^18^. However, large scale comparative studies of the proteome, the total protein complement of a genome, of the hESC-RPE cells are lacking, while the proteome of hRPE, especially related to AMD has been extensively studied^7, 23–25^. Stringent characterization of hPSC-RPE using standardized and quantifiable methods is important to gain confidence that they are safe and adequate replacements for diseased RPE if they are utilised in clinical settings.

In this study, we have performed a comparative proteomic analysis of *in vitro* differentiated hESC-RPE cells to primary hRPE using Isobaric tags for relative quantitation (iTRAQ). iTRAQ quantification is based on incorporation of stable isotopes into peptides followed by liquid chromatography fractionation and tandem mass spectrometry analysis. iTRAQ-based proteomics has previously been used for example to study early stage hESC differentiation^26^ and changes in visual cycle proteins in mice RPE in conjunction with RPE65 gene delivery^27^. We have also previously successfully applied the technique for characterization of corneal epithelial cells derived from hPSC^28^. In this study, the results showed that the overall spectrum of protein expression of the hESC-RPE cells and the primary RPE were similar.

## Results

### A large number of common proteins were expressed and regulated at similar level in hESC-RPE and hRPE

**Figure 1:**
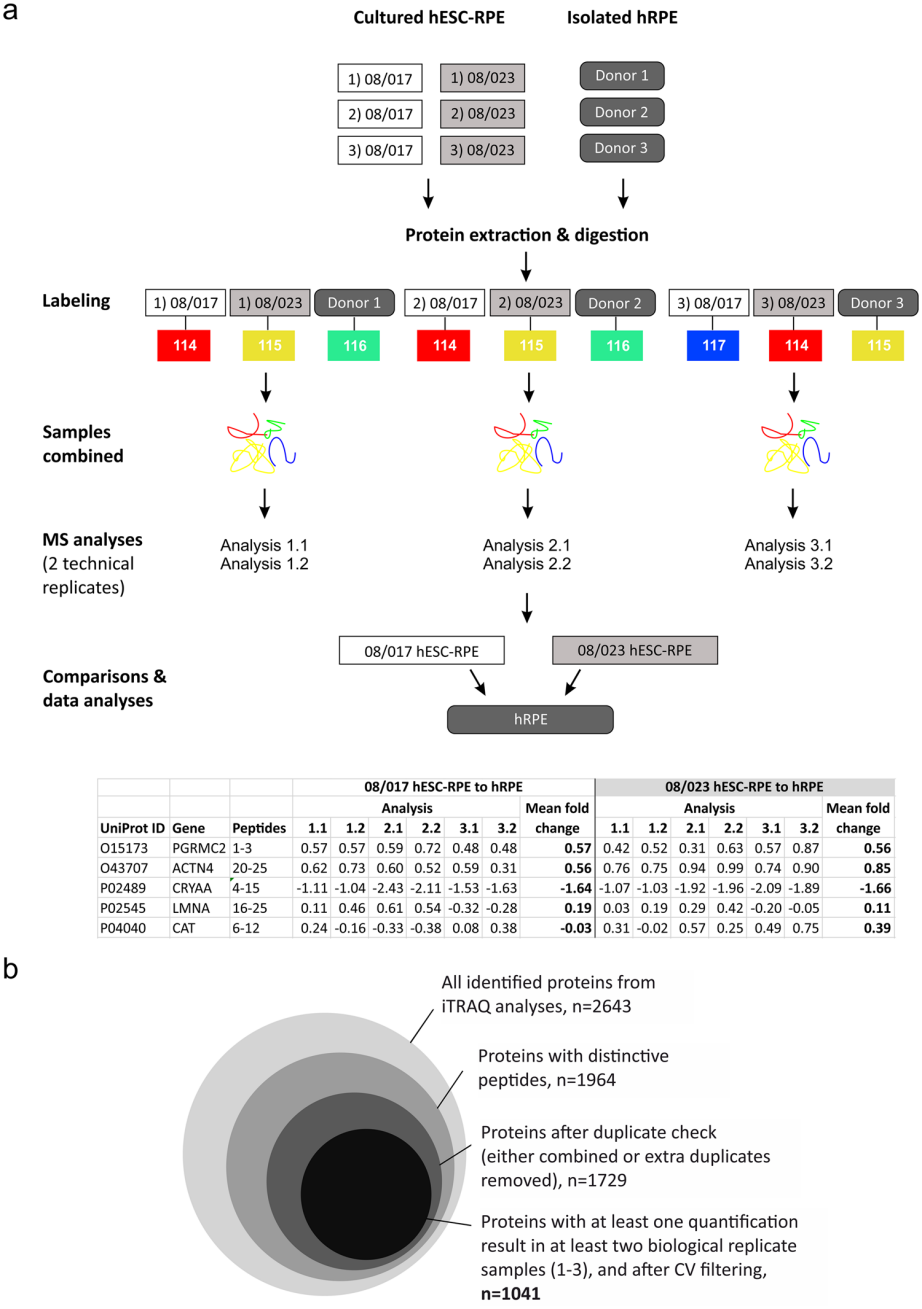
Workflow of the study. (**a**) Study design of the iTRAQ proteomics. Human ESC-RPE samples from three independent differentiation experiments consisted of three replicate samples each. Human RPE samples were collected from three cadaveric donor eyes. Total protein was extracted and digested with trypsin. Peptides were labeled with iTRAQ 4-plex labels 114–117 shown in colors, and analyzed as technical duplicates with Nano-RPLC-Triple TOF instrumentation. For final data analyses the results were pooled togerther from all six LC-MS/MS analyses separately for 08/017 nad 08/023 hESC-RPE. (**b**) A Venn diagram illustrating the data processing before data analyses from total number of protein hits to 1041 proteins  accepted for final data analyses.

iTRAQ proteomics was performed to compare protein expression of hESC-RPE to primary hRPE. The experimental setup and workflow of the study is shown in Fig. 1a. Three replicate samples of two hESC lines Regea08/017 and Regea08/023 differentiated to RPE cells (referred to as 08/017 and 08/023 hESC-RPE), were compared to hRPE samples from three different cadaveric human donor eyes. Digested peptides were labelled with iTRAQ reagents as shown in Fig. 1a, incorporating one biological sample of 08/017, one sample of 08/023, and one sample of hRPE to each of the analysis reactions. The labelled, combined samples were subjected to three LC-MS/MS analyses, which were conducted in two technical replicates each. For data analysis the replicate results were combined as shown to allow relative quantitative comparison of the protein expression in hESC-RPE and hRPE.

**Figure 2:**
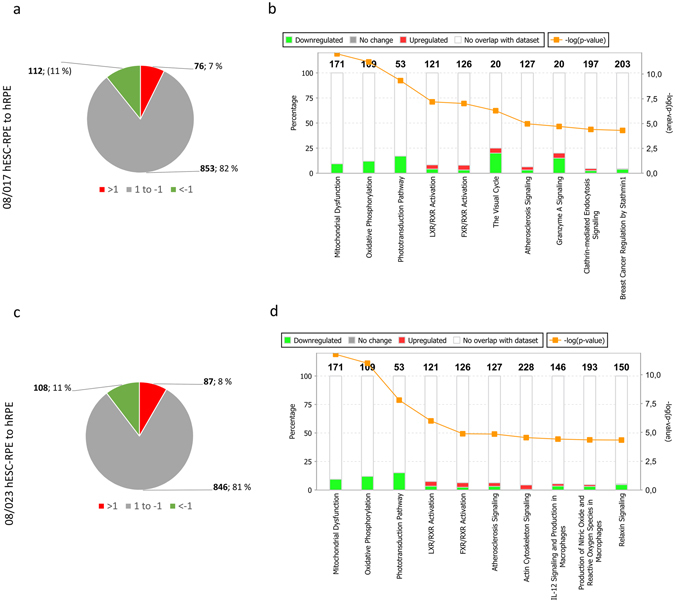
Protein expression differences between hESC-RPE and hRPE. The proportions of upregulated (green) and downregulated (red) proteins according to relative expression levels of the proteins compared to hRPE for (**a**) 08/017 hESC-RPE and (**c**) 08/023 hESC-RPE. For the majority of proteins in both RPE lines, there were no differences in expression level between hESC-RPE cells and hRPE (grey). Number of proteins included = 1041. Default settings of IPA were used to conduct core pathway analysis for the over- and underexpressed proteins (188 proteins for 08/017 and 195 proteins for 08/023). Ten enriched pathways with lowest p-values for the differentially expressed proteins in (**b**) 08/017 and (**d**) 08/023 hESC-RPE compared to hRPE. Total number of genes/proteins in each pathway shown on top of each bar and percentage present in the data analysed on the y-axis (left). Up- and downregulation of the proteins in the data is shown in red and green colours respectively, and p-value (orange line) is shown in -log scale, y-axis (right). Figures in (**b**,**c**) created with IPA (Qiagen) software.

The iTRAQ proteomics allows the identification and quantification of proteins that are expressed in all samples included in each labelling (Analysis 1.1–3.2 in Fig. 1). 2643 unique proteins (Uniprot Accession) expressed by both the hESC-RPE and hRPE were identified in at least one technical replicate sample (Fig. 1b). The number of proteins present after stringent data processing and filtering to ensure data quality are shown in Fig. 1b. 1041 unique proteins expressed in at least two biological replicate samples of both 08/017 and 08/023 hESC-RPE, and in the hRPE donor sample were included for final data analysis. A complete list of the proteins is provided in Supplementary Table S1. Log_2_ scale was used for analysing the data. As protein expression of the hESC-RPE were compared to the protein expression in hRPE, over two-fold differences in protein expression (corresponding to fold change values >1 or <−1 on log_2_ scale) were considered significant overexpression (upregulation) or underexpression (downregulation). Majority of the proteins were expressed at similar levels in hESC-RPE and hRPE. 82% of the proteins were similarly expressed in 08/017 hESC-RPE and 81% in 08/023 hESC-RPE, respectively, when compared to hRPE (Fig. 2a and c). Only 7% of the proteins were upregulated in 08/017 and 8% in 08/023 hESC-RPE cells, while 11% were downregulated in both cell lines comparing to hRPE. Pathway analysis of the over- and underexpressed proteins in the hESC-RPE compared to hRPE was conducted for both 08/017 and 08/023 to inspect if enriched active pathways arise from the differentially expressed set of proteins. IPA (Ingenuity Pathway Analysis, Qiagen) core analysis of the canonical pathways indicated that proteins associated with mitochondrial dysfunction, oxidative phosphorylation, and phototransduction pathways were downregulated in hESC-RPE (Fig. 2b and d). The log_2_ fold change values for the differentially expressed proteins for both 08/017 and 08/023 are presented in Supplementary Table S2, and visualised in Supplementary Fig. S1. Overall, mostly homogenous data with similar expression patterns across the mass spectrometry analyses (three analyses with two technical replicates each) was produced, as shown in Supplementary Fig. S2. Variations in the expression levels between the three analyses were thought to result from the three individual hRPE donor samples used for comparison in each analysis. A comparison of a pooled sample from the three hRPE donors to a single donor (Donor 1) was done to evaluate inter-donor variability in protein expression. The results showed mostly similar expression with only 2% of the proteins differentially expressed between pooled donor sample and Donor 1, indicating that the differences between the donors were subtle (Supplementary Fig. S3).

### hESC-RPE showed a normal RPE protein expression profile

**Figure 3:**
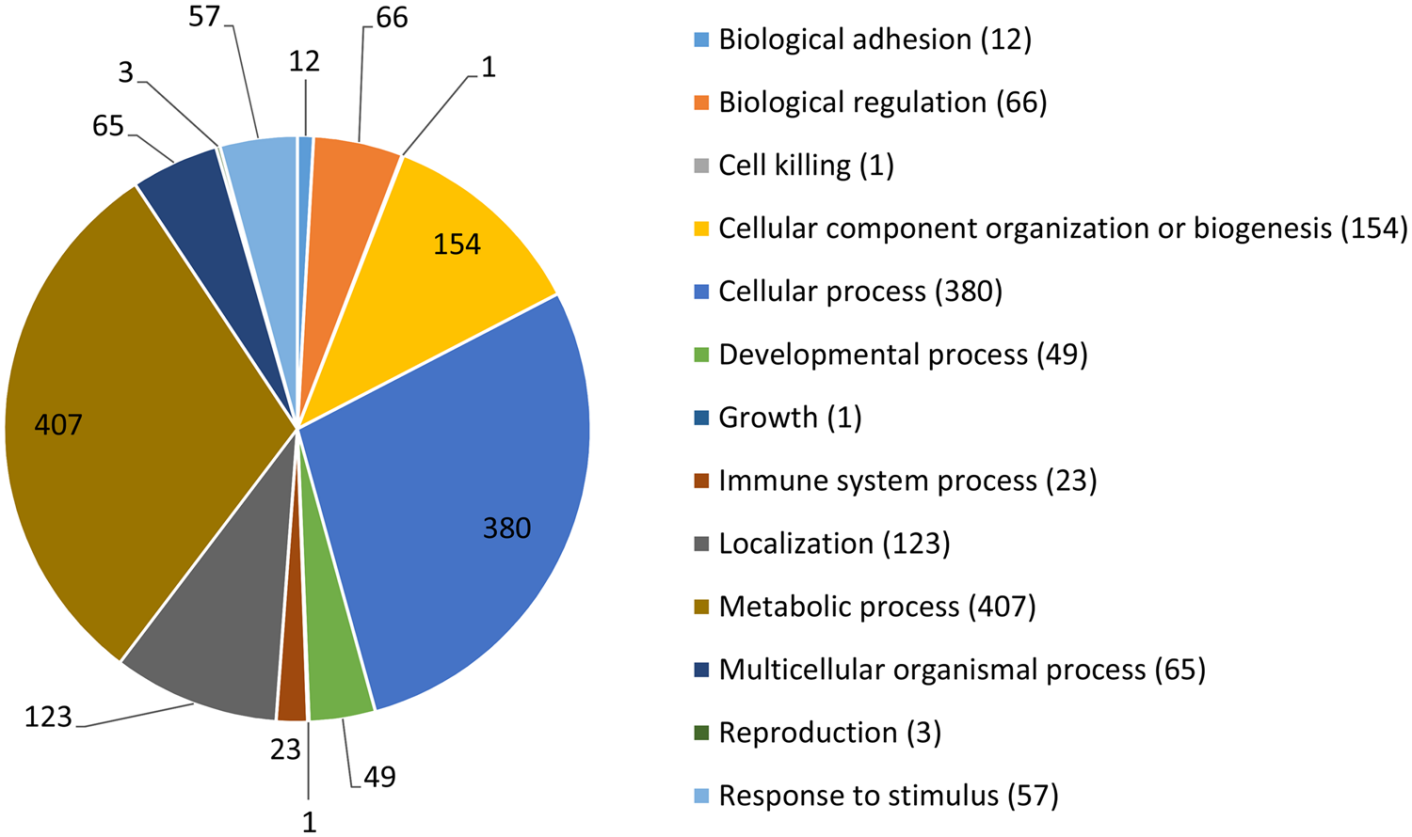
Distribution of proteins. A pie chart showing distribution of the proteins according to their *biological process* using Panther Classification System. The proteins were classified by their corresponding gene names. 852 of the 1041 proteins were classified into one or more of the 13 groups.

The PANTHER (Protein ANalysis Through Evolutionary Relationships) Classification System and Gene Ontology (GO) term mapping were used to examine specific protein groups of interest. PANTHER classification of the expressed proteins according to ***biological process***, classified the proteins into 13 groups as shown in Fig. 3. The largest group of proteins was metabolic processes which included 407 proteins. Most of these proteins (369) fell under the subclass of primary metabolic processes. Cellular processes included 380 proteins and contained proteins involved in cell communication (76), cell cycle (47), cellular component movement (19), cytokinesis (11) and growth/proliferation (1). Cellular component organisation and biogenesis was the third largest group with 154 proteins. Localisation with 123 proteins included proteins involved in transport. A subclass response to stress, the major subclass under response to stimulus, included 34 proteins. Immune system processes included 23 proteins.

**Figure 4:**
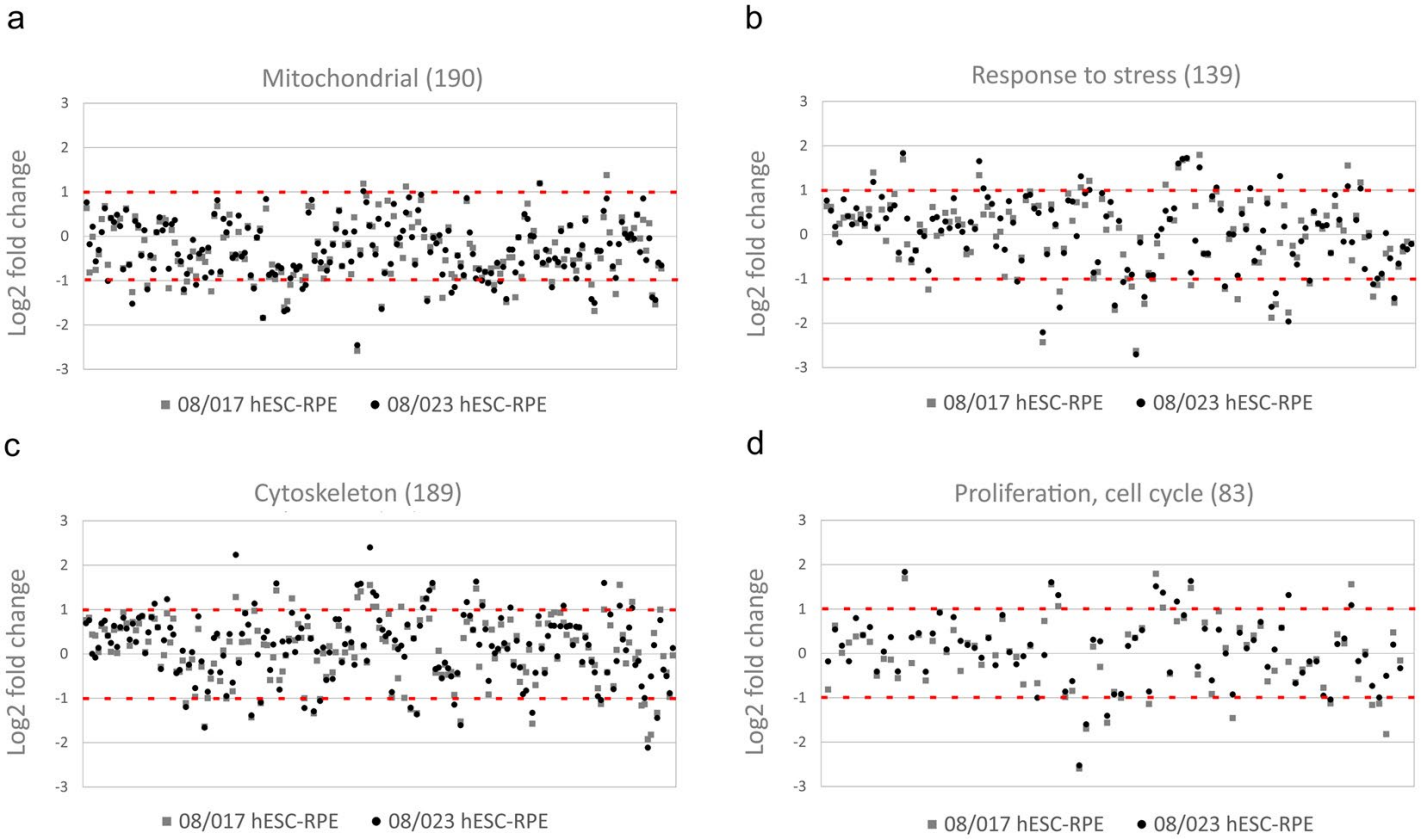
Proteins involved in mitochondrial functions, stress, cytoskeleton, and proliferation. Relative expression of (**a**) mitochondrial, (**b**) cellular stress-related, (**c**) cytoskeletal, and (**d**) proliferation and cell cycle proteins in both hESC-RPE cell lines compared to hRPE. Each dot represents a single protein, presented as mean fold changes on a log_2_ scale, where y = 0 denotes equal protein expression. Red dashed lines mark the borders for over and under expression (2-fold difference in expression). For protein names and exact expression values in each class see Supplementary Tables S4–S7.

To analyse the specific protein groups more carefully, GO term mapping was used to identify proteins connected to specific GO terms (Supplementary Table S3). The protein expression profile of hESC-RPE cells was found to closely resemble that of native RPE. A large number of ***mitochondrial*** proteins were expressed at similar level (85% for 08/017 and 86% for 08/023) or slightly downregulated, in the hESC-RPE cells compared to primary hRPE (Fig. 4a). All together 139 proteins involved in ***cellular stress*** were identified (Fig. 4b). Of these 78% were similarly regulated in 08/017 hESC-RPE and 79% in 08/023 hESC-RPE compared to hRPE. Proteins that fell into the category ***cellular stress*** included many proteins involved in immune responses, such as major histocompatibility complex MHC class I antigen (Fragment, HLA-A) and CD59 antigen that were similarly regulated. Other proteins of interest in the class included galectin-3-binding protein (LGALS3BP), clusterin (CLU), annexin A1 (ANXA1) and −5 (ANXA5), apolipoproteins A (APOA) and -E (APOE), crystallin alpha A (CRYAA), crystallin alpha B (CRYAB), cystatin C, catalase (CAT), galectin-1, superoxide dismutase 1 (SOD1) and −2 (SOD2), and protein S100. A large number of ***cytoskeletal proteins*** (189) were also mostly similarly regulated in the hESC-RPE and hRPE (83% for 08/017 and 80% for 08/023) (Fig. 4c). Cytoskeletal keratins: −8, −10, −18, and −19 could be detected and of these −8, 10, −18 were upregulated in both hESC-RPE lines as were ezrin (EZR), fibrillin-1 (FBN1), fibrillin-2 (FBN2), and filamin A (FLNA). ***Proliferation and cell cycle*** proteins also showed mostly similar regulation in hESC-RPE and hRPE (Fig. 4d).

### Important proteins for RPE function and characteristics were expressed in hESC-RPE cells

**Figure 5:**
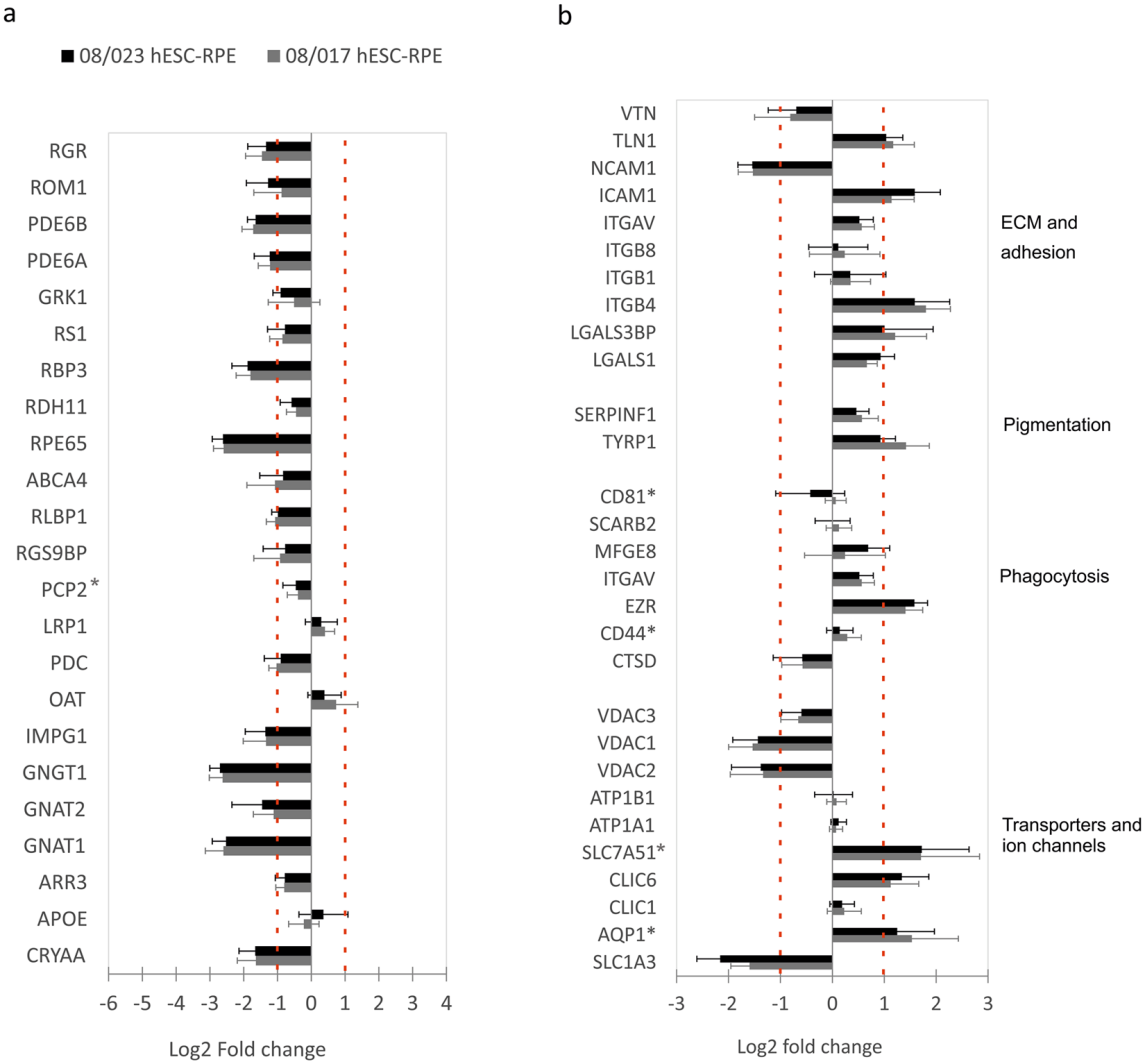
Proteins involved in important RPE functions. (**a**) Relative mean expression levels of the 23 proteins involved in perception of light stimulus and (**b**) of selected important RPE proteins. Protein expression levels shown on log_2_ scale. Red dashed lines mark the borders for overexpression and underexpression (over 2-fold difference in expression). Error bars denote standard deviation. For the full protein names and expression values see Supplementary Table S8. *Quantification based on less than two peptides.

Proteins involved in the ***perception of light stimulus*** were identified and 13 of these 23 proteins were downregulated in the 08/017 hESC-RPE and 11 proteins in the 08/023 hESC-RPE compared to the primary RPE, while the rest were similarly expressed (Fig. 5a). Important RPE-specific visual cycle proteins identified included for example retinal-specific ATP-binding cassette transporter (ABCA4), retinoid isomerohydrolase (RPE65), retinaldehyde-binding protein 1 (RLBP1), and retinol dehydrogenase 11 (RDH11). There were 46 proteins involved in ***extracellular matrix*** (ECM) and ***cell adhesion***. Among those were integrin subunits αV (ITGAV) and three beta chains −1 (ITGB1), −4 (ITGB4) and −8 (ITGB8), of which the integrin ß-4 was upregulated in both hESC-RPE lines. Integrin binding talin-1 (TLN1) was slightly upregulated as was intercellular adhesion molecule 1 (ICAM-1) and galectin-3 binding protein (LGALS3BP). Neural cell adhesion molecule 1, (NCAM-1) was downregulated in the hESC-RPE, while vitronectin (VTN) was among the similarly expressed proteins (Fig. 5b). Proteins involved in ***melanogenesis*** included pigment epithelium-derived factor (SERPINF1) that was similarly expressed in hESC-RPE and hRPE, while tyrosinase-related protein 1 (TYRP1), was upregulated in 08/017 hESC-RPE. A total of 98 proteins involved in ***phagocytosis***, ***protein degradation and autophagy*** were identified. These included proteasome subunits and 26 S proteasome proteins. Interesting proteins involved in phagocytosis included integrin alpha V, part of the αVβ5 integrin, and its ligands tetraspanin (CD81, fragment), and lactadherin (MFG-E8), and these proteins were expressed at similar level as compared to hRPE. Also, CD44, CD36 (scavenger receptor class B, member 2), and cathepsin-D were expressed at similar level in the hESC-RPE and hRPE. ***Transporters and ion channels*** (90 proteins in total) expressed included chloride intracellular channel proteins 1 and −6 (CLIC1, and CLIC6). Na^+^K^+^-transporting ATPase subunits alpha-1 and beta-1 (ATP1A1, ATP1B1) were similarly regulated. Aquaporin 1 (fragment) (AQP1) was upregulated in hESC-RPE. Voltage-dependent anion-selective channel proteins 1 (VDAC1) and −2 (VDAC2) were downregulated in hESC-RPE while VDAC3 was expressed at similar level. Amino acid transporter (SLC1A3) was downregulated in hESC-RPE, while large neutral amino acids transporter small subunit 1 (SLC7A5) was upregulated (Fig. 5b).

## Discussion

Ultimately, proteins expressed by a cell determine the phenotypic expression of genomic information^29^. The proteome is a dynamic entity characterizing the cell type and changing with the physiological status of the cell. Pioneering proteomic profiling studies have revealed the protein expressions in human RPE/choroid complex as well as *in vitro* cultured RPE cells^7, 25, 30–32^, changes in RPE proteome associated with the progressive stages of AMD^23, 24, 33, 34^, and in response to oxidative stress^35^. The protein content of drusen and lipofuscin has been elucidated with proteomic approaches^36^. To our best knowledge, large scale analyses of hPSC-RPE proteome have not been previously performed. The objective of this study was to compare the protein expression profile of RPE cells differentiated from hESCs to the native primary hRPE proteome. We chose a sample setup with a different biological replicate of both hESC-RPE and hRPE included in each analysis, in order to observe true biological differences between the samples. In the quantitative 4-plex iTRAQ analyses, 1041 common proteins were present in both hESC-RPE cell lines and primary hRPE samples. Importantly, majority of these proteins (>80%) were similarly regulated in the hESC-RPE and native hRPE, showing that the hESC-RPE cells resembled their native counterparts. The expression patterns of the two hESC-RPE lines were also similar, showing that our differentiation protocol yielded homogenous RPE cells with low cell line specific variation in protein expression.

The protein profile of the hESC-RPE cells resembled that of native primary hRPE. A large number of metabolic and respiratory proteins were expressed reflecting the highly metabolically active nature of the RPE. The pathway analysis of the differentially expressed proteins indicated mitochondrial dysfunction and downregulated oxidative phosphorylation in the hESC-RPE, reflecting reduced energy requirements in the *in vitro* cultured hESC-RPE cells compared to native cells. This is consistent with some previous published results on the proteome of *in vitro* cultured RPE cells^7^. Apart from single upregulated proteins, no signs of increased stress, apoptosis, immune response, proliferation, or retinal degeneration-related changes were observed in the hESC-RPE. Cellular stress markers and many proteins considered as biomarkers for AMD were similarly regulated: galectin-3-binding protein (LGALS3BP) which was slightly upregulated in hESC-RPE has been implicated as AMD marker, but also involved in attachment and spreading of RPE cells^37^. Clusterin (CLU) and apolipoprotein E (APOE) are major constituents of drusen^36^, and were similarly expressed in hESC-RPE and hRPE. Crystallin alpha A (CRYAA), downregulated in hESC-RPE and crystallin alpha B (CRYAB) that was similarly regulated, are protective of oxidative injury and both drusen constituents as well as AMD biomarkers^34, 38^. Cystatin-C (CST3) is abundantly secreted by the RPE^39^, while not much is known about the function of cystatin-B (CSTB) in the RPE. Annexins (ANXA1, ANXA5) involved in many processes including inflammation and apoptosis^40^, were also similarly regulated as were metabolic proteins such as prohibitin (PHB), superoxide dismutases 1 and −2 (SOD1, SOD2), and glutathione peroxidase (GPX1) that have been implicated as oxidative stress biomarkers^41–43^. While inflammation-related protein intercellular adhesion molecule 1 (ICAM-1) was upregulated, MHC class I antigen (fragment, HLA-A) was similarly regulated in both hESC-RPE lines and MHC class II antigen was not detected. Our unpublished results, in accordance with published data by others^44^, show that in the standard culture conditions, our hESC-RPE cells express MHC class I but not MHC class II antigens without exposure to proinflammatory cytokines. The membrane complement regulatory protein CD59 was also similarly regulated. Protein S100, and macrophage migration inhibitory factor (MIF) have previously been shown to part of macular and peripheral RPE proteome^25^.

As highly differentiated monolayer of polarized cells, the RPE cells maintain themselves outside the cell cycle. During *in vitro* cell culture and certain pathologies, RPE cells undergo dedifferentiation (epithelial-to-mesenchyme transition, EMT) to become actively proliferating cells acquiring a fibroblastic morphology. Dedifferentiated RPE cells express cell cycle markers and cytokeratins such as cytokeratin −8, −18, and −19^45^. Cytokeletal crosslinkers actin binding filamin-A (FLNA) and ezrin (EZR), which is localised to apical microvilli in the RPE, were upregulated in the hESC-RPE. Ezrin has a role in POS phagocytosis but its overexpression has also been linked EMT^46^. Fibrillins −1 and −2 are structural proteins of the ECM microfibrils, involved in a variety of functions including eye development and morphogenesis^47^. Overexpression of the cytoskeletal remodellers and cytokeratins -8 and -18 in both hESC-RPE lines and cytokeratin-19 in 08/023 hESC-RPE, most probably reflects the *in vitro* nature of the cells. The *in vitro* hPSC-RPE differentiation involves serial passaging after which the cells proliferate to colonize the cell culture surface and then regain their epithelial phenotype. The hRPE cells used for comparison in this study were not *in vitro* cultured to avoid the culture induced changes to their cellular phenotype and proteome. No signs of transfer to more active status of cell cycle or proliferation was detected in the hESC-RPE cells.

Importantly, highly RPE-specific proteins such as the visual cycle proteins were identified in the hESC-RPE. The RPE layer is the principle site for 11-cis retinal regeneration in the visual cycle. RLBP1 and RPE65 are important biochemical RPE markers present in hESC-RPE and hiPSC-RPE^18^. The downregulation of expression of these proteins is expected for the *in vitro* differentiated and cultured hESC-RPE as compared to native tissue, since the hESC-RPE cells have not been in contact with the neural retina or retinoids. It has been previously shown that hiPSC-RPE cells possess the machinery to process retinoids and can exhibit a functional visual cycle *in vitro* and *in vivo*. Human iPSC-RPE cells maintain RPE65 protein expression during culture in contrast to primary cells and after incubation with all-trans retinol, hiPSC-RPE release 11-cis retinaldehyde into the culture media^48^.

ECM and cell adhesion-related proteins identified included integrin subunits αV and three beta chains: 1, 4, and 8. Integrin ß-4, part of the primary laminin receptor integrin α6ß4, was upregulated in both hESC-RPE lines. Laminin is an important component of Bruch’s membrane and the epithelial cell basement membrane. Integrin α6ß4 promotes attachment of RPE to Bruch’s membrane^49^ and is thus important for integration of hESC-RPE cell transplants. Absorption of stray light by the abundant melanin granules is another important function of the RPE. Proteins involved in melanin biosynthesis such as tyrosinase-related protein 1 (TYRP1) was detected as being upregulated in the hESC-RPE. High pigmentation status and secretion of retina protective pigment epithelium-derived factor, PEDF (SERPINF1) by our hESC-RPE cells has been shown previously^18^.

RPE cells are among the most active phagocytes in the body. Circadian shedding of POS activates a synchronized phagocytic response by RPE cells every morning^50^. Many phagocytosis related proteins were detected in hESC-RPE. The integrin αvβ5 is in fact required for POS binding to the RPE^51^. Integrin αvβ5 complexes with the tetraspanin CD81, an interaction that promotes particle binding. Engulfment receptor CD36 at the apical, phagocytic surface of RPE cells and lactadherin (Milk fat globule-EGF factor 8) are also involved in POS recognition. Cathepsin-D (CTSD) is a major proteolytic enzyme in phagocytic cells that digests opsin. Ezrin is known to bind to lysosomal-associated membrane protein 1 (LAMP-1) and CD44 during the formation of phagocytic vacuoles^50, 52^.

The RPE also functions as a barrier between the neural retina and the fenestrated choroid performing a variety of vectorial transport functions (water, ions, metabolites, nutrients and waste products) between the two compartments^1^. Important transporters and ion channels were identified in the hESC-RPE such as Na^+^K^+^-transporting ATPase subunits alpha-1, and beta-1 and −2 (ATP1A1, ATP1B1) localized at the apical surface of the RPE as shown previously for our hESC-RPE^18^. Aquaporin 1 (fragment) (AQP1), channel protein that mediates water transport, is apically expressed both in human foetal RPE primary cultures and in hESC-RPE cells^19^. Proteomic analyses of the plasma membrane after subcellular fractionation could provide further insides into expression of transporter and channel proteins by the hPSCs.

A specific normal RPE gene expression signature of 154 genes has been proposed for validation of RPE-like cells derived from hPSC^53^. In that study, only eight proteins were analysed at protein level. The changes at the mRNA level often do not lead to corresponding changes at the protein level and large scale, protein level studies of the hPSC-RPE are therefore also important for validation. The iTRAQ method is limited to the detection of only those proteins that are present in all samples of each iTRAQ labelling. Thus proteins expressed in the hESC-RPE only, or solely in the primary hRPE sample could not be detected with this method. In addition, a 30 kDa filter was used in the experimental setup to reduce noise and ion suppression caused by the small molecules in the samples. Proteins under the limit such as lecithin retinol acyltransferase (LRAT), a 25 kDa visual cycle protein and an important RPE marker, could not be detected. In the iTRAQ, quantification of low abundancy proteins can cause miss quantification arising from background ions^54^, and therefore careful data checking is important especially for proteins with extreme fold change values. Data dependent analysis (DDA) mode is required for iTRAQ label intensity based quantification and causes missing value generation in the data as number of replicate runs increase. This is a well-documented phenomenon in ITRAQ analyses^55^ and was also observed in our data with 661 proteins detected in only one analysis run i.e. one biological replicate (Supplementary Table S1, p. 12–19). Despite of these limitations of the method, iTRAQ enables multiplexing, that is comparing four different samples in a single mass spectrometry experiment. Highly reliable comparative, quantitative data can thus be produced especially with samples that have similar protein expression patterns^56^. Although the 1041 proteins detected only presents a fraction of the total RPE proteome of over 4000 individual proteins detected from RPE-choroid samples in other studies^25^, the data presented provides new, important, and quantitative information on expression of over 1000 common proteins between hESC-RPE and hRPE. The idea of this study was to quantitatively compare the proteome of hESC-RPE to hRPE, not to accumulate the whole hESC-RPE proteome, which would require a much larger sample size and a different MS methodology. New methods like sequential window acquisition of all theoretical fragment ion spectra (SWATH), might enable analysis up to 4000 proteins from RPE samples. Building a whole proteome library of the hPSC-RPE is a target for our future studies.

To conclude, we were able to detect 1041 common proteins between the two hESC-RPE samples and primary hRPE and demonstrated that the hESC-RPE differentiation yields high quality RPE cells. The hESC-RPE cells differentiated *in vitro*, in serum-free conditions and in the absence of contact with their natural niche, still resembled the hRPE expressing RPE specific functional protein machinery. No protein abnormalities indicative of pathological behaviour of the hESC-RPE was detected which is important considering the future clinical applications. Proteomic approaches enabling absolute, label-free quantification such as SWATH technique could be used in the future to even more precisely compare the hPSC-RPE proteome to native tissue. As the hPSC-RPE also provide unlimited opportunities to model retinal degenerative diseases *in vitro*, quantitative, comparative proteomics opens possibilities to understand protein level disease mechanisms and discover new therapeutic targets for devastating diseases such as AMD, and also aid in validation of hPSC-RPE for clinical applications.

## Methods

### Human embryonic stem cell-derived RPE cells

The two hESC lines previously derived at our laboratory at University of Tampere were used in this study: Regea08/017 (46;XX) and Regea08/023 (46;XY)^57^. The undifferentiated hESCs were culture in hESC culture medium consisting of KnockOut DMEM supplemented with 20% KnockOut Serum Replacement (ko-SR, Thermo Fisher Scientific), 2 mM Glutamax, 0.1 mM 2-mercaptoethanol, 1% Non-essential amino acids, 50 U/ml penicillin/streptomycin, and 8 ng/ml human basic fibroblast growth factor (bFGF) on inactivated human foreskin fibroblast (CRL-2429; American Type Culture Collection) feeder cells. Undifferentiated stem cell colonies were passaged with TrypLE Select onto fresh feeder cell layer every 10 days. Both cell lines are routinely karyotyped and characterized for their self-renewal and differentiation capacities as well as absence of mycoplasma.

The hESCs were differentiated to RPE as previously described^18^. Briefly, hESCs were manually cut to suspension and cultured as floating cell aggregates in RPE basic medium consisting of same reagents as hESC medium except for 15% ko-SR and no bFGF. For enrichment, pigmented cells were isolated and seeded to human placental collagen IV (5 µg/cm^2^). For further maturation the cells were seeded to collagen IV (5 µg/cm^2^) coated PET cell culture inserts (Millipore) and cultured for 129 days (Regea08/017) or 139 days (Regea08/023). Regea08/017 at passage levels 33, 34 and 70, and Regea08/023 at passage levels 44, 61, and 70 were used for generating RPE cells. Cells from three independent differentiation experiments (replicates 1–3) were used for the study. Each biological replicate consisted of three cell culture inserts. The hESC-RPE cells were collected with TrypLE Select, washed twice with PBS, pelleted by centrifugation and stored as dry pellets at −80 °C until protein extraction.

### Human RPE cells

Primary hRPE from three different donors were used. The donor eyes had no macroscopical or microscopical signs of retinal degeneration. For human retinal pigment epithelium isolation, following enucleation, the eye bulb was washed with 5% povidone iodine (Betadine), the anterior segment was cut approximately 3.5 mm posterior to the limbus and the anterior segment, the vitreous and neural retina were removed. The eyecup was then filled with RPE cell culture medium (DMEM/F12, supplemented with 10% FCS, 1% antibiotics-antimycotics and 1% L-Glutamin) and the cells collected by a special glass cell scraper to gently remove the RPE from Bruch’s membrane while continuously bathed in their own cell culture medium. The RPE was collected as a pellet by centrifugation (1000 rpm, 10 min). The RPE pellet was washed twice with PBS, followed by centrifugation after each wash, and shipped for further analysis at −20 °C.

### Protein extraction

Human ECS-RPE pellets were suspended in 200 µl and hRPE pellets in 400 µl of RIPA lysis buffer, supplemented with 0.1% HALT protein inhibitor cocktail, and dissociated using a VWR disposable pestle. The samples were then vortexed for 2 min, incubated in an ultrasonic bath for 5 min, and on ice for 25 min. Dissociated samples were centrifuged at 14 800 rpm for 15 min at 4 °C, supernatants transferred to clean tubes and their protein concentrations determined using the DC Protein Assay Kit II (Bio-Rad Laboratories) according to the manufacturer’s instructions. Bovine serum albumin (BSA; Bio-Rad Laboratories) was used as a standard, and all samples analysed in duplicates. 15 µg of total protein/hESC-RPE insert were used and pooled together for each biological hESC-RPE replicate (1–3) for both cell lines (45 µg in total). Equal amount (45 µg) was used for each hRPE donor sample (1–3) (Fig. 1). Additional pooled hRPE sample was prepared by pooling 15 µg of protein from each hRPE donor (45 µg of protein in total). Proteins were precipitated by adding six volumes of cold acetone, followed by quick vortexing and overnight incubation at −20 °C. The following day, samples were centrifuged at 14 800 rpm for 15 min at 4 °C. The acetone was discarded and evaporated for 10 min, and the pellets subjected to protein digestion.

### Protein digestion, iTRAQ labelling, and mass spectrometry analysis

Protein digestion, labelling and mass spectrometry was done as described in detail previously^28^. 30 kDa size exclusion filters (Pall Corporation) were used in sample preparation. Samples were labelled with the isobaric iTRAQ tags as shown in Fig. 1 and pooled together for the mass spectrometry analysis. Labelled samples were analysed by Nano-RPLC- TripleTOF instrumentation using Eksigent ekspert™ 425 NanoLC coupled to high speed TripleTOF™ mass spectrometer (Sciex 5600+). A capillary RP-LC column (cHiPLC® ChromXP C18-CL, 3 µm particle size, 120 Å, 75 µm i.d × 15 cm; Eksigent, Concord, Canada) was used for LC separation of peptides.

### Data processing

Protein pilot software version 4.0.8085 (Ab Sciex) was used to analyse MS/MS data searched against the UniProt/Swiss-Prot protein database for protein identification. Some important settings in the Paragon search algorithm in protein pilot were configured as follows. Sample type: iTRAQ 4plex (peptide labelled), Cys-alkylation: MMTS, Digestion: Trypsin, Instrument: TripleTOF 5600+, Search effort: thorough ID. False discovery rate (FDR) analysis was performed in the Protein pilot and FDR <1% was set for protein identification. Only peptides with 99% confirmation were included in identification and quantification. Shared peptides were excluded from quantification that was performed using ProteinPilot software with straight average of peptides (proteins with distinctive peptides, n = 1964, Fig. 1b). Data processing included log_2_-transformation and central tendency normalization. Graphical presentation of the data normalization is shown in Supplementary Fig. S4. Ranking of proteins (measure of quality) was created by taking the mean of the rankings in different runs. Duplicate proteins with the same gene names were merged so that only one protein name was kept. If both SwissProt and Trembl duplicate proteins were present, SwissProt was chosen (number of proteins after duplicate check, n = 1729, Fig. 1b). The spectral peaks for proteins with over 4-fold difference (log_2_ fold change value >2 or <−2) in expression in either hESC-RPE line comparing to hRPE were manually checked, and misquantified proteins were omitted. These included phototransduction related proteins such as opsin, recoverin, rhodopsin, rod outer segment membrane protein 1, and peripherin-2 that were present in high abundance in the human donor samples but misquantified due to noise ions in the hESC-RPE. The manual checking confirmed that these photoreceptor specific proteins were not present in the hESC-RPE (data not shown). The data was filtered so, that only proteins which were identified in at least two biological replicate samples of both hESC-RPE lines and human RPE (i.e. in two of the three analysis runs), were considered reliable and included in data analyses. Proteins based on detection in only one biological replicates (one analysis run, n = 661) were filtered out. Finally, proteins with either technical or biological CV >100 (n = 27), were excluded from the final data analysis. The proteins filtered out are shown in Supplementary Table S1. P-values, which are shown in the Supplementary Table S1 and Supplementary Fig. S1, were obtained using a one sample t-test, where the means of technical replicates were taken prior to testing. Unadjusted p-values were presented (as no significant changes were seen for 08/017 hESC-RPE to hRPE, and only seven proteins (Uniprot IDs: O15537, B7ZB41, J3KPF3, Q6FHV6, Q76LA1, V9HWH2, X5DNM4) reached significance in fold change for 08/023 with adjusted the p-values).

Classification into specific groups of interest was done by GO mapping with the GO terms connected to each protein’s UniProt accession using UniProt.ws package in R (Marc Carlson. UniProt.ws: R Interface to UniProt Web Services. R package version 2.10.2.). The GO terms of interest as well as their offspring terms, i.e. more specific terms, were included in the GO term mapping process due to the loose hierarchical structure of GO annotation. The GO terms used are listed in Supplementary Table S3. Processing of the data, GO mapping, boxplots, and volcano plots for visualization were created using R software version 3.2.3 (R Core Team, Foundation for Statistical Computing, Vienna, Austria, https://www.Rproject.org/). QIAGEN’s Ingenuity® Pathway Analysis (IPA®, QIAGEN Redwood City, www.qiagen.com/ingenuity) was used to conduct canonical pathway analyses using the differentially expressed (log_2_ fold change >1 or <−1) proteins in 08/017 and 08/023 hESC-RPE compared to hRPE. The PANTHER Classification System Version 11.0 (release date July 15, 2016) was used to classify the proteins according to their involvement in biological processes (http://pantherdb.org/). Charts were created in Microsoft Excel 2013.

### Ethical issues

All native tissue collection was approved by the National Medical Ethics Committee of Hungary (14415/2013/EKU – 183/2013 and DEOEC RKEB/IKEB 3094/2010) and complied with the Guidelines of the Helsinki Declaration (1964). RPE/RPE-choroid collection was done within 12 hours of biologic death from cadavers and followed the EU Member States’ Directive 2004/23/EC on presumed consent practice for tissue collection (European Parliament CotEU. [2015.09.21]. (Available from: http://eur-lex.europa.eu/legal-content/EN/ALL/?uri=CELEX:32004L0023). All relevant data pertaining to the status of the patient before death were checked before tissue collection. The use of human embryos for research purposes at BioMediTech has been approved by the National Authority for Medicolegal Affairs Finland (Dnro 1426/32/300/05). The institute also has supportive statements of the Ethical Committee of the Pirkanmaa Hospital District to derive, culture, and differentiate hESC lines (Skottman/R05116). No new cell lines were derived for this study.

### Data Availability

The datasets of the study are available from PeptideAtlas online database http://www.peptideatlas.org/PASS/PASS01012.

## Electronic supplementary material

Supplementary Material

## References

[1] Strauss O (2005). The retinal pigment epithelium in visual function. Physiol. Rev..

[2] Ambati J, Atkinson JP, Gelfand BD (2013). Immunology of age-related macular degeneration. Nat. Rev. Immunol..

[3] Chen FK (2010). Long-term outcomes following full macular translocation surgery in neovascular age-related macular degeneration. Br. J. Ophthalmol..

[4] Little CW, Cox C, Wyatt J, del Cerro C, del Cerro M (1998). Correlates of photoreceptor rescue by transplantation of human fetal RPE in the RCS rat. Exp. Neurol..

[5] Coffey PJ (2002). Long-term preservation of cortically dependent visual function in RCS rats by transplantation. Nat. Neurosci..

[6] Wang S, Lu B, Wood P, Lund RD (2005). Grafting of ARPE-19 and Schwann cells to the subretinal space in RCS rats. Invest. Ophthalmol. Vis. Sci..

[7] Alge CS (2003). Comparative proteome analysis of native differentiated and cultured dedifferentiated human RPE cells. Invest. Ophthalmol. Vis. Sci..

[8] Rabin DM, Rabin RL, Blenkinsop TA, Temple S, Stern JH (2013). Chronic oxidative stress upregulates Drusen-related protein expression in adult human RPE stem cell-derived RPE cells: a novel culture model for dry AMD. Aging (Albany NY).

[9] Garcia JM (2015). Stem cell therapy for retinal diseases. World J. Stem Cells.

[10] Lund RD (2006). Human embryonic stem cell-derived cells rescue visual function in dystrophic RCS rats. Cloning Stem Cells.

[11] Vugler A (2008). Elucidating the phenomenon of HESC-derived RPE: anatomy of cell genesis, expansion and retinal transplantation. Exp. Neurol..

[12] Ilmarinen T (2015). Ultrathin Polyimide Membrane as Cell Carrier for Subretinal Transplantation of Human Embryonic Stem Cell Derived Retinal Pigment Epithelium. PLoS One.

[13] Schwartz SD, Tan G, Hosseini H, Nagiel A (2016). Subretinal Transplantation of Embryonic Stem Cell-Derived Retinal Pigment Epithelium for the Treatment of Macular Degeneration: An Assessment at 4 Years. Invest. Ophthalmol. Vis. Sci..

[14] Cyranoski D (2014). Japanese woman is first recipient of next-generation stem cells. 12 September 2014. Nature.

[15] Coghlan, A. Mutation alert halts stem-cell trial to cure blindness. *New Scientist* https://www.newscientist.com/article/dn27986/ (31 July, 2015).

[16] Klimanskaya I (2004). Derivation and comparative assessment of retinal pigment epithelium from human embryonic stem cells using transcriptomics. Cloning Stem Cells.

[17] Carr AJ (2009). Molecular characterization and functional analysis of phagocytosis by human embryonic stem cell-derived RPE cells using a novel human retinal assay. Mol. Vis..

[18] Vaajasaari H (2011). Toward the defined and xeno-free differentiation of functional human pluripotent stem cell-derived retinal pigment epithelial cells. Mol. Vis..

[19] Juuti-Uusitalo K (2013). Aquaporin expression and function in human pluripotent stem cell-derived retinal pigmented epithelial cells. Invest. Ophthalmol. Vis. Sci..

[20] Tucker BA, Anfinson KR, Mullins RF, Stone EM, Young MJ (2013). Use of a synthetic xeno-free culture substrate for induced pluripotent stem cell induction and retinal differentiation. Stem Cells Transl. Med..

[21] Ferrer M (2014). A multiplex high-throughput gene expression assay to simultaneously detect disease and functional markers in induced pluripotent stem cell-derived retinal pigment epithelium. Stem Cells Transl. Med..

[22] Maruotti J (2015). Small-molecule-directed, efficient generation of retinal pigment epithelium from human pluripotent stem cells. Proc. Natl. Acad. Sci. USA.

[23] An E (2006). Secreted proteome profiling in human RPE cell cultures derived from donors with age related macular degeneration and age matched healthy donors. J. Proteome Res..

[24] Nordgaard CL (2006). Proteomics of the retinal pigment epithelium reveals altered protein expression at progressive stages of age-related macular degeneration. Invest. Ophthalmol. Vis. Sci..

[25] Skeie JM, Mahajan VB (2014). Proteomic landscape of the human choroid-retinal pigment epithelial complex. JAMA Ophthalmol..

[26] Novak A (2012). Proteomics profiling of human embryonic stem cells in the early differentiation stage. Stem Cell. Rev..

[27] Zheng Q (2015). iTRAQ-Based Proteomic Analysis of Visual Cycle-Associated Proteins in RPE of rd12 Mice before and after RPE65 Gene Delivery. J. Ophthalmol..

[28] Mikhailova A (2015). Comparative proteomics reveals human pluripotent stem cell-derived limbal epithelial stem cells are similar to native ocular surface epithelial cells. Sci. Rep..

[29] Graves, P. R. & Haystead, T. A. Molecular biologist’s guide to proteomics. *Microbiol*. *Mol*. *Biol*. *Rev*. **66**, 39–63, table of contents (2002). 10.1128/MMBR.66.1.39-63.2002 PMC120780 11875127

[30] West KA (2003). Protein database, human retinal pigment epithelium. Mol. Cell. Proteomics.

[31] Alge CS, Hauck SM, Priglinger SG, Kampik A, Ueffing M (2006). Differential protein profiling of primary versus immortalized human RPE cells identifies expression patterns associated with cytoskeletal remodeling and cell survival. J. Proteome Res..

[32] Zhang P (2016). Defining the proteome of human iris, ciliary body, retinal pigment epithelium, and choroid. Proteomics.

[33] Nordgaard CL, Karunadharma PP, Feng X, Olsen TW, Ferrington DA (2008). Mitochondrial proteomics of the retinal pigment epithelium at progressive stages of age-related macular degeneration. Invest. Ophthalmol. Vis. Sci..

[34] Yuan X (2010). Quantitative proteomics: comparison of the macular Bruch membrane/choroid complex from age-related macular degeneration and normal eyes. Mol. Cell. Proteomics.

[35] Arnouk H (2011). Early biosignature of oxidative stress in the retinal pigment epithelium. J. Proteomics.

[36] Crabb JW (2002). Drusen proteome analysis: an approach to the etiology of age-related macular degeneration. Proc. Natl. Acad. Sci. USA.

[37] Priglinger CS (2013). Galectin-3 induces clustering of CD147 and integrin-beta1 transmembrane glycoprotein receptors on the RPE cell surface. PLoS One.

[38] Zhou P (2014). Protection of retina by alphaB crystallin in sodium iodate induced retinal degeneration. PLoS One.

[39] Kay P (2014). Age-related changes of cystatin C expression and polarized secretion by retinal pigment epithelium: potential age-related macular degeneration links. Invest. Ophthalmol. Vis. Sci..

[40] Iannaccone A (2015). Circulating Autoantibodies in Age-Related Macular Degeneration Recognize Human Macular Tissue Antigens Implicated in Autophagy, Immunomodulation, and Protection from Oxidative Stress and Apoptosis. PLoS One.

[41] Lee H (2010). Prohibitin as an oxidative stress biomarker in the eye. Int. J. Biol. Macromol..

[42] Hadziahmetovic M (2012). Microarray analysis of murine retinal light damage reveals changes in iron regulatory, complement, and antioxidant genes in the neurosensory retina and isolated RPE. Invest. Ophthalmol. Vis. Sci..

[43] Pilat A, Herrnreiter AM, Skumatz CM, Sarna T, Burke JM (2013). Oxidative stress increases HO-1 expression in ARPE-19 cells, but melanosomes suppress the increase when light is the stressor. Invest. Ophthalmol. Vis. Sci..

[44] Kamao H (2014). Characterization of human induced pluripotent stem cell-derived retinal pigment epithelium cell sheets aiming for clinical application. Stem Cell. Reports.

[45] Sheridan, C., Hiscott, P. & Grierson, I. In *Essentials in Ophthalmology: Vitreo-Retinal Surgery* (ed. B Kirchhof, D. W.) 101–119 (Springer, Berlin, 2005).

[46] Chen MJ (2014). Ezrin is required for epithelial-mesenchymal transition induced by TGF-beta1 in A549 cells. Int. J. Oncol..

[47] Hubmacher D, Tiedemann K, Reinhardt DP (2006). Fibrillins: from biogenesis of microfibrils to signaling functions. Curr. Top. Dev. Biol..

[48] Muniz A (2014). Retinoid uptake, processing, and secretion in human iPS-RPE support the visual cycle. Invest. Ophthalmol. Vis. Sci..

[49] Fang IM, Yang CH, Yang CM, Chen MS (2009). Overexpression of integrin alpha6 and beta4 enhances adhesion and proliferation of human retinal pigment epithelial cells on layers of porcine Bruch’s membrane. Exp. Eye Res..

[50] Kevany BM, Palczewski K (2010). Phagocytosis of retinal rod and cone photoreceptors. Physiology (Bethesda).

[51] Finnemann SC, Bonilha VL, Marmorstein AD, Rodriguez-Boulan E (1997). Phagocytosis of rod outer segments by retinal pigment epithelial cells requires alpha(v) beta5 integrin for binding but not for internalization. Proc. Natl. Acad. Sci. USA.

[52] Murad N (2014). miR-184 regulates ezrin, LAMP-1 expression, affects phagocytosis in human retinal pigment epithelium and is downregulated in age-related macular degeneration. FEBS J..

[53] Strunnikova NV (2010). Transcriptome analysis and molecular signature of human retinal pigment epithelium. Hum. Mol. Genet..

[54] Ow SY (2009). iTRAQ underestimation in simple and complex mixtures: “the good, the bad and the ugly”. J. Proteome Res..

[55] Bourassa S (2015). Evaluation of iTRAQ and SWATH-MS for the Quantification of Proteins Associated with Insulin Resistance in Human Duodenal Biopsy Samples. PLoS One.

[56] Evans C (2012). An insight into iTRAQ: where do we stand now?. Anal. Bioanal Chem..

[57] Skottman H (2010). Derivation and characterization of three new human embryonic stem cell lines in Finland. In Vitro Cell. Dev. Biol. Anim..

