# Supplementary material for: Comparative proteomic analysis of human embryonic stem cell-derived and primary human retinal pigment epithelium
Source: Sci Rep. 2017 Jul 20;7:6016. doi: 10.1038/s41598-017-06233-9 (PMC5519552; doi:10.1038/s41598-017-06233-9)
Supplement: Supplementary file 1 — Supplementary Material [file 41598_2017_6233_MOESM1_ESM.pdf]

Comparative proteomic analysis of human embryonic stem cell-derived and primary human retinal pigment epithelium

Heidi Hongisto, Antti Jylhä, Janika Nättinen, Jochen Rieck, Tanja Ilmarinen, Zoltán Veréb, Ulla Aapola, Roger Beuerman, Goran Petrovski, Hannu Uusitalo, Heli Skottman

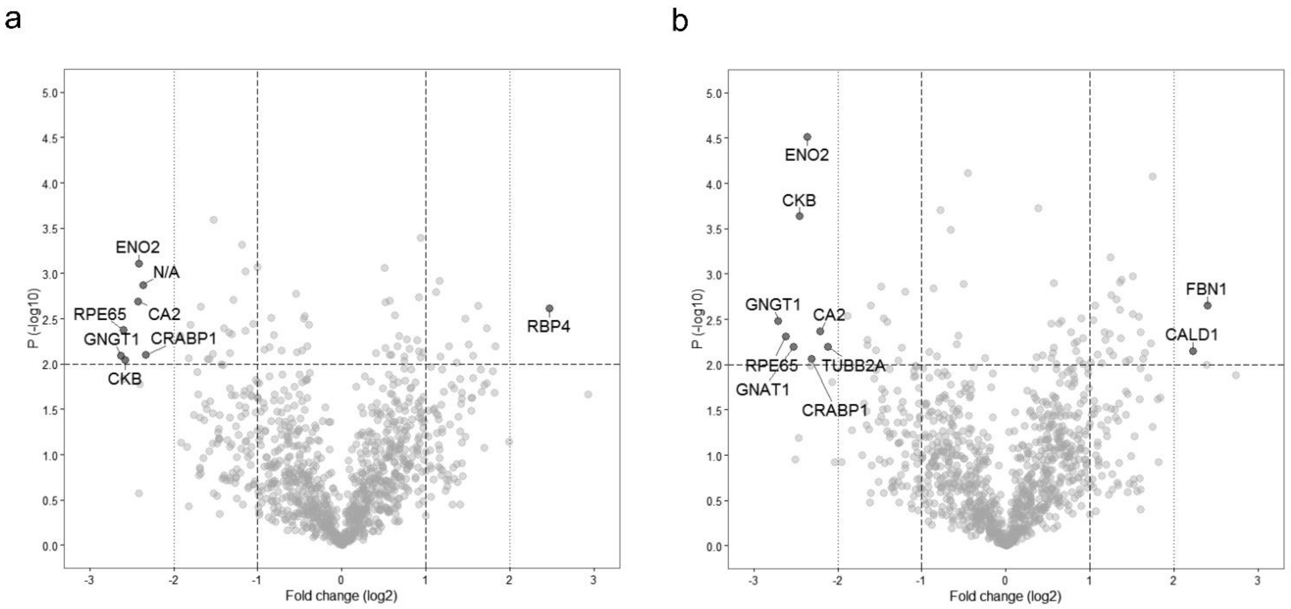

Supplementary Figure S1. Volcano plots for a) 08/017 and b) 08/023 hESC-RPE protein expression compared to the hRPE. Fold change values on log2 scale are shown on the x-axis with fold change of 1 equal to 2-fold difference in the expression (vertical dashed lines) and fold change of 2 equal to 4-fold difference in the expression (vertical dotted lines). Statistical significance (shown as negative log10 of p-value) for the difference in the expression compared to hRPE for each protein is shown on the y-axis. Student's t-test was used to measure statistical significance and p-value of 0.01 equals to the horizontal dashed line. Average values for the technical replicates were used for the statistics. Proteins with highest expression differences (with a p-value <0.01 and fold difference >4) are labeled.

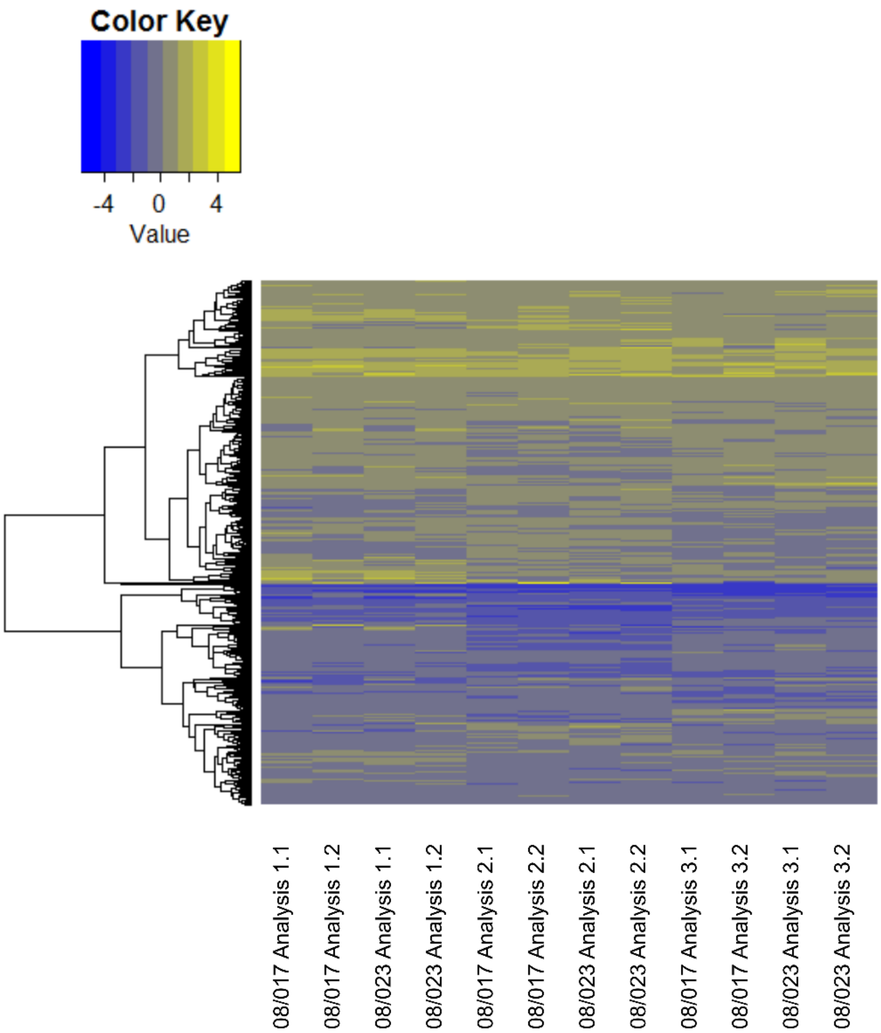

Supplementary Figure S2. A heat map showing differences in protein expression comparing the hESC-RPE samples to each of the hRPE donors showing all six technical replicates of the three MS analyses. Only proteins with complete reads (detected in all biological and technical replicates, n=431) are included in the heat map as gaps in the data would reduce readability. Log2 scale is used for the fold changes. Proteins are clustered based on expression differences (left).

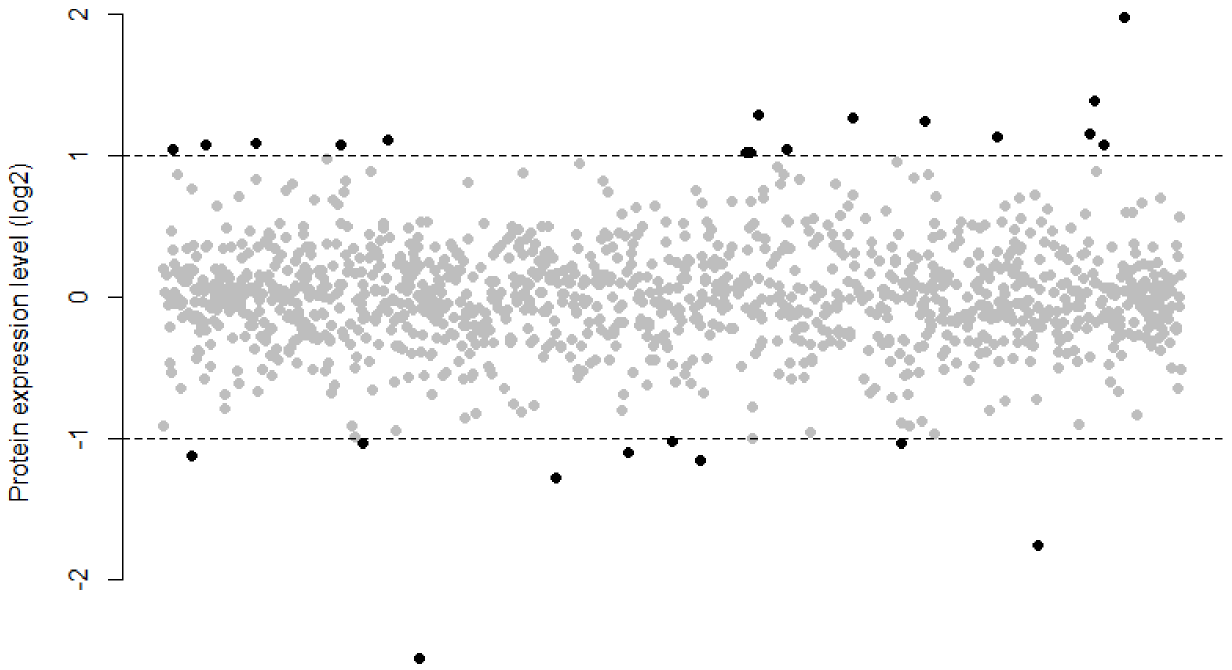

Supplementary Figure S3. A scatter plot showing expression levels of the donor sample 1 compared to pooled donor sample from all three hRPE donors on log2 scale. Two technical replicates were analysed and all proteins (also those identified in one technical replicate) were included. Average fold change was taken for those expressed in both technical replicates. Of the 1270 proteins present, only 25 (2%) were differentially expressed (2-fold difference in expression), shown in black.

a

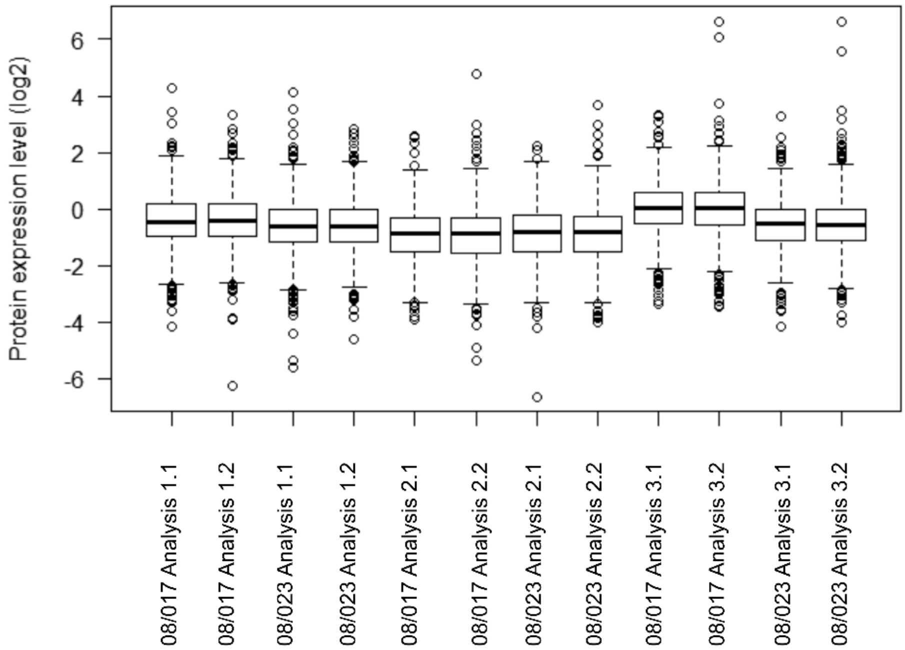

b

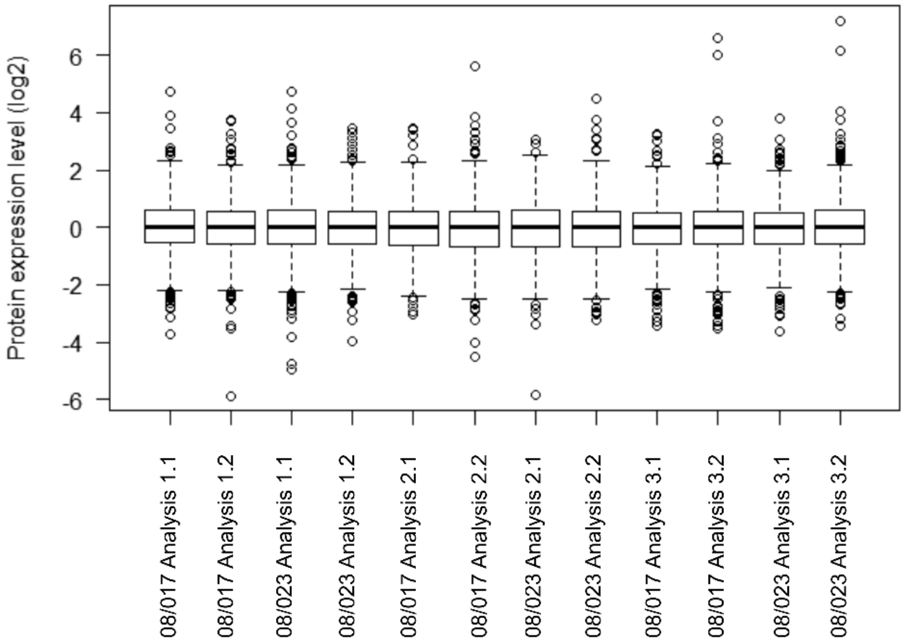

Supplementary Figure S4. Boxplots of the data on log2 scale a) before and b) after central tendency normalization showing all MS analyses and technical replicates.

Supplementary Table S1. Relative expression levels of the proteins for 08/017 and 08/023 hESC-RPE cells compared to hRPE presented for each analysis on log2 scale.
Mean number of peptides used for quantification, technical replicate CV, biological replicate CV, mean fold change, and unadjusted p-value shown for each protein.
Note! CV values are calculated using normal scale, not the log2 scale shown here. Technical replicate CV presents variability in the two MS analyses (1.1 and 1.2; 2.1 and 2.2; 3.1 and 3.2) and biological replicate CV between the biological replicate samples of hESC-RPE and hRPE (1-3).
Proteins in descending order of data completeness. Proteins filtered out shown at the end on pages 12-19.

Proteins included in the final analyses, n=1041

UniProt	Protein name	Gene name	Peptide mean	08/017 hESC-RPE to hRPE												08/023 hESC-RPE to hRPE											
				Analysis						Technical replicate CV			Biological CV	Fold change (mean)	Unadjusted p-value (t-test)	Analysis						Technical replicate CV			Biological CV	Fold change (mean)	Unadjusted p-value (t-test)
				1.1	1.2	2.1	2.2	3.1	3.2	1	2	3				1.1	1.2	2.1	2.2	3.1	3.2	1	2	3			
E9PAV3	Nascent polypeptide-associated complex subunit alpha, muscle-specific form	NACA	1.17	-0.40895	0.461363	-0.1593	0.157323	0.658828	0.700267	41.41	15.46	2.03	26.84	0.23	0.40	-0.00373	1.278032	0.404879	0.334741	0.894945	0.436132	58.99	3.44	22.30	14.18	0.56	0.03
O14745	Na(+)/H(+) exchange regulatory cofactor NHE-RF1	SLC9A3R1	8.67	1.385279	1.86852	1.280794	1.40678	1.46048	1.42337	23.47	6.17	1.82	10.85	1.47	0.00	1.407596	1.91543	1.841261	1.905157	1.435722	1.267986	24.64	3.13	8.21	17.71	1.63	0.01
O15020	Spectrin beta chain, non-erythrocytic 2	SPTBN2	7.17	0.689786	0.326325	0.678602	1.423683	0.228302	0.513301	17.72	35.73	13.92	27.85	0.64	0.09	0.787308	0.742157	1.53737	2.324298	-0.66314	0.60428	2.21	37.64	58.41	67.53	0.89	0.26
O15173	Membrane-associated progesterone receptor component 2	PGRMC2	2.33	0.567601	0.565982	0.594385	0.71805	0.475136	0.484701	0.08	6.06	0.47	6.16	0.57	0.01	0.418211	0.524127	0.312331	0.633025	0.573406	0.873194	5.19	15.65	14.64	10.46	0.56	0.02
O43707	Alpha-actinin-4	ACTN4	22.83	0.61939	0.72729	0.600866	0.524718	0.588709	0.307041	5.29	3.73	13.76	7.61	0.56	0.01	0.75638	0.750695	0.94059	0.993424	0.73618	0.90281	0.28	2.59	8.16	7.65	0.85	0.01
O75367	Core histone macro-H2A.1	H2AFY	6.67	-0.36798	-0.6498	0.234346	-0.10396	-0.27523	-0.39	13.77	16.51	5.62	21.20	-0.26	0.27	-0.30375	-0.38142	0.720371	-0.00835	-0.25936	-0.37114	3.81	34.98	5.48	31.17	-0.10	0.70
O75396	Vesicle-trafficking protein SEC22b	SEC22B	3.67	0.34501	0.377927	-0.21082	0.084603	0.705351	0.276563	1.61	14.43	20.86	19.27	0.26	0.26	-0.00747	-0.55411	0.132872	0.275097	0.681532	0.024729	26.48	6.97	31.65	21.87	0.09	0.68
O75947	ATP synthase subunit d, mitochondrial	ATP5H	10.83	-0.64311	-1.0145	-1.4123	-1.40605	-0.55535	-1.0898	18.10	0.31	25.90	22.23	-1.02	0.03	-0.72874	-0.73337	-1.41765	-1.43938	-0.17243	-0.59895	0.23	1.06	20.75	34.65	-0.85	0.11
O95336	6-phosphogluconolactonase	PGLS	2.83	1.09558	0.586227	0.790865	1.188078	2.148575	1.356725	24.71	19.35	37.86	37.71	1.19	0.05	1.056718	0.83427	0.541896	1.204035	2.60943	0.910006	10.88	31.90	74.83	46.30	1.19	0.05
O95831	Apoptosis-inducing factor 1, mitochondrial	AIFM1	10.67	-0.20686	-0.55221	-1.46109	-0.91167	-0.53779	-0.0817	16.85	26.61	22.17	29.75	-0.63	0.16	-0.07465	-0.46656	-1.32789	-0.79849	-0.46091	-0.26263	19.09	25.66	9.70	26.80	-0.57	0.15
P02489	Alpha-crystallin A chain	CRYAA	9.67	-1.10681	-1.04082	-2.43148	-2.1051	-1.53111	-1.62654	3.23	15.93	4.68	39.21	-1.64	0.04	-1.06636	-1.02826	-1.92145	-1.96239	-2.0942	-1.89325	1.87	2.01	9.83	39.66	-1.66	0.03
P02545	Prelamin-A/C	LMNA	20.50	0.108054	0.455838	0.610099	0.542927	-0.31607	-0.28296	16.96	3.29	1.62	29.08	0.19	0.54	0.028853	0.188148	0.289323	0.415668	-0.19664	-0.05199	7.80	6.19	7.08	16.48	0.11	0.50
P04040	Catalase	CAT	8.83	0.236079	-0.16365	-0.33404	-0.3794	0.07961	0.38357	19.47	2.22	14.84	20.25	-0.03	0.88	0.311725	-0.01573	0.566712	0.251973	0.487228	0.747865	15.98	15.37	12.74	15.99	0.39	0.10
P04181	Ornithine aminotransferase, mitochondrial	OAT	3.33	0.034428	0.40025	0.550178	1.888438	0.980367	0.555263	17.83	61.26	20.69	38.97	0.73	0.13	-0.22046	0.084517	0.71868	1.12038	0.546192	0.098772	14.89	19.56	21.76	35.20	0.39	0.31
P04350	Tubulin beta-4A chain	TUBB4A	46.00	0.028457	0.195182	0.536354	0.677087	0.732444	0.651165	8.16	6.89	3.98	20.27	0.47	0.12	-0.14389	0.371698	0.160078	0.459638	0.392583	-0.06179	25.01	14.63	22.09	6.59	0.20	0.08
P05023	Sodium/potassium-transporting ATPase subunit alpha-1	ATP1A1	33.17	-0.03971	0.187868	-0.12017	0.095007	0.193886	0.110032	11.13	10.53	4.11	5.58	0.07	0.27	0.025843	0.11108	-0.0338	0.067539	0.394944	0.168628	4.18	4.96	11.07	10.10	0.12	0.27
P05109	Protein S100-A8	S100A8	1.33	-0.73218	-0.64801	-1.5924	-1.54839	-2.52185	-2.3978	4.12	2.16	6.08	58.53	-1.57	0.09	-0.49832	0.144159	-1.4136	-1.15237	-2.75298	-2.29615	30.98	12.77	22.21	74.79	-1.33	0.19
P05556	Integrin beta-1	ITGB1	2.33	0.149462	-0.28122	0.62839	0.613721	0.735134	0.260371	20.95	0.72	23.06	23.18	0.35	0.24	-0.25717	-0.68085	0.93993	0.767182	0.983781	0.313317	20.62	8.46	32.28	41.53	0.34	0.49
P05787	Keratin, type II cytoskeletal 8	KRT8	17.50	1.785737	1.388262	1.527923	1.812191	1.052638	1.506304	19.36	13.89	22.05	13.42	1.51	0.01	1.86948	1.38205	1.650753	1.886236	0.998759	1.810548	23.67	11.52	38.77	10.76	1.60	0.00
P06737	Glycogen phosphorylase, liver form	PYGL	16.50	-0.67827	-0.33887	-0.4408	-0.65934	-0.8055	-0.36631	16.56	10.69	21.36	2.50	-0.55	0.00	-0.52677	-0.29156	-1.04394	-0.71162	-1.23945	-1.01301	11.50	16.22	11.08	25.86	-0.80	0.06
P06748	Nucleophosmin	NPM1	7.67	0.599781	0.343483	1.322935	0.611135	0.635892	0.643133	12.53	34.20	0.35	19.48	0.69	0.04	0.658437	0.706411	1.048766	0.292303	0.114252	0.503375	2.35	36.25	18.96	14.53	0.55	0.05
P07305	Histone H1.0	H1F0	2.17	-2.02482	-2.14108	-0.44693	-0.69816	-1.0726	-1.12142	5.70	12.28	2.39	47.76	-1.25	0.11	-1.58474	-1.29022	-0.69564	-0.69477	-1.5761	-1.44209	14.39	0.04	6.56	33.18	-1.21	0.04
P07737	Profilin-1	PFN1	10.00	0.757371	0.545318	0.405597	0.595168	1.437388	0.893868	10.37	9.28	26.33	26.22	0.77	0.06	0.731521	0.68738	1.107846	1.253985	1.554453	0.934521	2.16	7.16	29.93	19.82	1.04	0.03
P07919	Cytochrome b-c1 complex subunit 6, mitochondrial	UQCRH	2.33	-0.66822	-1.22379	-1.26872	-1.68158	-0.0779	-0.8711	26.90	20.10	37.93	35.22	-0.97	0.08	-1.07742	-0.93389	-1.01552	-1.43187	-0.04239	-0.70454	7.03	20.27	31.90	33.33	-0.87	0.08
P07954	Fumarate hydratase, mitochondrial	FH	8.33	-0.45165	-0.2338	-1.80563	-0.95497	-0.94521	-0.43879	10.66	40.53	24.57	32.27	-0.81	0.12	-0.25247	-0.15232	-1.66242	-1.00126	-1.10661	-0.8117	4.91	31.85	14.40	40.36	-0.83	0.13
P08708	40S ribosomal protein S17	RPS17	2.67	1.938882	0.249525	0.234346	0.155194	0.515203	-0.62458	74.48	3.88	53.13	52.47	0.41	0.36	1.060611	0.031603	-0.34707	-0.05504	0.053981	-1.23641	48.40	14.26	59.34	41.73	-0.08	0.83
P09382	Galectin-1	LGALS1	6.83	0.763182	0.70359	0.594034	0.297339	0.751501	0.874264	2.92	14.49	6.01	12.70	0.66	0.03	0.638025	0.687653	1.158265	0.737521	1.134361	1.219714	2.43	20.48	4.18	17.59	0.93	0.02
P09417	Dihydropteridine reductase	QDPR	4.00	-0.10068	-0.28538	-0.46511	0.14068	-0.17383	0.147772	9.04	29.26	15.70	6.65	-0.12	0.16	-0.2836	-0.04226	-0.31209	-0.07271	-0.39064	0.13178	11.80	11.71	25.33	2.87	-0.16	0.01
P10606	Cytochrome c oxidase subunit 5B, mitochondrial	COX5B	5.00	-0.76492	-0.42163	-1.31545	-1.22569	-0.37431	-0.32111	16.75	4.40	2.61	30.45	-0.74	0.12	-0.81708	-0.40137	-1.24758	-0.97771	-0.5761	-0.4915	20.24	13.19	4.15	20.36	-0.75	0.05
P10745	Retinol-binding protein 3	RBP3	23.67	-2.2667	-1.0768	-1.76093	-1.6656	-1.88047	-2.15628	55.22	4.67	13.48	15.74	-1.80	0.00	-2.44873	-1.2085	-1.94952	-1.54514	-1.85102	-2.31733	57.30	19.69	22.66	13.43	-1.89	0.00
P11177	Pyruvate dehydrogenase E1 component subunit beta, mitochondrial	PDHB	9.50	-0.17273	-0.02528	-1.17238	-0.63089	-0.84589	-1.00085	7.22	26.23	7.59	34.40	-0.64	0.14	-0.25273	0.014253	-0.7705	-0.72699	-1.08935	-0.66022	13.05	2.13	20.88	29.55	-0.58	0.13
P11216	Glycogen phosphorylase, brain form	PYGB	10.83	-0.16985	-0.32193	0.151579	0.173309	-0.59661	-0.02021	7.45	1.07	27.88	17.75	-0.13	0.47	-0.47104	-0.32568	-0.24278	0.048144	-0.07817	-0.41786	7.12	14.21	16.57	10.59	-0.25	0.10
P12235	ADP/ATP translocase 1	SLC25A4	14.50	-0.24764	-0.59029	-2.06035	-1.51109	-1.47486	-0.79508	16.72	26.60	32.71	45.75	-1.11	0.11	-0.25221	-0.62269	-2.33107	-1.79629	-1.32027	-0.86412	18.06	25.91	22.17	51.45	-1.20	0.13
P12271	Retinaldehyde-binding protein 1	RLBP1	19.50	-0.85143	-0.83922	-1.04934	-0.91068	-1.32655	-1.45245	0.60	6.79	6.17	18.67	-1.07	0.02	-0.8373	-0.7701	-1.1841	-1.14454	-1.13222	-0.86295	3.29	1.94	13.16	12.52	-0.99	0.01
P12532	Creatine kinase U-type, mitochondrial	CKMT1A	14.00	-1.43373	-0.96512	-3.03515	-2.8866	-1.04378	-1.70639	22.77	7.27	31.92	52.47	-1.85	0.08	-1.41972	-0.94276	-2.85861	-2.4881	-1.38739	-1.9238	23.17	18.06	25.99	46.86	-1.84	0.05
P13010	X-ray repair cross-complementing protein 5	XRCC5	10.83	-0.45948	-0.18875	0.546371	0.330423	-0.53273	-0.66524	13.23	10.56	6.49	39.23	-0.16	0.65	-0.52016	-0.26802	0.286213	-0.00759	-0.74741	-0.75676	12.33	14.35	0.46	31.83	-0.34	0.32
P13073	Cytochrome c oxidase subunit 4 isoform 1, mitochondrial	COX4I1	3.83	-0.70497	-1.24883	-1.28477	-1.50434	-1.08174	-0.73786	26.34	10.74	16.78	17.63	-1.09	0.02	-0.81284	-1.18878	-1.23383	-1.16816	-0.71854	-0.57132	18.32	3.22	7.21	19.84	-0.95	0.03
P13639	Elongation factor 2	EEF2	13.67	0.923397	1.02176	0.157294	0.676922	0.698563	1.171119	4.82	25.20	22.96	19.56	0.77	0.05	1.022826	1.130535	0.191886	0.720622	0.88423	1.240613	5.28	25.63	17.38	22.00	0.87	0.05
P13804	Electron transfer flavoprotein subunit alpha, mitochondrial	ETFA	9.67	0.373704	0.211832	-0.16137	-0.45617	-0.5821	-0.37087	7.93	14.40	10.33	29.35	-0.16	0.55	0.517681	0.0496	-0.45741	-0.69151	-0.41029	-0.15836	22.74	11.45	12.32	31.81	-0.19	0.53
P14854	Cytochrome c oxidase subunit 6B1	COX6B1	3.67	-0.46899	-0.75287	-0.77401	-0.95957	-0.60445	-0.66748	13.87	9.08	3.09	9.55	-0.70	0.01	-0.70675	-0.57628	-1.06814	-1.2125	-0.21021	-0.2875	6.39	7.07	3.79	30.01	-0.68	0.12
P16152	Carbonyl reductase [NADPH] 1	CBR1	7.00	0.798583	0.41499	-0.27737	0.119481	0.37003	0.393179	18.69	19.33	1.13	23.10	0.30	0.27	0.59834	0.263512	0.381176	0.423404	0.157164	0.211232	16.34	2.07	2.65	9.32	0.34	0.05
P16499	Rod cGMP-specific 3',5'-cyclic phosphodiesterase subunit alpha	PDE6A	16.67	-1.19825	-1.05101	-1.76093	-1.48946	-0.80233	-1.02078	7.21	13.27	10.69	23.94	-1.22	0.03	-1.40282	-0.90307	-1.57236	-1.65291	-0.45239	-1.39741	24.25	3.95	44.73	25.63	-1.23	0.03
P17174	Aspartate aminotransferase, cytoplasmic	GOT1	7.83	0.029036	-0.29769	-0.30887	-0.28516	-0.39677	-0.12081	15.95	1.16	13.48	6.26	-0.23	0.04	0.127629	-0.05915	-0.33139	0.159654	-0.28055	-0.303	9.14	23.84	1.10	11.41	-0.11	0.35
P17643	5,6-dihydroxyindole-2-carboxylic acid oxidase	TYRP1	5.00	1.903488	1.885831	0.852643	1.555717	0.947329	1.357277	0.87	33.79	19.96	29.37	1.42	0.03	1.238022	1.159827	0.815897	0.686062	0.527665	1.128748	3.83	6.36	29.04	16.69	0.93	0.02
P17858	ATP-dependent 6-phosphofructokinase, liver type	PFKL	7.83	0.191439	0.11787	0.075391	0.364814	0.048183	0.242921	3.61	14.14	9.53	3.05	0.17	0.02	0.066377	0.312776	0.29614	0.323895	0.175406	0.311593	12.05	1.36	6.67	4.01	0.25	0.02
P17987	T-complex protein 1 subunit alpha	TCP1	8.50	-0.18097	-0.30213	-0.04059	-0.07285	-0.03059	-0.03635	5.94	1.58	0.28	7.68	-0.11	0.24	-0.2118	0.024255	0.086435	-0.05137	-0.724	-0.62876	11.54	6.75	4.67	23.93	-0.25	0.36
P21281	V-type proton ATPase subunit B, brain isoform	ATP6V1B2	12.33	-0.24158	-0.6087	-0.20165	-0.64155	-0.23832	-0.35163	17.90	21.40	5.55	4.68	-0.38	0.01	-0.5772	-0.34207	-0.1472	-0.37383	-0.25023	0.050386	11.50	11.08	14.68	12.49	-0.27	0.12
P21796	Voltage-dependent anion-selective channel protein 1	VDAC1	29.00	-1.46926	-1.38399	-2.06146	-2.12681	-1.14001	-1.03722	4.18	3.20	5.04	33.06	-1.54	0.04	-1.38325	-1.35695	-2.00838	-1.99421	-0.99278	-0.87351	1.29	0.69	5.84	35.46	-1.43	0.04
P23396	40S ribosomal protein S3	RPS3	5.50	0.368071	0.286259	0.250701	0.427314	0.435787	0.705996	4.01	8.65	13.21	9.97	0.41	0.04	0.309782	0.47424	0.295315	0.285072	0.533677	0.559221	8.05	0.50	1.25	9.00	0.41	0.03
P23526	Adenosylhomocysteinase	AHCY	6.67	0.685631	0.554266	0.520551	0.544193	0.365263	0.760989	6.43	1.16	19.27	3.09	0.57	0.00	0.463728	0.538611	0.34597	0.67804	0.252589	0.250455	3.67	16.20	0.10	10.11	0.42	0.04
P24752	Acetyl-CoA acetyltransferase, mitochondrial	ACAT1	10.50	0.839969	0.62773	-0.12017	0.124568	0.739578	0.174817	10.38	11.97	27.33	24.55	0.40	0.20	0.670465	0.348867	-0.39286	0.105563	0.501028	0.128062	15.70	24.19	18.18	21.71	0.23	0.36
P25398	40S ribosomal protein S12	RPS12	5.00	0.486186	0.343333	0.598767	0.573719	1.751044	1.12715	7.00	1.23	30.11	42.13	0.81	0.12	0.490489	0.468199	0.611458	0.745069	1.271642	0.958627	1.09	6.54	15.28	23.57	0.76	0.06
P27824	Calnexin	CANX	19.33	-0.57701	-0.79507	-0.82335	-1.04729	-0.43614	-0.37508	10.67	10.95	2.99	18.24	-0.68	0.05	-0.46135	-0.4871	-0.80989	-0.95503	-0.45551	-0.61624	1.26	7.11	7.87	14.55	-0.63	0.04
P30048	Thioredoxin-dependent peroxide reductase, mitochondrial	PRDX3	7.00	0.018969	0.552972	-0.3699	-0.44285	-0.01949	0.460563	25.88	3.57	23.31	25.02	0.03	0.89	0.257033	0.357665	-0.47102	-0.53706	-0.1736	0.363605	4.93	3.24	26.03	27.18	-0.03	0.90
P30049	ATP synthase subunit delta, mitochondrial	ATP5D	4.83	-0.33756	-0.58316	-1.65781	-1.55225	-0.32988	-0.15015	12.01	5.17	8.80	42.82	-0.77	0.21	0.102338	-0.06906	-1.42239	-1.28998	-0.16569	0.046867	8.39	6.49	10.40	43.80	-0.47	0.41
P30084	Enoyl-CoA hydratase, mitochondrial	ECHS1	7.67	0.39047	0.325417	-0.67336	-0.51787	-0.08545	-0.6211	3.19	7.61	25.96	35.66	-0.20	0.56	0.401885	0.365041	-0.80104	-0.46076	0.326182	0.698982	1.81	16.60	18.17	37.29	0.09	0.83
P31150	Rab GDP dissociation inhibitor alpha	GDI1	9.00	0.267489	-1.00152	0.191817	-0.43249	-0.43311	-0.18716	58.48	30.13	12.03	7.78	-0.27	0.07	0.013088	-0.22309	0.089768	0.237062	0.162162	-0.53551	11.55	7.21	33.54	11.95	-0.04	0.73
P31930	Cytochrome b-c1 complex subunit 1, mitochondrial	UQCRC1	15.33	-0.33012	-0.91594	-1.29384	-1.40256	-0.92862	-0.61308	28.32	5.33	15.40	25.43	-0.91	0.05	-0.47682	-0.94106	-1.3052	-1.29369	-0.89687	-0.29904	22.56	0.56	28.89	25.02	-0.87	0.06
P35555	Fibrillin-1	FBN1	25.83	1.607747	0.988756	1.317947	1.315035	1.849868	2.22429	29.88	0.14	18.25	30.98	1.55	0.02	2.635805	2.18955	2.497953	2.681427	1.935242	2.457065	21.70	8.98	25.30	12.83	2.40	0.00
P35573	Glycogen debranching enzyme	AGL	4.83	1.265198	-0.66387	-1.03298	-0.99907	-0.84062	-0.80048	82.59	1.66	1.97	66.44	-0.51	0.34	1.177211	-0.21693	-0.69318	-1.74972	-0.72331	-0.92909	63.47	49.59	10.07	70.60	-0.52	0.42
P35580	Myosin-10	MYH10	10.17	1.357816	1.470366	0.552529	0.574781	0.442283	0.37564	5.51	1.09	3.27	40.21	0.80	0.13	1.376116	1.211269	0.285798	1.163715	0.740394	0.386223	8.07	41.75	17.27	26.61	0.86	0.06
P35913	Rod cGMP-specific 3',5'-cyclic phosphodiesterase subunit beta	PDE6B	13.17	-1.48295	-1.20485	-1.79916	-1.8682	-2.15332	-1.80665	13.59	3.38	16.91	24.11	-1.72	0.01	-1.6153	-1.28323	-1.61591	-1.60376	-2.01434	-1.77057	16.20	0.60	11.92	15.33	-1.65	0.01
P36955	Pigment epithelium-derived factor	SERPINF1	3.17	0.815571	1.024376	0.644927	0.269614	0.412975	0.25185	10.22	18.29	7.89	22.27	0.57	0.09	0.730729	0.683406	0.197634	0.607249	0.361375	0.200093	2.32	19.94	7.90	15.37	0.46	0.07
P37837	Transaldolase	TALDO1	12.00	-0.78276	-0.97031	0.035605	0.034261	-0.8131	-0.83536	9.18	0.07	1.09	38.09	-0.56	0.20	-0.63388	-0.93473	0.158719	-0.0831	-1.1643	-1.01431	14.69	11.82	7.34	42.58	-0.61	0.21
P38117	Electron transfer flavoprotein subunit beta	ETFB	5.67	0.516914	0.345426	-0.12507	-0.07257	0.478451	0.462513	8.40	2.57	0.78	20.54	0.27	0.28	0.457997	0.391824	-0.18267	0.117243	-0.1736	0.37768	3.24	14.65	26.70	16.19	0.16	0.35
P38606	V-type proton ATPase catalytic subunit A	ATP6V1A	8.67	-0.98726	-0.83109	-0.41689	-0.34518	-0.77265	-0.99126	7.65	3.51	10.69	21.55	-0.72	0.05	-1.06087	-0.70939	-0.40085	-0.29648	-0.74083	-0.72244	17.14	5.11	0.90	19.49	-0.66	0.05
P42126	Enoyl-CoA delta isomerase 1, mitochondrial	ECI1	4.00	-0.04763	-0.34152	-1.34814	-1.21647	-0.25241	-0.84092	14.35	6.45	28.45	35.52	-0.67	0.17	-0.09697	-0.37369	-1.47418	-1.29431	0.00619	-0.80532	13.52	8.80	38.76	37.68	-0.67	0.20
P47804	RPE-retinal G protein-coupled receptor	RGR	6.83	-1.64711	-1.27523	-1.78265	-2.11992	-0.76124	-1.16513	18.13	16.46	19.67	33.94	-1.46	0.04	-1.55482	-1.41394	-1.64174	-2.03396	-0.59097	-0.81132	6.90	19.11	10.78	41.88	-1.34	0.06
P47985	Cytochrome b-c1 complex subunit Rieske, mitochondrial	UQCRFS1	5.00	-0.76995	-0.61653	-1.01519	-1.12796	-0.37976	-0.947	7.51	5.52	27.45	15.61	-0.81	0.03	-0.8883	-0.52148	-1.27917	-1.32496	0.105053	-0.40561	17.88	2.24	24.77	39.60	-0.72	0.16
P48047	ATP synthase subunit O, mitochondrial	ATP5O	11.17	-0.04458	-0.27915	-1.37058	-1.28548	0.019922	0.208476	11.47	4.17	9.23	44.70	-0.46	0.41	0.060716	-0.0603	-1.12717	-1.06477	-0.11667	0.493372	5.93	3.06	29.46	41.51	-0.30	0.53
P49411	Elongation factor Tu, mitochondrial	TUFM	7.00	1.405706	1.057313	0.176419	0.610099	0.555518	-0.2749	16.99	21.10	39.61	40.71	0.59	0.22	1.385121	0.886459	0.477379	0.577795	0.31874	-0.22011	24.20	4.92	26.11	38.08	0.57	0.21
P49458	Signal recognition particle 9 kDa protein	SRP9	2.17	-0.31241	-0.88897	0.619446	0.082862	-0.26646	0.150321	27.89	26.00	20.29	31.89	-0.10	0.75	-0.48324	-0.77311	0.237676	0.245814	-0.06211	-0.08454	14.16	0.40	1.10	28.76	-0.15	0.61
P49588	Alanine--tRNA ligase, cytoplasmic	AARS	6.83	-0.45273	-0.78097	0.343657	0.68071	0.768259	0.418144	16.02	16.45	17.08	39.55	0.16	0.72	0.089833	-0.2886	-0.1025	0.340532	0.707596	0.04666	18.44	21.55	31.84	17.52	0.13	0.44
P50991	T-complex protein 1 subunit delta	CCT4	7.17	0.487731	-0.41681	0.15872	0.487031	-0.52185	0.001555	42.94	16.02	25.38	19.36	0.03	0.86	0.578667	-0.61053	-0.04315	-0.12569	-0.55772	-0.19416	55.20	4.04	17.73	16.01	-0.16	0.29
P51149	Ras-related protein Rab-7a	RAB7A	7.83	0.421519	-0.15053	0.05206	-0.02379	0.85258	0.739988	27.68	3.72	5.52	30.42	0.32	0.32	0.344193	-0.10273	-0.483	-0.39989	-0.0303	-0.24147	21.73	4.07	10.33	19.84	-0.15	0.45
P52209	6-phosphogluconate dehydrogenase, decarboxylating	PGD	9.67	0.31114	0.357182	0.458636	0.05674	-0.18618	-0.4034	2.26	19.57	10.63	22.09	0.10	0.67	0.180018	0.437775	0.167082	0.142839	-0.06341	-0.29247	12.60	1.19	11.20	16.71	0.10	0.58
P54868	Hydroxymethylglutaryl-CoA synthase, mitochondrial	HMGCS2	9.33	1.134297	0.95498	1.156106	1.2448	1.098142	1.145154	8.78	4.35	2.30	5.33	1.12	0.00	0.57205	0.602847	0.340584	0.736461	0.205486	0.749139	1.51	19.28	26.34	2.94	0.53	0.00
P55084	Trifunctional enzyme subunit beta, mitochondrial	HADHB	12.00	0.676915	0.442769	0.232768	0.474563	0.65475	0.450462	11.45	11.82	10.00	7.88	0.49	0.02	0.717464	0.350597	0.140239	0.326916	0.649857	0.717328	17.88	9.14	3.31	15.39	0.48	0.07
P56385	ATP synthase subunit e, mitochondrial	ATP5I	1.33	-0.01206	-0.12236	-0.72588	-0.26982	-0.65925	-0.27507	5.40	22.17	18.72	16.73	-0.34	0.13	-0.17201	0.196057	-0.52462	-0.34726	0.302401	0.063148	17.94	8.68	11.70	21.14	-0.08	0.71
P61026	Ras-related protein Rab-10	RAB10	3.33	0.126744	-0.41605	-0.50217	-0.53343	-1.49994	-1.09492	26.29	1.53	19.72	37.77	-0.65	0.19	-0.26532	-0.34738	-1.2706	-0.7493	-1.10617	-0.3128	4.02	25.28	37.93	23.51	-0.68	0.08
P61981	14-3-3 protein gamma	YWHAG	8.67	-0.27002	-0.57918	0.147277	-0.10255	-1.99226	-2.10735	15.10	12.21	5.64	58.92	-0.82	0.32	-0.01542	-0.1718	0.166406	-0.04407	-0.27404	-0.74559	7.66	10.30	22.91	18.99	-0.18	0.40
P62081	40S ribosomal protein S7	RPS7	3.17	0.091152	-0.48638	0.329805	0.611825	-0.26025	-0.17403	27.93	13.78	4.22	28.55	0.02	0.94	0.200757	-0.28108	-0.20379	0.264212	0.421125	0.54711	23.40	22.74	6.17	19.84	0.16	0.44
P62269	40S ribosomal protein S18	RPS18	2.00	-0.26457	-1.09899	0.46978	0.432207	0.246209	0.454387	39.79	1.84	10.19	35.61	0.04	0.92	-0.02141	-0.75032	0.44447	0.564888	0.357991	0.214372	34.98	5.90	7.03	28.17	0.13	0.66
P62851	40S ribosomal protein S25	RPS25	1.17	-1.187	-1.01565	-0.46621	-0.35762	0.51393	0.176944	8.39	5.32	16.44	49.49	-0.39	0.45	-0.37613	-0.22129	0.389121	0.084298	0.158646	-0.32295	7.58	14.88	23.39	18.96	-0.05	0.79
P63211	Guanine nucleotide-binding protein G(T) subunit gamma-T1	GNGT1	7.67	-2.13723	-2.4447	-2.92436	-3.24056	-2.59344	-2.40752	15.01	15.44	9.10	26.46	-2.62	0.01	-2.36508	-2.48063	-2.70551	-3.21895	-2.83175	-2.6281	5.66	24.90	9.97	18.24	-2.71	0.00
P63241	Eukaryotic translation initiation factor 5A-1	EIF5A	4.83	-0.60968	-1.78588	0.27872	-0.239	-1.22535	-0.63705	54.65	25.11	28.44	44.95	-0.70	0.20	-0.59232	-0.10273	0.372797	0.165066	-0.16918	-0.05955	23.77	10.16	5.37	21.53	-0.06	0.75

UniProt	Protein name	Gene name	Peptide mean	08/017 hESC-RPE to hRPE												08/023 hESC-RPE to hRPE											
				Analysis						Technical replicate CV			Biological	Fold change (mean)	Unadjusted p-value	Analysis						Technical replicate CV			Biological	Fold change (mean)	Unadjusted p-value
				1.1	1.2	2.1	2.2	3.1	3.2	1	2	3	CV		(t-test)	1.1	1.2	2.1	2.2	3.1	3.2	1	2	3	CV		(t-test)
P68366	Tubulin alpha-4A chain	TUBA4A	38.00	-1.32715	-1	-0.75421	-0.2128	-1.78633	-1.73904	15.97	26.23	2.32	44.74	-1.14	0.09	-1.45713	-1.18026	-0.16298	-0.45833	-1.07534	-1.63473	13.53	14.43	27.08	44.07	-0.99	0.10
P78363	Retinal-specific ATP-binding cassette transporter	ABCA4	23.00	-0.59029	-0.29793	-1.20439	-1.87592	-0.25724	-2.21216	14.28	32.33	83.43	35.81	-1.07	0.08	-1.27589	-0.31303	-1.23204	-1.6753	0.170454	-0.69376	45.52	21.56	41.14	40.75	-0.84	0.14
P78527	DNA-dependent protein kinase catalytic subunit	PRKDC	6.50	-0.64218	0.24184	0.319222	0.138766	0.028582	-0.36686	42.02	8.83	19.26	15.67	-0.05	0.77	0.210069	-0.74403	-0.61591	-0.2649	0.19143	-0.38673	45.13	17.12	27.96	12.46	-0.27	0.11
P80723	Brain acid soluble protein 1	BASP1	8.17	-0.8852	-0.55416	-0.64573	-0.58707	-1.02119	-1.37307	16.15	2.87	17.16	19.91	-0.84	0.04	-0.77561	-0.37684	-0.19333	-0.21865	-0.8713	-1.45201	19.42	1.24	28.08	30.84	-0.65	0.15
Q01813	ATP-dependent 6-phosphofructokinase, platelet type	PFKP	10.50	-0.13809	-0.32359	0.372812	0.09772	0.5848	0.737616	9.08	13.44	7.48	30.29	0.22	0.48	-0.26321	-0.09424	0.137711	-0.07298	0.403411	0.230156	8.27	10.31	8.48	17.38	0.06	0.73
Q02952	A-kinase anchor protein 12	AKAP12	5.83	-1.04377	-0.16514	-1.56313	-0.78589	-1.69751	-0.55068	41.78	37.20	53.43	22.50	-0.97	0.03	-0.54296	-0.41012	-0.86459	-0.27892	-1.33585	-1.19465	6.51	28.32	6.92	27.36	-0.77	0.09
Q03395	Rod outer segment membrane protein 1	ROM1	6.83	-0.35609	-0.71779	-1.78174	-2.03104	-0.14047	-0.26858	17.64	12.19	6.28	50.66	-0.88	0.23	-0.5603	-0.89403	-1.90999	-2.04433	-1.56749	-0.69758	16.28	6.58	41.39	40.07	-1.28	0.07
Q07954	Prolow-density lipoprotein receptor-related protein 1	LRP1	2.50	0.663307	0.675935	-0.04332	0.42437	0.532895	0.159588	0.62	22.72	18.20	16.65	0.40	0.10	0.696389	0.197595	-0.02888	-0.30674	0.982944	0.239434	24.21	13.58	35.66	27.42	0.30	0.34
Q07960	Rho GTPase-activating protein 1	ARHGAP1	6.17	0.852234	1.03015	1.114845	0.585538	0.143268	0.713686	8.71	25.66	27.60	17.30	0.74	0.04	0.941074	0.71771	0.721292	0.151498	0.844247	1.472307	10.93	27.57	30.31	24.98	0.81	0.06
Q08211	ATP-dependent RNA helicase A	DHX9	7.83	0.265362	0.161633	-0.15723	0.47608	-0.06293	-0.23659	5.08	30.55	8.50	13.47	0.07	0.58	0.206655	0.397523	-0.08388	-0.17869	-0.33637	-0.23793	9.34	4.65	4.82	22.03	-0.04	0.85
Q09666	Neuroblast differentiation-associated protein AHNAK	AHNAK	21.50	-0.04722	-0.2145	-0.17597	-0.14069	-0.65617	-0.52056	8.19	1.73	6.64	16.80	-0.29	0.19	0.058403	-0.11892	0.66734	0.7961	-0.07927	-0.08045	8.68	6.31	0.06	33.74	0.21	0.51
Q12797	Aspartyl/asparaginyl beta-hydroxylase	ASPH	4.67	-0.57878	-0.62675	-0.11098	-0.38078	-0.92013	-1.06416	2.35	13.19	7.05	25.70	-0.61	0.10	-0.05403	-0.5632	-0.43466	-0.57904	-0.36831	-0.4915	24.70	7.07	6.03	7.77	-0.42	0.02
Q13011	Delta(3,5)-Delta(2,4)-dienoyl-CoA isomerase, mitochondrial	ECH1	7.67	0.900696	0.954098	0.323253	0.249797	0.304645	1.382594	2.62	3.60	50.51	23.76	0.69	0.08	1.001861	1.141964	-0.0812	-0.30987	0.321722	1.126298	6.86	11.18	38.44	40.11	0.53	0.29
Q13885	Tubulin beta-2A chain	TUBB2A	44.67	-2.39853	-1.94854	-1.88467	-1.53993	-1.81408	-1.95273	21.88	16.82	6.79	15.55	-1.92	0.00	-2.43564	-2.43821	-2.15959	-1.91594	-1.60286	-2.12856	0.13	11.91	25.48	20.21	-2.11	0.01
Q14204	Cytoplasmic dynein 1 heavy chain 1	DYNC1H1	19.83	-0.0077	0.604321	-0.38124	1.450071	0.06592	0.415921	29.55	79.38	17.07	21.84	0.36	0.06	-0.00791	0.922597	-0.80192	1.248047	0.365554	0.335884	44.09	86.40	1.45	7.69	0.34	0.04
Q14240	Eukaryotic initiation factor 4A-II	EIF4A2	5.17	0.214186	0.451124	0.422901	0.639504	0.273643	0.105835	11.59	10.60	8.22	12.02	0.35	0.07	0.213853	0.228016	0.328342	0.444512	0.396359	0.077016	0.69	5.69	15.59	6.33	0.28	0.03
Q14257	Reticulocalbin-2	RCN2	4.67	1.195828	0.223748	0.454193	-0.05248	-0.38961	-0.01906	45.92	24.58	18.06	34.53	0.24	0.47	0.008085	0.026853	0.346965	0.461838	0.194866	0.104956	0.92	5.63	4.41	13.91	0.19	0.24
Q14894	Ketimine reductase mu-crystallin	CRYM	11.67	-0.86463	-1.37756	-1.5491	-1.54607	-1.75674	-1.36213	24.88	0.15	19.22	18.76	-1.41	0.01	-0.75734	-1.10344	-1.65602	-1.83448	-2.00435	-1.4042	16.88	8.74	29.00	33.80	-1.46	0.03
Q15084	Protein disulfide-isomerase A6	PDIA6	11.33	0.975682	1.075533	0.578321	0.601958	0.943917	0.659958	4.89	1.16	13.87	15.07	0.81	0.02	0.944266	0.906229	0.374164	0.374021	0.886021	0.72019	1.86	0.01	8.12	19.04	0.70	0.05
Q15149	Plectin	PLEC	25.83	0.608716	0.748574	0.569239	0.913077	0.521546	0.599028	6.85	16.77	3.80	6.62	0.66	0.01	0.418539	0.92492	0.79315	1.175829	0.677529	0.8457	24.57	18.65	8.23	11.26	0.81	0.01
Q16518	Retinoid isomerohydrolase	RPE65	24.67	-2.31095	-2.40194	-3.02432	-2.82179	-2.6966	-2.3266	4.46	9.91	18.04	19.32	-2.60	0.00	-2.48018	-2.37398	-3.01347	-2.94966	-2.64686	-2.2344	5.20	3.13	20.08	20.64	-2.62	0.00
Q5KU26	Collectin-12	COLEC12	2.67	1.855229	1.554266	0.775154	1.569551	1.270517	0.994904	14.70	37.98	13.47	22.44	1.34	0.02	2.15145	1.633213	1.202811	2.124668	1.788893	1.007687	25.13	43.71	37.38	15.91	1.65	0.01
Q6ZVX7	F-box only protein 50	NCCRP1	2.33	1.517464	1.042644	1.845506	1.644994	0.370895	2.912346	23.06	9.81	99.96	28.60	1.56	0.01	2.369177	1.450086	1.052187	1.605343	0.454857	2.690151	43.58	26.78	91.87	22.82	1.60	0.01
Q71UI9	Histone H2A.V	H2AFV	5.17	-1.90719	-2.24613	-1.28671	-1.74821	-0.97512	-0.66367	16.54	22.43	15.21	43.41	-1.47	0.06	-1.62405	-1.8822	-0.81879	-1.61531	-1.63911	-1.69723	12.62	38.08	2.85	23.08	-1.55	0.01
Q8NBX0	Saccharopine dehydrogenase-like oxidoreductase	SCCPDH	6.00	-0.63789	-0.15877	-0.81773	-0.83631	-0.76955	-0.05429	23.27	0.91	34.36	17.12	-0.55	0.06	-0.55	-0.19431	-0.89674	-0.5356	-0.58135	-1.10903	17.35	17.61	25.58	17.09	-0.64	0.04
Q8TC12	Retinol dehydrogenase 11	RDH11	4.00	-0.35634	-0.60292	-0.756	-0.72536	-0.10576	-0.1664	12.06	1.50	2.97	21.15	-0.45	0.12	-0.57655	-0.74256	-0.66522	-1.04641	-0.03792	-0.44967	8.13	18.58	20.05	22.52	-0.59	0.08
Q96FJ2	Dynein light chain 2, cytoplasmic	DYNLL2	3.00	-0.73677	-1.00533	-0.56391	-0.51636	-0.11557	-0.19216	13.12	2.33	3.75	24.76	-0.52	0.13	-0.31134	-0.8327	-0.86736	-0.48139	-0.55377	-0.48609	25.28	18.81	3.32	5.13	-0.59	0.01
Q96G03	Phosphoglucomutase-2	PGM2	6.67	-0.26504	-0.71655	-0.72019	-0.98179	-0.77218	-1.16894	21.95	12.79	19.32	18.16	-0.77	0.03	-0.41855	-0.74699	-0.59436	-0.89843	-0.96951	-1.2344	16.03	14.85	12.95	17.85	-0.81	0.03
Q96NY7	Chloride intracellular channel protein 6	CLIC6	5.67	1.537945	1.458189	0.726324	0.776623	1.792673	0.45583	3.91	2.47	61.20	25.06	1.12	0.03	0.96944	0.98003	1.755946	1.744541	1.898277	0.686664	0.52	0.56	56.12	26.24	1.34	0.03
Q9BRX8	Redox-regulatory protein FAM213A	FAM213A	5.00	-0.03064	-0.55835	-0.77447	-0.55767	-0.0704	0.015184	25.58	10.61	4.19	21.52	-0.33	0.22	0.010045	-0.48463	-0.82819	-0.74337	0.166958	-0.03741	24.01	4.16	10.00	28.40	-0.32	0.33
Q9BS26	Endoplasmic reticulum resident protein 44	ERP44	3.50	2.191439	0.534874	0.201991	0.252455	0.178898	0.447253	73.31	2.47	13.12	57.46	0.63	0.22	1.995391	0.624592	0.124539	0.339535	0.154379	0.26032	62.55	10.52	5.19	54.30	0.58	0.25
Q9P035	Very-long-chain (3R)-3-hydroxyacyl-CoA dehydratase 3	HACD3	4.17	-0.05068	0.080299	-0.5063	-0.79456	0.044664	-0.71727	6.42	14.08	36.50	22.53	-0.32	0.23	-0.74113	-0.10297	-0.63112	-1.17498	-0.16941	-0.40363	30.78	26.35	11.45	20.67	-0.54	0.10
Q9UHL4	Dipeptidyl peptidase 2	DPP7	5.50	1.594837	0.968624	0.818482	0.768134	0.946168	0.392964	30.22	2.47	26.79	24.25	0.91	0.04	1.592466	0.93579	0.197855	0.662341	0.619761	0.578991	31.64	22.57	2.00	33.46	0.76	0.10
Q9Y277	Voltage-dependent anion-selective channel protein 3	VDAC3	7.67	-0.51945	-0.81664	-1.11844	-0.86099	-0.20878	-0.42639	14.51	12.58	10.65	23.18	-0.66	0.08	-0.68299	-0.19104	-1.10522	-0.88625	-0.56903	-0.13301	23.88	10.71	21.21	22.94	-0.59	0.10
Q9Y490	Talin-1	TLN1	12.67	0.72129	1.513624	0.949289	1.809034	0.997622	1.045696	37.89	40.93	2.36	15.03	1.17	0.01	0.899862	0.95615	0.565002	1.529835	1.094343	1.168056	2.76	45.60	3.61	7.85	1.04	0.00
A0A024QZN7	Chromosome 10 open reading frame 70, isoform CRA_b	C10orf70	2.50	-0.77904	-1.36284	-0.88467	-1.30034	-0.94091	-0.23909	28.23	20.23	33.74	21.55	-0.92	0.03	-0.40688	-1.25877	-1.05283	-1.313	-0.83802	-0.413	40.58	12.72	20.68	19.36	-0.88	0.03
A0A024QZT0	Voltage-dependent anion channel 2, isoform CRA_c	VDAC2	16.17	-0.97212	-1.2679	-2.18618	-2.04403	-0.75414	-0.76907	14.45	6.96	0.73	42.51	-1.33	0.08	-0.96836	-1.07068	-2.15733	-2.04641	-1.02945	-1.00182	5.01	5.43	1.35	37.01	-1.38	0.06
A0A024R1A3	Ubiquitin-activating enzyme E1 (A1S9T and BN75 temperature sensitivity complementing), isoform CRA_a	UBE1	14.17	0.308122	0.375881	0.615126	0.813991	0.755315	0.507646	3.32	9.73	12.11	13.22	0.56	0.04	0.462615	0.23215	-0.23502	0.181181	0.177784	-0.07481	11.27	20.26	12.35	13.80	0.12	0.39
A0A024R1N1	Myosin, heavy polypeptide 9, non-muscle, isoform CRA_a	MYH9	23.00	0.80772	1.391883	1.00278	0.681532	0.289513	0.159967	28.25	15.68	6.35	29.87	0.72	0.11	0.716398	1.418696	1.524849	1.114448	1.249635	0.980167	33.76	19.98	13.17	8.97	1.17	0.00
A0A024R1T5	2',3'-cyclic-nucleotide 3'-phosphodiesterase	CNP	4.33	0.010982	-0.01297	-0.42755	-0.56506	-1.02375	-0.20448	1.17	6.73	39.11	22.16	-0.37	0.19	0.019371	0.110468	-0.22433	-0.09842	-1.43579	-0.53055	4.46	6.17	42.97	32.15	-0.36	0.38
A0A024R1Z6	Vesicle amine transport protein 1 homolog (T californica), isoform CRA_a	VAT1	8.50	0.171652	0.981853	-0.17357	-0.11477	0.432786	0.459432	38.70	2.88	1.31	26.09	0.29	0.32	-0.14075	0.823601	-0.57764	-0.46458	0.101781	-0.00568	45.58	5.54	5.26	31.33	-0.04	0.88
A0A024R2Z1	Guanine nucleotide binding protein (G protein), alpha transducing activity polypeptide 1, isoform CRA_a	GNAT1	25.83	-1.88085	-2.19917	-3.03732	-3.23807	-2.34241	-2.8922	15.54	9.82	26.63	37.65	-2.60	0.01	-1.92403	-2.42514	-2.50833	-2.54883	-2.55407	-3.19661	24.32	1.98	30.98	23.93	-2.53	0.01
A0A024R374	Cathepsin B, isoform CRA_a	CTSB	6.83	0.799262	0.809523	-0.02109	-0.11249	0.602953	0.848202	0.50	4.48	11.99	29.85	0.49	0.22	0.322979	0.275759	-0.05047	-0.16561	0.351199	1.010665	2.31	5.64	31.77	28.37	0.29	0.33
A0A024R3W7	Eukaryotic translation elongation factor 1 beta 2, isoform CRA_a	EEF1B2	2.67	-0.01524	0.289679	-0.69037	0.19627	1.192602	0.244592	14.89	42.14	44.86	35.61	0.20	0.54	-0.22327	0.455881	-0.59016	0.433343	0.397144	-0.05399	32.69	48.16	21.93	6.47	0.07	0.45
A0A024R3X4	Heat shock 60kDa protein 1 (Chaperonin), isoform CRA_a	HSPD1	41.17	-0.24718	-0.40671	-0.80101	-0.93618	0.498082	1.452736	7.81	6.62	45.15	71.79	-0.07	0.91	-0.05676	-0.34151	-0.94367	-0.84987	0.252589	0.858863	13.91	4.60	29.29	50.40	-0.18	0.71
A0A024R4F1	Enolase 1, (Alpha), isoform CRA_a	ENO1	61.00	-1.37281	-1.39643	-0.75869	-0.72536	-1.37033	-1.23426	1.16	1.63	6.66	25.53	-1.14	0.03	-1.14704	-0.97358	-1.0099	-0.9937	-1.72331	-1.65954	8.49	0.79	3.13	24.30	-1.25	0.03
A0A024R5K8	Serpin peptidase inhibitor, clade H (Heat shock protein 47), member 1, (Collagen binding protein 1), isofor	SERPINH1	9.17	1.494593	1.630982	0.83388	0.915036	0.853431	1.353521	6.68	3.98	24.27	24.75	1.18	0.03	1.638447	1.764575	1.156564	1.378589	1.164101	1.465036	6.18	10.86	14.70	16.98	1.43	0.01
A0A024R663	Kinectin 1 (Kinesin receptor), isoform CRA_a	KTN1	5.33	0.423428	0.247768	0.543101	0.273545	-1.44753	1.065685	8.60	13.17	99.26	4.11	0.18	0.43	0.244325	0.214596	0.464415	0.472426	-0.45268	0.42086	1.46	0.39	41.55	14.68	0.23	0.25
A0A024R6W0	Aspartate aminotransferase	GOT2	26.67	-0.48957	-0.33648	-1.58364	-1.31995	-0.42258	-0.62175	7.50	12.89	9.75	34.41	-0.80	0.14	-0.48538	-0.32872	-1.79225	-1.46423	-0.6375	-0.76438	7.67	16.01	6.21	38.76	-0.91	0.13
A0A024R7T3	Heterogeneous nuclear ribonucleoprotein F, isoform CRA_a	HNRPF	3.67	0.487169	-0.074	0.379551	0.093773	-0.25825	0.13573	27.16	13.96	19.19	11.07	0.13	0.31	0.042321	-0.63701	0.052675	0.075942	-0.33194	0.993885	32.69	1.14	60.76	25.75	0.03	0.87
A0A024R814	Ribosomal protein L7, isoform CRA_a	RPL7	4.50	0.465664	0.756462	0.79576	0.692014	0.81398	0.902664	14.20	5.08	4.35	8.32	0.74	0.01	0.358791	0.852934	0.69777	1.065852	0.752228	0.827959	23.99	17.94	3.71	9.21	0.76	0.01
A0A024R883	ATPase, H+ transporting, lysosomal 13kDa, V1 subunit G1, isoform CRA_a	ATP6V1G1	3.17	-1.0766	-0.58943	-0.35423	-0.52392	-0.14278	-0.17259	23.65	8.31	1.46	22.26	-0.48	0.14	-0.97912	0.281753	-0.50008	0.00378	0.023179	-0.15049	58.14	24.45	8.50	6.51	-0.22	0.12
A0A024R8S5	Protein disulfide-isomerase	P4HB	22.17	0.442095	0.014731	0.418149	-0.02164	0.975754	0.892042	20.79	21.13	4.10	30.07	0.45	0.20	0.503377	0.350769	0.310087	0.311748	0.901491	0.778392	7.47	0.08	6.03	20.04	0.53	0.08
A0A024R9B7	Cytochrome c oxidase subunit VIc, isoform CRA_a	COX6C	2.00	-0.53848	-1.00114	-1.43505	-1.27971	-0.59197	-0.64168	22.48	7.61	2.44	25.15	-0.91	0.06	-0.91285	-1.10061	-1.0455	-1.66646	-0.69961	-1.17325	9.19	29.97	23.01	14.25	-1.10	0.01
A0A024R9Z0	NADH dehydrogenase (Ubiquinone) 1 alpha subcomplex, 4, 9kDa, isoform CRA_b	NDUFA4	2.33	-0.52312	-0.53011	-0.62514	-0.83725	-0.40046	-0.19329	0.34	10.38	10.14	15.10	-0.52	0.05	-0.9761	-0.91175	-0.76576	-0.7998	-0.30231	-0.29064	3.15	1.67	0.57	24.32	-0.67	0.07
A0A024RA75	3-hydroxyisobutyrate dehydrogenase	HIBADH	9.67	1.898692	1.364941	-0.14956	0.114863	0.488151	0.359647	25.87	12.92	6.29	63.37	0.68	0.30	1.610518	1.316762	-0.14496	0.11048	0.570763	0.313145	14.35	12.49	12.59	54.94	0.63	0.29
A0A024RAD8	Aldehyde dehydrogenase 4 family, member A1, isoform CRA_a	ALDH4A1	3.17	-0.5068	-0.29909	-0.64656	-0.28709	-0.575	-0.50523	10.16	17.53	3.42	4.88	-0.47	0.01	-0.70926	-0.5454	-0.3317	-0.43938	-0.2924	-0.2776	8.02	5.28	0.73	11.82	-0.43	0.05
A0A024RAM4	Microtubule-associated protein 1B, isoform CRA_b	MAP1B	14.67	-0.24042	-0.32074	-0.79134	-0.53725	-0.14541	-0.4207	3.94	12.42	13.45	14.50	-0.41	0.08	-0.44068	-0.65599	-0.79532	-0.8095	-0.18978	-0.15478	10.53	0.69	1.72	22.24	-0.51	0.11
A0A024RB87	RAP1B, member of RAS oncogene family, isoform CRA_a	RAP1B	3.50	-0.11148	-0.34779	-0.50142	-0.16325	-1.01751	-1.44243	11.56	16.50	20.68	33.18	-0.60	0.20	-0.74701	-0.33677	-0.8363	0.094024	-0.55833	-0.60154	19.97	44.08	2.12	10.66	-0.50	0.02
A0A024RC87	Ribonuclease/angiogenin inhibitor 1, isoform CRA_a	RNH1	13.67	1.2067	1.044394	0.885583	0.822215	0.933049	0.878114	7.95	3.11	2.69	10.31	0.96	0.01	1.154258	1.113119	0.704619	0.824164	1.044188	1.042716	2.02	5.86	0.07	12.88	0.98	0.01
A0A024RCA7	Ribosomal protein, large, P2, isoform CRA_a	RPLP2	4.83	0.636966	0.39665	0.339055	0.533851	0.866831	0.756646	11.75	9.53	5.40	13.98	0.59	0.04	0.436137	0.536486	0.858391	0.516843	0.46403	0.798104	4.92	16.66	16.30	7.43	0.60	0.01
A0A024RDG6	Scavenger receptor class B, member 2, isoform CRA_a	SCARB2	9.17	0.411342	0.23943	0.003706	-0.14593	0.372624	-0.11667	8.42	7.33	23.75	13.66	0.13	0.38	0.051865	0.009444	-0.40621	-0.28967	0.547466	0.126494	2.08	5.71	20.49	23.85	0.01	0.98
A0A024RE27	Fumarylacetoacetate hydrolase domain containing 2A, isoform CRA_a	FAHD2A	3.00	0.147153	0.36796	-0.04141	0.217058	0.754818	0.544063	10.80	12.63	10.31	20.50	0.33	0.18	0.421978	0.46995	0.16121	0.392017	0.471804	0.132756	2.35	11.29	16.54	6.16	0.34	0.02
A0A087WTP3	Far upstream element-binding protein 2	KHSRP	4.67	-0.09016	-0.75768	0.337797	0.779083	0.106866	-0.18363	32.15	21.46	14.19	34.54	0.03	0.92	-0.53595	-0.35385	-0.07319	0.338338	0.002892	0.056164	8.91	20.03	2.61	20.35	-0.09	0.65
A0A087WVQ6	Clathrin heavy chain	CLTC	44.33	0.064858	-0.04277	0.13719	0.176111	-0.38431	-0.17786	5.27	1.91	10.10	14.91	-0.04	0.80	0.015474	0.037198	0.098067	-0.01854	-0.2174	-0.06268	1.06	5.71	7.58	6.75	-0.02	0.71
A0A087WZH7	Myristoylated alanine-rich C-kinase substrate	MARCKS	2.50	-0.91719	-1.39393	-0.81213	-1.19277	-2.37903	-0.90167	23.16	18.55	66.68	16.10	-1.27	0.02	-1.10402	-1.55703	-0.9138	-0.54036	-1.61737	-0.4909	22.02	18.20	52.57	20.31	-1.04	0.03
A0A090N7T9	Secernin 1	SCRN1	7.83	-0.92689	-0.88265	-0.73997	-0.76066	-0.92862	-0.81508	2.17	1.01	5.56	5.69	-0.84	0.00	-0.82871	-0.89117	-0.5533	-0.42542	-0.84247	-1.21538	3.06	6.26	18.18	19.37	-0.79	0.04
A0A090N8Y2	Protein disulfide-isomerase A4	ERP70	14.83	0.934872	0.9241	1.002384	1.093402	0.797828	0.754972	0.53	4.46	2.10	9.39	0.92	0.01	0.884514	1.081605	0.757668	0.975953	0.203512	0.416696	9.65	10.68	10.43	23.13	0.72	0.07
A0A0A0MQT0	Retinol binding protein 1, cellular	RBP1	10.33	1.647183	1.582683	1.040381	1.242685	0.442077	0.816292	3.16	9.90	18.24	33.10	1.13	0.06	1.185327	1.470587	0.288908	0.490142	0.748171	1.088539	13.94	9.85	16.61	31.62	0.88	0.08
A0A0A0MT83	Isovaleryl-CoA dehydrogenase, mitochondrial	IVD	2.17	-0.07764	-0.21803	-0.09136	-0.58193	0.283439	0.556993	6.88	23.82	13.37	28.28	-0.02	0.93	0.376791	0.262226	0.387767	-0.33925	-0.32027	0.529716	5.61	34.90	40.50	8.85	0.15	0.23
A2A274	Aconitate hydratase, mitochondrial	ACO2	21.50	-0.55515	-0.55723	-0.83229	-1.0386	-0.40971	-0.4109	0.10	10.09	0.06	17.88	-0.63	0.06	-0.49246	-0.40311	-1.00634	-1.17441	-0.33246	-0.46023	4.38	8.23	6.26	24.47	-0.64	0.10
A3RJH1	ATP-dependent RNA helicase DDX1	DDX1	4.50	0.070867	0.45168	0.491435	0.330842	0.078816	-0.00155	18.56	7.86	3.94	12.87	0.24	0.16	0.384701	0.295464	-0.48902	0.309306	-0.10706	0.050386	4.37	38.16	7.71	15.45	0.07	0.64
A8K288	cDNA FLJ76322	NA	6.83	0.451914	0.508962	0.510167	0.490788	0.876076	0.187159	2.80	0.95	33.14	3.37	0.50	0.00	0.180791	0.310823	0.616913	0.469513	0.951319	0.150418	6.37	7.22	38.28	12.81	0.45	0.05
A8K401	Prohibitin, isoform CRA_a	PHB	10.67	-0.53078	-0.3293	-0.50819	-0.50509	-0.53881	-0.32094	9.86	0.15	10.66	3.19	-0.46	0.00	0.058824	-0.01239	-0.59742	-0.16306	-0.32648	-0.07774	3.49	21.13	12.16	13.57	-0.19	0.25
A8K525	cDNA FLJ76817, highly similar to Homo sapiens non-POU domain containing, octamer-binding (NONO), mR	NA	2.50	-0.35382	0.824929	-0.30493	-0.01177	0.305324	0.061967	54.76	14.32	11.90	17.22	0.09	0.55	-0.36932	0.394174	0.818339	-0.4059	0.065487	0.162137	36.57	56.64	4.74	10.15	0.11	0.19
A8K651	cDNA FLJ75700, highly similar to Homo sapiens complement component 1, q subcomponent binding prote	NA	1.67	0.3641	-0.18831	-0.1127	-0.15383	0.148575	0.522874	26.75	2.02	18.24	16.57	0.10	0.55	1.205706	0.515983	0.08572	0.308695	0.365072	0.316587	33.18	10.91	2.38	26.99	0.47	0.15
A8K8U1	cDNA FLJ77762, highly similar to Homo sapiens cullin-associated and neddylation-dissociated 1 (CAND1), m	NA	9.17	-0.01245	0.328595	0.29177	-0.14331	-0.07967	-0.08724	16.64	21.16	0.37	8.78	0.05	0.56	-0.20876	0.377308	-0.0552	-0.19564	-0.25466	0.08026	28.34	6.88	16.34	8.91	-0.04	0.58
A8KAH7	cDNA FLJ75444, highly similar to Homo sapiens protein kinase, cAMP-dependent, regulatory, type II, alpha	NA	9.33	0.349652	0.424356	-0.1528	0.645247	-0.81408	-0.01221	3.66	38.15	38.32	25.78	0.07	0.79	0.153076	0.424044	-0.02167	0.358949	-0.41056	-0.3136	13.24	18.55	4.75	22.61	0.03	0.89
A8YXX4	Glutamine synthetase	PIG59	10.83	1.116885	-0.20791	-1.24968	-1.52848	-1.29362	-1.9128	60.72	13.62	29.89	89.62	-0.85	0.32	1.009174	-0.13552	-0.79225	-1.19969	-0.90343	-1.73095	53.34	19.84	39.48	72.76	-0.63	0.37
B0YIW2	Apolipoprotein C-III	APOC3	1.17	1.848467	1.641181	1.415766	1.543017	1.125438	2.132865	10.14	6.23	47.46	9.82	1.62	0.00	1.759619	1.640158	1.341283	1.538937	1.672284	2.405546	5.85	0.12	35.19	25.71	1.69	0.01
B0YJ88	Radixin	RDX	8.83	1.416955	1.04421	0.473412	0.924095	0.472116	-0.26858	18.17	21.91	35.53	37.00	0.68	0.17	1.898863	1.054807	0.92428	1.086201	-0.06838	0.209196	40.23	7.93	13.56	46.46	0.85	0.18
B1AKK2	Dimethylarginine dimethylaminohydrolase 1, isoform CRA_b	DDAH1	11.17	-0.61721	-1.26061	-0.33604	-0.21984	-0.56091	-1.35091	31.02	5.69	37.78	26.56	-0.72	0.08	-0.4863	-1.05732	-0.39086	0.288175	-0.46176	-1.41443	27.63	32.68	45.07	33.99	-0.59	0.16
B1AKZ4	Phosphoprotein enriched in astrocytes 15, isoform CRA_a	PEA15	5.17	0.49599	-1.85905	-1.6478	-1.72275	-2.40767	-1.61308	95.18	3.67	37.99	68.77	-1.46	0.07	0.564513	-0.81398	-1.20437	-0.97672	-1.70163	-1.43281	62.85	11.13	13.14	59.44	-0.93	0.16
B2R5T2	cDNA, FLJ92608, highly similar to Homo sapiens aldehyde dehydrogenase 1 family, member A3 (ALDH1A3)	NA	5.33	1.379541	3.081129	1.308882	1.328324	1.867252	1.934884	74.91	0.95	3.31	38.96	1.82	0.02	1.301627	2.395054	1.348755	1.562839	1.58923	2.115222	51.17	10.47	25.50	17.34	1.72	0.01
B2R7T8	cDNA, FLJ93598, highly similar to Homo sapiens capping protein (actin filament) muscle Z-line, beta (CAPZE	NA	5.50	0.025366	-0.17539	0.253372	0.235761	-0.34596	-0.46957	9.82	0.86	6.05	22.35	-0.08	0.71	0.296819	-0.16561	0.757818	0.397008	0.129455	-0.14575	22.47	17.59	13.45	23.21	0.21	0.37
B2RB23	cDNA, FLJ95265, highly similar to Homo sapiens acetyl-Coenzyme A acyltransferase 2 (mitochondrial 3-oxo	NA	8.50	1.032601	0.49084	0.074133	0.151638	0.43558	0.383678	26.25	3.80	2.54	23.56	0.43	0.15	0.813103	0.434517	0.008578	-0.24982	0.276857	0.438503	18.45	12.63	7.91	25.24	0.29	0.32
B2RDE8	cDNA, FLJ96580, highly similar to Homo sapiens hepatoma-derived growth factor (high-mobility group pro	NA	3.67	0.210618	0.203448	0.718614	0.33649	0.13489	-0.02437	0.35	18.62	7.80	17.56	0.26	0.20	0.034212	0.074926	0.963221	0.739033	0.051189	-0.00032	2.00	10.97	2.52	35.00	0.31	0.37
B3KM97	cDNA FLJ10554 fis, clone NT2RP2002385, highly similar to Synaptic glycoprotein SC2	NA	3.33	-1.12601	-0.44522	-1.5352	-0.94529	-0.91247	-0.61329	32.76	28.52	14.61	17.34	-0.93	0.03	-0.95598	-0.46595	-1.08629	-0.89375	-1.0265	-0.43686	23.79	9.22	28.50	11.31	-0.81	0.01
B3KRY3	cDNA FLJ35079 fis, clone PLACE6005283, highly similar to Lysosome-associated membrane glycoprotein 1	NA	3.17	1.402657	0.437172	0.005819	-0.80834	0.888211	0.850242	45.63	38.88	1.86	42.62	0.46	0.40	1.192919	0.201816	-0.3333	-1.08658	0.344212	1.060584	46.75	36.10	34.41	45.84	0.23	0.67
B3KS79	cDNA FLJ35730 fis, clone TESTI2003131, highly similar to ALPHA-1-ANTICHYMOTRYPSIN	NA	2.50	0.581349	1.1127	-0.11327	-0.30619	0.210411	-0.60704	25.75	9.44	39.03	45.35	0.15	0.72	0.234115	0.48278	0.211688	-0.27098	-0.1295	-0.56846	12.16	26.56	21.35	24.46	-0.02	0.94
B3KUY2	Prostaglandin E synthase 3 (Cytosolic), isoform CRA_c	PTGES3	1.50	0.048772	-0.15306	0.326638	-0.61748	0.17457	0.30921	9.88	44.69	6.59	12.51	0.01	0.91	0.466902	-0.06007	0.792274	-0.27098	-0.03962	-0.15812	25.55	49.88	5.80	15.66	0.12	0.39
B4DQI7	cDNA FLJ58042, highly similar to Protein NipSnap1	NA	2.67	-0.16256	-0.71717	-1.78813	-0.76738	0.876229	-0.08319	26.85	48.04	45.36	56.40	-0.44	0.46	0.23001	-0.41071	-1.694	-0.30924	0.686165	0.413967	30.90	63.10	13.30	45.86	-0.18	0.73
B4DRS6	Sideroflexin	NA	5.67	-0.35382	-0.49251	-0.84176	-0.97297	-0.25058	-0.06874	6.79	6.43	8.90	25.15	-0.50	0.15	-0.35075	-0.65009	-0.45394	-0.59649	-0.47549	0.038553	14.62	6.98	24.93	12.95	-0.41	0.05
B4DZZ0	cDNA FLJ52128, highly similar to PRA1 family protein 3	NA	6.67	0.295026	-0.34368	0.139598	0.201323	-0.35684	0.187904	30.80	3.02	26.39	8.28	0.02	0.81	-0.11679	-0.45533	-0.45637	-0.43802	-0.37607	0.693696	16.52	0.90	50.16	26.55	-0.19	0.40

UniProt	Protein name	Gene name	Peptide mean	08/017 hESC-RPE to hRPE: Analysis 1.1	Analysis 1.2	Analysis 2.1	Analysis 2.2	Analysis 3.1	Analysis 3.2	Technical replicate CV 1	Technical replicate CV 2	Technical replicate CV 3	Biological CV	Fold change (mean)	Unadjusted p-value (t-test)	08/023 hESC-RPE to hRPE: Analysis 1.1	Analysis 1.2	Analysis 2.1	Analysis 2.2	Analysis 3.1	Analysis 3.2	Technical replicate CV 1	Technical replicate CV 2	Technical replicate CV 3	Biological CV	Fold change (mean)	Unadjusted p-value (t-test)
B4E1J8	cDNA FLJ56285, highly similar to ADP-ribosylation factor-like protein 8B	NA	2.50	0.206359	0.580526	1.072874	0.478542	0.071784	0.122159	18.24	28.72	2.47	24.83	0.42	0.16	0.081159	0.52673	0.944145	0.239629	0.083163	0.144232	21.67	33.86	2.99	18.41	0.34	0.14
B4E2G8	cDNA FLJ54047, highly similar to Alpha-1 catenin (Cadherin-associated protein)	NA	6.17	0.199175	1.064955	0.274562	0.903666	0.478451	0.196809	41.21	30.35	13.76	12.44	0.52	0.03	-1.07142	0.494089	0.656625	0.445809	-0.67894	0.041468	69.99	10.31	34.59	31.73	-0.02	0.95
B4E3D4	cDNA FLJ56293, highly similar to Transmembrane glycoprotein NMB	NA	7.83	1.279459	1.157044	0.399984	0.454695	0.713899	0.528274	6.00	2.68	9.09	29.95	0.76	0.09	0.80397	0.852934	-0.4163	-0.13564	1.061233	0.861222	2.40	13.71	9.79	39.72	0.50	0.33
B5BUB5	Autoantigen La (Fragment)	SSB	5.67	0.023818	0.00946	0.66429	0.691198	-0.1544	-0.62088	0.70	1.32	22.67	37.65	0.10	0.77	-0.11965	-0.34318	0.475378	0.631723	0.034327	-1.04314	10.93	7.66	50.48	37.74	-0.06	0.87
B5MCX3	Septin-2	SEPT2	3.83	-0.07039	-0.21605	0.144642	-0.31044	-0.36312	-0.00723	7.13	22.12	17.36	3.91	-0.14	0.04	-0.08646	-0.21131	-0.1025	-0.35563	-0.38118	0.049145	6.12	12.37	20.94	2.97	-0.18	0.02
B5MDF5	GTP-binding nuclear protein Ran	RAN	4.67	0.402434	0.42379	0.518147	0.810688	-0.33552	-0.11192	1.05	14.29	10.94	29.50	0.28	0.39	0.160937	0.330759	0.4554	0.891278	0.05418	-0.13513	8.31	21.20	9.27	25.83	0.29	0.29
B7Z2N4	cDNA FLJ56074, highly similar to 150 kDa oxygen-regulated protein (Orp150)	NA	8.33	0.306691	0.047428	-0.18739	0.265892	0.570845	0.476395	12.67	22.04	4.63	17.26	0.25	0.23	0.383862	0.077016	-0.01067	-0.48492	0.67908	0.303983	14.98	23.04	18.28	24.62	0.16	0.54
B7Z6B3	Receptor expression-enhancing protein	NA	2.83	0.312884	0.182525	0.757859	0.791016	0.615416	1.040827	6.38	1.63	20.70	20.99	0.62	0.08	0.73771	0.264613	1.45706	1.520805	0.37196	0.830129	22.98	3.12	22.27	39.73	0.86	0.11
B9EKV4	Aldehyde dehydrogenase 9 family, member A1	ALDH9A1	12.50	0.625528	0.2808	0.160381	0.181003	0.412555	0.342918	16.82	1.01	3.41	10.21	0.33	0.06	0.910042	0.491113	0.120588	0.413586	0.350388	0.278262	20.39	14.31	3.53	17.62	0.43	0.09
C9J9K3	40S ribosomal protein SA (Fragment)	RPSA	4.17	0.896578	0.719818	0.55974	0.977019	2.050986	0.433714	8.65	20.31	71.89	28.11	0.94	0.03	0.610733	0.6856	0.471915	0.852253	1.813869	0.455309	3.67	18.53	62.07	26.62	0.81	0.04
D3DPU2	Adenylyl cyclase-associated protein	CAP1	5.83	0.132861	0.014731	-0.20043	-0.11791	-0.10245	-0.63639	5.79	4.04	25.88	14.66	-0.15	0.36	0.203995	0.010101	-0.10522	0.027	0.256922	0.034587	9.49	6.48	10.88	6.74	0.07	0.33
D3DQU2	Tripeptidyl peptidase I, isoform CRA_a	TPP1	7.00	-0.43506	-0.11968	-0.64324	-0.57053	-0.13279	-0.44705	15.40	3.56	15.34	12.73	-0.39	0.07	-0.25299	-0.29914	-1.00736	-0.95405	0.352333	0.089747	2.26	2.61	12.83	39.77	-0.35	0.43
D9IAI1	Epididymis secretory protein Li 34	PEBP1	14.67	0.47178	0.565982	0.542191	0.563766	0.340526	0.60322	4.62	1.06	12.84	2.61	0.51	0.00	0.594282	0.653178	0.839141	0.776935	0.260551	0.614664	2.89	3.05	17.27	12.47	0.62	0.03
E5KRK5	Mitochondrial NADH-ubiquinone oxidoreductase 75 kDa subunit	NDUFS1	18.00	-0.32001	-0.26905	-1.13581	-1.11648	-0.76884	-0.04872	2.50	0.95	34.58	28.69	-0.61	0.14	-0.51733	-0.37141	-1.05704	-1.29431	-0.87851	-0.00977	7.15	11.60	41.34	27.50	-0.69	0.11
E5RHW4	Erlin-2 (Fragment)	ERLIN2	5.33	1.765963	1.400538	1.111414	1.677252	0.98553	1.252385	17.82	27.38	13.04	16.07	1.37	0.01	1.503531	1.291599	0.742169	1.179511	1.770041	2.079551	10.37	21.27	15.11	33.32	1.43	0.04
E9KL44	Epididymis tissue sperm binding protein Li 14m	NA	27.67	0.479001	0.616135	0.230736	0.164397	-0.00399	0.051095	6.72	3.25	2.70	19.00	0.26	0.24	0.45672	0.570864	-0.2205	-0.21104	0.474634	0.333847	5.59	0.46	6.89	25.09	0.23	0.41
E9KL48	Epididymis tissue sperm binding protein Li 18mP	GLUD1	14.83	-0.06338	-0.52219	-0.52258	-0.95446	-0.63279	-0.44301	22.30	21.01	9.29	15.73	-0.52	0.06	-0.21842	-0.50732	-0.68055	-1.0485	-0.54258	-0.481	14.11	17.94	3.02	17.02	-0.58	0.06
E9PCR7	2-oxoglutarate dehydrogenase, mitochondrial	OGDH	10.67	-0.16344	0.146621	-0.7822	-0.53343	-0.57917	-0.5232	15.14	12.16	2.74	25.61	-0.41	0.18	-0.01232	0.221794	-0.7142	-0.75738	0.016448	-0.60675	11.45	2.12	30.08	28.55	-0.31	0.33
E9PIE4	Mitochondrial carrier homolog 2 (Fragment)	MTCH2	2.17	0.14056	0.025402	-0.95898	-1.05111	0.248211	0.131608	5.64	4.51	5.71	38.90	-0.24	0.59	0.178082	-0.04067	-0.25511	-0.96633	0.029473	-0.29615	10.70	34.17	15.89	21.68	-0.23	0.38
E9PLK3	Puromycin-sensitive aminopeptidase	NPEPPS	6.83	-0.09415	-0.10225	0.022617	0.011165	0.090679	-0.42791	0.40	0.56	25.15	5.85	-0.08	0.26	-0.4165	0.036553	-0.20437	-0.06477	-0.1846	-0.46821	22.02	6.84	13.86	6.70	-0.22	0.06
F1T0F7	Cellular retinoic acid binding protein 1	CRABP1	14.67	-1.94762	-1.89531	-2.41442	-2.5021	-2.50271	-2.71843	2.56	4.30	10.55	26.17	-2.33	0.01	-1.96023	-1.8382	-2.43671	-2.37121	-2.55286	-2.7154	5.98	3.21	7.96	26.93	-2.31	0.01
F5H7R9	Parathymosin (Fragment)	PTMS	1.00	-0.8046	-0.51403	-1.04824	-0.92713	-1.35309	-0.51712	14.19	5.93	39.86	12.00	-0.86	0.01	-0.86019	-0.59181	-0.65602	-0.63086	-1.75646	-0.70106	13.12	1.23	49.54	17.35	-0.87	0.04
F8W031	Uncharacterized protein (Fragment)	NA	4.67	0.728555	0.576968	0.745739	0.490037	0.45804	0.506453	7.42	12.50	2.37	6.26	0.58	0.01	0.861905	1.08732	1.574465	0.996463	0.76104	1.275965	11.03	27.96	24.97	12.71	1.09	0.01
G3V5Z7	Proteasome subunit alpha type	PSMA6	3.00	-0.0385	0.484477	0.626159	0.162985	-0.14711	-0.20611	25.36	22.51	2.89	20.08	0.15	0.48	0.157798	0.629016	0.451149	0.344115	0.034529	-0.03829	22.89	5.24	3.57	15.47	0.26	0.19
G4V2I9	Anion exchange protein	NA	6.50	-1.22423	-1.19873	-1.13348	-1.99276	-1.21623	-1.21445	1.25	40.91	0.09	11.07	-1.33	0.01	-1.12537	-1.14572	-1.47208	-1.84623	-1.10795	-1.06447	1.00	18.24	2.13	20.09	-1.29	0.02
G4XXL9	Cytochrome c	CYCS	9.50	-1.25961	-1.82839	-2.06256	-2.21219	-0.9856	-1.27986	27.52	7.33	14.37	33.45	-1.60	0.03	-1.3696	-1.69007	-2.04341	-2.43938	-1.25614	-1.35244	15.64	19.29	4.72	30.54	-1.69	0.03
G4Y815	Beta-synuclein	SNCB	3.50	-1.83307	-1.16727	0.367891	-1.02889	-0.83812	-0.48828	32.07	63.57	17.06	41.87	-0.83	0.14	-0.78011	-1.05502	0.314774	-0.84442	-1.28659	-0.79006	13.43	53.94	24.10	34.96	-0.74	0.09
H0Y7A7	Calmodulin (Fragment)	CALM2	5.83	-0.41209	-0.52164	-0.25095	-0.02674	-0.92013	-1.52462	5.37	10.97	29.20	34.47	-0.61	0.20	-0.44068	-0.12924	-0.36782	0.146947	-0.81129	-0.9426	15.21	24.97	6.43	26.43	-0.42	0.21
H0YBX3	Ribonuclease UK114 (Fragment)	HRSP12	2.33	0.195914	0.571485	-0.03108	-0.48429	0.404023	-0.89508	18.30	22.03	59.69	24.26	-0.04	0.87	0.271446	-0.00596	0.270564	-0.35466	0.280266	-1.52161	13.55	30.17	78.38	16.99	-0.18	0.52
H0YFI1	Ragulator complex protein LAMTOR1 (Fragment)	LAMTOR1	1.00	-0.02202	0.793951	-0.24559	-0.08147	0.966842	0.598561	38.96	8.04	17.95	31.66	0.34	0.35	0.037632	0.312776	0.096174	0.805515	0.036748	0.218242	13.44	34.08	8.88	14.09	0.25	0.13
H3BN98	Uncharacterized protein (Fragment)	NA	5.83	-0.8454	-0.99924	-0.41265	-0.90129	0.248682	0.365254	7.53	23.72	5.71	47.42	-0.42	0.37	-0.92361	-0.83388	-0.62877	-0.58131	-0.19522	0.152153	4.40	2.33	16.94	31.87	-0.50	0.19
H3BQZ7	HCG2044799	HNRNPUL2-BSCL2	2.83	1.272387	-0.13273	1.211865	1.734226	0.159755	0.033942	63.90	25.33	6.16	48.09	0.71	0.22	1.056824	-0.08978	0.179872	1.06814	0.100817	0.578704	53.42	42.21	23.21	11.54	0.48	0.03
H3BT58	Coactosin-like protein	COTL1	2.83	0.787791	0.85536	1.45187	1.325803	1.025838	0.42258	3.31	6.17	29.14	25.53	0.98	0.04	0.949382	1.142064	1.742547	1.793554	1.307088	0.843076	9.43	2.50	22.55	29.73	1.30	0.03
H6VRG3	Keratin 1	KRT1	10.00	1.715608	1.313017	0.771898	0.149976	0.42978	0.164514	19.61	30.02	12.96	49.22	0.76	0.19	1.502371	0.944861	0.32127	-0.1727	0.436944	0.774393	26.99	23.98	16.46	40.04	0.63	0.20
I3L2R6	Thioredoxin domain-containing protein 17	TXNDC17	1.17	-0.13096	1.285636	0.652534	0.310366	-0.08634	1.592341	64.34	16.69	74.10	16.99	0.60	0.02	1.038059	0.912096	0.027075	0.543405	0.273097	1.154655	6.17	25.04	41.91	22.59	0.66	0.08
I4AY87	Macrophage migration inhibitory factor (Fragment)	MIF	7.17	1.511884	1.464049	1.816903	1.642547	1.995871	2.328021	2.34	8.54	16.21	24.58	1.79	0.01	1.49306	1.608784	1.451427	1.373144	1.39628	1.740643	5.67	3.84	16.80	6.07	1.51	0.00
J3K000	PEPD protein	PEPD	3.83	0.729506	1.006912	0.521105	0.696901	1.363036	0.611107	13.55	8.61	36.04	14.53	0.82	0.02	0.686756	1.927066	0.946379	0.206552	1.136808	1.108337	57.30	35.49	1.40	27.26	1.00	0.04
J3KPF3	4F2 cell-surface antigen heavy chain	SLC3A2	9.33	0.160775	0.564827	0.271711	0.348549	0.898896	0.815811	19.68	3.77	4.07	21.71	0.51	0.10	0.422141	0.342972	0.308249	0.446365	0.165115	0.625113	3.88	6.76	22.36	1.31	0.38	0.00
J3KPX7	Prohibitin-2	PHB2	7.50	-0.28769	-0.55835	-0.46767	-1.00541	-0.01553	-0.05253	13.23	26.06	1.81	23.74	-0.40	0.19	-0.16979	-0.49298	-0.24938	-0.67732	-0.23482	-0.4831	15.77	20.82	12.14	4.47	-0.38	0.01
J3QRS3	Myosin regulatory light chain 12A	MYL12A	7.33	0.364712	0.582429	0.194135	0.322855	-0.05797	0.722278	10.65	6.30	37.34	7.53	0.35	0.03	0.701788	0.784989	0.803627	0.912616	0.740889	1.324303	4.08	5.34	28.21	11.30	0.88	0.01
J9JID7	Lamin B2, isoform CRA_a	LMNB2	13.67	-0.50932	-0.53506	-0.10327	0.019772	-1.05943	-0.35669	1.26	6.03	33.78	23.67	-0.42	0.17	-0.68123	-0.40166	-0.19449	-0.00126	-0.59128	-0.18488	13.66	9.46	19.79	15.69	-0.34	0.12
K7EKL3	Granulins (Fragment)	GRN	2.00	1.656329	1.705047	1.42211	1.091548	0.61423	0.407422	2.39	16.13	10.12	37.94	1.15	0.08	1.734948	1.469632	1.42867	1.68763	1.379927	1.314178	12.97	12.66	3.22	9.42	1.50	0.00
K7ELL7	Glucosidase 2 subunit beta	PRKCSH	7.17	0.135193	0.096013	0.355721	0.225425	0.388195	0.465996	1.92	6.38	3.81	10.75	0.28	0.09	0.232623	0.296182	0.464782	0.588407	0.797425	0.942341	3.11	6.06	7.10	21.27	0.55	0.09
K9JA46	Epididymis luminal secretory protein 52	EL52	35.00	-0.16675	-0.34441	-0.52678	-0.51034	-0.5886	0.056541	8.70	0.81	31.10	10.74	-0.35	0.06	-0.24206	-0.36288	-0.52863	-0.21455	-0.66478	-0.67075	5.92	15.33	0.29	13.09	-0.45	0.06
L7RXH0	Integrin, alpha V	ITGAV	7.50	0.720813	0.426053	0.602438	0.157323	0.838343	0.658794	14.40	21.64	8.79	12.23	0.57	0.03	0.716264	0.766325	0.62317	0.033178	0.528812	0.463717	2.45	28.52	3.19	13.66	0.52	0.05
M0R026	Acetolactate synthase-like protein	ILVBL	7.83	-0.13118	-0.30823	0.393339	-0.05057	-0.20137	-0.42506	8.67	21.59	10.94	19.07	-0.12	0.50	-0.15385	-0.37198	0.107963	-0.40256	-0.55286	-0.32107	10.67	24.76	11.34	10.60	-0.28	0.08
Q08ET0	Cell proliferation-inducing protein 47	hCG_39985	4.50	-0.42445	-0.02837	-0.53175	-0.59421	-0.64913	-0.85363	19.29	3.06	10.01	19.25	-0.51	0.08	-0.32034	-0.0459	-0.44702	-0.74211	-0.41856	-0.26674	13.41	14.41	7.43	14.08	-0.37	0.09
Q0VAS5	Histone H4	HIST1H4H	10.00	-2.30607	-2.11864	-0.6683	-0.49502	-0.23766	-0.49066	9.17	8.48	12.37	53.80	-1.05	0.21	-1.78464	-1.60717	-0.53009	-0.51418	-0.90575	-0.96166	8.69	0.78	2.74	38.04	-1.05	0.09
Q12771	P37 AUF1	NA	7.33	-0.65546	-0.76218	0.288952	0.380897	-0.63452	-0.65074	5.23	4.50	0.80	43.81	-0.34	0.42	-0.41972	-0.56418	0.524585	0.269455	-0.63044	-0.42704	7.07	12.47	9.95	39.27	-0.21	0.56
Q2TB59	Nicotinamide nucleotide transhydrogenase	NNT	8.50	0.052573	0.191031	-0.60727	-0.0533	0.274221	-0.00893	6.78	26.82	13.83	16.48	-0.03	0.88	0.209122	0.132145	-0.75419	0.10345	0.247547	-0.0694	3.77	40.84	15.47	15.37	-0.02	0.90
Q49AN9	SNRPG protein	SNRPG	1.50	0.148219	0.380263	0.743366	1.117052	0.3371	0.614341	11.35	18.21	13.55	24.60	0.56	0.11	0.099273	-0.11485	0.872005	1.367385	0.20405	0.33741	10.48	24.04	6.53	43.89	0.46	0.31
Q4W5L2	Alpha-synuclein (Fragment)	SNCA	4.33	0.971573	1.504085	0.416362	-0.10765	0.220644	0.245904	25.81	25.40	1.24	45.86	0.54	0.26	0.290204	1.282659	0.793588	0.932982	-0.02882	-0.72811	46.81	6.83	33.62	40.27	0.42	0.40
Q53ES0	Sodium/potassium-transporting ATPase subunit alpha (Fragment)	NA	40.67	-1.03608	-1.23001	-1.34544	-1.44931	-1.31537	-1.49923	9.49	5.09	9.00	11.15	-1.31	0.00	-1.01719	-1.06283	-1.25662	-1.41025	-1.25269	-1.4485	2.24	7.52	9.58	12.38	-1.24	0.01
Q53F20	Acidic (Leucine-rich) nuclear phosphoprotein 32 family, member E variant (Fragment)	NA	4.17	-1.1273	-0.84057	0.272589	0.112426	-0.24295	-0.67153	14.01	7.84	20.85	40.49	-0.42	0.35	-0.86816	-0.82878	0.067241	0.078574	-0.75926	-0.71997	1.93	0.56	1.93	37.38	-0.51	0.22
Q53FB6	Mitochondrial aldehyde dehydrogenase 2 variant (Fragment)	NA	10.67	0.19969	1.092724	-0.75913	-0.4954	0.004537	-0.49943	42.42	12.89	24.45	49.85	-0.08	0.86	-0.325	0.89963	-0.49329	-0.29679	-0.18484	-0.62578	56.66	9.62	21.45	34.47	-0.17	0.53
Q53G25	Ribosomal protein S5 variant (Fragment)	NA	3.33	0.343925	0.285169	0.834921	0.460454	-0.03316	-0.05414	2.88	18.25	1.03	24.15	0.31	0.26	0.339352	0.157853	1.344874	0.400071	0.581581	0.847485	8.88	44.72	13.00	23.48	0.61	0.08
Q53GF9	Full-length cDNA 5-PRIME end of clone CS0DF013YM24 of Fetal brain of Homo sapiens (Human) variant (Fr	NA	4.17	0.254347	0.158575	-0.13058	-0.08259	0.452432	0.5358	4.69	2.35	4.08	20.63	0.20	0.37	0.272716	-0.13334	0.382147	-0.08016	0.35912	0.10077	19.77	22.47	12.63	5.26	0.15	0.08
Q53GR7	Solute carrier family 25, member 13 (Citrin) variant (Fragment)	NA	9.83	-0.3453	-0.45325	-0.57928	-1.19639	0.030089	0.476496	5.29	29.79	21.71	39.80	-0.34	0.41	-0.55643	-0.43851	-0.55925	-1.0211	0.084724	0.582141	5.78	22.44	24.14	43.10	-0.32	0.44
Q53H26	Transferrin variant (Fragment)	NA	9.17	-0.28457	-0.91665	-1.13232	-0.81111	-1.88408	-1.01249	30.49	15.68	41.47	27.93	-1.01	0.05	-0.39473	-0.6075	-1.30332	-0.83493	0.232313	-0.96876	10.41	22.76	55.69	26.82	-0.65	0.10
Q53HW2	Ribosomal protein P0 variant (Fragment)	NA	2.50	0.004717	-0.41377	-0.69208	-0.06621	0.394589	0.3191	20.37	30.20	3.70	26.80	-0.08	0.77	0.135031	-0.12683	-0.72923	0.069945	0.656169	0.797981	12.80	38.20	6.95	37.56	0.13	0.71
Q53XJ5	Peptidyl-prolyl cis-trans isomerase	FKBP2	3.67	0.422694	0.310952	0.810948	0.879653	0.875086	0.868006	5.47	3.37	0.35	18.45	0.69	0.05	0.240991	0.361272	0.62317	0.736006	0.861762	0.892352	5.89	5.53	1.50	19.54	0.62	0.07
Q53Y06	ATPase, H+ transporting, lysosomal 31kDa, V1 subunit E isoform 1	ATP6V1E1	4.67	0.263723	-0.41024	0.174306	0.208642	-1.04292	-1.17722	32.44	1.68	6.58	41.08	-0.33	0.49	-0.12394	-0.42395	-0.11177	0.296213	-0.77156	-0.40788	14.65	19.86	17.73	23.84	-0.26	0.32
Q53Y51	D-dopachrome tautomerase	DDT	3.67	0.849615	0.097611	0.147277	-0.27332	0.049534	-0.0477	36.05	20.47	4.76	23.03	0.14	0.50	0.898569	0.126302	0.270145	-0.28014	-0.31615	-1.12156	36.97	26.65	38.48	40.70	-0.07	0.86
Q53YD7	EEF1G protein	EEF1G	3.33	0.645296	-0.1662	1.085669	0.636794	1.405817	0.82206	38.76	21.82	28.23	28.18	0.74	0.10	0.717064	0.236645	0.926545	1.01843	1.098309	0.047074	23.33	4.50	49.36	17.06	0.67	0.05
Q53Z07	60S ribosomal protein L9	RPL9	3.67	1.228874	0.099207	0.422901	0.42437	1.023365	0.401763	52.70	0.07	30.00	12.90	0.60	0.02	1.121398	0.318972	0.494724	0.515078	0.817863	0.315899	38.35	1.00	24.36	9.74	0.60	0.01
Q567R6	Single-stranded DNA-binding protein	SSBP1	2.83	1.087637	0.635815	-0.13756	0.16886	0.262266	0.39737	21.97	14.96	6.62	30.84	0.40	0.24	0.210069	0.460373	0.647284	-0.37089	0.728588	0.434548	12.24	47.93	14.36	13.11	0.35	0.11
Q59EJ3	Heat shock 70kDa protein 1A variant (Fragment)	NA	25.83	-0.19352	-0.33743	-0.47392	0.04988	-0.62543	-0.27866	7.05	25.39	16.91	8.86	-0.31	0.05	-0.41738	-0.49547	-0.22937	-0.01242	-0.82222	-0.6281	3.83	10.61	9.50	21.16	-0.43	0.13
Q59FD4	Hexokinase (Fragment)	NA	24.17	-1.01603	-0.89038	-1.46914	-1.32853	-0.76503	-0.99239	6.15	6.89	11.12	18.44	-1.08	0.02	-1.07465	-1.03502	-1.25783	-1.27953	-0.76417	-1.01994	1.94	1.06	12.50	13.15	-1.07	0.01
Q59GB4	Dihydropyrimidinase-like 2 variant (Fragment)	NA	19.83	-1.59266	-1.43594	-0.7722	-1.66226	-1.30083	-1.27781	7.67	42.29	1.13	12.47	-1.34	0.00	-1.86138	-1.82565	-0.79928	-1.4049	-0.87547	-0.99072	1.75	29.25	5.65	30.52	-1.29	0.04
Q59GX2	Solute carrier family 2 (Facilitated glucose transporter), member 1 variant (Fragment)	NA	8.50	1.130076	1.169165	2.096679	1.639758	1.040796	1.470338	1.92	22.21	20.90	28.92	1.42	0.02	1.024981	1.125696	1.739747	1.123858	0.852516	1.536669	4.93	29.74	32.92	13.66	1.23	0.01
Q59H77	T-complex protein 1 subunit gamma (Fragment)	NA	8.67	0.383847	0.220493	0.293285	0.604735	0.121974	-0.20204	8.00	15.21	15.81	16.73	0.24	0.24	0.49516	0.063373	0.829062	1.199537	0.15772	-0.05043	21.01	18.06	10.18	36.90	0.45	0.26
Q5NKV8	Intercellular adhesion molecule 1	ICAM1	2.83	1.480483	1.705221	1.145721	0.670808	0.612402	1.237177	10.99	23.07	30.15	27.82	1.14	0.04	1.687572	2.235523	1.899533	1.475243	0.779106	1.448814	26.54	20.65	32.25	27.83	1.59	0.02
Q5SRT3	Chloride intracellular channel protein	CLIC1	2.33	0.201574	0.197998	0.170543	0.032458	0.856059	-0.08125	0.18	6.76	44.39	13.18	0.23	0.11	-0.03302	0.18078	-0.03743	0.131596	0.593553	0.303289	10.46	8.27	14.18	16.35	0.19	0.28
Q5SU16	Beta 5-tubulin	TUBB	51.33	0.363029	0.400538	0.546916	0.763949	-0.2626	-0.1428	1.84	10.62	5.87	28.61	0.28	0.39	0.079708	0.279032	0.698238	0.975697	0.239162	0.403484	9.75	13.56	8.05	25.25	0.45	0.16
Q5T7Y6	Protein S100	S100A1	2.17	0.881912	0.613159	0.062758	0.137568	-0.31833	-0.67108	13.13	3.67	17.20	42.43	0.12	0.77	1.723249	1.52497	0.99493	0.069223	0.788833	0.565166	9.70	43.88	10.94	42.62	0.94	0.11
Q5TZZ9	Annexin	ANXA1	12.67	0.454789	0.563415	-0.19253	-0.46524	-0.71576	-0.50624	5.32	13.33	10.25	42.52	-0.14	0.71	0.193686	0.344362	0.56637	0.372852	0.310761	0.399269	7.38	9.47	4.34	7.06	0.36	0.02
Q5U000	Cathepsin Z	NA	1.33	1.466091	1.427042	-0.31776	-0.13084	0.828694	0.750023	1.91	9.15	3.85	52.78	0.67	0.30	0.669914	1.25956	0.678768	-0.10763	0.900273	0.712502	28.50	37.62	9.19	22.22	0.69	0.08
Q5U077	L-lactate dehydrogenase	LDHB	19.00	-0.29468	-0.17775	0.043599	0.141397	0.112825	0.187159	5.73	4.79	3.64	13.83	0.00	0.99	-0.29109	-0.12395	0.295728	0.198218	-0.15023	-0.14693	8.18	4.78	0.16	17.81	-0.04	0.82
Q5VSQ6	Procollagen-proline, 2-oxoglutarate 4-dioxygenase (Proline 4-hydroxylase), alpha polypeptide I variant (Fra	P4HA1	4.83	1.679007	1.433592	1.521844	1.310897	0.563959	0.64105	12.00	10.32	3.78	31.91	1.19	0.06	1.616033	1.517908	1.253259	1.073906	0.753578	0.942787	4.81	8.78	9.26	25.06	1.19	0.03
Q5VXV3	SET	SET	3.67	0.042669	-0.29606	0.126547	0.076876	-0.77361	-0.03302	16.53	2.43	35.52	15.78	-0.14	0.43	0.013956	0.097757	0.008075	0.380432	-0.54288	-0.49421	4.11	18.15	2.39	24.59	-0.09	0.72
Q68CM6	STXBP1 protein	stxbp1	14.83	-1.0737	-1.78653	-1.25031	-1.21219	-1.62587	-1.44474	34.24	1.87	8.87	10.45	-1.40	0.00	-1.2146	-1.53069	-0.93736	-1.14175	-1.50924	-1.35682	15.43	10.00	7.46	15.15	-1.28	0.01
Q68D11	Putative uncharacterized protein DKFZp686K23100	DKFZp686K23100	3.67	-0.15268	-0.08282	0.780101	0.434159	-0.93448	-0.61654	3.42	16.87	15.52	47.31	-0.10	0.83	-0.48232	-0.13455	0.576248	-0.0823	-0.88842	-0.46496	16.96	31.73	20.61	33.76	-0.25	0.46
Q69YG1	Myotrophin	DKFZp761E1322	2.67	1.041522	1.219841	1.265219	0.94369	0.620517	0.717215	8.73	15.69	4.74	17.14	0.97	0.02	1.147053	1.116579	1.24858	0.91208	0.734689	1.191025	1.49	16.42	22.18	5.35	1.06	0.00
Q6DEN2	DPYSL3 protein	DPYSL3	21.00	-0.99195	-0.54555	-1.0466	-1.27141	-1.03259	-1.23393	21.71	11.00	9.85	16.28	-1.02	0.01	-1.15874	-0.63427	-1.04081	-0.9917	-1.1075	-1.43281	25.43	2.41	15.88	13.34	-1.06	0.01
Q6FGJ9	GSTM3 protein	GSTM3	3.83	-0.45085	-0.67595	0.026525	-0.37598	-0.50132	-0.7936	11.01	19.60	14.28	18.50	-0.46	0.09	-0.53882	-0.70579	-0.0531	-0.64223	0.120733	-0.86529	8.17	28.48	46.53	12.21	-0.45	0.04
Q6FGX3	RAB6A protein	RAB6A	2.83	-0.48874	-0.86907	-0.5647	-0.36338	0.005512	-0.66883	18.53	9.85	32.46	12.87	-0.49	0.04	-0.76365	-1.66539	-0.91619	-0.81926	-0.41249	-0.85403	42.81	4.75	21.47	18.17	-0.91	0.03
Q6FGX9	Adenylate kinase isoenzyme 1	AK1	5.67	-0.40738	-0.06226	-0.04141	-0.85812	-0.77936	-0.87628	16.84	38.99	4.75	20.51	-0.50	0.10	-0.2338	0.138164	-0.19652	-0.73663	-0.47176	-0.95874	18.13	26.17	23.64	23.46	-0.41	0.17
Q6FHM2	GNB2 protein	GNB2	12.67	-0.31927	-0.01546	-0.51916	0.208185	-0.09065	-0.4407	14.84	34.91	17.07	5.07	-0.20	0.03	-0.22404	-0.06398	0.228417	0.512073	-0.36298	-0.09776	7.84	13.86	12.96	23.76	0.00	0.99
Q6FHU2	Epididymis secretory protein Li 35 (Fragment)	PGAM1	23.17	-0.66479	-0.4196	-0.38747	-0.33085	-0.59682	-0.22136	11.99	2.77	18.30	6.40	-0.44	0.02	-0.39761	-0.2459	-0.14468	-0.06689	-0.6753	-0.32295	7.43	3.81	17.18	13.38	-0.31	0.11
Q6FHV6	ENO2 protein	ENO2	53.67	-2.494	-2.16301	-2.25917	-2.47107	-2.57334	-2.51894	16.15	10.37	2.67	8.18	-2.41	0.00	-2.53754	-2.1394	-2.30707	-2.41226	-2.41524	-2.35189	19.39	5.15	3.10	2.04	-2.36	0.00
Q6FI52	Transgelin	TAGLN	2.83	-0.03587	0.164346	1.020397	1.959841	0.236156	-0.19507	9.80	44.48	20.98	66.16	0.52	0.39	-0.26084	0.727592	2.250123	3.068471	1.476716	0.703459	46.64	39.07	37.02	84.98	1.33	0.20
Q6FII1	Glutathione S-transferase kappa 1	LOC51064	2.00	0.302547	-0.08785	-0.35253	0.259519	-0.08619	0.890899	19.02	29.56	46.14	18.40	0.15	0.36	0.253728	0.268643	-0.43911	-0.09222	-0.1729	0.456402	0.73	16.92	30.36	18.14	0.05	0.80
Q6I9V5	SLC25A6 protein	SLC25A6	15.67	-1.1183	-0.92528	-2.14692	-2.18556	-1.92277	-1.8116	9.45	1.89	5.45	43.54	-1.69	0.04	-1.00967	-0.95766	-2.70386	-2.48386	-1.19664	-0.66395	2.55	10.76	25.82	50.92	-1.50	0.11
Q6IAL5	Putative uncharacterized protein tmp_locus_1	SUCLG1	2.33	-0.85926	-0.17197	-0.01894	-0.09015	0.054658	0.357773	33.06	3.49	14.80	23.29	-0.12	0.62	-0.54936	0.122866	0.191221	0.676146	0.039568	0.052039	32.36	23.55	0.61	22.63	0.09	0.68

				08/017 hESC-RPE to hRPE												08/023 hESC-RPE to hRPE											
				Analysis						Technical replicate CV			Biological	Fold change (mean)	Unadjusted p-value	Analysis						Technical replicate CV			Biological	Fold change (mean)	Unadjusted p-value
UniProt	Protein name	Gene name	Peptide mean	1.1	1.2	2.1	2.2	3.1	3.2	1	2	3	CV		(t-test)	1.1	1.2	2.1	2.2	3.1	3.2	1	2	3	CV		(t-test)
Q6IAT1	Epididymis secretory sperm binding protein Li 46e	GDI2	9.83	-0.10384	0.207235	0.088663	-0.04866	-0.31207	-0.68737	15.19	6.73	18.29	19.78	-0.14	0.51	-0.2032	-0.06652	-0.34225	0.005541	-0.53657	-0.28645	6.69	16.96	12.23	10.14	-0.24	0.11
Q6IAW5	CALU protein	CALU	7.83	0.404666	0.642154	0.763022	0.757726	0.336325	0.276563	11.61	0.26	2.93	15.70	0.53	0.06	0.81771	0.908931	0.957108	1.139292	0.838014	0.845461	4.47	8.92	0.36	8.14	0.92	0.01
Q6IB11	PGRMC1 protein	PGRMC1	3.83	1.314231	1.097167	0.444682	1.113767	0.661836	0.798526	10.62	32.22	6.69	18.35	0.91	0.03	1.033035	1.013925	0.308658	0.765401	0.945425	1.136365	0.94	22.20	9.34	18.02	0.87	0.03
Q6IB54	ATP synthase-coupling factor 6, mitochondrial	ATP5J	3.33	-0.46192	-0.65786	-1.04989	-1.1	-0.16942	-0.0297	9.59	2.46	6.84	33.07	-0.58	0.18	-0.74811	-0.248	-1.81522	-1.16306	-0.77935	-0.8329	24.27	31.43	2.62	32.16	-0.93	0.09
Q6IBH0	SLC25A11 protein	SLC25A11	7.00	-0.08681	0.240073	-1.57493	-1.44787	-0.76029	-0.30182	15.95	6.22	22.28	50.41	-0.66	0.29	-0.13448	0.115155	-1.09762	-1.51887	-1.29063	-0.23339	12.21	20.50	49.62	43.85	-0.69	0.21
Q6IBN1	HNRPK protein	HNRPK	10.33	-0.37843	-0.33839	0.423494	0.291965	-0.75367	-0.45226	1.96	6.44	14.72	36.29	-0.20	0.56	0.033998	0.075762	0.303335	0.450434	-0.71956	-0.33451	2.05	7.20	18.76	29.70	-0.03	0.92
Q6IBS5	DLST protein	DLST	3.83	-0.25938	-0.26106	-0.99762	-0.69731	-0.31764	0.511617	0.08	14.67	39.56	33.21	-0.34	0.35	-0.42825	-0.1661	-1.13492	-0.5704	-0.23214	-0.8579	12.81	27.32	30.20	18.23	-0.56	0.07
Q6IPT9	Elongation factor 1-alpha	EEF1A1	17.00	0.565741	0.709871	0.763959	0.683503	0.951888	0.788988	7.06	3.94	7.98	8.26	0.74	0.01	0.654266	0.761134	1.029928	0.985034	0.684365	0.745568	5.24	2.20	3.00	12.21	0.81	0.01
Q6IRT1	S-(hydroxymethyl)glutathione dehydrogenase	ADH5	5.67	0.391821	0.005495	0.008193	0.311853	0.489246	0.218593	18.82	14.83	13.23	7.09	0.24	0.06	0.773017	0.20965	-0.02193	0.194032	-0.05801	0.399431	27.27	10.57	22.23	16.04	0.25	0.18
Q6LES2	Annexin (Fragment)	ANXA4	8.50	-0.08284	0.203284	-0.26107	-0.22507	-0.15099	-0.57573	13.98	1.76	20.67	15.39	-0.18	0.29	0.146757	0.725061	-0.27426	-0.36893	-0.41718	-0.21837	27.97	4.64	9.73	33.52	-0.07	0.81
Q6LET3	HPRT1 protein (Fragment)	HPRT1	3.00	-1.16786	-1.36187	-1.10812	-0.86627	-1.90696	-1.42905	9.49	11.83	23.21	22.61	-1.31	0.02	-0.8373	-0.99582	-0.53999	-1.4387	-1.75437	-0.89524	7.76	42.68	40.91	13.20	-1.08	0.01
Q6LEU2	Sodium/potassium-transporting ATPase subunit beta (Fragment)	ATP1B1	6.67	-0.00513	0.199982	0.370763	0.093031	-0.16487	-0.01377	10.04	13.57	7.40	11.22	0.08	0.48	-0.13737	0.245409	0.546062	0.121656	-0.12589	-0.50602	18.65	20.65	18.52	22.21	0.02	0.91
Q6NVC0	SLC25A5 protein (Fragment)	SLC25A5	14.67	-0.87542	-0.85974	-1.51385	-1.78408	-0.74779	-0.75756	0.77	13.21	0.48	30.05	-1.09	0.06	-1.02877	-1.14378	-2.04969	-2.15686	-1.08891	-1.02909	5.63	5.25	2.93	35.53	-1.42	0.05
Q6NXR8	40S ribosomal protein S3a	RPS3A	4.17	-0.03971	-0.53039	0.035087	-0.14302	0.38145	0.566854	23.82	8.72	9.07	27.49	0.05	0.86	-0.00022	0.149334	0.473739	0.112115	0.301395	0.438661	7.32	17.63	6.72	10.44	0.25	0.11
Q6PKH8	ANP32A protein (Fragment)	ANP32A	6.33	-0.28841	-0.36896	0.382604	1.027294	-0.01836	-0.04332	3.95	31.08	1.22	40.15	0.12	0.74	-0.37926	-0.04908	0.954237	1.080961	-0.22343	-0.1998	16.11	6.21	1.16	53.51	0.20	0.68
Q75MT9	Malate dehydrogenase (Fragment)	MDH2	19.17	-0.76827	-0.56031	-1.60767	-1.56155	-0.39843	-0.40808	10.18	2.26	0.47	37.86	-0.88	0.13	-0.6086	-0.4037	-1.50977	-1.49591	-0.57056	-0.57101	10.03	0.68	0.02	33.79	-0.86	0.12
Q76LA1	CSTB protein	CSTB	3.67	1.611166	1.727405	1.371686	1.357116	2.228004	2.028756	5.69	0.71	9.75	27.22	1.72	0.02	1.634077	1.810028	1.595385	1.952152	1.727776	1.733579	8.61	17.40	0.28	2.31	1.74	0.00
Q7Z4W8	Heparin-binding protein HBp15	NA	1.33	-2.23022	-1.77544	-0.44116	0.784756	-0.71668	-1.56587	22.11	56.71	40.46	78.62	-0.99	0.26	0.085089	-1.8001	0.28953	0.464584	0.366677	0.207528	81.17	8.57	7.79	32.05	-0.06	0.89
Q7Z612	Acidic ribosomal phosphoprotein P1	NA	2.83	-0.58054	-0.64267	-0.2142	-0.30848	-0.06293	0.03021	3.04	4.62	4.56	20.27	-0.30	0.23	-0.07096	-0.17056	-0.30958	-0.70379	-0.26606	-0.53272	4.88	19.20	13.03	13.72	-0.34	0.10
Q86UY0	Protein BLOC1S5-TXNDC5	TXNDC5	4.00	0.571448	0.771218	0.651016	1.116566	1.323479	0.380859	9.78	22.62	44.62	9.39	0.80	0.01	0.729937	0.727459	0.770335	1.593768	1.652951	0.422139	0.12	39.30	56.91	18.07	0.98	0.02
Q86YI5	Acetyltransferase component of pyruvate dehydrogenase complex	DLAT	4.00	-0.44066	-0.8083	-0.68909	-0.97919	-0.65045	-0.75517	17.92	14.17	5.13	7.40	-0.72	0.01	-0.52519	-0.5988	-0.90857	-1.00025	-0.63686	-0.80009	3.61	4.49	7.99	13.47	-0.74	0.02
Q86Z22	Epididymis secretory protein Li 297	HEL-S-297	2.67	1.379692	0.580399	2.143443	1.704767	1.675466	1.721421	38.20	21.34	2.25	29.95	1.53	0.03	1.887952	0.547836	1.455585	0.739185	1.504093	1.267363	61.33	34.41	11.58	9.29	1.23	0.00
Q8IZ29	Tubulin, beta 2C	TUBB2C	54.83	-1.6651	-1.64149	-0.73776	-0.85478	-1.41213	-1.67265	1.16	5.73	12.73	34.40	-1.33	0.04	-1.57361	-1.81631	-1.14664	-0.91261	-1.43859	-1.77716	11.87	11.45	16.52	26.35	-1.44	0.02
Q8TBT6	Putative uncharacterized protein (Fragment)	NA	7.00	-0.5285	-0.61333	-1.39407	-1.34116	-1.00736	-0.96979	4.16	2.59	1.84	27.56	-0.98	0.05	-0.5142	-0.70472	-1.4876	-1.89984	-0.94105	-0.8798	9.32	20.07	3.00	34.86	-1.07	0.08
Q8WVX7	Ribosomal protein S19 (Fragment)	NA	4.00	1.042478	0.342435	0.676282	0.557868	0.592608	0.562171	33.65	5.80	1.49	5.71	0.63	0.00	1.152781	0.440538	0.717604	0.43409	0.468371	0.603998	34.22	13.85	6.64	11.62	0.64	0.02
R4SBI6	EPHX1	EPHX1	1.83	0.20653	-0.39543	-0.84176	-0.31896	-0.46132	-0.51368	29.08	25.35	2.57	19.32	-0.39	0.12	-0.00615	-0.06652	-0.87709	-0.46284	-0.36272	-0.27449	2.96	20.16	4.32	21.16	-0.34	0.20
V9HW12	Epididymis secretory sperm binding protein Li 2a	PRDX2	18.50	-1.33359	-1.41605	-1.45672	-1.15119	-1.26143	-1.10006	4.04	14.92	7.90	6.87	-1.29	0.00	-1.6472	-1.57596	-1.55739	-1.35821	-1.93471	-1.80607	3.49	9.75	6.30	14.22	-1.65	0.01
V9HW21	Epididymis luminal protein 76	CA2	21.50	-2.24975	-2.1827	-2.546	-2.62519	-2.57167	-2.38963	3.29	3.88	8.91	13.46	-2.43	0.00	-2.30429	-2.39006	-2.01347	-1.81926	-2.49227	-2.22036	4.20	9.50	13.29	18.19	-2.21	0.00
V9HW22	Epididymis luminal protein 33	HSPA8	35.50	-0.11382	-0.19134	0.292852	0.243798	0.001188	0.037939	3.80	2.40	1.80	14.81	0.05	0.75	-0.14317	-0.14159	0.342182	0.431473	-0.16732	-0.16578	0.08	4.38	0.08	22.86	0.03	0.90
V9HW24	Epididymis secretory protein Li 73	HEL-S-73	1.83	1.254925	-0.12195	1.818106	-0.03779	0.247152	0.619778	62.79	80.20	18.16	25.81	0.63	0.04	1.005468	0.398694	1.560548	0.49104	1.048193	0.404131	29.31	50.14	31.05	15.61	0.82	0.02
V9HW26	ATP synthase subunit alpha	ATP5A1	50.50	-0.08807	-0.26083	-0.91583	-1.03374	0.184937	0.054093	8.46	5.78	6.41	35.46	-0.34	0.40	-0.21409	-0.19861	-1.02579	-1.1847	0.350226	0.184163	0.76	7.78	8.13	43.79	-0.35	0.48
V9HW27	Crystallin, alpha B, isoform CRA_a	CRYAB	7.50	0.659075	0.395929	0.143683	0.24313	1.218964	1.084734	12.86	4.87	6.57	34.71	0.62	0.16	0.885226	0.716504	0.451519	0.689039	1.140096	1.155232	8.26	11.62	0.74	20.26	0.84	0.04
V9HW31	ATP synthase subunit beta	ATP5B	65.17	-0.92464	-0.93214	-1.75197	-1.9082	-1.07407	-0.95739	0.37	7.65	5.72	30.60	-1.26	0.05	-0.94244	-0.83034	-1.933	-2.05898	-0.71922	-0.6955	5.49	6.17	1.16	40.90	-1.20	0.10
V9HW35	Epididymis secretory protein Li 55	HEL-S-55	11.00	0.084484	0.31706	-0.49767	-0.28837	-0.0323	0.046312	11.37	10.24	3.85	20.19	-0.06	0.76	0.047843	0.387114	-0.29523	-0.34597	0.045791	0.001391	16.55	2.49	2.18	18.65	-0.03	0.88
V9HW43	Epididymis secretory protein Li 102	HSPB1	8.00	0.834902	0.992387	0.606448	0.408569	0.660775	0.555071	7.71	9.68	5.18	15.06	0.68	0.03	0.624728	0.778966	0.560204	0.693883	0.373237	0.314695	7.55	6.55	2.87	12.70	0.56	0.04
V9HW53	Dimethylarginine dimethylaminohydrolase 2, isoform CRA_a	DDAH2	3.50	-0.13183	-0.13148	0.341567	-0.78	-0.16393	-0.05811	0.02	52.36	5.18	0.81	-0.15	0.04	0.309782	-0.26749	0.418197	0.239202	0.059746	-0.45289	27.92	8.76	24.86	17.66	0.05	0.77
V9HW55	Epididymis secretory protein Li 275	HEL-S-275	2.17	0.624507	0.398523	1.005291	0.76519	0.110366	0.32667	11.05	11.74	10.58	23.35	0.54	0.11	0.503531	0.569825	1.01548	0.932453	0.252763	0.555293	3.25	4.07	14.77	21.30	0.64	0.07
V9HW56	Epididymis secretory protein Li 108	TPM4	8.50	0.768854	1.303358	0.385245	1.639843	0.598528	0.49687	25.90	57.89	4.98	20.88	0.87	0.03	0.465157	1.272935	1.078668	2.211355	1.583032	0.875413	38.59	52.83	34.00	29.17	1.25	0.03
V9HW71	Endoplasmic reticulum resident protein 29	ERP29	6.50	2.68826	2.181228	-0.0219	-0.0618	0.212946	0.818778	24.60	1.96	29.26	93.89	0.97	0.33	2.610087	2.198027	0.152585	-0.03836	0.305249	0.139381	20.06	9.35	8.12	97.23	0.89	0.36
V9HW72	Epididymis secretory sperm binding protein Li 94n	STIP1	4.17	0.719259	0.124328	0.845137	0.54618	0.229733	0.049184	28.75	14.60	8.84	19.20	0.42	0.12	0.508161	0.089082	0.610132	0.687473	-0.12476	0.003101	20.40	3.79	6.26	24.23	0.30	0.29
V9HW79	Epididymis secretory protein Li 113	HEL-S-113	5.50	1.517395	0.877641	1.008325	1.14617	0.63283	0.489037	30.85	6.75	7.04	22.80	0.95	0.04	1.591303	0.836122	0.586059	0.949018	0.490175	-0.13184	36.19	17.70	30.02	35.35	0.72	0.14
V9HW80	Epididymis luminal protein 220	VCP	24.17	0.529788	0.401832	0.624441	0.617335	0.549227	0.062508	6.27	0.35	23.63	10.22	0.46	0.04	0.438726	0.469632	0.601318	0.393747	0.425288	0.302421	1.51	10.16	6.02	4.76	0.44	0.01
V9HW88	Calreticulin, isoform CRA_b	CALR	20.17	0.842497	1.10416	0.762397	1.06445	0.940425	0.90047	12.79	14.75	1.96	2.35	0.94	0.00	0.906891	1.199803	0.707256	0.77664	0.828032	1.067036	14.31	3.40	11.69	11.01	0.91	0.01
V9HW90	Epididymis luminal protein 75	GSR	2.50	-0.17518	1.027452	0.033275	0.506461	0.810876	0.353466	55.75	22.99	22.23	11.18	0.43	0.04	-0.41738	0.679971	-0.25844	0.505866	0.648275	-0.51364	51.33	36.61	54.06	2.26	0.11	0.03
V9HW95	Epididymis secretory protein Li 84	RCN1	6.33	1.140114	0.316146	0.579386	-0.01098	0.045883	0.171307	39.32	28.54	6.14	24.86	0.37	0.18	0.807355	0.534053	0.767365	0.481853	-0.43272	0.438819	13.36	13.95	41.46	21.78	0.43	0.18
V9HW96	Chaperonin containing TCP1, subunit 2 (Beta), isoform CRA_b	CCT2	9.00	0.184354	0.072918	0.062505	0.237774	-0.31068	0.161105	5.46	8.58	22.92	7.74	0.07	0.44	0.087978	-0.0366	-0.12055	0.421898	-0.05029	0.189993	6.10	26.28	11.75	5.32	0.08	0.15
V9HW98	Epididymis luminal protein 2	YWHAE	18.67	-0.21164	-0.2477	0.358618	0.147836	-0.43028	-0.534	1.77	10.31	5.08	26.76	-0.15	0.55	-0.29109	-0.3092	0.007824	-0.21016	-0.10372	-0.24045	0.89	10.66	6.70	7.06	-0.19	0.08
V9HWA6	Epididymis luminal protein 32	DSTN	3.50	0.607166	0.98291	-0.66493	0.007236	-0.70729	-0.03867	18.31	32.36	32.20	48.69	0.03	0.94	0.044235	1.754226	-0.46333	0.236634	-0.32934	0.077422	75.21	33.65	19.81	53.66	0.22	0.58
V9HWB4	Epididymis secretory sperm binding protein Li 89n	HSPA5	36.67	0.922878	0.761957	0.818482	1.064198	0.972125	0.975126	7.88	12.01	0.15	4.66	0.92	0.00	0.871039	0.806064	0.606646	0.732214	0.806912	0.783625	3.18	6.15	1.14	5.97	0.77	0.00
V9HWB5	Epididymis secretory sperm binding protein Li 66p	PPA1	8.83	-0.88339	-0.43235	-0.4973	-0.36915	-0.86206	-1.0085	21.93	6.28	7.17	17.15	-0.68	0.04	-1.01056	-0.75701	-0.76964	-0.50699	-0.49518	-0.80682	12.40	12.84	15.22	9.36	-0.72	0.01
V9HWB7	Aconitate hydratase	ACO1	5.50	0.835674	1.321169	0.301481	0.738735	0.102966	0.451393	23.57	21.27	16.99	29.88	0.63	0.12	0.173038	0.768397	0.498132	1.293745	0.008453	0.834577	28.77	38.04	39.42	19.26	0.60	0.06
V9HWB9	L-lactate dehydrogenase	LDHA	29.67	-0.75426	-0.82133	-0.30723	-0.31338	-0.87302	-0.84828	3.29	0.30	1.21	21.71	-0.65	0.06	-0.7941	-0.8078	-0.69032	-0.67047	-1.33689	-1.20943	0.67	0.97	6.24	20.40	-0.92	0.04
V9HWC0	Epididymis luminal protein 70	MSN	14.83	0.895309	0.600821	0.338635	0.331471	0.911882	0.149557	14.38	0.35	36.52	14.43	0.54	0.05	0.627709	0.695022	0.472645	0.588743	0.71857	0.314867	3.30	5.69	19.66	5.30	0.57	0.01
V9HWC2	Epididymis secretory sperm binding protein Li 67p	PARK7	12.50	-0.59355	-0.33432	-0.20867	-0.32721	-0.35666	-0.1034	12.67	5.81	12.38	8.43	-0.32	0.05	-0.57068	-0.3232	-0.31713	-0.0595	-0.68192	-0.56118	12.10	12.59	5.91	15.42	-0.42	0.08
V9HWC3	Epididymis secretory sperm binding protein Li 1a	PDXK	1.83	0.1847	0.85042	-0.15959	-0.17451	-0.18968	-0.96896	32.06	0.73	37.29	39.62	-0.08	0.83	0.240435	0.984158	0.920943	0.430349	0.42775	0.172053	35.67	23.82	12.50	14.29	0.53	0.04
V9HWC6	Peptidyl-prolyl cis-trans isomerase	HEL-S-39	10.50	0.919863	0.633492	1.055253	0.666164	0.640383	0.541638	13.99	18.96	4.84	9.80	0.74	0.01	1.094971	0.860115	1.017981	0.48113	0.894945	0.678928	11.49	26.01	10.57	8.18	0.84	0.01
V9HWC7	Epididymis secretory sperm binding protein Li 128m	PRDX6	11.50	-0.58999	-0.17647	-0.42612	-0.52544	-0.77074	-0.50865	20.13	4.87	12.81	9.16	-0.50	0.02	-0.66065	-0.32402	-0.42613	-0.5411	-0.60694	-0.47653	16.42	5.63	6.39	2.30	-0.51	0.00
V9HWC9	Superoxide dismutase [Cu-Zn]	SOD1	11.50	0.258467	0.121539	0.578143	0.378871	0.575074	0.631229	6.71	9.75	2.75	14.23	0.42	0.07	0.207984	0.254489	0.36122	0.341927	0.327665	0.448422	2.28	0.95	5.92	5.64	0.32	0.02
V9HWD6	Epididymis secretory protein Li 1	YWHAB	9.50	-1.09836	-0.09068	0.588757	0.29884	-0.58231	-0.86106	47.47	14.16	13.62	46.26	-0.29	0.51	-1.16364	-0.33399	-0.05388	0.552897	-0.93748	-0.71997	39.58	29.31	10.64	45.10	-0.44	0.33
V9HWD9	Transketolase	TKT	22.17	1.180103	1.053294	0.901704	1.183327	0.79823	0.404755	6.21	13.76	19.17	17.86	0.92	0.03	0.893034	0.915021	0.878765	1.046679	0.263483	0.023045	1.08	8.22	11.76	28.33	0.67	0.13
V9HWE0	Annexin	ANXA5	18.83	0.811987	0.898157	1.011878	0.990748	0.182105	0.066023	4.22	1.04	5.69	29.39	0.66	0.14	0.44018	0.617715	0.650834	0.519134	0.631421	0.555293	8.69	6.45	3.73	2.34	0.57	0.00
V9HWE1	Epididymis luminal protein 113	VIM	93.50	0.075358	0.080658	0.428821	0.443495	-0.37976	-0.42696	0.26	0.72	2.31	28.29	0.04	0.89	0.607142	0.598488	1.214526	1.275097	0.497956	0.496259	0.42	2.97	0.08	29.74	0.78	0.08
V9HWE3	Carbonic anhydrase I, isoform CRA_a	CA1	15.00	-2.21643	-1.73065	-2.22655	-2.21463	-3.28338	-2.73245	23.59	0.58	26.68	33.56	-2.40	0.02	-2.24908	-1.87572	-2.09762	-2.11145	-3.06969	-2.50876	18.20	0.68	27.15	25.06	-2.32	0.01
V9HWE5	CNDP dipeptidase 2 (Metallopeptidase M20 family), isoform CRA_c	CNDP2	11.33	-0.09331	0.429299	-0.58205	-0.67231	-0.21948	-0.26111	25.34	4.42	2.04	28.30	-0.23	0.42	0.023473	0.376968	-0.50115	-0.66606	-0.18296	-0.15573	17.24	8.07	1.33	27.06	-0.18	0.50
V9HWF2	Malate dehydrogenase	MDH1	8.83	-0.59177	-0.42545	-0.51272	-0.20123	-0.67167	-0.44262	8.14	15.21	11.20	7.53	-0.47	0.02	-0.75771	-0.56711	-0.45671	-0.32907	-0.72059	-0.46791	9.33	6.25	12.35	9.80	-0.55	0.02
V9HWF4	Phosphoglycerate kinase	PGK1	32.33	-0.80598	-0.8663	-0.2748	-0.28966	-0.45805	-0.53237	2.96	0.73	3.64	18.85	-0.54	0.08	-0.68053	-0.74367	-0.32662	-0.53926	-0.74602	-0.82871	3.09	10.40	4.05	13.45	-0.64	0.03
V9HWF5	Peptidyl-prolyl cis-trans isomerase	PPIA	18.50	0.233067	0.148506	0.374857	0.318845	0.221604	0.212995	4.14	2.74	0.42	5.87	0.25	0.03	0.284458	0.372209	0.572849	0.761685	-0.08474	0.04666	4.30	9.24	6.44	23.65	0.33	0.24
V9HWG1	Epididymis secretory sperm binding protein Li 134P	SELENBP1	9.00	0.837438	0.760389	0.346162	0.051662	0.225913	0.181063	3.78	14.38	2.20	25.16	0.40	0.18	0.461979	0.422894	0.4467	0.123047	0.245978	0.215479	1.92	15.80	1.49	7.63	0.32	0.04
V9HWG2	Epididymis luminal protein 30	CRYL1	2.00	0.454933	0.712885	0.440196	0.299482	-0.02318	0.038215	12.61	6.89	3.01	19.77	0.32	0.20	0.546473	0.645592	0.868406	0.351452	0.196491	0.156962	4.86	25.07	1.94	16.63	0.46	0.08
V9HWG3	Epididymis secretory protein Li 45	TGM2	8.83	-0.7299	-0.82603	-1.5491	-0.87834	-0.96335	-0.95082	4.71	32.30	0.61	13.69	-0.98	0.02	-0.75512	-1.02109	-0.9301	-0.69723	-0.53238	-0.64776	13.00	11.39	5.65	10.80	-0.76	0.01
V9HWH2	Epididymis secretory protein Li 29	CKB	50.00	-2.16256	-2.32478	-2.1682	-2.70202	-3.12377	-3	7.94	25.87	6.06	27.49	-2.58	0.01	-2.373	-2.45987	-2.32092	-2.51815	-2.55043	-2.50876	4.26	9.65	2.04	4.48	-2.46	0.00
V9HWH9	Protein S100	S100A11	2.17	0.718063	0.758146	0.794385	-0.32291	0.895218	0.611754	1.96	52.18	13.85	15.52	0.58	0.08	0.530784	0.841666	1.033147	-0.2349	0.495025	0.79317	15.18	58.44	14.56	5.69	0.58	0.02
V9HWI0	Epididymis secretory protein Li 6	AKR1A1	4.33	0.307645	-0.4115	0.072874	0.009333	-0.32532	-0.31038	34.54	3.11	0.73	12.98	-0.11	0.42	1.374342	-0.49267	0.187003	0.129521	0.070222	-0.07143	80.57	2.82	6.94	27.64	0.20	0.26
V9HWI1	Epididymis secretory protein Li 10	BLVRB	5.83	-0.19374	-0.62734	-0.66157	-0.61546	-0.65111	-0.62894	21.09	2.26	1.09	10.06	-0.56	0.02	-0.15605	-0.58386	-0.81879	-0.5422	-0.86376	-0.56308	20.82	13.52	14.68	13.94	-0.59	0.03
V9HWI3	Cathepsin D (Lysosomal aspartyl peptidase), isoform CRA_a	CTSD	36.67	-0.49983	-0.59917	-1.03027	-1.03482	-0.13724	-0.14155	4.87	0.22	0.21	30.24	-0.57	0.16	-0.55998	-0.53164	-1.23085	-1.21309	-0.01267	0.074579	1.39	0.87	4.27	41.81	-0.58	0.25
V9HWI6	Epididymis secretory protein Li 51	HEL-S-51	9.50	3.476174	3.737929	3.4517	2.645289	2.235296	1.993698	12.79	38.53	11.81	47.05	2.92	0.02	3.209785	3.454716	2.903293	2.341777	2.180797	2.325072	11.98	27.18	7.07	39.01	2.74	0.01
V9HWJ0	Epididymis secretory sperm binding protein Li 164nA	GANAB	10.17	-0.25467	-0.53946	-0.40456	-0.14915	0.176798	0.054773	13.91	12.49	5.98	19.04	-0.19	0.35	-0.43504	-0.45322	-0.41529	-0.02212	-0.16592	-0.03434	0.89	19.15	6.44	11.97	-0.25	0.13
V9HWJ2	Isocitrate dehydrogenase [NADP]	IDH1	6.67	-0.43532	1.412354	0.441368	-0.07535	0.250916	0.312855	79.92	25.06	3.04	22.07	0.32	0.07	-0.01431	1.536486	0.827637	0.080245	-0.01183	1.108833	69.45	35.83	52.32	16.52	0.59	0.02
V9HWK2	Epididymis luminal protein 114	VCL	11.00	1.083277	0.964739	0.711023	0.224974	0.601848	0.202839	5.81	23.60	19.43	24.29	0.63	0.09	0.967648	0.97129	0.83815	0.387008	0.918219	0.595096	0.18	21.93	15.77	11.95	0.78	0.02
V9HWN7	Fructose-bisphosphate aldolase	ALDOA	31.17	0.376436	0.268718	0.597717	0.596389	1.118502	1.073093	5.28	0.07	2.23	27.87	0.67	0.10	0.420014	0.572495	0.626616	0.418692	0.54888	0.757142	7.47	10.17	10.19	5.95	0.56	0.01
V9HWP2	Epididymis luminal protein 35	HSP90B1	31.17	0.495711	0.672366	0.45187	0.564479	0.84544	0.863896	8.65	5.52	0.90	12.90	0.65	0.03	0.399728	0.575012	0.271403	0.264632	0.674682	0.717328	8.58	0.33	2.09	14.76	0.48	0.06
V9HVY3	Protein disulfide-isomerase	PDIA3	31.67	0.442529	0.558268	0.48501	0.477785	1.028582	1.009088	5.67	0.35	0.96	22.22	0.67	0.06	0.621313	0.785884	0.665267	0.5541	0.467922	0.690251	8.06	5.45	10.88	4.50	0.63	0.00
V9HVZ4	Glyceraldehyde-3-phosphate dehydrogenase	GAPDH	73.17	-0.72924	-0.7696	-0.55026	-0.4865	-0.04566	-0.03389	1.98	3.12	0.58	25.86	-0.44	0.17	-0.66933	-0.6805	-0.31146	-0.29214	-0.83396	-0.79192	0.55	0.95	2.06	18.91	-0.60	0.06
X5DNM4	Lactoylglutathione lyase (Fragment)	GLO1	4.83	-0.25608	-0.48957	-0.19556	-0.11078	-0.81974	-0.99746	11.42	4.15	8.70	25.27	-0.48	0.17	-0.62811	-0.65494	-0.58142	-0.70626	-0.72742	-0.62876	1.32	6.11	4.83	1.41	-0.65	0.00
X6R8A1	Carboxypeptidase	CTSA	1.00	0.905223	1.023722	0.215979	0.301623	0.178281	1.437632	5.80	4.20	58.08	25.28	0.68	0.09	0.543318	1.066638	-0.47243	0.081915	1.302066	2.354298	25.37	26.84	49.40	69.07	0.81	0.30
X6RJP6	Transgelin-2 (Fragment)	TAGLN2	7.83	-0.12709	-0.30026	0.064787	0.131564	0.392461	-0.12589	8.48	3.27	25.14	13.17	0.01	0.96	0.268902	0.115155	0.826068	0.832832	0.652754	0.449834	7.53	0.33	9.93	21.63	0.52	0.10
O15061	Synemin	SYNM	4.67	0.59066	0.258122	0.400787	-0.34284	0.091204	0.537067	16.23	35.66	21.68	12.42	0.26	0.16	0.702326	0.721992	0.790519	-0.07218	0.648539	0.766863	0.96	41.07	5.80	11.11	0.59	0.04
O43491	Band 4.1-like protein 2	EPB41L2	11.17	-0.78887	-0.84841	-0.97712	-0.9087	-1.20008	-1.30304	2.92	3.35	5.04	14.93	-1.00	0.02	-0.73128	-0.80433	-0.56187	-0.71742	-1.31409	-1.57292	3.58	7.62	12.65	27.14	-0.95	0.06
O75369	Filamin-B	FLNB	9.67	0.744152	0.851052	0.808226	0.62111	0.276531	0.217864	5.24	9.16	2.88	19.38	0.59	0.08	1.025089	0.886578	0.87601	0.804938	0.521196	0.416215	6.78	3.48	5.14	16.89	0.76	0.04
P07108	Acyl-CoA-binding protein	DBI	4.50	0.826823	0.892953	0.118024	0.213197	0.304759	0.334088	3.24	4.66	1.44	26.47	0.45	0.17	0.348502	0.29672	0.218664	0.602769	0.284687	0.296855	2.54	18.72	0.60	4.85	0.34	0.01
P09497	Clathrin light chain B	CLTB	3.83	0.059012	0.058043	0.536354	0.513496	0.309955	0.572377	0.05	1.12	12.83	16.48	0.34	0.14	-0.08507	0.154887	0.919605	0.506577	0.043786	0.199533	11.73	20.11	7.63	27.52	0.29	0.31
P10768	S-formylglutathione hydrolase	ESD	5.83	-0.42524	-0.28862	0.448378	-0.53381	-0.05957	0.26473	6.69	46.36	15.83	16.55	-0.10	0.54	-0.24181	-0.08908	0.417629	-0.30549	-0.42938	-0.37558	7.48	34.72	2.64	17.45	-0.17	0.33
P16070	CD44 antigen	CD44	1.00	0.313993	-0.16535	0.127275	0.434745	0.585545	0.433295	23.28	15.01	7.46	14.50	0.29	0.15	0.188695	-0.34794	0.382924	0.177392	0.259688	0.201769	26.00	10.06	2.84	12.18	0.14	0.33
P19404	NADH dehydrogenase [ubiquinone] flavoprotein 2, mitochondrial	NDUFV2	3.00	-1.15553	-0.61043	-1.51007	-0.98962	-1.58294	-3.0378	26.40	25.24	65.82	40.64	-1.48	0.07	-1.08623	-0.77311	-1.53522	-1.54146	-1.14223	-2.41215	15.29	0.31	58.51	28.57	-1.42	0.03

UniProt	Protein name	Gene name	Peptide mean	08/017 hESC-RPE to hRPE												08/023 hESC-RPE to hRPE											
				Analysis						Technical replicate CV			Biological	Fold change (mean)	Unadjusted p-value	Analysis						Technical replicate CV			Biological	Fold change (mean)	Unadjusted p-value
				1.1	1.2	2.1	2.2	3.1	3.2	1	2	3	CV		(t-test)	1.1	1.2	2.1	2.2	3.1	3.2	1	2	3	CV		(t-test)
P20674	Cytochrome c oxidase subunit 5A, mitochondrial	COX5A	5.50	0.534416	-0.39194	-1.09221	-1.71408	-1.32795	-1.2426	43.91	30.02	4.18	64.35	-0.87	0.21	0.918066	-0.21744	-0.53669	-1.84896	-1.3977	-0.89047	52.95	60.22	24.61	67.52	-0.66	0.32
P20700	Lamin-B1	LMNB1	10.67	-0.35836	-0.17389	0.392936	0.358453	-0.75509	0.954853	9.03	1.69	75.20	22.90	0.07	0.74	-0.46588	-0.07068	-0.23145	0.106735	-0.98376	-0.19245	19.25	16.50	37.84	16.50	-0.31	0.18
P21333	Filamin-A	FLNA	38.83	1.279703	1.27822	1.027436	0.960179	0.79485	1.030971	0.07	3.30	11.55	13.57	1.06	0.01	1.270175	1.293218	1.450871	1.393843	1.132476	1.327719	1.13	2.79	9.55	6.91	1.31	0.00
P21964	Catechol O-methyltransferase	COMT	4.17	-0.00296	-0.03516	0.291553	0.388972	0.124156	-0.58928	1.58	4.77	34.27	19.24	0.03	0.88	0.54076	0.341408	0.393751	0.321676	-0.43663	-0.6281	9.76	3.53	9.37	32.82	0.09	0.80
P22695	Cytochrome b-c1 complex subunit 2, mitochondrial	UQCRC2	13.50	-0.17695	-0.43929	-0.79318	-0.9709	-0.62414	-0.82556	12.82	8.70	9.86	21.39	-0.64	0.07	-0.72077	-0.62984	-0.86321	-0.84759	-1.39607	-0.45201	4.46	0.77	44.69	7.23	-0.82	0.01
P35998	26S protease regulatory subunit 7	PSMC2	2.67	0.382338	-0.523	1.035475	2.071931	0.280219	0.490949	42.97	48.72	10.31	63.42	0.62	0.33	-0.23431	0.04063	1.255168	1.070065	-0.00165	1.094868	13.43	9.06	51.30	41.19	0.54	0.28
P36575	Arrestin-C	ARR3	8.33	-0.7526	-0.91057	-0.66535	-0.49131	-0.73447	-1.23626	7.73	8.52	24.35	13.89	-0.80	0.02	-0.81477	-0.89608	-0.84173	-0.25252	-0.83433	-1.073	3.98	28.48	11.67	16.24	-0.79	0.02
P39656	Dolichyl-diphosphooligosaccharide--protein glycosyltransferase 48 kDa subunit	DDOST	4.50	0.488433	0.11787	-0.04523	0.224297	0.623968	0.812274	18.06	13.17	9.22	22.52	0.37	0.18	0.239136	0.129327	-0.03406	-0.11773	0.609497	0.768121	5.38	4.10	7.77	27.80	0.27	0.36
P46459	Vesicle-fusing ATPase	NSF	4.00	-0.41287	-0.0305	-0.08854	0.159685	-0.48362	-1.0913	18.63	12.14	29.35	26.61	-0.32	0.31	-0.18615	-0.2177	0.369471	0.074984	-0.38091	-1.18586	1.55	14.38	38.46	32.27	-0.25	0.47
P49419	Alpha-aminoadipic semialdehyde dehydrogenase	ALDH7A1	6.33	0.809743	0.542454	0.585228	0.285924	0.726373	-0.77172	13.06	14.62	67.47	17.80	0.36	0.22	0.542566	0.18078	0.363779	0.311951	0.763721	-0.54797	17.64	2.54	60.20	4.28	0.27	0.08
P49773	Histidine triad nucleotide-binding protein 1	HINT1	2.33	0.513608	0.63496	0.498019	0.510908	0.609656	0.901908	5.94	0.63	14.28	9.42	0.61	0.01	0.564365	0.715162	0.550561	0.915295	0.397929	0.814022	7.38	17.78	20.25	4.64	0.66	0.00
P52272	Heterogeneous nuclear ribonucleoprotein M	HNRNPM	6.50	0.536453	-0.14066	1.122413	0.732243	0.25467	0.502667	32.59	19.01	12.13	26.99	0.50	0.15	0.62629	-0.01462	0.830202	0.316822	0.075923	-0.14622	30.91	24.90	10.87	21.30	0.28	0.25
P54819	Adenylate kinase 2, mitochondrial	AK2	7.67	0.637852	-0.09129	-0.6231	-0.35425	0.122102	-0.2084	35.00	13.14	16.13	27.12	-0.09	0.73	0.374258	0.125494	-0.71794	-0.36763	0.514987	0.879724	12.16	17.09	17.78	40.22	0.13	0.75
P60174	Triosephosphate isomerase	TPI1	26.67	-0.14984	-0.15053	0.126547	0.117781	-0.03016	0.322607	0.03	0.43	17.20	11.26	0.04	0.72	-0.26611	-0.17827	0.056087	0.161235	-0.48126	-0.3549	4.30	5.15	6.19	18.78	-0.18	0.37
P63208	S-phase kinase-associated protein 1	SKP1	4.83	-0.14831	-0.21274	0.984276	0.980363	-0.19016	-0.07171	3.16	0.19	5.80	49.48	0.22	0.62	0.076803	-0.2835	0.936496	1.191935	-0.33376	0.411393	17.57	12.49	35.73	46.67	0.33	0.46
Q06830	Peroxiredoxin-1	PRDX1	17.50	0.03462	0.066588	0.110187	0.35907	0.106996	0.038215	1.57	12.17	3.37	7.31	0.12	0.18	0.179438	0.234586	0.125468	0.141009	-0.30487	-0.3915	2.70	0.76	4.24	19.58	0.00	0.99
Q08722	Leukocyte surface antigen CD47	CD47	2.33	-1.16168	-1.18702	-1.03081	-1.11534	-1.15083	-1.21412	1.24	4.14	3.10	4.29	-1.14	0.00	-1.17943	-0.60683	-0.40554	-1.187	-0.85363	-0.93521	27.70	37.39	4.00	5.58	-0.86	0.00
Q13813	Spectrin alpha chain, non-erythrocytic 1	SPTAN1	53.83	-0.32691	-0.4504	-0.34477	-0.34217	-0.58168	-0.44763	6.05	0.13	6.57	6.02	-0.42	0.01	-0.40921	-0.41335	-0.34514	-0.27984	-0.64395	-0.41843	0.20	3.20	11.03	7.42	-0.42	0.02
Q15435	Protein phosphatase 1 regulatory subunit 7	PPP1R7	1.83	-0.15006	-1.30823	-0.49505	-1.34585	-0.76218	-0.81757	53.90	40.53	2.71	8.80	-0.81	0.00	0.223832	-0.95424	-0.89063	-0.03732	-0.2522	0.012046	54.73	40.64	12.92	9.93	-0.32	0.09
Q15835	Rhodopsin kinase	GRK1	6.17	-0.7526	-0.71904	-1.15339	-0.83255	1.000629	-0.61048	1.64	15.66	71.67	55.45	-0.51	0.29	-1.13593	-0.82487	-0.90762	-1.18815	-0.82881	-0.56181	15.19	13.71	13.05	13.30	-0.91	0.01
Q16891	MIC	IMMT	13.83	-0.26174	-0.10817	-0.70412	-0.48798	0.325545	0.606287	7.52	10.57	13.72	38.03	-0.11	0.77	-0.03302	-0.22155	-0.49543	-0.3313	0.524075	0.709101	9.23	8.04	9.06	38.69	0.03	0.94
Q5SSJ5	Heterochromatin protein 1-binding protein 3	HP1BP3	4.83	-1.51297	-1.2538	-2.13756	-2.00753	-1.14556	-1.33656	12.67	6.37	9.35	28.10	-1.57	0.03	-1.48079	-1.02198	-1.40353	-1.4881	-1.705	-1.35025	22.30	4.14	17.30	10.43	-1.41	0.00
Q9H9B4	Sideroflexin-1	SFXN1	3.67	0.12476	-1.75993	-0.00717	0.032458	-0.03516	0.028963	81.15	1.94	3.14	19.94	-0.27	0.43	-0.07096	-1.82643	-0.59054	0.461838	0.004336	0.82482	76.79	49.41	39.16	38.21	-0.20	0.67
Q9UBQ7	Glyoxylate reductase/hydroxypyruvate reductase	GRHPR	3.50	-0.14875	1.821495	0.447212	0.313763	0.582935	0.657451	83.91	6.54	3.65	28.19	0.61	0.04	-0.63626	1.322501	0.541723	0.390285	0.434957	0.786731	83.55	7.42	17.16	6.73	0.47	0.03
Q9UBS4	DnaJ homolog subfamily B member 11	DNAJB11	2.17	0.880202	1.044762	-0.13872	-0.18976	0.981783	0.488534	8.06	2.50	23.94	36.50	0.51	0.28	0.062815	1.034511	-0.15057	0.537853	1.524434	1.489413	45.90	33.12	1.72	47.16	0.75	0.20
A0A024QZ30	Succinate dehydrogenase [ubiquinone] flavoprotein subunit, mitochondrial	SDHA	5.67	-0.92726	-0.17111	-1.07368	-0.82179	-0.2347	-1.12975	36.24	12.31	42.51	15.32	-0.73	0.03	-0.41475	0.089082	-1.09168	-0.78321	-0.506	-1.37225	24.45	15.06	41.23	32.60	-0.68	0.12
A0A024R0R9	N-ethylmaleimide-sensitive factor attachment protein, alpha, isoform CRA_c	NAPA	5.17	-0.41209	0.610675	-0.40737	0.174944	0.485857	-0.46099	48.13	28.16	44.81	9.52	0.00	0.98	-0.30944	0.234774	-0.77137	-0.35886	-0.86037	-0.12739	26.36	20.08	35.17	20.66	-0.37	0.16
A0A024R0Y5	ATP-dependent 6-phosphofructokinase	PFKM	8.83	1.104488	-0.53396	-0.64035	0.83217	-0.48109	-0.32624	72.66	66.50	7.58	30.03	-0.01	0.97	1.078671	-0.39093	-0.8059	0.792462	-0.6796	-0.37364	66.39	71.20	14.94	34.02	-0.06	0.83
A0A024R3E3	Apolipoprotein A-I, isoform CRA_a	APOA1	2.33	2.634717	2.317136	1.794767	2.021072	0.964121	0.421314	15.50	11.07	26.30	53.91	1.69	0.08	2.171774	2.231446	1.251878	1.297959	2.338172	1.711849	2.92	2.26	30.22	30.95	1.83	0.02
A0A024R407	Microtubule-associated protein	MAP2	5.00	-1.06791	-0.55947	0.639152	0.50609	-0.70364	-0.26858	24.67	6.52	21.16	52.68	-0.24	0.62	-0.99694	-0.46899	1.229713	0.158072	-0.87244	-0.23641	25.59	50.24	30.68	61.45	-0.20	0.70
A0A024R6Y2	Nuclear transport factor 2, isoform CRA_a	NUTF2	2.00	0.782079	0.676647	0.416561	0.635097	0.728146	1.284069	5.17	10.69	26.92	17.77	0.75	0.03	0.732708	0.602702	0.619222	0.652581	0.786198	0.999358	6.37	1.63	10.43	10.08	0.73	0.01
A0A024R713	Dihydrolipoyl dehydrogenase	DLD	10.67	-0.49566	-0.34537	-1.13931	-1.07091	0.228302	0.216161	7.36	3.35	0.60	44.49	-0.43	0.37	-0.41387	-0.34626	-1.44151	-0.90267	-0.04729	-0.19539	3.31	26.11	7.25	33.54	-0.56	0.22
A0A024R845	RAB14, member RAS oncogene family, isoform CRA_a	RAB14	5.67	-0.40738	-0.94854	-0.04223	0.214788	-0.1014	-0.2421	26.22	12.56	6.89	25.01	-0.25	0.37	-1.03011	-0.68651	-0.13049	0.078574	-0.15737	-0.71399	16.76	10.23	26.95	28.10	-0.44	0.21
A0A024R9C1	Polyadenylate-binding protein	PABPC1	4.17	0.577397	0.673556	0.776547	1.252455	1.046627	0.57769	4.71	23.12	22.78	14.09	0.82	0.02	0.782475	0.412004	0.888913	1.093196	0.62634	0.010984	18.06	10.00	29.71	22.78	0.64	0.08
A0A024R9U3	OCIA domain containing 1, isoform CRA_a	OCIAD1	2.00	0.016247	0.196176	0.210492	-0.02298	0.914703	1.340186	8.81	11.42	20.70	45.07	0.44	0.33	0.51385	0.930943	-0.03198	-0.07351	0.666628	0.991518	20.30	2.04	15.86	30.20	0.50	0.21
A0A024RAJ6	Beta-hexosaminidase	HEXB	3.17	0.844252	0.86852	0.339474	0.209326	-0.24643	-0.40902	1.19	6.37	7.96	39.99	0.27	0.52	-0.22891	0.45057	0.071582	0.538027	0.122443	-0.16266	32.70	22.66	13.93	11.67	0.13	0.30
A0A024RB18	Retinol dehydrogenase 5 (11-cis and 9-cis), isoform CRA_a	RDH5	10.67	-1.29058	-1.17797	-1.12017	-1.16887	-1.12927	-1.23327	5.52	2.39	5.10	3.09	-1.19	0.00	-1.30267	-1.04681	-1.53375	-1.03603	-0.97601	-1.2732	12.51	24.16	14.51	5.11	-1.19	0.00
A0A024RB75	Citrate synthase	CS	14.00	-0.54852	-0.86872	-1.73599	-1.76872	-0.66944	-1.21839	15.63	1.60	26.59	34.31	-1.13	0.07	-0.57003	-0.90101	-1.75334	-1.73873	-0.8846	-1.21141	16.15	0.72	15.95	33.38	-1.18	0.06
A0A087WWD4	Neural cell adhesion molecule 1	NCAM1	9.33	-1.64157	-1.37608	-1.16463	-1.99172	-1.59852	-1.40415	12.98	39.46	9.51	0.79	-1.53	0.00	-1.60994	-1.32237	-1.1156	-1.61918	-1.89111	-1.69584	14.05	24.43	9.56	15.42	-1.54	0.01
A0A0A0MSE2	Hydroxyacyl-coenzyme A dehydrogenase, mitochondrial	HADH	3.50	1.307645	1.007668	0.218487	0.026	-0.1014	0.581095	14.65	9.42	32.84	41.73	0.51	0.26	1.140111	1.356805	-0.19739	-0.34405	-0.11241	0.148681	10.60	7.18	12.76	60.12	0.33	0.55
A0A0C4DFU2	Superoxide dismutase	SOD2	6.00	-0.1575	0.02017	-1.38021	-1.20002	-0.46267	-0.31196	8.70	8.82	7.38	39.00	-0.58	0.25	-0.02319	0.074507	-0.88548	-0.82105	0.283158	0.32242	4.79	3.16	1.92	37.16	-0.17	0.67
A4D2P1	Ras-related C3 botulinum toxin substrate 1 (Rho family, small GTP binding protein Rac1)	RAC1	3.83	-0.05027	0.396938	0.385042	0.205673	-0.03516	0.041651	21.75	8.78	3.76	10.17	0.16	0.20	-0.14802	0.236084	-0.01808	-0.45452	-0.34527	0.062943	18.72	21.23	19.88	9.89	-0.11	0.31
A8KA82	DnaJ (Hsp40) homolog, subfamily C, member 3	DNAJC3	2.83	0.995564	0.414136	0.312194	1.21615	0.636252	0.215431	28.12	42.91	20.48	13.68	0.63	0.03	1.05366	0.761264	0.413075	0.696688	0.249114	0.292142	14.28	13.86	2.11	22.45	0.58	0.09
B2ZZ89	Epididymis luminal protein 102	SPTBN1	26.83	-0.44039	-0.57324	-0.42612	-0.23125	-0.42897	-0.41994	6.51	9.54	0.44	6.26	-0.42	0.01	-0.51045	-0.62235	-0.50258	-0.4874	-0.57795	-0.68273	5.48	0.74	5.13	4.66	-0.56	0.00
B7Z6Z4	Myosin light polypeptide 6	MYL6	6.17	1.144216	1.177918	0.509608	0.679394	0.895143	1.297241	1.65	8.31	19.58	20.18	0.95	0.03	1.361267	1.234305	1.24858	1.479864	1.274722	1.642361	6.22	11.31	17.92	5.99	1.37	0.00
C7DJS2	Glutathione S-transferase pi (Fragment)	GSTP1	19.67	1.409268	1.127546	1.993092	1.195694	3.276978	2.89305	13.76	38.12	18.71	71.36	1.98	0.07	1.257033	1.192397	0.745947	1.274471	1.705885	0.889003	3.17	25.62	39.00	11.03	1.18	0.01
D0PNI1	Epididymis luminal protein 4	YWHAZ	14.33	-0.63117	-0.90274	-0.3634	-0.27523	-1.43576	-1.52421	13.27	4.32	4.33	37.98	-0.86	0.13	-0.55579	-0.558	-0.45186	-0.34919	-0.69928	-0.80832	0.11	5.03	5.34	12.16	-0.57	0.03
D3DSQ1	N-acylsphingosine amidohydrolase (Acid ceramidase) 1, isoform CRA_c	ASAH1	10.17	-0.15838	-0.06842	-0.98497	-1.24868	-0.52567	-0.73268	4.41	12.89	10.13	34.24	-0.62	0.17	0.082401	-0.04977	-1.06867	-1.08926	0.123013	-0.24832	6.47	1.01	18.10	36.54	-0.38	0.40
D3DTE6	SH3 domain-binding glutamic acid-rich-like protein	SH3BGRL	2.67	0.586471	0.792855	-0.00027	0.367268	0.28229	0.238975	10.10	17.92	2.12	19.59	0.38	0.14	0.450159	0.797975	0.252197	0.822738	0.485457	0.418779	16.97	27.61	3.27	6.32	0.54	0.01
D6R956	Ubiquitin carboxyl-terminal hydrolase	UCHL1	6.67	0.601079	0.343034	1.378328	1.491257	0.661659	0.956384	12.61	5.53	14.40	34.67	0.91	0.08	0.184845	0.012725	1.550561	1.429694	0.57285	0.976908	8.43	5.92	19.68	46.94	0.79	0.19
E5RJD8	Tubulin-specific chaperone A	TBCA	2.33	1.576605	1.683756	0.658088	1.008809	0.233424	0.81549	5.25	17.11	28.15	40.67	1.00	0.09	0.673216	1.666914	0.703221	0.827439	0.185442	0.517802	46.87	6.08	16.22	31.04	0.76	0.08
E7EQR4	Ezrin	EZR	23.17	1.584306	1.908408	1.192397	1.184951	1.559935	1.022925	15.82	0.36	26.02	21.44	1.41	0.01	1.508392	1.967914	1.382341	1.497579	1.806084	1.332742	22.33	5.65	22.99	10.95	1.58	0.00
E7EVA0	Microtubule-associated protein	MAP4	2.83	-0.47445	-0.1628	-0.27929	-0.77819	0.270633	-1.40116	15.22	24.21	73.85	7.28	-0.47	0.03	-0.37897	-0.28941	0.057304	-1.17498	0.258305	-0.42216	4.39	56.98	32.75	14.40	-0.32	0.14
E9PCA1	T-complex protein 1 subunit epsilon	CCT5	9.83	0.105311	0.065318	-0.41301	-0.18976	-0.8833	-0.54283	1.96	10.92	16.61	26.97	-0.31	0.31	-0.03639	-0.24486	0.059248	0.17113	-0.77015	-0.60969	10.20	5.48	7.86	26.84	-0.24	0.42
E9PK25	Cofilin-1	CFL1	10.67	0.531151	0.404129	0.130668	0.33168	0.429676	0.328922	6.22	9.84	4.94	8.12	0.36	0.03	0.541814	0.649952	0.795776	0.707855	0.441215	0.31779	5.30	4.31	6.05	12.78	0.58	0.03
F5GWE5	Phosphatidylinositol transfer protein alpha isoform	PITPNA	1.83	0.398257	0.214616	0.394146	0.681368	0.147944	-0.21199	8.99	14.03	17.55	19.29	0.27	0.24	0.334842	-0.51801	0.485716	0.405988	0.449112	-0.86218	40.62	3.91	60.18	20.70	0.05	0.83
F6XZQ7	Glutathione S-transferase Mu 2	GSTM2	3.83	0.849068	0.603822	-0.2099	0.143547	-0.36222	-0.15596	11.99	17.24	10.09	37.80	0.14	0.68	0.095996	0.570419	0.020104	0.248153	-0.01601	0.094367	23.05	11.15	5.41	11.26	0.17	0.19
H3BR70	Pyruvate kinase	PKM	62.67	-1.41576	0.064592	-0.14194	0.062053	-0.67792	-0.65452	66.80	9.98	1.15	23.39	-0.46	0.16	-1.79905	0.240567	-0.08737	-0.02854	-0.86979	-0.91853	86.09	2.88	2.39	28.41	-0.58	0.16
J3QQX2	Rho GDP-dissociation inhibitor 1	ARHGDIA	5.00	0.926507	0.686117	0.672961	0.263039	-0.0283	-0.1225	11.76	19.96	4.62	29.51	0.40	0.26	1.174107	0.640088	0.870345	0.808544	0.279415	0.242152	25.88	3.03	1.83	23.29	0.67	0.08
Q0Z7D8	Cytochrome c oxidase subunit 2	COX2	3.17	-0.85998	-1.00952	-2.0748	-2.397	-1.37431	-1.07483	7.32	15.73	14.63	40.88	-1.47	0.07	-1.2743	-1.16635	-2.29958	-2.48386	-1.41524	-1.28306	5.29	9.02	6.47	38.03	-1.65	0.05
Q86VG2	Splicing factor proline/glutamine-rich (Polypyrimidine tract binding protein associated)	SFPQ	5.33	-0.71496	-0.9242	-0.37676	-0.01817	-0.40378	-0.61135	10.24	17.49	10.16	21.71	-0.51	0.11	-0.47865	-0.90019	0.380982	0.174712	-0.4459	-0.55111	20.51	10.09	5.15	37.52	-0.30	0.41
Q8WVC2	40S ribosomal protein S21	RPS21	1.83	0.303983	0.199486	0.864774	0.987162	0.709803	0.2586	5.12	5.99	21.94	24.15	0.55	0.11	0.601665	0.982709	1.213872	1.143639	-0.00083	0.556749	18.57	3.44	26.99	29.43	0.75	0.10
X5D2M8	Major vault protein isoform A (Fragment)	MVP	5.17	1.118246	0.822354	0.70843	0.361739	1.926482	-0.19572	14.45	16.91	88.59	22.93	0.79	0.03	1.058613	0.747942	0.261937	-0.20492	1.944405	0.072951	15.17	22.68	80.71	39.87	0.65	0.17
O43678	NADH dehydrogenase [ubiquinone] 1 alpha subcomplex subunit 2	NDUFA2	2.80	-0.68142	-0.75961	-2.00611	-1.16709	-0.09288		3.83	40.00	NA	46.93	-0.94	0.21	-0.8782	-0.99714	-1.3724	-1.26006	-0.48097		5.83	5.50	NA	29.02	-1.00	0.06
P10909	Clusterin	CLU	3.00	0.022462	-0.04884	0.232993	0.836901	-0.18857		3.49	29.17	NA	28.71	0.17	0.66	0.253361	-0.55314	0.111013	0.807679	-0.01079		38.53	33.50	NA	23.47	0.12	0.64
P17568	NADH dehydrogenase [ubiquinone] 1 beta subcomplex subunit 7	NDUFB7	1.00	-0.64557	-1.27938	-1.7011	-0.34384	-1.51864		30.57	62.02	NA	22.97	-1.10	0.02	-1.1854	-0.52022	-1.49045	-0.34662	-2.55529		32.04	53.30	NA	52.86	-1.22	0.12
P17900	Ganglioside GM2 activator	GM2A	1.80	0.784138	1.00946	1.028086	0.26633	0.927401		11.02	36.49	NA	8.53	0.80	0.01	0.406521	0.380697	-0.01526	0.081438	0.495612		1.27	4.74	NA	16.09	0.27	0.16
P20941	Phosducin	PDC	5.00	-1.27287	-0.69365	-1.04769	-0.95804	-1.18253		28.01	4.39	NA	8.17	-1.03	0.00	-1.64995	-0.68014	-0.87848	-0.98419	-0.3309		45.82	5.18	NA	29.06	-0.90	0.08
P30050	60S ribosomal protein L12	RPL12	3.40	0.537945	0.631411	0.876083	0.639673	0.91099		4.58	11.56	NA	11.22	0.72	0.02	0.624728	0.638673	0.799125	0.89291	0.447445		0.68	4.60	NA	13.89	0.68	0.03
Q10567	AP-1 complex subunit beta-1	AP1B1	4.60	0.919135	0.933771	1.232881	1.284301	0.19974		0.72	2.52	NA	34.55	0.91	0.13	0.720659	1.110978	1.432238	0.318239	-0.31358		19.01	52.04	NA	42.00	0.65	0.35
Q17R60	Interphotoreceptor matrix proteoglycan 1	IMPG1	4.80	-0.69379	-0.74267	-2.21543	-1.85193	-1.19271		2.40	17.72	NA	42.04	-1.34	0.08	-0.92778	-1.02736	-2.11286	-1.89608	-0.84878		4.88	10.61	NA	37.55	-1.36	0.07
Q5XKP0	MIC	MIC13	1.00	-1.29347	-1.20179	-1.3307	-2.05057	-1.06908		4.49	34.57	NA	19.64	-1.39	0.02	-0.55193	-0.61322	-1.23383	-1.32812	-0.58569		3.00	4.62	NA	25.34	-0.86	0.07
Q9NPJ3	Acyl-coenzyme A thioesterase 13	ACOT13	1.20	-0.35609	0.27045	-0.72019	-0.86195	-0.34561		30.23	6.94	NA	26.39	-0.40	0.21	-1.12489	-0.40691	-0.47102	-0.08899	-0.07861		34.48	18.62	NA	21.79	-0.43	0.21
Q9Y512	Sorting and assembly machinery component 50 homolog	SAMM50	3.40	-0.37307	-0.7987	-0.70758	-0.93972	0.330778		20.71	11.35	NA	44.66	-0.50	0.41	0.090656	-0.61525	-0.74738	-1.01599	0.645105		33.92	13.13	NA	52.74	-0.33	0.74
A0A024R694	Actinin, alpha 1, isoform CRA_a	ACTN1	13.80	1.270185	0.39116	0.626674	1.438543	0.834585		41.80	38.77	NA	9.37	0.91	0.01	1.675618	0.688611	1.133795	1.308185	1.352738		46.57	8.54	NA	4.60	1.23	0.00
A0A024R6G4	Aldehyde dehydrogenase 6 family, member A1, isoform CRA_b	ALDH6A1	7.80	-0.02542	-0.1205	0.16204	0.556614	-0.13294		4.66	19.22	NA	20.01	0.09	0.77	-0.05221	-0.04817	0.063613	0.450249	0.045791		0.20	18.84	NA	11.60	0.09	0.45
A0A024R8Q1	Glucosidase, alpha acid (Pompe disease, glycogen storage disease type II), isoform CRA_a	GAA	3.00	-0.31682	0.342734	0.136708	-0.10737	0.642265		31.78	11.93	NA	25.74	0.14	0.40	-0.15118	0.953307	0.092144	-0.13259	0.00907		51.64	10.99	NA	21.36	0.15	0.44
A0A024RAV0	Branched-chain-amino-acid aminotransferase	BCAT1	2.40	0.576605	1.281034	1.094384	0.119724	0.400002		33.86	46.03	NA	19.69	0.69	0.05	0.677332	2.515058	1.17394	0.982226	0.725715		79.59	9.38	NA	42.38	1.21	0.05
A0A087WUD3	Oligosaccharyltransferase complex subunit	OSTC	1.00	-0.06688	0.148677	0.204983	0.334819	-0.40915		10.55	6.36	NA	22.95	0.04	0.88	-0.08251	0.213836	-0.52171	-0.42474	-0.35609		14.47	4.75	NA	20.72	-0.23	0.26
A8K492	cDNA FLJ76789, highly similar to Homo sapiens methionine-tRNA synthetase (MARS), mRNA	NA	1.40	0.443109	0.648587	0.205673	0.690218	-0.74381		10.05	23.53	NA	41.70	0.25	0.86	0.634641	1.151419	0.096174	0.626338	-0.65107		25.06	25.70	NA	49.00	0.37	0.70
A8K761	NADH dehydrogenase (Ubiquinone) 1 beta subcomplex, 10, 22kDa, isoform CRA_b	NDUFB10	2.80	-0.58025	0.137849	-0.62473	-0.30034	0.055062		34.49	15.83	NA	17.44	-0.26	0.30	-0.77711	-0.05754	-0.76361	-0.68785	0.280777		34.55	3.71	NA	36.48	-0.40	0.44
B2R4R9	HCG26477	RPS28	2.00	-1.2535	-1.35359	-0.16642	0.402997	-0.38723		4.90	27.55	NA	46.21	-0.55	0.33	-0.64241	-0.72315	0.777586	0.877471	0.2346		3.96	4.89	NA	48.36	0.10	0.80
B2R8R5	cDNA, FLJ94025, highly similar to Homo sapiens tripartite motif-containing 28 (TRIM28), mRNA	NA	2.20	0.717704	0.723271	0.843218	0.726678	0.197669		0.27	5.71	NA	20.82	0.64	0.09	0.578667	0.556552	0.838858	0.44655	-0.28281		1.08	19.11	NA	31.77	0.43	0.41
B3KN29	cDNA FLJ13371 fis, clone PLACE1000656, highly similar to PRA1 family protein 2	NA	1.60	1.634812	1.355995	0.932861	0.564835	0.719683		13.62	17.94	NA	32.62	1.04	0.06	1.16661	0.75148	0.933717	-0.09356	1.293065		20.21	48.33	NA	26.39	0.81	0.07
B4DZ22	cDNA FLJ54042, highly similar to ATP-binding cassette sub-family D member 3	NA	2.80	-0.17695	-0.71094	-0.53367	-0.37052	-0.34898		25.88	7.99	NA	3.61	-0.43	0.01	-0.41943	-0.5512	-0.27794	-0.77887	-1.10528		6.45	24.31	NA	22.51	-0.63	0.07
E5KNY5	Leucine-rich PPR-motif containing	LRPPRC	9.40	-1.00335	0.751622	-0.19739	-0.94123	0.161381		76.77	35.67	NA	24.38	-0.25	0.49	-1.46377	0.539824	0.025833	-1.14398	0.260379		84.97	54.39	NA	24.68	-0.36	0.43
H7BYD0	NADH dehydrogenase [ubiquinone] 1 alpha subcomplex subunit 5 (Fragment)	NDUFA5	6.40	-0.63178	-0.9188	-1.0598	-0.95446	-0.45632		14.02	5.16	NA	19.26	-0.80	0.04	-0.70818	-1.07299	-1.09114	-0.68095	-0.45409		17.79	19.97	NA	17.59	-0.80	0.04
J3KQ32	Obg-like ATPase 1	OLA1	2.20	0.647309	0.757023	0.598417	0.742679	-0.01468		5.37	7.06	NA	25.57	0.55	0.19	-0.19967	0.423387	-0.05572	0.645501	0.06608		30.07	33.71	NA	9.82	0.18	0.15
Q53GL5	Isocitrate dehydrogenase 2 (NADP+), mitochondrial variant (Fragment)	NA	7.60	1.08671	0.766874	0.425075	0.556972	0.814298		15.61	6.46	NA	15.32	0.73	0.03	1.113824	1.0044	0.742472	1.026628	0.171372		5.36	13.88	NA	29.65	0.81	0.12
Q59ET3	Chaperonin containing TCP1, subunit 6A isoform a variant (Fragment)	NA	8.80	-0.44306	0.001897	0.124117	0.123601	-0.406		21.64	0.03	NA	18.83	-0.12	0.39	-0.56646	0.148142	-0.01118	-0.30238	-0.10084		34.33	14.22	NA	2.34	-0.17	0.04
Q5U0I6	H.sapiens ras-related Hrab1A protein	RAB1A	7.00	-0.43799	0.716356	-0.40001	0.504233	-0.03832		53.74	42.92	NA	10.00	0.07	0.42	-0.39992	-0.11796	0	0.426975	0.404662		13.78	20.78	NA	22.27	0.06	0.61
Q6IBT3	CCT7 protein	CCT7	8.20	-0.21918	-0.48717	-0.16345	-0.1509	0.996712		13.10	0.61	NA	54.52	0.00	0.74	-0.06912	-0.5998	0.06119	0.175829	0.777177		25.72	5.62	NA	38.64	0.07	0.62
Q6JSD8	Aquaporin 1 splice variant 2 (Fragment)	AQP1	1.00	0.841618	2.084512	2.275493	2.145216	0.309504		57.41	6.38	NA	57.29	1.53	0.14	0.604838	1.870517	1.804424	1.616907	0.343071		58.34	9.18	NA	42.96	1.25	0.11
Q7Z518	NADH dehydrogenase	NA	3.40	-0.22239	0.08639	-0.28251	0.323908	-0.46981		15.08	29.29	NA	18.11	-0.11	0.37	-0.14027	0.098992	0.032528	0.125363	-0.06492		11.70	4.55	NA	5.13	0.01	0.96

				08/017 hESC-RPE to hRPE												08/023 hESC-RPE to hRPE											
				Analysis						Technical replicate CV			Biological	Fold change (mean)	Unadjusted p-value	Analysis						Technical replicate CV			Biological	Fold change (mean)	Unadjusted p-value
UniProt	Protein name	Gene name	Peptide mean	1.1	1.2	2.1	2.2	3.1	3.2	1	2	3	CV		(t-test)	1.1	1.2	2.1	2.2	3.1	3.2	1	2	3	CV		(t-test)
Q8N1C8	HSPA9 protein (Fragment)	HSPA9	21.80	0.908373	0.422091	-0.55688	-0.361	0.061238		23.61	9.59	NA	39.53	0.09	0.81	0.78591	0.258178	-0.79884	-0.67449	0.295341		25.58	6.09	NA	40.55	-0.03	0.95
Q9BTT5	Similar to NADH dehydrogenase (Ubiquinone) 1 alpha subcomplex, 9 (39kD) (Fragment)	NA	5.80	-0.68648	-0.12112	-1.21913	-1.09944	-0.41735		27.36	5.86	NA	27.47	-0.71	0.12	-0.45053	0.335133	-1.63151	-0.88299	-2.74464		37.58	35.89	NA	82.00	-1.07	0.22
V9HW58	Epididymis secretory protein Li 77	DCTN2	4.20	0.239584	0.346322	0.111169	-0.64484	-0.46691		5.23	36.23	NA	27.75	-0.08	0.58	-0.18515	-0.26882	-0.22729	-0.14481	-0.40645		4.10	4.04	NA	7.98	-0.25	0.06
Q8N5K1	CDGSH iron-sulfur domain-containing protein 2	CISD2	1.40	-0.39232	-0.23737	-0.72588	-0.34685	-0.39053		7.59	18.47	NA	7.33	-0.42	0.02	-0.45563	0	-0.60933	-0.72574	-0.6433		22.15	5.70	NA	18.63	-0.49	0.07
Q9NQC3	Reticulon-4	RTN4	6.00	0.528151	-0.91988	-0.77583	-0.96471	-1.27152		65.56	9.24	NA	46.00	-0.68	0.13	-0.325	-0.89444	-0.46507	-0.67691	-1.05457		27.55	10.36	NA	18.06	-0.68	0.04
Q9P0J0	NADH dehydrogenase [ubiquinone] 1 alpha subcomplex subunit 13	NDUFA13	2.80	0.024785	-0.84978	-0.87783	-1.22692	-0.70159		41.60	17.03	NA	23.96	-0.73	0.06	0.563624	-0.6626	-0.88081	-1.60837	-1.17921		56.73	34.92	NA	55.25	-0.75	0.17
A0A087X0I2	Complement factor I	CFI	1.00	1.771221	1.565084	1.074888	1.405785	2.067188		10.09	16.15	NA	27.93	1.58	0.02	1.834888	1.188535	1.596808	1.442842	2.369161		31.16	7.54	NA	35.86	1.69	0.02
A0A4Z1	CADM1 protein	CADM1	3.80	-0.56823	-0.71094	0.536537	-0.54684	-0.98477		6.99	50.74	NA	39.69	-0.45	0.20	-0.55032	-0.52085	-2.15733	-0.5077	-0.56107		1.44	73.06	NA	20.84	-0.86	0.09
B3KSH1	Eukaryotic translation initiation factor 3 subunit F	EIF3F	1.20	-0.56852	-0.1438	0.477986	-1.18436	-2.14278		20.67	73.52	NA	57.01	-0.71	0.25	-0.44814	-0.00995	-0.54848	-0.30051	-0.83618		21.31	12.12	NA	21.13	-0.43	0.11
D6RIE3	Cytochrome c oxidase subunit 7A2, mitochondrial	COX7A2	2.60	-0.62569	-0.7987	-1.89252	-1.52544	-0.11103		8.47	17.90	NA	50.19	-0.99	0.21	-0.83652	-0.98487	-1.70221	-1.50519	-0.91313		7.27	9.64	NA	25.11	-1.19	0.04
H0YDL9	Tetraspanin (Fragment)	CD81	1.00	-0.21392	-0.06782	0.233219	0.121664	0.2488		7.15	5.46	NA	13.77	0.06	0.51	-1.03684	-0.12179	-0.96673	-0.57303	0.565324		43.40	19.18	NA	52.29	-0.43	0.59
O15537	Retinoschisin	RS1	3.80	-0.74697	-0.54167	-1.40597	-1.05166		-0.52117	10.05	17.28	NA	23.90	-0.85	0.07	-0.3272	-1.25718	-0.19362	-1.32243		-0.78895	44.07	52.67	NA	4.80	-0.78	0.00
O60262	Guanine nucleotide-binding protein G(I)/G(S)/G(O) subunit gamma-7	GNG7	1.00	0.332871	-0.41782	-0.94513	-0.85812		-0.36303	35.99	4.26	NA	30.34	-0.45	0.22	0.251337	-1.01084	-1.96327	-1.44624		-1.19612	58.20	25.07	NA	52.40	-1.07	0.11
O75475	PC4 and SFRS1-interacting protein	PSIP1	2.80	-1.58496	-1.40696	-1.37401	-1.01817		-0.73951	8.71	17.35	NA	26.63	-1.22	0.04	-2.06912	-1.28216	-0.7406	-0.94285		-0.81207	37.64	9.90	NA	28.58	-1.17	0.06
P15531	Nucleoside diphosphate kinase A	NME1	7.00	-0.33409	-1.45872	-0.21266	-0.67651		-1.90537	52.49	22.54	NA	45.73	-0.92	0.13	-0.22916	-1.713	-0.051	-0.08337		-0.9675	66.93	1.59	NA	35.02	-0.61	0.16
P16615	Sarcoplasmic/endoplasmic reticulum calcium ATPase 2	ATP2A2	6.40	0.197116	0.484342	0.173602	0.011165		-0.2066	14.03	7.95	NA	18.99	0.13	0.68	0.45975	0.527341	0.125932	-0.16306		-0.00784	3.31	14.12	NA	21.17	0.19	0.45
P62136	Serine/threonine-protein phosphatase PP1-alpha catalytic subunit	PPP1CA	3.20	0.190577	0.014731	0.765987	0.362765		0.795598	8.61	19.64	NA	23.27	0.43	0.14	0.650781	0.342104	0.620704	-0.93608		0.372397	15.07	69.67	NA	15.91	0.21	0.36
Q5TZA2	Rootletin	CROCC	10.80	0.262247	0.549085	-0.47097	-1.16827		-0.36213	14.01	33.53	NA	43.24	-0.24	0.54	-0.00637	0.347483	-0.33585	-1.1202		0.202141	17.26	37.52	NA	30.72	-0.18	0.74
Q7Z7H5	Transmembrane emp24 domain-containing protein 4	TMED4	2.20	0.487591	0.438293	0.032498	0.01769		0.235257	2.42	0.73	NA	15.19	0.24	0.20	0.586423	-0.07021	0.975499	0.000252		0.560673	31.64	46.06	NA	10.44	0.41	0.04
Q9H2U2	Inorganic pyrophosphatase 2, mitochondrial	PPA2	5.40	-0.1151	-0.04806	1.03638	0.205216		-0.06017	3.29	39.65	NA	32.10	0.20	0.56	0.622452	-0.18026	-0.50977	-0.20056		0.164431	38.36	15.10	NA	21.53	-0.02	0.96
Q9UHQ9	NADH-cytochrome b5 reductase 1	CYB5R1	4.20	-0.27454	-0.62881	-0.49805	-0.197		-0.75875	17.28	14.70	NA	14.60	-0.47	0.05	-0.1019	-0.3697	0.004548	0.061264		-1.10074	13.09	2.78	NA	36.55	-0.30	0.33
A0A024R1U4	RAB5C, member RAS oncogene family, isoform CRA_a	RAB5C	4.20	-0.92726	-1.56817	-0.20409	-0.69859		1.101363	30.91	24.00	NA	82.54	-0.46	0.80	-2.51357	-1.57727	-0.20117	-0.69886		0.213449	44.35	24.15	NA	62.94	-0.96	0.37
A0A087WZX2	NADH dehydrogenase [ubiquinone] 1 beta subcomplex subunit 6	NDUFB6	1.20	0.369139	-0.19721	-0.56862	-1.22015		-0.29643	27.41	31.40	NA	32.49	-0.38	0.33	0.65927	-0.24251	-0.67206	-1.87464		-0.37836	42.81	55.75	NA	47.22	-0.50	0.38
B2R761	cDNA, FLJ93299, highly similar to Homo sapiens sterol carrier protein 2 (SCP2), mRNA	NA	5.00	0.280675	0.485969	0.197837	0.004085		1.38319	10.05	9.48	NA	49.68	0.47	0.25	0.596457	0.674459	0.538416	0.487984		0.981469	3.82	2.47	NA	17.34	0.66	0.04
B4DLN1	Uncharacterized protein	NA	2.40	-0.23045	0.489624	-0.60404	-0.2958		0.383786	34.58	15.05	NA	27.54	-0.05	0.94	-0.05016	0.998677	-1.24218	-0.89608		-0.59118	49.26	16.88	NA	60.94	-0.36	0.48
B4E1S3	cDNA FLJ57860, highly similar to Transmembrane protein 109	NA	1.40	-0.11553	0.188034	0.268414	-0.09493		0.405609	14.82	17.71	NA	13.95	0.13	0.27	-0.0853	-0.01595	-0.3317	-0.50663		0.355595	3.40	8.56	NA	26.71	-0.12	0.88
B7Z4V9	cDNA FLJ57541, highly similar to Skeletal muscle and kidney-enriched inositol phosphatase (EC 3.1.3.56)	NA	2.60	0.017997	-0.22046	-0.44116	-0.41622		0.458816	11.66	1.22	NA	31.80	-0.12	0.94	0.087978	-0.07624	-0.92962	-0.24832		0.817793	8.04	32.79	NA	48.15	-0.07	0.87
E9PEB5	Far upstream element-binding protein 1	FUBP1	3.00	1.535095	0.976653	1.503732	1.750222		1.036149	27.03	12.05	NA	21.01	1.36	0.02	1.180888	0.928514	1.24057	1.493551		1.009602	12.34	12.37	NA	14.08	1.17	0.01
H0YI09	Methyltransferase-like protein 7A (Fragment)	METTL7A	1.20	-1.45246	-0.82771	-0.68482	-1.49986		0.869631	30.15	38.92	NA	84.20	-0.72	0.56	-1.52488	-0.85764	0.205776	-0.57641		-0.45847	32.13	37.42	NA	33.42	-0.64	0.18
H7C1U8	MIC	APOO	1.60	-0.27121	-0.29933	-0.93142	-0.93921		-0.27524	1.38	0.38	NA	23.99	-0.54	0.15	0.091479	-0.0357	-0.8911	-0.52576		-0.19367	6.23	17.81	NA	24.41	-0.31	0.31
Q53G17	NADH dehydrogenase (Ubiquinone) Fe-S protein 8, 23kDa (NADH-coenzyme Q reductase) variant (Fragmen	NA	1.60	-0.36316	-0.20397	-0.55649	-0.99172		-0.84548	7.79	21.17	NA	22.03	-0.59	0.07	-0.75697	-0.17329	-0.55888	-1.00025		0.224486	28.22	21.47	NA	36.10	-0.45	0.37
Q53GF8	Isocitrate dehydrogenase [NAD] subunit, mitochondrial (Fragment)	NA	7.00	-0.2201	-0.4629	-0.62228	-0.69389		-0.77221	11.87	3.51	NA	16.11	-0.55	0.04	-0.39214	-0.51171	-0.79884	-0.88765		-0.59862	5.86	4.35	NA	13.46	-0.64	0.03
Q53X12	V-type proton ATPase subunit a	NA	7.80	-0.27216	-0.31719	-0.50254	-0.74909		-0.36467	2.21	12.05	NA	11.54	-0.44	0.05	-0.76365	-0.77651	-0.34804	-0.60031		-0.4233	0.63	12.33	NA	12.57	-0.58	0.04
Q53YE7	Amine oxidase [flavin-containing]	MAOA	3.20	-1.40165	-1.11947	-1.15516	-1.17243		-1.00283	13.79	0.85	NA	8.90	-1.17	0.00	0.076595	-0.73851	-1.02321	-1.07218		-0.90644	38.92	2.40	NA	30.16	-0.73	0.07
Q5VZU9	Tripeptidyl-peptidase 2	TPP2	3.80	-0.8394	-1.23134	-0.43649	-1.05988		0.18343	19.09	30.09	NA	45.94	-0.68	0.28	-1.30808	-1.47205	-0.35806	-0.74972		0.468367	8.03	19.08	NA	62.76	-0.68	0.46
Q6FGB3	PCBD protein (Fragment)	PCBD	2.80	0.546057	0.068037	0.813363	0.516632		-0.75041	23.22	14.49	NA	44.29	0.24	0.88	0.401222	0.247266	0.686493	0.465498		-0.98689	7.54	10.81	NA	47.63	0.16	0.96
Q6FHU3	PSME1 protein (Fragment)	PSME1	2.60	0.623996	0.470297	-0.06035	0.441167		-0.18042	7.53	24.34	NA	24.87	0.26	0.47	1.156717	-0.00995	-0.34161	0.008555		0.136462	54.26	17.08	NA	30.59	0.19	0.49
Q6IAQ1	LYPLA1 protein	LYPLA1	4.20	0.41548	-0.07981	-0.55104	-0.18316		0.279593	24.04	17.93	NA	22.12	-0.02	0.91	-0.12752	0.280121	-0.54885	-0.696		0.294762	19.85	7.21	NA	30.31	-0.16	0.79
Q8WYJ5	Protein kinase C inhibitor-2	NA	2.60	-0.99078	-1.62207	-0.60646	-1.1017		-0.82181	30.46	24.04	NA	16.81	-1.03	0.02	-0.93279	-2.43464	-0.54294	-1.14733		-0.68823	67.61	29.20	NA	27.43	-1.15	0.07
P08247	Synaptophysin	SYP	3.40	-1.13723	-1.7484	-0.9631	-1.76783		-1.80665	29.52	38.45	NA	17.31	-1.48	0.01	-1.22583	-1.90101	-0.92721	-1.54293		-1.89087	32.50	29.73	NA	23.53	-1.50	0.01
P21912	Succinate dehydrogenase [ubiquinone] iron-sulfur subunit, mitochondrial	SDHB	1.60	-0.8077	-1.24073	-1.43076	-2.39007		-0.64853	21.07	45.36	NA	38.11	-1.30	0.09	-0.67632	-0.76074	-1.47982	-1.36016		-0.4256	4.14	5.86	NA	32.56	-0.94	0.10
A0A024R8T0	Integrin beta	ITGB4	2.20	1.472631	1.594171	1.434225	2.570083		1.935328	5.95	52.96	NA	19.63	1.80	0.01	1.325605	1.081709	0.957238	2.058359		2.515707	11.93	51.49	NA	48.50	1.59	0.05
A0A024RAI1	ARP3 actin-related protein 3 homolog (Yeast), isoform CRA_a	ACTR3	3.80	0.5114	0.212651	0.379959	0.579905		0.387248	14.59	9.78	NA	4.29	0.41	0.01	-0.17497	0.825219	0.219099	0.381401		1.021041	47.15	7.95	NA	28.40	0.45	0.15
B1ANH0	Guanylate kinase (Fragment)	GUK1	3.60	-0.15926	-0.72217	-0.72807	-1.92914		0.138429	27.25	55.69	NA	43.85	-0.68	0.33	-0.26216	-0.58882	-0.21608	-0.82551		0.196921	15.94	29.43	NA	27.66	-0.34	0.38
B3KUN1	Serine/threonine-protein phosphatase	PPP2CA	3.20	-0.79432	-0.72688	-0.20837	0.269833		-0.3685	3.31	23.23	NA	27.95	-0.37	0.25	-0.1725	-0.29399	0.141616	0.556848		-1.03434	5.95	20.21	NA	45.68	-0.16	0.53
O75643	U5 small nuclear ribonucleoprotein 200 kDa helicase	SNRNP200	5.20	0.301908	2.743679	-0.70455		0.352181	-0.35488	97.45	NA	33.98	97.73	0.47	0.72	0.316484	1.886876	-0.48053		-0.04005	-0.63606	70.17	NA	28.80	74.16	0.21	0.87
P04899	Guanine nucleotide-binding protein G(i) subunit alpha-2	GNAI2	6.80	0.539571	0.43619	0.323253		-0.70068	0.159588	5.06	NA	40.96	23.59	0.15	0.52	0.32805	0.285916	0.015355		-0.08189	0.981035	2.06	NA	49.86	18.15	0.31	0.18
P09471	Guanine nucleotide-binding protein G(o) subunit alpha	GNAO1	9.60	-0.95142	-0.74045	-0.36819		-1.72451	-1.18459	10.32	NA	26.16	35.55	-0.99	0.11	-1.28761	-0.96969	0.099249		-1.11913	-0.7161	15.52	NA	19.63	48.42	-0.80	0.23
P62316	Small nuclear ribonucleoprotein Sm D2	SNRPD2	1.20	1.130795	0.880943	0.764583		-0.90303	1.514737	12.22	NA	96.83	10.19	0.68	0.08	0.936502	0.562085	0.704619		-0.63653	0.71902	18.25	NA	61.95	20.18	0.46	0.16
Q5JWF2	Guanine nucleotide-binding protein G(s) subunit alpha isoforms XLas	GNAS	4.00	-2.04093	-1.01719	-1.34342		-0.69479	-0.77245	48.17	NA	3.81	28.11	-1.17	0.04	-1.20851	-0.78066	-0.03587		-1.82222	-0.68686	20.82	NA	52.94	44.59	-0.91	0.18
A0A024R1K7	Tyrosine 3-monooxygenase/tryptophan 5-monooxygenase activation protein, eta polypeptide, isoform CR	YWHAH	6.20	0.809182	-0.14779	-0.71583		-0.93101	-0.06697	45.26	NA	41.13	42.91	-0.21	0.45	0.863351	-0.17254	-0.08764		-1.26407	-0.63141	48.70	NA	30.52	43.66	-0.26	0.61
A0A024RDG9	3-hydroxybutyrate dehydrogenase, type 2, isoform CRA_b	BDH2	3.40	1.562545	1.370972	1.134537		1.444339	0.962634	9.38	NA	23.39	12.31	1.30	0.01	1.5323	1.362901	1.290254		1.524003	1.13344	8.29	NA	19.03	5.70	1.37	0.00
A0A024RDH8	Ribosomal protein L34, isoform CRA_a	RPL34	1.40	0.644793	0.489354	0.799115		1.01937	1.727623	7.61	NA	34.03	31.76	0.94	0.06	0.154849	0.674459	0.795339		0.633289	0.913988	25.20	NA	13.71	13.41	0.63	0.03
A8K2M0	Proteasome (Prosome, macropain) 26S subunit, ATPase, 4, isoform CRA_b	PSMC4	5.40	0.935181	1.106366	0.350534		1.477698	0.04768	8.38	NA	64.86	23.38	0.78	0.07	0.008956	0.989051	0.793004		0.770162	-0.03939	46.27	NA	38.67	13.01	0.50	0.05
B4E0E1	cDNA FLJ53442, highly similar to Poly (ADP-ribose) polymerase 1 (EC 2.4.2.30)	NA	4.00	0.006481	-0.26699	0.235922		0.252911	0.244353	13.36	NA	0.42	13.99	0.09	0.44	-0.63524	0.015997	0.24239		-0.24434	-0.36259	31.39	NA	5.79	22.35	-0.20	0.57
B4E1U9	cDNA FLJ54776, highly similar to Cell division control protein 42 homolog	NA	3.20	-0.94496	-0.25018	-0.13989		0.067921	-0.2606	33.41	NA	16.03	16.86	-0.31	0.23	-0.56873	-0.55703	-0.06231		-0.45183	-0.37725	0.57	NA	3.65	18.31	-0.40	0.14
B5BU83	Stathmin	STMN1	2.80	-0.1597	-0.6708	0.482356		-1.51624	-1.78068	24.79	NA	12.92	65.50	-0.73	0.48	-0.4756	-0.43434	-0.00787		-1.51336	-1.53892	2.02	NA	1.25	47.13	-0.79	0.28
B5BUB1	RuvB-like 1 (Fragment)	RUVBL1	3.60	0.28278	0.221958	-0.32671		0.010239	1.052594	2.98	NA	48.98	31.61	0.25	0.61	0.731785	0.542096	-0.96821		0.753455	1.623168	9.28	NA	41.38	63.21	0.54	0.70
C9JAB2	Serine/arginine-rich-splicing factor 7	SRSF7	3.00	-1.06667	-1.31672	0.733197		-0.39898	-0.45013	12.22	NA	2.51	67.06	-0.50	0.65	-1.00659	-0.75032	0.059248		-0.8131	-0.48939	12.53	NA	15.80	35.43	-0.60	0.22
F8W6I7	Heterogeneous nuclear ribonucleoprotein A1	HNRNPA1	12.80	-1.45031	-1.53478	0.378124		-0.52889	-0.30636	4.14	NA	10.89	59.12	-0.69	0.45	-0.68229	-0.52814	0.622677		-0.62884	-0.56498	7.55	NA	3.13	53.30	-0.36	0.68
Q5TZP7	APEX nuclease (Multifunctional DNA repair enzyme) 1	APEX1	5.80	-0.68237	-0.72939	0.529209		-0.90827	-1.00737	2.30	NA	4.86	59.48	-0.56	0.50	-0.71572	-0.60851	1.016231		-0.99566	-0.74165	5.25	NA	12.42	77.47	-0.41	0.80
Q68DI0	AP complex subunit beta	DKFZp781K0743	5.60	-0.23926	0.683874	0.162751		0.142382	0.462102	43.76	NA	15.61	5.55	0.24	0.03	-0.54776	0.453952	0.360234		0.376745	0.27985	47.21	NA	4.75	11.86	0.18	0.24
Q8NCF7	cDNA FLJ90278 fis, clone NT2RP1000325, highly similar to Phosphate carrier protein, mitochondrialprecurs	NA	7.40	-1.15246	-1.27156	-2.08151		-0.90513	-0.68328	5.83	NA	10.85	41.30	-1.22	0.07	-1.23973	-1.22386	-2.06549		-1.15138	-1.14031	0.78	NA	0.54	31.21	-1.36	0.04
R4GMT0	Alpha-centractin	ACTR1A	3.00	-1.04824	-0.39593	0.373835		-0.37467	0.004798	31.44	NA	18.49	36.31	-0.29	0.63	-0.2844	-0.65494	0.349152		-0.86866	0.619689	18.06	NA	67.10	26.99	-0.17	0.76
V9HW65	Annexin	HEL-S-270	22.60	1.006677	0.604321	0.131636		-0.08812	-0.10067	19.59	NA	0.62	34.72	0.31	0.41	0.901035	0.653178	0.423868		0.297025	0.350734	12.12	NA	2.63	17.28	0.53	0.07
P62906	60S ribosomal protein L10a	RPL10A	2.40	-0.26268	0.10469	0.119976		0.518329	0.433295	17.91	NA	4.17	19.58	0.18	0.40	-0.06751	0.378664	0.264889		0.507588	0.517354	21.70	NA	0.48	12.45	0.32	0.10
E9PM69	26S protease regulatory subunit 6A	PSMC3	3.40	0.708819	0.247928	0.223263		0.105437	0.42616	22.40	NA	15.66	10.31	0.34	0.05	0.635911	0.012506	0.088579		0.479388	0.185294	30.09	NA	14.36	10.10	0.28	0.09
P49006	MARCKS-related protein	MARCKSL1	1.00	1.493755	1.169756		2.014497	0.854514	-0.39056	15.81	NA	57.50	52.66	1.03	0.15	1.050596	1.169852		1.839123	1.453649	1.322763	5.84	NA	6.41	25.95	1.37	0.02
Q08380	Galectin-3-binding protein	LGALS3BP	5.20	1.58896	1.811201		0.254445	1.024396	1.362343	10.87	NA	16.49	45.92	1.21	0.13	1.008303	0.7056		0.142839	0.583239	2.584213	14.78	NA	84.88	61.46	1.00	0.17
Q96AQ6	Pre-B-cell leukemia transcription factor-interacting protein 1	PBXIP1	2.20	0.464237	0.430708		0.530752	-0.33234	-0.1618	1.64	NA	8.35	26.77	0.19	0.43	0.345919	0.950117		-0.00354	-0.31127	0.223203	29.19	NA	25.90	29.44	0.24	0.47
Q99714	3-hydroxyacyl-CoA dehydrogenase type-2	HSD17B10	3.80	0.491936	0.319496		0.53294	0.571598	0.01742	8.44	NA	26.83	7.43	0.39	0.03	0.160937	0.202774		0.42378	0.058356	-0.38646	2.05	NA	21.63	19.47	0.09	0.48
A0A024QZ64	Fructose-bisphosphate aldolase	ALDOC	29.40	-1.13636	-1.60061		-1.49243	-2.01299	-1.71889	22.56	NA	14.36	17.46	-1.59	0.01	-1.46437	-1.82175		-1.46563	-1.74741	-1.70001	17.43	NA	2.32	9.17	-1.64	0.00
A0A024R5H0	Barrier to autointegration factor 1, isoform CRA_a	BANF1	1.20	0.097053	1.049722		1.279313	1.286136	0.732352	45.07	NA	26.81	21.32	0.89	0.04	-0.00417	0.525506		0.875547	1.562316	0.504281	25.67	NA	49.65	28.05	0.69	0.09
A0A0C4DFR6	Protein SEC13 homolog	SEC13	2.00	1.781277	1.817946		0.703068	0.56254	0.196686	1.80	NA	17.84	54.77	1.01	0.15	2.702528	0.636973		0.756317	1.054678	-0.19956	86.88	NA	57.87	59.16	0.99	0.12
A4D1K0	V-type proton ATPase subunit F	ATP6V1F	2.80	-0.5285	-0.55723		0.391386	-0.43898	-1.02653	1.41	NA	28.41	44.04	-0.43	0.49	-0.42265	0.264062		-0.0791	0.283328	0.317447	33.04	NA	1.67	14.98	0.07	0.74
A8K0T9	cDNA FLJ75422, highly similar to Homo sapiens capping protein (actin filament) muscle Z-line, alpha 1, mR	NA	2.00	0.733418	-0.00323		0.770145	0.519695	-1.04071	35.34	NA	69.80	27.99	0.20	0.43	0.660102	0.064006		1.605508	0.292812	-0.0638	28.81	NA	17.39	58.87	0.51	0.27
B4DHQ3	Phosphoserine aminotransferase	NA	2.00	1.026912	1.875056		1.669234	0.53945	0.91073	40.41	NA	18.10	31.03	1.20	0.05	0.548122	1.25698		1.361013	0.575489	0.238346	34.06	NA	16.45	31.75	0.80	0.08
B4E0U6	cDNA FLJ53687, highly similar to Hsc70-interacting protein	NA	5.00	0.395716	0.114192		0.240678	0.083573	-0.06711	13.75	NA	7.38	9.40	0.15	0.17	0.249864	0.217442		-0.3313	-0.23774	-0.16674	1.59	NA	3.48	21.33	-0.05	0.62
D3DR22	Hydroxysteroid (17-beta) dehydrogenase 12, isoform CRA_a	HSD17B12	3.60	-0.20414	0.191529		-0.47657	0.126847	-0.65853	19.27	NA	37.57	16.61	-0.20	0.21	-0.00637	0.061896		-0.21221	0.356377	-0.19833	3.35	NA	26.86	11.17	0.00	0.73
H9ZYJ2	Thioredoxin	TXN	2.80	0.329741	0.451124		0.628462	0.316705	-0.97395	5.95	NA	59.35	27.25	0.15	0.51	0.21951	0.003741		0.807968	-0.03178	-0.71927	10.56	NA	33.07	40.62	0.06	0.65
Q32Q12	Nucleoside diphosphate kinase	NME1-NME2	7.20	-0.09751	0.339887		-2.2619	-0.10892	0.098462	21.28	NA	10.15	63.47	-0.41	0.45	0.169341	0.803162		-0.0791	0.45757	0.337071	30.58	NA	5.90	20.67	0.34	0.27
Q53FR4	Vacuolar protein sorting 35 variant (Fragment)	NA	2.00	-0.05907	0.392896		2.029168	0.629219	0.395115	21.97	NA	11.45	73.19	0.68	0.26	0.169341	0.149732		1.536201	0.820675	0.54109	0.96	NA	13.66	49.08	0.64	0.19
Q53FT8	Proteasome subunit beta type (Fragment)	NA	2.00	0.160952	0.199817		0.738261	-0.23634	-0.11973	1.90	NA	5.71	32.59	0.15	0.45	0.212341	0.597177		0.622409	-0.26134	-0.00633	18.75	NA	12.47	25.21	0.23	0.32
Q53HG2	NADH dehydrogenase (Ubiquinone) Fe-S protein 2, 49kDa (NADH-coenzyme Q reductase) variant (Fragmen	NA	5.40	-0.15684	-0.26083		-0.97555	-0.63582	-0.21823	5.09	NA	20.33	25.75	-0.45	0.14	-0.90052	-0.21207		-0.76766	0.127184	-0.09022	33.12	NA	10.64	28.94	-0.37	0.21
Q53HT6	B-cell receptor-associated protein 31 variant (Fragment)	NA	3.00	0.05447	0.186867		0.222265	-0.01003	-0.10537	6.48	NA	4.67	9.66	0.07	0.37	0.029067	0.412004		0.401792	0.281628	0.256382	18.66	NA	1.24	6.24	0.28	0.03
Q59FA2	Splicing factor, arginine/serine-rich 1 (Splicing factor 2, alternate splicing factor) variant (Fragment)	NA	4.80	-0.25186	-0.55416		0.35763	-0.39034	-0.46138	14.76	NA	3.48	32.88	-0.26	0.60	-0.22148	-0.16684		0.83863	-0.05286	-0.6364	2.68	NA	28.22	47.54	-0.05	0.81
Q59FF0	EBNA-2 co-activator variant (Fragment)	NA	3.80	1.019746	1.34221		-0.1085	0.825464	0.256117	15.74	NA	27.55	43.51	0.67	0.29	0.845048	0.605746		0.145807	0.443193	0.322078	11.70	NA	5.93	20.64	0.47	0.13
Q5QTS3	FWP004	NA	2.20	0.230553	0.073099		0.252676	0.288827	0.590042	7.71	NA	14.71	10.49	0.29	0.08	0.615604	-0.05457		0.857415	0.04499	0.531346	32.27	NA	23.61	22.92	0.40	0.13
Q6FGG2	VAMP3 protein	VAMP3	2.60	0.125121	-0.58004		-1.04403	-0.16973	-0.08739	33.89	NA	4.03	31.42	-0.35	0.25	-0.94792	-0.70113		-0.98719	-0.06082	-0.65482	12.07	NA	28.71	24.74	-0.67	0.06
Q6IB71	Proteasome subunit alpha type	PSMA3	1.40	0.668394	0.00019		-0.62397	-0.45211	0.237776	32.18	NA	33.18	33.44	-0.03	0.68	-0.13304	0.119421		-3.00531	-0.41883	0.196173	12.34	NA	29.69	71.08	-0.65	0.40
Q6IBG1	MYL9 protein	MYL9	5.40	0.93116	0.31187		0.241124	0.408137	1.020767	29.90	NA	29.58	17.70	0.58	0.07	0.711856	0.425686		0.980565	-0.07817	0.895693	13.98	NA	46.00	18.91	0.59	0.06
Q6NTA2	HNRNPL protein (Fragment)	HNRNPL	6.00	-0.65515	-0.52957		-1.23063	-0.14155	-0.04347	6.15	NA	4.81	37.92	-0.52	0.19	-0.1485	-0.42662		0.590252	-0.24728	-0.10488	13.59	NA	6.97	35.19	-0.07	0.89
P35080	Profilin-2	PFN2	3.60	-1.14243	-0.32122		0.703877	-0.08619	-0.0817	39.20	NA	0.22	48.04	-0.19	0.94	-1.14462	-0.63257		1.271338	-0.18554	-0.61099	24.84	NA	20.70	82.02	-0.26	0.99
P62280	40S ribosomal protein S11	RPS11	2.20	0.591836	0.393619		0.629826	1.177416	1.003177	9.70	NA	8.53	22.62	0.76	0.06	0.894567	0.865083		0.43521	0.529958	0.727579	1.45	NA	9.67	15.53	0.69	0.04

				08/017 hESC-RPE to hRPE												08/023 hESC-RPE to hRPE											
				Analysis						Technical replicate CV			Biological	Fold change (mean)	Unadjusted p-value	Analysis						Technical replicate CV			Biological	Fold change (mean)	Unadjusted p-value
UniProt	Protein name	Gene name	Peptide mean	1.1	1.2	2.1	2.2	3.1	3.2	1	2	3	CV		(t-test)	1.1	1.2	2.1	2.2	3.1	3.2	1	2	3	CV		(t-test)
K7ELC7	60S ribosomal protein L27 (Fragment)	RPL27	1.20	0.5154	0.220493		1.613635	1.183829	0.682852	14.40	NA	24.31	42.52	0.84	0.11	0.569247	0.554753		1.349275	1.09415	0.712763	0.71	NA	18.58	27.44	0.86	0.05
P04271	Protein S100-B	S100B	2.40	-0.18611		0.516666	-0.13518	-0.58482	-0.57426	NA	31.42	0.52	27.79	-0.19	0.48	2.362759		1.477197	1.572883	0.486047	0.675179	NA	4.69	9.26	57.98	1.31	0.10
P04844	Dolichyl-diphosphooligosaccharide--protein glycosyltransferase subunit 2	RPN2	7.20	0.903646		0.163934	-0.03914	0.390969	0.449531	NA	9.94	2.87	29.45	0.37	0.20	1.256116		0.208407	-0.23223	0.610446	0.503073	NA	21.43	5.26	43.46	0.47	0.24
P16401	Histone H1.5	HIST1H1B	5.40	1.29687		1.619964	1.947181	1.535453	2.209149	NA	15.97	32.43	21.19	1.72	0.01	1.599353		0.849711	0.860893	-0.25862	0.045208	NA	0.55	14.84	54.71	0.62	0.25
Q15417	Calponin-3	CNN3	2.40	-0.04661		0.150863	0.426334	0.289285	0.500371	NA	13.46	10.33	15.51	0.26	0.25	1.006668		0.300462	1.083107	-0.2071	0.076204	NA	37.45	13.84	34.61	0.45	0.23
Q8N142	Adenylosuccinate synthetase isozyme 1	ADSSL1	5.00	1.800788		1.573164	2.225313	0.245973	1.138237	NA	31.43	42.39	38.16	1.40	0.06	1.369856		1.122449	1.982673	0.702395	0.740579	NA	40.96	1.87	29.59	1.18	0.04
A0A024R3Q3	ADP-ribosylation factor 1, isoform CRA_a	ARF1	6.00	-0.43586		0.206822	0.236432	-0.55289	-1.00538	NA	1.45	22.00	35.95	-0.31	0.38	-0.43712		-0.12744	0.176053	-0.31641	-0.52192	NA	14.82	10.06	19.22	-0.25	0.21
A0A024R6K8	Tryptophanyl-tRNA synthetase, isoform CRA_a	WARS	2.20	0.328331		0.165826	0.338993	-0.08693	0.530526	NA	8.48	29.81	2.97	0.26	0.01	0.371212		0.311923	0.0769	0.238636	-0.22636	NA	11.49	22.60	11.98	0.15	0.21
A0A024RBX6	Pirin (Iron-binding nuclear protein), isoform CRA_a	PIR	1.20	1.10522		0.819984	0.549246	0.3034	0.653772	NA	13.23	17.09	22.37	0.69	0.05	1.125421		0.013852	1.069824	-0.39798	-0.18148	NA	49.56	10.59	44.86	0.33	0.38
A8K4W8	cDNA FLJ77917, highly similar to Homo sapiens ubiquitin-conjugating enzyme E2L 3 (UBE2L3), transcript va	NA	2.40	0.127645		0.163224	0.117538	-0.97512	-0.61027	NA	2.24	17.79	32.16	-0.24	0.63	0.337446		0.628091	1.004913	-0.27304	-0.1998	NA	18.36	3.59	35.83	0.30	0.42
B4DMA2	cDNA FLJ54023, highly similar to Heat shock protein HSP 90-beta	NA	27.00	0.332402		0.510353	0.764104	0.240186	-0.17594	NA	12.41	20.26	20.63	0.33	0.20	0.220826		0.411363	0.399498	0.10966	-0.17156	NA	0.58	13.74	14.74	0.19	0.26
B4DTA2	cDNA FLJ60148, highly similar to Homo sapiens heterogeneous nuclear ribonucleoprotein D-like (HNRPDL)	NA	4.60	-0.58467		0.132362	-0.46124	0.595201	-0.0922	NA	28.69	33.07	29.92	-0.08	0.56	-0.49246		0.126859	-0.38433	0.044388	0.02494	NA	24.80	0.95	18.10	-0.14	0.34
E5RI99	60S ribosomal protein L30 (Fragment)	RPL30	2.00	-0.4835		-0.23526	0.552126	-0.27253	0.635784	NA	37.66	43.10	26.00	0.04	0.85	0.241918		-0.38424	0.297651	-0.61864	0.535783	NA	32.81	53.74	8.84	0.01	0.64
F5H2F4	C-1-tetrahydrofolate synthase, cytoplasmic	MTHFD1	1.60	0.050864		0.334017	-0.03563	-0.06264	-0.02739	NA	18.02	1.73	7.16	0.05	0.45	0.012871		0.529327	0.316011	-0.15046	-0.47742	NA	10.44	15.96	25.62	0.05	0.87
Q7Z3X3	Putative uncharacterized protein DKFZp686E12166	DKFZp686E12166	4.60	1.304062		0.816978	0.502931	1.207206	1.026676	NA	15.33	8.84	21.55	0.97	0.03	1.155931		0.459639	0.47751	1.451534	0.974513	NA	0.88	23.17	26.48	0.90	0.06
Q7Z5A3	Small nuclear ribonucleoprotein Sm D1	SNRPD1	1.00	-0.97676		0.817129	1.164868	-1.37105	-1.3852	NA	16.96	0.69	93.29	-0.35	0.60	-0.84122		0.54814	0.731302	-0.80875	-0.85055	NA	8.97	2.05	64.63	-0.24	0.56
Q8TAS0	ATP synthase subunit gamma (Fragment)	NA	8.40	-0.52595		-1.60929	-1.33384	-0.7796	-1.11927	NA	13.46	16.57	31.59	-1.07	0.07	-0.4903		-1.517	-1.49805	-0.53447	-0.47891	NA	0.93	2.72	34.91	-0.90	0.13
Q96IR1	RPS4X protein (Fragment)	RPS4X	1.80	0		0.068078	-0.027	0.033644	0.645484	NA	4.66	29.55	15.01	0.14	0.39	0.326653		0.337383	0.014563	0.23776	0.105951	NA	15.76	6.46	6.01	0.20	0.05
U3KQK0	Histone H2B	HIST1H2BN	19.20	-2.80873		-1.71148	-2.28355	-1.01383	-0.83007	NA	27.68	8.99	64.27	-1.73	0.07	-1.88304		-1.12827	-1.45937	-1.27654	-1.23238	NA	16.16	2.16	22.65	-1.40	0.02
O15260	Surfeit locus protein 4	SURF4	3.00	0.520623		0.65051	0.510353	-0.1026	0.973543	NA	6.86	50.43	2.26	0.51	0.01	0.621456		0.163472	0.087626	0.064499	0.284955	NA	3.72	10.78	19.72	0.24	0.19
P60866	40S ribosomal protein S20	RPS20	2.60	0.359965		0.648144	0.255992	0.841701	1.048022	NA	19.10	10.10	22.77	0.63	0.08	0.962597		0.741564	0.703988	0.800753	0.23125	NA	1.84	27.56	14.67	0.69	0.03
Q9H0U4	Ras-related protein Rab-1B	RAB1B	6.80	-1.39232		-0.18197	-0.75087	-0.29225	0.036975	NA	27.53	16.07	40.41	-0.52	0.22	-0.34101		-0.81923	-0.67732	-1.10929	-0.44209	NA	6.95	32.13	16.72	-0.68	0.05
A8MU27	Small ubiquitin-related modifier 3	SUMO3	1.00	-0.4263		0.268414	-0.41481	-0.72983	-0.60811	NA	32.87	5.96	22.61	-0.38	0.15	-0.63354		-0.41024	-0.00025	-0.64362	-1.11551	NA	19.96	22.92	24.25	-0.56	0.10
P27348	14-3-3 protein theta	YWHAQ	8.80		0.593291	1.080665	-0.39319	-0.33958	-0.99802	NA	66.55	31.72	40.03	-0.01	0.84		0.262594	0.993787	-0.0791	0.073567	-0.17833	NA	50.29	12.31	20.68	0.21	0.27
A0A024R5Z9	Pyruvate kinase	PKM2	64.80		-2.15032	-1.56313	-1.58272	-1.62802	-1.86826	NA	0.96	11.75	19.67	-1.76	0.01		-2.58518	-1.72629	-1.86266	-1.65465	-1.9759	NA	6.68	15.68	28.19	-1.96	0.02
B2R4M6	Protein S100	NA	2.20		-0.53341	-1.98707	-1.73938	-2.31433	-2.80467	NA	12.11	23.80	72.32	-1.88	0.11		0.153897	-1.67691	-1.12734	-3.03707	-2.4691	NA	26.61	27.48	91.20	-1.63	0.25
B4DSW9	cDNA FLJ59415, highly similar to Beta-catenin	NA	4.60		0.313399	0.286564	-0.40955	0.165624	0.361408	NA	33.47	9.58	12.06	0.14	0.28		1.383824	0.443167	-0.80332	0.010302	-0.11134	NA	57.56	5.96	62.66	0.18	0.52
D9IWP9	Beta-2-glycoprotein I (Fragment)	NA	1.20		1.300894	1.435892	1.161807	1.952719	2.087996	NA	13.39	6.63	30.68	1.59	0.02		1.318884	0.78803	0.89223	1.759758	1.731517	NA	5.10	1.38	30.72	1.30	0.04
Q53SS8	Epididymis secretory protein Li 85	PCBP1	4.60		0.052013	0.115334	0.055726	0.412555	0.125791	NA	2.92	14.01	8.56	0.15	0.18		0.618864	0.211907	0.496774	0.135303	-0.14267	NA	13.92	13.58	20.95	0.26	0.22
Q59GL1	Synaptotagmin binding, cytoplasmic RNA interacting protein variant (Fragment)	NA	3.60		-0.44341	-0.17747	-0.15207	-1.18729	0.199027	NA	1.24	63.16	9.82	-0.35	0.07		-0.38545	-0.09735	-0.04798	-0.20973	0.230703	NA	2.42	21.42	14.39	-0.10	0.34
Q59GY2	Ribosomal protein L4 variant (Fragment)	NA	1.80		0.164177	1.452257	0.544735	1.488051	0.977209	NA	43.07	24.78	35.52	0.93	0.13		0.353704	1.118375	0.855046	0.605834	0.183975	NA	12.87	20.53	25.92	0.62	0.11
Q6ZVI6	NADH-cytochrome b5 reductase	NA	3.80		-0.39768	-0.4247	-0.5998	-0.60488	-0.58949	NA	8.57	0.75	6.96	-0.52	0.01		-0.35328	-0.57991	-0.52068	-0.5944	-0.55803	NA	2.90	1.78	8.63	-0.52	0.02
V9HW75	Epididymis secretory protein Li 109	NUCB2	1.80		0.797996	-0.03216	-0.13692	-0.53962	0.862964	NA	5.13	63.80	30.54	0.19	0.38		0.436473	0.031786	0.3152	-0.42938	0.783874	NA	13.85	56.19	8.89	0.23	0.09
V9HWH7	Epididymis secretory sperm binding protein Li 70p	ATIC	4.40		2.708129	1.393037	1.133248	0.29977	0.39178	NA	12.70	4.51	81.32	1.19	0.17		2.07853	1.146196	0.932189	0.276515	0.656556	NA	10.47	18.52	57.80	1.02	0.13
P02649	Apolipoprotein E	APOE	2.40		0.463017	-0.40071	-0.26982	-0.76788	-0.13068	NA	6.41	30.73	36.02	-0.22	0.74		0.988607	1.12512	-0.52104	-0.22391	0.423736	NA	72.94	31.22	29.63	0.36	0.23
Q05682	Caldesmon	CALD1	3.00		1.915068	1.028865	0.915036	1.723032	0.822859	NA	5.58	42.74	33.53	1.28	0.04		2.14645	2.897089	2.251019	2.436027	1.435657	NA	31.15	47.16	22.43	2.23	0.01
Q9P2E9	Ribosome-binding protein 1	RRBP1	4.20		1.200643	1.093267	0.114619	-0.03559	-0.05444	NA	46.21	0.92	40.90	0.46	0.24		1.010976	1.552115	0.342922	0.023993	-0.12973	NA	56.02	7.53	37.32	0.56	0.21
E9PL57	Protein NEDD8-MDP1 (Fragment)	NEDD8-MDP1	1.40		-0.05317	0.178294	0.215469	0.467069	-0.66345	NA	1.82	52.74	9.18	0.03	0.88		0.413329	0.433643	0.952282	0.493116	0.27985	NA	25.15	10.43	13.02	0.51	0.04
O14561	Acyl carrier protein, mitochondrial	NDUFAB1	3.00	-0.76292	-1.04238	-1.43505	-1.79593			13.65	17.60	NA	34.04	-1.26	0.18	-1.40921	-0.66051	-1.7406	-2.27037			35.89	25.68	NA	46.90	-1.52	0.20
O43181	NADH dehydrogenase [ubiquinone] iron-sulfur protein 4, mitochondrial	NDUFS4	2.75	0.048772	0.893158	-1.67717	-1.17183			40.24	24.52	NA	82.77	-0.48	0.70	-0.30672	0.756576	-0.99369	-0.65251			49.88	16.64	NA	52.88	-0.30	0.67
O43681	ATPase ASNA1	ASNA1	3.50	-0.59088	0.424215	-0.18408	-1.35055			47.80	54.25	NA	31.64	-0.43	0.43	-0.59133	-0.39556	0.014603	-0.4828			9.58	24.14	NA	13.55	-0.36	0.22
O95169	NADH dehydrogenase [ubiquinone] 1 beta subcomplex subunit 8, mitochondrial	NDUFB8	2.50	-0.34405	-0.64326	-0.88564	-0.82552			14.61	2.95	NA	18.01	-0.67	0.17	-0.52835	-0.33732	-0.86597	-0.65768			9.35	10.19	NA	16.03	-0.60	0.17
P13645	Keratin, type I cytoskeletal 10	KRT10	7.25	1.740799	1.755394	1.072748	0.375217			0.72	33.54	NA	46.37	1.24	0.25	1.111899	1.732706	0.996453	0.323492			29.97	32.40	NA	36.26	1.04	0.22
P14927	Cytochrome b-c1 complex subunit 7	UQCRB	3.75	-0.10046	-0.23715	-1.11902	-1.37564			6.69	12.54	NA	50.36	-0.71	0.41	-0.30917	-0.31057	-0.67206	-1.38696			0.07	34.34	NA	32.53	-0.67	0.31
P51970	NADH dehydrogenase [ubiquinone] 1 alpha subcomplex subunit 8	NDUFA8	4.50	-0.89835	-0.47235	-1.42008	-1.38043			20.73	1.94	NA	35.06	-1.04	0.21	-0.86059	-0.83466	-1.31713	-1.20666			1.27	5.41	NA	20.12	-1.05	0.12
P56134	ATP synthase subunit f, mitochondrial	ATP5J2	3.50	-0.89469	-0.60985	-0.90537	-1.21402			13.92	15.07	NA	14.95	-0.91	0.11	-0.7835	-0.353	-1.36782	-1.87279			20.95	24.50	NA	49.14	-1.09	0.29
Q15907	Ras-related protein Rab-11B	RAB11B	6.50	0.185912	0.111031	0.180167	0.198569			3.67	0.90	NA	1.98	0.17	0.08	0.155439	0.237206	0.011092	0.058116			4.01	2.30	NA	7.94	0.12	0.39
Q6ZS82	Regulator of G-protein signaling 9-binding protein	RGS9BP	3.00	0.001181	-1.8708	-1.08882	-0.74026			80.73	17.00	NA	12.40	-0.92	0.01	0.195026	-1.18326	-1.10522	-1.00886			62.85	4.72	NA	34.61	-0.78	0.22
A0A024R5K6	NADH dehydrogenase [ubiquinone] 1 subunit C2	NDUFC2	2.00	-0.56502	-0.73254	-0.90934	-0.09746			8.20	38.77	NA	9.75	-0.58	0.08	-0.66481	-0.53899	-0.99874	-0.56628			6.16	21.04	NA	8.12	-0.69	0.08
A0A024R7I7	H.sapiens ras-related Hrab3A protein	RAB3A	5.00	-0.69666	-0.58316	-0.74086	-1.47473			5.56	35.21	NA	20.58	-0.87	0.17	-1.21155	-0.24251	-0.85953	-1.77541			45.79	43.44	NA	28.94	-1.02	0.18
A0A024R9X3	Cyclic nucleotide gated channel alpha 1, isoform CRA_a	CNGA1	7.00	-2.63117	-0.77447	-1.38505	-1.73586			80.22	17.11	NA	6.23	-1.63	0.03	-0.35523	-1.15895	-1.50258	-1.69641			38.40	9.49	NA	42.49	-1.18	0.22
A0A0B4J2D5	Protein LOC102724023	LOC102724023	4.50	-0.46545	-0.23984	-0.94207	-0.45039			11.04	23.87	NA	15.97	-0.52	0.20	-0.64377	-0.621	-0.66202	-0.76594			1.12	5.09	NA	3.95	-0.67	0.04
B7ZB41	cDNA, FLJ79405, highly similar to Homo sapiens solute carrier family 25, member 24, transcript variant 1, n	NA	3.50	-0.3205	-0.76637	-0.24277	-0.2842			21.68	2.03	NA	12.85	-0.40	0.21	0.23001	-1.14572	-0.42783	-0.4881			62.74	2.95	NA	7.73	-0.46	0.00
C9JF17	Apolipoprotein D (Fragment)	APOD	1.75	-1.51578	0.11068	-1.46255	-1.54762			72.23	4.17	NA	48.01	-1.10	0.22	-1.99301	0.503291	-1.11232	-1.6332			98.84	25.26	NA	50.94	-1.06	0.18
D3DVQ1	Leucine zipper-EF-hand containing transmembrane protein 1, isoform CRA_a	LETM1	2.50	0.317472	0.805401	-0.17897	-0.45184			23.69	13.33	NA	42.33	0.12	0.83	-0.45563	1.179905	-0.62603	-0.28014			72.55	16.87	NA	48.24	-0.05	0.93
F1T0J3	Interphotoreceptor matrix proteoglycan 2	IMPG2	5.00	0.007265	0.065681	-1.46474	-1.31338			2.86	7.41	NA	64.62	-0.68	0.52	0.843339	0.234399	-1.45949	-1.05007			29.41	19.93	NA	78.69	-0.36	0.76
G3V0I5	NADH dehydrogenase (Ubiquinone) flavoprotein 1, 51kDa, isoform CRA_c	NDUFV1	9.25	-0.10236	-0.46578	-0.30134	-0.68793			17.72	18.84	NA	10.23	-0.39	0.17	-0.49863	-0.32485	-0.92193	-1.09896			8.51	8.67	NA	28.93	-0.71	0.25
J3KS22	L-xylulose reductase (Fragment)	DCXR	3.50	-1.83729	-1.64089	-1.46474	-0.36643			9.61	51.37	NA	43.72	-1.33	0.19	-0.88021	-1.07624	-1.67853	-0.57791			9.59	51.47	NA	2.49	-1.05	0.05
Q53GX6	Nucleobindin 1 variant (Fragment)	NA	3.75	-0.34505	-0.1946	0.01398	-0.00753			7.37	1.05	NA	13.25	-0.13	0.51	-0.11252	-0.0548	0.392016	0.249428			2.83	6.98	NA	19.76	0.12	0.66
Q68D64	Putative uncharacterized protein DKFZp686E23276 (Fragment)	DKFZp686E23276	2.50	0.4447	-0.19395	0.192744	0.454695			30.80	12.80	NA	8.28	0.22	0.26	0.916326	0.418944	0.737016	0.346697			24.14	19.01	NA	6.56	0.60	0.07
Q6N0B1	Succinyl-CoA ligase subunit beta (Fragment)	DKFZp686D0880	3.75	-0.19239	-0.11186	-0.26617	-0.41446			3.95	7.26	NA	9.15	-0.25	0.23	0	-0.01128	-0.62291	-0.60529			0.55	0.86	NA	29.39	-0.31	0.49
P38159	RNA-binding motif protein, X chromosome	RBMX	1.25	-1.03487	0.253034	0.100578	-0.8735			59.24	46.01	NA	2.65	-0.39	0.00	-1.02922	-0.25429	0	-1.3524			37.09	61.82	NA	3.29	-0.66	0.02
Q96AB3	Isochorismatase domain-containing protein 2, mitochondrial	ISOC2	3.00	-0.28194	0.669862	-0.84033	-1.007			45.03	8.16	NA	55.33	-0.36	0.63	0.303226	1.370932	-1.76275	-0.3058			50.07	65.90	NA	78.02	-0.10	0.93
A0A024R7Z5	Syndecan binding protein (Syntenin), isoform CRA_c	SDCBP	1.75	-0.38793	-0.17154	0.042055	-0.35122			10.59	19.16	NA	6.59	-0.22	0.18	-0.53373	0.718916	-0.12	0.262109			57.81	18.62	NA	6.90	0.08	0.08
A0A0C4DH75	MAGUK p55 subfamily member 2	MPP2	2.75	-0.00473	-0.48584	-0.03	-0.64484			23.36	29.69	NA	3.90	-0.29	0.10	-0.36367	-0.78408	0.184779	-0.32212			20.46	24.59	NA	24.84	-0.32	0.42
C9JRZ6	MIC	CHCHD3	1.50	0.586602	0.663644	0.043599	-0.49837			3.78	26.26	NA	39.49	0.20	0.72	0.341256	0.546931	-0.64371	0.253667			10.06	42.62	NA	27.77	0.12	0.76
E7EWZ0	NADH dehydrogenase [ubiquinone] 1 beta subcomplex subunit 9	NDUFB9	2.50	-0.31952	-0.7376	-1.34679	-0.97452			20.35	18.15	NA	30.64	-0.84	0.23	-0.65305	-0.61221	-1.51122	-1.29803			2.00	10.43	NA	36.79	-1.02	0.23
G3V2S9	SRA stem-loop-interacting RNA-binding protein, mitochondrial	SLIRP	1.50	0.727842	0.173464	0.701601	0.111207			26.84	28.54	NA	2.00	0.43	0.03	-0.02319	0.559994	1.101727	0.644209			28.20	22.24	NA	28.68	0.57	0.31
H3BS10	Beta-hexosaminidase	HEXA	1.50	1.236914	0.998386	0.709241	0.206816			11.66	24.38	NA	30.99	0.79	0.25	0.470702	1.052471	1.049989	0.398158			28.13	31.42	NA	1.48	0.74	0.02
P07814	Bifunctional glutamate/proline--tRNA ligase	EPRS	3.00	-0.08681	1.409284	-0.03433		1.19046		67.39	NA	NA	39.16	0.62	0.23	-0.14462	1.361615	0.17786		0.012559		67.78	NA	NA	30.18	0.35	0.27
P41208	Centrin-2	CETN2	1.25	-0.96596	-0.40696	-0.42826		0.211498		27.06	NA	NA	32.76	-0.40	0.38	0.234487	-0.09189	0.537021		0.438166		15.93	NA	NA	15.93	0.28	0.13
P60880	Synaptosomal-associated protein 25	SNAP25	4.00	-0.27477	-0.71219	-0.75466		-0.80844		21.28	NA	NA	12.69	-0.64	0.02	-0.12705	-0.61727	-0.40654		-0.76382		23.80	NA	NA	14.82	-0.48	0.05
Q16352	Alpha-internexin	INA	4.75	-1.15334	-1.89885	-0.04441		-0.89521		35.75	NA	NA	50.48	-1.00	0.20	-1.06728	-0.98052	-0.57236		-0.79577		4.25	NA	NA	15.57	-0.85	0.03
A8K454	cDNA FLJ78187, highly similar to Homo sapiens calmegin, mRNA	NA	1.25	0.766426	0.795373	0.640173		1.707878		1.42	NA	NA	43.26	0.98	0.09	1.096201	0.507327	0.969438		0.919969		28.47	NA	NA	4.81	0.87	0.00
B2R659	cDNA, FLJ92803, highly similar to Homo sapiens hydroxysteroid (17-beta) dehydrogenase 4 (HSD17B4), mR	NA	8.50	0.387313	0.80627	0.17125		0.38488		20.39	NA	NA	15.27	0.44	0.09	0.370025	0.962496	-0.07986		0.695772		28.64	NA	NA	27.88	0.49	0.23
B2RBD5	cDNA, FLJ95457, highly similar to Homo sapiens tubulin, beta, 4 (TUBB4), mRNA	NA	37.50	-0.51325	-0.74426	-1.6968		0.588895		11.30	NA	NA	75.08	-0.59	0.47	-1.00044	-1.3043	-0.71545		-0.2021		14.84	NA	NA	32.72	-0.81	0.13
G3XHN4	Cell adhesion molecule 2 isoform 4	CADM2	2.25	-0.50597	-1.40344	-1.05594		-0.99783		42.62	NA	NA	6.04	-0.99	0.00	-0.77111	-1.58651	-1.24338		-0.47837		38.93	NA	NA	30.18	-1.02	0.06
L7N2F9	Uncharacterized protein (Fragment)	NA	3.00	-0.86678	0.324356	-0.80563		-1.38888		55.28	NA	NA	42.43	-0.68	0.13	-0.84633	0.982263	-1.19855		-0.78825		79.28	NA	NA	58.37	-0.46	0.23
P11117	Lysosomal acid phosphatase	ACP2	1.75	0.847428	0.075443	-0.07955		0.914703		36.96	NA	NA	33.08	0.44	0.27	0.394236	-0.02849	-0.06761		1.222422		20.57	NA	NA	50.52	0.38	0.38
Q9HDC9	Adipocyte plasma membrane-associated protein	APMAP	3.25	0.373855	0.509362	0.659431		-0.1583		6.64	NA	NA	27.29	0.35	0.33	0.884158	0.728922	0.492387		0.176138		7.60	NA	NA	21.79	0.57	0.11
F8VZY9	Keratin, type I cytoskeletal 18	KRT18	6.25	1.55097	1.488204	1.219056		0.698563		3.08	NA	NA	27.47	1.24	0.04	1.284368	1.255689	1.182774		1.285281		1.41	NA	NA	3.79	1.25	0.00
P01034	Cystatin-C	CST3	1.00	0.412673	0.779647	0.193672			0.386492	17.89	NA	NA	14.40	0.44	0.08	0.370025	1.141465	0.512395			0.730421	36.93	NA	NA	10.34	0.69	0.01
P53621	Coatomer subunit alpha	COPA	2.25	1.040852	1.670399	1.419834			0.864051	30.38	NA	NA	20.18	1.25	0.02	0.58072	0.932214	1.434674			0.757775	17.14	NA	NA	28.61	0.93	0.05
A0A024R6S1	DnaJ (Hsp40) homolog, subfamily A, member 2, isoform CRA_a	DNAJA2	2.00	0.073114	-0.16557	0.26555			0.068316	11.67	NA	NA	10.92	0.06	0.40	0.066377	0.741761	0.487342			0.158305	32.51	NA	NA	11.94	0.36	0.07
A0A024RDR0	High-mobility group box 1, isoform CRA_a	HMGB1	4.00	-0.98375	-0.79309	-0.97087			-0.73268	9.33	NA	NA	8.45	-0.87	0.01	-0.53468	-0.71336	-1.28717			-1.16843	8.75	NA	NA	25.88	-0.93	0.04
A0A0A0MTS2	Glucose-6-phosphate isomerase (Fragment)	GPI	16.00	-0.36316	-0.17818	-0.17059			-0.02179	9.05	NA	NA	8.65	-0.18	0.17	-0.18939	-0.08369	-0.46822			-0.44325	5.18	NA	NA	13.27	-0.30	0.08
G3V4I5	GSK3-beta interaction protein (Fragment)	GSKIP	1.00	-1.70049	-1.86319	-1.29644			-1.70916	7.97	NA	NA	18.85	-1.64	0.01	-1.23045	-1.94445	-1.30707			-1.46259	34.30	NA	NA	8.43	-1.49	0.00
I3L3B4	Uncharacterized protein (Fragment)	NA	1.00	1.413265	0.328897	0.543646			0.411572	50.78	NA	NA	21.03	0.67	0.05	1.490645	0.279213	1.133449			0.622751	56.12	NA	NA	17.63	0.88	0.03
Q6IBA2	PC4 protein	PC4	2.00	-0.0843	-0.55751	0.032239			-0.99464	22.99	NA	NA	33.64	-0.40	0.29	0.104174	-0.78483	0.595971			-1.34535	42.24	NA	NA	61.87	-0.36	0.58
Q9H7G6	cDNA: FLJ20897 fis, clone ADKA03573	NA	4.25	2.084855	0.465356	0.953401			0.27376	71.97	NA	NA	40.41	0.94	0.11	1.72139	0.81348	0.672748			0.417978	43.09	NA	NA	34.45	0.91	0.09
Q9Y3E8	CGI-150 protein	NA	3.50	0.930644	0.57289	0.011878			-0.05605	17.45	NA	NA	33.67	0.36	0.46	0.974467	0.466287	-0.21667			0.158689	24.65	NA	NA	34.15	0.35	0.50
Q08426	Peroxisomal bifunctional enzyme	EHHADH	3.25	0.037308	0.030243	-1.25095			-0.30287	0.35	NA	NA	40.73	-0.37	0.32	0.065958	-0.94785	-0.60163			-0.38394	47.74	NA	NA	9.12	-0.47	0.02
A0A024R6C0	Niemann-Pick disease, type C2, isoform CRA_a	NPC2	5.50	1.415775	0.815899	1.439317			0.757148	28.99	NA	NA	23.17	1.11	0.03	1.186483	0.845718	1.450779			0.788096	16.62	NA	NA	23.81	1.07	0.03
H3BNQ7	4-aminobutyrate aminotransferase, mitochondrial	ABAT	2.75	-0.62265	0.094414	-0.26076			-1.34767	34.44	NA	NA	37.71	-0.53	0.23	-0.8578	0.03225	-0.16525			-1.75171	42.29	NA	NA	48.22	-0.69	0.26

UniProt	Protein name	Gene name	Peptide mean	08/017 hESC-RPE to hRPE												08/023 hESC-RPE to hRPE											
				Analysis						Technical replicate CV			Biological	Fold change (mean)	Unadjusted p-value	Analysis						Technical replicate CV			Biological	Fold change (mean)	Unadjusted p-value
				1.1	1.2	2.1	2.2	3.1	3.2	1	2	3	CV		(t-test)	1.1	1.2	2.1	2.2	3.1	3.2	1	2	3	CV		(t-test)
J3KRC4	5'(3')-deoxyribonucleotidase, cytosolic type	NT5C	2.00	-0.82887	-1.40898	-2.31084			-1.83965	28.06	NA	NA	43.54	-1.60	0.04	-1.71464	-1.39933	-3.38954			-0.92055	15.39	NA	NA	67.46	-1.86	0.12
S4R371	Fatty acid-binding protein, heart (Fragment)	FABP3	2.25	2.203669	1.339137	1.878319			1.511072	41.15	NA	NA	13.35	1.73	0.00	1.096201	-1.08744	1.553409			1.372066	90.39	NA	NA	37.75	0.73	0.18
O15540	Fatty acid-binding protein, brain	FABP7	2.00	-0.58674	-1.20179		-0.44572	-0.46575		29.70	NA	NA	15.44	-0.67	0.05	-0.87739	-1.37398		-0.63751	-1.11869		24.10	NA	NA	19.88	-1.00	0.03
F5H157	Ras-related protein Rab-35 (Fragment)	RAB35	3.75	-1.75326	-1.22467		0.11535	0.360698		25.62	NA	NA	53.28	-0.63	0.62	-2.67772	-2.47572		-1.34021	-0.1404		9.88	NA	NA	77.27	-1.66	0.19
A0A024R8P8	Ribosomal protein L38, isoform CRA_a	RPL38	1.25	-0.47663	-0.16791		0.100181	0.113342		15.07	NA	NA	15.96	-0.11	0.82	-0.29646	-0.00221		0.424344	-0.34003		14.37	NA	NA	28.73	-0.05	0.93
D3GKD8	A-gamma globin Osilo variant	HBG1	16.25	-0.57027	-1.52929		-1.18616	-2.14649		45.35	NA	NA	37.76	-1.36	0.05	-1.14365	-1.93768		-1.37841	-1.54862		37.96	NA	NA	6.02	-1.50	0.00
A0A024RDG1	Vesicle docking protein p115, isoform CRA_a	VDP	1.75	0.220958	0.730039		0.685636		1.364595	24.70	NA	NA	33.38	0.75	0.09	-0.1541	0.407692		0.221779		1.527712	27.19	NA	NA	58.53	0.50	0.30
Q05DH1	Proteasome subunit alpha type (Fragment)	PSMA7	2.00	0.186777	0.186701		-0.08594		-0.0024	0.00	NA	NA	9.84	0.07	0.72	-0.89318	0.072204		0.195135		-0.16099	45.63	NA	NA	19.09	-0.20	0.55
Q6FI54	RAB5B protein	RAB5B	1.50	0.454502	-0.17475		-0.6436		-0.73692	30.36	NA	NA	37.24	-0.28	0.28	-0.43623	-0.27707		0.181626		0.124924	7.79	NA	NA	19.14	-0.10	0.93
Q6IBT1	Proteasome subunit beta type	PSMB7	3.25	-0.06812	0.199817		0.392993		0.281339	13.09	NA	NA	11.09	0.20	0.12	0.506619	0.494715		0.566422		0.192431	0.58	NA	NA	13.32	0.44	0.07
O14531	Dihydropyrimidinase-related protein 4	DPYSL4	1.50	-0.554	-1.16046		-0.27014		-0.76354	29.29	NA	NA	22.15	-0.69	0.07	-0.24232	-1.62813		-0.09761		-1.75532	63.14	NA	NA	52.83	-0.93	0.19
P25189	Myelin protein P0	MPZ	3.00	-0.6887	-1.00038			-2.52587	-1.89535	15.22	NA	30.42	61.34	-1.53	0.27	-0.70104	-1.13359			-2.66117	-1.93767	21.04	NA	34.74	61.86	-1.61	0.26
P36871	Phosphoglucomutase-1	PGM1	5.50	0.178973	-0.05022			0.298634	-0.11162	11.21	NA	19.97	1.92	0.08	0.12	0.286436	0.096933			-0.02461	0.093966	9.27	NA	5.81	7.78	0.11	0.39
P51991	Heterogeneous nuclear ribonucleoprotein A3	HNRNPA3	5.25	-0.96942	-1.24703			-0.78008	-1.90749	13.56	NA	52.61	6.60	-1.23	0.06	-0.35888	-1.05686			-0.38172	-0.97759	33.56	NA	28.80	0.83	-0.69	0.01
Q15717	ELAV-like protein 1	ELAVL1	2.50	0.409268	-0.36577			0.358192	0.326444	37.10	NA	1.56	13.16	0.18	0.46	0.409329	0.030094			-0.10929	-0.15382	18.48	NA	2.18	17.72	0.04	0.84
Q9NP72	Ras-related protein Rab-18	RAB18	2.75	-0.46926	-0.14821			-0.32795	-1.07901	15.67	NA	36.00	17.33	-0.51	0.24	-0.51608	-0.01217			-0.31976	-0.49904	24.45	NA	8.78	8.05	-0.34	0.14
Q9UNZ2	NSFL1 cofactor p47	NSFL1C	9.50	0.714109	1.293634			1.21656	1.434498	28.03	NA	10.66	14.50	1.16	0.09	0.953235	1.465967			0.800515	1.123844	24.87	NA	15.78	12.76	1.09	0.07
A0A024R983	TROVE domain family, member 2, isoform CRA_b	TROVE2	2.00	0.728318	0.399531			0.687582	-0.28468	16.05	NA	45.93	14.23	0.38	0.28	0.515537	0.387619			0.912957	0.390476	6.27	NA	25.33	10.87	0.55	0.11
A0A024RA61	Heterogeneous nuclear ribonucleoprotein A2/B1, isoform CRA_c	HNRPA2B1	13.25	-1.60458	-1.82906			-0.84714	-1.04741	10.98	NA	9.80	36.81	-1.33	0.18	-0.62135	-0.80934			-1.04986	-1.17325	9.20	NA	6.04	19.38	-0.91	0.14
A0A024RCM3	HCG2005638, isoform CRA_a	hCG_2005638	4.50	-0.11937	-0.09716			-0.33693	-0.25351	1.09	NA	4.09	9.12	-0.20	0.28	-0.57003	-0.11438			-0.62214	-0.58635	22.15	NA	1.75	13.67	-0.47	0.17
A8K3Q7	Annexin	NA	28.25	-0.1586	-0.69672			-0.74545	-0.74305	26.07	NA	0.12	16.66	-0.59	0.17	-0.44814	-0.66574			-0.76452	-0.71083	10.65	NA	2.63	9.03	-0.65	0.09
A8K7N0	cDNA FLJ75556, highly similar to Homo sapiens ribosomal protein L14, mRNA	NA	1.50	-0.62265	-1.92961			0.293625	-0.11055	60.01	NA	19.68	57.23	-0.59	0.55	-1.13978	-0.66016			0.31874	0.032077	23.29	NA	14.00	49.85	-0.36	0.62
J3KR44	Ubiquitin thioesterase OTUB1	OTUB1	1.25	0.867419	-0.17969			-0.01963	-0.3013	49.18	NA	13.76	28.54	0.09	0.78	-0.29136	0.233837			-1.27054	0.323961	25.46	NA	71.06	12.63	-0.25	0.46
Q53SW3	Putative uncharacterized protein DPYSL5 (Fragment)	DPYSL5	2.25	0.269777	-0.64208			-0.5667	-0.39316	43.26	NA	8.50	17.65	-0.33	0.26	0.289307	-0.58518			-0.99155	-0.33343	41.60	NA	31.71	26.29	-0.41	0.36
Q53XX5	Cold inducible RNA binding protein	CIRBP	3.00	-1.31192	-1.28307			-1.32655	-1.8781	1.41	NA	26.71	13.62	-1.45	0.07	-0.97007	-1.29453			-1.64492	-1.63473	15.84	NA	0.50	25.05	-1.39	0.12
Q59EH3	Acid phosphatase 1 isoform c variant (Fragment)	NA	1.25	-0.73808	0.104513			-0.21607	-0.29383	40.16	NA	3.81	0.08	-0.29	0.07	-1.10638	0.34575			0.420661	-0.20103	65.72	NA	30.01	16.97	-0.14	0.68
Q5U071	High-mobility group box 2	NA	4.25	-0.95256	-1.12816			-0.49264	-1.43859	8.60	NA	44.77	7.26	-1.00	0.02	-0.77448	-1.0781			-0.58042	-1.23037	14.83	NA	31.33	2.41	-0.92	0.01
Q6FHJ5	SCAMP3 protein (Fragment)	SCAMP3	1.25	0.242748	-0.18378			-1.23453	0.434341	20.75	NA	73.75	10.58	-0.19	0.55	0.550518	0.272114			-0.46575	0.14926	13.60	NA	29.70	26.34	0.13	0.73
V9HWG9	Epididymis secretory protein Li 21	GSTO1	1.00	0.506559	0.684464			0.817633	1.078658	8.71	NA	12.76	17.35	0.77	0.14	-0.21384	1.10074			0.335389	0.768623	60.31	NA	21.08	0.98	0.50	0.07
X5DP00	HCG15924, isoform CRA_a (Fragment)	SNRPN	2.00	-0.20278	-0.25697			0.731939	1.586799	2.66	NA	40.71	65.69	0.46	0.62	-0.23586	-0.43911			0.279244	0.698454	9.95	NA	20.40	39.95	0.08	0.88
O60664	Perilipin-3	PLIN3	2.00	0.995465	1.336283			1.498874	0.824057	16.63	NA	32.48	1.21	1.16	0.00	1.499119	1.443458			1.606037	1.159648	2.73	NA	21.71	3.50	1.43	0.02
O75964	ATP synthase subunit g, mitochondrial	ATP5L	2.75	-0.39051		-0.821	-0.39735	-0.34259		NA	20.62	NA	9.02	-0.49	0.03	-0.42119		-0.43158	-0.21367	-0.50865		NA	10.66	NA	6.61	-0.39	0.02
P49327	Fatty acid synthase	FASN	2.50	-0.09079		1.258588	0.003559	1.696925		NA	57.91	NA	59.90	0.72	0.29	-0.16315		1.402871	-0.68907	1.022059		NA	87.68	NA	38.01	0.39	0.36
Q16658	Fascin	FSCN1	2.75	0.970167		-0.15663	0.064324	-0.22225		NA	10.81	NA	47.99	0.16	0.59	0.165243		0.308862	-0.20056	0.502488		NA	24.71	NA	16.09	0.19	0.22
Q6YN16	Hydroxysteroid dehydrogenase-like protein 2	HSDL2	4.50	1.10183		0.194599	0.003559	0.702004		NA	9.35	NA	33.21	0.50	0.16	1.422714		0.277471	0.175829	1.636285		NA	4.98	NA	43.88	0.88	0.13
Q53GD1	Guanine nucleotide-binding protein subunit gamma (Fragment)	NA	1.00	1.013519		1.321027	0.903948	2.089793		NA	20.30	NA	44.22	1.33	0.05	1.245434		1.838433	0.859642	1.478498		NA	46.21	NA	8.35	1.36	0.00
Q549N0	Cofilin 2 (Muscle), isoform CRA_a	CFL2	4.75	1.088192		0.877237	-0.12854	0.838421		NA	47.39	NA	21.31	0.67	0.07	0.417719		0.553151	0.174488	0.302736		NA	18.45	NA	4.01	0.36	0.01
Q5JR95	40S ribosomal protein S8	RPS8	2.50	0.738264		0.419536	0.710981	0.440533		NA	14.24	NA	10.39	0.58	0.02	0.95719		0.295728	0.476604	0.678951		NA	8.85	NA	19.56	0.60	0.05
Q5RLJ0	CLE	NA	2.75	-1.20595		0.592101	0.358659	-0.02773		NA	11.42	NA	51.50	-0.07	0.66	-0.68404		0.230576	-0.46528	0.648407		NA	33.46	NA	45.88	-0.07	0.91
Q6QN92	Mitochondrial glycine cleavage system H-protein (Fragment)	NA	2.00	-0.11084		0.567272	0.509428	0.138323		NA	2.83	NA	23.13	0.28	0.42	0.036991		0.269725	0.461289	1.052885		NA	9.38	NA	37.25	0.46	0.25
V9HWA2	Aldo-keto reductase family 7, member A2 (Aflatoxin aldehyde reductase)	AKR7A2	1.50	1.64019		-0.26521	-0.40815	0.672137		NA	7.00	NA	64.36	0.41	0.37	1.422795		-0.55627	-0.9619	0.629551		NA	19.75	NA	64.88	0.13	0.57
A0A024R7V6	RAB2, member RAS oncogene family, isoform CRA_a	RAB2	2.75	-0.20641		0.386056	0.142831		-0.20188	NA	11.89	NA	19.87	0.03	0.79	-0.02052		0.296964	0.044478		0.024098	NA	12.34	NA	7.26	0.09	0.42
E9PBS1	Multifunctional protein ADE2 (Fragment)	PAICS	4.25	0.747087		0.157532	1.219777		0.878806	NA	49.83	NA	4.75	0.75	0.01	0.812978		0.160984	2.05854		1.104459	NA	81.57	NA	20.31	1.03	0.01
Q53GE2	Capping protein (Actin filament) muscle Z-line, alpha 2 variant (Fragment)	NA	2.50	-0.10279		0.738767	0.340868		0.124625	NA	19.38	NA	23.69	0.28	0.42	0.322103		0.617243	0.883503		0.811951	NA	13.01	NA	17.55	0.66	0.06
Q7Z759	CCT8 protein	CCT8	7.25	0.307963		0.374653	0.487219		0.294305	NA	5.51	NA	5.33	0.37	0.02	0.158584		0.87242	0.350067		0.636864	NA	25.33	NA	17.95	0.50	0.09
Q9UK22	F-box only protein 2	FBXO2	1.25	1.623996		0.96363	0.250462		0.07571	NA	34.26	NA	55.43	0.73	0.23	1.447108		0.011594	-0.52285		-1.09205	NA	25.90	NA	89.54	-0.04	0.97
E7EMK3	Flotillin-2	FLOT2	1.50	0.176189		-0.23152	0.104356		-0.50343	NA	16.39	NA	22.93	-0.11	0.58	-1.28334		-0.75334	0.177615		-0.955	NA	44.11	NA	39.60	-0.70	0.10
Q5TCU3	Tropomyosin beta chain	TPM2	6.75	0.54079		0.980386	-0.08622		0.045217	NA	50.02	NA	18.63	0.37	0.15	1.510547		1.567651	1.230194		1.178877	NA	16.46	NA	11.51	1.37	0.01
Q6NSD4	Glutathione peroxidase	GPX1	1.50	1.187296		0.888735	0.492101		0.636057	NA	19.32	NA	21.85	0.80	0.04	1.347297		0.741261	0.593935		1.038553	NA	7.21	NA	23.12	0.93	0.04
P45974	Ubiquitin carboxyl-terminal hydrolase 5	USP5	3.00	-0.49427		0.324735		-0.31016	0.031455	NA	NA	16.67	28.57	-0.11	0.71	-0.468		0.877801		-0.5178	-0.06761	NA	NA	21.89	54.55	-0.04	0.93
A1A508	PRSS3 protein	PRSS3	3.50	1.176537		0.976485		1.365426	1.472682	NA	NA	5.25	15.47	1.25	0.01	1.107635		0.695584		1.311344	1.359105	NA	NA	2.34	21.65	1.12	0.03
Q53YE2	Syntaxin 3A	STX3A	3.25	-0.73513		-2.72019		-0.82617	-0.68011	NA	NA	7.15	57.33	-1.24	0.17	-1.08949		-2.14945		-1.10084	-1.50147	NA	NA	19.51	34.59	-1.46	0.04
Q59H49	Polypyrimidine tract-binding protein 1 isoform c variant (Fragment)	NA	2.75	1.036541		0.918068		0.673715	0.646026	NA	NA	1.36	13.01	0.82	0.02	1.474098		0.343778		0.729087	0.665628	NA	NA	3.11	41.77	0.80	0.13
Q5VU08	Adducin 3 (Gamma), isoform CRA_a	ADD3	1.25	0.290365		0.663788		-1.12286	-1.44435	NA	NA	15.69	55.85	-0.40	0.87	0.62629		-0.24278		-0.99814	-0.84016	NA	NA	7.74	53.34	-0.36	0.73
Q5VVD0	Ribosomal protein L11, isoform CRA_b	RPL11	1.50	0.39377		0.366863		0.845051	1.135344	NA	NA	14.18	26.18	0.69	0.10	0.596602		0.20028		0.226846	0.591123	NA	NA	17.76	13.63	0.40	0.07
Q9H3H0	Vacuolar ATP synthase subunit D homolog	VATD	1.75	-0.86141		-0.42862		-0.46517	0.392857	NA	NA	40.86	30.54	-0.34	0.20	-0.97136		-0.22729		-0.76066	0.636451	NA	NA	63.58	34.91	-0.33	0.27
Q9HBB3	60S ribosomal protein L6	NA	1.25	0.761558		1.442441		0.100361	1.024177	NA	NA	43.79	31.95	0.83	0.07	0.525466		1.76036		0.153078	0.859099	NA	NA	33.93	53.33	0.82	0.15
B3KTM6	Ribosomal protein L5, isoform CRA_b	RPL5	2.75	0.540925		-0.02837		0.515496	0.688651	NA	NA	8.48	22.35	0.43	0.21	0.536839		1.071221		0.363787	0.481307	NA	NA	5.76	25.18	0.61	0.08
B2RDN9	cDNA, FLJ96699, highly similar to Homo sapiens thyroid autoantigen 70kDa (Ku antigen) (G22P1), mRNA	NA	14.25	-0.71173			0.470573	-0.93635	-0.60059	NA	NA	16.38	52.55	-0.44	0.49	-0.37414			0.196457	-0.586	-0.51395	NA	NA	3.53	28.33	-0.32	0.39
B7Z5S9	cDNA FLJ61359, highly similar to Adapter-relatedprotein complex 2 alpha- 2 subunit	NA	3.25	-0.06564			-0.36507	-0.28798	-0.86491	NA	NA	27.91	17.14	-0.40	0.15	0.291278			-0.2646	-0.68259	-1.21091	NA	NA	25.61	40.52	-0.47	0.48
C9JV77	Alpha-2-HS-glycoprotein	AHSG	1.00	-0.70433			0.03709	-0.46055	-0.39706	NA	NA	3.11	26.55	-0.38	0.23	-0.42265			-0.08873	-0.21933	-0.04248	NA	NA	8.66	12.18	-0.19	0.18
Q5VY30	Retinol binding protein 4, plasma, isoform CRA_b	RBP4	2.50	2.538352			2.639081	1.836896	2.633531	NA	NA	38.08	12.15	2.41	0.00	2.48967			1.9235	2.212641	3.268719	NA	NA	49.57	30.35	2.47	0.01
Q6NWZ1	CKAP4 protein (Fragment)	CKAP4	11.50	0.458662			-0.22138	0.202294	0.473649	NA	NA	13.26	23.39	0.23	0.46	0.590066			0.212009	0.691809	0.780637	NA	NA	4.35	18.00	0.57	0.08
Q8N8J5	cDNA FLJ39374 fis, clone PEBLM2008576, highly similar to Homo sapiens TOLLIP protein	NA	1.00	-0.13874			0.660338	0.799355	0.898652	NA	NA	4.86	32.55	0.55	0.27	0.438241			0.757511	-0.20281	0.219346	NA	NA	20.54	24.89	0.30	0.21
P11171	Protein 4.1	EPB41	2.25	-0.1897			-0.13576	-0.22683	-0.63157	NA	NA	19.71	9.99	-0.30	0.11	-1.25168			-0.75696	-0.80477	-0.81095	NA	NA	0.30	17.78	-0.91	0.03
E7EPK1	Septin-7	SEPT7	2.50	-0.47581			-0.3375	-0.0905	-0.0987	NA	NA	0.40	13.58	-0.25	0.11	-0.87498			-0.19622	-0.58166	-0.01431	NA	NA	27.45	23.74	-0.42	0.16
Q92506	Estradiol 17-beta-dehydrogenase 8	HSD17B8	1.50		0.665441	-0.16137	-0.12537	0.510696		NA	1.76	NA	27.24	0.22	0.30		-1.40925	0.202701	0.398732	-0.00083		NA	9.59	NA	50.96	-0.20	0.56
Q9NUJ1	Mycophenolic acid acyl-glucuronide esterase, mitochondrial	ABHD10	3.75		-0.35943	-1.28736	-1.40745	-0.575		NA	5.88	NA	32.40	-0.91	0.13		-0.84809	-1.19044	-1.48245	0.020124		NA	14.26	NA	48.80	-0.88	0.21
C9JW74	Mesoderm-specific transcript homolog protein	MEST	1.75		0.776991	-0.92889	-0.35999	-0.19399		NA	27.53	NA	51.83	-0.18	0.97		1.372635	-0.3027	0.022783	0.597385		NA	15.89	NA	50.80	0.42	0.30
B4DEA8	cDNA FLJ56425, highly similar to Very-long-chain specific acyl-CoAdehydrogenase, mitochondrial (EC 1.3.9	NA	8.00		0.598064	0.286781	0.298625		0.848908	NA	0.58	NA	19.04	0.51	0.07		0.753573	0.445771	0.292097		0.483606	NA	7.52	NA	13.93	0.49	0.04
B7Z6N2	cDNA FLJ56154, highly similar to Gelsolin	NA	12.25		0.536315	0.308132	-0.11648		0.117607	NA	20.66	NA	17.61	0.21	0.22		0.554753	0.294284	0.16956		0.435499	NA	6.11	NA	11.09	0.36	0.05
H0YJ66	Dehydrogenase/reductase SDR family member 7 (Fragment)	DHRS7	1.00		-0.11988	-0.08432	-1.74555		0.463025	NA	73.48	NA	39.21	-0.37	0.68		-1.12203	-1.2706	-2.23668		-0.72563	NA	45.66	NA	31.73	-1.34	0.06
O14618	Copper chaperone for superoxide dismutase	CCS	1.00		0.207893	0.477605	0.681203		0.442268	NA	9.96	NA	12.90	0.45	0.06		1.415809	1.09854	0.920504		0.71967	NA	8.72	NA	24.50	1.04	0.04
P26012	Integrin beta-8	ITGB8	2.75		0.56944	-0.4347	1.024704		-0.2138	NA	66.00	NA	26.88	0.24	0.44		0.442323	-0.43877	0.742959		-0.28724	NA	54.88	NA	24.64	0.11	0.68
A0A024R7X6	Retinol dehydrogenase 10 (All-trans), isoform CRA_a	RDH10	1.50		-0.30964	0.107729	-0.48761		-1.95986	NA	28.77	NA	52.95	-0.66	0.29		-0.28779	-0.53558	0.066816		-1.14409	NA	29.10	NA	31.87	-0.48	0.20
H7C3T4	Peroxiredoxin-4 (Fragment)	PRDX4	5.50		0.266984	0.187866	0.595866		0.036562	NA	19.87	NA	12.72	0.27	0.16		0.318619	-0.34482	-1.4069		-0.14646	NA	49.83	NA	36.50	-0.39	0.57
P07741	Adenine phosphoribosyltransferase	APRT	4.50		-0.39094	0.970275		0.522422	0.938871	NA	NA	20.27	42.66	0.51	0.41		0.091773	-0.38457		0.643914	0.735702	NA	NA	4.50	37.44	0.27	0.71
Q9Y3I0	tRNA-splicing ligase RtcB homolog	RTCB	3.00		0.472761	0.812458		0.388088	-0.09311	NA	NA	23.37	22.36	0.40	0.13		0.205069	-1.06973		0.057562	-0.81132	NA	NA	41.34	41.68	-0.40	0.38
C9J0K6	Sorcin	SRI	2.50		-0.06087	-0.00877		0.484858	-1.52584	NA	NA	85.19	6.58	-0.28	0.35		-0.29616	-0.21755		-0.26954	-0.21414	NA	NA	2.72	2.78	-0.25	0.01
P35914	Hydroxymethylglutaryl-CoA lyase, mitochondrial	HMGCL	1.00		-0.29117	-1.43792		-0.37612	0.152612	NA	NA	25.63	42.43	-0.49	0.28		0.358009	-0.34772		-0.09155	0.577127	NA	NA	32.20	24.60	0.12	0.74
B2R6K4	cDNA, FLJ92996, highly similar to Homo sapiens guanine nucleotide binding protein (G protein), beta polyp	NA	26.50		-1.23581		-2.11192	-1.91537	-1.62654	NA	NA	14.11	31.11	-1.72	0.02		-1.21821		-1.93222	-1.32027	-1.19858	NA	NA	5.96	25.32	-1.42	0.02
C9JIF9	Acylamino-acid-releasing enzyme	APEH	1.50		0.564699		-0.52354	1.150969	-0.20644	NA	NA	62.02	38.09	0.25	0.67		0.638248		-0.5345	1.181162	0.198228	NA	NA	46.40	41.64	0.37	0.58
Q53F48	Heterogeneous nuclear ribonucleoprotein H3 isoform a variant (Fragment)	NA	2.75		1.373245		1.192468	-0.06996	0.378904	NA	NA	21.82	38.60	0.72	0.14		1.184663		1.394131	-0.066	0.251355	NA	NA	15.49	40.93	0.69	0.16
Q53FV3	COP9 signalosome subunit 4 variant (Fragment)	NA	1.50		0.096723		-0.43214	0.321747	0.221505	NA	NA	4.91	23.82	0.05	0.93		-0.81903		-0.685	0.325522	-0.06851	NA	NA	19.19	38.63	-0.31	0.26
Q01650	Large neutral amino acids transporter small subunit 1	SLC7A5	1.25		0.873917		0.863226	3.253014	1.83875	NA	NA	64.25	80.25	1.71	0.13		1.259375		1.010685	3.038763	1.584927	NA	NA	65.78	59.14	1.72	0.06
A0A024QZQ2	Prosaposin (Variant Gaucher disease and variant metachromatic leukodystrophy), isoform CRA_b	PSAP	20.75		-0.80997		-0.47583	-0.46595	-0.76739	NA	NA	14.72	11.51	-0.63	0.02		-0.78294		-1.1279	-0.12431	-0.35929	NA	NA	11.49	31.76	-0.60	0.11
G3V4C1	Heterogeneous nuclear ribonucleoproteins C1/C2	HNRNPC	5.25		-0.58886		0.303333	0.179516	0.225016	NA	NA	2.23	30.24	0.03	0.93		-0.45564		0.403891	-0.17734	-0.11296	NA	NA	3.15	30.96	-0.09	0.82
J3QTR3	Ubiquitin-40S ribosomal protein S27a (Fragment)	RPS27A	5.00		0.097256		0.216604	0.209807	0.323963	NA	NA	5.59	6.00	0.21	0.06		0.083062		-0.04147	-0.2871	-0.17857	NA	NA	5.32	10.85	-0.11	0.56
P53618	Coatomer subunit beta	COPB1	1.25			-0.27672	0.76736	0.195474	0.538333	NA	49.05	16.73	1.93	0.31	0.12			-0.68015	0.76644	0.137936	-0.06559	NA	65.50	9.96	8.69	0.04	0.06
A0A024R4G1	Leucine rich repeat containing 47, isoform CRA_a	LRRC47	1.25			-0.02405	0.888509	-0.29379	0.279477	NA	43.29	27.73	23.41	0.21	0.51			-0.12882	-0.45972	-0.07948	0.435815	NA	16.15	24.99	23.59	-0.06	0.85
A8K168	Malic enzyme	NA	4.75			-1.04605	-0.44644	-0.24609	-0.81557	NA	28.97	27.56	10.39	-0.64	0.11			-1.03561	-1.29307	-0.65237	-1.0087	NA	12.59	17.38	16.54	-1.00	0.11
B2RBR9	cDNA, FLJ95650, highly similar to Homo sapiens karyopherin (importin) beta 1 (KPNB1), mRNA	NA	7.00			-0.38021	0.28614	-0.22846	0.196932	NA	32.09	20.70	0.43	-0.03	0.29			-0.3317	0.304614	-0.6404	-0.10327	NA	30.69	26.03	17.95	-0.19	0.48
B2RDE1	cDNA, FLJ96568, highly similar to Homo sapiens tropomyosin 3 (TPM3), mRNA	NA	9.25			0.523136	0.175411	0.333554	0.178069	NA	16.96	7.61	4.99	0.30	0.10			0.897633	0.716021	0.744103	0.814509	NA	8.89	3.45	1.47	0.79	0.01
B7Z2R9	cDNA FLJ52540, highly similar to Lysosome-associated membrane glycoprotein 2	NA	4.50			-0.15811	0.172607	0.100491	0.216405	NA	16.14	5.68	7.00	0.08	0.47			-0.88034	-1.19391	0.050191	0.324474	NA	15.31	13.40	56.57	-0.42	0.61

				08/017 hESC-RPE to hRPE												08/023 hESC-RPE to hRPE											
				Analysis						Technical replicate CV			Biological	Fold change (mean)	Unadjusted p-value	Analysis						Technical replicate CV			Biological	Fold change (mean)	Unadjusted p-value
UniProt	Protein name	Gene name	Peptide mean	1.1	1.2	2.1	2.2	3.1	3.2	1	2	3	CV		(t-test)	1.1	1.2	2.1	2.2	3.1	3.2	1	2	3	CV		(t-test)
G8JLB6	Heterogeneous nuclear ribonucleoprotein H	HNRNPH1	6.50			0.746529	0.595168	-0.19752	0.106755	NA	7.41	14.86	34.12	0.31	0.54			0.583865	0.171802	-0.25516	-0.46259	NA	20.06	10.15	35.85	0.01	0.98
I3L3D7	Serine protease 33 (Fragment)	PRSS33	1.00			0.844695	0.661671	1.241487	1.228699	NA	8.96	0.63	23.26	0.99	0.15			0.656625	0.829712	0.173207	0.409621	NA	8.47	11.56	21.86	0.52	0.26
Q53F64	Heterogeneous nuclear ribonucleoprotein AB isoform a variant (Fragment)	NA	2.50			0.689326	0.99088	-1.71898	-1.3153	NA	14.73	19.66	95.07	-0.34	0.82			1.011845	0.393555	-1.99443	-1.06313	NA	29.85	44.12	90.59	-0.41	0.77
Q53FM7	NADH dehydrogenase (Ubiquinone) Fe-S protein 3, 30kDa (NADH-coenzyme Q reductase) variant (Fragmer	NA	6.75			-0.7964	-0.40536	-0.28321	0.030486	NA	19.05	15.31	22.83	-0.36	0.37			-0.98264	-0.57566	0.335061	0.431694	NA	19.82	4.73	53.52	-0.20	0.79
Q53H01	N-acylsphingosine amidohydrolase (Acid ceramidase) 1 preproprotein isoform a variant (Fragment)	NA	9.00			-1.28607	-1.00859	0.035827	-0.33834	NA	13.56	18.24	47.21	-0.65	0.42			-1.90619	-1.46423	0.351685	0.415091	NA	21.49	3.11	86.47	-0.65	0.64
Q59F66	DEAD box polypeptide 17 isoform p82 variant (Fragment)	NA	3.00			0.418942	0.480244	0.499962	0.172562	NA	3.00	15.98	5.11	0.39	0.09			0.69183	0.367189	0.626875	-0.5486	NA	15.84	54.62	18.66	0.28	0.45
Q68DH9	Calcium-transporting ATPase	DKFZp686M088	4.00			-0.47355	-1.04294	-0.65045	-0.7124	NA	27.55	3.04	2.41	-0.72	0.03			-0.7309	-1.15517	-0.66346	-0.70733	NA	20.65	2.15	11.85	-0.81	0.10
Q6IPX5	Ribosomal protein S2	RPS2	3.75			0.185304	-0.00938	0.236275	0.733627	NA	9.53	24.14	20.20	0.29	0.39			0.02906	0.114681	0.123962	0.528974	NA	4.20	19.72	13.10	0.20	0.36
Q96DV6	40S ribosomal protein S6	RPS6	1.75			1.252037	-0.55845	0.421141	1.052935	NA	78.67	30.48	7.71	0.54	0.22			1.132641	0.035889	0.60882	0.753336	NA	51.31	7.08	0.16	0.63	0.05
Q9UM54	Unconventional myosin-VI	MYO6	1.50			-0.63828	-1.10964	-0.96499	-1.16989	NA	22.90	10.03	10.23	-0.97	0.06			-0.7018	-0.72324	-0.47061	-1.6434	NA	1.05	54.51	11.18	-0.88	0.12
A0A024R4I7	Leucine zipper and CTNNBIP1 domain containing, isoform CRA_a	LZIC	1.75			0.793009	0.320112	0.814934	0.071413	NA	22.97	35.66	4.18	0.50	0.07			0.390279	0.027743	-0.05264	-0.46112	NA	17.68	19.89	22.49	-0.02	0.93
A8K4C8	60S ribosomal protein L13	RPL13	3.25			0.90383	0.336699	0.649858	0.22888	NA	27.44	20.49	9.46	0.53	0.11			0.602319	0.211791	-0.18132	0.432963	NA	19.02	29.66	12.80	0.27	0.31
C9J3L8	Translocon-associated protein subunit alpha	SSR1	1.25			-0.34443	-0.00992	-1.64454	0.476192	NA	16.32	88.55	2.83	-0.38	0.31			-0.26724	-0.48457	-0.73979	0.697794	NA	10.63	65.16	25.35	-0.20	0.46
Q1HBJ4	Mitogen-activated protein kinase	MAPK1	2.75			0.147517	-0.26823	-1.00933	-0.71193	NA	20.24	14.52	38.58	-0.46	0.46			-0.25904	-0.13121	-0.91625	-0.41073	NA	6.26	24.53	21.77	-0.43	0.32
O94826	Mitochondrial import receptor subunit TOM70	TOMM70A	10.00	0.038842	0.069666	-1.39129				1.51	NA	NA	65.47	-0.43	0.52	0.093945	-0.18126	-1.22315				13.45	NA	NA	55.06	-0.44	0.48
Q05639	Elongation factor 1-alpha 2	EEF1A2	10.00	-1.40686	-2.82032	-1.24842				64.22	NA	NA	33.59	-1.83	0.16	-1.7233	-1.2535	-0.5913				22.82	NA	NA	41.76	-1.19	0.26
Q9NUN5	Probable lysosomal cobalamin transporter	LMBRD1	1.00	-0.18208	0.963669	-0.11241				53.38	NA	NA	29.65	0.22	0.68	0.255198	0.38644	0.050722				6.43	NA	NA	13.27	0.23	0.40
A0A024R3V8	Translin-associated factor X, isoform CRA_c	TSNAX	1.00	-0.73874	-1.29886	1.513048				27.11	NA	NA	99.05	-0.17	0.88	-0.87578	-1.26617	-0.33777				19.02	NA	NA	34.58	-0.83	0.31
A0A024R9I0	ATPase, H+ transporting, lysosomal 42kDa, V1 subunit C1, isoform CRA_a	ATP6V1C1	2.33	-0.42868	-0.4893	-0.78585				2.97	NA	NA	15.97	-0.57	0.16	-0.18241	-0.65148	-0.44806				22.79	NA	NA	2.45	-0.43	0.02
A0A024RA98	Peptidyl arginine deiminase, type II, isoform CRA_a	PADI2	2.33	-0.14918	0.395063	-0.20195				26.36	NA	NA	17.09	0.01	0.85	-0.31406	-0.24617	-0.66282				3.33	NA	NA	18.67	-0.41	0.25
A6NP24	Quinone oxidoreductase (Fragment)	CRYZ	2.33	-0.10448	0.06858	-1.73159				8.47	NA	NA	75.43	-0.59	0.49	-0.38525	0.287542	-1.04969				32.39	NA	NA	48.86	-0.38	0.47
B4E106	cDNA FLJ53399, highly similar to Monocarboxylate transporter 1	NA	2.67	0.907743	1.070932	0.107975				7.99	NA	NA	42.01	0.70	0.43	0.982141	1.154491	1.976721				8.44	NA	NA	42.99	1.37	0.18
B4E192	cDNA FLJ54224, highly similar to Glial fibrillary acidic protein, astrocyte	NA	15.00	-0.7299	-1.0776	-1.51992				16.96	NA	NA	30.24	-1.11	0.16	-1.16757	-0.98968	-1.1189				8.71	NA	NA	2.11	-1.09	0.01
B7ZAC0	cDNA, FLJ79134, highly similar to Dynamin-1 (EC 3.6.5.5)	NA	3.33	0.483373	-0.0778	0.245121				27.16	NA	NA	0.75	0.22	0.06	0.035282	0.056397	0.167082				1.03	NA	NA	5.94	0.09	0.33
K7ELJ7	Calpain small subunit 1	CAPNS1	4.00	1.354202	0.261293	0.111414				51.14	NA	NA	38.12	0.58	0.41	0.96776	0.312776	0.111247				31.56	NA	NA	27.39	0.46	0.39
Q53GD0	Hydroxysteroid (17-beta) dehydrogenase 2 variant (Fragment)	NA	2.33	0.389719	2.495897	-0.11585				88.11	NA	NA	82.08	0.92	0.55	0.165829	2.274029	0.621855				88.17	NA	NA	45.09	1.02	0.20
Q7Z4F3	Caveolin	NA	1.00	-1.34155	-0.07001	-1.36306				58.58	NA	NA	37.92	-0.92	0.20	-1.0445	0.200474	0.422735				57.50	NA	NA	34.32	-0.14	1.00
S4R435	Protein RPS10-NUDT3 (Fragment)	RPS10-NUDT3	1.33	0.294865	-0.26265	0.245568				26.99	NA	NA	9.92	0.09	0.46	0.047843	0.526424	0.091432				23.24	NA	NA	10.54	0.22	0.30
V9HWF6	Alpha-1-acid glycoprotein	HEL-S-153w	1.33	-2.86212	-1.29234	-1.2984				70.15	NA	NA	27.82	-1.82	0.14	-1.68686	-1.89772	-0.84037				10.32	NA	NA	44.86	-1.47	0.22
Q8NF37	Lysophosphatidylcholine acyltransferase 1	LPCAT1	1.33	-0.42815	-0.44418		-1.37701			0.79	NA	NA	44.55	-0.75	0.30	-0.10284	-0.98487		-1.99019			41.93	NA	NA	68.02	-1.03	0.33
Q9P0L0	Vesicle-associated membrane protein-associated protein A	VAPA	2.67	0.310029	-0.13648		-0.27619			21.71	NA	NA	18.53	-0.03	0.69	0.502449	-0.19684		0.089762			33.62	NA	NA	5.14	0.13	0.16
A0A024QZB4	Sulfotransferase	hCG_1993905	1.33	-0.64004	0.313857		0.268958			45.12	NA	NA	17.29	-0.02	0.85	-0.26637	-0.01952		0.566933			12.07	NA	NA	33.86	0.09	0.66
B2R6D0	cDNA, FLJ92896, highly similar to Homo sapiens proteasome (prosome, macropain) 26S subunit, non-ATPa	NA	2.00	0.935901	0.619598		0.592198			15.44	NA	NA	9.50	0.72	0.09	0.808607	0.502047		0.404082			14.97	NA	NA	12.68	0.57	0.15
B4DX69	cDNA FLJ55158, highly similar to Thioredoxin, mitochondrial	NA	2.33	-0.03709	-0.47261		-0.96214			21.19	NA	NA	34.74	-0.49	0.34	0.43727	-0.40399		-1.27281			40.10	NA	NA	61.72	-0.41	0.51
F4ZW62	NF45	NA	3.67	-0.43586	-0.25652		1.082364			8.78	NA	NA	64.70	0.13	0.70	0.065121	0.326021		1.29395			12.75	NA	NA	51.13	0.56	0.40
Q6IBA0	NADH dehydrogenase (Ubiquinone) Fe-S protein 5, 15kDa (NADH-coenzyme Q reductase)	NDUFS5	2.00	-0.32814	-0.06881		-1.77052			12.68	NA	NA	70.45	-0.72	0.43	-1.43979	-0.22824		-1.18413			56.12	NA	NA	23.01	-0.95	0.11
Q9H3K5	My017 protein	NA	2.67	-0.23833	0.294331		-0.17659			25.81	NA	NA	11.20	-0.04	0.60	-0.43979	0.258178		-0.53596			33.56	NA	NA	23.64	-0.24	0.39
D3DQ84	Acyl-CoA synthetase long-chain family member 6, isoform CRA_e	ACSL6	4.33	-0.47417	0.301126		-1.00647			37.11	NA	NA	45.89	-0.39	0.45	0.399229	0.105766		-0.61724			14.33	NA	NA	41.72	-0.04	0.75
P61604	10 kDa heat shock protein, mitochondrial	HSPE1	7.33	0.52446	0.484206			0.901593		1.97	NA	NA	19.34	0.64	0.18	0.917834	0.766584			0.606649		7.41	NA	NA	11.62	0.76	0.10
P62195	26S protease regulatory subunit 8	PSMC5	2.33	0.38445	-0.12236			-0.22928		24.59	NA	NA	18.64	0.01	0.83	0.535932	-0.08136			0.062322		29.80	NA	NA	9.68	0.17	0.33
Q53GE3	Pyruvate dehydrogenase E1 component subunit alpha (Fragment)	NA	4.00	-0.56008	-0.19438			-0.48441		17.83	NA	NA	5.82	-0.41	0.08	-0.45864	-0.38142			-0.83359		3.78	NA	NA	20.16	-0.56	0.20
Q53HB7	Diablo isoform 1 variant (Fragment)	NA	2.33	0.933739	0.3807			0.690789		26.78	NA	NA	0.35	0.67	0.02	1.015474	0.853788			0.596291		7.92	NA	NA	16.62	0.82	0.14
P37108	Signal recognition particle 14 kDa protein	SRP14	2.00	-0.43666	0.373831			-1.10952		38.71	NA	NA	52.90	-0.39	0.48	-0.13834	0.66948			-2.03962		38.59	NA	NA	95.34	-0.50	0.58
P36405	ADP-ribosylation factor-like protein 3	ARL3	2.67	0.166224	-0.13585				-0.40415	14.75	NA	NA	20.79	-0.12	0.52	0.711589	-0.23238				-0.36617	44.68	NA	NA	32.80	0.04	0.87
P55795	Heterogeneous nuclear ribonucleoprotein H2	HNRNPH2	6.67	0.905538	0.264299				-1.2326	30.92	NA	NA	80.10	-0.02	0.78	0.101112	0.382726				-0.86373	13.76	NA	NA	51.98	-0.13	0.67
Q8IVA1	Purkinje cell protein 2 homolog	PCP2	1.33	-0.3274	-0.73982				-0.11192	20.08	NA	NA	19.82	-0.39	0.37	-0.20068	-0.89526				-0.28046	33.40	NA	NA	11.06	-0.46	0.20
Q8IVF2	Protein AHNAK2	AHNAK2	1.33	-1.07743	-0.20989				-0.56733	41.28	NA	NA	0.59	-0.62	0.04	-0.50018	-0.01595				-0.4623	23.51	NA	NA	10.98	-0.33	0.18
B4DFY5	Mitogen-activated protein kinase kinase 1, isoform CRA_d	MAP2K1	1.00	-1.3321	-0.67746				0.376728	31.55	NA	NA	61.52	-0.54	0.73	-0.53437	-2.48186				-1.25622	83.19	NA	NA	2.67	-1.42	0.06
B4DH02	cDNA FLJ50510, highly similar to Heat shock 70 kDa protein 4	NA	8.33	0.77887	-0.36138				-0.30968	53.15	NA	NA	30.32	0.04	0.88	0.372566	-0.3777				-0.37308	35.97	NA	NA	20.38	-0.13	0.50
B4DLR3	cDNA FLJ54020, highly similar to Heterogeneous nuclear ribonucleoprotein U	NA	10.00	-0.31927	-0.50754				-0.09824	9.21	NA	NA	15.24	-0.31	0.35	-0.3586	-0.23186				-0.36562	6.21	NA	NA	3.52	-0.32	0.07
Q59ER5	WD repeat-containing protein 1 isoform 1 variant (Fragment)	NA	8.33	0.55466	0.754888				0.472224	9.80	NA	NA	9.10	0.59	0.10	0.496714	0.643054				0.682134	7.17	NA	NA	5.41	0.61	0.06
Q5TZC3	Protein kinase C and casein kinase substrate in neurons 1, isoform CRA_a	PACSIN1	1.33	-0.91793	0.508962				-0.83284	64.74	NA	NA	38.15	-0.41	0.35	-0.81554	-0.27787				-1.38729	26.05	NA	NA	41.19	-0.83	0.26
Q96HX3	Dolichyl-diphosphooligosaccharide--protein glycosyltransferase subunit 1 (Fragment)	NA	7.33	0.123495	0.123631				0.782593	0.01	NA	NA	31.75	0.34	0.40	0.285537	0.238327				0.527044	2.31	NA	NA	12.95	0.35	0.21
W8QEY1	Lactoferrin	NA	3.00	1.911673	1.380773				1.736472	25.73	NA	NA	3.23	1.68	0.02	1.926334	1.017848				2.163906	43.11	NA	NA	30.00	1.70	0.12
A0A024RBE7	Thymopoietin, isoform CRA_c	TMPO	3.33	-0.208	-0.02161				-0.87862	9.12	NA	NA	36.72	-0.37	0.42	-0.41825	-0.34179				0.382286	3.75	NA	NA	36.49	-0.13	1.00
P23786	Carnitine O-palmitoyltransferase 2, mitochondrial	CPT2	5.33	0.223994		-1.2984	-1.48577			NA	9.17	NA	71.74	-0.85	0.60	0.174009		-0.99067	-1.56965			NA	28.00	NA	64.68	-0.80	0.59
P35613	Basigin	BSG	8.67	-2.19735		-0.83417	-0.69432			NA	6.85	NA	65.05	-1.24	0.29	-1.26479		-0.5033	-0.66846			NA	8.09	NA	32.78	-0.81	0.22
Q92896	Golgi apparatus protein 1	GLG1	1.67	0.703269		-0.14048	0.814141			NA	45.15	NA	14.11	0.46	0.22	0.986903		0.547448	0.662341			NA	5.63	NA	18.56	0.73	0.15
A0A024R0P9	Translocase of outer mitochondrial membrane 40 homolog (Yeast), isoform CRA_c	TOMM40	1.67	0.426068		0.429411	-0.29904			NA	34.96	NA	15.40	0.19	0.40	-0.12776		-0.02862	-0.00937			NA	0.94	NA	5.33	-0.06	0.41
A0A024R3W2	Translocase of outer mitochondrial membrane 20 homolog (Yeast), isoform CRA_a	TOMM20	1.67	0.675313		-0.79778	-0.1051			NA	33.31	NA	50.83	-0.08	0.88	0.464363		-0.58332	0.259371			NA	40.17	NA	27.38	0.05	0.71
A0A024R9P6	Family with sequence similarity 82, member C, isoform CRA_a	FAM82C	2.00	-0.77533		0.12776	-0.22261			NA	17.09	NA	35.43	-0.29	0.46	-0.47773		-0.19565	-0.56218			NA	17.87	NA	5.41	-0.41	0.07
B3KMV8	cDNA FLJ12766 fis, clone NT2RP2001520, highly similar to Calcium-binding mitochondrial carrier protein A	NA	9.00	0.079649		-0.23401	-0.63744			NA	19.65	NA	24.33	-0.26	0.62	-0.74774		-0.4484	-0.9414			NA	23.93	NA	3.62	-0.71	0.02
C9JAZ1	Metaxin-2 (Fragment)	MTX2	4.67	-0.53078		-0.5948	0.035805			NA	30.43	NA	13.95	-0.36	0.19	-1.13448		-1.77483	-0.07218			NA	74.95	NA	21.82	-0.99	0.07
D9ZGG2	Vitronectin	VTN	2.00	-0.06461		-1.42221	-0.94073			NA	23.38	NA	51.31	-0.81	0.47	-0.07303		-1.07559	-0.93318			NA	6.97	NA	44.05	-0.69	0.45
H0Y695	Aminomethyltransferase (Fragment)	AMT	1.33	-0.91348		-0.90686	-1.5994			NA	33.31	NA	14.58	-1.14	0.10	-0.6844		-2.83856	-2.25463			NA	28.24	NA	79.43	-1.93	0.33
Q6ZS99	cDNA FLJ45706 fis, clone FEBRA2028457, highly similar to Nucleolin	NA	10.00	-0.08243		1.21095	1.478447			NA	13.07	NA	64.99	0.87	0.54	-0.22942		1.452629	1.42378			NA	1.41	NA	73.71	0.88	0.60
X5DQN2	Aldehyde dehydrogenase 5 family member A1 isoform B (Fragment)	ALDH5A1	5.33	-0.10723		-0.4813	-0.86291			NA	18.60	NA	26.74	-0.48	0.40	0.352112		-1.00431	-1.57115			NA	27.43	NA	71.71	-0.74	0.67
P18077	60S ribosomal protein L35a	RPL35A	1.00	0.47107		-0.1329	0.483264			NA	29.75	NA	12.87	0.27	0.27	0.522113		0.371232	0.563522			NA	9.41	NA	2.53	0.49	0.04
P49720	Proteasome subunit beta type-3	PSMB3	2.00	-0.67418		-0.58205	-0.64319			NA	3.00	NA	3.03	-0.63	0.03	-0.51013		-0.27273	-0.13647			NA	6.67	NA	15.00	-0.31	0.26
Q6NVY1	3-hydroxyisobutyryl-CoA hydrolase, mitochondrial	HIBCH	2.33	-0.57731		-0.53828	-0.81065			NA	13.31	NA	4.45	-0.64	0.05	-1.09136		-1.40956	-0.52068			NA	42.24	NA	9.48	-1.01	0.04
Q9UK45	U6 snRNA-associated Sm-like protein LSm7	LSM7	1.00	-0.02022		-0.49992	-0.0773			NA	20.57	NA	12.37	-0.20	0.46	0.131035		0.244527	-0.92981			NA	54.58	NA	17.43	-0.18	0.73
Q7Z4Y4	GTP:AMP phosphotransferase AK3, mitochondrial	AK3	2.33	0.592097		0.898295	-0.13518			NA	48.59	NA	5.87	0.45	0.14	0.951083		0.865214	0.372462			NA	23.92	NA	15.20	0.73	0.13
A0A024R8G3	Prostaglandin D2 synthase 21kDa (Brain), isoform CRA_a	PTGDS	2.00	0.512781		1.188913		0.157125		NA	NA	NA	37.52	0.62	0.18	0.394069		1.093331		0.637548		NA	NA	NA	25.25	0.71	0.07
B3KNK4	Phosphatidate cytidylyltransferase	NA	3.33	-0.74104		-0.55844		-0.40175		NA	NA	NA	11.70	-0.57	0.03	-0.6844		-0.56075		-0.92722		NA	NA	NA	12.64	-0.72	0.02
Q6DHZ2	Protein kinase, cAMP-dependent, regulatory, type II, beta	PRKAR2B	4.67	-1.6626		1.217462		-0.22225		NA	NA	NA	89.16	-0.22	0.81	-1.68969		1.223224		-0.16918		NA	NA	NA	88.52	-0.21	0.82
Q96GX3	KIAA0118 protein (Fragment)	NA	2.33	-0.43027		-1.6231		-0.34153		NA	NA	NA	41.33	-0.80	0.19	-0.05813		-1.53302		-0.68358		NA	NA	NA	47.91	-0.76	0.22
B2R7U5	cDNA, FLJ93607	NA	2.67	-0.1198		0.096369			0.066697	NA	NA	NA	7.94	0.01	0.85	0.241362		0.0819			0.712241	NA	NA	NA	23.62	0.35	0.21
E5KLM2	Mitochondrial dynamin-like 120 kDa protein	NA	4.00	0.489274		-0.7722			-0.37912	NA	NA	NA	46.70	-0.22	0.61	0.091273		-0.49508			-0.03939	NA	NA	NA	20.16	-0.15	0.49
F8WCE4	Synaptogyrin-1	SYNGR1	1.00	0.086339		0.48747			-0.37197	NA	NA	NA	29.20	0.07	0.81	0.315075		0.041408			-0.21215	NA	NA	NA	18.26	0.05	0.78
G3V4F2	Acyl-coenzyme A thioesterase 1	ACOT1	4.33	0.469223		-0.31018			-0.77704	NA	NA	NA	44.69	-0.21	0.63	0.38319		-0.18468			-0.74487	NA	NA	NA	38.42	-0.18	0.63
Q4W4Y1	Dopamine receptor interacting protein 4	DRIP4	5.00	0.188851		0.314114			-0.03853	NA	NA	NA	12.16	0.15	0.27	0.505076		0.436264			-0.19759	NA	NA	NA	24.59	0.25	0.38
Q5VWC4	26S proteasome non-ATPase regulatory subunit 4	PSMD4	1.00	0.586864		1.060727			1.173314	NA	NA	NA	20.29	0.94	0.03	0.460228		0.831198			1.256651	NA	NA	NA	27.61	0.85	0.07
X5DQV1	Collapsin response mediator protein 1 isoform B (Fragment)	CRMP1	10.67	-0.99626		-0.73511			-1.26824	NA	NA	NA	18.37	-1.00	0.02	-1.59067		-0.80192			-1.2701	NA	NA	NA	27.90	-1.22	0.03
A0A024R7X3	ATPase, H+ transporting, lysosomal 50/57kDa, V1 subunit H, isoform CRA_b	ATP6V1H	4.67	-0.04255		-0.77583			-0.60531	NA	NA	NA	27.87	-0.47	0.17	-0.2556		-0.78875			-0.22536	NA	NA	NA	20.44	-0.42	0.15
Q24JU4	Eukaryotic translation initiation factor 3 subunit A	EIF3A	2.00	-0.12623		0.617892			-0.03505	NA	NA	NA	29.86	0.15	0.58	0.805977		0.924814			0.70897	NA	NA	NA	7.52	0.81	0.01
P28070	Proteasome subunit beta type-4	PSMB4	2.67	-0.30218			0.230153	-1.10802		NA	NA	NA	43.45	-0.39	0.42	1.122103			-0.77887	-0.08299		NA	NA	NA	67.69	0.09	0.89
A8KAK1	cDNA FLJ77398, highly similar to Homo sapiens UDP-glucose ceramide glucosyltransferase-like 1, transcript	NA	2.00	0.80299			0.430643	-0.15799		NA	NA	NA	31.93	0.36	0.33	1.167391			0.104389	-0.59128		NA	NA	NA	61.81	0.23	0.70
B7Z737	cDNA FLJ52784, highly similar to Bcl-2-like 13 protein	NA	4.33	0.618621			-0.1346	0.048589		NA	NA	NA	28.51	0.18	0.52	0.638869			0.025265	0.271385		NA	NA	NA	21.71	0.31	0.22
Q53FH6	Mitogen-activated protein kinase kinase 1 interacting protein 1 variant (Fragment)	NA	1.00	0.221633			0.598654	0.050615		NA	NA	NA	19.98	0.29	0.21	1.161917			0.253879	0.166221		NA	NA	NA	41.17	0.53	0.24
Q6FHQ0	RBBP7 protein (Fragment)	RBBP7	1.00	-0.00138			0.678241	0.906597		NA	NA	NA	30.03	0.53	0.19	0.15071			0.073546	0.281458		NA	NA	NA	7.35	0.17	0.11
Q8TBI1	Amine oxidase [flavin-containing]	MAOB	1.67	0.214016			0.307386	-0.09706		NA	NA	NA	14.15	0.14	0.37	0.262523			0.055933	0.500005		NA	NA	NA	15.44	0.27	0.17
V9HW01	Epididymis secretory protein Li 310	RPL24	1.00	-0.30024			-1.05823		0.340911	NA	NA	NA	46.28	-0.34	0.49	-0.54392			0.918235		-0.04623	NA	NA	NA	53.29	0.11	0.82
A0A024QYX3	RNA binding motif (RNP1, RRM) protein 3, isoform CRA_c	RBM3	1.00	0.146797				-1.5667	-1.89115	NA	NA	15.84	80.55	-1.10	0.55	-0.49647				-0.73979	-1.88216	NA	NA	53.24	33.85	-1.04	0.27
B4DEA3	cDNA FLJ56531, highly similar to UV excision repair protein RAD23 homolog B	NA	4.00	0.432208				0.310857	-0.06637	NA	NA	18.38	14.54	0.23	0.32	0.326304				0.013379	-0.16171	NA	NA	8.57	19.38	0.06	0.64

				08/017 hESC-RPE to hRPE												08/023 hESC-RPE to hRPE											
				Analysis						Technical replicate CV			Biological	Fold change (mean)	Unadjusted p-value	Analysis						Technical replicate CV			Biological	Fold change (mean)	Unadjusted p-value
UniProt	Protein name	Gene name	Peptide mean	1.1	1.2	2.1	2.2	3.1	3.2	1	2	3	CV		(t-test)	1.1	1.2	2.1	2.2	3.1	3.2	1	2	3	CV		(t-test)
E9KL35	Epididymis tissue sperm binding protein Li 3a	NA	4.33	1.141453				0.546454	0.681884	NA	NA	6.63	25.48	0.79	0.19	0.858646				0.393686	0.832174	NA	NA	21.33	11.21	0.69	0.11
Q53HM8	Annexin (Fragment)	NA	2.33	0.667031				-0.5293	0.156674	NA	NA	33.00	38.82	0.10	0.67	-0.48815				-0.30717	0.171863	NA	NA	23.27	21.41	-0.21	0.41
Q59EF6	Calpain 2, large [catalytic] subunit variant (Fragment)	NA	1.67	-0.16919				-1.04551	0.789151	NA	NA	79.49	15.37	-0.14	0.09	-0.16585				-0.4732	0.032077	NA	NA	24.51	1.60	-0.20	0.09
Q8N169	Amino acid transporter	SLC1A3	3.33	-1.35357				-2.00792	-1.41957	NA	NA	28.44	16.12	-1.59	0.07	-1.6527				-2.50395	-2.32375	NA	NA	8.82	36.34	-2.16	0.12
Q96N86	cDNA FLJ31237 fis, clone KIDNE2004846, highly similar to 26S PROTEASOME REGULATORY SUBUNIT S3	NA	1.33	0.759466				0.168861	-2.24729	NA	NA	96.79	61.44	-0.44	0.90	0.512929				0.428979	-0.78932	NA	NA	56.40	27.49	0.05	0.72
D6RBV2	Vesicular integral-membrane protein VIP36	LMAN2	1.67	0.591966				0.899121	1.020976	NA	NA	5.97	18.01	0.84	0.15	0.376116				0.727589	0.622612	NA	NA	5.14	14.65	0.58	0.18
P16403	Histone H1.2	HIST1H1C	14.67		-1.89178	-0.49692	-0.72493			NA	11.15	NA	59.14	-1.04	0.30		-1.87249	-0.87106	-0.82909			NA	2.06	NA	48.12	-1.19	0.23
P23368	NAD-dependent malic enzyme, mitochondrial	ME2	3.67		0.572379	-0.3583	-0.72536			NA	17.89	NA	51.55	-0.17	0.98		0.961365	-0.16043	-0.54293			NA	18.64	NA	59.75	0.09	0.72
Q9Y230	RuvB-like 2	RUVBL2	2.33		0.890191	1.024833	-0.45184			NA	66.66	NA	20.56	0.49	0.30		0.987496	0.807102	-0.2272			NA	48.63	NA	29.31	0.52	0.32
Q9Y3D6	Mitochondrial fission 1 protein	FIS1	2.00		-0.02373	-0.63293	-1.90425			NA	58.57	NA	51.83	-0.85	0.49		0.099609	-2.42443	-1.48457			NA	44.50	NA	84.19	-1.27	0.53
A8K132	cDNA FLJ75476, highly similar to Homo sapiens glutaminase (GLS), mRNA	NA	5.33		0.445838	-1.11155	-2.00647			NA	42.51	NA	82.84	-0.89	0.68		0.805307	-0.56149	-1.16929			NA	29.36	NA	72.68	-0.31	0.98
B4DHC6	Amino acid transporter	NA	5.33		-1.84876	-1.42292	-0.59183			NA	39.64	NA	42.75	-1.29	0.18		-1.98139	-1.69728	-1.18413			NA	24.89	NA	27.27	-1.62	0.10
B4DWN1	cDNA FLJ52285, highly similar to Vesicular integral-membrane protein VIP36	NA	2.00		0.926499	0.723277	2.92187			NA	90.83	NA	58.91	1.52	0.20		0.259836	1.023967	2.715521			NA	74.56	NA	79.83	1.33	0.41
B7Z4Z4	cDNA FLJ51918, highly similar to Peroxisomal membrane protein PEX14	NA	3.67		-0.1267	-1.67125	-1.28805			NA	18.67	NA	61.34	-1.03	0.45		-0.07787	-1.4876	-1.20259			NA	13.92	NA	58.12	-0.92	0.46
B7ZB83	cDNA, FLJ79447, highly similar to Nucleosome assembly protein 1-like 4	NA	2.00		0.65499	-0.56548	-0.23094			NA	16.32	NA	49.03	-0.05	0.85		0.403533	0.110075	-0.87464			NA	46.47	NA	33.81	-0.12	0.98
C9JKQ2	NADH dehydrogenase [ubiquinone] 1 beta subcomplex subunit 3 (Fragment)	NDUFB3	1.00		-0.86976	-0.78448	-0.52392			NA	12.74	NA	10.83	-0.73	0.09		-1.15404	-0.71919	-0.62112			NA	4.80	NA	23.54	-0.83	0.17
H0Y886	NADH dehydrogenase [ubiquinone] 1 beta subcomplex subunit 5, mitochondrial (Fragment)	NDUFB5	1.00		0.053477	-1.70975	-1.73762			NA	1.37	NA	77.54	-1.13	0.52		-0.21207	-1.17607	-1.07218			NA	5.09	NA	43.23	-0.82	0.38
J3KPE3	Guanine nucleotide-binding protein subunit beta-2-like 1	GNB2L1	3.33		0.760949	1.589461	0.449878			NA	53.12	NA	17.96	0.93	0.09		0.561488	0.911556	0.493193			NA	20.36	NA	7.64	0.66	0.07
Q9UQD4	SH3-containing Grb-2-like 1 protein	NA	1.33		0.108921	1.158245	0.873047			NA	13.93	NA	43.35	0.71	0.43		0.28284	1.587407	0.80436			NA	37.46	NA	45.63	0.89	0.35
Q15257	Serine/threonine-protein phosphatase 2A activator	PPP2R4	2.00		-1.6915	-0.8196	-0.87543			NA	2.74	NA	40.24	-1.13	0.20		-1.00243	-0.94611	-1.17669			NA	11.28	NA	2.66	-1.04	0.02
Q9H936	Mitochondrial glutamate carrier 1	SLC25A22	3.00		-1.9095	-0.31446	-0.55883			NA	11.95	NA	66.71	-0.93	0.36		-1.69078	-1.14105	-0.60453			NA	26.00	NA	40.17	-1.15	0.20
A0A087WV22	60S ribosomal protein L10	RPL10	1.33		0.427324	0.96621	0.672463			NA	14.35	NA	19.46	0.69	0.19		0.121043	1.070016	0.522827			NA	26.50	NA	33.71	0.57	0.40
F8W0J6	Nucleosome assembly protein 1-like 1	NAP1L1	1.33		-1.09453	-0.17029	0.650127			NA	39.16	NA	63.38	-0.20	0.64		-0.6432	-0.91095	-0.80597			NA	5.14	NA	10.48	-0.79	0.09
Q2Q9B7	Glucose-6-phosphate 1-dehydrogenase (Fragment)	G6PD	2.33		-0.26722	-0.16969	0.004348			NA	8.52	NA	9.16	-0.14	0.31		0.093426	-0.19391	-0.41327			NA	10.73	NA	19.14	-0.17	0.69
F8W8I8	Septin-8	SEPT8	2.00		-0.01738	0.406197			-0.20677	NA	NA	NA	22.42	0.06	0.77		0.010976	0.584709			-0.98477	NA	NA	NA	49.51	-0.13	0.80
B3KV96	cDNA FLJ16285 fis, clone OCBBF2004038, highly similar to Dihydropyrimidinase-related protein 1	NA	9.33		-1.24613		-1.33118	-1.71805		NA	NA	NA	16.60	-1.43	0.01		-1.67591		-1.19333	-1.2876		NA	NA	NA	16.88	-1.39	0.01
Q4LE36	ACLY variant protein (Fragment)	ACLY	1.00		0.045406		0.434159	0.021713		NA	NA	NA	16.71	0.17	0.34		-0.05732		0.248366	-0.00663		NA	NA	NA	11.66	0.06	0.58
Q6FHT8	RNP24 protein	RNP24	2.33		0.003981		-1.44142	-0.38504		NA	NA	NA	45.02	-0.61	0.29		0.410016		-2.77714	-1.0244		NA	NA	NA	92.80	-1.13	0.34
V9HW06	Serine hydroxymethyltransferase	SHMT2	1.33		-1.79573		0.26106	-0.7931		NA	NA	NA	67.63	-0.78	0.32		-2.40282		0.276347	-0.73531		NA	NA	NA	77.10	-0.95	0.35
F5GZN3	Lactadherin	MFGE8	2.00		0.526464		0.840144		-0.63551	NA	NA	NA	45.50	0.24	0.64		0.934752		0.925694		0.21271	NA	NA	NA	26.03	0.69	0.10
Q96QV6	Histone H2A type 1-A	HIST1H2AA	7.67		-3.50592			-1.32235	-1.34085	NA	NA	0.91	90.12	-2.06	0.27		-2.9504			-2.06449	-2.05732	NA	NA	0.35	42.27	-2.36	0.11
F4ZW64	NF90a	NA	7.00		0.706501			-0.39236	0.236337	NA	NA	30.34	35.98	0.18	0.57		0.700185			-0.03538	0.270118	NA	NA	14.92	27.80	0.31	0.39
H0YBL1	Inositol monophosphatase 1 (Fragment)	IMPA1	1.33		-0.4115			0.540315	-0.37471	NA	NA	43.40	27.37	-0.08	0.63		-0.65425			0.376267	-0.55362	NA	NA	44.06	30.83	-0.28	0.41
Q53G79	Carnitine O-palmitoyltransferase II, mitochondrial variant (Fragment)	NA	2.00		-0.44341			-0.4619	-0.41673	NA	NA	2.21	0.21	-0.44	0.00		-1.39122			-0.41469	-0.81245	NA	NA	19.37	37.84	-0.87	0.24
Q6IAX2	RPL21 protein	RPL21	1.00		-0.40847			1.163691	0.464972	NA	NA	33.59	58.30	0.41	0.80		0.605167			0.691809	-0.19759	NA	NA	42.26	14.19	0.37	0.25
P08727	Keratin, type I cytoskeletal 19	KRT19	7.67			0.744948	0.760374	0.87501		NA	0.76	NA	5.99	0.79	0.05			1.754672	1.446921	1.08121		NA	15.03	NA	25.58	1.43	0.12
Q70J99	Protein unc-13 homolog D	UNC13D	3.33			1.224058	0.248022	0.496201		NA	46.09	NA	15.66	0.66	0.12			0.71668	0.052531	-0.23312		NA	31.99	NA	31.59	0.18	0.85
Q9UJZ1	Stomatin-like protein 2, mitochondrial	STOML2	5.33			-0.22098	-0.06318	0.549418		NA	7.73	NA	33.16	0.09	0.66			-0.37502	-0.83764	0.726465		NA	22.48	NA	60.30	-0.16	0.94
A0A024R994	Copine III, isoform CRA_a	CPNE3	2.00			-0.46804	-0.29063	-0.66722		NA	8.68	NA	14.20	-0.48	0.17			-0.66522	-0.8417	-1.011		NA	8.64	NA	12.72	-0.84	0.09
B4DN60	cDNA FLJ52703, highly similar to Asparaginyl-tRNA synthetase, cytoplasmic (EC6.1.1.22)	NA	2.33			0.092645	-0.05521	0.135272		NA	7.24	NA	5.62	0.06	0.41			0.440746	-0.24922	0.748786		NA	33.19	NA	29.56	0.31	0.42
Q53EP4	Dolichyl-diphosphooligosaccharide--protein glycosyltransferase subunit 1 (Fragment)	NA	11.33			0.147517	0.272673	0.062175		NA	6.13	NA	7.31	0.16	0.32			0.042883	0.250064	-0.0031		NA	10.14	NA	7.51	0.10	0.51
Q8NAB7	cDNA FLJ35635 fis, clone SPLEN2011805, highly similar to BONE/CARTILAGE PROTEOGLYCAN I	NA	1.00			1.466906	1.495283	0.3524		NA	1.39	NA	52.66	1.10	0.35			2.094576	1.975248	0.990457		NA	5.85	NA	49.12	1.69	0.21
V9HWG7	Epididymis secretory protein Li 52	WDR1	7.00			-0.01921	-0.13258	0.597328		NA	5.55	NA	32.36	0.15	0.58			0.034506	-0.03215	0.781995		NA	3.27	NA	37.35	0.26	0.50
O60888	Protein CutA	CUTA	1.67			0.624441	0.311853	0.802084		NA	15.26	NA	15.89	0.58	0.16			0.839989	1.071867	0.990457		NA	11.34	NA	1.46	0.97	0.01
P68032	Actin, alpha cardiac muscle 1	ACTC1	27.33			-0.39931	-0.05084		0.360638	NA	17.00	NA	27.83	-0.03	0.86			0.0495	0.216577		0.649476	NA	8.18	NA	24.93	0.31	0.37
B4DDL4	cDNA FLJ53966, moderately similar to Homo sapiens cytidylate kinase (CMPK), mRNA	NA	1.67			0.263344	1.799296		1.14029	NA	68.89	NA	4.24	1.07	0.03			1.154178	1.73449		1.484754	NA	28.07	NA	0.56	1.46	0.01
Q4LE63	ATP2B2 variant protein (Fragment)	ATP2B2	1.67			-0.63293	0.774621		0.113563	NA	63.99	NA	6.00	0.09	0.14			0.441864	0.171802		0.2265	NA	13.20	NA	4.25	0.28	0.10
Q5JR08	Rho-related GTP-binding protein RhoC (Fragment)	RHOC	3.67			-0.55649	0.553564		-0.13579	NA	51.87	NA	11.67	-0.05	0.49			0.047787	0.277389		0.116062	NA	11.23	NA	2.50	0.15	0.11
Q9UNM1	Chaperonin 10-related protein (Fragment)	EPFP1	7.67			0.067572	0.218418		0.954853	NA	7.39	NA	38.68	0.41	0.41			0.251984	0.464767		0.592685	NA	10.41	NA	11.27	0.44	0.15
E9PKZ0	60S ribosomal protein L8 (Fragment)	RPL8	1.00			1.567272	-0.31174		1.1219	NA	80.96	NA	10.16	0.79	0.18			1.919539	0.754972		0.7198	NA	54.17	NA	35.12	1.13	0.19
Q71U36	Tubulin alpha-1A chain	TUBA1A	44.00			-1.11729		-0.95114	-1.42563	NA	NA	23.05	2.53	-1.16	0.02			-1.43466		-0.65954	-0.10857	NA	NA	26.68	50.45	-0.73	0.33
A0A087WYV8	Fibrillin-2	FBN2	2.33			1.394953		0.369381	1.421472	NA	NA	49.40	19.75	1.06	0.14			2.219534		0.574239	1.367344	NA	NA	37.92	55.43	1.39	0.24
A8KA83	cDNA FLJ78586, highly similar to Homo sapiens VAMP (vesicle-associated membrane protein)-associated p	NA	2.33			-0.54716		-0.20862	0.278079	NA	NA	23.63	29.10	-0.16	0.54			-0.01654		-0.21716	-0.11227	NA	NA	5.14	7.21	-0.12	0.44
I6L893	Uncharacterized protein	NA	2.33			1.613568		1.295904	0.438936	NA	NA	40.81	32.88	1.12	0.19			-0.1472		-0.01643	-0.62446	NA	NA	29.37	6.93	-0.26	0.23
Q2LE71	Actin-related protein 2/3 complex subunit 3	ARPC3	2.00			0.247132		1.085089	1.050958	NA	NA	1.67	39.19	0.79	0.36			0.094042		0.858003	0.355428	NA	NA	24.39	25.90	0.44	0.40
A0A024R0Q4	Phospholipase D family, member 3, isoform CRA_b	PLD3	1.00			0.347204		0.646111	-0.43019	NA	NA	50.43	6.91	0.19	0.31			0.152813		1.187622	0.392108	NA	NA	38.03	33.24	0.58	0.38
G1EP01	MHC class I antigen (Fragment)	HLA-A	1.67			-1.09674		0.176674	0.313083	NA	NA	6.68	61.46	-0.20	0.64			-1.10196		0.006808	0.447951	NA	NA	21.45	61.57	-0.22	0.63
Q02318	Sterol 26-hydroxylase, mitochondrial	CYP27A1	1.00				0.934339	1.05775	2.142116	NA	NA	50.78	36.65	1.38	0.16				0.308695	0.557055	1.688259	NA	NA	52.77	43.72	0.85	0.33
A0A0A0MSQ0	Plastin-3	PLS3	5.67				0.818483	1.363036	1.229663	NA	NA	6.53	23.28	1.14	0.14				0.875685	0.411985	0.840926	NA	NA	20.87	11.41	0.71	0.10
B4DZ87	cDNA FLJ57240, highly similar to Mitochondrial proteins import receptor	NA	5.67				-1.26063	-0.14665	-0.3654	NA	NA	10.70	47.52	-0.59	0.37				-1.26733	0.494144	0.109329	NA	NA	18.75	70.60	-0.22	0.65
Q53FI7	Four and a half LIM domains 1 variant (Fragment)	NA	2.00				-1.5407	-0.59555	-1.33121	NA	NA	35.30	30.10	-1.16	0.14				-0.5433	-0.81966	-1.20054	NA	NA	18.56	22.08	-0.85	0.19
Q6IAT9	Proteasome subunit beta type	PSMB6	1.00				1.195925	0.460988	-0.27832	NA	NA	35.46	49.64	0.46	0.45				0.769256	0.664021	-0.33154	NA	NA	46.95	25.16	0.37	0.36
A0A0D9SFB1	Dynamin-1	DNM1	4.00				0.189813	-0.91722	-0.0182	NA	NA	42.69	28.46	-0.25	0.75				-0.24115	-0.94622	-0.13725	NA	NA	38.64	11.96	-0.44	0.23
O95881	Thioredoxin domain-containing protein 12	TXNDC12	1.00	-0.10575		-0.0168				NA	NA	NA	4.36	-0.06	0.40	-0.94412		-0.61475				NA	NA	NA	16.07	-0.78	0.13
P31689	DnaJ homolog subfamily A member 1	DNAJA1	1.00	-1.30558		-0.60203				NA	NA	NA	33.82	-0.95	0.22	-0.70497		0.019854				NA	NA	NA	34.80	-0.34	0.52
Q6FI13	Histone H2A type 2-A	HIST2H2AA3	8.50	-1.68269		-1.67041				NA	NA	NA	0.60	-1.68	0.00	-1.39877		-0.65562				NA	NA	NA	35.64	-1.03	0.22
Q9NSE4	Isoleucine--tRNA ligase, mitochondrial	IARS2	3.00	0.989033		0.644588				NA	NA	NA	16.80	0.82	0.13	1.10346		0.773891				NA	NA	NA	16.08	0.94	0.11
A0A024R056	Guanine nucleotide binding protein (G protein), beta polypeptide 1, isoform CRA_a	GNB1	36.50	-1.24042		-2.15752				NA	NA	NA	43.49	-1.70	0.17	-1.27165		-1.9301				NA	NA	NA	31.72	-1.60	0.13
B2R603	cDNA, FLJ92712, highly similar to Homo sapiens heterogeneous nuclear ribonucleoprotein C (C1/C2) (HNRI	NA	5.00	-0.85107		0.1146				NA	NA	NA	45.64	-0.37	0.59	-0.76142		0.046563				NA	NA	NA	38.60	-0.36	0.54
B4DF39	Receptor expression-enhancing protein	NA	1.00	-2.36265		-2.37264				NA	NA	NA	0.49	-2.37	0.00	-2.71752		-2.21256				NA	NA	NA	24.50	-2.47	0.06
B4DKM8	cDNA FLJ52316, highly similar to NADPH--cytochrome P450 reductase (EC 1.6.2.4)	NA	2.00	0.121868		0.398979				NA	NA	NA	13.54	0.26	0.31	0.495627		0.026826				NA	NA	NA	22.78	0.26	0.47
B4DXI8	cDNA FLJ51373, highly similar to 26S proteasome non-ATPase regulatory subunit 7	NA	1.00	0.71902		0.368712				NA	NA	NA	17.09	0.54	0.20	0.227954		0.747153				NA	NA	NA	25.18	0.49	0.31
G3V4P8	Glia maturation factor beta (Fragment)	GMFB	1.00	1.044102		0.758172				NA	NA	NA	13.97	0.90	0.10	0.615461		0.32208				NA	NA	NA	14.33	0.47	0.19
G8JLH6	Tetraspanin (Fragment)	CD9	1.50	-0.68395		-0.91934				NA	NA	NA	11.51	-0.80	0.09	-0.69571		-0.60356				NA	NA	NA	4.52	-0.65	0.05
H0Y360	AMP deaminase 2 (Fragment)	AMPD2	1.00	0.622079		0.785647				NA	NA	NA	8.01	0.70	0.07	0.663426		1.350046				NA	NA	NA	33.03	1.01	0.21
K7EPS4	Matrix-remodeling-associated protein 7	MXRA7	1.00	0.958362		1.357688				NA	NA	NA	19.45	1.16	0.11	1.586568		0.938874				NA	NA	NA	31.22	1.26	0.16
Q59EN5	Prosaposin variant (Fragment)	NA	23.00	-0.6651		-0.3566				NA	NA	NA	15.06	-0.51	0.19	-0.71141		-1.04394				NA	NA	NA	16.23	-0.88	0.12
Q5T4U5	Acyl-Coenzyme A dehydrogenase, C-4 to C-12 straight chain, isoform CRA_a	ACADM	6.00	0.258302		0.134537				NA	NA	NA	6.06	0.20	0.19	0.601376		-0.10522				NA	NA	NA	33.96	0.25	0.61
A4D177	Chromobox homolog 3 (HP1 gamma homolog, Drosophila)	CBX3	1.00	-0.09373		-0.33638				NA	NA	NA	11.87	-0.22	0.33	0.016774		0.349152				NA	NA	NA	16.22	0.18	0.47
H0YCY6	Triokinase/FMN cyclase (Fragment)	TKFC	3.00	-0.93176		0.225985				NA	NA	NA	53.88	-0.35	0.65	-0.66203		-0.0442				NA	NA	NA	29.83	-0.35	0.46
Q02790	Peptidyl-prolyl cis-trans isomerase FKBP4	FKBP4	1.50	-0.34955			0.147122			NA	NA	NA	24.11	-0.10	0.75	-0.64686			0.67662			NA	NA	NA	60.67	0.01	0.99
A0A090N8G0	Glycyl-tRNA synthetase	GARS	2.00	-0.71851			-1.11992			NA	NA	NA	19.55	-0.92	0.14	-1.34936			-0.35563			NA	NA	NA	46.87	-0.85	0.34
Q549J4	Erythroid associated factor	ERAF	1.50	-0.17162			-0.76379			NA	NA	NA	28.62	-0.47	0.36	0.234487			-0.86266			NA	NA	NA	51.32	-0.31	0.67
Q9BR63	FARSB protein (Fragment)	FARSB	1.00	-0.44173			0.346062			NA	NA	NA	37.68	-0.05	0.92	0.254463			-0.11882			NA	NA	NA	18.19	0.07	0.78
Q9BW75	THOP1 protein (Fragment)	THOP1	1.50	2.1276			0.50107			NA	NA	NA	72.23	1.31	0.35	2.054135			0.831557			NA	NA	NA	56.58	1.44	0.26
Q9Y436	Putative uncharacterized protein DKFZp586M2023 (Fragment)	DKFZp586M2023	1.00	0.652953			0.255108			NA	NA	NA	19.38	0.45	0.26	-0.03347			0.712945			NA	NA	NA	35.79	0.34	0.53
W6A4U0	Tetraspanin (Fragment)	CD63	1.00	1.644226			1.087584			NA	NA	NA	26.95	1.37	0.13	1.217909			0.527911			NA	NA	NA	33.19	0.87	0.24
Q14683	Structural maintenance of chromosomes protein 1A	SMC1A	1.00	1.590398			1.520956			NA	NA	NA	3.40	1.56	0.01	1.219604			0.950977			NA	NA	NA	13.13	1.09	0.08
A0A087X1S2	Nuclease-sensitive element-binding protein 1	YBX1	1.50	0.621311			0.946428			NA	NA	NA	15.87	0.78	0.13	1.774235			0.661384			NA	NA	NA	51.99	1.22	0.27
P09960	Leukotriene A-4 hydrolase	LTA4H	2.50	0.807383				0.476141		NA	NA	NA	16.16	0.64	0.16	-0.2248				-0.05672		NA	NA	NA	8.23	-0.14	0.34
P61160	Actin-related protein 2	ACTR2	1.50	1.017997				-0.5041		NA	NA	NA	68.37	0.26	0.79	1.141303				0.04198		NA	NA	NA	51.42	0.59	0.48
E9PKP7	Nucleolar transcription factor 1	UBTF	1.00	0.680358				0.360044		NA	NA	NA	15.64	0.52	0.19	0.338832				0.631688		NA	NA	NA	14.30	0.49	0.19
F5H6I7	Atlastin-3	ATL3	1.00	-0.25773				-0.85724		NA	NA	NA	28.97	-0.56	0.31	-0.14438				-1.57363		NA	NA	NA	64.83	-0.86	0.44
H3BSJ5	Ubiquinone biosynthesis protein COQ9, mitochondrial	COQ9	1.00	-1.66447				-0.33041		NA	NA	NA	61.09	-1.00	0.38	-0.40079				0.091338		NA	NA	NA	23.89	-0.15	0.64

				08/017 hESC-RPE to hRPE												08/023 hESC-RPE to hRPE											
				Analysis						Technical replicate CV			Biological	Fold change (mean)	Unadjusted p-value	Analysis						Technical replicate CV			Biological	Fold change (mean)	Unadjusted p-value
UniProt	Protein name	Gene name	Peptide mean	1.1	1.2	2.1	2.2	3.1	3.2	1	2	3	CV		(t-test)	1.1	1.2	2.1	2.2	3.1	3.2	1	2	3	CV		(t-test)
Q4VB24	Histone cluster 1, H1e	HIST1H1E	16.00	-1.5096				-0.39254		NA	NA	NA	52.17	-0.95	0.34	-1.75475				-1.49227		NA	NA	NA	12.83	-1.62	0.05
Q53HR2	Acyl-Coenzyme A dehydrogenase, very long chain variant (Fragment)	NA	9.00	0.440355				0.135272		NA	NA	NA	14.90	0.29	0.31	0.178663				0.000827		NA	NA	NA	8.71	0.09	0.50
Q59EK7	CS0DF038YO05 variant (Fragment)	NA	2.00	-0.69029				0.310293		NA	NA	NA	47.17	-0.19	0.77	-0.78653				-0.0358		NA	NA	NA	35.99	-0.41	0.47
V5YQL4	Adenosylhomocysteinase	FGFR2-AHCYL1	3.00	0.019163				0.250093		NA	NA	NA	11.29	0.13	0.45	0.509393				0.357023		NA	NA	NA	7.46	0.43	0.11
B3KT66	Proteasome (Prosome, macropain) 26S subunit, non-ATPase, 6, isoform CRA_c	PSMD6	1.50	0.132861				0.100491		NA	NA	NA	1.59	0.12	0.09	-0.31488				0.284856		NA	NA	NA	28.98	-0.02	0.97
A0A024R877	Delta-aminolevulinic acid dehydratase	ALAD	2.00	0.071055					0.511419	NA	NA	NA	21.42	0.29	0.41	-0.10049					-0.03741	NA	NA	NA	3.09	-0.07	0.27
A0A024RAG3	V-ral simian leukemia viral oncogene homolog B (Ras related GTP binding protein), isoform CRA_a	RALB	2.50	-0.34805					0.03021	NA	NA	NA	18.43	-0.16	0.56	-0.79107					-0.01951	NA	NA	NA	36.94	-0.41	0.48
A0A024RCN6	Valyl-tRNA synthetase, isoform CRA_a	VARS	2.00	1.074891					-0.33602	NA	NA	NA	64.12	0.37	0.69	1.236441					0.464958	NA	NA	NA	36.94	0.85	0.27
B4E043	KH domain containing, RNA binding, signal transduction associated 1, isoform CRA_b	KHDRBS1	1.50	0.149107					-0.41994	NA	NA	NA	27.53	-0.14	0.72	-0.02564					-0.54017	NA	NA	NA	24.95	-0.28	0.47
E7EX90	Dynactin subunit 1	DCTN1	2.00	0.301908					-0.27746	NA	NA	NA	28.02	0.01	0.97	0.226831					0.062738	NA	NA	NA	8.03	0.14	0.33
J3KMX5	40S ribosomal protein S13	RPS13	2.00	-0.1213					0.992278	NA	NA	NA	52.02	0.44	0.58	-0.89399					0.893735	NA	NA	NA	77.90	0.00	1.00
Q6FHM9	CD59 antigen, complement regulatory protein, isoform CRA_b	CD59	2.00	0.87849					0.16464	NA	NA	NA	34.29	0.52	0.38	0.68866					0.942564	NA	NA	NA	12.41	0.82	0.10
Q8IZ09	SH3GL2 protein	SH3GL2	1.00	0.359044					-0.4869	NA	NA	NA	40.31	-0.06	0.90	0.484393					-0.09502	NA	NA	NA	28.02	0.19	0.62
Q96BS4	FBL protein (Fragment)	FBL	1.00	1.378784					1.471612	NA	NA	NA	4.55	1.43	0.02	1.321051					1.088036	NA	NA	NA	11.40	1.20	0.06
O60518	Ran-binding protein 6	RANBP6	2.00		0.8584	0.595263				NA	NA	NA	12.86	0.73	0.11		0.855981	-0.62447				NA	NA	NA	66.80	0.12	0.90
Q96HE7	ERO1-like protein alpha	ERO1L	2.00		0.594926	0.02366				NA	NA	NA	27.64	0.31	0.47		0.948177	0.53859				NA	NA	NA	19.94	0.74	0.17
Q9H3Z4	DnaJ homolog subfamily C member 5	DNAJC5	1.50		-0.84876	-0.51196				NA	NA	NA	16.43	-0.68	0.15		-1.06237	-0.48548				NA	NA	NA	27.90	-0.77	0.23
Q9NYU2	UDP-glucose:glycoprotein glucosyltransferase 1	UGGT1	4.00		0.796139	1.717163				NA	NA	NA	43.67	1.26	0.22		0.602847	1.329349				NA	NA	NA	34.87	0.97	0.23
A0A024R3J7	HCG2032701, isoform CRA_a	hCG_2032701	1.50		0.881871	0.482926				NA	NA	NA	19.43	0.68	0.18		-0.10155	-0.06708				NA	NA	NA	1.69	-0.08	0.13
A0A087WX97	Bcl-2-like protein 13	BCL2L13	4.00		0.483935	0.433342				NA	NA	NA	2.48	0.46	0.04		0.464371	0.544328				NA	NA	NA	3.92	0.50	0.05
A0A087X1F5	Protein SYNJ2BP-COX16 (Fragment)	SYNJ2BP-COX16	1.00		0.26066	-0.5379				NA	NA	NA	38.17	-0.14	0.79		-0.19305	-0.80325				NA	NA	NA	29.47	-0.50	0.35
A8K9V9	cDNA FLJ76064	NA	1.50		-0.54499	-0.53252				NA	NA	NA	0.61	-0.54	0.01		-0.16957	-1.37897				NA	NA	NA	56.03	-0.77	0.42
B2RCC2	cDNA, FLJ95978, highly similar to Homo sapiens Y box binding protein 2 (YBX2), mRNA	NA	1.00		0.1657	0.767389				NA	NA	NA	29.07	0.47	0.36		0.371017	0.618727				NA	NA	NA	12.11	0.49	0.16
B4DIH2	cDNA FLJ60563, highly similar to Ganglioside-induceddifferentiation-associated protein 1	NA	2.50		1.020264	-0.13174				NA	NA	NA	53.64	0.44	0.58		1.225663	-0.2761				NA	NA	NA	67.61	0.47	0.64
B7Z8A2	cDNA FLJ51671, highly similar to Prenylcysteine oxidase (EC 1.8.3.5)	NA	1.50		-1.27661	-1.01572				NA	NA	NA	12.75	-1.15	0.07		-1.40224	-1.1156				NA	NA	NA	14.00	-1.26	0.07
F5GXX5	Dolichyl-diphosphooligosaccharide--protein glycosyltransferase subunit DAD1	DAD1	1.00		0.827605	0.276752				NA	NA	NA	26.68	0.55	0.29		0.970165	0.24858				NA	NA	NA	34.65	0.61	0.34
F6T1Q0	2',5'-phosphodiesterase 12	PDE12	1.50		1.319724	0.591574				NA	NA	NA	34.95	0.96	0.23		1.073984	0.810425				NA	NA	NA	12.88	0.94	0.09
F8W7Q4	Protein FAM162A	FAM162A	1.50		0.094236	-0.76723				NA	NA	NA	41.01	-0.34	0.58		-0.32623	-0.74314				NA	NA	NA	20.29	-0.53	0.24
Q08AJ9	Histone H2A	HIST1H2AB	6.50		-0.55723	0.043084				NA	NA	NA	29.01	-0.26	0.55		-0.77387	0.570977				NA	NA	NA	61.52	-0.10	0.90
Q3MHU6	NDUFB1 protein (Fragment)	NDUFB1	1.00		-0.94927	-1.80286				NA	NA	NA	40.66	-1.38	0.19		-1.09307	-0.53779				NA	NA	NA	26.88	-0.82	0.21
Q53F10	UV excision repair protein RAD23 homolog B variant (Fragment)	NA	2.50		-0.57098	-0.28962				NA	NA	NA	13.75	-0.43	0.20		-0.60482	0.666543				NA	NA	NA	58.57	0.03	0.97
Q53GY0	Plastin 3 variant (Fragment)	NA	4.00		1.724823	0.933138				NA	NA	NA	37.86	1.33	0.18		1.758466	0.760659				NA	NA	NA	47.04	1.26	0.24
Q5T5C7	Serine--tRNA ligase, cytoplasmic	SARS	2.50		-0.15686	0.928272				NA	NA	NA	50.81	0.39	0.61		0.039987	1.000253				NA	NA	NA	45.40	0.52	0.47
Q5T697	Guanine nucleotide binding protein (G protein), alpha transducing activity polypeptide 2	GNAT2	12.00		-1.53809	-0.68951				NA	NA	NA	40.43	-1.11	0.23		-2.08697	-0.81879				NA	NA	NA	58.44	-1.45	0.26
Q6IAS8	RAB27A protein	RAB27A	2.50		0.204767	0.362953				NA	NA	NA	7.75	0.28	0.17		0.607917	-0.0325				NA	NA	NA	30.88	0.29	0.53
Q6MZZ3	Putative uncharacterized protein DKFZp686I0746	DKFZp686I0746	2.50		-1.06762	-0.34141				NA	NA	NA	34.86	-0.70	0.30		-0.18953	-0.66162				NA	NA	NA	22.93	-0.43	0.32
Q8TB01	Similar to cytoskeleton-associated protein 4 (Fragment)	NA	14.50		-0.04003	-0.42328				NA	NA	NA	18.67	-0.23	0.44		0.105561	0.089055				NA	NA	NA	0.81	0.10	0.05
P11233	Ras-related protein Ral-A	RALA	2.00		-0.16919	-0.56195				NA	NA	NA	19.13	-0.37	0.31		-0.26273	-0.61088				NA	NA	NA	16.98	-0.44	0.24
Q9H3N1	Thioredoxin-related transmembrane protein 1	TMX1	1.50		-0.99129	-1.80934				NA	NA	NA	39.05	-1.40	0.18		-0.33955	-1.90335				NA	NA	NA	69.93	-1.12	0.39
A0A087X2I2	Glutathione peroxidase	GPX4	1.50		0.568416	-0.3583				NA	NA	NA	43.92	0.11	0.86		0.819112	-0.00051				NA	NA	NA	39.13	0.41	0.50
B4DHE8	RNA-binding protein Musashi homolog 2	MSI2	1.50		-0.14758	-0.06617				NA	NA	NA	3.99	-0.11	0.23		0.455721	0.42009				NA	NA	NA	1.75	0.44	0.03
A8K2G0	cDNA FLJ76605, highly similar to Homo sapiens secretory carrier membrane protein 1 (SCAMP1), transcrip	NA	1.50		0.477405		-0.32853			NA	NA	NA	38.50	0.07	0.88		0.417294		0.206989			NA	NA	NA	10.29	0.31	0.21
A8K5W7	cDNA FLJ75180, highly similar to Homo sapiens mitochondrial isoleucine tRNA synthetase, mRNA	NA	2.00		2.270017		0.53294			NA	NA	NA	76.15	1.40	0.35		1.849392		0.151726			NA	NA	NA	74.77	1.00	0.45
B4DHD2	cDNA FLJ55458, highly similar to Programmed cell death 6-interacting protein	NA	5.50		0.506559		0.59848			NA	NA	NA	4.50	0.55	0.05		-0.2316		0.339535			NA	NA	NA	27.63	0.05	0.88
B4DHK9	cDNA FLJ51656, highly similar to Actin-like protein 2	NA	1.00		1.047336		0.892775			NA	NA	NA	7.57	0.97	0.05		0.825343		1.446643			NA	NA	NA	29.99	1.14	0.17
B4DIV0	cDNA FLJ52546, highly similar to Beta-soluble NSF attachment protein	NA	3.00		-0.46709		-0.04077			NA	NA	NA	20.74	-0.25	0.44		-0.31358		-0.16081			NA	NA	NA	7.48	-0.24	0.20
B4DP80	cDNA FLJ56357, highly similar to Homo sapiens apolipoprotein A-I binding protein (APOA1BP), mRNA	NA	1.50		0.478223		-1.09099			NA	NA	NA	70.13	-0.31	0.76		0.376459		0.225022			NA	NA	NA	7.42	0.30	0.16
E7EQ64	Trypsin-1	PRSS1	2.50		0.99791		0.807679			NA	NA	NA	9.31	0.90	0.07		0.991934		0.752727			NA	NA	NA	11.70	0.87	0.09
J3KQA0	Synaptotagmin I, isoform CRA_b	SYT1	1.50		-1.09777		-0.54261			NA	NA	NA	26.88	-0.82	0.21		-1.48865		-0.46423			NA	NA	NA	48.20	-0.98	0.31
Q59GX9	Ribosomal protein L5 variant (Fragment)	NA	1.50		0.489354		0.916154			NA	NA	NA	20.77	0.70	0.19		0.63044		1.53933			NA	NA	NA	43.13	1.08	0.25
Q8TF11	Vacuolar proton pump subunit SFD alpha isoform	NA	3.50		-0.0927		0.965454			NA	NA	NA	49.66	0.44	0.56		-0.29183		0.314185			NA	NA	NA	29.27	0.01	0.98
Q5JTV8	Torsin-1A-interacting protein 1	TOR1AIP1	1.00		0.256852		0.189119			NA	NA	NA	3.32	0.22	0.10		0.270653		-0.4261			NA	NA	NA	33.50	-0.08	0.86
A0A0B4J2C3	Translationally-controlled tumor protein	TPT1	1.50		0.333424		-0.02862			NA	NA	NA	17.65	0.15	0.55		0.280302		-0.50555			NA	NA	NA	37.59	-0.11	0.82
A0A0C4DGE4	Syntaxin-3 (Fragment)	STX3	5.00		-0.89038		-1.626			NA	NA	NA	35.29	-1.26	0.18		-0.80202		-1.68419			NA	NA	NA	41.94	-1.24	0.22
O15400	Syntaxin-7	STX7	2.00		-0.4917			-0.00343		NA	NA	NA	23.71	-0.25	0.50		0.116782			-0.51395		NA	NA	NA	30.43	-0.20	0.64
Q7Z406	Myosin-14	MYH14	5.50		0.143703			0.929604		NA	NA	NA	37.59	0.54	0.40		0.33653			0.751368		NA	NA	NA	20.19	0.54	0.23
A0A024R4A0	Nucleolin, isoform CRA_b	NCL	12.50		-0.52164			0.079213		NA	NA	NA	29.03	-0.22	0.60		-0.10747			0.003924		NA	NA	NA	5.46	-0.05	0.52
A0A024R8W0	DEAD (Asp-Glu-Ala-Asp) box polypeptide 48, isoform CRA_a	DDX48	3.50		-1.38696			-0.14587		NA	NA	NA	57.34	-0.77	0.43		-1.58916			-0.24998		NA	NA	NA	61.30	-0.92	0.40
A8K7F6	cDNA FLJ78244, highly similar to Homo sapiens eukaryotic translation initiation factor 4A, isoform 1 (EIF4A	NA	5.00		0.855675			1.497786		NA	NA	NA	30.96	1.18	0.17		0.850859			0.466576		NA	NA	NA	18.72	0.66	0.18
B7Z3I9	Delta-aminolevulinic acid dehydratase	NA	2.50		0.581415			-0.58545		NA	NA	NA	54.27	0.00	1.00		-0.09943			-1.4169		NA	NA	NA	60.43	-0.76	0.46
H0Y8X4	2'-deoxynucleoside 5'-phosphate N-hydrolase 1 (Fragment)	DNPH1	2.00		0.95253			0.927474		NA	NA	NA	1.23	0.94	0.01		0.264796			0.99617		NA	NA	NA	35.10	0.63	0.33
Q53HU0	Chaperonin containing TCP1, subunit 8 (Theta) variant (Fragment)	NA	6.50		0.168743			-0.26932		NA	NA	NA	21.31	-0.05	0.86		0.214216			-0.26457		NA	NA	NA	23.25	-0.03	0.93
Q59EG8	Proteasome 26S non-ATPase subunit 2 variant (Fragment)	NA	3.50		-0.22845			-2.67948		NA	NA	NA	97.69	-1.45	0.45		0.067585			-0.64362		NA	NA	NA	34.17	-0.29	0.57
Q09028	Histone-binding protein RBBP4	RBBP4	1.00		0.29943				0.548418	NA	NA	NA	12.17	0.42	0.18		0.192976				0.138408	NA	NA	NA	2.67	0.17	0.10
B2R923	cDNA, FLJ94174	NA	3.50		0.99734				0.166152	NA	NA	NA	39.65	0.58	0.39		1.30691				0.895348	NA	NA	NA	20.04	1.10	0.12
B3KTA3	Fascin	NA	3.00		0.114893				-0.50183	NA	NA	NA	29.78	-0.19	0.64		-0.33538				0.021991	NA	NA	NA	17.43	-0.16	0.54
B4DVZ8	Leukotriene A(4) hydrolase	NA	2.50		-0.75063				0.666651	NA	NA	NA	64.37	-0.04	0.96		-0.78218				0.616481	NA	NA	NA	63.64	-0.08	0.92
Q8TAF6	Acyl-CoA synthetase 4	FACL4	1.50		-0.35554				0.212507	NA	NA	NA	27.49	-0.07	0.84		-0.84412				-0.6334	NA	NA	NA	10.31	-0.74	0.09
Q9BS10	Similar to ribosomal protein S8 (Fragment)	NA	1.50		1.916326				0.081465	NA	NA	NA	79.49	1.00	0.47		2.733932				0.465579	NA	NA	NA	92.80	1.60	0.39
A0A024R1K9	Parvalbumin, isoform CRA_a	PVALB	1.50		-0.25244				-1.37416	NA	NA	NA	52.37	-0.81	0.38		-0.17852				-1.15359	NA	NA	NA	46.05	-0.67	0.40
M0R268	U1 small nuclear ribonucleoprotein A (Fragment)	SNRPA	1.00		1.009177				0.123717	NA	NA	NA	42.09	0.57	0.42		0.603427				-0.45113	NA	NA	NA	49.50	0.08	0.91
A0A024R9D7	2,4-dienoyl CoA reductase 1, mitochondrial, isoform CRA_b	DECR1	1.00			-0.43434		-1.08145		NA	NA	NA	31.20	-0.76	0.26			-1.00127		-0.13018		NA	NA	NA	41.44	-0.57	0.42
E7EQV9	Ribosomal protein L15 (Fragment)	RPL15	1.50			1.004499		0.774238		NA	NA	NA	11.26	0.89	0.08			1.044601		0.232841		NA	NA	NA	38.77	0.64	0.36
I3L0A0	HCG2044781	TMEM189-UBE2V1	1.00			-0.0281		-0.33076		NA	NA	NA	14.78	-0.18	0.45			0.080464		-1.36325		NA	NA	NA	65.39	-0.64	0.54
Q59GU5	Dynein, cytoplasmic, intermediate polypeptide 2 variant (Fragment)	NA	1.00			0.050526		0.29237		NA	NA	NA	11.83	0.17	0.39			-0.161		0.222777		NA	NA	NA	18.70	0.03	0.90
Q9UM02	Prolyl endopeptidase	NA	1.50			-1.84794		-0.11209		NA	NA	NA	76.11	-0.98	0.46			0.210376		0.165115		NA	NA	NA	2.22	0.19	0.08
V9HW33	Epididymis secretory sperm binding protein Li 5a	HEL-S-5a	6.50			0.838334		-0.02219		NA	NA	NA	40.97	0.41	0.52			0.680348		0.027852		NA	NA	NA	31.45	0.35	0.47
V9HVZ0	Epididymis secretory protein Li 91	HEL-S-91	1.50			0.882423		-0.36276		NA	NA	NA	57.50	0.26	0.75			1.157812		0.7636		NA	NA	NA	19.20	0.96	0.13
X5D945	Harvey rat sarcoma viral oncoprotein isoform A (Fragment)	HRAS	1.00			-0.07061		-0.54227		NA	NA	NA	22.91	-0.31	0.42			0.651962		-0.10884		NA	NA	NA	36.45	0.27	0.61
E5RG77	Proline synthase co-transcribed bacterial homolog protein (Fragment)	PROSC	1.50			0.375879		0.74526		NA	NA	NA	18.01	0.56	0.20			1.25644		1.287823		NA	NA	NA	1.54	1.27	0.01
K7EP04	Heat shock protein beta-6	HSPB6	1.00			1.585581		0.536273		NA	NA	NA	49.28	1.06	0.29			0.886999		-0.51513		NA	NA	NA	63.78	0.19	0.83
Q0VDC6	Peptidyl-prolyl cis-trans isomerase	FKBP1A	2.00			1.431771			0.905911	NA	NA	NA	25.49	1.17	0.14			1.802176			0.866633	NA	NA	NA	44.31	1.33	0.21
Q3ZCW5	Succinyl-CoA ligase subunit beta (Fragment)	SUCLG2	1.00			-0.1685			0.087597	NA	NA	NA	12.52	-0.04	0.81			-0.694			0.587565	NA	NA	NA	58.98	-0.05	0.95
Q6MZV5	Putative uncharacterized protein DKFZp686M09245	DKFZp686M09245	1.50			1.419635			0.753883	NA	NA	NA	32.06	1.09	0.19			1.423302			1.249645	NA	NA	NA	8.50	1.34	0.04
Q9UNL6	Hemoglobin gamma-G (Fragment)	HBG2	11.50			-0.97608			-1.6296	NA	NA	NA	31.49	-1.30	0.16			-0.91714			-1.57228	NA	NA	NA	31.57	-1.24	0.16
P58107	Epiplakin	EPPK1	3.50			1.666462			0.882184	NA	NA	NA	37.52	1.27	0.19			2.196365			0.914897	NA	NA	NA	58.98	1.56	0.25
A8K878	Mesencephalic astrocyte-derived neurotrophic factor	MANF	1.00			-0.44008			0.606473	NA	NA	NA	49.16	0.08	0.90			-0.98815			0.070099	NA	NA	NA	49.66	-0.46	0.55
O00231	26S proteasome non-ATPase regulatory subunit 11	PSMD11	3.00				0.408966	0.075238		NA	NA	NA	16.28	0.24	0.38				-0.57904	0.219761		NA	NA	NA	38.18	-0.18	0.73
O00487	26S proteasome non-ATPase regulatory subunit 14	PSMD14	1.50				0.098951	0.660863		NA	NA	NA	27.20	0.38	0.41				0.137572	1.448961		NA	NA	NA	60.19	0.79	0.44
B3KX72	cDNA FLJ44920 fis, clone BRAMY3011501, highly similar to Heterogeneous nuclear ribonucleoprotein U	NA	4.00				-0.37495	-0.24278		NA	NA	NA	6.47	-0.31	0.13				0.270292	-0.27254		NA	NA	NA	26.30	0.00	1.00
B4DVJ0	Glucose-6-phosphate isomerase	NA	14.50				-0.10993	0.105957		NA	NA	NA	10.56	0.00	0.99				-0.47051	-0.17313		NA	NA	NA	14.52	-0.32	0.28
B7Z1V4	cDNA FLJ56034, highly similar to 4-aminobutyrate aminotransferase, mitochondrial (EC 2.6.1.19)	NA	3.00				-1.0386	-0.66989		NA	NA	NA	17.97	-0.85	0.14				-1.50519	-0.65172		NA	NA	NA	40.65	-1.08	0.24
E9PF82	Calcium/calmodulin-dependent protein kinase type II subunit delta	CAMK2D	1.00				1.061042	0.188498		NA	NA	NA	41.51	0.62	0.39				0.977749	-0.46518		NA	NA	NA	65.36	0.26	0.78
E9PRY8	Elongation factor 1-delta	EEF1D	3.50				0.603521	0.895594		NA	NA	NA	14.27	0.75	0.12				0.739638	0.543499		NA	NA	NA	9.60	0.64	0.10

				08/017 hESC-RPE to hRPE																08/023 hESC-RPE to hRPE															
				Analysis						Technical replicate CV			Biological	Fold change (mean)	Unadjusted p-value	Analysis						Technical replicate CV			Biological	Fold change (mean)	Unadjusted p-value
UniProt	Protein name	Gene name	Peptide mean	1.1	1.2	2.1	2.2	3.1	3.2	1	2	3	CV		(t-test)	1.1	1.2	2.1	2.2	3.1	3.2	1	2	3	CV		(t-test)
F6TLX2	Glyoxalase domain-containing protein 4	GLOD4	2.50				1.012211	0.466462		NA	NA	NA	26.43	0.74	0.23				0.450249	-0.10929		NA	NA	NA	27.09	0.17	0.65
Q53HV6	Niemann-Pick disease, type C2 variant (Fragment)	NA	3.50				1.134689	0.92909		NA	NA	NA	10.06	1.03	0.06				1.236955	0.900383		NA	NA	NA	16.42	1.07	0.10
V9HWJ1	Glutathione synthetase	GSS	2.50				0.52215	0.395652		NA	NA	NA	6.20	0.46	0.09				0.569656	0.361053		NA	NA	NA	10.21	0.47	0.14
P30711	Glutathione S-transferase theta-1	GSTT1	1.00				0.681697	0.671259		NA	NA	NA	0.51	0.68	0.00				0.979925	1.955803		NA	NA	NA	46.09	1.47	0.20
Q13509	Tubulin beta-3 chain	TUBB3	31.50				-0.61991		-3.02394	NA	NA	NA	96.47	-1.82	0.37				0.014563		-1.02909	NA	NA	NA	49.03	-0.51	0.51
Q14315	Filamin-C	FLNC	5.50				-0.06815		0.737786	NA	NA	NA	38.50	0.33	0.56				0.450249		0.636313	NA	NA	NA	9.11	0.54	0.11
Q3ZCW2	Galectin-related protein	LGALSL	1.00				-2.06483		-1.32023	NA	NA	NA	35.71	-1.69	0.14				-1.45937		-0.93153	NA	NA	NA	25.59	-1.20	0.14
A0A088QF11	Choline transporter-like protein 2 isoform 2	SLC44A2	2.00				0.150926		-0.26399	NA	NA	NA	20.20	-0.06	0.83				0.421898		-0.09045	NA	NA	NA	24.85	0.17	0.63
B2RA03	cDNA, FLJ94640, highly similar to Homo sapiens keratin 18 (KRT18), mRNA	NA	9.50				1.59447		1.263141	NA	NA	NA	16.17	1.43	0.07				2.047228		1.128552	NA	NA	NA	43.56	1.59	0.18
B4DDM5	cDNA FLJ53298, highly similar to Peroxisomal multifunctional enzyme type 2	NA	6.00				0.289811		0.238256	NA	NA	NA	2.53	0.26	0.06				0.043254		0.234893	NA	NA	NA	9.38	0.14	0.38
B4DWX6	cDNA FLJ53936, highly similar to Medium-chain specific acyl-CoA dehydrogenase, mitochondrial (EC 1.3.99	NA	7.00				-0.45147		-0.09779	NA	NA	NA	17.25	-0.27	0.36				-0.4049		-0.14646	NA	NA	NA	12.63	-0.28	0.28
M0R3D6	60S ribosomal protein L18a (Fragment)	RPL18A	1.00				1.449878		-0.08065	NA	NA	NA	68.69	0.68	0.54				0.966041		0.663061	NA	NA	NA	14.80	0.81	0.12
Q53GW1	Vesicle transport-related protein isoform a variant (Fragment)	NA	1.00				-1.84339		0.552086	NA	NA	NA	96.25	-0.65	0.69				-0.5704		0.470995	NA	NA	NA	48.94	-0.05	0.94
Q5T0G8	Annexin	ANXA11	4.50				-0.4261		1.29344	NA	NA	NA	75.54	0.43	0.70				-0.32275		1.464881	NA	NA	NA	77.90	0.57	0.64
P14136	Glial fibrillary acidic protein	GFAP	12.50				-1.46451		-0.50684	NA	NA	NA	45.29	-0.99	0.29				-1.62929		-0.09479	NA	NA	NA	68.84	-0.86	0.46

Proteins discarded based on CV>100, n=27

				08/017 hESC-RPE to hRPE																08/023 hESC-RPE to hRPE															
				Analysis						Technical replicate CV			Biological	Fold change (mean)	Unadjusted p-value	Analysis						Technical replicate CV			Biological	Fold change (mean)	Unadjusted p-value
UniProt	Protein name	Gene name		1.1	1.2	2.1	2.2	3.1	3.2	1	2	3	CV		(t-test)	1.1	1.2	2.1	2.2	3.1	3.2	1	2	3	CV		(t-test)
B4DQY1	cDNA FLJ56133, highly similar to Serine/threonine-protein phosphatase 2A 65 kDa regulatory subunit A alp	NA	8.00	-0.3918	2.257805	-0.29775	-1.57484	-1.09601	-0.57069	102.54	58.80	25.47	97.26	-0.28	0.69	-0.51545	1.624378	-0.48512	-2.29245	-0.85588	-0.41016	89.11	78.57	21.67	77.62	-0.49	0.48
Q53GF5	Proteasome subunit alpha type (Fragment)	NA	2.00	0.147863	2.568128	-0.21605	5.627716	-0.85977	-2.77438	96.90	136.58	82.13	139.54	0.75	0.63	0.279953	2.280166	1.103141	4.498103	-0.47434	-1.01388	84.86	116.87	26.14	116.35	1.11	0.39
Q53XL8	Proteasome (Prosome, macropain) 26S subunit, ATPase, 1	PSMC1	2.83	-0.7848	-0.11124	0.90071	-0.68624	-0.75556	0.115652	32.43	70.78	41.45	27.78	-0.22	0.32	-1.42089	1.466605	0.398366	0.926756	-0.12183	1.040636	107.75	25.61	54.08	3.97	0.38	0.18
E7EPM6	Long-chain-fatty-acid--CoA ligase 1	ACSL1	1.83	1.01994	1.104866	1.388994	0.088328	0.033507	-1.73762	4.16	59.76	77.33	49.83	0.32	0.65	0.89551	1.024472	1.776479	-1.05007	0.444257	0.294413	6.32	106.48	7.34	21.92	0.56	0.10
Q53FS4	Lectin, mannose-binding, 1 variant (Fragment)	NA	1.40	-0.61178	2.077063	-1.10412	-0.51184	0.345705		103.45	28.63	NA	65.52	0.04	0.86	-0.80977	2.314549	-1.43124	-0.24055	-0.01058		112.32	55.26	NA	79.19	-0.04	0.95
A0A0A6YYA0	Protein TMED7-TICAM2	TMED7-TICAM2	3.60	-0.3498	0.394918	-0.34275	-0.51071		-0.83991	35.71	8.22	NA	31.60	-0.33	0.24	-2.99126	0.216114	-0.36815	-1.78756		-0.46791	113.79	64.45	NA	15.14	-1.08	0.07
A4QPB0	IQ motif containing GTPase activating protein 1	IQGAP1	4.00	1.985856	-0.75415	-0.24245	0.435135		1.423423	104.60	32.61	NA	40.74	0.57	0.21	1.128331	0.320561	-0.13243	0.461472		2.068721	38.59	28.70	NA	68.92	0.77	0.22
K7ELC2	40S ribosomal protein S15	RPS15	3.00	0.04286	1.45646	-0.29905	-0.42894		-0.21708	64.23	6.36	NA	52.60	0.11	0.89	-0.92486	1.735819	-0.01808	0.330533		-0.09845	102.80	17.00	NA	39.77	0.20	0.40
V9HWH6	Purine nucleoside phosphorylase	PNP	2.00	0.143415	0	-1.5956		0.019095	-3.51167	7.02	NA	118.90	57.37	-0.99	0.20	0.064492	0.369483	-5.83314		-0.09001	-2.32589	14.89	NA	91.89	98.38	-1.56	0.34
P35527	Keratin, type I cytoskeletal 9	KRT9	2.00	2.635288	1.562258		0.719331	0.178157	-0.58758	50.29	NA	36.67	81.97	0.90	0.32	2.365015	-0.19659		0.600774	0.556493	0.03396	100.45	NA	25.33	49.38	0.67	0.10
A0A024R324	Ras homolog gene family, member A, isoform CRA_a	RHOA	4.60	0.00452	0.411712		-0.47107	1.275839	-2.25874	19.83	NA	118.95	28.94	-0.21	0.39	0.254096	0.823228		-0.32212	0.892719	-0.63473	27.54	NA	68.58	29.43	0.20	0.69
Q6IAV5	Succinyl-CoA:3-ketoacid-coenzyme A transferase	OXCT	7.40		-0.23313	-0.00504	-0.47547	3.211121	0.244592	NA	22.85	109.34	109.16	0.55	0.59		-0.15355	-0.13743	-0.29803	2.577915	0.138214	NA	7.86	97.40	86.86	0.43	0.59
P02768	Serum albumin	ALB	87.50	3.898454	3.235203	1.032887	0.830837			31.95	9.89	NA	103.00	2.25	0.34	3.635735	3.125267	0.830628	0.912214			24.76	4.00	NA	99.70	2.13	0.34
E7EV99	Alpha-adducin	ADD1	2.25	-0.62812	2.092991		0.028328	-1.52105		104.17	NA	NA	84.47	-0.01	0.74	-0.32472	2.223352		-1.52467	-1.15184		100.12	NA	NA	114.61	-0.19	0.53
P61266	Syntaxin-1B	STX1B	1.50	-1.37588	1.619351			0.436303	-0.80196	109.90	NA	57.22	40.21	-0.03	0.87	-2.28067	1.030957			0.723838	-1.45494	115.54	NA	90.25	7.72	-0.50	0.16
Q8N111	Cell cycle exit and neuronal differentiation protein 1	CEND1	1.00	-1.8046	-1.48824			-1.8195	-2.42108	15.44	NA	29.07	21.95	-1.88	0.08	-4.97739	-0.47235			-1.43691	-1.69376	129.49	NA	12.56	7.32	-2.15	0.17
Q96DI8	Heme oxygenase (Decycling) 1	HMOX1	2.50		0.578621	-0.01118		2.493795	-0.25166	NA	NA	104.72	61.75	0.70	0.23		0.682308	-0.39651		2.702681	-0.60512	NA	NA	115.48	73.11	0.60	0.41
O14980	Exportin-1	XPO1	4.00			0.387273	-0.52924	2.540652	3.117282	NA	43.47	27.89	107.12	1.38	0.52			-0.67085	-0.65211	2.322879	3.765147	NA	0.92	65.34	123.42	1.19	0.64
P35908	Keratin, type II cytoskeletal 2 epidermal	KRT2	2.00	2.308281	2.330105	0.160144				1.07	NA	NA	89.68	1.60	0.46	2.439655	2.923062	-0.18986				23.47	NA	NA	107.83	1.72	0.55
A0A024R1Q5	N-acetylgalactosaminidase, alpha-, isoform CRA_a	NAGA	1.00	-1.80804	1.935708	-0.4304				121.77	NA	NA	66.40	-0.10	0.59	-1.96365	0.733568	-0.42206				103.64	NA	NA	17.67	-0.55	0.12
B4DM33	cDNA FLJ52068, highly similar to Microtubule-associated protein RP/EB family member 1	NA	1.33	-0.1978	1.103983	1.793544				59.80	NA	NA	55.58	0.90	0.34	-0.19892	0.790605	3.052157				46.68	NA	NA	103.09	1.21	0.44
Q53HS0	Glutaminyl-tRNA synthetase variant (Fragment)	NA	2.00	1.116794		-0.68653			2.369991	NA	NA	NA	87.16	0.93	0.40	-1.92986		-0.22907			2.17305	NA	NA	NA	122.69	0.00	1.00
E5RFU2	Bifunctional epoxide hydrolase 2	EPHX2	3.00	-1.12087		1.19344				NA	NA	NA	94.07	0.04	0.98	-2.55032		0.491488				NA	NA	NA	110.79	-1.03	0.62
Q13526	Peptidyl-prolyl cis-trans isomerase NIMA-interacting 1	PIN1	1.00	2.227067				-0.33358		NA	NA	NA	100.43	0.95	0.59	0.972458				0.094827		NA	NA	NA	41.74	0.53	0.44
A0A024R4A4	RAB17, member RAS oncogene family, isoform CRA_a	RAB17	1.00		-2.22379	-1.52907				NA	NA	NA	33.41	-1.88	0.12		-3.97618	-1.05441				NA	NA	NA	108.45	-2.52	0.33
B2R5V9	cDNA, FLJ92652, highly similar to Homo sapiens high density lipoprotein binding protein (vigilin)(HDLBP), n	NA	2.50			1.43295		-1.23882		NA	NA	NA	103.05	0.10	0.95			0.744588		-1.27904		NA	NA	NA	85.59	-0.27	0.84
O43823	A-kinase anchor protein 8	AKAP8	1.00			1.721992		-2.87995		NA	NA	NA	130.23	-0.58	0.84			2.395387		-1.17734		NA	NA	NA	119.49	0.61	0.79

Proteins filtered out based on detection in only one biological replicates (one analysis run), n=661

				08/017 hESC-RPE to hRPE																08/023 hESC-RPE to hRPE															
				Analysis						Technical replicate CV			Biological	Fold change (mean)	Unadjusted p-value	Analysis						Technical replicate CV			Biological	Fold change (mean)	Unadjusted p-value
UniProt	Protein name	Gene name		1.1	1.2	2.1	2.2	3.1	3.2	1	2	3	CV		(t-test)	1.1	1.2	2.1	2.2	3.1	3.2	1	2	3	CV		(t-test)
O43490	Prominin-1	PROM1	3.00	-0.48295	-0.7654					13.80	NA	NA	NA	-0.62	NA	-0.54904	-0.6851					6.66	NA	NA	NA	-0.62	NA
O75489	NADH dehydrogenase [ubiquinone] iron-sulfur protein 3, mitochondrial	NDUFS3	7.00	0.643281	0.238143					19.73	NA	NA	NA	0.44	NA	0.707569	0.072413					30.64	NA	NA	NA	0.39	NA
O94919	Endonuclease domain-containing 1 protein	ENDOD1	1.00	0.049343	0.079401					1.47	NA	NA	NA	0.06	NA	-0.4312	-0.83466					19.65	NA	NA	NA	-0.63	NA
P13473	Lysosome-associated membrane glycoprotein 2	LAMP2	4.50	0.262411	0.04467					10.65	NA	NA	NA	0.15	NA	0.147152	-0.12491					13.30	NA	NA	NA	0.01	NA
Q13151	Heterogeneous nuclear ribonucleoprotein A0	HNRNPA0	2.00	-1.00809	-0.62763					18.54	NA	NA	NA	-0.82	NA	-0.87418	-0.70077					8.49	NA	NA	NA	-0.79	NA
Q13432	Protein unc-119 homolog A	UNC119	1.50	-0.69156	0.239269					44.10	NA	NA	NA	-0.23	NA	-0.7426	0.013161					36.22	NA	NA	NA	-0.36	NA
Q8IY95	Transmembrane protein 192	TMEM192	2.00	-0.12601	0.726602					40.61	NA	NA	NA	0.30	NA	-0.41416	-0.58617					8.42	NA	NA	NA	-0.50	NA
Q9GZP4	PITH domain-containing protein 1	PITHD1	1.00	-1.67953	-0.32217					62.02	NA	NA	NA	-1.00	NA	-1.11442	-0.68651					20.82	NA	NA	NA	-0.90	NA
Q9Y4W6	AFG3-like protein 2	AFG3L2	3.00	1.355741	-0.17026					68.52	NA	NA	NA	0.59	NA	-0.02008	-0.36686					16.92	NA	NA	NA	-0.19	NA
A0A024R216	Hepatoma-derived growth factor, related protein 3, isoform CRA_a	HDGFRP3	2.50	-1.66074	-0.33935					60.59	NA	NA	NA	-1.00	NA	-1.14365	-0.14719					46.99	NA	NA	NA	-0.65	NA
A0A024R6D4	Enhancer of rudimentary homolog	ERH	2.00	-0.43293	0.081196					24.94	NA	NA	NA	-0.18	NA	-0.09674	-0.03366					3.09	NA	NA	NA	-0.07	NA
A8K1D2	cDNA FLJ76601, highly similar to Homo sapiens LIM and SH3 protein 1 (LASP1), mRNA	NA	1.00	1.652077	2.534317					41.94	NA	NA	NA	2.09	NA	1.421978	1.752462					16.13	NA	NA	NA	1.59	NA
B2R5W6	cDNA, FLJ92661, highly similar to Homo sapiens microtubule-associated protein, RP/EB family, member 3 (	NA	1.50	-0.36849	0.121713					23.80	NA	NA	NA	-0.12	NA	-1.67842	-0.86043					39.05	NA	NA	NA	-1.27	NA
B4DT01	cDNA FLJ60347, highly similar to Homo sapiens hydroxyacylglutathione hydrolase (HAGH), transcript variar	NA	2.50	1.2767	2.118788					40.14	NA	NA	NA	1.70	NA	1.478744	3.290045					78.70	NA	NA	NA	2.38	NA
B4DWI8	cDNA FLJ57805, highly similar to Homo sapiens paraspeckle component 1 (PSPC1), transcript variant alpha,	NA	1.50	-0.34255	-1.24433					42.81	NA	NA	NA	-0.79	NA	-0.4095	-0.68226					13.33	NA	NA	NA	-0.55	NA
B4DX99	Malic enzyme	NA	4.50	-2.28481	-1.53094					36.13	NA	NA	NA	-1.91	NA	-0.86218	-1.03502					8.46	NA	NA	NA	-0.95	NA
B7Z795	Carboxylic ester hydrolase	NA	2.00	-0.0515	-0.08423					1.60	NA	NA	NA	-0.07	NA	-1.2248	0.124282					61.69	NA	NA	NA	-0.55	NA
D6RB01	Soluble lamin-associated protein of 75 kDa	FAM169A	3.50	0.043815	0.581415					26.05	NA	NA	NA	0.31	NA	0.092096	-0.5454					30.75	NA	NA	NA	-0.23	NA
E7DVW5	Fatty acid binding protein 5 (Psoriasis-associated)	FABP5	2.00	-0.06812	-0.38226					15.34	NA	NA	NA	-0.23	NA	-0.39185	-0.4409					2.40	NA	NA	NA	-0.42	NA
E9PGC8	Microtubule-associated protein 1A	MAP1A	3.00	-0.16609	-0.758					28.61	NA	NA	NA	-0.46	NA	-0.4753	-0.26325					10.37	NA	NA	NA	-0.37	NA
H3BLU2	Limbic system-associated membrane protein (Fragment)	LSAMP	1.00	-1.78006	-2.13606					17.36	NA	NA	NA	-1.96	NA	-1.75994	-2.06007					14.66	NA	NA	NA	-1.91	NA
J3KTA4	Probable ATP-dependent RNA helicase DDX5	DDX5	2.50	0.151944	1.626193					66.56	NA	NA	NA	0.89	NA	0.221954	1.183596					45.46	NA	NA	NA	0.70	NA
N0E472	Casein kinase II subunit beta	CSNK2B-LY6G5B--991	1.00	-0.37843	-0.95957					28.10	NA	NA	NA	-0.67	NA	-0.67877	0.169852					40.43	NA	NA	NA	-0.25	NA
Q13344	Fus-like protein (Fragment)	NA	3.00	-0.06194	-0.04963					0.60	NA	NA	NA	-0.06	NA	0.080123	-0.02422					5.11	NA	NA	NA	0.03	NA
Q4L233	Brain-specific protein p25 alpha, isoform CRA_b	FREP1	2.50	-3.71012	-3.45299					12.57	NA	NA	NA	-3.58	NA	-2.90011	-2.48063					20.42	NA	NA	NA	-2.69	NA
Q4LE64	NUMA1 variant protein (Fragment)	NUMA1	1.00	4.722521	-0.69949					134.97	NA	NA	NA	2.01	NA	4.711948	-0.92006					135.83	NA	NA	NA	1.90	NA
Q53QE9	Putative uncharacterized protein UGP2 (Fragment)	UGP2	2.50	0.006285	0.491921					23.58	NA	NA	NA	0.25	NA	0.267082	0.192397					3.66	NA	NA	NA	0.23	NA
Q53ZP9	Heat shock protein apg-1	NA	4.00	0.377345	-1.5806					83.52	NA	NA	NA	-0.60	NA	-0.78011	-0.61694					7.99	NA	NA	NA	-0.70	NA
Q5HYB6	Epididymis luminal protein 189	DKFZp686J1372	6.00	1.169991	1.044118					6.17	NA	NA	NA	1.11	NA	1.211678	1.464691					12.37	NA	NA	NA	1.34	NA
Q5MJP6	Poly(RC) binding protein 3	PCBP3	4.50	0.205335	-0.39993					29.24	NA	NA	NA	-0.10	NA	-0.13617	-0.11915					0.83	NA	NA	NA	-0.13	NA
Q6IB58	FLOT1 protein	FLOT1	1.00	0.29342	-0.03089					15.83	NA	NA	NA	0.13	NA	0.690834	0.775623					4.15	NA	NA	NA	0.73	NA
Q6LES8	TFAM protein (Fragment)	TFAM	2.50	-0.95066	-0.01297					44.41	NA	NA	NA	-0.48	NA	0.347469	-0.91756					58.31	NA	NA	NA	-0.29	NA

				08/017 hESC-RPE to hRPE												08/023 hESC-RPE to hRPE											
				Analysis						Technical replicate CV			Biological	Fold change (mean)	Unadjusted p-value	Analysis						Technical replicate CV			Biological	Fold change (mean)	Unadjusted p-value
UniProt	Protein name	Gene name	Peptide mean	1.1	1.2	2.1	2.2	3.1	3.2	1	2	3	CV		(t-test)	1.1	1.2	2.1	2.2	3.1	3.2	1	2	3	CV		(t-test)
Q7Z3H5	Mitogen-activated protein kinase (Fragment)	DKFZp686O0215	2.50	0.234574	-0.1539					18.93	NA	NA	NA	0.04	NA	0.061556	-0.39266					22.08	NA	NA	NA	-0.17	NA
Q8J014	Ribosomal protein S2	rps2	5.00	-0.24554	-0.09129					7.55	NA	NA	NA	-0.17	NA	-0.21587	0.553102					36.82	NA	NA	NA	0.17	NA
S4R3Z2	Aldo-keto reductase family 1 member C3	AKR1C3	1.50	-0.26079	-0.05396					10.12	NA	NA	NA	-0.16	NA	-0.44963	-0.01284					21.25	NA	NA	NA	-0.23	NA
X6R700	Chromatin target of PRMT1 protein	CHTOP	1.00	-0.26599	-0.01392					12.32	NA	NA	NA	-0.14	NA	-1.83378	-0.13697					74.74	NA	NA	NA	-0.99	NA
P22676	Calretinin	CALB2	3.00	-0.02682	-0.05199					1.23	NA	NA	NA	-0.04	NA	-0.14003	0.362129					24.37	NA	NA	NA	0.11	NA
Q99729	Heterogeneous nuclear ribonucleoprotein A/B	HNRNPAB	3.00	-0.71141	-1.67322					45.47	NA	NA	NA	-1.19	NA	-0.63116	-1.24721					29.74	NA	NA	NA	-0.94	NA
A0A024RD08	Mitochondrial carrier homolog 1 (C. elegans), isoform CRA_d	MTCH1	1.00	-1.0642	0.101509					54.22	NA	NA	NA	-0.48	NA	-0.95768	0.283384					57.34	NA	NA	NA	-0.34	NA
E7EQT4	Apoptotic chromatin condensation inducer in the nucleus	ACIN1	1.50	0.652077	-0.0762					34.96	NA	NA	NA	0.29	NA	0.828239	-0.80934					72.63	NA	NA	NA	0.01	NA
F5H760	Microsomal glutathione S-transferase 1 (Fragment)	MGST1	1.00	1.847866	1.934864					4.26	NA	NA	NA	1.89	NA	1.921888	1.779544					6.97	NA	NA	NA	1.85	NA
Q3B790	High-mobility group nucleosome binding domain 1	HMGN1	1.00	-3.14243	-5.88056					104.55	NA	NA	NA	-4.51	NA	-3.81438	-3.21309					29.05	NA	NA	NA	-3.51	NA
O75880	Protein SCO1 homolog, mitochondrial	SCO1	1.00			0.092894	-0.78862			NA	41.91	NA	NA	-0.35	NA			0.06893	-1.95454			NA	85.59	NA	NA	-0.94	NA
O95168	NADH dehydrogenase [ubiquinone] 1 beta subcomplex subunit 4	NDUFB4	2.00			0.355721	-0.19972			NA	26.89	NA	NA	0.08	NA			0.050478	-1.31551			NA	62.36	NA	NA	-0.63	NA
O95372	Acyl-protein thioesterase 2	LYPLA2	2.50			0.347412	1.383425			NA	48.70	NA	NA	0.87	NA			0.614932	1.169224			NA	26.84	NA	NA	0.89	NA
Q15056	Eukaryotic translation initiation factor 4H	EIF4H	1.00			-0.25	-0.12106			NA	6.32	NA	NA	-0.19	NA			0.512927	0.249428			NA	12.88	NA	NA	0.38	NA
Q6P587	Acylpyruvase FAHD1, mitochondrial	FAHD1	1.50			-1.13581	-0.57799			NA	27.00	NA	NA	-0.86	NA			-0.97466	-0.83764			NA	6.71	NA	NA	-0.91	NA
Q8IXI1	Mitochondrial Rho GTPase 2	RHOT2	2.50			-0.37058	-0.40222			NA	1.55	NA	NA	-0.39	NA			-1.03354	-0.42237			NA	29.51	NA	NA	-0.73	NA
Q9BRR6	ADP-dependent glucokinase	ADPGK	2.50			-0.43577	0.33461			NA	36.89	NA	NA	-0.05	NA			-0.27488	0.936678			NA	56.12	NA	NA	0.33	NA
Q9HBL7	Plasminogen receptor (KT)	PLGRKT	1.00			-1.87978	-0.10198			NA	77.56	NA	NA	-0.99	NA			-1.66402	-0.53122			NA	52.83	NA	NA	-1.10	NA
Q9NRZ7	1-acyl-sn-glycerol-3-phosphate acyltransferase gamma	AGPAT3	2.00			-0.54329	-0.59581			NA	2.57	NA	NA	-0.57	NA			-1.01859	-1.32559			NA	14.99	NA	NA	-1.17	NA
Q9Y305	Acyl-coenzyme A thioesterase 9, mitochondrial	ACOT9	3.00			-0.54214	0.717248			NA	58.08	NA	NA	0.09	NA			-0.75206	-0.41563			NA	16.42	NA	NA	-0.58	NA
Q9Y678	Coatomer subunit gamma-1	COPG1	2.50			1.095997	1.589923			NA	23.98	NA	NA	1.34	NA			0.552288	1.683943			NA	52.79	NA	NA	1.12	NA
A0A024R5Y0	Guanine nucleotide binding protein (G protein), beta 5, isoform CRA_c	GNB5	2.50			-1.94666	-1.57484			NA	18.12	NA	NA	-1.76	NA			-1.68828	-1.79367			NA	5.16	NA	NA	-1.74	NA
A0A024RBX9	Pyruvate dehydrogenase E1 component subunit alpha	PDHA1	6.50			-0.2099	-0.74202			NA	25.79	NA	NA	-0.48	NA			-0.73595	-0.59154			NA	7.07	NA	NA	-0.66	NA
B2R5I8	cDNA, FLJ92490, highly similar to Homo sapiens RAB32, member RAS oncogene family (RAB32), mRNA	NA	1.00			0.709241	0.290026			NA	20.40	NA	NA	0.50	NA			1.093805	0.318037			NA	37.13	NA	NA	0.71	NA
B2RAW8	cDNA, FLJ95164, highly similar to Homo sapiens SEC14-like 2 (S. cerevisiae) (SEC14L2), mRNA	NA	1.00			0.881128	0.741261			NA	6.85	NA	NA	0.81	NA			1.128596	1.036258			NA	4.52	NA	NA	1.08	NA
B3KQS1	cDNA PSEC0102 fis, clone NT2RP2003127, highly similar to Prominin-1	NA	4.50			-1.02271	-1.01124			NA	0.56	NA	NA	-1.02	NA			-1.33681	-1.00177			NA	16.35	NA	NA	-1.17	NA
B4DPX0	Lon protease homolog	NA	5.50			0	-1.03104			NA	48.49	NA	NA	-0.52	NA			-0.36358	-0.64539			NA	13.77	NA	NA	-0.50	NA
B4DWV5	GrpE protein homolog	NA	1.50			-0.90041	-0.64319			NA	12.57	NA	NA	-0.77	NA			-1.4581	-0.77455			NA	32.89	NA	NA	-1.12	NA
B4E3E6	cDNA FLJ58832, highly similar to Heterogeneous nuclear ribonucleoprotein A3	NA	3.00			0.351573	0.377451			NA	1.27	NA	NA	0.36	NA			0.505638	0.511011			NA	0.26	NA	NA	0.51	NA
B5BU72	Phosphatidylinositol-binding clathrin assembly protein isoform 2	PICALM	2.00			-0.06756	0.418268			NA	23.59	NA	NA	0.18	NA			0.164149	0.61962			NA	22.14	NA	NA	0.39	NA
B7Z4P9	cDNA FLJ51678, highly similar to Ras-related protein Rab-18	NA	1.50			-0.59801	-0.88611			NA	14.07	NA	NA	-0.74	NA			-0.98015	-1.24922			NA	13.15	NA	NA	-1.11	NA
B7Z8X5	cDNA FLJ61541, highly similar to Homo sapiens PDZ and LIM domain 5 (PDLIM5), transcript variant 2, mRN	NA	1.00			0.870004	-0.05467			NA	43.83	NA	NA	0.41	NA			1.393173	0.298472			NA	51.22	NA	NA	0.85	NA
D3DSM5	Collagen, type XVIII, alpha 1, isoform CRA_b	COL18A1	2.00			-0.18709	-0.85383			NA	32.11	NA	NA	-0.52	NA			0.269515	-0.50233			NA	36.95	NA	NA	-0.12	NA
F1T0G5	Guanine nucleotide binding protein (G protein), beta polypeptide 3	GNB3	6.00			-0.07201	-0.58193			NA	24.74	NA	NA	-0.33	NA			-0.49472	0.311748			NA	38.53	NA	NA	-0.09	NA
F5GYQ1	V-type proton ATPase subunit d 1	ATP6V0D1	1.50			-0.03895	-0.74379			NA	33.87	NA	NA	-0.39	NA			-0.95835	-0.10981			NA	40.43	NA	NA	-0.53	NA
I6L975	Hydroxysteroid dehydrogenase like 1	HSDL1	2.00			-0.30821	-0.27428			NA	1.66	NA	NA	-0.29	NA			-1.09114	-0.89092			NA	9.80	NA	NA	-0.99	NA
J3QT77	Serum paraoxonase/arylesterase 2	PON2	1.50			0.264227	-1.37154			NA	72.56	NA	NA	-0.55	NA			0.159172	-0.93608			NA	51.24	NA	NA	-0.39	NA
K7EPR0	Transmembrane protein 205	TMEM205	1.00			0.378532	1.194082			NA	38.94	NA	NA	0.79	NA			0.196751	1.005667			NA	38.64	NA	NA	0.60	NA
Q53FE8	cDNA FLJ36526 fis, clone TRACH2003347, highly similar to NSFL1 cofactor p47 (Fragment)	NA	10.00			1.349078	1.172724			NA	8.63	NA	NA	1.26	NA			1.501622	1.630012			NA	6.29	NA	NA	1.57	NA
Q547S8	Electron transfer flavoprotein ubiquinone oxidoreductase	ETFDH	2.00			1.091775	0.434354			NA	31.68	NA	NA	0.76	NA			0.560719	0.187399			NA	18.20	NA	NA	0.37	NA
Q5EK51	Lactoferrin	NA	4.00			1.782261	1.334923			NA	21.75	NA	NA	1.56	NA			1.434393	1.055448			NA	18.47	NA	NA	1.24	NA
Q5JPB8	Putative uncharacterized protein DKFZp686O1117	DKFZp686O1117	1.50			1.490775	1.428784			NA	3.04	NA	NA	1.46	NA			1.099131	0.549625			NA	26.61	NA	NA	0.82	NA
Q5T6H7	Xaa-Pro aminopeptidase 1	XPNPEP1	2.00			0.353648	0.834981			NA	23.38	NA	NA	0.59	NA			-0.1104	0.579991			NA	33.21	NA	NA	0.23	NA
Q6FG42	NDUFA7 protein (Fragment)	NDUFA7	2.50			-0.89449	-0.94732			NA	2.59	NA	NA	-0.92	NA			-0.79313	-0.28998			NA	24.41	NA	NA	-0.54	NA
Q6PK82	AP3D1 protein (Fragment)	AP3D1	1.00			1.230172	0.738261			NA	23.88	NA	NA	0.98	NA			0.918669	-0.94723			NA	80.53	NA	NA	-0.01	NA
Q6ZSA3	cDNA FLJ45695 fis, clone FEBRA2013570, highly similar to 2-oxoisovalerate dehydrogenase alpha subunit, r	NA	1.00			-0.39582	-0.25905			NA	6.70	NA	NA	-0.33	NA			-0.6374	-0.2055			NA	21.01	NA	NA	-0.42	NA
Q7Z4Z1	Epsilon-COP	NA	1.00			-0.79134	0.822364			NA	71.76	NA	NA	0.02	NA			-0.05047	-0.42949			NA	18.47	NA	NA	-0.24	NA
P08133	Annexin A6	ANXA6	42.00			-1.44729	-1.23683			NA	10.30	NA	NA	-1.34	NA			-1.53669	-1.14677			NA	19.00	NA	NA	-1.34	NA
Q92542	Nicastrin	NCSTN	1.50			-0.23745	-0.94225			NA	33.87	NA	NA	-0.59	NA			-0.34965	-0.5942			NA	11.96	NA	NA	-0.47	NA
A0A087WUI2	Heterogeneous nuclear ribonucleoproteins A2/B1	HNRNPA2B1	7.50			0.117291	0.190737			NA	3.60	NA	NA	0.15	NA			0.687122	0.478959			NA	10.18	NA	NA	0.58	NA
E7EQB8	Isocitrate dehydrogenase [NAD] subunit, mitochondrial	IDH3G	1.00			0.195525	-0.04702			NA	11.86	NA	NA	0.07	NA			-0.31272	-0.26036			NA	2.57	NA	NA	-0.29	NA
E9PCG9	D-beta-hydroxybutyrate dehydrogenase, mitochondrial	BDH1	1.50			-1.33204	-1.31011			NA	1.07	NA	NA	-1.32	NA			-2.33489	-1.38565			NA	44.92	NA	NA	-1.86	NA
E9PD91	Regulator of G-protein-signaling 9	RGS9	3.50			-1.44224	-0.87737			NA	27.34	NA	NA	-1.16	NA			-1.33425	-1.77974			NA	21.66	NA	NA	-1.56	NA
H0YMZ1	Proteasome subunit alpha type (Fragment)	PSMA4	3.50			1.708917	0.206359			NA	67.64	NA	NA	0.96	NA			0.101373	-0.45072			NA	26.73	NA	NA	-0.17	NA
O43347	RNA-binding protein Musashi homolog 1	MSI1	1.00					-1.26411	-1.1313	NA	NA	6.50	NA	-1.20	NA					-0.66576	-2.35956	NA	NA	74.64	NA	-1.51	NA
P62304	Small nuclear ribonucleoprotein E	SNRPE	3.00					-0.0896	-0.13037	NA	NA	2.00	NA	-0.11	NA					-0.1606	-0.45143	NA	NA	14.21	NA	-0.31	NA
Q96C86	m7GpppX diphosphatase	DCPS	1.00					1.709375	0.597254	NA	NA	51.96	NA	1.15	NA					1.217807	0.469604	NA	NA	35.87	NA	0.84	NA
Q9Y426	C2 domain-containing protein 2	C2CD2	1.00					0.368515	2.097934	NA	NA	75.89	NA	1.23	NA					-0.01601	1.28434	NA	NA	59.74	NA	0.63	NA
A0A024R462	Fibronectin 1, isoform CRA_n	FN1	4.50					0.286537	0.204311	NA	NA	4.03	NA	0.25	NA					1.544988	1.347204	NA	NA	9.68	NA	1.45	NA
A8K5U9	cDNA FLJ75056, highly similar to Homo sapiens phosphatidylinositol binding clathrin assembly protein (PIC	NA	1.50					-0.06527	0.920694	NA	NA	46.53	NA	0.43	NA					-0.27304	0.534158	NA	NA	38.56	NA	0.13	NA
B2R4A2	Cytochrome b-c1 complex subunit 7	NA	2.00					-0.48148	-0.36412	NA	NA	5.75	NA	-0.42	NA					-0.42409	-1.22836	NA	NA	38.43	NA	-0.83	NA
B2RBS8	cDNA, FLJ95666, highly similar to Homo sapiens albumin (ALB), mRNA	NA	79.00					1.804447	1.399943	NA	NA	19.70	NA	1.60	NA					0.943276	1.140352	NA	NA	9.64	NA	1.04	NA
B2RDG0	Proteasome subunit alpha type	NA	3.50					0.127871	0.685755	NA	NA	27.01	NA	0.41	NA					0.079058	1.186235	NA	NA	51.75	NA	0.63	NA
B4DE50	cDNA FLJ59582, highly similar to Ras-related protein Rab-3A	NA	2.00					-0.23191	0.219078	NA	NA	21.93	NA	-0.01	NA					-0.68657	-0.40874	NA	NA	13.58	NA	-0.55	NA
B4E0S6	cDNA FLJ55635, highly similar to pre-mRNA-splicing factorATP-dependent RNA helicase DHX15 (EC 3.6.1.-)	NA	1.00					-0.44867	-0.03983	NA	NA	19.91	NA	-0.24	NA					-0.1879	-0.47058	NA	NA	13.81	NA	-0.33	NA
B4E2V7	cDNA FLJ54243, highly similar to Secretory carrier-associated membrane protein1	NA	1.00					-0.06205	0.011964	NA	NA	3.63	NA	-0.03	NA					-0.16964	-0.46082	NA	NA	14.22	NA	-0.32	NA
G3V325	Protein ATP5J2-PTCD1	ATP5J2-PTCD1	2.00					-0.59176	-0.48156	NA	NA	5.40	NA	-0.54	NA					-0.37179	0.041468	NA	NA	20.12	NA	-0.17	NA
H3BSC1	Ras-related protein Rab-11A	RAB11A	4.00					0.222203	-0.32571	NA	NA	26.54	NA	-0.05	NA					0.017879	0.000321	NA	NA	0.86	NA	0.01	NA
M0R389	Platelet-activating factor acetylhydrolase IB subunit gamma (Fragment)	PAFAH1B3	1.00					-0.15955	-0.02567	NA	NA	6.56	NA	-0.09	NA					0.357345	-0.04623	NA	NA	19.65	NA	0.16	NA
Q53FU3	Coatomer protein complex, subunit zeta 1 variant (Fragment)	NA	1.00					0.221724	0.29246	NA	NA	3.47	NA	0.26	NA					-0.28508	0.421659	NA	NA	33.96	NA	0.07	NA
Q59GE4	Ribosomal protein S10 variant (Fragment)	NA	2.00					0.595849	0.223685	NA	NA	18.14	NA	0.41	NA					0.313425	-0.24476	NA	NA	27.02	NA	0.03	NA
Q59GV0	Propionyl Coenzyme A carboxylase, beta polypeptide variant (Fragment)	NA	1.50					0.267617	0.228277	NA	NA	1.93	NA	0.25	NA					0.229671	1.170532	NA	NA	44.55	NA	0.70	NA
Q5T8R5	Monocarboxylate transporter 1 (Fragment)	SLC16A1	2.00					2.686887	2.067035	NA	NA	29.92	NA	2.38	NA					1.767613	1.394961	NA	NA	18.16	NA	1.58	NA
Q68CR4	Putative uncharacterized protein DKFZp781I1948	DKFZp781I1948	3.50					-2.02204	-2.12635	NA	NA	5.11	NA	-2.07	NA					-1.82808	-2.06268	NA	NA	11.47	NA	-1.95	NA
Q6EZE9	Defensin, alpha 3, neutrophil-specific	DEFA3	1.50					-3.42483	-3.27165	NA	NA	7.50	NA	-3.35	NA					-3.6314	-3.4305	NA	NA	9.83	NA	-3.53	NA
Q6FIC5	Chloride intracellular channel protein	CLIC4	1.50					-0.22486	0.962707	NA	NA	55.13	NA	0.37	NA					0.784878	1.144813	NA	NA	17.55	NA	0.96	NA
Q6LEN2	Prostacyclin synthase	PTGIS	1.50					0.063514	-2.90273	NA	NA	109.33	NA	-1.42	NA					0.800159	-2.06626	NA	NA	107.31	NA	-0.63	NA
Q6N0A8	Putative uncharacterized protein DKFZp686O07189	DKFZp686O07189	1.00					-0.05086	1.06068	NA	NA	51.94	NA	0.50	NA					-0.97073	0.497625	NA	NA	66.34	NA	-0.24	NA
Q75MU2	Putative uncharacterized protein WBSCR1 (Fragment)	WBSCR1	3.00					-0.78609	-0.02783	NA	NA	36.33	NA	-0.41	NA					-0.00788	-0.08181	NA	NA	3.62	NA	-0.04	NA
Q8WZ56	Serine/threonine-protein phosphatase	NA	2.00					0.148196	0.091714	NA	NA	2.77	NA	0.12	NA					0.238636	0.164049	NA	NA	3.65	NA	0.20	NA
V9HW38	Epididymis secretory protein Li 106	HEL-S-106	4.00					0.985106	6.614157	NA	NA	135.82	NA	3.80	NA					0.129833	7.213357	NA	NA	139.35	NA	3.67	NA
V9HW39	Epididymis secretory protein Li 100	HEL-S-100	1.00					0.980296	0.843484	NA	NA	6.70	NA	0.91	NA					0.788594	0.856027	NA	NA	3.30	NA	0.82	NA
A0A0A6YYG9	Protein ARPC4-TTLL3	ARPC4-TTLL3	1.50					-0.07834	-0.19572	NA	NA	5.75	NA	-0.14	NA					0.266236	-0.35956	NA	NA	30.20	NA	-0.05	NA
M0R210	40S ribosomal protein S16	RPS16	1.00					0.145924	1.198165	NA	NA	49.40	NA	0.67	NA					-0.52849	0.66792	NA	NA	55.50	NA	0.07	NA
Q5QPM0	RNA-binding protein Raly (Fragment)	RALY	1.00					0.089103	0.471205	NA	NA	18.62	NA	0.28	NA					-0.2668	-0.04314	NA	NA	10.94	NA	-0.15	NA
O75323	Protein NipSnap homolog 2	GBAS	2.00	0.26896						NA	NA	NA	NA	0.27	NA	1.07836						NA	NA	NA	NA	1.08	NA
O75381	Peroxisomal membrane protein PEX14	PEX14	3.00	-0.53334						NA	NA	NA	NA	-0.53	NA	-0.15727						NA	NA	NA	NA	-0.16	NA
P01023	Alpha-2-macroglobulin	A2M	1.00	0.841398						NA	NA	NA	NA	0.84	NA	1.045722						NA	NA	NA	NA	1.05	NA
P06702	Protein S100-A9	S100A9	3.00	-0.92763						NA	NA	NA	NA	-0.93	NA	-0.19315						NA	NA	NA	NA	-0.19	NA
P50583	Bis(5'-nucleosyl)-tetraphosphatase [asymmetrical]	NUDT2	1.00	-0.67324						NA	NA	NA	NA	-0.67	NA	0.047631						NA	NA	NA	NA	0.05	NA
P54577	Tyrosine--tRNA ligase, cytoplasmic	YARS	1.00	0.751303						NA	NA	NA	NA	0.75	NA	0.753133						NA	NA	NA	NA	0.75	NA
Q10713	Mitochondrial-processing peptidase subunit alpha	PMPCA	1.00	0.718063						NA	NA	NA	NA	0.72	NA	0.889254						NA	NA	NA	NA	0.89	NA
Q8IYJ1	Copine-9	CPNE9	1.00	-0.03891						NA	NA	NA	NA	-0.04	NA	-0.4253						NA	NA	NA	NA	-0.43	NA
Q969E2	Secretory carrier-associated membrane protein 4	SCAMP4	1.00	0.242416						NA	NA	NA	NA	0.24	NA	0.531543						NA	NA	NA	NA	0.53	NA

				08/017 hESC-RPE to hRPE												08/023 hESC-RPE to hRPE											
				Analysis						Technical replicate CV			Biological	Fold change (mean)	Unadjusted p-value	Analysis						Technical replicate CV			Biological	Fold change (mean)	Unadjusted p-value
UniProt	Protein name	Gene name	Peptide mean	1.1	1.2	2.1	2.2	3.1	3.2	1	2	3	CV		(t-test)	1.1	1.2	2.1	2.2	3.1	3.2	1	2	3	CV		(t-test)
Q99426	Tubulin-folding cofactor B	TBCB	1.00	0.369291						NA	NA	NA	NA	0.37	NA	0.313489						NA	NA	NA	NA	0.31	NA
Q9BRF8	Serine/threonine-protein phosphatase CPPED1	CPPED1	1.00	0.160775						NA	NA	NA	NA	0.16	NA	-0.29592						NA	NA	NA	NA	-0.30	NA
Q9H479	Fructosamine-3-kinase	FN3K	1.00	-0.03648						NA	NA	NA	NA	-0.04	NA	0.850284						NA	NA	NA	NA	0.85	NA
Q9Y2J2	Band 4.1-like protein 3	EPB41L3	4.00	-2.1433						NA	NA	NA	NA	-2.14	NA	-2.88547						NA	NA	NA	NA	-2.89	NA
Q9Y3B4	Splicing factor 3B subunit 6	SF3B6	1.00	0.79994						NA	NA	NA	NA	0.80	NA	1.036564						NA	NA	NA	NA	1.04	NA
A0A024R040	Succinyl-CoA:3-ketoacid-coenzyme A transferase	OXCT1	6.00	0.247565						NA	NA	NA	NA	0.25	NA	0.289666						NA	NA	NA	NA	0.29	NA
A0A024R1N8	Solute carrier 16 (Monocarboxylic acid transporters), member 8, isoform CRA_a	SLC16A8	1.00	0.103664						NA	NA	NA	NA	0.10	NA	0.488929						NA	NA	NA	NA	0.49	NA
A0A024R1U2	PHD finger protein 5A, isoform CRA_a	PHF5A	1.00	-0.56502						NA	NA	NA	NA	-0.57	NA	0.312078						NA	NA	NA	NA	0.31	NA
A0A024R4T4	Ubiquitin-conjugating enzyme E2M (UBC12 homolog, yeast), isoform CRA_a	UBE2M	1.00	0.645296						NA	NA	NA	NA	0.65	NA	0.350222						NA	NA	NA	NA	0.35	NA
A0A024R4X0	NADH-cytochrome b5 reductase	CYB5R3	5.00	-0.23068						NA	NA	NA	NA	-0.23	NA	-0.30375						NA	NA	NA	NA	-0.30	NA
A0A024RBC7	Calcium-transporting ATPase	ATP2B1	7.00	-1.17851						NA	NA	NA	NA	-1.18	NA	-0.71356						NA	NA	NA	NA	-0.71	NA
A0A024RDY0	RAN binding protein 5, isoform CRA_d	RANBP5	3.00	0.311616						NA	NA	NA	NA	0.31	NA	-0.76402						NA	NA	NA	NA	-0.76	NA
A0A087WZQ7	Beta-soluble NSF attachment protein	NAPB	2.00	-0.82468						NA	NA	NA	NA	-0.82	NA	-0.38927						NA	NA	NA	NA	-0.39	NA
A0A0A0MS51	Gelsolin	GSN	11.00	0.548617						NA	NA	NA	NA	0.55	NA	0.560061						NA	NA	NA	NA	0.56	NA
A0A0D9SG07	Band 4.1-like protein 1	EPB41L1	1.00	0.558408						NA	NA	NA	NA	0.56	NA	0.461661						NA	NA	NA	NA	0.46	NA
A0A0J9YYJ0	Protein canopy homolog 4 (Fragment)	CNPY4	1.00	-0.70497						NA	NA	NA	NA	-0.70	NA	1.225519						NA	NA	NA	NA	1.23	NA
A1L306	TNR protein	TNR	1.00	-0.17807						NA	NA	NA	NA	-0.18	NA	-1.5142						NA	NA	NA	NA	-1.51	NA
A7E2D8	Calcium-transporting ATPase	ATP2B4	14.00	-0.81461						NA	NA	NA	NA	-0.81	NA	-0.90503						NA	NA	NA	NA	-0.91	NA
A8K6V3	cDNA FLJ78677, highly similar to Homo sapiens splicing factor 3b, subunit 3, 130kDa (SF3B3), mRNA	NA	2.00	0.81948						NA	NA	NA	NA	0.82	NA	-0.14317						NA	NA	NA	NA	-0.14	NA
B1PS43	Myosin heavy chain 11 smooth muscle isoform	MYH11	3.00	2.760716						NA	NA	NA	NA	2.76	NA	4.146942						NA	NA	NA	NA	4.15	NA
B2R7W4	cDNA, FLJ93632, highly similar to Homo sapiens heterogeneous nuclear ribonucleoprotein R (HNRPR), mRN	NA	6.00	-0.27121						NA	NA	NA	NA	-0.27	NA	-0.40688						NA	NA	NA	NA	-0.41	NA
B2RAQ4	Sodium/potassium-transporting ATPase subunit alpha	NA	19.00	0.696118						NA	NA	NA	NA	0.70	NA	-0.08205						NA	NA	NA	NA	-0.08	NA
B2RB89	Protein kinase, cAMP-dependent, catalytic, beta, isoform CRA_a	PRKACB	1.00	-0.72664						NA	NA	NA	NA	-0.73	NA	-0.88911						NA	NA	NA	NA	-0.89	NA
B3KNU0	cDNA FLJ30470 fis, clone BRAWH1000040, highly similar to Rap1 GTPase-GDP dissociation stimulator 1	NA	4.00	-2.77466						NA	NA	NA	NA	-2.77	NA	-0.70497						NA	NA	NA	NA	-0.70	NA
B3KP90	cDNA FLJ31447 fis, clone NT2NE2000913, highly similar to Protein phosphatase 2C isoform beta (EC 3.1.3.1	NA	1.00	1.26994						NA	NA	NA	NA	1.27	NA	1.571018						NA	NA	NA	NA	1.57	NA
B3KY11	cDNA FLJ46571 fis, clone THYMU3041428, highly similar to Probable ATP-dependent RNA helicase DDX23 (	NA	1.00	0.160423						NA	NA	NA	NA	0.16	NA	-0.24206						NA	NA	NA	NA	-0.24	NA
B4DFG0	Protein DEK	DEK	1.00	-0.02742						NA	NA	NA	NA	-0.03	NA	0.483297						NA	NA	NA	NA	0.48	NA
B4DIS3	Dpy-30-like protein, isoform CRA_b	LOC84661	2.00	0.094659						NA	NA	NA	NA	0.09	NA	-1.32885						NA	NA	NA	NA	-1.33	NA
B4DJB1	cDNA FLJ53049, highly similar to AP-2 complex subunit mu-1	NA	2.00	0.497803						NA	NA	NA	NA	0.50	NA	0.532603						NA	NA	NA	NA	0.53	NA
B4DL06	cDNA FLJ59565, highly similar to Beta-catenin	NA	2.00	0.263559						NA	NA	NA	NA	0.26	NA	0.502294						NA	NA	NA	NA	0.50	NA
B4DLT2	cDNA FLJ56637, highly similar to Nuclear pore complex protein Nup155	NA	1.00	-0.45813						NA	NA	NA	NA	-0.46	NA	-2.29566						NA	NA	NA	NA	-2.30	NA
B4DP10	cDNA FLJ55118, highly similar to Secretory carrier-associated membrane protein 1	NA	1.00	-1.35407						NA	NA	NA	NA	-1.35	NA	-0.5684						NA	NA	NA	NA	-0.57	NA
B4DPJ2	Annexin	NA	4.00	1.103297						NA	NA	NA	NA	1.10	NA	0.903849						NA	NA	NA	NA	0.90	NA
B4DPW5	cDNA FLJ59374, highly similar to Homo sapiens caldesmon 1 (CALD1), transcript variant 2, mRNA	NA	1.00	1.252283						NA	NA	NA	NA	1.25	NA	1.653291						NA	NA	NA	NA	1.65	NA
B4DR82	cDNA FLJ60331, highly similar to Nicastrin	NA	2.00	0.031927						NA	NA	NA	NA	0.03	NA	0.047631						NA	NA	NA	NA	0.05	NA
B4DSU5	cDNA FLJ55583, highly similar to Polypyrimidine tract-binding protein 2	NA	2.00	-0.61028						NA	NA	NA	NA	-0.61	NA	-1.04677						NA	NA	NA	NA	-1.05	NA
B4DTS5	cDNA FLJ58882, highly similar to 26S proteasome non-ATPase regulatory subunit 11	NA	2.00	0.637852						NA	NA	NA	NA	0.64	NA	1.594717						NA	NA	NA	NA	1.59	NA
B4DZX7	Thioredoxin domain containing, isoform CRA_b	TXNDC	1.00	-0.10533						NA	NA	NA	NA	-0.11	NA	-0.49771						NA	NA	NA	NA	-0.50	NA
B4E091	cDNA FLJ55438, highly similar to Splicing factor 3 subunit 1	NA	1.00	-0.07391						NA	NA	NA	NA	-0.07	NA	-0.71716						NA	NA	NA	NA	-0.72	NA
B7Z2F7	cDNA FLJ54655, highly similar to Heat shock 70 kDa protein 12A	NA	1.00	-0.30704						NA	NA	NA	NA	-0.31	NA	-0.99431						NA	NA	NA	NA	-0.99	NA
B7Z6U8	cDNA FLJ53665, highly similar to Four and a half LIM domains protein 1	NA	2.00	-0.77499						NA	NA	NA	NA	-0.77	NA	-0.30998						NA	NA	NA	NA	-0.31	NA
B8ZZB8	CB1 cannabinoid receptor-interacting protein 1	CNRIP1	1.00	-1.83659						NA	NA	NA	NA	-1.84	NA	-0.98128						NA	NA	NA	NA	-0.98	NA
D3DQR0	Protein kinase C and casein kinase substrate in neurons 3, isoform CRA_b	PACSIN3	1.00	0.890539						NA	NA	NA	NA	0.89	NA	-0.50266						NA	NA	NA	NA	-0.50	NA
D3DUG9	Ubiquitin carboxyl-terminal hydrolase	USP14	2.00	0.503507						NA	NA	NA	NA	0.50	NA	0.349363						NA	NA	NA	NA	0.35	NA
E7EPA1	Phosphoribosyl pyrophosphate synthase-associated protein 2 (Fragment)	PRPSAP2	1.00	0.552783						NA	NA	NA	NA	0.55	NA	0.055454						NA	NA	NA	NA	0.06	NA
E9PP60	GDP-L-fucose synthase	TSTA3	1.00	0.845786						NA	NA	NA	NA	0.85	NA	0.056929						NA	NA	NA	NA	0.06	NA
F4ZW65	NF90b	NA	11.00	0.262575						NA	NA	NA	NA	0.26	NA	0.272716						NA	NA	NA	NA	0.27	NA
F8RHH9	MHC class I antigen (Fragment)	HLA-C	2.00	1.096593						NA	NA	NA	NA	1.10	NA	0.437432						NA	NA	NA	NA	0.44	NA
F8VZQ9	SAP domain-containing ribonucleoprotein	SARNP	2.00	1.005599						NA	NA	NA	NA	1.01	NA	0.777626						NA	NA	NA	NA	0.78	NA
G3V500	Echinoderm microtubule-associated protein-like 1 (Fragment)	EML1	1.00	-0.59177						NA	NA	NA	NA	-0.59	NA	-0.36903						NA	NA	NA	NA	-0.37	NA
H0YMV8	40S ribosomal protein S27	RPS27L	1.00	0.613613						NA	NA	NA	NA	0.61	NA	0.599931						NA	NA	NA	NA	0.60	NA
H7BY36	RNA-binding protein EWS (Fragment)	EWSR1	1.00	1.84606						NA	NA	NA	NA	1.85	NA	0.881305						NA	NA	NA	NA	0.88	NA
H7BY58	Protein-L-isoaspartate O-methyltransferase	PCMT1	4.00	1.380978						NA	NA	NA	NA	1.38	NA	1.431839						NA	NA	NA	NA	1.43	NA
H7BYY1	Tropomyosin 1 (Alpha), isoform CRA_m	TPM1	4.00	0.525008						NA	NA	NA	NA	0.53	NA	1.100908						NA	NA	NA	NA	1.10	NA
H7C3G9	N-acetyl-D-glucosamine kinase	NAGK	1.00	0.639116						NA	NA	NA	NA	0.64	NA	0.187349						NA	NA	NA	NA	0.19	NA
K7ERI9	Apolipoprotein C-I (Fragment)	APOC1	1.00	-0.77466						NA	NA	NA	NA	-0.77	NA	-1.16168						NA	NA	NA	NA	-1.16	NA
Q05CK9	SYNCRIP protein (Fragment)	SYNCRIP	5.00	-0.85463						NA	NA	NA	NA	-0.85	NA	-1.49554						NA	NA	NA	NA	-1.50	NA
Q53HD9	Tetratricopeptide repeat domain 1 variant (Fragment)	NA	1.00	1.394968						NA	NA	NA	NA	1.39	NA	1.406356						NA	NA	NA	NA	1.41	NA
Q53HW7	Sulfide dehydrogenase like (Fragment)	NA	1.00	1.412673						NA	NA	NA	NA	1.41	NA	0.781455						NA	NA	NA	NA	0.78	NA
Q53TX0	Putative uncharacterized protein GLS (Fragment)	GLS	2.00	1.096961						NA	NA	NA	NA	1.10	NA	1.497335						NA	NA	NA	NA	1.50	NA
Q53XC0	Eukaryotic translation initiation factor 2, subunit 1 alpha, 35kDa, isoform CRA_a	EIF2S1	1.00	0.873664						NA	NA	NA	NA	0.87	NA	1.194739						NA	NA	NA	NA	1.19	NA
Q53Y03	COX4 neighbor, isoform CRA_a	COX4NB	1.00	1.438976						NA	NA	NA	NA	1.44	NA	0.649944						NA	NA	NA	NA	0.65	NA
Q549C5	HCG2010808, isoform CRA_a	MST065	1.00	-0.15159						NA	NA	NA	NA	-0.15	NA	0.495938						NA	NA	NA	NA	0.50	NA
Q59EC0	Adenosine deaminase, RNA-specific isoform ADAR-a variant (Fragment)	NA	1.00	0.102747						NA	NA	NA	NA	0.10	NA	0.072225						NA	NA	NA	NA	0.07	NA
Q59EL2	COP9 constitutive photomorphogenic homolog subunit 2 variant (Fragment)	NA	1.00	-1.47636						NA	NA	NA	NA	-1.48	NA	-0.79183						NA	NA	NA	NA	-0.79	NA
Q59EL5	Aldo-keto reductase family 1, member B1 variant (Fragment)	NA	2.00	0.403327						NA	NA	NA	NA	0.40	NA	-0.09182						NA	NA	NA	NA	-0.09	NA
Q59GY3	Arginine/serine-rich splicing factor 6 variant (Fragment)	NA	2.00	0.521584						NA	NA	NA	NA	0.52	NA	-0.03999						NA	NA	NA	NA	-0.04	NA
Q5TBH8	Dihydroxyacetone phosphate acyltransferase (Fragment)	GNPAT	1.00	1.6331						NA	NA	NA	NA	1.63	NA	2.762948						NA	NA	NA	NA	2.76	NA
Q5TZN6	Nucleolar protein 3 (Apoptosis repressor with CARD domain)	NOL3	1.00	0.176537						NA	NA	NA	NA	0.18	NA	-0.64926						NA	NA	NA	NA	-0.65	NA
Q5U5J2	CSNK2A1 protein	CSNK2A1	1.00	0.083555						NA	NA	NA	NA	0.08	NA	-0.17894						NA	NA	NA	NA	-0.18	NA
Q63HR1	Putative uncharacterized protein DKFZp686P17171	DKFZp686P17171	1.00	-1.76058						NA	NA	NA	NA	-1.76	NA	-1.29404						NA	NA	NA	NA	-1.29	NA
Q6IB91	PCK2 protein	PCK2	1.00	-0.0931						NA	NA	NA	NA	-0.09	NA	-0.56678						NA	NA	NA	NA	-0.57	NA
Q6IBP2	HMOX2 protein	HMOX2	3.00	-0.3051						NA	NA	NA	NA	-0.31	NA	-0.9038						NA	NA	NA	NA	-0.90	NA
Q6ZVA1	cDNA FLJ16001 fis, clone BRAWH2001395, moderately similar to MYELIN BASIC PROTEIN	NA	1.00	-2.10384						NA	NA	NA	NA	-2.10	NA	-3.16315						NA	NA	NA	NA	-3.16	NA
Q7Z517	Transgelin	NA	2.00	-0.31119						NA	NA	NA	NA	-0.31	NA	-0.75143						NA	NA	NA	NA	-0.75	NA
Q8N1H4	cDNA FLJ40872 fis, clone TUTER2000283, highly similar to Homo sapiens transformer-2-beta (SFRS10) gene	NA	1.00	0.672599						NA	NA	NA	NA	0.67	NA	1.367904						NA	NA	NA	NA	1.37	NA
Q9UFM8	Neuroplastin (Fragment)	DKFZp566H1924	1.00	0.618493						NA	NA	NA	NA	0.62	NA	0.624728						NA	NA	NA	NA	0.62	NA
Q9UFQ0	Putative uncharacterized protein DKFZp434K0435 (Fragment)	DKFZp434K0435	6.00	0.037116						NA	NA	NA	NA	0.04	NA	-0.3127						NA	NA	NA	NA	-0.31	NA
V9HW34	Epididymis luminal protein 213	HEL-213	1.00	0.748142						NA	NA	NA	NA	0.75	NA	0.930076						NA	NA	NA	NA	0.93	NA
V9HW44	Epididymis secretory protein Li 303	PAFAH1B2	2.00	-0.22538						NA	NA	NA	NA	-0.23	NA	0.298779						NA	NA	NA	NA	0.30	NA
V9HW91	Epididymis secretory sperm binding protein Li 8a	NIT2	3.00	0.169378						NA	NA	NA	NA	0.17	NA	0.217061						NA	NA	NA	NA	0.22	NA
X6RLR1	Dynactin subunit 3	DCTN3	2.00	-2.42789						NA	NA	NA	NA	-2.43	NA	-4.74811						NA	NA	NA	NA	-4.75	NA
A6NDG6	Phosphoglycolate phosphatase	PGP	2.00		1.174894					NA	NA	NA	NA	1.17	NA		1.623448					NA	NA	NA	NA	1.62	NA
O75521	Enoyl-CoA delta isomerase 2, mitochondrial	ECI2	1.00		0.261927					NA	NA	NA	NA	0.26	NA		-0.00088					NA	NA	NA	NA	0.00	NA
P23378	Glycine dehydrogenase (decarboxylating), mitochondrial	GLDC	1.00		-0.90097					NA	NA	NA	NA	-0.90	NA		-0.8167					NA	NA	NA	NA	-0.82	NA
P36542	ATP synthase subunit gamma, mitochondrial	ATP5C1	10.00		-0.46316					NA	NA	NA	NA	-0.46	NA		-0.52941					NA	NA	NA	NA	-0.53	NA
P62318	Small nuclear ribonucleoprotein Sm D3	SNRPD3	1.00		0.739393					NA	NA	NA	NA	0.74	NA		1.401032					NA	NA	NA	NA	1.40	NA
P68133	Actin, alpha skeletal muscle	ACTA1	21.00		0.146278					NA	NA	NA	NA	0.15	NA		0.431416					NA	NA	NA	NA	0.43	NA
P68363	Tubulin alpha-1B chain	TUBA1B	62.00		-0.9928					NA	NA	NA	NA	-0.99	NA		-1.25561					NA	NA	NA	NA	-1.26	NA
Q13242	Serine/arginine-rich splicing factor 9	SRSF9	2.00		0.968236					NA	NA	NA	NA	0.97	NA		-0.12299					NA	NA	NA	NA	-0.12	NA
Q52LJ0	Protein FAM98B	FAM98B	2.00		0.013415					NA	NA	NA	NA	0.01	NA		-0.3301					NA	NA	NA	NA	-0.33	NA
Q5SRE7	Phytanoyl-CoA dioxygenase domain-containing protein 1	PHYHD1	1.00		-0.51702					NA	NA	NA	NA	-0.52	NA		-0.05137					NA	NA	NA	NA	-0.05	NA
Q6YHU6	Thyroid adenoma-associated protein	THADA	1.00		0.440113					NA	NA	NA	NA	0.44	NA		0.541037					NA	NA	NA	NA	0.54	NA
Q7Z4V5	Hepatoma-derived growth factor-related protein 2	HDGFRP2	1.00		-0.79507					NA	NA	NA	NA	-0.80	NA		-1.42218					NA	NA	NA	NA	-1.42	NA

				08/017 hESC-RPE to hRPE												08/023 hESC-RPE to hRPE											
				Analysis						Technical replicate CV			Biological	Fold change (mean)	Unadjusted p-value	Analysis						Technical replicate CV			Biological	Fold change (mean)	Unadjusted p-value
UniProt	Protein name	Gene name	Peptide mean	1.1	1.2	2.1	2.2	3.1	3.2	1	2	3	CV		(t-test)	1.1	1.2	2.1	2.2	3.1	3.2	1	2	3	CV		(t-test)
Q8NBU5	ATPase family AAA domain-containing protein 1	ATAD1	1.00		-0.10082					NA	NA	NA	NA	-0.10	NA		-1.15747					NA	NA	NA	NA	-1.16	NA
Q8TDB4	Protein MGARP	MGARP	3.00		-0.90097					NA	NA	NA	NA	-0.90	NA		-0.70507					NA	NA	NA	NA	-0.71	NA
Q92928	Putative Ras-related protein Rab-1C	RAB1C	7.00		-1.15834					NA	NA	NA	NA	-1.16	NA		-1.08837					NA	NA	NA	NA	-1.09	NA
Q96AG4	Leucine-rich repeat-containing protein 59	LRRC59	1.00		0.019234					NA	NA	NA	NA	0.02	NA		-0.48741					NA	NA	NA	NA	-0.49	NA
Q9BVA1	Tubulin beta-2B chain	TUBB2B	63.00		-0.99205					NA	NA	NA	NA	-0.99	NA		-0.62847					NA	NA	NA	NA	-0.63	NA
Q9H477	Ribokinase	RBKS	1.00		-0.42163					NA	NA	NA	NA	-0.42	NA		-0.37426					NA	NA	NA	NA	-0.37	NA
Q9P0I2	ER membrane protein complex subunit 3	EMC3	1.00		0.306661					NA	NA	NA	NA	0.31	NA		-0.87855					NA	NA	NA	NA	-0.88	NA
Q9UBI6	Guanine nucleotide-binding protein G(I)/G(S)/G(O) subunit gamma-12	GNG12	1.00		1.562001					NA	NA	NA	NA	1.56	NA		1.730716					NA	NA	NA	NA	1.73	NA
Q9UIF8	Bromodomain adjacent to zinc finger domain protein 2B	BAZ2B	1.00		-0.63469					NA	NA	NA	NA	-0.63	NA		-0.58684					NA	NA	NA	NA	-0.59	NA
Q9UKA9	Polypyrimidine tract-binding protein 2	PTBP2	3.00		0.9251					NA	NA	NA	NA	0.93	NA		0.44621					NA	NA	NA	NA	0.45	NA
Q9Y266	Nuclear migration protein nudC	NUDC	2.00		1.173296					NA	NA	NA	NA	1.17	NA		1.175033					NA	NA	NA	NA	1.18	NA
A0A024R0Y6	ADP-ribosylation factor 3, isoform CRA_b	ARF3	6.00		-0.40419					NA	NA	NA	NA	-0.40	NA		-0.4281					NA	NA	NA	NA	-0.43	NA
A0A024R179	Nuclear cap binding protein subunit 1, 80kDa, isoform CRA_a	NCBP1	1.00		-0.12257					NA	NA	NA	NA	-0.12	NA		1.028366					NA	NA	NA	NA	1.03	NA
A0A024R1A4	Ubiquitin-conjugating enzyme E2L 3, isoform CRA_a	UBE2L3	2.00		0.473308					NA	NA	NA	NA	0.47	NA		0.203156					NA	NA	NA	NA	0.20	NA
A0A024R1K8	Splicing factor 3a, subunit 1, 120kDa, isoform CRA_a	SF3A1	1.00		1.047428					NA	NA	NA	NA	1.05	NA		0.378834					NA	NA	NA	NA	0.38	NA
A0A024R1N4	X-ray repair complementing defective repair in Chinese hamster cells 6 (Ku autoantigen, 70kDa), isoform C	XRCC6	16.00		-0.15686					NA	NA	NA	NA	-0.16	NA		-0.3043					NA	NA	NA	NA	-0.30	NA
A0A024R977	ADP-ribosylation factor-like 8A, isoform CRA_a	ARL8A	3.00		0.105043					NA	NA	NA	NA	0.11	NA		0.465808					NA	NA	NA	NA	0.47	NA
A0A024RC37	Uncharacterized protein	P15RS	1.00		3.723429					NA	NA	NA	NA	3.72	NA		3.119878					NA	NA	NA	NA	3.12	NA
A0A024RD80	Heat shock protein 90kDa alpha (Cytosolic), class B member 1, isoform CRA_a	HSP90AB1	30.00		0.37471					NA	NA	NA	NA	0.37	NA		0.25615					NA	NA	NA	NA	0.26	NA
A0A087WUK2	Heterogeneous nuclear ribonucleoprotein D-like	HNRNPDL	7.00		-0.51159					NA	NA	NA	NA	-0.51	NA		-0.19608					NA	NA	NA	NA	-0.20	NA
A0A087X130	Ig kappa chain C region	IGKC	2.00		-1.03536					NA	NA	NA	NA	-1.04	NA		-0.35582					NA	NA	NA	NA	-0.36	NA
A0A0A0MS87	Protein NDRG2	NDRG2	1.00		0.017547					NA	NA	NA	NA	0.02	NA		-0.49609					NA	NA	NA	NA	-0.50	NA
A0A0A1HAW1	H.sapiens ras-related Hrab2 protein	NA	3.00		0.024656					NA	NA	NA	NA	0.02	NA		-0.13141					NA	NA	NA	NA	-0.13	NA
A0A0J9YYL3	Poly(U)-binding-splicing factor PUF60 (Fragment)	PUF60	1.00		0.454453					NA	NA	NA	NA	0.45	NA		-0.46777					NA	NA	NA	NA	-0.47	NA
A8K5C4	cDNA FLJ76290, highly similar to Homo sapiens TIA1 cytotoxic granule-associated RNA bindingprotein-like	NA	1.00		0.024469					NA	NA	NA	NA	0.02	NA		0.193361					NA	NA	NA	NA	0.19	NA
A8K644	Splicing factor, arginine/serine-rich 4, isoform CRA_b	SFRS4	3.00		0.880737					NA	NA	NA	NA	0.88	NA		0.921085					NA	NA	NA	NA	0.92	NA
A8K7W3	cDNA FLJ75780, highly similar to Homo sapiens SGT1, suppressor of G2 allele of SKP1 (SUGT1), mRNA	NA	2.00		-1.19134					NA	NA	NA	NA	-1.19	NA		-0.32347					NA	NA	NA	NA	-0.32	NA
A8K8U3	Calcium-transporting ATPase	NA	10.00		-0.17197					NA	NA	NA	NA	-0.17	NA		-0.07485					NA	NA	NA	NA	-0.07	NA
A8KAQ0	cDNA FLJ75858, highly similar to Homo sapiens dopachrome tautomerase (dopachrome delta-isomerase, t	NA	3.00		2.125677					NA	NA	NA	NA	2.13	NA		2.334827					NA	NA	NA	NA	2.33	NA
B1AKR6	Dynein light chain roadblock-type 1	DYNLRB1	1.00		0.049264					NA	NA	NA	NA	0.05	NA		-0.4278					NA	NA	NA	NA	-0.43	NA
B2R4K7	60S ribosomal protein L6	NA	1.00		-1.55082					NA	NA	NA	NA	-1.55	NA		-1.05045					NA	NA	NA	NA	-1.05	NA
B2R694	Terpene cyclase/mutase family member	NA	1.00		1.166377					NA	NA	NA	NA	1.17	NA		0.544969					NA	NA	NA	NA	0.54	NA
B2RD40	cDNA, FLJ96442, highly similar to Homo sapiens copine II (CPNE2), mRNA	NA	1.00		-0.3019					NA	NA	NA	NA	-0.30	NA		-0.5593					NA	NA	NA	NA	-0.56	NA
B2RDI5	cDNA, FLJ96627, highly similar to Homo sapiens calpain 1, (mu/I) large subunit (CAPN1), mRNA	NA	1.00		-0.04649					NA	NA	NA	NA	-0.05	NA		0.283022					NA	NA	NA	NA	0.28	NA
B3KY28	cDNA FLJ46689 fis, clone TRACH3012106, highly similar to Band 4.1-like protein 3	NA	3.00		-1.90737					NA	NA	NA	NA	-1.91	NA		-1.03863					NA	NA	NA	NA	-1.04	NA
B3KY43	cDNA FLJ46798 fis, clone TRACH3031660, highly similar to cAMP-dependent protein kinase type II-beta reg	NA	6.00		-1.62032					NA	NA	NA	NA	-1.62	NA		-1.35695					NA	NA	NA	NA	-1.36	NA
B4DDX2	cDNA FLJ54590, highly similar to KH domain-containing, RNA-binding, signaltransduction-associated protei	NA	3.00		0.540497					NA	NA	NA	NA	0.54	NA		0.232337					NA	NA	NA	NA	0.23	NA
B4DNW3	cDNA FLJ51914, highly similar to Protein DEK	NA	2.00		-1.96327					NA	NA	NA	NA	-1.96	NA		-2.5694					NA	NA	NA	NA	-2.57	NA
B4DR52	Histone H2B	NA	23.00		-1.85905					NA	NA	NA	NA	-1.86	NA		-0.94955					NA	NA	NA	NA	-0.95	NA
B4DVE0	cDNA FLJ55794, highly similar to Medium-chain specific acyl-CoA dehydrogenase, mitochondrial (EC 1.3.99	NA	5.00		0.743141					NA	NA	NA	NA	0.74	NA		0.860843					NA	NA	NA	NA	0.86	NA
B4E2L0	cDNA FLJ54730, highly similar to cAMP-dependent protein kinase, beta-2-catalytic subunit (EC 2.7.11.11)	NA	1.00		0.126416					NA	NA	NA	NA	0.13	NA		-0.26061					NA	NA	NA	NA	-0.26	NA
B7Z5V0	cDNA FLJ53647, highly similar to Four and a half LIM domains protein 1	NA	2.00		-0.6113					NA	NA	NA	NA	-0.61	NA		-0.63222					NA	NA	NA	NA	-0.63	NA
C9J0A2	Synaptoporin	SYNPR	1.00		0.440113					NA	NA	NA	NA	0.44	NA		0.036339					NA	NA	NA	NA	0.04	NA
D3DP78	Aspartyl-tRNA synthetase, isoform CRA_b	DARS	1.00		0.305279					NA	NA	NA	NA	0.31	NA		0.503291					NA	NA	NA	NA	0.50	NA
D3DQX7	Serum amyloid A protein	SAA1	2.00		0.353026					NA	NA	NA	NA	0.35	NA		0.500178					NA	NA	NA	NA	0.50	NA
E5KLM1	Mitochondrial dynamin-like 120 kDa protein	NA	4.00		-0.19764					NA	NA	NA	NA	-0.20	NA		0.502202					NA	NA	NA	NA	0.50	NA
E5RIM7	Copper transport protein ATOX1	ATOX1	2.00		0.659324					NA	NA	NA	NA	0.66	NA		0.631863					NA	NA	NA	NA	0.63	NA
E5RK17	Protein NDRG1 (Fragment)	NDRG1	1.00		-0.82873					NA	NA	NA	NA	-0.83	NA		-0.59747					NA	NA	NA	NA	-0.60	NA
E7EX59	Propionyl-CoA carboxylase beta chain, mitochondrial	PCCB	1.00		0.408426					NA	NA	NA	NA	0.41	NA		0.629301					NA	NA	NA	NA	0.63	NA
E9KL42	Epididymis secretory sperm binding protein Li 188n	NA	4.00		-0.38077					NA	NA	NA	NA	-0.38	NA		-0.29318					NA	NA	NA	NA	-0.29	NA
E9PC15	Acylglycerol kinase, mitochondrial	AGK	1.00		-2.50054					NA	NA	NA	NA	-2.50	NA		-0.97012					NA	NA	NA	NA	-0.97	NA
F5GWX2	Heme-binding protein 1	HEBP1	1.00		0.63972					NA	NA	NA	NA	0.64	NA		0.937401					NA	NA	NA	NA	0.94	NA
F5H365	Protein transport protein Sec23A	SEC23A	1.00		0.207564					NA	NA	NA	NA	0.21	NA		-0.5045					NA	NA	NA	NA	-0.50	NA
F6S8M0	N-acetylglucosamine-6-sulfatase	GNS	3.00		1.274772					NA	NA	NA	NA	1.27	NA		1.057033					NA	NA	NA	NA	1.06	NA
H0Y8C6	Importin-5 (Fragment)	IPO5	4.00		-1.08483					NA	NA	NA	NA	-1.08	NA		-0.91465					NA	NA	NA	NA	-0.91	NA
H3BTC0	RNA-binding protein with serine-rich domain 1 (Fragment)	RNPS1	1.00		0.137158					NA	NA	NA	NA	0.14	NA		-0.1467					NA	NA	NA	NA	-0.15	NA
H7C5R1	Ceruloplasmin (Fragment)	CP	2.00		-0.01642					NA	NA	NA	NA	-0.02	NA		0.356805					NA	NA	NA	NA	0.36	NA
K7EQA9	Hsp90 co-chaperone Cdc37 (Fragment)	CDC37	2.00		1.148763					NA	NA	NA	NA	1.15	NA		-2.55508					NA	NA	NA	NA	-2.56	NA
Q05DF2	SF3A2 protein (Fragment)	SF3A2	2.00		0.139917					NA	NA	NA	NA	0.14	NA		0.246152					NA	NA	NA	NA	0.25	NA
Q3LIE9	Predicted protein product of Nbla02942	Nbla02942	2.00		-0.74649					NA	NA	NA	NA	-0.75	NA		-0.68474					NA	NA	NA	NA	-0.68	NA
Q53FN7	BZW1 protein variant (Fragment)	NA	1.00		-0.55723					NA	NA	NA	NA	-0.56	NA		-0.74035					NA	NA	NA	NA	-0.74	NA
Q53FX5	Lin-7 homolog C variant (Fragment)	NA	1.00		0.31767					NA	NA	NA	NA	0.32	NA		-0.21821					NA	NA	NA	NA	-0.22	NA
Q53G61	Small nuclear ribonucleoprotein polypeptide A' variant (Fragment)	NA	1.00		1.142929					NA	NA	NA	NA	1.14	NA		1.353187					NA	NA	NA	NA	1.35	NA
Q53GI5	Adaptor-related protein complex 1, mu 2 subunit variant (Fragment)	NA	1.00		1.591339					NA	NA	NA	NA	1.59	NA		1.655208					NA	NA	NA	NA	1.66	NA
Q53HG7	Cortactin isoform a variant (Fragment)	NA	1.00		0.56816					NA	NA	NA	NA	0.57	NA		1.505543					NA	NA	NA	NA	1.51	NA
Q59F12	Protein 4.1 variant (Fragment)	NA	1.00		-0.70753					NA	NA	NA	NA	-0.71	NA		-0.77424					NA	NA	NA	NA	-0.77	NA
Q59GA1	Splicing factor, arginine/serine-rich 10 (Transformer 2 homolog, Drosophila) variant (Fragment)	NA	1.00		-2.0307					NA	NA	NA	NA	-2.03	NA		-2.02736					NA	NA	NA	NA	-2.03	NA
Q5SQT9	SAR1 gene homolog A (S. cerevisiae), isoform CRA_a	SAR1A	2.00		0.113314					NA	NA	NA	NA	0.11	NA		-0.01195					NA	NA	NA	NA	-0.01	NA
Q63HL4	NADPH--cytochrome P450 reductase	DKFZp686G04235	2.00		0.300047					NA	NA	NA	NA	0.30	NA		-0.33872					NA	NA	NA	NA	-0.34	NA
Q6FHG5	Gamma-synuclein	SNCG	3.00		-0.54306					NA	NA	NA	NA	-0.54	NA		-0.25245					NA	NA	NA	NA	-0.25	NA
Q6FIE9	TOLLIP protein	TOLLIP	2.00		-0.43286					NA	NA	NA	NA	-0.43	NA		-0.86886					NA	NA	NA	NA	-0.87	NA
Q6IAT2	GCSH protein	GCSH	3.00		0.656557					NA	NA	NA	NA	0.66	NA		0.149136					NA	NA	NA	NA	0.15	NA
Q6MZS5	Putative uncharacterized protein DKFZp686A13234 (Fragment)	DKFZp686A13234	8.00		0.502679					NA	NA	NA	NA	0.50	NA		0.5576					NA	NA	NA	NA	0.56	NA
Q6N095	Putative uncharacterized protein DKFZp686K03196	DKFZp686K03196	2.00		0.088177					NA	NA	NA	NA	0.09	NA		0.225945					NA	NA	NA	NA	0.23	NA
Q6P163	APOC2 protein	APOC2	1.00		0.5388					NA	NA	NA	NA	0.54	NA		-0.28269					NA	NA	NA	NA	-0.28	NA
Q6XCI7	SEC14-like protein 3	NA	1.00		0.093525					NA	NA	NA	NA	0.09	NA		-0.58089					NA	NA	NA	NA	-0.58	NA
Q8IW47	TMCC2 protein (Fragment)	TMCC2	1.00		-0.19091					NA	NA	NA	NA	-0.19	NA		-0.47756					NA	NA	NA	NA	-0.48	NA
Q8WUI6	COPG protein (Fragment)	COPG	2.00		0.99696					NA	NA	NA	NA	1.00	NA		1.034296					NA	NA	NA	NA	1.03	NA
Q96F88	Processing of 1, ribonuclease P/MRP subunit (S. cerevisiae)	POP1	1.00		0.547268					NA	NA	NA	NA	0.55	NA		1.00484					NA	NA	NA	NA	1.00	NA
V9HW41	Epididymis secretory protein Li 71	UBE2N	3.00		1.083527					NA	NA	NA	NA	1.08	NA		0.496278					NA	NA	NA	NA	0.50	NA
O75306	NADH dehydrogenase [ubiquinone] iron-sulfur protein 2, mitochondrial	NDUFS2	11.00			-0.73247				NA	NA	NA	NA	-0.73	NA			-0.67085				NA	NA	NA	NA	-0.67	NA
O95822	Malonyl-CoA decarboxylase, mitochondrial	MLYCD	1.00			-0.31743				NA	NA	NA	NA	-0.32	NA			-0.32948				NA	NA	NA	NA	-0.33	NA
P12429	Annexin A3	ANXA3	1.00			-1.47871				NA	NA	NA	NA	-1.48	NA			-1.26511				NA	NA	NA	NA	-1.27	NA
P20618	Proteasome subunit beta type-1	PSMB1	4.00			0.107729				NA	NA	NA	NA	0.11	NA			0.131718				NA	NA	NA	NA	0.13	NA
P28074	Proteasome subunit beta type-5	PSMB5	1.00			1.110678				NA	NA	NA	NA	1.11	NA			0.793296				NA	NA	NA	NA	0.79	NA
P82909	28S ribosomal protein S36, mitochondrial	MRPS36	1.00			-0.45417				NA	NA	NA	NA	-0.45	NA			0.443912				NA	NA	NA	NA	0.44	NA
Q13642	Four and a half LIM domains protein 1	FHL1	2.00			-0.41442				NA	NA	NA	NA	-0.41	NA			0.332367				NA	NA	NA	NA	0.33	NA
Q14689	Disco-interacting protein 2 homolog A	DIP2A	1.00			1.461238				NA	NA	NA	NA	1.46	NA			0.674493				NA	NA	NA	NA	0.67	NA
Q92538	Golgi-specific brefeldin A-resistance guanine nucleotide exchange factor 1	GBF1	2.00			-2.26298				NA	NA	NA	NA	-2.26	NA			-2.37897				NA	NA	NA	NA	-2.38	NA
Q9BT22	Chitobiosyldiphosphodolichol beta-mannosyltransferase	ALG1	1.00			0.527371				NA	NA	NA	NA	0.53	NA			0.000253				NA	NA	NA	NA	0.00	NA
Q9H7Z7	Prostaglandin E synthase 2	PTGES2	1.00			-0.70412				NA	NA	NA	NA	-0.70	NA			-0.6378				NA	NA	NA	NA	-0.64	NA
Q9NZZ3	Charged multivesicular body protein 5	CHMP5	1.00			1.361509				NA	NA	NA	NA	1.36	NA			1.24271				NA	NA	NA	NA	1.24	NA
Q9UBV2	Protein sel-1 homolog 1	SEL1L	1.00			-0.27223				NA	NA	NA	NA	-0.27	NA			-0.6319				NA	NA	NA	NA	-0.63	NA
A0A024QZT4	Serpin peptidase inhibitor, clade B (Ovalbumin), member 9, isoform CRA_a	SERPINB9	3.00			1.479983				NA	NA	NA	NA	1.48	NA			0.926945				NA	NA	NA	NA	0.93	NA

UniProt	Protein name	Gene name	Peptide mean	08/017 hESC-RPE to hRPE												08/023 hESC-RPE to hRPE											
				Analysis						Technical replicate CV			Biological	Fold change (mean)	Unadjusted p-value	Analysis						Technical replicate CV			Biological	Fold change (mean)	Unadjusted p-value
				1.1	1.2	2.1	2.2	3.1	3.2	1	2	3	CV		(t-test)	1.1	1.2	2.1	2.2	3.1	3.2	1	2	3	CV		(t-test)
A0A024R2D5	alpha-1,2-Mannosidase	EDEM1	1.00			0.680588				NA	NA	NA	NA	0.68	NA			-0.78744				NA	NA	NA	NA	-0.79	NA
A0A024R843	Tripartite motif-containing 32, isoform CRA_a	TRIM32	2.00			3.202365				NA	NA	NA	NA	3.20	NA			2.616294				NA	NA	NA	NA	2.62	NA
A0A024RA40	Carboxypeptidase, vitellogenic-like, isoform CRA_a	CPVL	1.00			1.082543				NA	NA	NA	NA	1.08	NA			0.940194				NA	NA	NA	NA	0.94	NA
A0A087X1C7	Ig gamma-1 chain C region	IGHG1	6.00			-1.2216				NA	NA	NA	NA	-1.22	NA			-1.15507				NA	NA	NA	NA	-1.16	NA
A0A087X1N8	Serpin B6	SERPINB6	1.00			-1.07591				NA	NA	NA	NA	-1.08	NA			-0.97765				NA	NA	NA	NA	-0.98	NA
A0A0A0MT30	Aldo-keto reductase family 1 member C1	AKR1C1	1.00			-1.70801				NA	NA	NA	NA	-1.71	NA			-1.37502				NA	NA	NA	NA	-1.38	NA
A0A0C4DGQ8	Dehydrogenase/reductase SDR family member 7B	DHRS7B	2.00			0.183205				NA	NA	NA	NA	0.18	NA			1.23499				NA	NA	NA	NA	1.23	NA
A8K103	cDNA FLJ75454, highly similar to Homo sapiens arrestin, beta 1 (ARRB1), transcript variant 1, mRNA	NA	3.00			0.048989				NA	NA	NA	NA	0.05	NA			-1.4042				NA	NA	NA	NA	-1.40	NA
A8K8B9	cDNA FLJ77368, highly similar to Homo sapiens protein kinase, cAMP-dependent, catalytic, alpha (PRKACA	NA	1.00			-0.89055				NA	NA	NA	NA	-0.89	NA			-0.70675				NA	NA	NA	NA	-0.71	NA
A8K8W7	cDNA FLJ75224, highly similar to Homo sapiens lipase, hormone-sensitive (LIPE), mRNA	NA	2.00			0.727125				NA	NA	NA	NA	0.73	NA			0.815035				NA	NA	NA	NA	0.82	NA
B2R4W8	HCG1994130, isoform CRA_a	hCG_1994130	2.00			1.644672				NA	NA	NA	NA	1.64	NA			0.460558				NA	NA	NA	NA	0.46	NA
B2R657	Annexin	NA	3.00			-0.117				NA	NA	NA	NA	-0.12	NA			-0.32693				NA	NA	NA	NA	-0.33	NA
B2R6Y1	Histone H3	NA	5.00			-2.35355				NA	NA	NA	NA	-2.35	NA			-2.4869				NA	NA	NA	NA	-2.49	NA
B2RAQ8	cDNA, FLJ95058, highly similar to Homo sapiens carnitine palmitoyltransferase 1A (liver) (CPT1A),nuclear g	NA	1.00			-0.01519				NA	NA	NA	NA	-0.02	NA			-0.22522				NA	NA	NA	NA	-0.23	NA
B2RB13	cDNA, FLJ95253, highly similar to Homo sapiens acyl-CoA synthetase long-chain family member 6(ACSL6), r	NA	3.00			-0.43828				NA	NA	NA	NA	-0.44	NA			-0.15563				NA	NA	NA	NA	-0.16	NA
B3KTQ2	cDNA FLJ38589 fis, clone HCHON2010074, highly similar to LACTADHERIN	NA	5.00			0.709079				NA	NA	NA	NA	0.71	NA			1.094635				NA	NA	NA	NA	1.09	NA
B3KVA7	cDNA FLJ16309 fis, clone SKMUS2007816, highly similar to Creatine kinase, sarcomeric mitochondrial (EC 2	NA	7.00			-0.85033				NA	NA	NA	NA	-0.85	NA			-0.17779				NA	NA	NA	NA	-0.18	NA
B4DE32	cDNA FLJ53970, highly similar to ATP-dependent DNA helicase 2 subunit 1 (EC3.6.1.-)	NA	10.00			0.397772				NA	NA	NA	NA	0.40	NA			0.059976				NA	NA	NA	NA	0.06	NA
B4DFP1	cDNA FLJ51818, highly similar to Phosphoglucomutase-1 (EC 5.4.2.2)	NA	4.00			0.156106				NA	NA	NA	NA	0.16	NA			0.235528				NA	NA	NA	NA	0.24	NA
B4DN86	cDNA FLJ56047, highly similar to A kinase anchor protein 1, mitochondrial	NA	1.00			-0.72019				NA	NA	NA	NA	-0.72	NA			-0.1472				NA	NA	NA	NA	-0.15	NA
B4DSN2	cDNA FLJ52740, highly similar to Magnesium transporter MRS2L, mitochondrial	NA	1.00			-2.56156				NA	NA	NA	NA	-2.56	NA			-1.76018				NA	NA	NA	NA	-1.76	NA
B4DUT8	Calponin	CNN2	1.00			0.323253				NA	NA	NA	NA	0.32	NA			0.734129				NA	NA	NA	NA	0.73	NA
B4DVE1	cDNA FLJ53478, highly similar to Galectin-3-binding protein	NA	6.00			0.757702				NA	NA	NA	NA	0.76	NA			0.54814				NA	NA	NA	NA	0.55	NA
B4DWH5	cDNA FLJ53224, highly similar to Calpain-1 catalytic subunit (EC 3.4.22.52)	NA	2.00			0.3032				NA	NA	NA	NA	0.30	NA			0.311923				NA	NA	NA	NA	0.31	NA
B4DWS6	cDNA FLJ61181, highly similar to Homo sapiens hydroxysteroid (17-beta) dehydrogenase 12 (HSD17B12), m	NA	2.00			0.41795				NA	NA	NA	NA	0.42	NA			0.21627				NA	NA	NA	NA	0.22	NA
B4DZR0	cDNA FLJ55529, highly similar to Heat shock 70 kDa protein 4L	NA	1.00			0.68587				NA	NA	NA	NA	0.69	NA			0.123146				NA	NA	NA	NA	0.12	NA
B4E299	cDNA FLJ54574, highly similar to Staphylococcal nuclease domain-containing protein 1	NA	4.00			0.57974				NA	NA	NA	NA	0.58	NA			0.43832				NA	NA	NA	NA	0.44	NA
B5MBW9	Coiled-coil-helix-coiled-coil-helix domain-containing protein 10, mitochondrial	CHCHD10	1.00			-0.67972				NA	NA	NA	NA	-0.68	NA			-1.14664				NA	NA	NA	NA	-1.15	NA
B7Z3K9	Fructose-bisphosphate aldolase	NA	28.00			-2.10185				NA	NA	NA	NA	-2.10	NA			-2.16752				NA	NA	NA	NA	-2.17	NA
B7Z653	cDNA FLJ61657, highly similar to Band 4.1-like protein 1	NA	1.00			0.253372				NA	NA	NA	NA	0.25	NA			0.57913				NA	NA	NA	NA	0.58	NA
B7Z6L0	Annexin	NA	3.00			0.783647				NA	NA	NA	NA	0.78	NA			0.084766				NA	NA	NA	NA	0.08	NA
B7Z8W8	Reticulon	NA	1.00			-0.17987				NA	NA	NA	NA	-0.18	NA			-0.64569				NA	NA	NA	NA	-0.65	NA
C9J168	Phosphoribosyl pyrophosphate synthase-associated protein 1 (Fragment)	PRPSAP1	1.00			-0.03923				NA	NA	NA	NA	-0.04	NA			-0.20729				NA	NA	NA	NA	-0.21	NA
C9J3D7	Peroxisomal carnitine O-octanoyltransferase	CROT	1.00			-0.66367				NA	NA	NA	NA	-0.66	NA			-0.41394				NA	NA	NA	NA	-0.41	NA
C9JA28	Translocon-associated protein subunit gamma	SSR3	1.00			0.729365				NA	NA	NA	NA	0.73	NA			1.033394				NA	NA	NA	NA	1.03	NA
D3DTC2	Acyltransferase like 2, isoform CRA_a	AYTL2	2.00			-0.73115				NA	NA	NA	NA	-0.73	NA			-0.21314				NA	NA	NA	NA	-0.21	NA
D3DUW7	Dynamin 1-like, isoform CRA_a	DNM1L	1.00			0.207281				NA	NA	NA	NA	0.21	NA			0.325113				NA	NA	NA	NA	0.33	NA
E5RJZ7	Carboxypeptidase Q (Fragment)	CPQ	1.00			0.84617				NA	NA	NA	NA	0.85	NA			0.76885				NA	NA	NA	NA	0.77	NA
E9PFW3	AP-2 complex subunit mu	AP2M1	3.00			0.714581				NA	NA	NA	NA	0.71	NA			0.960883				NA	NA	NA	NA	0.96	NA
E9PRB9	Signal peptidase complex subunit 2	SPCS2	1.00			0.68587				NA	NA	NA	NA	0.69	NA			0.40965				NA	NA	NA	NA	0.41	NA
F5GXQ8	Nesprin-1	SYNE1	1.00			3.41522				NA	NA	NA	NA	3.42	NA			2.191055				NA	NA	NA	NA	2.19	NA
F5H5D3	Tubulin alpha-1C chain	TUBA1C	44.00			-0.68269				NA	NA	NA	NA	-0.68	NA			-0.88454				NA	NA	NA	NA	-0.88	NA
F6WQW2	Ran-specific GTPase-activating protein	RANBP1	1.00			0.042055				NA	NA	NA	NA	0.04	NA			0.147111				NA	NA	NA	NA	0.15	NA
F8VS27	Elongation factor Ts	TSFM	1.00			0.608188				NA	NA	NA	NA	0.61	NA			1.13656				NA	NA	NA	NA	1.14	NA
H0YCS0	Beta-2-syntrophin (Fragment)	SNTB2	1.00			2.861058				NA	NA	NA	NA	2.86	NA			2.074228				NA	NA	NA	NA	2.07	NA
H3BQI1	Dynein light chain roadblock-type 2	DYNLRB2	1.00			-0.50405				NA	NA	NA	NA	-0.50	NA			0.020104				NA	NA	NA	NA	0.02	NA
I3L3T0	HCG15164, isoform CRA_b	PAM16	1.00			-0.16523				NA	NA	NA	NA	-0.17	NA			0.07615				NA	NA	NA	NA	0.08	NA
J3KNC0	Transcription initiation factor IIA subunit 1	GTF2A1	2.00			0.196913				NA	NA	NA	NA	0.20	NA			0.353912				NA	NA	NA	NA	0.35	NA
J3KTL2	Serine/arginine-rich-splicing factor 1	SRSF1	4.00			0.499707				NA	NA	NA	NA	0.50	NA			0.680821				NA	NA	NA	NA	0.68	NA
L8B4J3	Ubiquitin C	UbC	4.00			0.409594				NA	NA	NA	NA	0.41	NA			0.318837				NA	NA	NA	NA	0.32	NA
L8E7F4	Alternative protein SP1	SP1	1.00			2.396917				NA	NA	NA	NA	2.40	NA			1.972472				NA	NA	NA	NA	1.97	NA
M0QYT0	Uncharacterized protein (Fragment)	NA	5.00			-0.04168				NA	NA	NA	NA	-0.04	NA			0.089055				NA	NA	NA	NA	0.09	NA
M0R2S2	Epidermal growth factor receptor substrate 15-like 1	EPS15L1	1.00			0.214838				NA	NA	NA	NA	0.21	NA			-0.31366				NA	NA	NA	NA	-0.31	NA
Q05CW6	PSMD1 protein (Fragment)	PSMD1	1.00			0.038704				NA	NA	NA	NA	0.04	NA			-0.17065				NA	NA	NA	NA	-0.17	NA
Q0IJ56	ST13 protein (Fragment)	ST13	3.00			0.097113				NA	NA	NA	NA	0.10	NA			0.035				NA	NA	NA	NA	0.04	NA
Q1LZN2	NOMO3 protein (Fragment)	NOMO3	1.00			0.817279				NA	NA	NA	NA	0.82	NA			0.942303				NA	NA	NA	NA	0.94	NA
Q53FG3	Interleukin enhancer binding factor 2 variant (Fragment)	NA	3.00			1.326955				NA	NA	NA	NA	1.33	NA			1.092382				NA	NA	NA	NA	1.09	NA
Q53G72	B-cell receptor-associated protein 31 variant (Fragment)	NA	2.00			0.042312				NA	NA	NA	NA	0.04	NA			0.871729				NA	NA	NA	NA	0.87	NA
Q53H34	Ribosomal protein L13a variant (Fragment)	NA	2.00			0.376288				NA	NA	NA	NA	0.38	NA			0.861459				NA	NA	NA	NA	0.86	NA
Q53HM6	Splicing factor 3a, subunit 3 variant (Fragment)	NA	1.00			-0.34983				NA	NA	NA	NA	-0.35	NA			-0.21579				NA	NA	NA	NA	-0.22	NA
Q53R19	Actin related protein 2/3 complex, subunit 2, 34kDa, isoform CRA_a	ARPC2	1.00			0.332123				NA	NA	NA	NA	0.33	NA			-0.01475				NA	NA	NA	NA	-0.01	NA
Q53T76	Glycerol-3-phosphate dehydrogenase (Fragment)	GPD2	2.00			1.154797				NA	NA	NA	NA	1.15	NA			1.503408				NA	NA	NA	NA	1.50	NA
Q59E88	DnaJ (Hsp40) homolog, subfamily A, member 3 variant (Fragment)	NA	2.00			0.054359				NA	NA	NA	NA	0.05	NA			0.098067				NA	NA	NA	NA	0.10	NA
Q59FN3	Acyl-Coenzyme A dehydrogenase family, member 9 variant (Fragment)	NA	1.00			-0.06118				NA	NA	NA	NA	-0.06	NA			-0.07212				NA	NA	NA	NA	-0.07	NA
Q5M7Z9	TARS protein (Fragment)	TARS	1.00			-0.17657				NA	NA	NA	NA	-0.18	NA			-0.01911				NA	NA	NA	NA	-0.02	NA
Q5QP19	NFS1 nitrogen fixation 1 (S. cerevisiae), isoform CRA_b	NFS1	1.00			0.698175				NA	NA	NA	NA	0.70	NA			-0.07372				NA	NA	NA	NA	-0.07	NA
Q5QPE2	Protein transport protein Sec23B (Fragment)	SEC23B	2.00			-0.26617				NA	NA	NA	NA	-0.27	NA			0.079028				NA	NA	NA	NA	0.08	NA
Q5R3B4	Mitochondrial pyruvate carrier 2 (Fragment)	MPC2	1.00			-0.49692				NA	NA	NA	NA	-0.50	NA			-0.98815				NA	NA	NA	NA	-0.99	NA
Q5STU3	Spliceosome RNA helicase DDX39B	DDX39B	5.00			0.270173				NA	NA	NA	NA	0.27	NA			-0.04133				NA	NA	NA	NA	-0.04	NA
Q5U0J0	E2F transcription factor 2	NA	1.00			0.964038				NA	NA	NA	NA	0.96	NA			1.66734				NA	NA	NA	NA	1.67	NA
Q6FGI7	COX7A1 protein	COX7A1	1.00			-2.37127				NA	NA	NA	NA	-2.37	NA			-0.33841				NA	NA	NA	NA	-0.34	NA
Q6IBN4	PECI protein	PECI	1.00			0.322829				NA	NA	NA	NA	0.32	NA			0.198517				NA	NA	NA	NA	0.20	NA
Q6IPH7	RPL14 protein	RPL14	1.00			0.097113				NA	NA	NA	NA	0.10	NA			0.097121				NA	NA	NA	NA	0.10	NA
Q6LEU9	ATP5G3 protein (Fragment)	ATP5G3	1.00			-2.27191				NA	NA	NA	NA	-2.27	NA			-1.87895				NA	NA	NA	NA	-1.88	NA
Q6MZM7	Putative uncharacterized protein DKFZp686O12165 (Fragment)	DKFZp686O12165	3.00			0.736541				NA	NA	NA	NA	0.74	NA			2.056574				NA	NA	NA	NA	2.06	NA
Q6ZMU0	Delta-aminolevulinic acid dehydratase	NA	2.00			0.668965				NA	NA	NA	NA	0.67	NA			0.923613				NA	NA	NA	NA	0.92	NA
Q6ZR81	cDNA FLJ46566 fis, clone THYMU3040829, moderately similar to Cold-inducible RNA-binding protein	NA	1.00			-2.01358				NA	NA	NA	NA	-2.01	NA			-0.95491				NA	NA	NA	NA	-0.95	NA
Q71U02	Regulatory myosin light chain long version	NA	4.00			0.490114				NA	NA	NA	NA	0.49	NA			0.976014				NA	NA	NA	NA	0.98	NA
Q7LD69	NADH-ubiquinone oxidoreductase Fe-S protein 7 variant	NA	4.00			-0.50593				NA	NA	NA	NA	-0.51	NA			-0.7705				NA	NA	NA	NA	-0.77	NA
Q86U62	Proteasome subunit beta type	NA	2.00			0.699155				NA	NA	NA	NA	0.70	NA			0.350343				NA	NA	NA	NA	0.35	NA
Q8IWR8	Ribosomal protein L19 (Fragment)	NA	1.00			1.142603				NA	NA	NA	NA	1.14	NA			1.800433				NA	NA	NA	NA	1.80	NA
Q8N652	Guanine nucleotide binding protein (G protein), alpha z polypeptide	GNAZ	1.00			-0.25822				NA	NA	NA	NA	-0.26	NA			0.539113				NA	NA	NA	NA	0.54	NA
Q8N9M2	cDNA FLJ36887 fis, clone BNGH42005504, highly similar to 26S PROTEASOME REGULATORY SUBUNIT S3	NA	2.00			-0.44476				NA	NA	NA	NA	-0.44	NA			-0.28255				NA	NA	NA	NA	-0.28	NA
Q8TCD0	Uncharacterized protein	NA	2.00			-1.26044				NA	NA	NA	NA	-1.26	NA			-1.5913				NA	NA	NA	NA	-1.59	NA
Q96BA7	HNRPU protein	NA	2.00			-0.21051				NA	NA	NA	NA	-0.21	NA			0.011343				NA	NA	NA	NA	0.01	NA
Q96CP5	PMPCB protein (Fragment)	PMPCB	2.00			0.03793				NA	NA	NA	NA	0.04	NA			0.301283				NA	NA	NA	NA	0.30	NA
Q9BPW0	Serine/threonine-protein phosphatase (Fragment)	PPP5C	1.00			-0.22036				NA	NA	NA	NA	-0.22	NA			0.572339				NA	NA	NA	NA	0.57	NA
Q9BTQ7	Similar to ribosomal protein L23 (Fragment)	NA	1.00			0.229833				NA	NA	NA	NA	0.23	NA			-0.00532				NA	NA	NA	NA	-0.01	NA
Q9BV61	TRAP1 protein (Fragment)	TRAP1	3.00			-0.83181				NA	NA	NA	NA	-0.83	NA			-0.30676				NA	NA	NA	NA	-0.31	NA
Q9NVF8	cDNA FLJ10762 fis, clone NT2RP4000008, moderately similar to CHLORINE CHANNEL PROTEIN P64	NA	4.00			0.716518				NA	NA	NA	NA	0.72	NA			0.835175				NA	NA	NA	NA	0.84	NA
V9HW09	Epididymis secretory sperm binding protein Li 91n	PSMD8	1.00			-1.43792				NA	NA	NA	NA	-1.44	NA			-1.37437				NA	NA	NA	NA	-1.37	NA
V9HW51	Epididymis secretory protein Li 114	TXNL1	1.00			0.431378				NA	NA	NA	NA	0.43	NA			1.070859				NA	NA	NA	NA	1.07	NA
V9HW83	Aldehyde dehydrogenase 1 family, member A1, isoform CRA_a	ALDH1A1	2.00			0.10945				NA	NA	NA	NA	0.11	NA			0.518238				NA	NA	NA	NA	0.52	NA
V9HWD3	Epididymis luminal protein 117	ERLEC1	1.00			-0.92034				NA	NA	NA	NA	-0.92	NA			-1.05021				NA	NA	NA	NA	-1.05	NA

				08/017 hESC-RPE to hRPE												08/023 hESC-RPE to hRPE											
				Analysis						Technical replicate CV			Biological	Fold change (mean)	Unadjusted p-value	Analysis						Technical replicate CV			Biological	Fold change (mean)	Unadjusted p-value
UniProt	Protein name	Gene name	Peptide mean	1.1	1.2	2.1	2.2	3.1	3.2	1	2	3	CV		(t-test)	1.1	1.2	2.1	2.2	3.1	3.2	1	2	3	CV		(t-test)
O75506	Heat shock factor-binding protein 1	HSBP1	1.00				0.189813			NA	NA	NA	NA	0.19	NA				-0.55512			NA	NA	NA	NA	-0.56	NA
P20933	N(4)-(beta-N-acetylglucosaminyl)-L-asparaginase	AGA	1.00				1.769295			NA	NA	NA	NA	1.77	NA				1.35629			NA	NA	NA	NA	1.36	NA
P24311	Cytochrome c oxidase subunit 7B, mitochondrial	COX7B	1.00				-4.49912			NA	NA	NA	NA	-4.50	NA				-1.16589			NA	NA	NA	NA	-1.17	NA
P26599	Polypyrimidine tract-binding protein 1	PTBP1	2.00				1.070488			NA	NA	NA	NA	1.07	NA				0.940365			NA	NA	NA	NA	0.94	NA
P32929	Cystathionine gamma-lyase	CTH	1.00				0.224974			NA	NA	NA	NA	0.22	NA				-0.20696			NA	NA	NA	NA	-0.21	NA
P46783	40S ribosomal protein S10	RPS10	1.00				-0.0607			NA	NA	NA	NA	-0.06	NA				-0.22956			NA	NA	NA	NA	-0.23	NA
P46939	Utrophin	UTRN	1.00				3.076876			NA	NA	NA	NA	3.08	NA				2.191714			NA	NA	NA	NA	2.19	NA
P61599	N-alpha-acetyltransferase 20	NAA20	2.00				3.839518			NA	NA	NA	NA	3.84	NA				3.092398			NA	NA	NA	NA	3.09	NA
P62263	40S ribosomal protein S14	RPS14	1.00				-0.80097			NA	NA	NA	NA	-0.80	NA				-0.01497			NA	NA	NA	NA	-0.01	NA
Q13825	Methylglutaconyl-CoA hydratase, mitochondrial	AUH	1.00				0.179141			NA	NA	NA	NA	0.18	NA				0.367972			NA	NA	NA	NA	0.37	NA
Q15120	[Pyruvate dehydrogenase (acetyl-transferring)] kinase isozyme 3, mitochondrial	PDK3	1.00				-1.35593			NA	NA	NA	NA	-1.36	NA				-0.7493			NA	NA	NA	NA	-0.75	NA
Q5VV17	OTU domain-containing protein 1	OTUD1	1.00				3.573741			NA	NA	NA	NA	3.57	NA				3.766829			NA	NA	NA	NA	3.77	NA
Q86SX6	Glutaredoxin-related protein 5, mitochondrial	GLRX5	1.00				-0.6187			NA	NA	NA	NA	-0.62	NA				0.375578			NA	NA	NA	NA	0.38	NA
Q8TB36	Ganglioside-induced differentiation-associated protein 1	GDAP1	2.00				-0.18886			NA	NA	NA	NA	-0.19	NA				-0.40623			NA	NA	NA	NA	-0.41	NA
Q96HN2	Adenosylhomocysteinase 3	AHCYL2	2.00				0.553025			NA	NA	NA	NA	0.55	NA				0.672034			NA	NA	NA	NA	0.67	NA
Q9H2G2	STE20-like serine/threonine-protein kinase	SLK	1.00				0.889079			NA	NA	NA	NA	0.89	NA				0.172922			NA	NA	NA	NA	0.17	NA
Q9HBJ8	Collectrin	TMEM27	1.00				-0.73367			NA	NA	NA	NA	-0.73	NA				-1.97127			NA	NA	NA	NA	-1.97	NA
Q9HCC0	Methylcrotonoyl-CoA carboxylase beta chain, mitochondrial	MCCC2	2.00				-0.15883			NA	NA	NA	NA	-0.16	NA				-0.25433			NA	NA	NA	NA	-0.25	NA
Q9UHG3	Prenylcysteine oxidase 1	PCYOX1	1.00				-0.74335			NA	NA	NA	NA	-0.74	NA				-1.419			NA	NA	NA	NA	-1.42	NA
Q9UJU6	Drebrin-like protein	DBNL	2.00				0.783685			NA	NA	NA	NA	0.78	NA				1.503376			NA	NA	NA	NA	1.50	NA
Q9ULD0	2-oxoglutarate dehydrogenase-like, mitochondrial	OGDHL	6.00				-0.49986			NA	NA	NA	NA	-0.50	NA				-0.18355			NA	NA	NA	NA	-0.18	NA
Q9UMX5	Neudesin	NENF	2.00				0.624876			NA	NA	NA	NA	0.62	NA				0.884186			NA	NA	NA	NA	0.88	NA
A0A024R4E5	High density lipoprotein binding protein (Vigilin), isoform CRA_a	HDLBP	1.00				-0.78771			NA	NA	NA	NA	-0.79	NA				-1.15911			NA	NA	NA	NA	-1.16	NA
A0A024R5Z7	Annexin	ANXA2	23.00				0.066088			NA	NA	NA	NA	0.07	NA				0.50106			NA	NA	NA	NA	0.50	NA
A0A024R7N7	Interferon, gamma-inducible protein 30, isoform CRA_b	IFI30	3.00				1.045417			NA	NA	NA	NA	1.05	NA				-0.15209			NA	NA	NA	NA	-0.15	NA
A0A024RAJ9	Arylsulfatase B, isoform CRA_a	ARSB	1.00				1.377248			NA	NA	NA	NA	1.38	NA				-0.71038			NA	NA	NA	NA	-0.71	NA
A0A087WT95	Acyl-coenzyme A thioesterase 2, mitochondrial	ACOT2	7.00				-0.17719			NA	NA	NA	NA	-0.18	NA				-0.20114			NA	NA	NA	NA	-0.20	NA
A0A0A1HAN9	H.sapiens ras-related Hrab5 protein	NA	2.00				1.177278			NA	NA	NA	NA	1.18	NA				0.303797			NA	NA	NA	NA	0.30	NA
A0A0A6YYL4	Coronin	CORO7-PAM16	1.00				0.181236			NA	NA	NA	NA	0.18	NA				-0.09788			NA	NA	NA	NA	-0.10	NA
A0A0C4DFQ1	Metaxin-1	MTX1	1.00				-0.90425			NA	NA	NA	NA	-0.90	NA				-0.33161			NA	NA	NA	NA	-0.33	NA
A0A0C4DGS0	NADH dehydrogenase [ubiquinone] 1 alpha subcomplex subunit 6	NDUFA6	2.00				-1.1068			NA	NA	NA	NA	-1.11	NA				-1.16533			NA	NA	NA	NA	-1.17	NA
A0A0J9YXC7	LIM and senescent cell antigen-like-containing domain protein 3	LIMS3L	1.00				0.272891			NA	NA	NA	NA	0.27	NA				0.740847			NA	NA	NA	NA	0.74	NA
A6NLM8	Translocon-associated protein subunit delta	SSR4	2.00				0.395601			NA	NA	NA	NA	0.40	NA				0.270711			NA	NA	NA	NA	0.27	NA
A8K7G2	cDNA FLJ75762, highly similar to Homo sapiens protease, serine, 25 (PRSS25), nuclear gene encodingmitoc	NA	2.00				-0.46487			NA	NA	NA	NA	-0.46	NA				0.050095			NA	NA	NA	NA	0.05	NA
A8K940	cDNA FLJ77630, highly similar to Homo sapiens BPY2 interacting protein 1, mRNA	NA	1.00				0.545458			NA	NA	NA	NA	0.55	NA				0.27092			NA	NA	NA	NA	0.27	NA
B2R5W3	cDNA, FLJ92658, highly similar to Homo sapiens poly (ADP-ribose) polymerase family, member 1 (PARP1), r	NA	3.00				0.330632			NA	NA	NA	NA	0.33	NA				0.794791			NA	NA	NA	NA	0.79	NA
B2R6F3	Splicing factor arginine/serine-rich 3	SFRS3	1.00				0.911536			NA	NA	NA	NA	0.91	NA				-0.57453			NA	NA	NA	NA	-0.57	NA
B2R6K1	cDNA, FLJ92992, highly similar to Homo sapiens methylmalonyl Coenzyme A mutase (MUT), nuclear gene e	NA	3.00				1.262599			NA	NA	NA	NA	1.26	NA				0.794645			NA	NA	NA	NA	0.79	NA
B2R6N6	cDNA, FLJ93036, highly similar to Homo sapiens tyrosine 3-monooxygenase/tryptophan 5-monooxygenase	NA	4.00				-0.27078			NA	NA	NA	NA	-0.27	NA				-0.78451			NA	NA	NA	NA	-0.78	NA
B2RE88	cDNA, FLJ96465, highly similar to Homo sapiens solute carrier family 25 (mitochondrial carrier;phosphate c	NA	7.00				-2.04403			NA	NA	NA	NA	-2.04	NA				-1.9565			NA	NA	NA	NA	-1.96	NA
B3KP89	cDNA FLJ31446 fis, clone NT2NE2000909, highly similar to Guanine nucleotide-binding protein G(o) subuni	NA	8.00				-0.00435			NA	NA	NA	NA	0.00	NA				-0.52394			NA	NA	NA	NA	-0.52	NA
B4DIU3	cDNA FLJ61278, highly similar to Protein-arginine deiminase type-2 (EC 3.5.3.15)	NA	1.00				-0.44321			NA	NA	NA	NA	-0.44	NA				-0.41226			NA	NA	NA	NA	-0.41	NA
B4DKS0	cDNA FLJ53381, highly similar to Monocarboxylate transporter 1	NA	2.00				0.171905			NA	NA	NA	NA	0.17	NA				2.008241			NA	NA	NA	NA	2.01	NA
B4DM97	cDNA FLJ55002, highly similar to Alpha-centractin	NA	2.00				0.238892			NA	NA	NA	NA	0.24	NA				0.774429			NA	NA	NA	NA	0.77	NA
B4DPM7	cDNA FLJ55739, highly similar to 26S proteasome non-ATPase regulatory subunit 3	NA	1.00				0.258198			NA	NA	NA	NA	0.26	NA				-0.56965			NA	NA	NA	NA	-0.57	NA
B4DPR2	cDNA FLJ50830, highly similar to Serum albumin	NA	72.00				2.321009			NA	NA	NA	NA	2.32	NA				0.645501			NA	NA	NA	NA	0.65	NA
B4DPZ3	cDNA FLJ53290, highly similar to Cytoplasmic dynein 1 intermediate chain 2	NA	2.00				-1.08036			NA	NA	NA	NA	-1.08	NA				0.766737			NA	NA	NA	NA	0.77	NA
B4DU60	cDNA FLJ51306, highly similar to Homo sapiens citrate lyase beta like (CLYBL), transcript variant 2, mRNA	NA	1.00				1.332308			NA	NA	NA	NA	1.33	NA				0.669339			NA	NA	NA	NA	0.67	NA
B4DZT2	cDNA FLJ57683, highly similar to Signal transducing adapter molecule 1	NA	1.00				0.087832			NA	NA	NA	NA	0.09	NA				0.092131			NA	NA	NA	NA	0.09	NA
B7Z6C2	cDNA FLJ50663, highly similar to Phosphoglucomutase-1 (EC 5.4.2.2)	NA	5.00				0.703877			NA	NA	NA	NA	0.70	NA				0.393363			NA	NA	NA	NA	0.39	NA
B7Z6K0	cDNA FLJ53199, highly similar to E3 ubiquitin-protein ligase NEDD4-like protein (EC 6.3.2.-)	NA	1.00				1.349687			NA	NA	NA	NA	1.35	NA				0.037119			NA	NA	NA	NA	0.04	NA
B7ZAY4	cDNA, FLJ79348, highly similar to Cell division control protein 42 homolog	NA	2.00				-0.33284			NA	NA	NA	NA	-0.33	NA				-0.23193			NA	NA	NA	NA	-0.23	NA
B7ZLX8	Oxysterol-binding protein	OSBPL1A	1.00				-1.0865			NA	NA	NA	NA	-1.09	NA				-1.29989			NA	NA	NA	NA	-1.30	NA
D3DSM7	Collagen, type VI, alpha 1, isoform CRA_a	COL6A1	1.00				1.311428			NA	NA	NA	NA	1.31	NA				3.41914			NA	NA	NA	NA	3.42	NA
D3DV26	S100 calcium binding protein A10 (Annexin II ligand, calpactin I, light polypeptide (P11)), isoform CRA_b (F	S100A10	1.00				-1.83396			NA	NA	NA	NA	-1.83	NA				-1.45383			NA	NA	NA	NA	-1.45	NA
D6RAZ4	Calmegin (Fragment)	CLGN	1.00				0.372165			NA	NA	NA	NA	0.37	NA				0.234276			NA	NA	NA	NA	0.23	NA
E5RJU9	Protein LYRIC	MTDH	1.00				-0.0443			NA	NA	NA	NA	-0.04	NA				-0.25795			NA	NA	NA	NA	-0.26	NA
E9PAM4	Uncharacterized protein	NA	1.00				0.689237			NA	NA	NA	NA	0.69	NA				-0.01242			NA	NA	NA	NA	-0.01	NA
E9PE82	Short-chain-specific acyl-CoA dehydrogenase, mitochondrial	ACADS	1.00				2.113889			NA	NA	NA	NA	2.11	NA				1.493462			NA	NA	NA	NA	1.49	NA
E9PLN8	Uncharacterized protein (Fragment)	NA	1.00				1.258198			NA	NA	NA	NA	1.26	NA				1.281236			NA	NA	NA	NA	1.28	NA
F4ZW66	NF110b	NA	3.00				0.527281			NA	NA	NA	NA	0.53	NA				0.641462			NA	NA	NA	NA	0.64	NA
F8WDD7	Actin-related protein 2/3 complex subunit 4	ARPC4	1.00				0.189813			NA	NA	NA	NA	0.19	NA				0.67425			NA	NA	NA	NA	0.67	NA
F8WDI0	Ubiquitin-like-conjugating enzyme ATG3	ATG3	1.00				-0.13373			NA	NA	NA	NA	-0.13	NA				-0.63399			NA	NA	NA	NA	-0.63	NA
G5E9F5	MpV17 transgene, murine homolog, glomerulosclerosis, isoform CRA_d	MPV17	1.00				0.09229			NA	NA	NA	NA	0.09	NA				-3.04225			NA	NA	NA	NA	-3.04	NA
H0UID5	AP complex subunit beta	AP2B1	3.00				0.257536			NA	NA	NA	NA	0.26	NA				0.135277			NA	NA	NA	NA	0.14	NA
H0YBX6	Ubiquitin-conjugating enzyme E2 variant 2 (Fragment)	UBE2V2	1.00				0.817586			NA	NA	NA	NA	0.82	NA				0.642594			NA	NA	NA	NA	0.64	NA
H0YJG7	Activator of 90 kDa heat shock protein ATPase homolog 1 (Fragment)	AHSA1	1.00				0.964914			NA	NA	NA	NA	0.96	NA				0.051313			NA	NA	NA	NA	0.05	NA
H0YKI0	Deoxyuridine 5'-triphosphate nucleotidohydrolase, mitochondrial	DUT	1.00				0.962887			NA	NA	NA	NA	0.96	NA				0.092368			NA	NA	NA	NA	0.09	NA
H3BM30	Trans-2-enoyl-CoA reductase, mitochondrial (Fragment)	MECR	1.00				0.150214			NA	NA	NA	NA	0.15	NA				-0.33894			NA	NA	NA	NA	-0.34	NA
H7BXZ6	Mitochondrial Rho GTPase	RHOT1	2.00				-0.5407			NA	NA	NA	NA	-0.54	NA				0.126519			NA	NA	NA	NA	0.13	NA
K7EQ77	NADH dehydrogenase [ubiquinone] 1 alpha subcomplex subunit 11	NDUFA11	1.00				-0.38009			NA	NA	NA	NA	-0.38	NA				-0.61376			NA	NA	NA	NA	-0.61	NA
K7EQN7	ATP-dependent RNA helicase DDX39A (Fragment)	DDX39A	3.00				0.024186			NA	NA	NA	NA	0.02	NA				0.086201			NA	NA	NA	NA	0.09	NA
K7EQY5	Tyrosine-protein kinase	MATK	1.00				3.318633			NA	NA	NA	NA	3.32	NA				2.705575			NA	NA	NA	NA	2.71	NA
Q08ES8	Cell growth-inhibiting protein 34	NA	2.00				0.510723			NA	NA	NA	NA	0.51	NA				0.01056			NA	NA	NA	NA	0.01	NA
Q0VAC0	Heterogeneous nuclear ribonucleoprotein A1	HNRPA1	8.00				0.265673			NA	NA	NA	NA	0.27	NA				0.642917			NA	NA	NA	NA	0.64	NA
Q502X2	Diablo homolog (Drosophila)	DIABLO	5.00				0.275289			NA	NA	NA	NA	0.28	NA				0.846931			NA	NA	NA	NA	0.85	NA
Q53GA0	Tumor protein D52-like 2 isoform e variant (Fragment)	NA	1.00				-1.47693			NA	NA	NA	NA	-1.48	NA				-1.26672			NA	NA	NA	NA	-1.27	NA
Q53H18	Beta-galactosidase (Fragment)	NA	2.00				-0.00356			NA	NA	NA	NA	0.00	NA				0.749428			NA	NA	NA	NA	0.75	NA
Q53HG1	13kDa differentiation-associated protein variant (Fragment)	NA	2.00				-0.32126			NA	NA	NA	NA	-0.32	NA				-0.29276			NA	NA	NA	NA	-0.29	NA
Q567U8	COPS7A protein	COPS7A	1.00				1.415997			NA	NA	NA	NA	1.42	NA				1.687159			NA	NA	NA	NA	1.69	NA
Q59GF8	Heat shock 70kDa protein 4 isoform a variant (Fragment)	NA	6.00				0.263698			NA	NA	NA	NA	0.26	NA				0.407891			NA	NA	NA	NA	0.41	NA
Q59GS3	Proteasome 26S ATPase subunit 5 variant (Fragment)	NA	2.00				0.579023			NA	NA	NA	NA	0.58	NA				0.146035			NA	NA	NA	NA	0.15	NA
Q5MK14	Leukemia-associated protein (Fragment)	NA	1.00				0.878076			NA	NA	NA	NA	0.88	NA				0.176946			NA	NA	NA	NA	0.18	NA
Q6FGY1	HPCAL1 protein	HPCAL1	1.00				-0.33085			NA	NA	NA	NA	-0.33	NA				-0.6911			NA	NA	NA	NA	-0.69	NA
Q6FIH7	ATP synthase, H+ transporting, mitochondrial F0 complex, subunit C1 (Subunit 9), isoform CRA_a	ATP5G1	1.00				-1.46306			NA	NA	NA	NA	-1.46	NA				-1.9812			NA	NA	NA	NA	-1.98	NA
Q6N030	Putative uncharacterized protein DKFZp686I15212	DKFZp686I15212	3.00				0.052425			NA	NA	NA	NA	0.05	NA				-0.06662			NA	NA	NA	NA	-0.07	NA
Q6PJF2	IGK@ protein	IGK@	5.00				-0.79547			NA	NA	NA	NA	-0.80	NA				-1.02674			NA	NA	NA	NA	-1.03	NA
Q8N5Z7	60S ribosomal protein L6	RPL6	1.00				1.469336			NA	NA	NA	NA	1.47	NA				1.227395			NA	NA	NA	NA	1.23	NA
Q8TA92	Similar to AFG3 ATPase family gene 3-like 2 (Yeast) (Fragment)	NA	4.00				-0.78953			NA	NA	NA	NA	-0.79	NA				-0.43494			NA	NA	NA	NA	-0.43	NA
Q9HBM8	cDNA, FLJ78849, highly similar to 1-acyl-sn-glycerol-3-phosphate acyltransferase alpha (EC 2.3.1.51)	NA	2.00				-0.23994			NA	NA	NA	NA	-0.24	NA				-0.23104			NA	NA	NA	NA	-0.23	NA
R9WWI7	SEC62 splice variant	NA	2.00				0.319267			NA	NA	NA	NA	0.32	NA				0.450249			NA	NA	NA	NA	0.45	NA
V9HW29	Kinesin-like protein	KIF5B	2.00				-1.16059			NA	NA	NA	NA	-1.16	NA				-1.27708			NA	NA	NA	NA	-1.28	NA
V9HW68	Epididymis luminal protein 214	HEL-214	5.00				-0.8467			NA	NA	NA	NA	-0.85	NA				-1.31237			NA	NA	NA	NA	-1.31	NA
V9HW92	Epididymis secretory protein Li 112	PDLIM1	1.00				0.021332			NA	NA	NA	NA	0.02	NA				0.037119			NA	NA	NA	NA	0.04	NA

				08/017 hESC-RPE to hRPE												08/023 hESC-RPE to hRPE											
				Analysis						Technical replicate CV			Biological	Fold change (mean)	Unadjusted p-value	Analysis						Technical replicate CV			Biological	Fold change (mean)	Unadjusted p-value
UniProt	Protein name	Gene name	Peptide mean	1.1	1.2	2.1	2.2	3.1	3.2	1	2	3	CV		(t-test)	1.1	1.2	2.1	2.2	3.1	3.2	1	2	3	CV		(t-test)
X5D927	Cytochrome c oxidase subunit 1	COX1	1.00				-4.02138			NA	NA	NA	NA	-4.02	NA				-1.88579			NA	NA	NA	NA	-1.89	NA
P02790	Hemopexin	HPX	1.00					1.191133		NA	NA	NA	NA	1.19	NA					1.33948		NA	NA	NA	NA	1.34	NA
P05937	Calbindin	CALB1	1.00					0.16313		NA	NA	NA	NA	0.16	NA					-0.38091		NA	NA	NA	NA	-0.38	NA
P22307	Non-specific lipid-transfer protein	SCP2	5.00					0.595664		NA	NA	NA	NA	0.60	NA					0.422823		NA	NA	NA	NA	0.42	NA
P61457	Pterin-4-alpha-carbinolamine dehydratase	PCBD1	3.00					-0.13079		NA	NA	NA	NA	-0.13	NA					0.094827		NA	NA	NA	NA	0.09	NA
Q3KQV9	UDP-N-acetylhexosamine pyrophosphorylase-like protein 1	UAP1L1	1.00					-0.72728		NA	NA	NA	NA	-0.73	NA					-0.63493		NA	NA	NA	NA	-0.63	NA
Q86U42	Polyadenylate-binding protein 2	PABPN1	2.00					-1.39218		NA	NA	NA	NA	-1.39	NA					-1.43524		NA	NA	NA	NA	-1.44	NA
Q8N2C7	Protein unc-80 homolog	UNC80	1.00					3.025941		NA	NA	NA	NA	3.03	NA					3.782356		NA	NA	NA	NA	3.78	NA
Q8N7X1	RNA-binding motif protein, X-linked-like-3	RBMXL3	1.00					0.140988		NA	NA	NA	NA	0.14	NA					-1.30742		NA	NA	NA	NA	-1.31	NA
Q8TDZ2	Protein-methionine sulfoxide oxidase MICAL1	MICAL1	1.00					0.272486		NA	NA	NA	NA	0.27	NA					-0.32986		NA	NA	NA	NA	-0.33	NA
Q96CS3	FAS-associated factor 2	FAF2	2.00					0.035009		NA	NA	NA	NA	0.04	NA					0.312094		NA	NA	NA	NA	0.31	NA
Q9H2D6	TRIO and F-actin-binding protein	TRIOBP	1.00					0.460988		NA	NA	NA	NA	0.46	NA					0.572989		NA	NA	NA	NA	0.57	NA
Q9H444	Charged multivesicular body protein 4b	CHMP4B	1.00					1.026113		NA	NA	NA	NA	1.03	NA					0.635021		NA	NA	NA	NA	0.64	NA
A0A024R2M6	Acetyl-Coenzyme A acyltransferase 1 (Peroxisomal 3-oxoacyl-Coenzyme A thiolase), isoform CRA_a	ACAA1	2.00					-0.46691		NA	NA	NA	NA	-0.47	NA					-0.95341		NA	NA	NA	NA	-0.95	NA
A0A024R7A8	Aldo-keto reductase family 1, member B1 (Aldose reductase), isoform CRA_a	AKR1B1	3.00					0.626053		NA	NA	NA	NA	0.63	NA					0.104861		NA	NA	NA	NA	0.10	NA
A0A024R9Y3	HECT, UBA and WWE domain containing 1, isoform CRA_a	HUWE1	1.00					1.036441		NA	NA	NA	NA	1.04	NA					0.549022		NA	NA	NA	NA	0.55	NA
A0A024RAN2	Calpastatin, isoform CRA_a	CAST	2.00					-0.48109		NA	NA	NA	NA	-0.48	NA					-0.41773		NA	NA	NA	NA	-0.42	NA
A0A024RDE8	PDZ and LIM domain 5, isoform CRA_c	PDLIM5	3.00					0.44793		NA	NA	NA	NA	0.45	NA					0.18344		NA	NA	NA	NA	0.18	NA
A0A087WZE4	Spectrin alpha chain, erythrocytic 1	SPTA1	3.00					-0.04753		NA	NA	NA	NA	-0.05	NA					0.142252		NA	NA	NA	NA	0.14	NA
A8K3K1	cDNA FLJ78096, highly similar to Homo sapiens actin, alpha, cardiac muscle (ACTC), mRNA	NA	25.00					0.762914		NA	NA	NA	NA	0.76	NA					0.09289		NA	NA	NA	NA	0.09	NA
B2R6S5	UMP-CMP kinase	CMPK	2.00					-0.99923		NA	NA	NA	NA	-1.00	NA					0.996688		NA	NA	NA	NA	1.00	NA
B2R8A2	cDNA, FLJ93804, highly similar to Homo sapiens gp25L2 protein (HSGP25L2G), mRNA	NA	2.00					-0.23717		NA	NA	NA	NA	-0.24	NA					0.089396		NA	NA	NA	NA	0.09	NA
B2R9T9	cDNA, FLJ94551	NA	1.00					0.100752		NA	NA	NA	NA	0.10	NA					0.257441		NA	NA	NA	NA	0.26	NA
B3KPZ0	cDNA FLJ32485 fis, clone SKNMC2001596, highly similar to Ankyrin repeat and FYVE domain-containing pr	NA	1.00					-1.70068		NA	NA	NA	NA	-1.70	NA					0.2346		NA	NA	NA	NA	0.23	NA
B3KUZ7	cDNA FLJ40986 fis, clone UTERU2014898, highly similar to Vacuolar ATP synthase subunit H (EC 3.6.3.14)	NA	2.00					-0.00119		NA	NA	NA	NA	0.00	NA					0.115207		NA	NA	NA	NA	0.12	NA
B4DDB9	cDNA FLJ56339, highly similar to Signal peptidase complex subunit 2 (EC 3.4.-.-)	NA	1.00					-1.05797		NA	NA	NA	NA	-1.06	NA					-0.08957		NA	NA	NA	NA	-0.09	NA
B4DEX8	S-adenosylmethionine synthase	MAT2A	2.00					0.38488		NA	NA	NA	NA	0.38	NA					0.001654		NA	NA	NA	NA	0.00	NA
B4DJU4	cDNA FLJ53344, highly similar to Splicing factor 1	NA	2.00					0.341519		NA	NA	NA	NA	0.34	NA					-0.77121		NA	NA	NA	NA	-0.77	NA
B4DSQ5	cDNA FLJ53608, highly similar to Protein transport protein Sec23A	NA	1.00					-0.54512		NA	NA	NA	NA	-0.55	NA					-0.23069		NA	NA	NA	NA	-0.23	NA
B4DWL1	cDNA FLJ59240, highly similar to Far upstream element-binding protein 1	NA	4.00					0.687234		NA	NA	NA	NA	0.69	NA					0.776332		NA	NA	NA	NA	0.78	NA
B7Z768	cDNA FLJ52138, highly similar to NADH dehydrogenase (ubiquinone) 1 alpha subcomplex subunit 8 (EC 1.6	NA	1.00					-0.27219		NA	NA	NA	NA	-0.27	NA					0.288924		NA	NA	NA	NA	0.29	NA
B7Z9A0	cDNA FLJ56212, highly similar to Gelsolin	NA	12.00					0.145292		NA	NA	NA	NA	0.15	NA					0.415865		NA	NA	NA	NA	0.42	NA
C1PHA2	Kinesin-like protein	KIF5B-ALK	3.00					-0.5669		NA	NA	NA	NA	-0.57	NA					0.122633		NA	NA	NA	NA	0.12	NA
D6RGI3	Septin 11, isoform CRA_b	SEPT11	2.00					-0.35184		NA	NA	NA	NA	-0.35	NA					-0.16291		NA	NA	NA	NA	-0.16	NA
E9PDG8	Clathrin coat assembly protein AP180	SNAP91	2.00					-0.64388		NA	NA	NA	NA	-0.64	NA					-1.37876		NA	NA	NA	NA	-1.38	NA
F5H579	Probable cysteine--tRNA ligase, mitochondrial (Fragment)	CARS2	1.00					-1.43727		NA	NA	NA	NA	-1.44	NA					-0.80911		NA	NA	NA	NA	-0.81	NA
G3V1D3	Dipeptidyl peptidase 3	DPP3	2.00					0.484059		NA	NA	NA	NA	0.48	NA					0.241613		NA	NA	NA	NA	0.24	NA
H3BRU6	Poly(rC)-binding protein 2 (Fragment)	PCBP2	4.00					0.144407		NA	NA	NA	NA	0.14	NA					0.231081		NA	NA	NA	NA	0.23	NA
H3BU49	ADP-ribosylation factor-like protein 2-binding protein	ARL2BP	1.00					0.059227		NA	NA	NA	NA	0.06	NA					-0.39961		NA	NA	NA	NA	-0.40	NA
H7C2W6	Prolyl 3-hydroxylase 1 (Fragment)	P3H1	1.00					0.375967		NA	NA	NA	NA	0.38	NA					0.046992		NA	NA	NA	NA	0.05	NA
H7C3S9	COP9 signalosome complex subunit 8 (Fragment)	COPS8	1.00					1.324875		NA	NA	NA	NA	1.32	NA					0.502926		NA	NA	NA	NA	0.50	NA
I3L4A5	Inositol polyphosphate 5-phosphatase K (Fragment)	INPP5K	1.00					-0.329		NA	NA	NA	NA	-0.33	NA					-0.46547		NA	NA	NA	NA	-0.47	NA
J3QRU3	Septin-4	SEPT4	1.00					-1.14001		NA	NA	NA	NA	-1.14	NA					-0.66019		NA	NA	NA	NA	-0.66	NA
K7ESP4	Dephospho-CoA kinase domain-containing protein (Fragment)	DCAKD	1.00					0.751916		NA	NA	NA	NA	0.75	NA					0.339153		NA	NA	NA	NA	0.34	NA
Q0VGD6	HNRPR protein (Fragment)	HNRPR	3.00					-1.90906		NA	NA	NA	NA	-1.91	NA					-0.7495		NA	NA	NA	NA	-0.75	NA
Q13747	Alpha-1 antitrypsin (Fragment)	NA	1.00					-1.17999		NA	NA	NA	NA	-1.18	NA					-2.03368		NA	NA	NA	NA	-2.03	NA
Q32XH3	Ribosomal protein L18a-like protein	NA	1.00					0.708605		NA	NA	NA	NA	0.71	NA					1.19586		NA	NA	NA	NA	1.20	NA
Q59FV6	ARP3 actin-related protein 3 homolog variant (Fragment)	NA	3.00					-0.69434		NA	NA	NA	NA	-0.69	NA					0.270871		NA	NA	NA	NA	0.27	NA
Q59GX6	DEAD/H (Asp-Glu-Ala-Asp/His) box polypeptide 3 variant (Fragment)	NA	2.00					0.720701		NA	NA	NA	NA	0.72	NA					-0.67431		NA	NA	NA	NA	-0.67	NA
Q63HL5	Putative uncharacterized protein DKFZp686G12235	DKFZp686G12235	2.00					-0.27591		NA	NA	NA	NA	-0.28	NA					0.204768		NA	NA	NA	NA	0.20	NA
Q6AWB3	Putative uncharacterized protein DKFZp686E191	DKFZp686E191	1.00					-0.16033		NA	NA	NA	NA	-0.16	NA					-0.73462		NA	NA	NA	NA	-0.73	NA
Q6IBM8	U5-116KD protein	U5-116KD	2.00					0.057079		NA	NA	NA	NA	0.06	NA					-0.26035		NA	NA	NA	NA	-0.26	NA
Q6ZTQ7	cDNA FLJ44352 fis, clone TRACH3006412, highly similar to Homo sapiens COP9 constitutive photomorphog	NA	1.00					-1.65243		NA	NA	NA	NA	-1.65	NA					-0.87889		NA	NA	NA	NA	-0.88	NA
Q7Z4W5	RNA helicase	NA	1.00					0.556849		NA	NA	NA	NA	0.56	NA					0.384369		NA	NA	NA	NA	0.38	NA
Q8NHX0	Mitogen-activated protein kinase	NA	2.00					0.284472		NA	NA	NA	NA	0.28	NA					-0.16291		NA	NA	NA	NA	-0.16	NA
Q8TBR3	Fusion (Involved in t(1216) in malignant liposarcoma)	FUS	1.00					-1.00231		NA	NA	NA	NA	-1.00	NA					-0.57302		NA	NA	NA	NA	-0.57	NA
Q96Q74	Mitochondrial ribosomal protein L7/L12 (Fragment)	MRPL7/L12	1.00					0.135399		NA	NA	NA	NA	0.14	NA					-0.16616		NA	NA	NA	NA	-0.17	NA
Q9NSK3	Putative uncharacterized protein DKFZp762I166 (Fragment)	DKFZp762I166	1.00					0.828537		NA	NA	NA	NA	0.83	NA					0.115589		NA	NA	NA	NA	0.12	NA
Q9NV55	cDNA FLJ10915 fis, clone OVARC1000288, weakly similar to VACUOLAR AMINOPEPTIDASE I (EC 3.4.11.22)	NA	1.00					-0.83462		NA	NA	NA	NA	-0.83	NA					-1.16014		NA	NA	NA	NA	-1.16	NA
U3KQD5	RNA-binding protein 4B	RBM4B	1.00					0.973976		NA	NA	NA	NA	0.97	NA					1.171831		NA	NA	NA	NA	1.17	NA
V9HW04	Serine/threonine-protein phosphatase	PPP1CB	2.00					-0.24195		NA	NA	NA	NA	-0.24	NA					0.140189		NA	NA	NA	NA	0.14	NA
V9HWI4	Epididymis luminal protein 110	LTF	4.00					1.351907		NA	NA	NA	NA	1.35	NA					1.557547		NA	NA	NA	NA	1.56	NA
O14910	Protein lin-7 homolog A	LIN7A	1.00						-0.31038	NA	NA	NA	NA	-0.31	NA						-0.03084	NA	NA	NA	NA	-0.03	NA
O43776	Asparagine--tRNA ligase, cytoplasmic	NARS	2.00						0.187655	NA	NA	NA	NA	0.19	NA						-0.65718	NA	NA	NA	NA	-0.66	NA
O60493	Sorting nexin-3	SNX3	2.00						1.121185	NA	NA	NA	NA	1.12	NA						0.358437	NA	NA	NA	NA	0.36	NA
O95741	Copine-6	CPNE6	3.00						-0.66838	NA	NA	NA	NA	-0.67	NA						-1.55708	NA	NA	NA	NA	-1.56	NA
P15924	Desmoplakin	DSP	1.00						1.618122	NA	NA	NA	NA	1.62	NA						1.673435	NA	NA	NA	NA	1.67	NA
P23193	Transcription elongation factor A protein 1	TCEA1	1.00						0.761073	NA	NA	NA	NA	0.76	NA						-0.30141	NA	NA	NA	NA	-0.30	NA
P28288	ATP-binding cassette sub-family D member 3	ABCD3	1.00						3.707425	NA	NA	NA	NA	3.71	NA						4.037536	NA	NA	NA	NA	4.04	NA
P34949	Mannose-6-phosphate isomerase	MPI	1.00						-0.89193	NA	NA	NA	NA	-0.89	NA						-0.58379	NA	NA	NA	NA	-0.58	NA
P35606	Coatomer subunit beta'	COPB2	1.00						-0.57426	NA	NA	NA	NA	-0.57	NA						-0.42963	NA	NA	NA	NA	-0.43	NA
P37059	Estradiol 17-beta-dehydrogenase 2	HSD17B2	5.00						0.743791	NA	NA	NA	NA	0.74	NA						0.975493	NA	NA	NA	NA	0.98	NA
P40126	L-dopachrome tautomerase	DCT	2.00						2.21923	NA	NA	NA	NA	2.22	NA						1.890101	NA	NA	NA	NA	1.89	NA
P48735	Isocitrate dehydrogenase [NADP], mitochondrial	IDH2	8.00						1.038352	NA	NA	NA	NA	1.04	NA						0.344003	NA	NA	NA	NA	0.34	NA
P50226	Sulfotransferase 1A2	SULT1A2	2.00						-0.22317	NA	NA	NA	NA	-0.22	NA						-0.67211	NA	NA	NA	NA	-0.67	NA
P56381	ATP synthase subunit epsilon, mitochondrial	ATP5E	1.00						0.562363	NA	NA	NA	NA	0.56	NA						0.171482	NA	NA	NA	NA	0.17	NA
Q06124	Tyrosine-protein phosphatase non-receptor type 11	PTPN11	1.00						0.298103	NA	NA	NA	NA	0.30	NA						0.374711	NA	NA	NA	NA	0.37	NA
Q16531	DNA damage-binding protein 1	DDB1	1.00						-0.04231	NA	NA	NA	NA	-0.04	NA						-0.10903	NA	NA	NA	NA	-0.11	NA
Q5T2N8	ATPase family AAA domain-containing protein 3C	ATAD3C	1.00						-2.95109	NA	NA	NA	NA	-2.95	NA						-0.75712	NA	NA	NA	NA	-0.76	NA
Q6P2Q9	Pre-mRNA-processing-splicing factor 8	PRPF8	2.00						-0.62066	NA	NA	NA	NA	-0.62	NA						-1.11783	NA	NA	NA	NA	-1.12	NA
Q8WY22	BRI3-binding protein	BRI3BP	1.00						-1.29747	NA	NA	NA	NA	-1.30	NA						-1.86881	NA	NA	NA	NA	-1.87	NA
Q92665	28S ribosomal protein S31, mitochondrial	MRPS31	1.00						-0.2749	NA	NA	NA	NA	-0.27	NA						-0.60122	NA	NA	NA	NA	-0.60	NA
Q96M27	Protein PRRC1	PRRC1	1.00						-0.71611	NA	NA	NA	NA	-0.72	NA						-1.21787	NA	NA	NA	NA	-1.22	NA
Q9P0K7	Ankycorbin	RAI14	1.00						1.962126	NA	NA	NA	NA	1.96	NA						2.846176	NA	NA	NA	NA	2.85	NA
Q9ULC5	Long-chain-fatty-acid--CoA ligase 5	ACSL5	1.00						-1.89482	NA	NA	NA	NA	-1.89	NA						-0.39263	NA	NA	NA	NA	-0.39	NA
Q9UPN3	Microtubule-actin cross-linking factor 1, isoforms 1/2/3/5	MACF1	3.00						-1.45091	NA	NA	NA	NA	-1.45	NA						-1.7438	NA	NA	NA	NA	-1.74	NA
Q9Y2V2	Calcium-regulated heat stable protein 1	CARHSP1	1.00						0.155279	NA	NA	NA	NA	0.16	NA						-0.18318	NA	NA	NA	NA	-0.18	NA
A0A024R2M7	Oxidative-stress responsive 1, isoform CRA_a	OXSR1	1.00						1.187655	NA	NA	NA	NA	1.19	NA						2.334908	NA	NA	NA	NA	2.33	NA
A0A024R8U1	Solute carrier family 16 (Monocarboxylic acid transporters), member 3, isoform CRA_a	SLC16A3	1.00						1.692067	NA	NA	NA	NA	1.69	NA						0.978104	NA	NA	NA	NA	0.98	NA
A0A024R8V0	Septin 9, isoform CRA_a	SEPT9	1.00						1.129607	NA	NA	NA	NA	1.13	NA						1.340795	NA	NA	NA	NA	1.34	NA
A0A024RA94	Microfibrillar-associated protein 2, isoform CRA_d	MFAP2	1.00						0.991283	NA	NA	NA	NA	0.99	NA						0.791563	NA	NA	NA	NA	0.79	NA
A0A024RAV4	Cold shock domain protein A, isoform CRA_b	CSDA	1.00						0.252088	NA	NA	NA	NA	0.25	NA						0.843911	NA	NA	NA	NA	0.84	NA
A0A024RCY1	Vacuolar protein sorting 52 (Yeast), isoform CRA_b	VPS52	1.00						2.423318	NA	NA	NA	NA	2.42	NA						2.82355	NA	NA	NA	NA	2.82	NA
A0A0A0N0L3	TDP43 isoform H	TDP43	3.00						0.861798	NA	NA	NA	NA	0.86	NA						0.219898	NA	NA	NA	NA	0.22	NA

				08/017 hESC-RPE to hRPE											
				Analysis						Technical replicate CV			Biological	Fold change (mean)	Unadjusted p-value
UniProt	Protein name	Gene name	Peptide mean	1.1	1.2	2.1	2.2	3.1	3.2	1	2	3	CV		(t-test)
A1KY36	Cell proliferation-inducing protein 41	NA	1.00						0.934145	NA	NA	NA	NA	0.93	NA
A4D275	Actin related protein 2/3 complex, subunit 1B, 41kDa	ARPC1B	2.00						1.051299	NA	NA	NA	NA	1.05	NA
A8K8A6	cDNA FLJ76931, highly similar to Homo sapiens testes-specific heterogenous nuclear ribonucleoprotein G-T	NA	1.00						0.081065	NA	NA	NA	NA	0.08	NA
A8KAQ5	cDNA FLJ77404, highly similar to Homo sapiens small nuclear ribonucleoprotein 70kDa polypeptide (RNP a	NA	1.00						0.305555	NA	NA	NA	NA	0.31	NA
B2R6S9	cDNA, FLJ93097, highly similar to Homo sapiens low density lipoprotein receptor-related protein associate	NA	1.00						-0.13347	NA	NA	NA	NA	-0.13	NA
B2R984	cDNA, FLJ94268, highly similar to Homo sapiens histone 1, H1e (HIST1H1E), mRNA	NA	13.00						-0.48413	NA	NA	NA	NA	-0.48	NA
B2R9K8	cDNA, FLJ94440, highly similar to Homo sapiens chaperonin containing TCP1, subunit 6A (zeta 1)(CCT6A), n	NA	9.00						-0.95602	NA	NA	NA	NA	-0.96	NA
B2RB47	cDNA, FLJ95309, highly similar to Homo sapiens adenosine monophosphate deaminase 2 (isoform L)(AMPD	NA	1.00						0.220898	NA	NA	NA	NA	0.22	NA
B3KQH1	cDNA FLJ90452 fis, clone NT2RP3001475, highly similar to Splicing factor 3B subunit 3	NA	2.00						0.555936	NA	NA	NA	NA	0.56	NA
B3KTP9	cDNA FLJ38578 fis, clone HCHON2007674, highly similar to NUCLEOLIN	NA	11.00						-0.14577	NA	NA	NA	NA	-0.15	NA
B3KUI4	cDNA FLJ39967 fis, clone SPLEN2027488, highly similar to Homo sapiens family with sequence similarity 82	NA	1.00						-0.64123	NA	NA	NA	NA	-0.64	NA
B4DDN7	cDNA FLJ59839, highly similar to Homo sapiens biglycan (BGN), mRNA	NA	1.00						1.521497	NA	NA	NA	NA	1.52	NA
B4DE59	cDNA FLJ60424, highly similar to Junction plakoglobin	NA	6.00						0.956457	NA	NA	NA	NA	0.96	NA
B4DFR2	cDNA FLJ59194, moderately similar to Dynein light chain 2A, cytoplasmic	NA	1.00						-0.30113	NA	NA	NA	NA	-0.30	NA
B4DIZ1	Ermin	ERMN	1.00						0.226828	NA	NA	NA	NA	0.23	NA
B4DPP0	cDNA FLJ51032, highly similar to CD9 antigen	NA	1.00						-0.14639	NA	NA	NA	NA	-0.15	NA
B4DW11	cDNA FLJ57622, highly similar to Clusterin	NA	1.00						0.130059	NA	NA	NA	NA	0.13	NA
B4E2Z6	Eukaryotic translation initiation factor 3 subunit C	EIF3C	1.00						0.807275	NA	NA	NA	NA	0.81	NA
B4E304	cDNA FLJ53962, highly similar to AP-2 complex subunit mu-1	NA	1.00						0.233455	NA	NA	NA	NA	0.23	NA
B7Z238	cDNA FLJ55839, highly similar to Bcl-2-like 13 protein	NA	4.00						-0.06771	NA	NA	NA	NA	-0.07	NA
B7Z2V8	cDNA FLJ54958, highly similar to Rap1 GTPase-GDP dissociation stimulator 1	NA	2.00						-3.42259	NA	NA	NA	NA	-3.42	NA
B7Z9F3	cDNA, FLJ78817, highly similar to Metaxin-2	NA	1.00						1.323624	NA	NA	NA	NA	1.32	NA
D6REX3	Protein transport protein Sec31A	SEC31A	2.00						0.891127	NA	NA	NA	NA	0.89	NA
E5RGS4	Prefoldin subunit 1	PFDN1	1.00						0.416874	NA	NA	NA	NA	0.42	NA
E9PKY2	Terminal uridylyltransferase 4 (Fragment)	ZCCHC11	1.00						1.852435	NA	NA	NA	NA	1.85	NA
E9PRQ7	UBX domain-containing protein 1	UBXN1	2.00						1.054705	NA	NA	NA	NA	1.05	NA
F4ZW63	NF45	NA	2.00						0.64458	NA	NA	NA	NA	0.64	NA
F8W1D1	Melanocyte protein PMEL (Fragment)	PMEL	2.00						0.979791	NA	NA	NA	NA	0.98	NA
G3V1C3	Apoptosis inhibitor 5	API5	2.00						0.810985	NA	NA	NA	NA	0.81	NA
H0YC42	Uncharacterized protein	NA	3.00						2.135698	NA	NA	NA	NA	2.14	NA
H7BY53	EF-hand calcium-binding domain-containing protein 2 (Fragment)	EFCAB2	1.00						2.144899	NA	NA	NA	NA	2.14	NA
I1SRC5	UBE2L3/KRAS fusion protein	NA	3.00						-0.223	NA	NA	NA	NA	-0.22	NA
I3L0M9	Transcription elongation factor B polypeptide 2 (Fragment)	TCEB2	1.00						-0.47369	NA	NA	NA	NA	-0.47	NA
I3L170	Microtubule-associated protein	MAPT	1.00						0.845688	NA	NA	NA	NA	0.85	NA
J3KN16	Proteasome-associated protein ECM29 homolog	KIAA0368	1.00						-0.15817	NA	NA	NA	NA	-0.16	NA
J3QRC4	60S ribosomal protein L26 (Fragment)	RPL26	1.00						0.814767	NA	NA	NA	NA	0.81	NA
K7N7A8	Uncharacterized protein (Fragment)	NA	2.00						0.548128	NA	NA	NA	NA	0.55	NA
O15451	Proline and glutamic acid rich nuclear protein isoform (Fragment)	NA	1.00						6.021562	NA	NA	NA	NA	6.02	NA
Q4W5K9	Putative uncharacterized protein LIM (Fragment)	LIM	2.00						0.287141	NA	NA	NA	NA	0.29	NA
Q53F45	Splicing factor, arginine/serine-rich 4 variant (Fragment)	NA	2.00						0.106492	NA	NA	NA	NA	0.11	NA
Q53FN8	Aldehyde dehydrogenase 6A1 variant (Fragment)	NA	2.00						-0.11636	NA	NA	NA	NA	-0.12	NA
Q53G70	NADH dehydrogenase (Ubiquinone) flavoprotein 1, 51kDa variant (Fragment)	NA	2.00						-1.23759	NA	NA	NA	NA	-1.24	NA
Q53H88	Dynactin 2 variant (Fragment)	NA	3.00						-0.07483	NA	NA	NA	NA	-0.07	NA
Q53HI7	Dehydrogenase/reductase (SDR family) member 3 variant (Fragment)	NA	1.00						2.105671	NA	NA	NA	NA	2.11	NA
Q53HV2	Chaperonin containing TCP1, subunit 7 (Eta) variant (Fragment)	NA	8.00						-0.30723	NA	NA	NA	NA	-0.31	NA
Q58F09	Glucosidase I	GCS1	1.00						0.066427	NA	NA	NA	NA	0.07	NA
Q59EA2	Coronin (Fragment)	NA	2.00						-0.72636	NA	NA	NA	NA	-0.73	NA
Q59FI9	Ribosomal protein L12 variant (Fragment)	NA	2.00						0.65754	NA	NA	NA	NA	0.66	NA
Q59G26	Guanine nucleotide-binding protein, beta-3 subunit variant (Fragment)	NA	2.00						-0.08319	NA	NA	NA	NA	-0.08	NA
Q5HYG8	Serine hydroxymethyltransferase	DKFZp686P09201	1.00						-0.91813	NA	NA	NA	NA	-0.92	NA
Q5U0P9	Purine-rich element binding protein A	NA	1.00						0.274578	NA	NA	NA	NA	0.27	NA
Q68DQ4	Putative uncharacterized protein DKFZp779L0468	DKFZp779L0468	1.00						-0.87888	NA	NA	NA	NA	-0.88	NA
Q6FHM6	NHP2 non-histone chromosome protein 2-like 1 (S. cerevisiae)	NHP2L1	1.00						0.936953	NA	NA	NA	NA	0.94	NA
Q86SZ7	Full-length cDNA clone CS0DJ015YJ12 of T cells (Jurkat cell line) of Homo sapiens (human)	PSME2	2.00						-0.83536	NA	NA	NA	NA	-0.84	NA
Q8IY97	Adaptor-related protein complex 1, gamma 1 subunit	AP1G1	1.00						0.797307	NA	NA	NA	NA	0.80	NA
Q8N542	DYNC1I1 protein	DYNC1I1	1.00						0.533556	NA	NA	NA	NA	0.53	NA
Q8NAK9	cDNA FLJ35170 fis, clone PLACE6012942, highly similar to SPLICING FACTOR, ARGININE/SERINE-RICH 2	NA	1.00						0.484398	NA	NA	NA	NA	0.48	NA
Q96FS1	CTNND1 protein (Fragment)	CTNND1	2.00						0.940492	NA	NA	NA	NA	0.94	NA
Q9Y4T0	Putative uncharacterized protein DKFZp564P2062 (Fragment)	DKFZp564P2062	1.00						-0.01035	NA	NA	NA	NA	-0.01	NA
R4GN98	Protein S100 (Fragment)	S100A6	2.00						0.113302	NA	NA	NA	NA	0.11	NA
V9GZ56	U6 snRNA-associated Sm-like protein LSm4 (Fragment)	LSM4	1.00						-0.45963	NA	NA	NA	NA	-0.46	NA
V9HW84	Epididymis secretory sperm binding protein Li 124m	HEL-S-124m	17.00						0.052867	NA	NA	NA	NA	0.05	NA
V9HWA0	Aminoacylase-1	ACY1	1.00						-0.13673	NA	NA	NA	NA	-0.14	NA
V9HWF9	Epididymis luminal protein 20	BPNT1	2.00						-0.98593	NA	NA	NA	NA	-0.99	NA
X5DP03	Serine threonine kinase 39 isoform B (Fragment)	STK39	1.00						1.359647	NA	NA	NA	NA	1.36	NA

	08/023 hESC-RPE to hRPE											
	Analysis						Technical replicate CV			Biological	Fold change (mean)	Unadjusted p-value
UniProt	1.1	1.2	2.1	2.2	3.1	3.2	1	2	3	CV		(t-test)
A1KY36						0.549014	NA	NA	NA	NA	0.55	NA
A4D275						0.91694	NA	NA	NA	NA	0.92	NA
A8K8A6						-0.41215	NA	NA	NA	NA	-0.41	NA
A8KAQ5						0.334526	NA	NA	NA	NA	0.33	NA
B2R6S9						0.423736	NA	NA	NA	NA	0.42	NA
B2R984						-1.43744	NA	NA	NA	NA	-1.44	NA
B2R9K8						-0.51701	NA	NA	NA	NA	-0.52	NA
B2RB47						0.07234	NA	NA	NA	NA	0.07	NA
B3KQH1						-0.06581	NA	NA	NA	NA	-0.07	NA
B3KTP9						-0.2815	NA	NA	NA	NA	-0.28	NA
B3KUI4						-0.47474	NA	NA	NA	NA	-0.47	NA
B4DDN7						1.33019	NA	NA	NA	NA	1.33	NA
B4DE59						0.952002	NA	NA	NA	NA	0.95	NA
B4DFR2						-0.24147	NA	NA	NA	NA	-0.24	NA
B4DIZ1						-0.20844	NA	NA	NA	NA	-0.21	NA
B4DPP0						-1.03171	NA	NA	NA	NA	-1.03	NA
B4DW11						0.660219	NA	NA	NA	NA	0.66	NA
B4E2Z6						-0.67929	NA	NA	NA	NA	-0.68	NA
B4E304						-0.65515	NA	NA	NA	NA	-0.66	NA
B7Z238						0.208084	NA	NA	NA	NA	0.21	NA
B7Z2V8						-1.62545	NA	NA	NA	NA	-1.63	NA
B7Z9F3						0.751557	NA	NA	NA	NA	0.75	NA
D6REX3						0.627194	NA	NA	NA	NA	0.63	NA
E5RGS4						0.064991	NA	NA	NA	NA	0.06	NA
E9PKY2						3.053175	NA	NA	NA	NA	3.05	NA
E9PRQ7						1.058838	NA	NA	NA	NA	1.06	NA
F4ZW63						0.732999	NA	NA	NA	NA	0.73	NA
F8W1D1						0.579851	NA	NA	NA	NA	0.58	NA
G3V1C3						0.627609	NA	NA	NA	NA	0.63	NA
H0YC42						1.715243	NA	NA	NA	NA	1.72	NA
H7BY53						2.500503	NA	NA	NA	NA	2.50	NA
I1SRC5						-1.09342	NA	NA	NA	NA	-1.09	NA
I3L0M9						-0.80645	NA	NA	NA	NA	-0.81	NA
I3L170						1.325328	NA	NA	NA	NA	1.33	NA
J3KN16						-0.03675	NA	NA	NA	NA	-0.04	NA
J3QRC4						0.828803	NA	NA	NA	NA	0.83	NA
K7N7A8						0.538587	NA	NA	NA	NA	0.54	NA
O15451						6.149519	NA	NA	NA	NA	6.15	NA
Q4W5K9						0.868511	NA	NA	NA	NA	0.87	NA
Q53F45						-0.17905	NA	NA	NA	NA	-0.18	NA
Q53FN8						-0.04822	NA	NA	NA	NA	-0.05	NA
Q53G70						-1.63806	NA	NA	NA	NA	-1.64	NA
Q53H88						-0.98817	NA	NA	NA	NA	-0.99	NA
Q53HI7						1.631139	NA	NA	NA	NA	1.63	NA
Q53HV2						-0.31148	NA	NA	NA	NA	-0.31	NA
Q58F09						-0.44821	NA	NA	NA	NA	-0.45	NA
Q59EA2						-0.27812	NA	NA	NA	NA	-0.28	NA
Q59FI9						0.185294	NA	NA	NA	NA	0.19	NA
Q59G26						0.091556	NA	NA	NA	NA	0.09	NA
Q5HYG8						-1.37225	NA	NA	NA	NA	-1.37	NA
Q5U0P9						-0.1356	NA	NA	NA	NA	-0.14	NA
Q68DQ4						-1.32482	NA	NA	NA	NA	-1.32	NA
Q6FHM6						-0.18732	NA	NA	NA	NA	-0.19	NA
Q86SZ7						-0.05532	NA	NA	NA	NA	-0.06	NA
Q8IY97						0.50413	NA	NA	NA	NA	0.50	NA
Q8N542						0.191494	NA	NA	NA	NA	0.19	NA
Q8NAK9						0.223753	NA	NA	NA	NA	0.22	NA
Q96FS1						0.305369	NA	NA	NA	NA	0.31	NA
Q9Y4T0						0.005662	NA	NA	NA	NA	0.01	NA
R4GN98						0.675045	NA	NA	NA	NA	0.68	NA
V9GZ56						-1.30247	NA	NA	NA	NA	-1.30	NA
V9HW84						-0.02255	NA	NA	NA	NA	-0.02	NA
V9HWA0						0.182089	NA	NA	NA	NA	0.18	NA
V9HWF9						-0.11691	NA	NA	NA	NA	-0.12	NA
X5DP03						2.841912	NA	NA	NA	NA	2.84	NA

Supplementary Table S2. Differentially expressed proteins (>2-fold difference in expression) for both hESC-RPE lines compared to hRPE presented on log2 scale.
Mean number of peptides used for quantification, fold change value for each replicate analysis, mean fold change, and standard deviation shown for each protein.
Upregulated proteins marked with red color and downregulated with green color.

				08/017 hESC-RPE to hRPE							
				Analysis							
UniProt	Protein name	Gene name	Peptide mean	1.1	1.2	2.1	2.2	3.1	3.2	Fold change (mean)	StDev
V9HWI6	Epididymis secretory protein Li 51	HEL-S-51	9.50	3.476174	3.737929	3.4517	2.645289	2.235296	1.993698	2.92	0.73
Q5VY30	Retinol binding protein 4, plasma, isoform CRA_b	RBP4	2.50	2.538352			2.639081	1.836896	2.633531	2.41	0.39
C7DJS2	Glutathione S-transferase pi (Fragment)	GSTP1	19.67	1.409268	1.127546	1.993092	1.195694	3.276978	2.89305	1.98	0.91
B2R5T2	cDNA, FLJ92608, highly similar to Homo sapiens aldehyde dehydrogenase 1 fami	NA	5.33	1.379541	3.081129	1.308882	1.328324	1.867252	1.934884	1.82	0.68
A0A024R8T0	Integrin beta	ITGB4	2.20	1.472631	1.594171	1.434225	2.570083		1.935328	1.80	0.47
I4AY87	Macrophage migration inhibitory factor (Fragment)	MIF	7.17	1.511884	1.464049	1.816903	1.642547	1.995871	2.328021	1.79	0.33
S4R371	Fatty acid-binding protein, heart (Fragment)	FABP3	2.25	2.203669	1.339137	1.878319			1.511072	1.73	0.39
P16401	Histone H1.5	HIST1H1B	5.40	1.29687		1.619964	1.947181	1.535453	2.209149	1.72	0.36
Q76LA1	CSTB protein	CSTB	3.67	1.611166	1.727405	1.371686	1.357116	2.228004	2.028756	1.72	0.35
Q01650	Large neutral amino acids transporter small subunit 1	SLC7A5	1.25		0.873917		0.863226	3.253014	1.83875	1.71	1.13
A0A024R3E3	Apolipoprotein A-I, isoform CRA_a	APOA1	2.33	2.634717	2.317136	1.794767	2.021072	0.964121	0.421314	1.69	0.84
W8QEY1	Lactoferrin	NA	3.00	1.911673	1.380773				1.736472	1.68	0.27
B0YIW2	Apolipoprotein C-III	APOC3	1.17	1.848467	1.641181	1.415766	1.543017	1.125438	2.132865	1.62	0.35
D9IWP9	Beta-2-glycoprotein I (Fragment)	NA	1.20		1.300894	1.435892	1.161807	1.952719	2.087996	1.59	0.41
A0A087X0I2	Complement factor I	CFI	1.00	1.771221	1.565084	1.074888	1.405785	2.067188		1.58	0.37
Q14683	Structural maintenance of chromosomes protein 1A	SMC1A	1.00	1.590398			1.520956			1.56	0.05
Q6ZVX7	F-box only protein 50	NCCRP1	2.33	1.517464	1.042644	1.845506	1.644994	0.370895	2.912346	1.56	0.85
P35555	Fibrillin-1	FBN1	25.83	1.607747	0.988756	1.317947	1.315035	1.849868	2.22429	1.55	0.44
Q86Z22	Epididymis secretory protein Li 297	HEL-S-297	2.67	1.379692	0.580399	2.143443	1.704767	1.675466	1.721421	1.53	0.53
Q6JSD8	Aquaporin 1 splice variant 2 (Fragment)	AQP1	1.00	0.841618	2.084512	2.275493	2.145216	0.309504		1.53	0.90
B4DWN1	cDNA FLJ52285, highly similar to Vesicular integral-membrane protein VIP36	NA	2.00		0.926499	0.723277	2.92187			1.52	1.21
P05787	Keratin, type II cytoskeletal 8	KRT8	17.50	1.785737	1.388262	1.527923	1.812191	1.052638	1.506304	1.51	0.28
O14745	Na(+)/H(+) exchange regulatory cofactor NHE-RF1	SLC9A3R1	8.67	1.385279	1.86852	1.280794	1.40678	1.46048	1.42337	1.47	0.20
B2RA03	cDNA, FLJ94640, highly similar to Homo sapiens keratin 18 (KRT18), mRNA	NA	9.50				1.59447		1.263141	1.43	0.23
Q96BS4	FBL protein (Fragment)	FBL	1.00	1.378784					1.471612	1.43	0.07
Q59GX2	Solute carrier family 2 (Facilitated glucose transporter), member 1 variant (Fragr	NA	8.50	1.130076	1.169165	2.096679	1.639758	1.040796	1.470338	1.42	0.40
P17643	5,6-dihydroxyindole-2-carboxylic acid oxidase	TYRP1	5.00	1.903488	1.885831	0.852643	1.555717	0.947329	1.357277	1.42	0.45
E7EQR4	Ezrin	EZR	23.17	1.584306	1.908408	1.192397	1.184951	1.559935	1.022925	1.41	0.33
A8K5W7	cDNA FLJ75180, highly similar to Homo sapiens mitochondrial isoleucine tRNA s	NA	2.00		2.270017		0.53294			1.40	1.23
Q8N142	Adenylosuccinate synthetase isozyme 1	ADSSL1	5.00	1.800788		1.573164	2.225313	0.245973	1.138237	1.40	0.75
Q02318	Sterol 26-hydroxylase, mitochondrial	CYP27A1	1.00				0.934339	1.05775	2.142116	1.38	0.66
W6A4U0	Tetraspanin (Fragment)	CD63	1.00	1.644226			1.087584			1.37	0.39
E5RHW4	Erlin-2 (Fragment)	ERLIN2	5.33	1.765963	1.400538	1.111414	1.677252	0.98553	1.252385	1.37	0.31
E9PEB5	Far upstream element-binding protein 1	FUBP1	3.00	1.535095	0.976653	1.503732	1.750222		1.036149	1.36	0.34
Q5KU26	Collectin-12	COLEC12	2.67	1.855229	1.554266	0.775154	1.569551	1.270517	0.994904	1.34	0.40
Q53GD1	Guanine nucleotide-binding protein subunit gamma (Fragment)	NA	1.00	1.013519		1.321027	0.903948	2.089793		1.33	0.54
Q53GY0	Plastin 3 variant (Fragment)	NA	4.00		1.724823	0.933138				1.33	0.56
Q9BW75	THOP1 protein (Fragment)	THOP1	1.50	2.1276			0.50107			1.31	1.15
A0A024RDG9	3-hydroxybutyrate dehydrogenase, type 2, isoform CRA_b	BDH2	3.40	1.562545	1.370972	1.134537		1.444339	0.962634	1.30	0.24
Q05682	Caldesmon	CALD1	3.00		1.915068	1.028865	0.915036	1.723032	0.822859	1.28	0.50
P58107	Epiplakin	EPPK1	3.50			1.666462			0.882184	1.27	0.55
Q9NYU2	UDP-glucose:glycoprotein glucosyltransferase 1	UGGT1	4.00		0.796139	1.717163				1.26	0.65
P53621	Coatomer subunit alpha	COPA	2.25	1.040852	1.670399	1.419834			0.864051	1.25	0.36
A1A508	PRSS3 protein	PRSS3	3.50	1.176537		0.976485		1.365426	1.472682	1.25	0.22
F8VZY9	Keratin, type I cytoskeletal 18	KRT18	6.25	1.55097	1.488204	1.219056		0.698563		1.24	0.39
P13645	Keratin, type I cytoskeletal 10	KRT10	7.25	1.740799	1.755394	1.072748	0.375217			1.24	0.66
Q08380	Galectin-3-binding protein	LGALS3BP	5.20	1.58896	1.811201		0.254445	1.024396	1.362343	1.21	0.61
B4DHQ3	Phosphoserine aminotransferase	NA	2.00	1.026912	1.875056		1.669234	0.53945	0.91073	1.20	0.55
O95336	6-phosphogluconolactonase	PGLS	2.83	1.09558	0.586227	0.790865	1.188078	2.148575	1.356725	1.19	0.54
Q5VSQ6	Procollagen-proline, 2-oxoglutarate 4-dioxygenase (Proline 4-hydroxylase), alph	P4HA1	4.83	1.679007	1.433592	1.521844	1.310897	0.563959	0.64105	1.19	0.47
V9HWH7	Epididymis secretory sperm binding protein Li 70p	ATIC	4.40		2.708129	1.393037	1.133248	0.29977	0.39178	1.19	0.97
A0A024R5K8	Serpin peptidase inhibitor, clade H (Heat shock protein 47), member 1, (Collage	SERPINH1	9.17	1.494593	1.630982	0.83388	0.915036	0.853431	1.353521	1.18	0.35
A8K7F6	cDNA FLJ78244, highly similar to Homo sapiens eukaryotic translation initiation	NA	5.00		0.855675			1.497786		1.18	0.45
Q9Y490	Talin-1	TLN1	12.67	0.72129	1.513624	0.949289	1.809034	0.997622	1.045696	1.17	0.41
Q0VDC6	Peptidyl-prolyl cis-trans isomerase	FKBP1A	2.00			1.431771			0.905911	1.17	0.37
Q9UNZ2	NSFL1 cofactor p47	NSFL1C	9.50	0.714109	1.293634			1.21656	1.434498	1.16	0.31
O60664	Perilipin-3	PLIN3	2.00	0.995465	1.336283			1.498874	0.824057	1.16	0.31
K7EPS4	Matrix-remodeling-associated protein 7	MXRA7	1.00	0.958362		1.357688				1.16	0.28
K7EKL3	Granulins (Fragment)	GRN	2.00	1.656329	1.705047	1.42211	1.091548	0.61423	0.407422	1.15	0.54
Q5NKV8	Intercellular adhesion molecule 1	ICAM1	2.83	1.480483	1.705221	1.145721	0.670808	0.612402	1.237177	1.14	0.43
A0A0A0MSQ0	Plastin-3	PLS3	5.67				0.818483	1.363036	1.229663	1.14	0.28
A0A0A0MQT0	Retinol binding protein 1, cellular	RBP1	10.33	1.647183	1.582683	1.040381	1.242685	0.442077	0.816292	1.13	0.46
Q96NY7	Chloride intracellular channel protein 6	CLIC6	5.67	1.537945	1.458189	0.726324	0.776623	1.792673	0.45583	1.12	0.54
P54868	Hydroxymethylglutaryl-CoA synthase, mitochondrial	HMGCS2	9.33	1.134297	0.95498	1.156106	1.2448	1.098142	1.145154	1.12	0.10
I6L893	Uncharacterized protein	NA	2.33			1.613568		1.295904	0.438936	1.12	0.61
A0A024R6C0	Niemann-Pick disease, type C2, isoform CRA_a	NPC2	5.50	1.415775	0.815899	1.439317			0.757148	1.11	0.37
Q8NAB7	cDNA FLJ35635 fis, clone SPLEN2011805, highly similar to BONE/CARTILAGE PRC	NA	1.00			1.466906	1.495283	0.3524		1.10	0.65
Q6MZV5	Putative uncharacterized protein DKFZp686M09245	DKFZp686M09245	1.50			1.419635			0.753883	1.09	0.47
B4DDL4	cDNA FLJ53966, moderately similar to Homo sapiens cytidylate kinase (CMPK), r	NA	1.67			0.263344	1.799296		1.14029	1.07	0.77
A0A087WYV8	Fibrillin-2	FBN2	2.33			1.394953		0.369381	1.421472	1.06	0.60
P21333	Filamin-A	FLNA	38.83	1.279703	1.27822	1.027436	0.960179	0.79485	1.030971	1.06	0.19
K7EP04	Heat shock protein beta-6	HSPB6	1.00			1.585581		0.536273		1.06	0.74
B3KN29	cDNA FLJ13371 fis, clone PLACE1000656, highly similar to PRA1 family protein 2	NA	1.60	1.634812	1.355995	0.932861	0.564835	0.719683		1.04	0.45
Q53HV6	Niemann-Pick disease, type C2 variant (Fragment)	NA	3.50				1.134689	0.92909		1.03	0.15
P49006	MARCKS-related protein	MARCKSL1	1.00	1.493755	1.169756		2.014497	0.854514	-0.39056	1.03	0.90
A0A0C4DFR6	Protein SEC13 homolog	SEC13	2.00	1.781277	1.817946		0.703068	0.56254	0.196686	1.01	0.74
Q5U071	High-mobility group box 2	NA	4.25	-0.95256	-1.12816			-0.49264	-1.43859	-1.00	0.40
O43491	Band 4.1-like protein 2	EPB41L2	11.17	-0.78887	-0.84841	-0.97712	-0.9087	-1.20008	-1.30304	-1.00	0.20
Q53H26	Transferrin variant (Fragment)	NA	9.17	-0.28457	-0.91665	-1.13232	-0.81111	-1.88408	-1.01249	-1.01	0.52
O75947	ATP synthase subunit d, mitochondrial	ATP5H	10.83	-0.64311	-1.0145	-1.4123	-1.40605	-0.55535	-1.0898	-1.02	0.36
Q6DEN2	DPYSL3 protein	DPYSL3	21.00	-0.99195	-0.54555	-1.0466	-1.27141	-1.03259	-1.23393	-1.02	0.26
Q8WYJ5	Protein kinase C inhibitor-2	NA	2.60	-0.99078	-1.62207	-0.60646	-1.1017		-0.82181	-1.03	0.38
B7Z4Z4	cDNA FLJ51918, highly similar to Peroxisomal membrane protein PEX14	NA	3.67		-0.1267	-1.67125	-1.28805			-1.03	0.80
P20941	Phosducin	PDC	5.00	-1.27287	-0.69365	-1.04769	-0.95804	-1.18253		-1.03	0.22
P16403	Histone H1.2	HIST1H1C	14.67		-1.89178	-0.49692	-0.72493			-1.04	0.75
P51970	NADH dehydrogenase [ubiquinone] 1 alpha subcomplex subunit 8	NDUFA8	4.50	-0.89835	-0.47235	-1.42008	-1.38043			-1.04	0.45
Q0VAS5	Histone H4	HIST1H4H	10.00	-2.30607	-2.11864	-0.6683	-0.49502	-0.23766	-0.49066	-1.05	0.91
P12271	Retinaldehyde-binding protein 1	RLBP1	19.50	-0.85143	-0.83922	-1.04934	-0.91068	-1.32655	-1.45245	-1.07	0.26
P78363	Retinal-specific ATP-binding cassette transporter	ABCA4	23.00	-0.59029	-0.29793	-1.20439	-1.87592	-0.25724	-2.21216	-1.07	0.83
Q8TAS0	ATP synthase subunit gamma (Fragment)	NA	8.40	-0.52595		-1.60929	-1.33384	-0.7796	-1.11927	-1.07	0.43
Q59FD4	Hexokinase (Fragment)	NA	24.17	-1.01603	-0.89038	-1.46914	-1.32853	-0.76503	-0.99239	-1.08	0.27
Q6NVC0	SLC25A5 protein (Fragment)	SLC25A5	14.67	-0.87542	-0.85974	-1.51385	-1.78408	-0.74779	-0.75756	-1.09	0.44
P13073	Cytochrome c oxidase subunit 4 isoform 1, mitochondrial	COX4I1	3.83	-0.70497	-1.24883	-1.28477	-1.50434	-1.08174	-0.73786	-1.09	0.32
P17568	NADH dehydrogenase [ubiquinone] 1 beta subcomplex subunit 7	NDUFB7	1.00	-0.64557	-1.27938	-1.7011	-0.34384	-1.51864		-1.10	0.58
A0A024QYX3	RNA binding motif (RNP1, RRM) protein 3, isoform CRA_c	RBM3	1.00	0.146797				-1.5667	-1.89115	-1.10	1.10
C9JF17	Apolipoprotein D (Fragment)	APOD	1.75	-1.51578	0.11068	-1.46255	-1.54762			-1.10	0.81
B4E192	cDNA FLJ54224, highly similar to Glial fibrillary acidic protein, astrocyte	NA	15.00	-0.7299	-1.0776	-1.51992				-1.11	0.40
P12235	ADP/ATP translocase 1	SLC25A4	14.50	-0.24764	-0.59029	-2.06035	-1.51109	-1.47486	-0.79508	-1.11	0.68

				08/023 hESC-RPE to hRPE							
				Analysis							
UniProt	Protein name	Gene name	Peptide mean	1.1	1.2	2.1	2.2	3.1	3.2	Fold change (mean)	StDev
V9HWI6	Epididymis secretory protein Li 51	HEL-S-51	9.50	3.209785	3.454716	2.903293	2.341777	2.180797	2.325072	2.74	0.53
Q5VY30	Retinol binding protein 4, plasma, isoform CRA_b	RBP4	2.50	2.48967			1.9235	2.212641	3.268719	2.47	0.58
P35555	Fibrillin-1	FBN1	25.83	2.635805	2.18955	2.497953	2.681427	1.935242	2.457065	2.40	0.29
Q05682	Caldesmon	CALD1	3.00		2.14645	2.897089	2.251019	2.436027	1.435657	2.23	0.53
A0A024R3E3	Apolipoprotein A-I, isoform CRA_a	APOA1	2.33	2.171774	2.231446	1.251878	1.297959	2.338172	1.711849	1.83	0.48
Q76LA1	CSTB protein	CSTB	3.67	1.634077	1.810028	1.595385	1.952152	1.727776	1.733579	1.74	0.13
Q01650	Large neutral amino acids transporter small subunit 1	SLC7A5	1.25		1.259375		1.010685	3.038763	1.584927	1.72	0.91
B2R5T2	cDNA, FLJ92608, highly similar to Homo sapiens aldehyde dehydrogenase 1 family, member A3 (A	NA	5.33	1.301627	2.395054	1.348755	1.562839	1.58923	2.115222	1.72	0.44
W8QEY1	Lactoferrin	NA	3.00	1.926334	1.017848				2.163906	1.70	0.60
B0YIW2	Apolipoprotein C-III	APOC3	1.17	1.759619	1.640158	1.341283	1.338937	1.672284	2.405546	1.69	0.39
Q8NAB7	cDNA FLJ35635 fis, clone SPLEN2011805, highly similar to BONE/CARTILAGE PROTEOGLYCAN I	NA	1.00			2.094576	1.975248	0.990457		1.69	0.61
A0A087X0I2	Complement factor I	CFI	1.00	1.834888	1.188535	1.596808	1.442842	2.369161		1.69	0.45
Q5KU26	Collectin-12	COLEC12	2.67	2.15145	1.633213	1.202811	2.124668	1.788893	1.007687	1.65	0.47
O14745	Na(+)/H(+) exchange regulatory cofactor NHE-RF1	SLC9A3R1	8.67	1.407596	1.91543	1.841261	1.905157	1.435722	1.267986	1.63	0.29
Q6ZVX7	F-box only protein 50	NCCRP1	2.33	2.369177	1.450086	1.052187	1.605343	0.454857	2.690151	1.60	0.83
Q9BS10	Similar to ribosomal protein S8 (Fragment)	NA	1.50		2.733932				0.465579	1.60	1.60
P05787	Keratin, type II cytoskeletal 8	KRT8	17.50	1.86948	1.38205	1.650753	1.886236	0.998759	1.810548	1.60	0.35
B2RA03	cDNA, FLJ94640, highly similar to Homo sapiens keratin 18 (KRT18), mRNA	NA	9.50				2.047228		1.128552	1.59	0.65
A0A024R8T0	Integrin beta	ITGB4	2.20	1.325605	1.081709	0.957238	2.058359		2.515707	1.59	0.67
Q5NKV8	Intercellular adhesion molecule 1	ICAM1	2.83	1.687572	2.235523	1.899533	1.475243	0.779106	1.448814	1.59	0.49
E7EQR4	Ezrin	EZR	23.17	1.508392	1.967914	1.382341	1.497579	1.806084	1.332742	1.58	0.25
P58107	Epiplakin	EPPK1	3.50			2.196365			0.914897	1.56	0.91
I4AY87	Macrophage migration inhibitory factor (Fragment)	MIF	7.17	1.49306	1.608784	1.451427	1.373144	1.39628	1.740643	1.51	0.14
K7EKL3	Granulins (Fragment)	GRN	2.00	1.734948	1.469632	1.42867	1.68763	1.379927	1.314178	1.50	0.17
P30711	Glutathione S-transferase theta-1	GSTT1	1.00				0.979925	1.955803		1.47	0.69
B4DDL4	cDNA FLJ53966, moderately similar to Homo sapiens cytidylate kinase (CMPK), mRNA	NA	1.67			1.154178	1.73449		1.484754	1.46	0.29
Q9BW75	THOP1 protein (Fragment)	THOP1	1.50	2.054135			0.831557			1.44	0.86
A0A024R5K8	Serpin peptidase inhibitor, clade H (Heat shock protein 47), member 1, (Collagen binding protein	SERPINH1	9.17	1.638447	1.764575	1.156564	1.378589	1.164101	1.465036	1.43	0.25
E5RHW4	Erlin-2 (Fragment)	ERLIN2	5.33	1.503531	1.291599	0.742169	1.179511	1.770041	2.079551	1.43	0.47
P08727	Keratin, type I cytoskeletal 19	KRT19	7.67			1.754672	1.446921	1.08121		1.43	0.34
O60664	Perilipin-3	PLIN3	2.00	1.499119	1.443458			1.606037	1.159648	1.43	0.19
A0A087WYV8	Fibrillin-2	FBN2	2.33			2.219534		0.574239	1.367344	1.39	0.82
B7Z6Z4	Myosin light polypeptide 6	MYL6	6.17	1.361267	1.234305	1.24858	1.479864	1.274722	1.642361	1.37	0.16
Q5TCU3	Tropomyosin beta chain	TPM2	6.75	1.510547		1.567651	1.230194		1.178877	1.37	0.20
B4E106	cDNA FLJ53399, highly similar to Monocarboxylate transporter 1	NA	2.67	0.982141	1.154491	1.976721				1.37	0.53
A0A024RDG9	3-hydroxybutyrate dehydrogenase, type 2, isoform CRA_b	BDH2	3.40	1.5323	1.362901	1.290254		1.524003	1.13344	1.37	0.17
P49006	MARCKS-related protein	MARCKSL1	1.00	1.050596	1.169852		1.839123	1.453649	1.322763	1.37	0.30
Q53GD1	Guanine nucleotide-binding protein subunit gamma (Fragment)	NA	1.00	1.245434		1.838433	0.859642	1.478498		1.36	0.41
Q96NY7	Chloride intracellular channel protein 6	CLIC6	5.67	0.96944	0.98003	1.755946	1.744541	1.898277	0.686664	1.34	0.52
Q6MZV5	Putative uncharacterized protein DKFZp686M09245	DKFZp686M09245	1.50			1.423302			1.249645	1.34	0.12
Q0VDC6	Peptidyl-prolyl cis-trans isomerase	FKBP1A	2.00			1.802176			0.866633	1.33	0.66
B4DWN1	cDNA FLJ52285, highly similar to Vesicular integral-membrane protein VIP36	NA	2.00		0.259836	1.023967	2.715521			1.33	1.26
Q6FI52	Transgelin	TAGLN	2.83	-0.26084	0.727592	2.250123	3.068471	1.476716	0.703459	1.33	1.20
P04271	Protein S100-B	S100B	2.40	2.362759		1.477197	1.572883	0.486047	0.675179	1.31	0.76
P21333	Filamin-A	FLNA	38.83	1.270175	1.293218	1.450871	1.393843	1.132476	1.327719	1.31	0.11
D9IWP9	Beta-2-glycoprotein I (Fragment)	NA	1.20		1.318884	0.78803	0.89223	1.759758	1.731517	1.30	0.45
H3BT58	Coactosin-like protein	COTL1	2.83	0.949382	1.142064	1.742547	1.793554	1.307088	0.843076	1.30	0.40
E5RG77	Proline synthase co-transcribed bacterial homolog protein (Fragment)	PROSC	1.50			1.25644		1.287823		1.27	0.02
K7EPS4	Matrix-remodeling-associated protein 7	MXRA7	1.00	1.586568		0.938874				1.26	0.46
Q53GY0	Plastin 3 variant (Fragment)	NA	4.00		1.758466	0.760659				1.26	0.71
F8VZY9	Keratin, type I cytoskeletal 18	KRT18	6.25	1.284368	1.255689	1.182774		1.285281		1.25	0.05
Q6JSD8	Aquaporin 1 splice variant 2 (Fragment)	AQP1	1.00	0.604838	1.870517	1.804424	1.616907	0.343071		1.25	0.72
V9HW56	Epididymis secretory protein Li 108	TPM4	8.50	0.465157	1.272935	1.078668	2.211355	1.583032	0.875413	1.25	0.60
Q59GX2	Solute carrier family 2 (Facilitated glucose transporter), member 1 variant (Fragment)	NA	8.50	1.024981	1.125696	1.739747	1.123858	0.852516	1.536669	1.23	0.33
Q86Z22	Epididymis secretory protein Li 297	HEL-S-297	2.67	1.887952	0.547836	1.455585	0.739185	1.504093	1.267363	1.23	0.50
A0A024R694	Actinin, alpha 1, isoform CRA_a	ACTN1	13.80	1.675618	0.688611	1.133795	1.308185	1.352738		1.23	0.36
A0A087X1S2	Nuclease-sensitive element-binding protein 1	YBX1	1.50	1.774235			0.661384			1.22	0.79
A0A024RAV0	Branched-chain-amino-acid aminotransferase	BCAT1	2.40	0.677332	2.515058	1.17394	0.982226	0.725715		1.21	0.75
Q96BS4	FBL protein (Fragment)	FBL	1.00	1.321051					1.088036	1.20	0.16
Q5VSQ6	Procollagen-proline, 2-oxoglutarate 4-dioxygenase (Proline 4-hydroxylase), alpha polypeptide I va	P4HA1	4.83	1.616033	1.517908	1.253259	1.073906	0.753578	0.942787	1.19	0.33
O95336	6-phosphogluconolactonase	PGLS	2.83	1.056718	0.83427	0.541896	1.204035	2.60943	0.910006	1.19	0.73
Q8N142	Adenylosuccinate synthetase isozyme 1	ADSSL1	5.00	1.369856		1.122449	1.982673	0.702395	0.740579	1.18	0.53
C7DJS2	Glutathione S-transferase pi (Fragment)	GSTP1	19.67	1.257033	1.192397	0.745947	1.274471	1.705885	0.889003	1.18	0.34
E9PEB5	Far upstream element-binding protein 1	FUBP1	3.00	1.180888	0.928514	1.24057	1.493551		1.009602	1.17	0.22
A0A024R1N1	Myosin, heavy polypeptide 9, non-muscle, isoform CRA_a	MYH9	23.00	0.716398	1.418696	1.524849	1.114448	1.249635	0.980167	1.17	0.30
B4DHK9	cDNA FLJ51656, highly similar to Actin-like protein 2	NA	1.00		0.825343		1.446643			1.14	0.44
E9PKZ0	60S ribosomal protein L8 (Fragment)	RPL8	1.00			1.919539	0.754972		0.7198	1.13	0.68
A1A508	PRSS3 protein	PRSS3	3.50	1.107635		0.695584		1.311344	1.359105	1.12	0.30
B2R923	cDNA, FLJ94174	NA	3.50		1.30691				0.895348	1.10	0.29
F8W031	Uncharacterized protein (Fragment)	NA	4.67	0.861905	1.08732	1.574465	0.996463	0.76104	1.275965	1.09	0.30
Q9UNZ2	NSFL1 cofactor p47	NSFL1C	9.50	0.953235	1.465967			0.800515	1.123844	1.09	0.29
Q14683	Structural maintenance of chromosomes protein 1A	SMC1A	1.00	1.219604			0.950977			1.09	0.19
Q59GX9	Ribosomal protein L5 variant (Fragment)	NA	1.50		0.63044		1.53933			1.08	0.64
Q53HV6	Niemann-Pick disease, type C2 variant (Fragment)	NA	3.50				1.236955	0.900383		1.07	0.24
A0A024R6C0	Niemann-Pick disease, type C2, isoform CRA_a	NPC2	5.50	1.186483	0.845718	1.450779			0.788096	1.07	0.31
Q69YG1	Myotrophin	DKFZp761E1322	2.67	1.147053	1.116579	1.24858	0.91208	0.734689	1.191025	1.06	0.20
P07737	Profilin-1	PFN1	10.00	0.731521	0.68738	1.107846	1.253985	1.554453	0.934521	1.04	0.33
P13645	Keratin, type I cytoskeletal 10	KRT10	7.25	1.111899	1.732706	0.996453	0.323492			1.04	0.58
O14618	Copper chaperone for superoxide dismutase	CCS	1.00		1.415809	1.09854	0.920504		0.71967	1.04	0.30
Q9Y490	Talin-1	TLN1	12.67	0.899862	0.95615	0.565002	1.529835	1.094343	1.168056	1.04	0.32
E9PBS1	Multifunctional protein ADE2 (Fragment)	PAICS	4.25	0.812978		0.160984	2.05854		1.104459	1.03	0.79
Q53GD0	Hydroxysteroid (17-beta) dehydrogenase 2 variant (Fragment)	NA	2.33	0.165829	2.274029	0.621855				1.02	1.11
V9HWH7	Epididymis secretory sperm binding protein Li 70p	ATIC	4.40		2.07853	1.146196	0.932189	0.276515	0.656556	1.02	0.68
H0Y360	AMP deaminase 2 (Fragment)	AMPD2	1.00	0.663426		1.350046				1.01	0.49
Q08380	Galectin-3-binding protein	LGALS3BP	5.20	1.008303	0.7056		0.142839	0.583239	2.584213	1.00	0.94
J3K000	PEPD protein	PEPD	3.83	0.686756	1.927066	0.946379	0.206552	1.136808	1.108337	1.00	0.57
A8K5W7	cDNA FLJ75180, highly similar to Homo sapiens mitochondrial isoleucine tRNA synthetase, mRNA	NA	2.00		1.849392		0.151726			1.00	1.20
O15540	Fatty acid-binding protein, brain	FABP7	2.00	-0.87739	-1.37398		-0.63751	-1.11869		-1.00	0.32
Q6NVY1	3-hydroxyisobutyryl-CoA hydrolase, mitochondrial	HIBCH	2.33	-1.09136		-1.40956	-0.52068			-1.01	0.45
E7EWZ0	NADH dehydrogenase [ubiquinone] 1 beta subcomplex subunit 9	NDUFB9	2.50	-0.65305	-0.61221	-1.51122	-1.29803			-1.02	0.45
G3XHN4	Cell adhesion molecule 2 isoform 4	CADM2	2.25	-0.77111	-1.58651	-1.24338		-0.47837		-1.02	0.49
A0A024R7I7	H.sapiens ras-related Hrab3A protein	RAB3A	5.00	-1.21155	-0.24251	-0.85953	-1.77541			-1.02	0.64
Q8NF37	Lysophosphatidylcholine acyltransferase 1	LPCAT1	1.33	-0.10284	-0.98487		-1.99019			-1.03	0.94
Q6FI13	Histone H2A type 2-A	HIST2H2AA3	8.50	-1.39877		-0.65562				-1.03	0.53
A0A087WZH7	Myristoylated alanine-rich C-kinase substrate	MARCKS	2.50	-1.10402	-1.55703	-0.9138	-0.54036	-1.61737	-0.4909	-1.04	0.48
A0A024QYX3	RNA binding motif (RNP1, RRM) protein 3, isoform CRA_c	RBM3	1.00	-0.49647				-0.73979	-1.88216	-1.04	0.74
Q15257	Serine/threonine-protein phosphatase 2A activator	PPP2R4	2.00		-1.00243	-0.94611	-1.17669			-1.04	0.12
Q0VAS5	Histone H4	HIST1H4H	10.00	-1.78464	-1.60717	-0.53009	-0.51418	-0.90575	-0.96166	-1.05	0.54

				08/017 hESC-RPE to hRPE							
				Analysis							
UniProt	Protein name	Gene name	Peptide mean	1.1	1.2	2.1	2.2	3.1	3.2	Fold change (mean)	StDev
Q5T697	Guanine nucleotide binding protein (G protein), alpha transducing activity polyp	GNAT2	12.00		-1.53809	-0.68951				-1.11	0.60
Q15257	Serine/threonine-protein phosphatase 2A activator	PPP2R4	2.00		-1.6915	-0.8196	-0.87543			-1.13	0.49
H0Y886	NADH dehydrogenase [ubiquinone] 1 beta subcomplex subunit 5, mitochondria	NDUFB5	1.00		0.053477	-1.70975	-1.73762			-1.13	1.03
A0A024RB75	Citrate synthase	CS	14.00	-0.54852	-0.86872	-1.73599	-1.76872	-0.66944	-1.21839	-1.13	0.53
P68366	Tubulin alpha-4A chain	TUBA4A	38.00	-1.32715	-1	-0.75421	-0.2128	-1.78633	-1.73904	-1.14	0.61
H0Y695	Aminomethyltransferase (Fragment)	AMT	1.33	-0.91348		-0.90686	-1.5994			-1.14	0.40
A0A024R4F1	Enolase 1, (Alpha), isoform CRA_a	ENO1	61.00	-1.37281	-1.39643	-0.75869	-0.72536	-1.37033	-1.23426	-1.14	0.32
Q08722	Leukocyte surface antigen CD47	CD47	2.33	-1.16168	-1.18702	-1.03081	-1.11534	-1.15083	-1.21412	-1.14	0.06
B7Z8A2	cDNA FLJ51671, highly similar to Prenylcysteine oxidase (EC 1.8.3.5)	NA	1.50		-1.27661	-1.01572				-1.15	0.18
Q53FI7	Four and a half LIM domains 1 variant (Fragment)	NA	2.00				-1.5407	-0.59555	-1.33121	-1.16	0.50
Q71U36	Tubulin alpha-1A chain	TUBA1A	44.00			-1.11729		-0.95114	-1.42563	-1.16	0.24
Q53YE7	Amine oxidase [flavin-containing]	MAOA	3.20	-1.40165	-1.11947	-1.15516	-1.17243		-1.00283	-1.17	0.15
Q5JWF2	Guanine nucleotide-binding protein G(s) subunit alpha isoforms XLas	GNAS	4.00	-2.04093	-1.01719	-1.34342		-0.69479	-0.77245	-1.17	0.55
A0A024RB18	Retinol dehydrogenase 5 (11-cis and 9-cis), isoform CRA_a	RDH5	10.67	-1.29058	-1.17797	-1.12017	-1.16887	-1.12927	-1.23327	-1.19	0.06
Q8NCF7	cDNA FLJ90278 fis, clone NT2RP1000325, highly similar to Phosphate carrier pro	NA	7.40	-1.15246	-1.27156	-2.08151		-0.90513	-0.68328	-1.22	0.53
P16499	Rod cGMP-specific 3',5'-cyclic phosphodiesterase subunit alpha	PDE6A	16.67	-1.19825	-1.05101	-1.76093	-1.48946	-0.80233	-1.02078	-1.22	0.35
O75475	PC4 and SFRS1-interacting protein	PSIP1	2.80	-1.58496	-1.40696	-1.37401	-1.01817		-0.73951	-1.22	0.34
P51991	Heterogeneous nuclear ribonucleoprotein A3	HNRNPA3	5.25	-0.96942	-1.24703			-0.78008	-1.90749	-1.23	0.49
Q53YE2	Syntaxin 3A	STX3A	3.25	-0.73513		-2.72019		-0.82617	-0.68011	-1.24	0.99
P35613	Basigin	BSG	8.67	-2.19735		-0.83417	-0.69432			-1.24	0.83
P07305	Histone H1.0	H1F0	2.17	-2.02482	-2.14108	-0.44693	-0.69816	-1.0726	-1.12142	-1.25	0.69
V9HW31	ATP synthase subunit beta	ATP5B	65.17	-0.92464	-0.93214	-1.75197	-1.9082	-1.07407	-0.95739	-1.26	0.45
A0A0C4DGE4	Syntaxin-3 (Fragment)	STX3	5.00		-0.89038		-1.626			-1.26	0.52
O14561	Acyl carrier protein, mitochondrial	NDUFAB1	3.00	-0.76292	-1.04238	-1.43505	-1.79593			-1.26	0.45
A0A087WZH7	Myristoylated alanine-rich C-kinase substrate	MARCKS	2.50	-0.91719	-1.39393	-0.81213	-1.19277	-2.37903	-0.90167	-1.27	0.59
V9HW12	Epididymis secretory sperm binding protein Li 2a	PRDX2	18.50	-1.33359	-1.41605	-1.45672	-1.15119	-1.26143	-1.10006	-1.29	0.14
B4DHC6	Amino acid transporter	NA	5.33		-1.84876	-1.42292	-0.59183			-1.29	0.64
Q9UNL6	Hemoglobin gamma-G (Fragment)	HBG2	11.50			-0.97608			-1.6296	-1.30	0.46
P21912	Succinate dehydrogenase [ubiquinone] iron-sulfur subunit, mitochondrial	SDHB	1.60	-0.8077	-1.24073	-1.43076	-2.39007		-0.64853	-1.30	0.68
Q6LET3	HPRT1 protein (Fragment)	HPRT1	3.00	-1.16786	-1.36187	-1.10812	-0.86627	-1.90696	-1.42905	-1.31	0.36
Q53ES0	Sodium/potassium-transporting ATPase subunit alpha (Fragment)	NA	40.67	-1.03608	-1.23001	-1.34544	-1.44931	-1.31537	-1.49923	-1.31	0.17
J3KS22	L-xylulose reductase (Fragment)	DCXR	3.50	-1.83729	-1.64089	-1.46474	-0.36643			-1.33	0.66
G4V2I9	Anion exchange protein	NA	6.50	-1.22423	-1.19873	-1.13348	-1.99276	-1.21623	-1.21445	-1.33	0.33
Q8IZ29	Tubulin, beta 2C	TUBB2C	54.83	-1.6651	-1.64149	-0.73776	-0.85478	-1.41213	-1.67265	-1.33	0.43
A0A024RA61	Heterogeneous nuclear ribonucleoprotein A2/B1, isoform CRA_c	HNRPA2B1	13.25	-1.60458	-1.82906			-0.84714	-1.04741	-1.33	0.46
A0A024QZT0	Voltage-dependent anion channel 2, isoform CRA_c	VDAC2	16.17	-0.97212	-1.2679	-2.18618	-2.04403	-0.75414	-0.76907	-1.33	0.64
Q17R60	Interphotoreceptor matrix proteoglycan 1	IMPG1	4.80	-0.69379	-0.74267	-2.21543	-1.85193	-1.19271		-1.34	0.68
Q59GB4	Dihydropyrimidinase-like 2 variant (Fragment)	NA	19.83	-1.59266	-1.43594	-0.7722	-1.66226	-1.30083	-1.27781	-1.34	0.32
D3GKD8	A-gamma globin Osilo variant	HBG1	16.25	-0.57027	-1.52929		-1.18616	-2.14649		-1.36	0.66
Q3MHU6	NDUFB1 protein (Fragment)	NDUFB1	1.00		-0.94927	-1.80286				-1.38	0.60
Q5XKP0	MIC	MIC13	1.00	-1.29347	-1.20179	-1.3307	-2.05057	-1.06908		-1.39	0.38
Q68CM6	STXBP1 protein	stxbp1	14.83	-1.0737	-1.78653	-1.25031	-1.21219	-1.62587	-1.44474	-1.40	0.27
Q9H3N1	Thioredoxin-related transmembrane protein 1	TMX1	1.50		-0.99129	-1.80934				-1.40	0.58
Q14894	Ketimine reductase mu-crystallin	CRYM	11.67	-0.86463	-1.37756	-1.5491	-1.54607	-1.75674	-1.36213	-1.41	0.30
B3KV96	cDNA FLJ16285 fis, clone OCBBF2004038, highly similar to Dihydropyrimidinase-	NA	9.33		-1.24613		-1.33118	-1.71805		-1.43	0.25
Q53XX5	Cold inducible RNA binding protein	CIRBP	3.00	-1.31192	-1.28307			-1.32655	-1.8781	-1.45	0.29
Q59EG8	Proteasome 26S non-ATPase subunit 2 variant (Fragment)	NA	3.50		-0.22845			-2.67948		-1.45	1.73
P47804	RPE-retinal G protein-coupled receptor	RGR	6.83	-1.64711	-1.27523	-1.78265	-2.11992	-0.76124	-1.16513	-1.46	0.49
B1AKZ4	Phosphoprotein enriched in astrocytes 15, isoform CRA_a	PEA15	5.17	0.49599	-1.85905	-1.6478	-1.72275	-2.40767	-1.61308	-1.46	1.00
Q0Z7D8	Cytochrome c oxidase subunit 2	COX2	3.17	-0.85998	-1.00952	-2.0748	-2.397	-1.37431	-1.07483	-1.47	0.63
Q71UI9	Histone H2A.V	H2AFV	5.17	-1.90719	-2.24613	-1.28671	-1.74821	-0.97512	-0.66367	-1.47	0.60
P19404	NADH dehydrogenase [ubiquinone] flavoprotein 2, mitochondrial	NDUFV2	3.00	-1.15553	-0.61043	-1.51007	-0.98962	-1.58294	-3.0378	-1.48	0.84
P08247	Synaptophysin	SYP	3.40	-1.13723	-1.7484	-0.9631	-1.76783		-1.80665	-1.48	0.40
P25189	Myelin protein P0	MPZ	3.00	-0.6887	-1.00038			-2.52587	-1.89535	-1.53	0.84
A0A087WWD4	Neural cell adhesion molecule 1	NCAM1	9.33	-1.64157	-1.37608	-1.16463	-1.99172	-1.59852	-1.40415	-1.53	0.28
P21796	Voltage-dependent anion-selective channel protein 1	VDAC1	29.00	-1.46926	-1.38399	-2.06146	-2.12681	-1.14001	-1.03722	-1.54	0.46
Q5SSJ5	Heterochromatin protein 1-binding protein 3	HP1BP3	4.83	-1.51297	-1.2538	-2.13756	-2.00753	-1.14556	-1.33656	-1.57	0.41
P05109	Protein S100-A8	S100A8	1.33	-0.73218	-0.64801	-1.5924	-1.54839	-2.52185	-2.3978	-1.57	0.79
A0A024QZ64	Fructose-bisphosphate aldolase	ALDOC	29.40	-1.13636	-1.60061		-1.49243	-2.01299	-1.71889	-1.59	0.32
Q8N169	Amino acid transporter	SLC1A3	3.33	-1.35357				-2.00792	-1.41957	-1.59	0.36
J3KRC4	5'(3')-deoxyribonucleotidase, cytosolic type	NT5C	2.00	-0.82887	-1.40898	-2.31084			-1.83965	-1.60	0.63
G4XXL9	Cytochrome c	CYCS	9.50	-1.25961	-1.82839	-2.06256	-2.21219	-0.9856	-1.27986	-1.60	0.50
A0A024R9X3	Cyclic nucleotide gated channel alpha 1, isoform CRA_a	CNGA1	7.00	-2.63117	-0.77447	-1.38505	-1.73586			-1.63	0.78
P02489	Alpha-crystallin A chain	CRYAA	9.67	-1.10681	-1.04082	-2.43148	-2.1051	-1.53111	-1.62654	-1.64	0.55
G3V4I5	GSK3-beta interaction protein (Fragment)	GSKIP	1.00	-1.70049	-1.86319	-1.29644			-1.70916	-1.64	0.24
Q6FI13	Histone H2A type 2-A	HIST2H2AA3	8.50	-1.68269		-1.67041				-1.68	0.01
Q6I9V5	SLC25A6 protein	SLC25A6	15.67	-1.1183	-0.92528	-2.14692	-2.18556	-1.92277	-1.8116	-1.69	0.54
Q3ZCW2	Galectin-related protein	LGALSL	1.00				-2.06483		-1.32023	-1.69	0.53
A0A024R056	Guanine nucleotide binding protein (G protein), beta polypeptide 1, isoform CR/	GNB1	36.50	-1.24042		-2.15752				-1.70	0.65
P35913	Rod cGMP-specific 3',5'-cyclic phosphodiesterase subunit beta	PDE6B	13.17	-1.48295	-1.20485	-1.79916	-1.8682	-2.15332	-1.80665	-1.72	0.33
B2R6K4	cDNA, FLJ92996, highly similar to Homo sapiens guanine nucleotide binding pro	NA	26.50		-1.23581		-2.11192	-1.91537	-1.62654	-1.72	0.38
U3KQK0	Histone H2B	HIST1H2BN	19.20	-2.80873		-1.71148	-2.28355	-1.01383	-0.83007	-1.73	0.84
A0A024R5Z9	Pyruvate kinase	PKM2	64.80		-2.15032	-1.56313	-1.58272	-1.62802	-1.86826	-1.76	0.25
P10745	Retinol-binding protein 3	RBP3	23.67	-2.2667	-1.0768	-1.76093	-1.6656	-1.88047	-2.15628	-1.80	0.42
V9HWF6	Alpha-1-acid glycoprotein	HEL-S-153w	1.33	-2.86212	-1.29234	-1.2984				-1.82	0.90
Q13509	Tubulin beta-3 chain	TUBB3	31.50				-0.61991		-3.02394	-1.82	1.70
Q05639	Elongation factor 1-alpha 2	EEF1A2	10.00	-1.40686	-2.82032	-1.24842				-1.83	0.87
P12532	Creatine kinase U-type, mitochondrial	CKMT1A	14.00	-1.43373	-0.96512	-3.03515	-2.8866	-1.04378	-1.70639	-1.85	0.91
B2R4M6	Protein S100	NA	2.20		-0.53341	-1.98707	-1.73938	-2.31433	-2.80467	-1.88	0.85
Q13885	Tubulin beta-2A chain	TUBB2A	44.67	-2.39853	-1.94854	-1.88467	-1.53993	-1.81408	-1.95273	-1.92	0.28
Q96QV6	Histone H2A type 1-A	HIST1H2AA	7.67		-3.50592			-1.32235	-1.34085	-2.06	1.26
F1T0F7	Cellular retinoic acid binding protein 1	CRABP1	14.67	-1.94762	-1.89531	-2.41442	-2.5021	-2.50271	-2.71843	-2.33	0.33
B4DF39	Receptor expression-enhancing protein	NA	1.00	-2.36265		-2.37264				-2.37	0.01
V9HWE3	Carbonic anhydrase I, isoform CRA_a	CA1	15.00	-2.21643	-1.73065	-2.22655	-2.21463	-3.28338	-2.73245	-2.40	0.54
Q6FHV6	ENO2 protein	ENO2	53.67	-2.494	-2.16301	-2.25917	-2.47107	-2.57334	-2.51894	-2.41	0.16
V9HW21	Epididymis luminal protein 76	CA2	21.50	-2.24975	-2.1827	-2.546	-2.62519	-2.57167	-2.38963	-2.43	0.18
V9HWH2	Epididymis secretory protein Li 29	CKB	50.00	-2.16256	-2.32478	-2.1682	-2.70202	-3.12377	-3	-2.58	0.42
Q16518	Retinoid isomerohydrolase	RPE65	24.67	-2.31095	-2.40194	-3.02432	-2.82179	-2.6966	-2.3266	-2.60	0.30
A0A024R2Z1	Guanine nucleotide binding protein (G protein), alpha transducing activity polyp	GNAT1	25.83	-1.88085	-2.19917	-3.03732	-3.23807	-2.34241	-2.8922	-2.60	0.53
P63211	Guanine nucleotide-binding protein G(T) subunit gamma-T1	GNGT1	7.67	-2.13723	-2.4447	-2.92436	-3.24056	-2.59344	-2.40752	-2.62	0.40

				08/023 hESC-RPE to hRPE							
				Analysis							
UniProt	Protein name	Gene name	Peptide mean	1.1	1.2	2.1	2.2	3.1	3.2	Fold change (mean)	StDev
J3KS22	L-xylulose reductase (Fragment)	DCXR	3.50	-0.88021	-1.07624	-1.67853	-0.57791			-1.05	0.46
P51970	NADH dehydrogenase [ubiquinone] 1 alpha subcomplex subunit 8	NDUFA8	4.50	-0.86059	-0.83466	-1.31713	-1.20666			-1.05	0.24
C9JF17	Apolipoprotein D (Fragment)	APOD	1.75	-1.99301	0.503291	-1.11232	-1.6332			-1.06	1.10
Q6DEN2	DPYSL3 protein	DPYSL3	21.00	-1.15874	-0.63427	-1.04081	-0.9917	-1.1075	-1.43281	-1.06	0.26
Q8TBT6	Putative uncharacterized protein (Fragment)	NA	7.00	-0.5142	-0.70472	-1.4876	-1.89984	-0.94105	-0.8798	-1.07	0.52
Q59FD4	Hexokinase (Fragment)	NA	24.17	-1.07465	-1.03502	-1.25783	-1.27953	-0.76417	-1.01994	-1.07	0.19
O60262	Guanine nucleotide-binding protein G(I)/G(S)/G(O) subunit gamma-7	GNG7	1.00	0.251337	-1.01084	-1.96327	-1.44624		-1.19612	-1.07	0.82
Q9BTT5	Similar to NADH dehydrogenase (Ubiquinone) 1 alpha subcomplex, 9 (39kD) (Fragment)	NA	5.80	-0.45053	0.335133	-1.63151	-0.88299	-2.74464		-1.07	1.17
Q6LET3	HPRT1 protein (Fragment)	HPRT1	3.00	-0.8373	-0.99582	-0.53999	-1.4387	-1.75437	-0.89524	-1.08	0.44
B7Z1V4	cDNA FLJ56034, highly similar to 4-aminobutyrate aminotransferase, mitochondrial (EC 2.6.1.19)	NA	3.00				-1.50519	-0.65172		-1.08	0.60
B4E192	cDNA FLJ54224, highly similar to Glial fibrillary acidic protein, astrocyte	NA	15.00	-1.16757	-0.98968	-1.1189				-1.09	0.09
P56134	ATP synthase subunit f, mitochondrial	ATP5J2	3.50	-0.7835	-0.353	-1.36782	-1.87279			-1.09	0.67
A0A024R9B7	Cytochrome c oxidase subunit VIc, isoform CRA_a	COX6C	2.00	-0.91285	-1.10061	-1.0455	-1.66646	-0.69961	-1.17325	-1.10	0.32
Q9H3N1	Thioredoxin-related transmembrane protein 1	TMX1	1.50		-0.33955	-1.90335				-1.12	1.11
Q6FHT8	RNP24 protein	RNP24	2.33		0.410016		-2.77714	-1.0244		-1.13	1.60
Q9H936	Mitochondrial glutamate carrier 1	SLC25A22	3.00		-1.69078	-1.14105	-0.60453			-1.15	0.54
Q8WYJ5	Protein kinase C inhibitor-2	NA	2.60	-0.93279	-2.43464	-0.54294	-1.14733		-0.68823	-1.15	0.75
O75475	PC4 and SFRS1-interacting protein	PSIP1	2.80	-2.06912	-1.28216	-0.7406	-0.94285		-0.81207	-1.17	0.54
A0A024RB75	Citrate synthase	CS	14.00	-0.57003	-0.90101	-1.75334	-1.73873	-0.8846	-1.21141	-1.18	0.49
A0A024R9X3	Cyclic nucleotide gated channel alpha 1, isoform CRA_a	CNGA1	7.00	-0.35523	-1.15895	-1.50258	-1.69641			-1.18	0.59
D6RIE3	Cytochrome c oxidase subunit 7A2, mitochondrial	COX7A2	2.60	-0.83652	-0.98487	-1.70221	-1.50519	-0.91313		-1.19	0.39
Q05639	Elongation factor 1-alpha 2	EEF1A2	10.00	-1.7233	-1.2535	-0.5913				-1.19	0.57
P16403	Histone H1.2	HIST1H1C	14.67		-1.87249	-0.87106	-0.82909			-1.19	0.59
A0A024RB18	Retinol dehydrogenase 5 (11-cis and 9-cis), isoform CRA_a	RDH5	10.67	-1.30267	-1.04681	-1.53375	-1.03603	-0.97601	-1.2732	-1.19	0.21
Q3ZCW2	Galectin-related protein	LGALSL	1.00				-1.45937		-0.93153	-1.20	0.37
V9HW31	ATP synthase subunit beta	ATP5B	65.17	-0.94244	-0.83034	-1.933	-2.05898	-0.71922	-0.6955	-1.20	0.63
P12235	ADP/ATP translocase 1	SLC25A4	14.50	-0.25221	-0.62269	-2.33107	-1.79629	-1.32027	-0.86412	-1.20	0.77
P07305	Histone H1.0	H1F0	2.17	-1.58474	-1.29022	-0.69564	-0.69477	-1.5761	-1.44209	-1.21	0.42
P17568	NADH dehydrogenase [ubiquinone] 1 beta subcomplex subunit 7	NDUFB7	1.00	-1.1854	-0.52022	-1.49045	-0.34662	-2.55529		-1.22	0.88
X5DQV1	Collapsin response mediator protein 1 isoform B (Fragment)	CRMP1	10.67	-1.59067		-0.80192			-1.2701	-1.22	0.40
P16499	Rod cGMP-specific 3',5'-cyclic phosphodiesterase subunit alpha	PDE6A	16.67	-1.40282	-0.90307	-1.57236	-1.65291	-0.45239	-1.39741	-1.23	0.46
Q53ES0	Sodium/potassium-transporting ATPase subunit alpha (Fragment)	NA	40.67	-1.01719	-1.06283	-1.25662	-1.41025	-1.25269	-1.4485	-1.24	0.18
A0A0C4DGE4	Syntaxin-3 (Fragment)	STX3	5.00		-0.80202		-1.68419			-1.24	0.62
Q9UNL6	Hemoglobin gamma-G (Fragment)	HBG2	11.50			-0.91714			-1.57228	-1.24	0.46
A0A024R4F1	Enolase 1, (Alpha), isoform CRA_a	ENO1	61.00	-1.14704	-0.97358	-1.0099	-0.9937	-1.72331	-1.65954	-1.25	0.35
B7Z8A2	cDNA FLJ51671, highly similar to Prenylcysteine oxidase (EC 1.8.3.5)	NA	1.50		-1.40224	-1.1156				-1.26	0.20
Q9Y3D6	Mitochondrial fission 1 protein	FIS1	2.00		0.099609	-2.42443	-1.48457			-1.27	1.28
Q03395	Rod outer segment membrane protein 1	ROM1	6.83	-0.5603	-0.89403	-1.90999	-2.04433	-1.56749	-0.69758	-1.28	0.64
Q68CM6	STXBP1 protein	stxbp1	14.83	-1.2146	-1.53069	-0.93736	-1.14175	-1.50924	-1.35682	-1.28	0.23
Q59GB4	Dihydropyrimidinase-like 2 variant (Fragment)	NA	19.83	-1.86138	-1.82565	-0.79928	-1.4049	-0.87547	-0.99072	-1.29	0.48
G4V2I9	Anion exchange protein	NA	6.50	-1.12537	-1.14572	-1.47208	-1.84623	-1.10795	-1.06447	-1.29	0.31
P05109	Protein S100-A8	S100A8	1.33	-0.49832	0.144159	-1.4136	-1.15237	-2.75298	-2.29615	-1.33	1.08
H0YJ66	Dehydrogenase/reductase SDR family member 7 (Fragment)	DHRS7	1.00		-1.12203	-1.2706	-2.23668		-0.72563	-1.34	0.64
P47804	RPE-retinal G protein-coupled receptor	RGR	6.83	-1.55482	-1.41394	-1.64174	-2.03396	-0.59097	-0.81132	-1.34	0.54
Q17R60	Interphotoreceptor matrix proteoglycan 1	IMPG1	4.80	-0.92778	-1.02736	-2.11286	-1.89608	-0.84878		-1.36	0.59
Q8NCF7	cDNA FLJ90278 fis, clone NT2RP1000325, highly similar to Phosphate carrier protein, mitochondri	NA	7.40	-1.23973	-1.22386	-2.06549		-1.15138	-1.14031	-1.36	0.39
A0A024QZT0	Voltage-dependent anion channel 2, isoform CRA_c	VDAC2	16.17	-0.96836	-1.07068	-2.15733	-2.04641	-1.02945	-1.00182	-1.38	0.56
B3KV96	cDNA FLJ16285 fis, clone OCBBF2004038, highly similar to Dihydropyrimidinase-related protein 1	NA	9.33		-1.67591		-1.19333	-1.2876		-1.39	0.26
Q53XX5	Cold inducible RNA binding protein	CIRBP	3.00	-0.97007	-1.29453			-1.64492	-1.63473	-1.39	0.32
U3KQK0	Histone H2B	HIST1H2BN	19.20	-1.88304		-1.12827	-1.45937	-1.27654	-1.23238	-1.40	0.30
Q5SSJ5	Heterochromatin protein 1-binding protein 3	HP1BP3	4.83	-1.48079	-1.02198	-1.40353	-1.4881	-1.705	-1.35025	-1.41	0.22
P19404	NADH dehydrogenase [ubiquinone] flavoprotein 2, mitochondrial	NDUFV2	3.00	-1.08623	-0.77311	-1.53522	-1.54146	-1.14223	-2.41215	-1.42	0.57
Q6NVC0	SLC25A5 protein (Fragment)	SLC25A5	14.67	-1.02877	-1.14378	-2.04969	-2.15686	-1.08891	-1.02909	-1.42	0.54
B2R6K4	cDNA, FLJ92996, highly similar to Homo sapiens guanine nucleotide binding protein (G protein), b	NA	26.50		-1.21821		-1.93222	-1.32027	-1.19858	-1.42	0.35
B4DFY5	Mitogen-activated protein kinase kinase 1, isoform CRA_d	MAP2K1	1.00	-0.53437	-2.48186				-1.25622	-1.42	0.98
P21796	Voltage-dependent anion-selective channel protein 1	VDAC1	29.00	-1.38325	-1.35695	-2.00838	-1.99421	-0.99278	-0.87351	-1.43	0.48
Q8IZ29	Tubulin, beta 2C	TUBB2C	54.83	-1.57361	-1.81631	-1.14664	-0.91261	-1.43859	-1.77716	-1.44	0.36
Q5T697	Guanine nucleotide binding protein (G protein), alpha transducing activity polypeptide 2	GNAT2	12.00		-2.08697	-0.81879				-1.45	0.90
Q14894	Ketimine reductase mu-crystallin	CRYM	11.67	-0.75734	-1.10344	-1.65602	-1.83448	-2.00435	-1.4042	-1.46	0.47
Q53YE2	Syntaxin 3A	STX3A	3.25	-1.08949		-2.14945		-1.10084	-1.50147	-1.46	0.50
V9HWF6	Alpha-1-acid glycoprotein	HEL-S-153w	1.33	-1.68686	-1.89772	-0.84037				-1.47	0.56
G3V4I5	GSK3-beta interaction protein (Fragment)	GSKIP	1.00	-1.23045	-1.94445	-1.30707			-1.46259	-1.49	0.32
P08247	Synaptophysin	SYP	3.40	-1.22583	-1.90101	-0.92721	-1.54293		-1.89087	-1.50	0.42
D3GKD8	A-gamma globin Osilo variant	HBG1	16.25	-1.14365	-1.93768		-1.37841	-1.54862		-1.50	0.33
Q6I9V5	SLC25A6 protein	SLC25A6	15.67	-1.00967	-0.95766	-2.70386	-2.48386	-1.19664	-0.66395	-1.50	0.87
O14561	Acyl carrier protein, mitochondrial	NDUFAB1	3.00	-1.40921	-0.66051	-1.7406	-2.27037			-1.52	0.67
A0A087WWD4	Neural cell adhesion molecule 1	NCAM1	9.33	-1.60994	-1.32237	-1.1156	-1.61918	-1.89111	-1.69584	-1.54	0.28
Q71UI9	Histone H2A.V	H2AFV	5.17	-1.62405	-1.8822	-0.81879	-1.61531	-1.63911	-1.69723	-1.55	0.37
A0A024R056	Guanine nucleotide binding protein (G protein), beta polypeptide 1, isoform CRA_a	GNB1	36.50	-1.27165		-1.9301				-1.60	0.47
P25189	Myelin protein P0	MPZ	3.00	-0.70104	-1.13359			-2.66117	-1.93767	-1.61	0.87
B4DHC6	Amino acid transporter	NA	5.33		-1.98139	-1.69728	-1.18413			-1.62	0.40
Q4VB24	Histone cluster 1, H1e	HIST1H1E	16.00	-1.75475				-1.49227		-1.62	0.19
B2R4M6	Protein S100	NA	2.20		0.153897	-1.67691	-1.12734	-3.03707	-2.4691	-1.63	1.24
A0A024QZ64	Fructose-bisphosphate aldolase	ALDOC	29.40	-1.46437	-1.82175		-1.46563	-1.74741	-1.70001	-1.64	0.17
V9HW12	Epididymis secretory sperm binding protein Li 2a	PRDX2	18.50	-1.6472	-1.57596	-1.55739	-1.35821	-1.93471	-1.80607	-1.65	0.20
P35913	Rod cGMP-specific 3',5'-cyclic phosphodiesterase subunit beta	PDE6B	13.17	-1.6153	-1.28323	-1.61591	-1.60376	-2.01434	-1.77057	-1.65	0.24
Q0Z7D8	Cytochrome c oxidase subunit 2	COX2	3.17	-1.2743	-1.16635	-2.29958	-2.48386	-1.41524	-1.28306	-1.65	0.58
F5H157	Ras-related protein Rab-35 (Fragment)	RAB35	3.75	-2.67772	-2.47572		-1.34021	-0.1404		-1.66	1.17
P02489	Alpha-crystallin A chain	CRYAA	9.67	-1.06636	-1.02826	-1.92145	-1.96239	-2.0942	-1.89325	-1.66	0.48
G4XXL9	Cytochrome c	CYCS	9.50	-1.3696	-1.69007	-2.04341	-2.43938	-1.25614	-1.35244	-1.69	0.47
P12532	Creatine kinase U-type, mitochondrial	CKMT1A	14.00	-1.41972	-0.94276	-2.85861	-2.4881	-1.38739	-1.9238	-1.84	0.73
J3KRC4	5'(3')-deoxyribonucleotidase, cytosolic type	NT5C	2.00	-1.71464	-1.39933	-3.38954			-0.92055	-1.86	1.07
P10745	Retinol-binding protein 3	RBP3	23.67	-2.44873	-1.2085	-1.94952	-1.54514	-1.85102	-2.31733	-1.89	0.47
H0Y695	Aminomethyltransferase (Fragment)	AMT	1.33	-0.6844		-2.83856	-2.25463			-1.93	1.11
A0A024R5Z9	Pyruvate kinase	PKM2	64.80		-2.58518	-1.72629	-1.86266	-1.65465	-1.9759	-1.96	0.37
Q13885	Tubulin beta-2A chain	TUBB2A	44.67	-2.43564	-2.43821	-2.15959	-1.91594	-1.60286	-2.12856	-2.11	0.32
Q8N169	Amino acid transporter	SLC1A3	3.33	-1.6527				-2.50395	-2.32375	-2.16	0.45
V9HW21	Epididymis luminal protein 76	CA2	21.50	-2.30429	-2.39006	-2.01347	-1.81926	-2.49227	-2.22036	-2.21	0.25
F1T0F7	Cellular retinoic acid binding protein 1	CRABP1	14.67	-1.96023	-1.8382	-2.43671	-2.37121	-2.55286	-2.7154	-2.31	0.34
V9HWE3	Carbonic anhydrase I, isoform CRA_a	CA1	15.00	-2.24908	-1.87572	-2.09762	-2.11145	-3.06969	-2.50876	-2.32	0.42
Q96QV6	Histone H2A type 1-A	HIST1H2AA	7.67		-2.9504			-2.06449	-2.05732	-2.36	0.51
Q6FHV6	ENO2 protein	ENO2	53.67	-2.53754	-2.1394	-2.30707	-2.41226	-2.41524	-2.35189	-2.36	0.13
V9HWH2	Epididymis secretory protein Li 29	CKB	50.00	-2.373	-2.45987	-2.32092	-2.51815	-2.55043	-2.50876	-2.46	0.09
B4DF39	Receptor expression-enhancing protein	NA	1.00	-2.71752		-2.21256				-2.47	0.36
A0A024R2Z1	Guanine nucleotide binding protein (G protein), alpha transducing activity polypeptide 1, isoform	GNAT1	25.83	-1.92403	-2.42514	-2.50833	-2.54883	-2.55407	-3.19661	-2.53	0.41
Q16518	Retinoid isomerohydrolase	RPE65	24.67	-2.48018	-2.37398	-3.01347	-2.94966	-2.64686	-2.2344	-2.62	0.31
P63211	Guanine nucleotide-binding protein G(T) subunit gamma-T1	GNGT1	7.67	-2.36508	-2.48063	-2.70551	-3.21895	-2.83175	-2.6281	-2.71	0.30

Supplementary Table S3. The GO terms used for classification of the proteins.
The last column specifies the GO domain of the GO term:
cellular component (CC), biological process (BP), molecular function (MF).

GO term identifier	GO term name	GO domain
GO:0009583	detection of light stimulus	BP
GO:0050953	sensory perception of light stimulus	BP
GO:0043473	pigmentation	BP
GO:0006582	melanin metabolic process	BP
GO:0042470	melanosome	CC
GO:0031012	extracellular matrix	CC
GO:0007155	cell adhesion	BP
GO:0005201	extracellular matrix structural constituent	MF
GO:1990430	extracellular matrix protein binding	MF
GO:0006909	phagocytosis	BP
GO:0006508	proteolysis	BP
GO:0006914	autophagy	BP
GO:0006897	endocytosis	BP
GO:0000502	proteasome	CC
GO:0034702	ion channel complex	CC
GO:1990351	transporter complex	CC
GO:0005215	transporter activity	MF
GO:0005216	ion channel activity	MF
GO:0005739	mitochondrion	CC
GO:0006950	response to stress	BP
GO:0006979	response to oxidative stress	BP
GO:0002376	immune system process	BP
GO:0006955	immune response	BP
GO:0005856	cytoskeleton	CC
GO:0005198	structural molecule activity	MF
GO:0007010	cytoskeleton organization	BP

Supplementary Table S4. The 190 mitochondrial proteins presented in Figure 4a. Differences in expression for both hESC-RPE lines compared to hRPE presented on log2 scale.
Mean number of peptides used for quantification,fold change value for each replicate analysis, technical replicate CV, biological replicate CV, mean fold change, and standard deviation shown for each protein. Note! CV values are calculated using normal scale, not the log2 scale shown here.
Differentially expressed proteins (>2-fold difference in expression i.e. values >1 and <-1 on log2 scale) marked with red color for upregulation and green color for downregulation.

				08/017 hESC-RPE to hRPE												08/023 hESC-RPE to hRPE										
				Analysis						Technical replicate CV						Analysis						Technical replicate CV				
UniProt	Protein name	Gene name	Peptide mean	1.1	1.2	2.1	2.2	3.1	3.2	1	2	3	Biological CV	Fold change (mean)	StDev	1.1	1.2	2.1	2.2	3.1	3.2	1	2	3	Biological CV	Fold change (mean)	StDev
P61604	10 kDa heat shock protein, mitochondrial	HSPE1	7.33	0.52446	0.484206	NA	NA	0.901593	NA	1.97	NA	NA	19.34	0.64	0.23	0.917834	0.766584	NA	NA	0.606649	NA	7.41	NA	NA	11.62	0.76	0.16
P61981	14-3-3 protein gamma	YWHAG	8.67	-0.27002	-0.57918	0.147277	-0.10255	-1.99226	-2.10735	15.10	12.21	5.64	58.92	-0.82	0.98	-0.01542	-0.1718	0.166406	-0.04407	-0.27404	-0.74559	7.66	10.30	22.91	18.99	-0.18	0.31
P27348	14-3-3 protein theta	YWHAQ	8.80	NA	0.593291	1.080665	-0.39319	-0.33958	-0.99802	NA	66.55	31.72	40.03	-0.01	0.83	NA	0.262594	0.993787	-0.0791	0.073567	-0.17833	NA	50.29	12.31	20.68	0.21	0.47
A0A024R9D7	2,4-dienoyl CoA reductase 1, mitochondrial, isoform CRA_b	DECR1	1.00	NA	NA	-0.43434	NA	-1.08145	NA	NA	NA	NA	31.20	-0.76	0.46	NA	NA	-1.00127	NA	-0.13018	NA	NA	NA	NA	41.44	-0.57	0.62
E9PCR7	2-oxoglutarate dehydrogenase, mitochondrial	OGDH	10.67	-0.16344	0.146621	-0.7822	-0.53343	-0.57917	-0.5232	15.14	12.16	2.74	25.61	-0.41	0.34	-0.01232	0.221794	-0.7142	-0.75738	0.016448	-0.60675	11.45	2.12	30.08	28.55	-0.31	0.43
Q99714	3-hydroxyacyl-CoA dehydrogenase type-2	HSD17B10	3.80	0.491936	0.319496	NA	0.53294	0.571598	0.01742	8.44	NA	26.83	7.43	0.39	0.23	0.160937	0.202774	NA	0.42378	0.058356	-0.38646	2.05	NA	21.63	19.47	0.09	0.30
A0A024RA75	3-hydroxyisobutyrate dehydrogenase	HIBADH	9.67	1.898692	1.364941	-0.14956	0.114863	0.488151	0.359647	25.87	12.92	6.29	63.37	0.68	0.79	1.610518	1.316762	-0.14496	0.11048	0.570763	0.313145	14.35	12.49	12.59	54.94	0.63	0.69
Q6NVY1	3-hydroxyisobutyryl-CoA hydrolase, mitochondrial	HIBCH	2.33	-0.57731	NA	-0.53828	-0.81065	NA	NA	NA	13.31	NA	4.45	-0.64	0.15	-1.09136	NA	-1.40956	-0.52068	NA	NA	NA	42.24	NA	9.48	-1.01	0.45
P23396	40S ribosomal protein S3	RPS3	5.50	0.368071	0.286259	0.250701	0.427314	0.435787	0.705996	4.01	8.65	13.21	9.97	0.41	0.16	0.309782	0.47424	0.295315	0.285072	0.533677	0.559221	8.05	0.50	1.25	9.00	0.41	0.13
P62906	60S ribosomal protein L10a	RPL10A	2.40	-0.26268	0.10469	0.119976	NA	0.518329	0.433295	17.91	NA	4.17	19.58	0.18	0.31	-0.06751	0.378664	0.264889	NA	0.507588	0.517354	21.70	NA	0.48	12.45	0.32	0.24
P18077	60S ribosomal protein L35a	RPL35A	1.00	0.47107	NA	-0.1329	0.483264	NA	NA	NA	29.75	NA	12.87	0.27	0.35	0.522113	NA	0.371232	0.563522	NA	NA	NA	9.41	NA	2.53	0.49	0.10
P24752	Acetyl-CoA acetyltransferase, mitochondrial	ACAT1	10.50	0.839969	0.62773	-0.12017	0.124568	0.739578	0.174817	10.38	11.97	27.33	24.55	0.40	0.39	0.670465	0.348867	-0.39286	0.105563	0.501028	0.128062	15.70	24.19	18.18	21.71	0.23	0.37
Q86YI5	Acetyltransferase component of pyruvate dehydrogenase complex	DLAT	4.00	-0.44066	-0.8083	-0.68909	-0.97919	-0.65045	-0.75517	17.92	14.17	5.13	7.40	-0.72	0.18	-0.52519	-0.5988	-0.90857	-1.00025	-0.63686	-0.80009	3.61	4.49	7.99	13.47	-0.74	0.19
V9HWB7	Aconitate hydratase	ACO1	5.50	0.835674	1.321169	0.301481	0.738735	0.102966	0.451393	23.57	21.27	16.99	29.88	0.63	0.44	0.173038	0.768397	0.498132	1.293745	0.008453	0.834577	28.77	38.04	39.42	19.26	0.60	0.47
A2A274	Aconitate hydratase, mitochondrial	ACO2	21.50	-0.55515	-0.55723	-0.83229	-1.0386	-0.40971	-0.4109	0.10	10.09	0.06	17.88	-0.63	0.25	-0.49246	-0.40311	-1.00634	-1.17441	-0.33246	-0.46023	4.38	8.23	6.26	24.47	-0.64	0.35
O14561	Acyl carrier protein, mitochondrial	NDUFAB1	3.00	-0.76292	-1.04238	-1.43505	-1.79593	NA	NA	13.65	17.60	NA	34.04	-1.26	0.45	-1.40921	-0.66051	-1.7406	-2.27037	NA	NA	35.89	25.68	NA	46.90	-1.52	0.67
P07108	Acyl-CoA-binding protein	DBI	4.50	0.826823	0.892953	0.118024	0.213197	0.304759	0.334088	3.24	4.66	1.44	26.47	0.45	0.33	0.348502	0.29672	0.218664	0.602769	0.284687	0.296855	2.54	18.72	0.60	4.85	0.34	0.13
Q5T4U5	Acyl-Coenzyme A dehydrogenase, C-4 to C-12 straight chain, isoform CRA_a	ACADM	6.00	0.258302	NA	0.134537	NA	NA	NA	NA	NA	NA	6.06	0.20	0.09	0.601376	NA	-0.10522	NA	NA	NA	NA	NA	NA	33.96	0.25	0.50
Q9NPJ3	Acyl-coenzyme A thioesterase 13	ACOT13	1.20	-0.35609	0.27045	-0.72019	-0.86195	-0.34561	NA	30.23	6.94	NA	26.39	-0.40	0.44	-1.12489	-0.40691	-0.47102	-0.08899	-0.07861	NA	34.48	18.62	NA	21.79	-0.43	0.43
P54819	Adenylate kinase 2, mitochondrial	AK2	7.67	0.637852	-0.09129	-0.6231	-0.35425	0.122102	-0.2084	35.00	13.14	16.13	27.12	-0.09	0.43	0.374258	0.125494	-0.71794	-0.36763	0.514987	0.879724	12.16	17.09	17.78	40.22	0.13	0.59
P12235	ADP/ATP translocase 1	SLC25A4	14.50	-0.24764	-0.59029	-2.06035	-1.51109	-1.47486	-0.79508	16.72	26.60	32.71	45.75	-1.11	0.68	-0.25221	-0.62269	-2.33107	-1.79629	-1.32027	-0.86412	18.06	25.91	22.17	51.45	-1.20	0.77
A0A024RAD8	Aldehyde dehydrogenase 4 family, member A1, isoform CRA_a	ALDH4A1	3.17	-0.5068	-0.29909	-0.64656	-0.28709	-0.575	-0.50523	10.16	17.53	3.42	4.88	-0.47	0.15	-0.70926	-0.5454	-0.3317	-0.43938	-0.2924	-0.2776	8.02	5.28	0.73	11.82	-0.43	0.17
X5DQN2	Aldehyde dehydrogenase 5 family member A1 isoform B (Fragment)	ALDH5A1	5.33	-0.10723	NA	-0.4813	-0.86291	NA	NA	NA	18.60	NA	26.74	-0.48	0.38	0.352112	NA	-1.00431	-1.57115	NA	NA	NA	27.43	NA	71.71	-0.74	0.99
A0A024R6G4	Aldehyde dehydrogenase 6 family, member A1, isoform CRA_b	ALDH6A1	7.80	-0.02542	-0.1205	0.16204	0.556614	-0.13294	NA	4.66	19.22	NA	20.01	0.09	0.29	-0.05221	-0.04817	0.063613	0.450249	0.045791	NA	0.20	18.84	NA	11.60	0.09	0.21
B9EKV4	Aldehyde dehydrogenase 9 family, member A1	ALDH9A1	12.50	0.625528	0.2808	0.160381	0.181003	0.412555	0.342918	16.82	1.01	3.41	10.21	0.33	0.17	0.910043	0.491113	0.120588	0.413586	0.350388	0.278262	20.39	14.31	3.53	17.62	0.43	0.27
V9HWA2	Aldo-keto reductase family 7, member A2 (Aflatoxin aldehyde reductase)	AKR7A2	1.50	1.64019	NA	-0.26521	-0.40815	0.672137	NA	NA	7.00	NA	64.36	0.41	0.95	1.422795	NA	-0.55627	-0.9619	0.629551	NA	NA	19.75	NA	64.88	0.13	1.09
P49419	Alpha-aminoadipic semialdehyde dehydrogenase	ALDH7A1	6.33	0.809743	0.542454	0.585228	0.285924	0.726373	-0.77172	13.06	14.62	67.47	17.80	0.36	0.58	0.542566	0.18078	0.363779	0.311951	0.763721	-0.54797	17.64	2.54	60.20	4.28	0.27	0.45
Q53YE7	Amine oxidase [flavin-containing]	MAOA	3.20	-1.40165	-1.11947	-1.15516	-1.17243	NA	-1.00283	13.79	0.85	NA	8.90	-1.17	0.15	0.076595	-0.73851	-1.02321	-1.07218	NA	-0.90644	38.92	2.40	NA	30.16	-0.73	0.47
Q8TBI1	Amine oxidase [flavin-containing]	MAOB	1.67	0.214016	NA	NA	0.307386	-0.09706	NA	NA	NA	NA	14.15	0.14	0.21	0.262523	NA	NA	0.055933	0.500005	NA	NA	NA	NA	15.44	0.27	0.22
Q5TZZ9	Annexin	ANXA1	12.67	0.454789	0.563415	-0.19253	-0.46524	-0.71576	-0.50624	5.32	13.33	10.25	42.52	-0.14	0.53	0.193686	0.344362	0.56637	0.372852	0.310761	0.399269	7.38	9.47	4.34	7.06	0.36	0.12
Q5TZP7	APEX nuclease (Multifunctional DNA repair enzyme) 1	APEX1	5.80	-0.68237	-0.72939	0.529209	NA	-0.90827	-1.00737	2.30	NA	4.86	59.48	-0.56	0.62	-0.71572	-0.60851	1.016231	NA	-0.99566	-0.74165	5.25	NA	12.42	77.47	-0.41	0.81
O95831	Apoptosis-inducing factor 1, mitochondrial	AIFM1	10.67	-0.20686	-0.55221	-1.46109	-0.91167	-0.53779	-0.0817	16.85	26.61	22.17	29.75	-0.63	0.50	-0.07465	-0.46656	-1.32789	-0.79849	-0.46091	-0.26263	19.09	25.66	9.70	26.80	-0.57	0.44
V9HW31	ATP synthase subunit beta	ATP5B	65.17	-0.92464	-0.93214	-1.75197	-1.9082	-1.07407	-0.95739	0.37	7.65	5.72	30.60	-1.26	0.45	-0.94244	-0.83034	-1.933	-2.05898	-0.71922	-0.6955	5.49	6.17	1.16	40.90	-1.20	0.63
O75947	ATP synthase subunit d, mitochondrial	ATP5H	10.83	-0.64311	-1.0145	-1.4123	-1.40605	-0.55535	-1.0898	18.10	0.31	25.90	22.23	-1.02	0.36	-0.72874	-0.73337	-1.41765	-1.43938	-0.17243	-0.59895	0.23	1.06	20.75	34.65	-0.85	0.49
P30049	ATP synthase subunit delta, mitochondrial	ATP5D	4.83	-0.33756	-0.58316	-1.65781	-1.55225	-0.32988	-0.15015	12.01	5.17	8.80	42.82	-0.77	0.66	0.102338	-0.06906	-1.42239	-1.28998	-0.16569	0.046867	8.39	6.49	10.40	43.80	-0.47	0.70
P56385	ATP synthase subunit e, mitochondrial	ATP5I	1.33	-0.01206	-0.12236	-0.72588	-0.26982	-0.65925	-0.27507	5.40	22.17	18.72	16.73	-0.34	0.29	-0.17201	0.196057	-0.52462	-0.34726	0.302401	0.063148	17.94	8.68	11.70	21.14	-0.08	0.32
P56134	ATP synthase subunit f, mitochondrial	ATP5J2	3.50	-0.89469	-0.60985	-0.90537	-1.21402	NA	NA	13.92	15.07	NA	14.95	-0.91	0.25	-0.7835	-0.353	-1.36782	-1.87279	NA	NA	20.95	24.50	NA	49.14	-1.09	0.67
O75964	ATP synthase subunit g, mitochondrial	ATP5L	2.75	-0.39051	NA	-0.821	-0.39735	-0.34259	NA	NA	20.62	NA	9.02	-0.49	0.22	-0.42119	NA	-0.43158	-0.21367	-0.50865	NA	NA	10.66	NA	6.61	-0.39	0.13
P48047	ATP synthase subunit O, mitochondrial	ATP5O	11.17	-0.04458	-0.27915	-1.37058	-1.28548	0.019922	0.208476	11.47	4.17	9.23	44.70	-0.46	0.69	0.060716	-0.0603	-1.12717	-1.06477	-0.11667	0.493372	5.93	3.06	29.46	41.51	-0.30	0.65
Q6IB54	ATP synthase-coupling factor 6, mitochondrial	ATP5J	3.33	-0.46192	-0.65786	-1.04989	-1.1	-0.16942	-0.0297	9.59	2.46	6.84	33.07	-0.58	0.44	-0.74811	-0.248	-1.81522	-1.16306	-0.77935	-0.8329	24.27	31.43	2.62	32.16	-0.93	0.52
Q53Y06	ATPase, H+ transporting, lysosomal 31kDa, V1 subunit E isoform 1	ATP6V1E1	4.67	0.263723	-0.41024	0.174306	0.208642	-1.04292	-1.17722	32.44	1.68	6.58	41.08	-0.33	0.65	-0.12394	-0.42395	-0.11177	0.296213	-0.77156	-0.40788	14.65	19.86	17.73	23.84	-0.26	0.36
P35613	Basigin	BSG	8.67	-2.19735	NA	-0.83417	-0.69432	NA	NA	NA	6.85	NA	65.05	-1.24	0.83	-1.26479	NA	-0.5033	-0.66846	NA	NA	NA	8.09	NA	32.78	-0.81	0.40
A0A087WX97	Bcl-2-like protein 13	BCL2L13	4.00	NA	0.483935	0.433342	NA	NA	NA	NA	NA	NA	2.48	0.46	0.04	NA	0.464371	0.544328	NA	NA	NA	NA	NA	NA	3.92	0.50	0.06
X6R8A1	Carboxypeptidase	CTSA	1.00	0.905223	1.023722	0.215979	0.301623	0.178281	1.437632	5.80	4.20	58.08	25.28	0.68	0.52	0.543318	1.066638	-0.47243	0.081915	1.302066	2.354298	25.37	26.84	49.40	69.07	0.81	0.99
P23786	Carnitine O-palmitoyltransferase 2, mitochondrial	CPT2	5.33	0.223994	NA	-1.2984	-1.48577	NA	NA	NA	9.17	NA	71.74	-0.85	0.94	0.174009	NA	-0.99067	-1.56965	NA	NA	NA	28.00	NA	64.68	-0.80	0.89
P04040	Catalase	CAT	8.83	0.236079	-0.16365	-0.33404	-0.3794	0.07961	0.38357	19.47	2.22	14.84	20.25	-0.03	0.31	0.311725	-0.01573	0.566712	0.251973	0.487228	0.747865	15.98	15.37	12.74	15.99	0.39	0.27
P21964	Catechol O-methyltransferase	COMT	4.17	-0.00296	-0.03516	0.291553	0.388972	0.124156	-0.58928	1.58	4.77	34.27	19.24	0.03	0.34	0.54076	0.341408	0.393751	0.321676	-0.43663	-0.6281	9.76	3.53	9.37	32.82	0.09	0.49
A0A024R374	Cathepsin B, isoform CRA_a	CTSB	6.83	0.799262	0.809523	-0.02109	-0.11249	0.602953	0.848202	0.50	4.48	11.99	29.85	0.49	0.44	0.322979	0.275759	-0.05047	-0.16561	0.351199	1.010665	2.31	5.64	31.77	28.37	0.29	0.41
Q8N5K1	CDGSH iron-sulfur domain-containing protein 2	CISD2	1.40	-0.39232	-0.23737	-0.72588	-0.34685	-0.39053	NA	7.59	18.47	NA	7.33	-0.42	0.18	-0.45563	0	-0.60933	-0.72574	-0.6433	NA	22.15	5.70	NA	18.63	-0.49	0.29
B4DX69	cDNA FLJ55158, highly similar to Thioredoxin, mitochondrial	NA	2.33	-0.03709	-0.47261	NA	-0.96214	NA	NA	21.19	NA	NA	34.74	-0.49	0.46	0.43727	-0.40399	NA	-1.27281	NA	NA	40.10	NA	NA	61.72	-0.41	0.86
A8K651	cDNA FLJ75700, highly similar to Homo sapiens complement component 1, q subcomponent binding protei	NA	1.67	0.3641	-0.18831	-0.1127	-0.15383	0.148575	0.522874	26.75	2.02	18.24	16.57	0.10	0.30	1.205706	0.515983	0.08572	0.308695	0.365072	0.316587	33.18	10.91	2.38	26.99	0.47	0.39
B7ZB41	cDNA, FLJ79405, highly similar to Homo sapiens solute carrier family 25, member 24, transcript variant 1, m	NA	3.50	-0.3205	-0.76637	-0.24277	-0.2842	NA	NA	21.68	2.03	NA	12.85	-0.40	0.24	0.23001	-1.14572	-0.42783	-0.4881	NA	NA	62.74	2.95	NA	7.73	-0.46	0.56
Q08ET0	Cell proliferation-inducing protein 47	hCG_39985	4.50	-0.42445	-0.02837	-0.53175	-0.59421	-0.64913	-0.85363	19.29	3.06	10.01	19.25	-0.51	0.28	-0.32034	-0.0459	-0.44702	-0.74211	-0.41856	-0.26674	13.41	14.41	7.43	14.08	-0.37	0.23
Q5SRT3	Chloride intracellular channel protein	CLIC1	2.33	0.201574	0.197998	0.170543	0.032458	0.856059	-0.08125	0.18	6.76	44.39	13.18	0.23	0.33	-0.03302	0.18078	-0.03743	0.131596	0.593553	0.303289	10.46	8.27	14.18	16.35	0.19	0.24
A0A024QZN7	Chromosome 10 open reading frame 70, isoform CRA_b	C10orf70	2.50	-0.77904	-1.36284	-0.88467	-1.30034	-0.94091	-0.23909	28.23	20.23	33.74	21.55	-0.92	0.41	-0.40688	-1.25877	-1.05283	-1.313	-0.83802	-0.413	40.58	12.72	20.68	19.36	-0.88	0.40
A0A024RB75	Citrate synthase	CS	14.00	-0.54852	-0.86872	-1.73599	-1.76872	-0.66944	-1.21839	15.63	1.60	26.59	34.31	-1.13	0.53	-0.57003	-0.90101	-1.75334	-1.73873	-0.8846	-1.21141	16.15	0.72	15.95	33.38	-1.18	0.49
A0A087WVQ6	Clathrin heavy chain	CLTC	44.33	0.064858	-0.04277	0.13719	0.176111	-0.38431	-0.17786	5.27	1.91	10.10	14.91	-0.04	0.21	0.015474	0.037198	0.098067	-0.01854	-0.2174	-0.06268	1.06	5.71	7.58	6.75	-0.02	0.11
P10909	Clusterin	CLU	3.00	0.022462	-0.04884	0.232993	0.836901	-0.18857	NA	3.49	29.17	NA	28.71	0.17	0.40	0.253361	-0.55314	0.111013	0.807679	-0.01079	NA	38.53	33.50	NA	23.47	0.12	0.49
P12532	Creatine kinase U-type, mitochondrial	CKMT1A	14.00	-1.43373	-0.96512	-3.03515	-2.8866	-1.04378	-1.70639	22.77	7.27	31.92	52.47	-1.85	0.91	-1.41972	-0.94276	-2.85861	-2.4881	-1.38739	-1.9238	23.17	18.06	25.99	46.86	-1.84	0.73
V9HW27	Crystallin, alpha B, isoform CRA_a	CRYAB	7.50	0.659075	0.395929	0.143683	0.24313	1.218964	1.084734	12.86	4.87	6.57	34.71	0.62	0.45	0.885226	0.716504	0.451519	0.689039	1.140096	1.155232	8.26	11.62	0.74	20.26	0.84	0.28
P31930	Cytochrome b-c1 complex subunit 1, mitochondrial	UQCRC1	15.33	-0.33012	-0.91594	-1.29384	-1.40256	-0.92862	-0.61308	28.32	5.33	15.40	25.43	-0.91	0.40	-0.47682	-0.94106	-1.3052	-1.29369	-0.89687	-0.29904	22.56	0.56	28.89	25.02	-0.87	0.41
P22695	Cytochrome b-c1 complex subunit 2, mitochondrial	UQCRC2	13.50	-0.17695	-0.43929	-0.79318	-0.9709	-0.62414	-0.82556	12.82	8.70	9.86	21.39	-0.64	0.29	-0.72077	-0.62984	-0.86321	-0.84759	-1.39607	-0.45201	4.46	0.77	44.69	7.23	-0.82	0.32
P07919	Cytochrome b-c1 complex subunit 6, mitochondrial	UQCRH	2.33	-0.66822	-1.22379	-1.26872	-1.68158	-0.0779	-0.8711	26.90	20.10	37.93	35.22	-0.97	0.56	-1.07742	-0.93389	-1.01552	-1.43187	-0.04239	-0.70454	7.03	20.27	31.90	33.33	-0.87	0.47
P14927	Cytochrome b-c1 complex subunit 7	UQCRB	3.75	-0.10046	-0.23715	-1.11902	-1.37564	NA	NA	6.69	12.54	NA	50.36	-0.71	0.63	-0.30917	-0.31057	-0.67206	-1.38696	NA	NA	0.07	34.34	NA	32.53	-0.67	0.51
P47985	Cytochrome b-c1 complex subunit Rieske, mitochondrial	UQCRFS1	5.00	-0.76995	-0.61653	-1.01519	-1.12796	-0.37976	-0.947	7.51	5.52	27.45	15.61	-0.81	0.28	-0.8883	-0.52148	-1.27917	-1.32496	0.105053	-0.40561	17.88	2.24	24.77	39.60	-0.72	0.55
G4XXL9	Cytochrome c	CYCS	9.50	-1.25961	-1.82839	-2.06256	-2.21219	-0.9856	-1.27986	27.52	7.33	14.37	33.45	-1.60	0.50	-1.3696	-1.69007	-2.04341	-2.43938	-1.25614	-1.35244	15.64	19.29	4.72	30.54	-1.69	0.47
Q0Z7D8	Cytochrome c oxidase subunit 2	COX2	3.17	-0.85998	-1.00952	-2.0748	-2.397	-1.37431	-1.07483	7.32	15.73	14.63	40.88	-1.47	0.63	-1.2743	-1.16635	-2.29958	-2.48386	-1.41524	-1.28306	5.29	9.02	6.47	38.03	-1.65	0.58
P13073	Cytochrome c oxidase subunit 4 isoform 1, mitochondrial	COX4I1	3.83	-0.70497	-1.24883	-1.28477	-1.50434	-1.08174	-0.73786	26.34	10.74	16.78	17.63	-1.09	0.32	-0.81284	-1.18878	-1.23383	-1.16816	-0.71854	-0.57132	18.32	3.22	7.21	19.84	-0.95	0.28
P20674	Cytochrome c oxidase subunit 5A, mitochondrial	COX5A	5.50	0.534416	-0.39194	-1.09221	-1.71408	-1.32795	-1.2426	43.91	30.02	4.18	64.35	-0.87	0.81	0.918066	-0.21744	-0.53669	-1.84896	-1.3977	-0.89047	52.95	60.22	24.61	67.52	-0.66	0.97
P10606	Cytochrome c oxidase subunit 5B, mitochondrial	COX5B	5.00	-0.76492	-0.42163	-1.31545	-1.22569	-0.37431	-0.32111	16.75	4.40	2.61	30.45	-0.74	0.44	-0.81708	-0.40137	-1.24758	-0.97771	-0.5761	-0.4915	20.24	13.19	4.15	20.36	-0.75	0.32
P14854	Cytochrome c oxidase subunit 6B1	COX6B1	3.67	-0.46899	-0.75287	-0.77401	-0.95957	-0.60445	-0.66748	13.87	9.08	3.09	9.55	-0.70	0.17	-0.70675	-0.57628	-1.06814	-1.2125	-0.21021	-0.2875	6.39	7.07	3.79	30.01	-0.68	0.41
D6RIE3	Cytochrome c oxidase subunit 7A2, mitochondrial	COX7A2	2.60	-0.62569	-0.7987	-1.89252	-1.52544	-0.11103	NA	8.47	17.90	NA	50.19	-0.99	0.71	-0.83652	-0.98487	-1.70221	-1.50519	-0.91313	NA	7.27	9.64	NA	25.11	-1.19	0.39
A0A024R9B7	Cytochrome c oxidase subunit VIc, isoform CRA_a	COX6C	2.00	-0.53848	-1.00114	-1.43505	-1.27971	-0.59197	-0.64168	22.48	7.61	2.44	25.15	-0.91	0.38	-0.91285	-1.10061	-1.0455	-1.66646	-0.69961	-1.17325	9.19	29.97	23.01	14.25	-1.10	0.32
Q13011	Delta(3,5)-Delta(2,4)-dienoyl-CoA isomerase, mitochondrial	ECH1	7.67	0.900696	0.954098	0.323253	0.249797	0.304645	1.382594	2.62	3.60	50.51	23.76	0.69	0.46	1.001861	1.141964	-0.0812	-0.30987	0.321722	1.126298	6.86	11.18	38.44	40.11	0.53	0.64
Q53HB7	Diablo isoform 1 variant (Fragment)	NA	2.33	0.933739	0.3807	NA	NA	0.690789	NA	26.78	NA	NA	0.35	0.67	0.28	1.015474	0.853788	NA	NA	0.596291	NA	7.92	NA	NA	16.62	0.82	0.21
A0A024R713	Dihydrolipoyl dehydrogenase	DLD	10.67	-0.49566	-0.34537	-1.13931	-1.07091	0.228302	0.216161	7.36	3.35	0.60	44.49	-0.43	0.60	-0.41387	-0.34626	-1.44151	-0.90267	-0.04729	-0.19539	3.31	26.11	7.25	33.54	-0.56	0.52
P09417	Dihydropteridine reductase	QDPR	4.00	-0.10068	-0.28538	-0.46511	0.14068	-0.17383	0.147772	9.04	29.26	15.70	6.65	-0.12	0.24	-0.2836	-0.04226	-0.31209	-0.07271	-0.39064	0.13178	11.80	11.71	25.33	2.87	-0.16	0.20
B1AKK2	Dimethylarginine dimethylaminohydrolase 1, isoform CRA_b	DDAH1	11.17	-0.61721	-1.26061	-0.33604	-0.21984	-0.56091	-1.35091	31.02	5.69	37.78	26.56	-0.72	0.47	-0.4863	-1.05732	-0.39086	0.288175	-0.46176	-1.41443	27.63	32.68	45.07	33.99	-0.59	0.59
P31689	DnaJ homolog subfamily A member 1	DNAJA1	1.00	-1.30558	NA	-0.60203	NA	NA	NA	NA	NA	NA	33.82	-0.95	0.50	-0.70497	NA	0.019854	NA	NA	NA	NA	NA	NA	34.80	-0.34	0.51
Q9H3Z4	DnaJ homolog subfamily C member 5	DNAJC5	1.50	NA	-0.84876	-0.51196	NA	NA	NA	NA	NA	NA	16.43	-0.68	0.24	NA	-1.06237	-0.48548	NA	NA	NA	NA	NA	NA	27.90	-0.77	0.41
Q96FJ2	Dynein light chain 2, cytoplasmic	DYNLL2	3.00	-0.73677	-1.00533	-0.56391	-0.51636	-0.11557	-0.19216	13.12	2.33	3.75	24.76	-0.52	0.33	-0.31134	-0.8327	-0.86736	-0.48139	-0.55377	-0.48609	25.28	18.81	3.32	5.13	-0.59	0.22
P13804	Electron transfer flavoprotein subunit alpha, mitochondrial	ETFA	9.67	0.373704	0.211832	-0.16137	-0.45617	-0.5821	-0.37087	7.93	14.40	10.33	29.35	-0.16	0.38	0.517681	0.0496	-0.45741	-0.69151	-0.41029	-0.15836	22.74	11.45	12.32	31.81	-0.19	0.43
P38117	Electron transfer flavoprotein subunit beta	ETFB	5.67	0.516914	0.345426	-0.12507	-0.07257	0.478451	0.462513	8.40	2.57	0.78	20.54	0.27	0.29	0.457997	0.391824	-0.18267	0.117243	-0.1736	0.37768	3.24	14.65	26.70	16.19	0.16	0.29
P49411	Elongation factor Tu, mitochondrial	TUFM	7.00	1.405706	1.057313	0.176419	0.610099	0.555518	-0.2749	16.99	21.10	39.61	40.71	0.59	0.60	1.385121	0.886459	0.477379	0.577795	0.31874	-0.22011	24.20	4.92	26.11	38.08	0.57	0.54
P42126	Enoyl-CoA delta isomerase 1, mitochondrial	ECI1	4.00	-0.04763	-0.34152	-1.34814	-1.21647	-0.25241	-0.84092	14.35	6.45	28.45	35.52	-0.67	0.54	-0.09697	-0.37369	-1.47418	-1.29431	0.00619	-0.80532	13.52	8.80	38.76	37.68	-0.67	0.62

UniProt	Protein name	Gene name	Peptide mean	08/017 hESC-RPE to hRPE: Analysis 1.1	1.2	2.1	2.2	3.1	3.2	Technical replicate CV 1	2	3	Biological CV	Fold change (mean)	StDev	08/023 hESC-RPE to hRPE: Analysis 1.1	1.2	2.1	2.2	3.1	3.2	Technical replicate CV 1	2	3	Biological CV	Fold change (mean)	StDev
P30084	Enoyl-CoA hydratase, mitochondrial	ECHS1	7.67	0.39047	0.325417	-0.67336	-0.51787	-0.08545	-0.6211	3.19	7.61	25.96	35.66	-0.20	0.48	0.401885	0.365041	-0.80104	-0.46076	0.326182	0.698982	1.81	16.60	18.17	37.29	0.09	0.58
V9HW98	Epididymis luminal protein 2	YWHAE	18.67	-0.21164	-0.2477	0.358618	0.147836	-0.43028	-0.534	1.77	10.31	5.08	26.76	-0.15	0.34	-0.29109	-0.3092	0.007824	-0.21016	-0.10372	-0.24045	0.89	10.66	6.70	7.06	-0.19	0.12
D0PNI1	Epididymis luminal protein 4	YWHAZ	14.33	-0.63117	-0.90274	-0.3634	-0.27523	-1.43576	-1.52421	13.27	4.32	4.339	37.98	-0.86	0.53	-0.55579	-0.558	-0.45186	-0.34919	-0.69928	-0.80832	0.11	5.03	5.34	12.16	-0.57	0.17
V9HW90	Epididymis luminal protein 75	GSR	2.50	-0.17518	1.027452	0.033275	0.506461	0.810876	0.353466	55.75	22.99	22.23	11.18	0.43	0.46	-0.41738	0.679971	-0.25844	0.505866	0.648275	-0.51364	51.33	36.61	54.06	2.26	0.11	0.56
V9HWH2	Epididymis secretory protein Li 29	CKB	50.00	-2.16256	-2.32478	-2.1682	-2.70202	-3.12377	-3	7.94	25.87	6.06	27.49	-2.58	0.42	-2.373	-2.45987	-2.32092	-2.51815	-2.55043	-2.50876	4.26	9.65	2.04	4.48	-2.46	0.09
V9HWC2	Epididymis secretory sperm binding protein Li 67p	PARK7	12.50	-0.59355	-0.33432	-0.20867	-0.32721	-0.35666	-0.1034	12.67	5.81	12.38	8.43	-0.32	0.16	-0.57068	-0.3232	-0.31713	-0.0595	-0.68192	-0.56118	12.10	12.59	5.91	15.42	-0.42	0.23
V9HWH7	Epididymis secretory sperm binding protein Li 70p	ATIC	4.40	NA	2.708129	1.393037	1.133248	0.29977	0.39178	NA	12.70	4.51	81.32	1.19	0.97	NA	2.07853	1.146196	0.932189	0.276515	0.656556	NA	10.47	18.52	57.80	1.02	0.68
V9HWB4	Epididymis secretory sperm binding protein Li 89n	HSPA5	36.67	0.922878	0.761957	0.818482	1.064198	0.972125	0.975126	7.88	12.01	0.15	4.66	0.92	0.11	0.871039	0.806064	0.606646	0.732214	0.806912	0.783625	3.18	6.15	1.14	5.97	0.77	0.09
E9KL44	Epididymis tissue sperm binding protein Li 14m	NA	27.67	0.479001	0.616135	0.230736	0.164397	-0.00399	0.051095	6.72	3.25	2.70	19.00	0.26	0.24	0.45672	0.570864	-0.2205	-0.21104	0.474634	0.333847	5.59	0.46	6.89	25.09	0.23	0.36
Q92506	Estradiol 17-beta-dehydrogenase 8	HSD17B8	1.50	NA	0.665441	-0.16137	-0.12537	0.510696	NA	NA	1.76	NA	27.24	0.22	0.43	NA	-1.40925	0.202701	0.398732	-0.00083	NA	NA	9.59	NA	50.96	-0.20	0.82
A0A024R9P6	Family with sequence similarity 82, member C, isoform CRA_a	FAM82C	2.00	-0.77533	NA	0.12776	-0.22261	NA	NA	NA	17.09	NA	35.43	-0.29	0.46	-0.47773	NA	-0.19565	-0.56218	NA	NA	NA	17.87	NA	5.41	-0.41	0.19
P49327	Fatty acid synthase	FASN	2.50	-0.09079	NA	1.258588	0.003559	1.696925	NA	NA	57.91	NA	59.90	0.72	0.90	-0.16315	NA	1.402871	-0.68907	1.022059	NA	NA	87.68	NA	38.01	0.39	0.98
A0A024QZ64	Fructose-bisphosphate aldolase	ALDOC	29.40	-1.13636	-1.60061	NA	-1.49243	-2.01299	-1.71889	22.56	NA	14.36	17.46	-1.59	0.32	-1.46437	-1.82175	NA	-1.46563	-1.74741	-1.70001	17.43	NA	2.32	9.17	-1.64	0.17
P07954	Fumarate hydratase, mitochondrial	FH	8.33	-0.45165	-0.2338	-1.80563	-0.95497	-0.94521	-0.43879	10.66	40.53	24.57	32.27	-0.81	0.57	-0.25247	-0.15232	-1.66242	-1.00126	-1.10661	-0.8117	4.91	31.85	14.40	40.36	-0.83	0.56
P17900	Ganglioside GM2 activator	GM2A	1.80	0.784138	1.00946	1.028086	0.26633	0.927401	NA	11.02	36.49	NA	8.53	0.80	0.32	0.406521	0.380697	-0.01526	0.081438	0.495612	NA	1.27	4.74	NA	16.09	0.27	0.22
A8YXX4	Glutamine synthetase	PIG59	10.83	1.116885	-0.20791	-1.24968	-1.52848	-1.29362	-1.9128	60.72	13.62	29.89	89.62	-0.85	1.12	1.009174	-0.13552	-0.79225	-1.19969	-0.90343	-1.73095	53.34	19.84	39.48	72.76	-0.63	0.96
Q7Z4Y4	GTP:AMP phosphotransferase AK3, mitochondrial	AK3	2.33	0.592097	NA	0.898295	-0.13518	NA	NA	NA	48.59	NA	5.87	0.45	0.53	0.951083	NA	0.865214	0.372462	NA	NA	NA	23.92	NA	15.20	0.73	0.31
A0A024R3X4	Heat shock 60kDa protein 1 (Chaperonin), isoform CRA_a	HSPD1	41.17	-0.24718	-0.40671	-0.80101	-0.93618	0.498082	1.452736	7.81	6.62	45.15	71.79	-0.07	0.90	-0.05676	-0.34151	-0.94367	-0.84987	0.252589	0.858863	13.91	4.60	29.29	50.40	-0.18	0.68
Q6IBN1	HNRPK protein	HNRPK	10.33	-0.37843	-0.33839	0.423494	0.291965	-0.75367	-0.45226	1.96	6.44	14.72	36.29	-0.20	0.46	0.033998	0.075762	0.303335	0.450434	-0.71956	-0.33451	2.05	7.20	18.76	29.70	-0.03	0.43
P35914	Hydroxymethylglutaryl-CoA lyase, mitochondrial	HMGCL	1.00	NA	-0.29117	-1.43792	NA	-0.37612	0.152612	NA	NA	25.63	42.43	-0.49	0.67	NA	0.358009	-0.34772	NA	-0.09155	0.577127	NA	NA	32.20	24.60	0.12	0.42
P54868	Hydroxymethylglutaryl-CoA synthase, mitochondrial	HMGCS2	9.33	1.134297	0.95498	1.156106	1.2448	1.098142	1.145154	8.78	4.35	2.30	5.33	1.12	0.10	0.57205	0.602847	0.340584	0.736461	0.205486	0.749139	1.51	19.28	26.34	2.94	0.53	0.22
Q6YN16	Hydroxysteroid dehydrogenase-like protein 2	HSDL2	4.50	1.10183	NA	0.194599	0.003559	0.702004	NA	NA	9.35	NA	53.21	0.50	0.50	1.422714	NA	0.277471	0.175829	1.636285	NA	NA	4.98	NA	43.88	0.88	0.76
Q9H2U2	Inorganic pyrophosphatase 2, mitochondrial	PPA2	5.40	-0.1151	-0.04806	1.03638	0.205216	NA	-0.06017	3.29	39.65	NA	32.10	0.20	0.48	0.622452	-0.18026	-0.50977	-0.20056	NA	0.164431	38.36	15.10	NA	21.53	-0.02	0.43
Q53GF8	Isocitrate dehydrogenase [NAD] subunit, mitochondrial (Fragment)	NA	7.00	-0.2201	-0.4629	-0.62228	-0.69389	NA	-0.77221	11.87	3.51	NA	16.11	-0.55	0.22	-0.39214	-0.51171	-0.79884	-0.88765	NA	-0.59862	5.86	4.35	NA	13.46	-0.64	0.20
V9HWJ2	Isocitrate dehydrogenase [NADP]	IDH1	6.67	-0.43532	1.412354	0.441368	-0.07535	0.250916	0.312855	79.92	25.06	3.04	22.07	0.32	0.62	-0.01431	1.536486	0.827637	0.080245	-0.01183	1.108833	69.45	35.83	52.32	16.52	0.59	0.66
Q9NSE4	Isoleucine--tRNA ligase, mitochondrial	IARS2	3.00	0.989033	NA	0.644588	NA	NA	NA	NA	NA	NA	16.80	0.82	0.24	1.10346	NA	0.773891	NA	NA	NA	NA	NA	NA	16.08	0.94	0.23
A0A0A0MT83	Isovaleryl-CoA dehydrogenase, mitochondrial	IVD	2.17	-0.07764	-0.21803	-0.09136	-0.58193	0.283439	0.556993	6.88	23.82	13.37	28.28	-0.02	0.40	0.376791	0.262226	0.387767	-0.33925	-0.32027	0.529716	5.61	34.90	40.50	8.85	0.15	0.38
Q14894	Ketimine reductase mu-crystallin	CRYM	11.67	-0.86463	-1.37756	-1.5491	-1.54607	-1.75674	-1.36213	24.88	0.15	19.22	18.76	-1.41	0.30	-0.75734	-1.10344	-1.65602	-1.83448	-2.00435	-1.4042	16.88	8.74	29.00	33.80	-1.46	0.47
E5KNY5	Leucine-rich PPR-motif containing	LRPPRC	9.40	-1.00335	0.751622	-0.19739	-0.94123	0.161381	NA	76.77	35.67	NA	24.38	-0.25	0.75	-1.46377	0.539824	0.025833	-1.14398	0.260379	NA	84.97	54.39	NA	24.68	-0.36	0.89
Q5U077	L-lactate dehydrogenase	LDHB	19.00	-0.29468	-0.17775	0.043599	0.141397	0.112825	0.187159	5.73	4.79	3.64	13.83	0.00	0.19	-0.29109	-0.12395	0.295728	0.198218	-0.15023	-0.14693	8.18	4.78	0.16	17.81	-0.04	0.23
Q6IAQ1	LYPLA1 protein	LYPLA1	4.20	0.41548	-0.07981	-0.55104	-0.18316	NA	0.279593	24.04	17.93	NA	22.12	-0.02	0.38	-0.12752	0.280121	-0.54885	-0.696	NA	0.294762	19.85	7.21	NA	30.31	-0.16	0.46
Q16891	MIC	IMMT	13.83	-0.26174	-0.10817	-0.70412	-0.48798	0.325545	0.606287	7.52	10.57	13.72	38.03	-0.11	0.49	-0.03302	-0.22155	-0.49543	-0.3313	0.524075	0.709101	9.23	8.04	9.06	38.69	0.03	0.49
Q5XKP0	MIC	MIC13	1.00	-1.29347	-1.20179	-1.3307	-2.05057	-1.06908	NA	4.49	34.57	NA	19.64	-1.39	0.38	-0.55193	-0.61322	-1.23383	-1.32812	-0.58569	NA	3.00	4.62	NA	25.34	-0.86	0.38
H7C1U8	MIC	APOO	1.60	-0.27121	-0.29933	-0.93142	-0.93921	NA	-0.27524	1.38	0.38	NA	23.99	-0.54	0.36	0.091479	-0.0357	-0.8911	-0.52576	NA	-0.19367	6.23	17.81	NA	24.41	-0.31	0.40
C9JRZ6	MIC	CHCHD3	1.50	0.586602	0.663644	0.043599	-0.49837	NA	NA	3.78	26.26	NA	39.49	0.20	0.54	0.341256	0.546931	-0.64371	0.253667	NA	NA	10.06	42.62	NA	27.77	0.12	0.53
Q9Y3D6	Mitochondrial fission 1 protein	FIS1	2.00	NA	-0.02373	-0.63293	-1.90425	NA	NA	NA	58.57	NA	51.83	-0.85	0.96	NA	0.099609	-2.42443	-1.48457	NA	NA	NA	44.50	NA	84.19	-1.27	1.28
Q9H936	Mitochondrial glutamate carrier 1	SLC25A22	3.00	NA	-1.9095	-0.31446	-0.55883	NA	NA	NA	11.95	NA	66.71	-0.93	0.86	NA	-1.69078	-1.14105	-0.60453	NA	NA	NA	26.00	NA	40.17	-1.15	0.54
O94826	Mitochondrial import receptor subunit TOM70	TOMM70A	10.00	0.038842	0.069666	-1.39129	NA	NA	NA	1.51	NA	NA	65.47	-0.43	0.83	0.093945	-0.18126	-1.22315	NA	NA	NA	13.45	NA	NA	55.06	-0.44	0.69
E5KRK5	Mitochondrial NADH-ubiquinone oxidoreductase 75 kDa subunit	NDUFS1	18.00	-0.32001	-0.26905	-1.13581	-1.11648	-0.76884	-0.04872	2.50	0.95	34.58	28.69	-0.61	0.46	-0.51733	-0.37141	-1.05704	-1.29431	-0.87851	-0.00977	7.15	11.60	41.34	27.50	-0.69	0.48
Q9NUJ1	Mycophenolic acid acyl-glucuronide esterase, mitochondrial	ABHD10	3.75	NA	-0.35943	-1.28736	-1.40745	-0.575	NA	NA	5.88	NA	32.40	-0.91	0.52	NA	-0.84809	-1.19044	-1.48245	0.020124	NA	NA	14.26	NA	48.80	-0.88	0.65
P35580	Myosin-10	MYH10	10.17	1.357816	1.470366	0.552529	0.574781	0.442283	0.37564	5.51	1.09	3.27	40.21	0.80	0.49	1.376116	1.211269	0.285798	1.163715	0.740394	0.386223	8.07	41.75	17.27	26.61	0.86	0.46
P23368	NAD-dependent malic enzyme, mitochondrial	ME2	3.67	NA	0.572379	-0.3583	-0.72536	NA	NA	NA	17.89	NA	51.55	-0.17	0.67	NA	0.961365	-0.16043	-0.54293	NA	NA	NA	18.64	NA	59.75	0.09	0.78
A8K761	NADH dehydrogenase (Ubiquinone) 1 beta subcomplex, 10, 22kDa, isoform CRA_b	NDUFB10	2.80	-0.58025	0.137849	-0.62473	-0.30034	0.055062	NA	34.49	15.83	NA	17.44	-0.26	0.35	-0.77711	-0.05754	-0.76361	-0.68785	0.280777	NA	34.55	3.71	NA	36.48	-0.40	0.48
Q6IBA0	NADH dehydrogenase (Ubiquinone) Fe-S protein 5, 15kDa (NADH-coenzyme Q reductase)	NDUFS5	2.00	-0.32814	-0.06881	NA	-1.77052	NA	NA	12.68	NA	NA	70.45	-0.72	0.92	-1.43979	-0.22824	NA	-1.18413	NA	NA	56.12	NA	NA	23.01	-0.95	0.64
Q9P0J0	NADH dehydrogenase [ubiquinone] 1 alpha subcomplex subunit 13	NDUFA13	2.80	0.024785	-0.84978	-0.87783	-1.22692	-0.70159	NA	41.60	17.03	NA	23.96	-0.73	0.46	0.563624	-0.6626	-0.88081	-1.60837	-1.17921	NA	56.73	34.92	NA	55.25	-0.75	0.82
O43678	NADH dehydrogenase [ubiquinone] 1 alpha subcomplex subunit 2	NDUFA2	2.80	-0.68142	-0.75961	-2.00611	-1.16709	-0.09288	NA	3.83	40.00	NA	46.93	-0.94	0.71	-0.8782	-0.99714	-1.3724	-1.26006	-1.48097	NA	5.83	5.50	NA	29.02	-1.00	0.35
H7BYD0	NADH dehydrogenase [ubiquinone] 1 alpha subcomplex subunit 5 (Fragment)	NDUFA5	6.40	-0.63178	-0.9188	-1.0598	-0.95446	-0.45632	NA	14.02	5.16	NA	19.26	-0.80	0.25	-0.70818	-1.07299	-1.09114	-0.68095	-0.45409	NA	17.79	19.97	NA	17.59	-0.80	0.27
P51970	NADH dehydrogenase [ubiquinone] 1 alpha subcomplex subunit 8	NDUFA8	4.50	-0.89835	-0.47235	-1.42008	-1.38043	NA	NA	20.73	1.94	NA	35.06	-1.04	0.45	-0.86059	-0.83466	-1.31713	-1.20666	NA	NA	1.27	5.41	NA	20.12	-1.05	0.24
C9JKQ2	NADH dehydrogenase [ubiquinone] 1 beta subcomplex subunit 3 (Fragment)	NDUFB3	1.00	NA	-0.86976	-0.78448	-0.52392	NA	NA	NA	12.74	NA	10.83	-0.73	0.18	NA	-1.15404	-0.71919	-0.62112	NA	NA	NA	4.80	NA	23.54	-0.83	0.28
P17568	NADH dehydrogenase [ubiquinone] 1 beta subcomplex subunit 7	NDUFB7	1.00	-0.64557	-1.27938	-1.7011	-0.34384	-1.51864	NA	30.57	62.02	NA	22.97	-1.10	0.58	-1.1854	-0.52022	-1.49045	-0.34662	-2.55529	NA	32.04	53.30	NA	52.86	-1.22	0.88
O95169	NADH dehydrogenase [ubiquinone] 1 beta subcomplex subunit 8, mitochondrial	NDUFB8	2.50	-0.34405	-0.64326	-0.88564	-0.82552	NA	NA	14.61	2.95	NA	18.01	-0.67	0.24	-0.52835	-0.33732	-0.86597	-0.65768	NA	NA	9.35	10.19	NA	16.03	-0.60	0.22
E7EWZ0	NADH dehydrogenase [ubiquinone] 1 beta subcomplex subunit 9	NDUFB9	2.50	-0.31952	-0.7376	-1.34679	-0.97452	NA	NA	20.35	18.15	NA	30.64	-0.84	0.43	-0.65305	-0.61221	-1.51122	-1.29803	NA	NA	2.00	10.43	NA	36.79	-1.02	0.45
A0A024R5K6	NADH dehydrogenase [ubiquinone] 1 subunit C2	NDUFC2	2.00	-0.56502	-0.73254	-0.90934	-0.09746	NA	NA	8.20	38.77	NA	9.75	-0.58	0.35	-0.66481	-0.53899	-0.99874	-0.56628	NA	NA	6.16	21.04	NA	8.12	-0.69	0.21
P19404	NADH dehydrogenase [ubiquinone] flavoprotein 2, mitochondrial	NDUFV2	3.00	-1.15553	-0.61043	-1.51007	-1.98162	-1.58294	-3.0378	26.40	25.24	65.82	40.64	-1.48	0.84	-1.08623	-0.77311	-1.53522	-1.54146	-1.14223	-2.41215	15.29	0.31	58.51	28.57	-1.42	0.57
O43181	NADH dehydrogenase [ubiquinone] iron-sulfur protein 4, mitochondrial	NDUFS4	2.75	0.048772	0.893158	-1.67717	-1.17183	NA	NA	40.24	24.52	NA	82.77	-0.48	1.17	-0.30672	0.756576	-0.99369	-0.65251	NA	NA	49.88	16.64	NA	52.88	-0.30	0.76
Q9UHQ9	NADH-cytochrome b5 reductase 1	CYB5R1	4.20	-0.27454	-0.62881	-0.49805	-0.197	NA	-0.75875	17.28	14.70	NA	14.60	-0.47	0.24	-0.1019	-0.3697	0.004548	0.061264	NA	-1.10074	13.09	2.78	NA	36.55	-0.30	0.48
Q3MHU6	NDUFB1 protein (Fragment)	NDUFB1	1.00	NA	-0.94927	-1.80286	NA	NA	NA	NA	NA	NA	40.66	-1.38	0.60	NA	-1.09307	-0.53779	NA	NA	NA	NA	NA	NA	26.88	-0.82	0.39
Q2TB59	Nicotinamide nucleotide transhydrogenase	NNT	8.50	0.052573	0.191031	-0.60727	-0.0533	0.274221	-0.00893	6.78	26.82	13.83	16.48	-0.03	0.31	0.209122	0.132145	-0.75419	0.10345	0.247547	-0.0694	3.77	40.84	15.47	15.37	-0.02	0.38
P15531	Nucleoside diphosphate kinase A	NME1	7.00	-0.33409	-1.45872	-0.21266	-0.67651	NA	-1.90537	52.49	22.54	NA	45.73	-0.92	0.74	-0.22916	-1.713	-0.051	-0.08337	NA	-0.9675	66.93	1.59	NA	35.02	-0.61	0.72
A0A024R9U3	OCIA domain containing 1, isoform CRA_a	OCIAD1	2.00	0.016247	0.196176	0.210492	-0.02298	0.914703	1.340186	8.81	11.42	20.70	45.07	0.44	0.56	0.51385	0.930943	-0.03198	-0.07351	0.666628	0.991518	20.30	2.04	15.86	30.20	0.50	0.46
P04181	Ornithine aminotransferase, mitochondrial	OAT	3.33	0.034428	0.40025	0.550178	1.888438	0.980367	0.555263	17.83	61.26	20.69	38.97	0.73	0.64	-0.22046	0.084517	0.71868	1.12038	0.546192	0.098772	14.89	19.56	21.76	35.20	0.39	0.49
Q02790	Peptidyl-prolyl cis-trans isomerase FKBP4	FKBP4	1.50	-0.34955	NA	NA	0.147122	NA	NA	NA	NA	NA	24.11	-0.10	0.35	-0.64686	NA	NA	0.67662	NA	NA	NA	NA	NA	60.67	0.01	0.94
Q06830	Peroxiredoxin-1	PRDX1	17.50	0.03462	0.066588	0.110187	0.35907	0.106996	0.038215	1.57	12.17	3.37	7.31	0.12	0.12	0.179438	0.234586	0.125468	0.141009	-0.30487	-0.3915	2.70	0.76	4.24	19.58	0.00	0.27
Q08426	Peroxisomal bifunctional enzyme	EHHADH	3.25	0.037308	0.030243	-1.25095	NA	NA	-0.30287	0.35	NA	NA	40.73	-0.37	0.61	0.065958	-0.94785	-0.60163	NA	NA	-0.38394	47.74	NA	NA	9.12	-0.47	0.42
Q5VSQ6	Procollagen-proline, 2-oxoglutarate 4-dioxygenase (Proline 4-hydroxylase), alpha polypeptide I variant (Fra	P4HA1	4.83	1.679007	1.433592	1.521844	1.310897	0.563959	0.64105	12.00	10.32	3.78	31.91	1.19	0.47	1.616033	1.517908	1.253259	1.073906	0.753578	0.942787	4.81	8.78	9.26	25.06	1.19	0.33
A0A024QZQ2	Prosaposin (Variant Gaucher disease and variant metachromatic leukodystrophy), isoform CRA_b	PSAP	20.75	NA	-0.80997	NA	-0.47583	-0.46595	-0.76739	NA	NA	14.72	11.51	-0.63	0.18	NA	-0.78294	NA	-1.1279	-0.12431	-0.35929	NA	NA	11.49	31.76	-0.60	0.45
P49720	Proteasome subunit beta type-3	PSMB3	2.00	-0.67418	NA	-0.58205	-0.64319	NA	NA	NA	3.00	NA	3.03	-0.63	0.05	-0.51013	NA	-0.27273	-0.13647	NA	NA	NA	6.67	NA	15.00	-0.31	0.19
F8W7Q4	Protein FAM162A	FAM162A	1.50	NA	0.094236	-0.76723	NA	NA	NA	NA	NA	NA	41.01	-0.34	0.61	NA	-0.32623	-0.74314	NA	NA	NA	NA	NA	NA	20.29	-0.53	0.29
Q8WYJ5	Protein kinase C inhibitor-2	NA	2.60	-0.99078	-1.62207	-0.60646	-1.1017	NA	-0.82181	30.46	24.04	NA	16.81	-1.03	0.38	-0.93279	-2.43464	-0.54294	-1.14733	NA	-0.68823	67.61	29.20	NA	27.43	-1.15	0.75
A0A087X1F5	Protein SYNJ2BP-COX16 (Fragment)	SYNJ2BP-COX16	1.00	NA	0.26066	-0.5379	NA	NA	NA	NA	NA	NA	38.17	-0.14	0.56	NA	-0.19305	-0.80325	NA	NA	NA	NA	NA	NA	29.47	-0.50	0.43
P11177	Pyruvate dehydrogenase E1 component subunit beta, mitochondrial	PDHB	9.50	-0.17273	-0.02528	-1.17238	-0.63089	-0.84589	-1.00085	7.22	26.23	7.59	34.40	-0.64	0.46	-0.25273	0.014253	-0.7705	-0.72699	-1.08935	-0.66022	13.05	2.13	20.88	29.55	-0.58	0.40
Q15907	Ras-related protein Rab-11B	RAB11B	6.50	0.185912	0.111031	0.180167	0.198569	NA	NA	3.67	0.90	NA	1.98	0.17	0.04	0.155439	0.237206	0.011092	0.058116	NA	NA	4.01	2.30	NA	7.94	0.12	0.10
Q9H0U4	Ras-related protein Rab-1B	RAB1B	6.80	-1.39232	NA	-0.18197	-0.75087	-0.29225	0.036975	NA	27.53	16.07	40.41	-0.52	0.57	-0.34101	NA	-0.81923	-0.67732	-1.10929	-0.44209	NA	6.95	32.13	16.72	-0.68	0.31
Q9BRX8	Redox-regulatory protein FAM213A	FAM213A	5.00	-0.03064	-0.55835	-0.77447	-0.55767	-0.0704	0.015184	25.58	10.61	4.19	21.52	-0.33	0.34	0.010045	-0.48463	-0.82819	-0.74337	0.166958	-0.03741	24.01	4.16	10.00	28.40	-0.32	0.42
Q6IRT1	S-(hydroxymethyl)glutathione dehydrogenase	ADH5	5.67	0.391821	0.005495	0.008193	0.311853	0.489246	0.218593	18.82	14.83	13.23	7.09	0.24	0.20	0.773017	0.20965	-0.02193	0.194032	-0.05801	0.399431	27.27	10.57	22.23	16.04	0.25	0.31
Q8NBX0	Saccharopine dehydrogenase-like oxidoreductase	SCCPDH	6.00	-0.63789	-0.15877	-0.81773	-0.83631	-0.76955	-0.05429	23.27	0.91	34.36	17.12	-0.55	0.35	-0.55	-0.19431	-0.89674	-0.5356	-0.58135	-1.10903	17.35	17.61	25.58	17.09	-0.64	0.32
V9HW06	Serine hydroxymethyltransferase	SHMT2	1.33	NA	-1.79573	NA	0.26106	-0.7931	NA	NA	NA	NA	67.63	-0.78	1.03	NA	-2.40282	NA	0.276347	-0.73531	NA	NA	NA	NA	77.10	-0.95	1.35
B4DRS6	Sideroflexin	NA	5.67	-0.35382	-0.49251	-0.84176	-0.97297	-0.25058	-0.06874	6.79	6.43	8.90	25.15	-0.50	0.35	-0.35075	-0.65009	-0.45394	-0.59546	-0.47549	0.038553	14.62	6.98	24.93	12.95	-0.41	0.25
Q9H9B4	Sideroflexin-1	SFXN1	3.67	0.12476	-1.75993	-0.00717	0.032458	-0.03516	0.028963	81.15	1.94	3.14	19.94	-0.27	0.73	-0.07096	-1.82643	-0.59054	0.461838	0.004336	0.82482	76.79	49.41	39.16	38.21	-0.20	0.93
Q567R6	Single-stranded DNA-binding protein	SSBP1	2.83	1.087637	0.635815	-0.13756	0.16886	0.262266	0.39737	21.97	14.96	6.62	30.84	0.40	0.42	0.210069	0.460373	0.647284	-0.37089	0.728588	0.434548	12.24	47.93	14.36	13.11	0.35	0.40
Q6IBH0	SLC25A11 protein	SLC25A11	7.00	-0.08681	0.240073	-1.57493	-1.44787	-0.76029	-0.30182	15.95	6.22	22.28	50.41	-0.66	0.74	-0.13448	0.115155	-1.09762	-1.51887	-1.29063	-0.23339	12.21	20.50	49.62	43.85	-0.69	0.69
Q6NVC0	SLC25A5 protein (Fragment)	SLC25A5	14.67	-0.87542	-0.85974	-1.51385	-1.78408	-0.74779	-0.75756	0.77	13.21	0.48	30.05	-1.09	0.44	-1.02877	-1.14378	-2.04969	-2.15686	-1.08891	-1.02909	5.63	5.25	2.93	35.53	-1.42	0.54
Q6I9V5	SLC25A6 protein	SLC25A6	15.67	-1.1183	-0.92528	-2.14692	-2.18556	-1.92277	-1.8116	9.45	1.89	5.45	43.54	-1.69	0.54	-1.00967	-0.95766	-2.70386	-2.48386	-1.19664	-0.66395	2.55	10.76	25.82	50.92	-1.50	0.87
Q53GR7	Solute carrier family 25, member 13 (Citrin) variant (Fragment)	NA	9.83	-0.3453	-0.45325	-0.57928	-1.19639	0.030089	0.476496	5.29	29.79	21.71	39.80	-0.34	0.57	-0.55643	-0.43851	-0.55925	-1.0211	0.084724	0.582141	5.78	22.44	24.14	43.10	-0.32	0.57
Q9Y512	Sorting and assembly machinery component 50 homolog	SAMM50	3.40	-0.37307	-0.7987	-0.70758	-0.93972	0.330778	NA	20.71	11.35	NA	44.66	-0.50	0.51	0.090656	-0.61525	-0.74738	-1.01599	0.645105	NA	33.92	13.13	NA	52.74	-0.33	0.68
G3V2S9	SRA stem-loop-interacting RNA-binding protein, mitochondrial	SLIRP	1.50	0.727842	0.173464	0.701601	0.111207	NA	NA	26.84	28.54	NA	2.00	0.43	0.33	-0.02319	0.559994	1.101727	0.644209	NA	NA	28.20	22.24	NA	28.68	0.57	0.46
Q02318	Sterol 26-hydroxylase, mitochondrial	CYP27A1	1.00	NA	NA	NA	0.934339	1.05775	2.142116	NA	NA	50.78	36.65	1.38	0.66	NA	NA	NA	0.308695	0.557055	1.688259	NA	NA	52.77	43.72	0.85	0.74
Q9UJZ1	Stomatin-like protein 2, mitochondrial	STOML2	5.33	NA	NA	-0.22098	-0.06318	0.549418	NA	NA	7.73	NA	33.16	0.09	0.41	NA	NA	-0.37502	-0.83764	0.726465	NA	NA	22.48	NA	60.30	-0.16	0.80
A0A024QZ30	Succinate dehydrogenase [ubiquinone] flavoprotein subunit, mitochondrial	SDHA	5.67	-0.92726	-0.17111	-1.07368	-0.82179	-0.2347	-1.12975	36.24	12.31	42.51	15.32	-0.73	0.42	-0.41475	0.089082	-1.09168	-0.78321	-0.506	-1.37225	24.45	15.06	41.23	32.60	-0.68	0.52
P21912	Succinate dehydrogenase [ubiquinone] iron-sulfur subunit, mitochondrial	SDHB	1.60	-0.8077	-1.24073	-1.43076	-2.39007	NA	-0.64853	21.07	45.36	NA	38.11	-1.30	0.68	-0.67632	-0.76074	-1.47982	-1.36016	NA	-0.4256	4.14	5.86	NA	32.56	-0.94	0.46

UniProt	Protein name	Gene name	Peptide mean	08/017 hESC-RPE to hRPE												08/023 hESC-RPE to hRPE											
				Analysis						Technical replicate CV			Biological CV	Fold change (mean)	StDev	Analysis						Technical replicate CV			Biological CV	Fold change (mean)	StDev
				1.1	1.2	2.1	2.2	3.1	3.2	1	2	3				1.1	1.2	2.1	2.2	3.1	3.2	1	2	3			
A0A0C4DFU2	Superoxide dismutase	SOD2	6.00	-0.1575	0.02017	-1.38021	-1.20002	-0.46267	-0.31196	8.70	8.82	7.38	39.00	-0.58	0.57	-0.02319	0.074507	-0.88548	-0.82105	0.283158	0.32242	4.79	3.16	1.92	37.16	-0.17	0.54
V9HWC9	Superoxide dismutase [Cu-Zn]	SOD1	11.50	0.258467	0.121539	0.578143	0.378871	0.575074	0.631229	6.71	9.75	2.75	14.23	0.42	0.21	0.207984	0.254489	0.36122	0.341927	0.327665	0.448422	2.28	0.95	5.92	5.64	0.32	0.08
H9ZYJ2	Thioredoxin	TXN	2.80	0.329741	0.451124	NA	0.628462	0.316705	-0.97395	5.95	NA	59.35	27.25	0.15	0.64	0.21951	0.003741	NA	0.807968	-0.03178	-0.71927	10.56	NA	33.07	40.62	0.06	0.55
P30048	Thioredoxin-dependent peroxide reductase, mitochondrial	PRDX3	7.00	0.018969	0.552972	-0.3699	-0.44285	-0.01949	0.460563	25.88	3.57	23.31	25.02	0.03	0.41	0.257033	0.357665	-0.47102	-0.53706	-0.1736	0.363605	4.93	3.24	26.03	27.18	-0.03	0.41
A0A024R3W2	Translocase of outer mitochondrial membrane 20 homolog (Yeast), isoform CRA_a	TOMM20	1.67	0.675313	NA	-0.79778	-0.1051	NA	NA	NA	33.31	NA	50.83	-0.08	0.74	0.464363	NA	-0.58332	0.259371	NA	NA	NA	40.17	NA	27.38	0.05	0.56
A0A024R0P9	Translocase of outer mitochondrial membrane 40 homolog (Yeast), isoform CRA_c	TOMM40	1.67	0.426068	NA	0.429411	-0.29904	NA	NA	NA	34.96	NA	15.40	0.19	0.42	-0.12776	NA	-0.02862	-0.00937	NA	NA	NA	0.94	NA	5.33	-0.06	0.06
P55084	Trifunctional enzyme subunit beta, mitochondrial	HADHB	12.00	0.676915	0.442769	0.232768	0.474563	0.65475	0.450462	11.45	11.82	10.00	7.88	0.49	0.16	0.717464	0.350597	0.140239	0.326916	0.649857	0.717328	17.88	9.14	3.31	15.39	0.48	0.24
B4DLN1	Uncharacterized protein	NA	2.40	-0.23045	0.489624	-0.60404	-0.2958	NA	0.383786	34.58	15.05	NA	27.54	-0.05	0.47	-0.05016	0.998677	-1.24218	-0.89608	NA	-0.59118	49.26	16.88	NA	60.94	-0.36	0.87
A0A024RCN6	Valyl-tRNA synthetase, isoform CRA_a	VARS	2.00	1.074891	NA	NA	NA	NA	-0.33602	NA	NA	NA	64.12	0.37	1.00	1.236441	NA	NA	NA	NA	0.464958	NA	NA	NA	36.94	0.85	0.55
Q9P035	Very-long-chain (3R)-3-hydroxyacyl-CoA dehydratase 3	HACD3	4.17	-0.05068	0.080299	-0.5063	-0.79456	0.044664	-0.71727	6.42	14.08	36.50	22.53	-0.32	0.40	-0.74113	-0.10297	-0.63112	-1.17498	-0.16941	-0.40363	30.78	26.35	11.45	20.67	-0.54	0.40
A0A024R1Z6	Vesicle amine transport protein 1 homolog (T californica), isoform CRA_a	VAT1	8.50	0.171652	0.981853	-0.17357	-0.11477	0.432786	0.459432	38.70	2.88	1.31	26.09	0.29	0.43	-0.14075	0.823601	-0.57764	-0.46458	0.101781	-0.00568	45.58	5.54	5.26	31.33	-0.04	0.50
A0A024QZT0	Voltage-dependent anion channel 2, isoform CRA_c	VDAC2	16.17	-0.97212	-1.2679	-2.18618	-2.04403	-0.75414	-0.76907	14.45	6.96	0.73	42.51	-1.33	0.64	-0.96836	-1.07068	-2.15733	-2.04641	-1.02945	-1.00182	5.01	5.43	1.35	37.01	-1.38	0.56
P21796	Voltage-dependent anion-selective channel protein 1	VDAC1	29.00	-1.46926	-1.38399	-2.06146	-2.12681	-1.14001	-1.03722	4.18	3.20	5.04	33.06	-1.54	0.46	-1.38325	-1.35695	-2.00838	-1.99421	-0.99278	-0.87351	1.29	0.69	5.84	35.46	-1.43	0.48
Q9Y277	Voltage-dependent anion-selective channel protein 3	VDAC3	7.67	-0.51945	-0.81664	-1.11844	-0.86099	-0.20878	-0.42639	14.51	12.58	10.65	23.18	-0.66	0.33	-0.68299	-0.19104	-1.10522	-0.88625	-0.56903	-0.13301	23.88	10.71	21.21	22.94	-0.59	0.38
P38606	V-type proton ATPase catalytic subunit A	ATP6V1A	8.67	-0.98726	-0.83109	-0.41689	-0.34518	-0.77265	-0.99126	7.65	3.51	10.69	21.55	-0.72	0.28	-1.06087	-0.70939	-0.40085	-0.29648	-0.74083	-0.72244	17.14	5.11	0.90	19.49	-0.66	0.27

Supplementary Table S5. The 139 proteins involved in response to stress presented in Figure 4b. Differences in expression for both hESC-RPE lines compared to hRPE presented on log2 scale.
Mean number of peptides used for quantification, fold change value for each replicate analysis, technical replicate CV, biological replicate CV, mean fold change, and standard deviation shown for each protein. Note! CV values are calculated using normal scale, not the log2 scale shown here.
Differentially expressed proteins (>2-fold difference in expression i.e. values >1 and <-1 on log2 scale) marked with red color for upregulation and green color for downregulation.

				08/017 hESC-RPE to hRPE												08/023 hESC-RPE to hRPE											
				Analysis						Technical replicate CV						Analysis						Technical replicate CV					
UniProt	Protein name	Gene name	Peptide mean	1.1	1.2	2.1	2.2	3.1	3.2	1	2	3	Biological CV	Fold change (mean)	StDev	1.1	1.2	2.1	2.2	3.1	3.2	1	2	3	Biological CV	Fold change (mean)	StDev
P61604	10 kDa heat shock protein, mitochondrial	HSPE1	7.33	0.52446	0.484206	NA	NA	0.901593	NA	1.97	NA	NA	19.34	0.64	0.23	0.917834	0.766584	NA	NA	0.606649	NA	7.41	NA	NA	11.62	0.76	0.16
P35998	26S protease regulatory subunit 7	PSMC2	2.67	0.382338	-0.523	1.035475	2.071931	0.280219	0.490949	42.97	48.72	10.31	63.42	0.62	0.87	-0.23431	0.04063	1.255168	1.070065	-0.00165	1.094868	13.43	9.06	51.30	41.19	0.54	0.67
P62195	26S protease regulatory subunit 8	PSMC5	2.33	0.38445	-0.12236	NA	NA	-0.22928	NA	24.59	NA	NA	18.64	0.01	0.33	0.535932	-0.08136	NA	NA	0.062322	NA	29.80	NA	NA	9.68	0.17	0.32
O00231	26S proteasome non-ATPase regulatory subunit 11	PSMD11	3.00	NA	NA	NA	0.408966	0.075238	NA	NA	NA	NA	16.28	0.24	0.24	NA	NA	NA	-0.57904	0.219761	NA	NA	NA	NA	38.18	-0.18	0.56
O00487	26S proteasome non-ATPase regulatory subunit 14	PSMD14	1.50	NA	NA	NA	0.098951	0.660863	NA	NA	NA	NA	27.20	0.38	0.40	NA	NA	NA	0.137572	1.448961	NA	NA	NA	NA	60.19	0.79	0.93
P23396	40S ribosomal protein S3	RPS3	5.50	0.368071	0.286259	0.250701	0.427314	0.435787	0.705996	4.01	8.65	13.21	9.97	0.41	0.16	0.309782	0.47424	0.295315	0.285072	0.533677	0.559221	8.05	0.50	1.25	9.00	0.41	0.13
P24752	Acetyl-CoA acetyltransferase, mitochondrial	ACAT1	10.50	0.839969	0.62773	-0.12017	0.124568	0.739578	0.174817	10.38	11.97	27.33	24.55	0.40	0.39	0.670465	0.348867	-0.39286	0.105563	0.501028	0.128062	15.70	24.19	18.18	21.71	0.23	0.37
P61160	Actin-related protein 2	ACTR2	1.50	1.017997	NA	NA	NA	-0.5041	NA	NA	NA	NA	68.37	0.26	1.08	1.141303	NA	NA	NA	0.04198	NA	NA	NA	NA	51.42	0.59	0.78
P07108	Acyl-CoA-binding protein	DBI	4.50	0.826823	0.892953	0.118024	0.213197	0.304759	0.334088	3.24	4.66	1.44	26.47	0.45	0.33	0.348502	0.29672	0.218664	0.602769	0.284687	0.296855	2.54	18.72	0.60	4.85	0.34	0.13
Q5T4U5	Acyl-Coenzyme A dehydrogenase, C-4 to C-12 straight chain, isoform CRA_a	ACADM	6.00	0.258302	NA	0.134537	NA	NA	NA	NA	NA	NA	6.06	0.20	0.09	0.601376	NA	-0.10522	NA	NA	NA	NA	NA	NA	33.96	0.25	0.50
P23526	Adenosylhomocysteinase	AHCY	6.67	0.685631	0.542266	0.520551	0.544193	0.365263	0.760989	6.43	1.16	19.27	3.09	0.57	0.14	0.463728	0.538611	0.34597	0.67804	0.252589	0.250455	3.67	16.20	0.10	10.11	0.42	0.17
Q8N142	Adenylosuccinate synthetase isozyme 1	ADSSL1	5.00	1.800788	NA	1.573164	2.225313	0.245973	1.138237	NA	31.43	42.39	38.16	1.40	0.75	1.369856	NA	1.122449	1.982673	0.702395	0.740579	NA	40.96	1.87	29.59	1.18	0.53
P49588	Alanine--tRNA ligase, cytoplasmic	AARS	6.83	-0.45273	-0.78097	0.343657	0.68071	0.768259	0.418144	16.02	16.45	17.08	39.55	0.16	0.63	0.089833	-0.2886	-0.1025	0.340532	0.707596	0.04666	18.44	21.55	31.84	17.52	0.13	0.35
O43707	Alpha-actinin-4	ACTN4	22.83	0.61939	0.72729	0.600866	0.524718	0.588709	0.307041	5.29	3.73	13.76	7.61	0.56	0.14	0.75638	0.750695	0.94059	0.993424	0.73618	0.90281	0.28	2.59	8.16	7.65	0.85	0.11
Q5TZZ9	Annexin	ANXA1	12.67	0.454789	0.563415	-0.19253	-0.46524	-0.71576	-0.50624	5.32	13.33	10.25	42.52	-0.14	0.53	0.193686	0.344362	0.56637	0.372852	0.310761	0.399269	7.38	9.47	4.34	7.06	0.36	0.12
V9HWE0	Annexin	ANXA5	18.83	0.811987	0.898157	1.011878	0.990748	0.182105	0.066023	4.22	1.04	5.69	29.39	0.66	0.42	0.44018	0.617715	0.650834	0.519134	0.631421	0.555293	8.69	6.45	3.73	2.34	0.57	0.08
Q10567	AP-1 complex subunit beta-1	AP1B1	4.60	0.919135	0.933771	1.232881	1.284301	0.19974	NA	0.72	2.52	NA	34.55	0.91	0.43	0.720659	1.110978	1.432238	0.318239	-0.31358	NA	19.01	52.04	NA	42.00	0.65	0.68
Q5TZP7	APEX nuclease (Multifunctional DNA repair enzyme) 1	APEX1	5.80	-0.68237	-0.72939	0.529209	NA	-0.90827	-1.00737	2.30	NA	4.86	59.48	-0.56	0.62	-0.71572	-0.60851	1.016231	NA	-0.99566	-0.74165	5.25	NA	12.42	77.47	-0.41	0.81
A0A024R3E3	Apolipoprotein A-I, isoform CRA_a	APOA1	2.33	2.634717	2.317136	1.794767	2.021072	0.964121	0.421314	15.50	11.07	26.30	53.91	1.69	0.84	2.171774	2.231446	1.251878	1.297959	2.338172	1.711849	2.92	2.26	30.22	30.95	1.83	0.48
P02649	Apolipoprotein E	APOE	2.40	NA	0.463017	-0.40071	-0.26982	-0.76788	-0.13068	NA	6.41	30.73	36.02	-0.22	0.45	NA	0.988607	1.12512	-0.52104	-0.22391	0.423736	NA	72.94	31.22	29.63	0.36	0.72
O95831	Apoptosis-inducing factor 1, mitochondrial	AIFM1	10.67	-0.20686	-0.55221	-1.46109	-0.91167	-0.53779	-0.0817	16.85	26.61	22.17	29.75	-0.63	0.50	-0.07465	-0.46656	-1.32789	-0.79849	-0.46091	-0.26263	19.09	25.66	9.70	26.80	-0.57	0.44
O43681	ATPase ASNA1	ASNA1	3.50	-0.59088	0.424215	-0.18408	-1.35055	NA	-0.07632	47.80	54.25	NA	31.64	-0.36	0.66	-0.59133	-0.39556	0.014603	-0.4828	NA	-0.36948	9.58	24.14	NA	13.55	-0.36	0.23
Q01813	ATP-dependent 6-phosphofructokinase, platelet type	PFKP	10.50	-0.13809	-0.32359	0.372812	0.09772	0.5848	0.737616	9.08	13.44	7.48	30.29	0.22	0.42	-0.26321	-0.09424	0.137711	-0.07298	0.403411	0.230156	8.27	10.31	8.48	17.38	0.06	0.24
Q08211	ATP-dependent RNA helicase A	DHX9	7.83	0.265362	0.161633	-0.15723	0.47608	-0.06293	-0.23659	5.08	30.55	8.50	13.47	0.07	0.27	0.206655	0.397523	-0.08388	-0.06246	-0.33637	-0.23793	9.34	4.65	4.82	22.03	-0.04	0.28
P35613	Basigin	BSG	8.67	-2.19735	NA	-0.83417	-0.69432	NA	NA	NA	6.85	NA	65.05	-1.24	0.83	-1.26479	NA	-0.5033	-0.66846	NA	NA	NA	8.09	NA	32.78	-0.81	0.40
P07814	Bifunctional glutamate/proline--tRNA ligase	EPRS	3.00	-0.08681	1.409284	-0.03433	NA	1.19046	NA	67.39	NA	NA	39.16	0.62	0.79	-0.14462	1.361615	0.17786	NA	0.012559	NA	67.78	NA	NA	30.18	0.35	0.69
P04040	Catalase	CAT	8.83	0.236079	-0.16365	-0.33404	-0.3794	0.07961	0.38357	19.47	2.22	14.84	20.25	-0.03	0.31	0.311725	-0.01573	0.566712	0.251973	0.487228	0.747865	15.98	15.37	12.74	15.99	0.39	0.27
P21964	Catechol O-methyltransferase	COMT	4.17	-0.00296	-0.03516	0.291553	0.388972	0.124156	-0.58928	1.58	4.77	34.27	19.24	0.03	0.34	0.54076	0.341408	0.393751	0.321676	-0.43663	-0.6281	9.76	3.53	9.37	32.82	0.09	0.49
A0A024R374	Cathepsin B, isoform CRA_a	CTSB	6.83	0.799262	0.809523	-0.02109	-0.11249	0.602953	0.848202	0.50	4.48	11.99	29.85	0.49	0.44	0.322979	0.275759	-0.05047	-0.16561	0.351199	1.010665	2.31	5.64	31.77	28.37	0.29	0.41
P16070	CD44 antigen	CD44	1.00	0.313993	-0.16535	0.127275	0.434745	0.585545	0.433295	23.28	15.01	7.46	14.50	0.29	0.27	0.188695	-0.34794	0.382924	0.177392	0.259688	0.201769	26.00	10.06	2.84	12.18	0.14	0.25
Q6FHM9	CD59 antigen, complement regulatory protein, isoform CRA_b	CD59	2.00	0.87849	NA	NA	NA	NA	0.16464	NA	NA	NA	34.29	0.52	0.50	0.68866	NA	NA	NA	NA	0.942564	NA	NA	NA	12.41	0.82	0.18
B4DMA2	cDNA FLJ54023, highly similar to Heat shock protein HSP 90-beta	NA	27.00	0.332402	NA	0.510353	0.764104	0.240186	-0.17594	NA	12.41	20.26	20.63	0.33	0.35	0.220826	NA	0.411363	0.399498	0.10966	-0.17156	NA	0.58	13.74	14.74	0.19	0.24
B4DEA3	cDNA FLJ56531, highly similar to UV excision repair protein RAD23 homolog B	NA	4.00	0.432208	NA	NA	NA	0.310857	-0.06637	NA	NA	18.38	14.54	0.23	0.26	0.326304	NA	NA	NA	0.013379	-0.16171	NA	NA	8.57	19.38	0.06	0.25
B2RDN9	cDNA, FLJ96699, highly similar to Homo sapiens thyroid autoantigen 70kDa (Ku antigen) (G22P1), mRNA	NA	14.25	-0.71173	NA	NA	0.470573	-0.93635	-0.60059	NA	NA	16.38	52.55	-0.44	0.63	-0.37414	NA	NA	0.196457	-0.586	-0.51395	NA	NA	3.53	28.33	-0.32	0.35
P41208	Centrin-2	CETN2	1.25	-0.96596	-0.40696	-0.42826	NA	0.211498	NA	27.06	NA	NA	32.76	-0.40	0.48	0.234487	-0.09189	0.537021	NA	0.438166	NA	15.93	NA	NA	15.93	0.28	0.28
P10909	Clusterin	CLU	3.00	0.022462	-0.04884	0.232993	0.836901	-0.18857	NA	3.49	29.17	NA	28.71	0.17	0.40	0.253361	-0.55314	0.111013	0.807679	-0.01079	NA	38.53	33.50	NA	23.47	0.12	0.49
Q5KU26	Collectin-12	COLEC12	2.67	1.855229	1.554266	0.775154	1.569551	1.270517	0.994904	14.70	37.98	13.47	22.44	1.34	0.40	2.15145	1.633213	1.202811	2.124668	1.788893	1.007687	25.13	43.71	37.38	15.91	1.65	0.47
O14618	Copper chaperone for superoxide dismutase	CCS	1.00	NA	0.207893	0.477605	0.681203	NA	0.442268	NA	9.96	NA	12.90	0.45	0.19	NA	1.415809	1.09854	0.920504	NA	0.71967	NA	8.72	NA	24.50	1.04	0.30
V9HW27	Crystallin, alpha B, isoform CRA_a	CRYAB	7.50	0.659075	0.395929	0.143683	0.24313	1.218964	1.084734	12.86	4.87	6.57	34.71	0.62	0.45	0.885226	0.716504	0.451519	0.689039	1.140096	1.155232	8.26	11.62	0.74	20.26	0.84	0.28
P01034	Cystatin-C	CST3	1.00	0.412673	0.779647	0.193672	NA	NA	0.386492	17.89	NA	NA	14.40	0.44	0.24	0.370025	1.141465	0.512395	NA	NA	0.730421	36.93	NA	NA	10.34	0.69	0.34
P78527	DNA-dependent protein kinase catalytic subunit	PRKDC	6.50	-0.64218	0.24184	0.319222	0.138766	0.028582	-0.36686	42.02	8.83	19.26	15.67	-0.05	0.38	0.210069	-0.74403	-0.61591	-0.2649	0.19143	-0.38673	45.13	17.12	27.96	12.46	-0.27	0.40
A0A024R6S1	DnaJ (Hsp40) homolog, subfamily A, member 2, isoform CRA_a	DNAJA2	2.00	0.073114	-0.16557	0.26555	NA	NA	0.068316	11.67	NA	NA	10.92	0.06	0.18	0.066377	0.741761	0.487342	NA	NA	0.158305	32.51	NA	NA	11.94	0.36	0.31
P31689	DnaJ homolog subfamily A member 1	DNAJA1	1.00	-1.30558	NA	-0.60203	NA	NA	NA	NA	NA	NA	33.82	-0.95	0.50	-0.70497	NA	0.019854	NA	NA	NA	NA	NA	NA	34.80	-0.34	0.51
Q9UBS4	DnaJ homolog subfamily B member 11	DNAJB11	2.17	0.880202	1.044762	-0.13872	-0.18976	0.981783	0.488534	8.06	2.50	23.94	36.50	0.51	0.56	0.062815	1.034511	-0.15057	0.537853	1.524434	1.419863	45.90	33.12	1.72	47.16	0.75	0.71
P39656	Dolichyl-diphosphooligosaccharide--protein glycosyltransferase 48 kDa subunit	DDOST	4.50	0.488433	0.11787	-0.04523	0.224297	0.623968	0.812274	18.06	13.17	9.22	22.52	0.37	0.33	0.239136	0.129327	-0.03406	-0.11773	0.609497	0.768121	5.38	4.10	7.77	27.80	0.27	0.35
Q6DEN2	DPYSL3 protein	DPYSL3	21.00	-0.99195	-0.54555	-1.0466	-1.27141	-1.03259	-1.23393	21.71	11.00	9.85	16.28	-1.02	0.26	-1.15874	-0.63427	-1.04081	-0.9917	-1.1075	-1.43281	25.43	2.41	15.88	13.34	-1.06	0.26
Q96FJ2	Dynein light chain 2, cytoplasmic	DYNLL2	3.00	-0.73677	-1.00533	-0.56391	-0.51636	-0.11557	-0.19216	13.12	2.33	3.75	24.76	-0.52	0.33	-0.31134	-0.8327	-0.86736	-0.48139	-0.55377	-0.48609	25.28	18.81	3.32	5.13	-0.59	0.22
P13639	Elongation factor 2	EEF2	13.67	0.923397	1.02176	0.157294	0.676922	0.698563	1.171119	4.82	25.20	22.96	19.56	0.77	0.36	1.022826	1.130535	0.191886	0.720622	0.88423	1.240613	5.28	25.63	17.38	22.00	0.87	0.38
V9HW71	Endoplasmic reticulum resident protein 29	ERP29	6.50	2.68826	2.181228	-0.0219	-0.0618	0.212946	0.818778	24.60	1.96	29.26	93.89	0.97	1.19	2.610087	2.198027	0.152585	-0.03836	0.305249	0.139381	20.06	9.35	8.12	97.23	0.89	1.18
Q9BS26	Endoplasmic reticulum resident protein 44	ERP44	3.50	2.191439	0.534874	0.201991	0.252455	0.178898	0.447253	73.31	2.47	13.12	57.46	0.63	0.78	1.995391	0.624592	0.124539	0.339535	0.154379	0.26032	62.55	10.52	5.19	54.30	0.58	0.71
V9HWP2	Epididymis luminal protein 35	HSP90B1	31.17	0.495711	0.672366	0.45187	0.564479	0.84544	0.863896	8.65	5.52	0.90	12.90	0.65	0.18	0.399728	0.575012	0.271403	0.264632	0.674682	0.717328	8.58	0.33	2.09	14.76	0.48	0.20
V9HW21	Epididymis luminal protein 76	CA2	21.50	-2.24975	-2.1827	-2.546	-2.62519	-2.57167	-2.38963	3.29	3.88	8.91	13.46	-2.43	0.18	-2.30429	-2.39006	-2.01347	-1.81926	-2.49227	-2.22036	4.20	9.50	13.29	18.19	-2.21	0.25
K9JA46	Epididymis luminal secretory protein 52	EL52	35.00	-0.16675	-0.34441	-0.52678	-0.51034	-0.5886	0.056541	8.70	0.81	31.10	10.74	-0.35	0.25	-0.24206	-0.36288	-0.52863	-0.21455	-0.66478	-0.67075	5.92	15.33	0.29	13.09	-0.45	0.20
V9HW43	Epididymis secretory protein Li 102	HSPB1	8.00	0.834902	0.992387	0.606448	0.408569	0.660775	0.555071	7.71	9.68	5.18	15.06	0.68	0.21	0.624728	0.778966	0.560204	0.693883	0.373237	0.314695	7.55	6.55	2.87	12.70	0.56	0.18
V9HW75	Epididymis secretory protein Li 109	NUCB2	1.80	NA	0.797996	-0.03216	-0.13692	-0.53962	0.862964	NA	5.13	63.80	30.54	0.19	0.61	NA	0.436473	0.031786	0.3152	-0.42938	0.783874	NA	13.85	56.19	8.89	0.23	0.46
V9HW12	Epididymis secretory sperm binding protein Li 2a	PRDX2	18.50	-1.33359	-1.41605	-1.45672	-1.15119	-1.26143	-1.10006	4.04	14.92	7.90	6.87	-1.29	0.14	-1.6472	-1.57596	-1.55739	-1.35821	-1.93471	-1.80607	3.49	9.75	6.30	14.22	-1.65	0.20
V9HWC2	Epididymis secretory sperm binding protein Li 67p	PARK7	12.50	-0.59355	-0.33432	-0.20867	-0.32721	-0.35666	-0.1034	12.67	5.81	12.38	8.43	-0.32	0.16	-0.57068	-0.3232	-0.31713	-0.0595	-0.68192	-0.56118	12.10	12.59	5.91	15.42	-0.42	0.23
V9HWB4	Epididymis secretory sperm binding protein Li 89n	HSPA5	36.67	0.922878	0.761957	0.818482	1.064198	0.972125	0.975126	7.88	12.01	0.15	4.66	0.92	0.11	0.871039	0.806064	0.606646	0.732214	0.806912	0.783625	3.18	6.15	1.14	5.97	0.77	0.09
Q96HE7	ERO1-like protein alpha	ERO1L	2.00	NA	0.594926	0.02366	NA	NA	NA	NA	NA	NA	27.64	0.31	0.40	NA	0.948177	0.53859	NA	NA	NA	NA	NA	NA	19.94	0.74	0.29
Q9UK22	F-box only protein 2	FBXO2	1.25	1.623996	NA	0.96363	0.250462	NA	0.075711	NA	34.26	NA	55.43	0.73	0.71	1.447108	NA	0.011594	-0.52285	NA	-1.09205	NA	25.90	NA	89.54	-0.04	1.09
P21333	Filamin-A	FLNA	38.83	1.279703	1.27822	1.027436	0.960179	0.79485	1.030971	0.07	3.30	11.55	13.57	1.06	0.19	1.270175	1.293218	1.450871	1.393843	1.132476	1.327719	1.13	2.79	9.55	6.91	1.31	0.11
P09382	Galectin-1	LGALS1	6.83	0.763182	0.70359	0.594034	0.297339	0.751501	0.874264	2.92	14.49	6.01	12.70	0.66	0.20	0.638025	0.687653	1.158265	0.737521	1.134361	1.219714	2.43	20.48	4.18	17.59	0.93	0.27
Q08380	Galectin-3-binding protein	LGALS3BP	5.20	1.58896	1.811201	NA	0.254445	1.024396	1.362343	10.87	NA	16.49	45.92	1.21	0.61	1.008303	0.7056	NA	0.142839	0.583239	2.584213	14.78	NA	84.88	61.46	1.00	0.94
P14136	Glial fibrillary acidic protein	GFAP	12.50	NA	NA	NA	-1.46451	NA	-0.50684	NA	NA	NA	45.29	-0.99	0.68	NA	NA	NA	-1.62929	NA	-0.09479	NA	NA	NA	68.84	-0.86	1.09
A8YXX4	Glutamine synthetase	PIG59	10.83	1.116885	-0.20791	-1.24968	-1.52848	-1.29362	-1.9128	60.72	13.62	29.89	89.62	-0.85	1.12	1.009174	-0.13552	-0.79225	-1.19969	-0.90343	-1.73095	53.34	19.84	39.48	72.76	-0.63	0.96
Q6NSD4	Glutathione peroxidase	GPX1	1.50	1.187296	NA	0.888735	0.492101	NA	0.636057	NA	19.32	NA	21.85	0.80	0.31	1.347297	NA	0.741261	0.593935	NA	1.038553	NA	7.21	NA	23.12	0.93	0.33
A0A087X2I2	Glutathione peroxidase	GPX4	1.50	NA	0.568416	-0.3583	NA	NA	NA	NA	NA	NA	43.92	0.11	0.66	NA	0.819112	-0.00051	NA	NA	NA	NA	NA	NA	39.13	0.41	0.58
Q92896	Golgi apparatus protein 1	GLG1	1.67	0.703269	NA	-0.14048	0.814141	NA	NA	NA	45.15	NA	14.11	0.46	0.52	0.986903	NA	0.547448	0.662341	NA	NA	NA	5.63	NA	18.56	0.73	0.23
A0A024R056	Guanine nucleotide binding protein (G protein), beta polypeptide 1, isoform CRA_a	GNB1	36.50	-1.24042	NA	-2.15752	NA	NA	NA	NA	NA	NA	43.49	-1.70	0.65	-1.27165	NA	-1.9301	NA	NA	NA	NA	NA	NA	31.72	-1.60	0.47
P04899	Guanine nucleotide-binding protein G(i) subunit alpha-2	GNAI2	6.80	0.539571	0.43619	0.323253	NA	-0.70068	0.159588	5.06	NA	40.96	23.59	0.15	0.50	0.32805	0.285916	0.015355	NA	-0.08189	0.981035	2.06	NA	49.86	18.15	0.31	0.42
O60262	Guanine nucleotide-binding protein G(I)/G(S)/G(O) subunit gamma-7	GNG7	1.00	0.332871	-0.41782	-0.94513	-0.85812	NA	-0.36303	35.99	4.26	NA	30.34	-0.45	0.51	0.251337	-1.01084	-1.96327	-1.44624	NA	-1.19612	58.20	25.07	NA	52.40	-1.07	0.82
P09471	Guanine nucleotide-binding protein G(o) subunit alpha	GNAO1	9.60	-0.95142	-0.74045	-0.36819	NA	-1.72451	-1.18459	10.32	NA	26.16	35.55	-0.99	0.51	-1.28761	-0.96969	0.099249	NA	-1.11913	-0.7161	15.52	NA	19.63	48.42	-0.80	0.54
Q5JWF2	Guanine nucleotide-binding protein G(s) subunit alpha isoforms XLas	GNAS	4.00	-2.04093	-1.01719	-1.34342	NA	-0.69479	-0.77245	48.17	NA	3.81	28.11	-1.17	0.55	-1.20851	-0.78066	-0.03587	NA	-1.82222	-0.68686	20.82	NA	52.94	44.59	-0.91	0.66
P63211	Guanine nucleotide-binding protein G(T) subunit gamma-T1	GNGT1	7.67	-2.13723	-2.4447	-2.92436	-3.24056	-2.59344	-2.40752	15.01	15.44	9.10	26.46	-2.62	0.40	-2.36508	-2.48063	-2.70551	-3.21895	-2.83175	-2.6281	5.66	24.90	9.97	18.24	-2.71	0.30
A0A024R3X4	Heat shock 60kDa protein 1 (Chaperonin), isoform CRA_a	HSPD1	41.17	-0.24718	-0.40671	-0.80101	-0.93618	0.498082	1.452736	7.81	6.62	45.15	71.79	-0.07	0.90	-0.05676	-0.34151	-0.94367	-0.84987	0.252589	0.858863	13.91	4.60	29.29	50.40	-0.18	0.68
Q5SSJ5	Heterochromatin protein 1-binding protein 3	HP1BP3	4.83	-1.51297	-1.2538	-2.13756	-2.00753	-1.14556	-1.33656	12.67	6.37	9.35	28.10	-1.57	0.41	-1.48079	-1.02198	-1.40353	-1.4881	-1.705	-1.35025	22.30	4.14	17.30	10.43	-1.41	0.22
A0A024RDR0	High-mobility group box 1, isoform CRA_a	HMGB1	4.00	-0.98375	-0.79309	-0.97087	NA	NA	-0.73268	9.33	NA	NA	8.45	-0.87	0.13	-0.53468	-0.71336	-1.28717	NA	NA	-1.16843	8.75	NA	NA	25.88	-0.93	0.36
Q5U071	High-mobility group box 2	NA	4.25	-0.95256	-1.12816	NA	NA	-0.49264	-1.43859	8.60	NA	44.77	7.26	-1.00	0.40	-0.77448	-1.0781	NA	NA	-0.58042	-1.23037	14.83	NA	31.33	2.41	-0.92	0.29
Q6IBN1	HNRPK protein	HNRPK	10.33	-0.37843	-0.33839	0.423494	0.291965	-0.75367	-0.45226	1.96	6.44	14.72	36.29	-0.20	0.46	0.033998	0.075762	0.303335	0.450434	-0.71956	-0.33451	2.05	7.20	18.76	29.70	-0.03	0.43
P35914	Hydroxymethylglutaryl-CoA lyase, mitochondrial	HMGCL	1.00	NA	-0.29117	-1.43792	NA	-0.37612	0.152612	NA	NA	25.63	42.43	-0.49	0.67	NA	0.358009	-0.34772	NA	-0.09155	0.577127	NA	NA	32.20	24.60	0.12	0.42
P54868	Hydroxymethylglutaryl-CoA synthase, mitochondrial	HMGCS2	9.33	1.134297	0.95498	1.136603	1.2448	1.098142	1.145154	8.78	4.35	2.30	5.33	1.12	0.10	0.57205	0.602847	0.340584	0.736461	0.205486	0.749139	1.51	19.28	26.34	2.94	0.53	0.22
P05556	Integrin beta-1	ITGB1	2.33	0.149462	-0.28122	0.62839	0.613721	0.735134	0.260371	20.95	0.72	23.06	23.18	0.35	0.39	-0.25717	-0.68085	0.93993	0.767182	0.983781	0.313317	20.62	8.46	32.28	41.53	0.34	0.69
V9HWJ2	Isocitrate dehydrogenase [NADP]	IDH1	6.67	-0.43532	1.812354	0.441368	-0.07535	0.250916	0.312855	79.92	25.06	3.04	22.07	0.32	0.62	-0.01431	1.536486	0.827637	0.080245	-0.01183	1.108833	69.45	35.83	52.32	16.52	0.59	0.66
P05787	Keratin, type II cytoskeletal 8	KRT8	17.50	1.785737	1.388262	1.527923	1.812191	1.052638	1.506304	19.36	13.89	22.05	13.42	1.51	0.28	1.86948	1.38205	1.650753	1.886236	0.998759	1.810548	23.67	11.52	38.77	10.76	1.60	0.35
W8QEY1	Lactoferrin	NA	3.00	1.911673	1.380773	NA	NA	NA	1.736472	25.73	NA	NA	3.23	1.68	0.27	1.926334	1.017848	NA	NA	NA	2.163906	43.11	NA	NA	30.00	1.70	0.60
Q01650	Large neutral amino acids transporter small subunit 1	SLC7A5	1.25	NA	0.873917	NA	0.863226	3.253014	1.83875	NA	NA	64.25	80.25	1.71	1.13	NA	1.259375	NA	1.010685	3.038763	1.584927	NA	NA	65.78	59.14	1.72	0.91
Q08722	Leukocyte surface antigen CD47	CD47	2.33	-1.16168	-1.18702	-1.03081	-1.11534	-1.15083	-1.21412	1.24	4.14	3.10	4.29	-1.14	0.06	-1.17943	-0.60683	-0.40554	-1.187	-0.85363	-0.93521	27.70	37.39	4.00	5.58	-0.86	0.31
P09960	Leukotriene A-4 hydrolase	LTA4H	2.50	0.807383	NA	NA	NA	0.476141	NA	NA	NA	NA	16.16	0.64	0.23	-0.2248	NA	NA	NA	-0.05672	NA	NA	NA	NA	8.23	-0.14	0.12
I4AY87	Macrophage migration inhibitory factor (Fragment)	MIF	7.17	1.511884	1.464049	1.816903	1.642547	1.995871	2.328022	2.34	8.54	16.21	24.58	1.79	0.33	1.49306	1.608784	1.451427	1.373144	1.39628	1.740643	5.67	3.84	16.80	6.07	1.51	0.14
G1EP01	MHC class I antigen (Fragment)	HLA-A	1.67	NA	NA	-1.09674	NA	0.176674	0.313083	NA	NA	6.68	61.46	-0.20	0.78	NA	NA	-1.10196	NA	0.006808	0.447951	NA	NA	21.45	61.57	-0.22	0.80
O94826	Mitochondrial import receptor subunit TOM70	TOMM70A	10.00	0.038842	0.069666	-1.39129	NA	NA	NA	1.51	NA	NA	65.47	-0.43	0.83	0.093945	-0.18126	-1.22315	NA	NA	NA	13.45	NA	NA	55.06	-0.44	0.69

UniProt	Protein name	Gene name	Peptide mean	08/017 hESC-RPE to hRPE												08/023 hESC-RPE to hRPE											
				Analysis						Technical replicate CV			Biological CV	Fold change (mean)	StDev	Analysis						Technical replicate CV			Biological CV	Fold change (mean)	StDev
				1.1	1.2	2.1	2.2	3.1	3.2	1	2	3				1.1	1.2	2.1	2.2	3.1	3.2	1	2	3			
Q1HBJ4	Mitogen-activated protein kinase	MAPK1	2.75	NA	NA	0.147517	-0.26823	-1.00933	-0.71193	NA	20.24	14.52	38.58	-0.46	0.51	NA	NA	-0.25904	-0.13121	-0.91625	-0.41073	NA	6.26	24.53	21.77	-0.43	0.34
P35580	Myosin-10	MYH10	10.17	1.357816	1.470366	0.552529	0.574781	0.442283	0.37564	5.51	1.09	3.27	40.21	0.80	0.49	1.376116	1.211269	0.285798	1.163715	0.740394	0.386223	8.07	41.75	17.27	26.61	0.86	0.46
Q69YG1	Myotrophin	DKFZp761E1322	2.67	1.041522	1.219841	1.265219	0.94369	0.620517	0.717215	8.73	15.69	4.74	17.14	0.97	0.26	1.147053	1.116579	1.24858	0.91208	0.734689	1.191025	1.49	16.42	22.18	5.35	1.06	0.20
P06748	Nucleophosmin	NPM1	7.67	0.599781	0.343483	1.322935	0.611135	0.635892	0.643133	12.53	34.20	0.35	19.48	0.69	0.33	0.658437	0.706411	1.048766	0.292303	0.114252	0.503375	2.35	36.25	18.96	14.53	0.55	0.33
O75475	PC4 and SFRS1-interacting protein	PSIP1	2.80	-1.58496	-1.40696	-1.37401	-1.01817	NA	-0.73951	8.71	17.35	NA	26.63	-1.22	0.34	-2.06912	-1.28216	-0.7406	-0.94285	NA	-0.81207	37.64	9.90	NA	28.58	-1.17	0.54
Q02790	Peptidyl-prolyl cis-trans isomerase FKBP4	FKBP4	1.50	-0.34955	NA	NA	0.147122	NA	NA	NA	NA	NA	24.11	-0.10	0.35	-0.64686	NA	NA	0.67662	NA	NA	NA	NA	NA	60.67	0.01	0.94
Q06830	Peroxiredoxin-1	PRDX1	17.50	0.03462	0.066588	0.110187	0.35907	0.106996	0.038215	1.57	12.17	3.37	7.31	0.12	0.12	0.179438	0.234586	0.125468	0.141009	-0.30487	-0.3915	2.70	0.76	4.24	19.58	0.00	0.27
B1AKZ4	Phosphoprotein enriched in astrocytes 15, isoform CRA_a	PEA15	5.17	0.49599	-1.85905	-1.6478	-1.72275	-2.40767	-1.61308	95.18	3.67	37.99	68.77	-1.46	1.00	0.564513	-0.81398	-1.20437	-0.97672	-1.70163	-1.43281	62.85	11.13	13.14	59.44	-0.93	0.80
P36955	Pigment epithelium-derived factor	SERPINF1	3.17	0.815571	1.024376	0.644927	0.269614	0.412975	0.251851	10.22	18.29	7.89	22.27	0.57	0.31	0.730729	0.683406	0.197634	0.607249	0.361375	0.200093	2.32	19.94	7.90	15.37	0.46	0.24
P02545	Prelamin-A/C	LMNA	20.50	0.108054	0.455838	0.610099	0.542927	-0.31607	-0.28296	16.96	3.29	1.62	29.08	0.19	0.41	0.028853	0.188148	0.289323	0.415668	-0.19664	-0.05199	7.80	6.19	7.08	16.48	0.11	0.23
P07737	Profilin-1	PFN1	10.00	0.757371	0.545318	0.405597	0.595168	1.437388	0.893868	10.37	9.28	26.33	26.22	0.77	0.37	0.731521	0.68738	1.107846	1.253985	1.554453	0.934521	2.16	7.16	29.93	19.82	1.04	0.33
A0A024QZQ2	Prosaposin (Variant Gaucher disease and variant metachromatic leukodystrophy), isoform CRA_b	PSAP	20.75	NA	-0.80997	NA	-0.47583	-0.46595	-0.76739	NA	NA	14.72	11.51	-0.63	0.18	NA	-0.78294	NA	-1.1279	-0.12431	-0.35929	NA	NA	11.49	31.76	-0.60	0.45
P49720	Proteasome subunit beta type-3	PSMB3	2.00	-0.67418	NA	-0.58205	-0.64319	NA	NA	NA	3.00	NA	3.03	-0.63	0.05	-0.51013	NA	-0.27273	-0.13647	NA	NA	NA	6.67	NA	15.00	-0.31	0.19
P28070	Proteasome subunit beta type-4	PSMB4	2.67	-0.30218	NA	NA	0.230153	-1.10802	NA	NA	NA	NA	43.45	-0.39	0.67	1.122103	NA	NA	-0.77887	-0.08299	NA	NA	NA	NA	67.69	0.09	0.96
Q15084	Protein disulfide-isomerase A6	PDIA6	11.33	0.975682	1.075533	0.578321	0.601958	0.943917	0.659958	4.89	1.16	13.87	15.07	0.81	0.22	0.944266	0.906229	0.374164	0.374021	0.886021	0.72019	1.86	0.01	8.12	19.04	0.70	0.26
B2R4M6	Protein S100	NA	2.20	NA	-0.53341	-1.98707	-1.73938	-2.31433	-2.80467	NA	12.11	23.80	72.32	-1.88	0.85	NA	0.153897	-1.67691	-1.12734	-3.03707	-2.4691	NA	26.61	27.48	91.20	-1.63	1.24
P05109	Protein S100-A8	S100A8	1.33	-0.73218	-0.64801	-1.5924	-1.54839	-2.52185	-2.3978	4.12	2.16	6.08	58.53	-1.57	0.79	-0.49832	0.144159	-1.4136	-1.15237	-2.75298	-2.29615	30.98	12.77	22.21	74.79	-1.33	1.08
P04271	Protein S100-B	S100B	2.40	-0.18611	NA	0.516666	-0.13518	-0.58482	-0.57426	NA	31.42	0.52	27.79	-0.19	0.45	2.362759	NA	1.477197	1.572883	0.486047	0.675179	NA	4.69	9.26	57.98	1.31	0.76
Q70J99	Protein unc-13 homolog D	UNC13D	3.33	NA	NA	1.224058	0.248022	0.496201	NA	NA	46.09	NA	15.66	0.66	0.51	NA	NA	0.71668	0.052531	-0.23312	NA	NA	31.99	NA	31.59	0.18	0.49
A0A024R5Z9	Pyruvate kinase	PKM2	64.80	NA	-2.15032	-1.56313	-1.58272	-1.62802	-1.86826	NA	0.96	11.75	19.67	-1.76	0.25	NA	-2.58518	-1.72629	-1.86266	-1.65465	-1.9759	NA	6.68	15.68	28.19	-1.96	0.37
A0A024R845	RAB14, member RAS oncogene family, isoform CRA_a	RAB14	5.67	-0.40738	-0.94854	-0.04223	0.214788	-0.1014	-0.2421	26.22	12.56	6.89	25.01	-0.25	0.40	-1.03011	-0.68651	-0.13049	0.078574	-0.15737	-0.71399	16.76	10.23	26.95	28.10	-0.44	0.43
Q9H0U4	Ras-related protein Rab-1B	RAB1B	6.80	-1.39232	NA	-0.18197	-0.75087	-0.29225	0.036975	NA	27.53	16.07	40.41	-0.52	0.57	-0.34101	NA	-0.81923	-0.67732	-1.10929	-0.44209	NA	6.95	32.13	16.72	-0.68	0.31
P51149	Ras-related protein Rab-7a	RAB7A	7.83	0.421519	-0.15053	0.05206	-0.02379	0.85258	0.739988	27.68	3.72	5.52	30.42	0.32	0.42	0.344193	-0.10273	-0.483	-0.39989	-0.0303	-0.24147	21.73	4.07	10.33	19.84	-0.15	0.30
Q5JR08	Rho-related GTP-binding protein RhoC (Fragment)	RHOC	3.67	NA	NA	-0.55649	0.553564	NA	-0.13579	NA	51.87	NA	11.67	-0.05	0.56	NA	NA	0.047787	0.277389	NA	0.116062	NA	11.23	NA	2.50	0.15	0.12
A0A024QYX3	RNA binding motif (RNP1, RRM) protein 3, isoform CRA_c	RBM3	1.00	0.146797	NA	NA	NA	-1.5667	-1.89115	NA	NA	15.84	80.55	-1.10	1.10	-0.49647	NA	NA	NA	-0.73979	-1.88216	NA	NA	53.24	33.85	-1.04	0.74
Q9Y230	RuvB-like 2	RUVBL2	2.33	NA	0.890191	1.024833	-0.45184	NA	NA	NA	66.66	NA	20.56	0.49	0.82	NA	0.987496	0.807102	-0.2272	NA	NA	NA	48.63	NA	29.31	0.52	0.66
Q6IRT1	S-(hydroxymethyl)glutathione dehydrogenase	ADH5	5.67	0.391821	0.005495	0.008193	0.311853	0.489246	0.218593	18.82	14.83	13.23	7.09	0.24	0.20	0.773017	0.20965	-0.02193	0.194032	-0.05801	0.399431	27.27	10.57	22.23	16.04	0.25	0.31
P16615	Sarcoplasmic/endoplasmic reticulum calcium ATPase 2	ATP2A2	6.40	0.197116	0.484342	0.173602	0.011165	NA	-0.2066	14.03	7.95	NA	18.99	0.13	0.25	0.45975	0.527341	0.125932	-0.16306	NA	-0.00784	3.31	14.12	NA	21.17	0.19	0.30
P62136	Serine/threonine-protein phosphatase PP1-alpha catalytic subunit	PPP1CA	3.20	0.190577	0.014731	0.765987	0.362765	NA	0.795598	8.61	19.64	NA	23.27	0.43	0.35	0.650781	0.342104	0.620704	-0.93608	NA	0.372397	15.07	69.67	NA	15.91	0.21	0.66
Q13813	Spectrin alpha chain, non-erythrocytic 1	SPTAN1	53.83	-0.32691	-0.4504	-0.34477	-0.34217	-0.58168	-0.44763	6.05	0.13	6.57	6.02	-0.42	0.10	-0.40921	-0.41335	-0.34514	-0.27984	-0.64395	-0.41843	0.20	3.20	11.03	7.42	-0.42	0.12
O15020	Spectrin beta chain, non-erythrocytic 2	SPTBN2	7.17	0.689786	0.326325	0.678602	1.423683	0.228302	0.513301	17.72	35.73	13.92	27.85	0.64	0.42	0.787308	0.742157	1.53737	2.324298	-0.66314	0.60428	2.21	37.64	58.41	67.53	0.89	1.00
P63208	S-phase kinase-associated protein 1	SKP1	4.83	-0.14831	-0.21274	0.984276	0.980363	-0.19016	-0.07171	3.16	0.19	5.80	49.48	0.22	0.59	0.076803	-0.2835	0.936496	1.191935	-0.33376	0.411393	17.57	12.49	35.73	46.67	0.33	0.63
Q9UJZ1	Stomatin-like protein 2, mitochondrial	STOML2	5.33	NA	NA	-0.22098	-0.06318	0.549418	NA	NA	7.73	NA	33.16	0.09	0.41	NA	NA	-0.37502	-0.83764	0.726465	NA	NA	22.48	NA	60.30	-0.16	0.80
Q14683	Structural maintenance of chromosomes protein 1A	SMC1A	1.00	1.590398	NA	NA	1.520956	NA	NA	NA	NA	NA	3.40	1.56	0.05	1.219604	NA	NA	0.950977	NA	NA	NA	NA	NA	13.13	1.09	0.19
A0A0C4DFU2	Superoxide dismutase	SOD2	6.00	-0.1575	0.02017	-1.38021	-1.20002	-0.46267	-0.31196	8.70	8.82	7.38	39.00	-0.58	0.57	-0.02319	0.074507	-0.88548	-0.82105	0.283158	0.32242	4.79	3.16	1.92	37.16	-0.17	0.54
V9HWC9	Superoxide dismutase [Cu-Zn]	SOD1	11.50	0.258467	0.121539	0.578143	0.378871	0.575074	0.631229	6.71	9.75	2.75	14.23	0.42	0.21	0.207984	0.254489	0.36122	0.341927	0.327665	0.448422	2.28	0.95	5.92	5.64	0.32	0.08
Q9Y490	Talin-1	TLN1	12.67	0.72129	1.513624	0.949289	1.809034	0.997622	1.045696	37.89	40.93	2.36	15.03	1.17	0.41	0.899862	0.95615	0.565002	1.529835	1.094343	1.168056	2.76	45.60	3.61	7.85	1.04	0.32
O95881	Thioredoxin domain-containing protein 12	TXNDC12	1.00	-0.10575	NA	-0.0168	NA	NA	NA	NA	NA	NA	4.36	-0.06	0.06	-0.94412	NA	-0.61475	NA	NA	NA	NA	NA	NA	16.07	-0.78	0.23
P30048	Thioredoxin-dependent peroxide reductase, mitochondrial	PRDX3	7.00	0.018969	0.552972	-0.3699	-0.44285	-0.01949	0.460563	25.88	3.57	23.31	25.02	0.03	0.41	0.257033	0.357665	-0.47102	-0.53706	-0.1736	0.363605	4.93	3.24	26.03	27.18	-0.03	0.41
Q9H3N1	Thioredoxin-related transmembrane protein 1	TMX1	1.50	NA	-0.99129	-1.80934	NA	NA	NA	NA	NA	NA	39.05	-1.40	0.58	NA	-0.33955	-1.90335	NA	NA	NA	NA	NA	NA	69.93	-1.12	1.11
P68366	Tubulin alpha-4A chain	TUBA4A	38.00	-1.32715	-1	-0.75421	-0.2128	-1.78633	-1.73904	15.97	26.23	2.32	44.74	-1.14	0.61	-1.45713	-1.18026	-0.16298	-0.45833	-1.07534	-1.63473	13.53	14.43	27.08	44.07	-0.99	0.57
Q9UM54	Unconventional myosin-VI	MYO6	1.50	NA	NA	-0.63828	-1.10964	-0.96499	-1.16989	NA	22.90	10.03	10.23	-0.97	0.24	NA	NA	-0.7018	-0.72324	-0.47061	-1.6434	NA	1.05	54.51	11.18	-0.88	0.52
Q53F10	UV excision repair protein RAD23 homolog B variant (Fragment)	NA	2.50	NA	-0.57098	-0.28962	NA	NA	NA	NA	NA	NA	13.75	-0.43	0.20	NA	-0.60482	0.666543	NA	NA	NA	NA	NA	NA	58.57	0.03	0.90
Q9P035	Very-long-chain (3R)-3-hydroxyacyl-CoA dehydratase 3	HACD3	4.17	-0.05068	0.080299	-0.5063	-0.79456	0.044664	-0.71727	6.42	14.08	36.50	22.53	-0.32	0.40	-0.74113	-0.10297	-0.63112	-1.17498	-0.16941	-0.40363	30.78	26.35	11.45	20.67	-0.54	0.40
P21796	Voltage-dependent anion-selective channel protein 1	VDAC1	29.00	-1.46926	-1.38399	-2.06146	-2.12681	-1.14001	-1.03722	4.18	3.20	5.04	33.06	-1.54	0.46	-1.38325	-1.35695	-2.00838	-1.99421	-0.99278	-0.87351	1.29	0.69	5.84	35.46	-1.43	0.48
P38606	V-type proton ATPase catalytic subunit A	ATP6V1A	8.67	-0.98726	-0.83109	-0.41689	-0.34518	-0.77265	-0.99126	7.65	3.51	10.69	21.55	-0.72	0.28	-1.06087	-0.70939	-0.40085	-0.29648	-0.74083	-0.72244	17.14	5.11	0.90	19.49	-0.66	0.27
P21281	V-type proton ATPase subunit B, brain isoform	ATP6V1B2	12.33	-0.24158	-0.6087	-0.20165	-0.64155	-0.23832	-0.35163	17.90	21.40	5.55	4.68	-0.38	0.20	-0.5772	-0.34207	-0.1472	-0.37383	-0.25023	0.050386	11.50	11.08	14.68	12.49	-0.27	0.21
P13010	X-ray repair cross-complementing protein 5	XRCC5	10.83	-0.45948	-0.18875	0.546371	0.330423	-0.53273	-0.66524	13.23	10.56	6.49	39.23	-0.16	0.49	-0.52016	-0.26802	0.286213	-0.00759	-0.74741	-0.75676	12.33	14.35	0.46	31.83	-0.34	0.42

Supplementary Table S6. The 189 cytoskeletal proteins presented in Figure 4c. Differences in expression for both hESC-RPE lines compared to hRPE presented on log2 scale.
Mean number of peptides used for quantification, fold change value for each replicate analysis, technical replicate CV, biological replicate CV, mean fold change, and standard deviation shown for each protein. Note! CV values are calculated using normal scale, not the log2 scale shown here.
Differentially expressed proteins (>2-fold difference in expression i.e. values >1 and <-1 on log2 scale) marked with red color for upregulation and green color for downregulation.

UniProt	Protein name	Gene name	Peptide mean	08/017 hESC-RPE to hRPE													08/023 hESC-RPE to hRPE												
				Analysis						Technical replicate CV			Biological CV	Fold change (mean)	StDev	Analysis						Technical replicate CV			Biological CV	Fold change (mean)	StDev
				1.1	1.2	2.1	2.2	3.1	3.2	1	2	3				1.1	1.2	2.1	2.2	3.1	3.2	1	2	3			
P62280	40S ribosomal protein S11	RPS11	2.20	0.591836	0.393619	NA	0.629826	1.177416	1.003177	9.70	NA	8.53	22.62	0.76	0.32	0.894567	0.865083	NA	0.43521	0.529958	0.727579	1.45	NA	9.67	15.53	0.69	0.20
P25398	40S ribosomal protein S12	RPS12	5.00	0.486186	0.343333	0.598767	0.573719	1.751044	1.12715	7.00	1.23	30.11	42.13	0.81	0.53	0.490489	0.468199	0.611458	0.745069	1.271642	0.958627	1.09	6.54	15.28	23.57	0.76	0.31
J3KMX5	40S ribosomal protein S13	RPS13	2.00	-0.1213	NA	NA	NA	NA	0.992278	NA	NA	NA	52.02	0.44	0.79	-0.89399	NA	NA	NA	NA	0.893735	NA	NA	NA	77.90	0.00	1.26
P08708	40S ribosomal protein S17	RPS17	2.67	1.938882	0.249525	0.234346	0.155194	0.515203	-0.62458	74.48	3.88	53.13	52.47	0.41	0.84	1.060611	0.031603	-0.34707	-0.05504	0.053981	-1.23641	48.40	14.26	59.34	41.73	-0.08	0.74
P62269	40S ribosomal protein S18	RPS18	2.00	-0.26457	-1.09899	0.46978	0.432207	0.246209	0.454387	39.79	1.84	10.19	35.61	0.04	0.62	-0.02141	-0.75032	0.44447	0.564888	0.357991	0.214372	34.98	5.90	7.03	28.17	0.13	0.48
P60866	40S ribosomal protein S20	RPS20	2.60	0.359965	NA	0.648144	0.255992	0.841701	1.048022	NA	19.10	10.10	22.77	0.63	0.33	0.962597	NA	0.741564	0.703988	0.800753	0.23125	NA	1.84	27.56	14.67	0.69	0.27
Q8WVC2	40S ribosomal protein S21	RPS21	1.83	0.303983	0.199486	0.864774	0.987162	0.709803	0.2586	5.12	5.99	21.94	24.15	0.55	0.34	0.601665	0.982709	1.213872	1.143639	-0.00083	0.556749	18.57	3.44	26.99	29.43	0.75	0.46
P23396	40S ribosomal protein S3	RPS3	5.50	0.368071	0.286259	0.250701	0.427314	0.435787	0.705996	4.01	8.65	13.21	9.97	0.41	0.16	0.309782	0.47424	0.295315	0.285072	0.533677	0.559221	8.05	0.50	1.25	9.00	0.41	0.13
Q6NXR8	40S ribosomal protein S3a	RPS3A	4.17	-0.03971	-0.53039	0.035087	-0.14302	0.38145	0.566854	23.82	8.72	9.07	27.49	0.05	0.39	-0.00022	0.149334	0.473739	0.112115	0.301395	0.438661	7.32	17.63	6.72	10.44	0.25	0.19
Q96DV6	40S ribosomal protein S6	RPS6	1.75	NA	NA	1.252037	-0.55845	0.421141	1.052935	NA	78.67	30.48	7.71	0.54	0.81	NA	NA	1.132641	0.035889	0.60882	0.753336	NA	51.31	7.08	0.16	0.63	0.46
P62081	40S ribosomal protein S7	RPS7	3.17	0.091152	-0.48638	0.329805	0.611825	-0.26025	-0.17403	27.93	13.78	4.22	28.55	0.02	0.41	0.200757	-0.28108	-0.20379	0.264212	0.421125	0.54711	23.40	22.74	6.17	19.84	0.16	0.33
Q5JR95	40S ribosomal protein S8	RPS8	2.50	0.738264	NA	0.419536	0.710981	0.440533	NA	NA	14.24	NA	10.39	0.58	0.17	0.95719	NA	0.295728	0.476604	0.678951	NA	NA	8.85	NA	19.56	0.60	0.28
C9J9K3	40S ribosomal protein SA (Fragment)	RPSA	4.17	0.896578	0.719818	0.55974	0.977019	2.050986	0.433714	8.65	20.31	71.89	28.11	0.94	0.58	0.610733	0.6856	0.471915	0.852253	1.813869	0.455309	3.67	18.53	62.07	26.62	0.81	0.51
A0A087WV22	60S ribosomal protein L10	RPL10	1.33	NA	0.427324	0.96621	0.672463	NA	NA	NA	14.35	NA	19.46	0.69	0.27	NA	0.121043	1.070016	0.522827	NA	NA	NA	26.50	NA	33.71	0.57	0.48
P62906	60S ribosomal protein L10a	RPL10A	2.40	-0.26268	0.10469	0.119976	NA	0.518329	0.433295	17.91	NA	4.17	19.58	0.18	0.31	-0.06751	0.378664	0.264889	NA	0.507588	0.517354	21.70	NA	0.48	12.45	0.32	0.24
P30050	60S ribosomal protein L12	RPL12	3.40	0.537945	0.631411	0.876083	0.639673	0.91099	NA	4.58	11.56	NA	11.22	0.72	0.16	0.624728	0.638673	0.799125	0.89291	0.447445	NA	0.68	4.60	NA	13.89	0.68	0.17
A8K4C8	60S ribosomal protein L13	RPL13	3.25	NA	NA	0.90383	0.336699	0.649858	0.22888	NA	27.44	20.49	9.46	0.53	0.31	NA	NA	0.602319	0.211791	-0.18132	0.432963	NA	19.02	29.66	12.80	0.27	0.34
M0R3D6	60S ribosomal protein L18a (Fragment)	RPL18A	1.00	NA	NA	NA	1.449878	NA	-0.08065	NA	NA	NA	68.69	0.68	1.08	NA	NA	NA	0.966041	NA	0.663061	NA	NA	NA	14.80	0.81	0.21
K7ELC7	60S ribosomal protein L27 (Fragment)	RPL27	1.20	0.5154	0.220493	NA	1.613635	1.183829	0.682852	14.40	NA	24.31	42.52	0.84	0.55	0.569247	0.554753	NA	1.349275	1.09415	0.712763	0.71	NA	18.58	27.44	0.86	0.35
E5RI99	60S ribosomal protein L30 (Fragment)	RPL30	2.00	-0.4835	NA	-0.23526	0.552126	-0.27253	0.635784	NA	37.66	43.10	26.00	0.04	0.52	0.241918	NA	-0.38424	0.297651	-0.61864	0.535783	NA	32.81	53.74	8.84	0.01	0.49
P18077	60S ribosomal protein L35a	RPL35A	1.00	0.47107	NA	-0.1329	0.483264	NA	NA	NA	29.75	NA	12.87	0.27	0.35	0.522113	NA	0.371232	0.563522	NA	NA	NA	9.41	NA	2.53	0.49	0.10
Q9HBB3	60S ribosomal protein L6	NA	1.25	0.761558	NA	1.442441	NA	0.100361	1.024177	NA	NA	43.79	31.95	0.83	0.56	0.525466	NA	1.76036	NA	0.153078	0.859099	NA	NA	33.93	53.33	0.82	0.69
E9PKZ0	60S ribosomal protein L8 (Fragment)	RPL8	1.00	NA	NA	1.567273	-0.31174	NA	1.1219	NA	80.96	NA	10.16	0.79	0.98	NA	NA	1.919539	0.754972	NA	0.7198	NA	54.17	NA	35.12	1.13	0.68
Q53Z07	60S ribosomal protein L9	RPL9	3.67	1.228874	0.099207	0.422901	0.42437	1.023365	0.401763	52.70	0.07	30.00	12.90	0.60	0.43	1.121398	0.318972	0.494724	0.515078	0.817863	0.315899	38.35	1.00	24.36	9.74	0.60	0.32
Q7Z612	Acidic ribosomal phosphoprotein P1	NA	2.83	-0.58054	-0.64267	-0.2142	-0.30848	-0.06293	0.03021	3.04	4.62	4.56	20.27	-0.30	0.27	-0.07096	-0.17056	-0.30958	-0.70379	-0.26606	-0.53272	4.88	19.20	13.03	13.72	-0.34	0.24
P68032	Actin, alpha cardiac muscle 1	ACTC1	27.33	NA	NA	-0.39931	-0.05084	NA	0.360638	NA	17.00	NA	27.83	-0.03	0.38	NA	NA	0.0495	0.216577	NA	0.649476	NA	8.18	NA	24.93	0.31	0.31
A0A024R694	Actinin, alpha 1, isoform CRA_a	ACTN1	13.80	1.270185	0.39116	0.626674	1.438543	0.834585	NA	41.80	38.77	NA	9.37	0.91	0.44	1.675618	0.688611	1.133795	1.308185	1.352738	NA	46.57	8.54	NA	4.60	1.23	0.36
P61160	Actin-related protein 2	ACTR2	1.50	1.017997	NA	NA	NA	-0.5041	NA	NA	NA	NA	68.37	0.26	1.08	1.141303	NA	NA	NA	0.04198	NA	NA	NA	NA	51.42	0.59	0.78
Q2LE71	Actin-related protein 2/3 complex subunit 3	ARPC3	2.00	NA	NA	0.247132	NA	1.085089	1.050958	NA	NA	1.67	39.19	0.79	0.47	NA	NA	0.094042	NA	0.858003	0.355428	NA	NA	24.39	25.90	0.44	0.39
Q9NPJ3	Acyl-coenzyme A thioesterase 13	ACOT13	1.20	-0.35609	0.27045	-0.72019	-0.86195	-0.34561	NA	30.23	6.94	NA	26.39	-0.40	0.44	-1.12489	-0.40691	-0.47102	-0.08899	-0.07861	NA	34.48	18.62	NA	21.79	-0.43	0.43
Q5VU08	Adducin 3 (Gamma), isoform CRA_a	ADD3	1.25	0.290365	NA	0.663788	NA	-1.12286	-1.44435	NA	NA	15.69	55.85	-0.40	1.04	0.62629	NA	-0.24278	NA	-0.99814	-0.84016	NA	NA	7.74	53.34	-0.36	0.74
D3DPU2	Adenylyl cyclase-associated protein	CAP1	5.83	0.132861	0.014731	-0.20043	-0.11791	-0.10245	-0.63639	5.79	4.04	25.88	14.66	-0.15	0.26	0.203995	0.010101	-0.10522	0.027	0.256922	0.034587	9.49	6.48	10.88	6.74	0.07	0.13
P12235	ADP/ATP translocase 1	SLC25A4	14.50	-0.24764	-0.59029	-2.06035	-1.51109	-1.47486	-0.79508	16.72	26.60	32.71	45.75	-1.11	0.68	-0.25221	-0.62269	-2.33107	-1.79629	-1.32027	-0.86412	18.06	25.91	22.17	51.45	-1.20	0.77
A0A024R3Q3	ADP-ribosylation factor 1, isoform CRA_a	ARF1	6.00	-0.43586	NA	0.206822	0.236432	-0.55289	-1.00538	NA	1.45	22.00	35.95	-0.31	0.53	-0.43712	NA	-0.12744	0.176053	-0.31641	-0.52192	NA	14.82	10.06	19.22	-0.25	0.28
P36405	ADP-ribosylation factor-like protein 3	ARL3	2.67	0.166224	-0.13585	NA	NA	NA	-0.40415	14.75	NA	NA	20.79	-0.12	0.29	0.711589	-0.23238	NA	NA	NA	-0.36617	44.68	NA	NA	32.80	0.04	0.59
Q02952	A-kinase anchor protein 12	AKAP12	5.83	-1.04377	-0.16514	-1.56313	-0.78589	-1.69751	-0.55068	41.78	37.20	53.43	22.50	-0.97	0.59	-0.54296	-0.41012	-0.86459	-0.27892	-1.33585	-1.19465	6.51	28.32	6.92	27.36	-0.77	0.43
O43707	Alpha-actinin-4	ACTN4	22.83	0.61939	0.72729	0.600866	0.524718	0.588709	0.307041	5.29	3.73	13.76	7.61	0.56	0.14	0.75638	0.750695	0.94059	0.993424	0.73618	0.90281	0.28	2.59	8.16	7.65	0.85	0.11
R4GMT0	Alpha-centractin	ACTR1A	3.00	-1.04824	-0.39593	0.373835	NA	-0.37467	0.004798	31.44	NA	18.49	36.31	-0.29	0.53	-0.2844	-0.65494	0.349152	NA	-0.86866	0.619689	18.06	NA	67.10	26.99	-0.17	0.64
P02489	Alpha-crystallin A chain	CRYAA	9.67	-1.10681	-1.04082	-2.43148	-2.1051	-1.53111	-1.62654	3.23	15.93	4.68	39.21	-1.64	0.55	-1.06636	-1.02826	-1.92145	-1.96239	-2.0942	-1.89325	1.87	2.01	9.83	39.66	-1.66	0.48
Q16352	Alpha-internexin	INA	4.75	-1.15334	-1.89885	-0.04441	NA	-0.89521	NA	35.75	NA	NA	50.48	-1.00	0.76	-1.06728	-0.98052	-0.57236	NA	-0.79577	NA	4.25	NA	NA	15.57	-0.85	0.22
Q5TZP7	APEX nuclease (Multifunctional DNA repair enzyme) 1	APEX1	5.80	-0.68237	-0.72939	0.529209	NA	-0.90827	-1.00737	2.30	NA	4.86	59.48	-0.56	0.62	-0.71572	-0.60851	1.016231	NA	-0.99566	-0.74165	5.25	NA	12.42	77.47	-0.41	0.81
P02649	Apolipoprotein E	APOE	2.40	NA	0.463017	-0.40071	-0.26982	-0.76788	-0.13068	NA	6.41	30.73	36.02	-0.22	0.45	NA	0.988607	1.12512	-0.52104	-0.22391	0.423736	NA	72.94	31.22	29.63	0.36	0.72
A0A024RAI1	ARP3 actin-related protein 3 homolog (Yeast), isoform CRA_a	ACTR3	3.80	0.5114	0.212651	0.379959	0.579905	NA	0.387248	14.59	9.78	NA	4.29	0.41	0.14	-0.17497	0.825219	0.219099	0.381401	NA	1.021041	47.15	7.95	NA	28.40	0.45	0.48
Q12797	Aspartyl/asparaginyl beta-hydroxylase	ASPH	4.67	-0.57878	-0.62675	-0.11098	-0.38078	-0.92013	-1.06416	2.35	13.19	7.05	25.70	-0.61	0.35	-0.05403	-0.5632	-0.43466	-0.57904	-0.36831	-0.4915	24.70	7.07	6.03	7.77	-0.42	0.19
Q08211	ATP-dependent RNA helicase A	DHX9	7.83	0.265362	0.161633	-0.15723	0.47608	-0.06293	-0.23659	5.08	30.55	8.50	13.47	0.07	0.27	0.206655	0.397523	-0.08388	-0.17869	-0.33637	-0.23793	9.34	4.65	4.82	22.03	-0.04	0.28
O43491	Band 4.1-like protein 2	EPB41L2	11.17	-0.78887	-0.84841	-0.97712	-0.9087	-1.20008	-1.30304	2.92	3.35	5.04	14.93	-1.00	0.20	-0.73128	-0.80433	-0.56187	-0.71742	-1.31409	-1.57292	3.58	7.62	12.65	27.14	-0.95	0.40
Q5SU16	Beta 5-tubulin	TUBB	51.33	0.363029	0.400538	0.546916	0.763949	-0.2626	-0.1428	1.84	10.62	5.87	28.61	0.28	0.40	0.079708	0.279032	0.698238	0.975697	0.239162	0.403484	9.75	13.56	8.05	25.25	0.45	0.33
P80723	Brain acid soluble protein 1	BASP1	8.17	-0.8852	-0.55416	-0.64573	-0.58707	-1.02119	-1.37307	16.15	2.87	17.16	19.91	-0.84	0.32	-0.77561	-0.37684	-0.19333	-0.21865	-0.8713	-1.45201	19.42	1.24	28.08	30.84	-0.65	0.49
Q05682	Caldesmon	CALD1	3.00	NA	1.915068	1.028865	0.915036	1.723032	0.822859	NA	5.58	42.74	33.53	1.28	0.50	NA	2.14645	2.897089	2.251019	2.436027	1.435657	NA	31.15	47.16	22.43	2.23	0.53
Q59EF6	Calpain 2, large [catalytic] subunit variant (Fragment)	NA	1.67	-0.16919	NA	NA	NA	-1.04551	0.789151	NA	NA	79.49	15.37	-0.14	0.92	-0.16585	NA	NA	NA	-0.4732	0.032077	NA	NA	24.51	1.60	-0.20	0.25
Q15417	Calponin-3	CNN3	2.40	-0.04661	NA	0.150863	0.426334	0.289285	0.500371	NA	13.46	10.33	15.51	0.26	0.22	1.006668	NA	0.300462	1.083107	-0.2071	0.076204	NA	37.45	13.84	34.61	0.45	0.57
V9HW88	Calreticulin, isoform CRA_b	CALR	20.17	0.842497	1.10416	0.762397	1.06445	0.940425	0.90047	12.79	14.75	1.96	2.35	0.94	0.13	0.906891	1.199803	0.707256	0.77664	0.828032	1.067036	14.31	3.40	11.69	11.01	0.91	0.19
Q53GE2	Capping protein (Actin filament) muscle Z-line, alpha 2 variant (Fragment)	NA	2.50	-0.10279	NA	0.738767	0.340868	NA	0.124625	NA	19.38	NA	23.69	0.28	0.36	0.322103	NA	0.617243	0.883503	NA	0.811951	NA	13.01	NA	17.55	0.66	0.25
B3KV96	cDNA FLJ16285 fis, clone OCBBF2004038, highly similar to Dihydropyrimidinase-related protein 1	NA	9.33	NA	-1.24613	NA	-1.33118	-1.71805	NA	NA	NA	NA	16.60	-1.43	0.25	NA	-1.67591	NA	-1.19333	-1.2876	NA	NA	NA	NA	16.88	-1.39	0.26
B4DHK9	cDNA FLJ51656, highly similar to Actin-like protein 2	NA	1.00	NA	1.047336	NA	0.892775	NA	NA	NA	NA	NA	7.57	0.97	0.11	NA	0.825343	NA	1.446643	NA	NA	NA	NA	NA	29.99	1.14	0.44
B4E2G8	cDNA FLJ54047, highly similar to Alpha-1 catenin (Cadherin-associated protein)	NA	6.17	0.199175	1.064955	0.274562	0.903666	0.478451	0.196809	41.21	30.35	13.76	12.44	0.52	0.38	-1.07142	0.494089	0.656625	0.445809	-0.67894	0.041468	69.99	10.31	34.59	31.73	-0.02	0.70
B4E192	cDNA FLJ54224, highly similar to Glial fibrillary acidic protein, astrocyte	NA	15.00	-0.7299	-1.0776	-1.51992	NA	NA	NA	16.96	NA	NA	30.24	-1.11	0.40	-1.16757	-0.98968	-1.1189	NA	NA	NA	8.71	NA	NA	2.11	-1.09	0.09
B7Z6N2	cDNA FLJ56154, highly similar to Gelsolin	NA	12.25	NA	0.536315	0.308132	-0.11648	NA	0.117607	NA	20.66	NA	17.61	0.21	0.28	NA	0.554753	0.294284	0.16956	NA	0.435499	NA	6.11	NA	11.09	0.36	0.17
A8K0T9	cDNA FLJ75422, highly similar to Homo sapiens capping protein (actin filament) muscle Z-line, alpha 1, mRI	NA	2.00	0.733418	-0.00323	NA	0.770145	0.519695	-1.04071	35.34	NA	69.80	27.99	0.20	0.76	0.660102	0.064006	NA	1.605508	0.292812	-0.0638	28.81	NA	17.39	58.87	0.51	0.67
A8K7N0	cDNA FLJ75556, highly similar to Homo sapiens ribosomal protein L14, mRNA	NA	1.50	-0.62265	-1.92961	NA	NA	0.293625	-0.11055	60.01	NA	19.68	57.23	-0.59	0.97	-1.13978	-0.66016	NA	NA	0.31874	0.032077	23.29	NA	14.00	49.85	-0.36	0.66
B2R7T8	cDNA, FLJ93598, highly similar to Homo sapiens capping protein (actin filament) muscle Z-line, beta (CAPZI	NA	5.50	0.025366	-0.17539	0.253372	0.235761	-0.34596	-0.46957	9.82	0.86	6.05	22.35	-0.08	0.30	0.296819	-0.16561	0.757818	0.397008	0.129455	-0.14575	22.47	17.59	13.45	23.21	0.21	0.35
B2RA03	cDNA, FLJ94640, highly similar to Homo sapiens keratin 18 (KRT18), mRNA	NA	9.50	NA	NA	NA	1.59447	NA	1.263141	NA	NA	NA	16.17	1.43	0.23	NA	NA	NA	2.047228	NA	1.128552	NA	NA	NA	43.56	1.59	0.65
B2RBD5	cDNA, FLJ95457, highly similar to Homo sapiens tubulin, beta, 4 (TUBB4), mRNA	NA	37.50	-0.51325	-0.74426	-1.6968	NA	0.588895	NA	11.30	NA	NA	75.08	-0.59	0.94	-1.00044	-1.3043	-0.71545	NA	-0.2021	NA	14.84	NA	NA	32.72	-0.81	0.47
P41208	Centrin-2	CETN2	1.25	-0.96596	-0.40696	-0.42826	NA	0.211498	NA	27.06	NA	NA	32.76	-0.40	0.48	0.234487	-0.09189	0.537021	NA	0.438166	NA	15.93	NA	NA	15.93	0.28	0.28
A0A087WVQ6	Clathrin heavy chain	CLTC	44.33	0.064858	-0.04277	0.13719	0.176111	-0.38431	-0.17786	5.27	1.91	10.10	14.91	-0.04	0.21	0.015474	0.037198	0.098067	-0.01854	-0.2174	-0.06268	1.06	5.71	7.58	6.75	-0.02	0.11
P09497	Clathrin light chain B	CLTB	3.83	0.059012	0.058043	0.536354	0.513496	0.309955	0.572377	0.05	1.12	12.83	16.48	0.34	0.24	-0.08507	0.154887	0.919606	0.506577	0.043786	0.199533	11.73	20.11	7.63	27.52	0.29	0.37
P53621	Coatomer subunit alpha	COPA	2.25	1.040852	1.670399	1.419834	NA	NA	0.864051	30.38	NA	NA	20.18	1.25	0.36	0.58072	0.932214	1.434674	NA	NA	0.757775	17.14	NA	NA	28.61	0.93	0.37
P53618	Coatomer subunit beta	COPB1	1.25	NA	NA	-0.27672	0.76736	0.195474	0.538333	NA	49.05	16.73	1.93	0.31	0.45	NA	NA	-0.68015	0.76644	0.137936	-0.06559	NA	65.50	9.96	8.69	0.04	0.60
Q549N0	Cofilin 2 (Muscle), isoform CRA_a	CFL2	4.75	1.088192	NA	0.877237	-0.12854	0.838421	NA	NA	47.39	NA	21.31	0.67	0.54	0.417719	NA	0.553151	0.174488	0.302736	NA	NA	18.45	NA	4.01	0.36	0.16
E9PK25	Cofilin-1	CFL1	10.67	0.531151	0.404129	0.130668	0.33168	0.429676	0.328922	6.22	9.84	4.94	8.12	0.36	0.13	0.541814	0.649952	0.795776	0.707855	0.441215	0.31779	5.30	4.31	6.05	12.78	0.58	0.18
X5DQV1	Collapsin response mediator protein 1 isoform B (Fragment)	CRMP1	10.67	-0.99626	NA	-0.73511	NA	NA	-1.26824	NA	NA	NA	18.37	-1.00	0.27	-1.59067	NA	-0.80192	NA	NA	-1.2701	NA	NA	NA	27.90	-1.22	0.40
V9HW27	Crystallin, alpha B, isoform CRA_a	CRYAB	7.50	0.659075	0.395929	0.143683	0.24313	1.218964	1.084734	12.86	4.87	6.57	34.71	0.62	0.45	0.885226	0.716504	0.451519	0.689039	1.140096	1.155232	8.26	11.62	0.74	20.26	0.84	0.28
Q14204	Cytoplasmic dynein 1 heavy chain 1	DYNC1H1	19.83	-0.0077	0.604321	-0.38124	1.450071	0.06592	0.415921	29.55	79.38	17.07	21.84	0.36	0.64	-0.00791	0.922597	-0.80192	1.248047	0.365554	0.335884	44.09	86.40	1.45	7.69	0.34	0.72
Q59GB4	Dihydropyrimidinase-like 2 variant (Fragment)	NA	19.83	-1.59266	-1.43594	-0.7722	-1.66226	-1.30083	-1.27781	7.67	42.29	1.13	12.47	-1.34	0.32	-1.86138	-1.82565	-0.79928	-1.4049	-0.87547	-0.99072	1.75	29.25	5.65	30.52	-1.29	0.48
V9HW53	Dimethylarginine dimethylaminohydrolase 2, isoform CRA_a	DDAH2	3.50	-0.13183	-0.13148	0.341567	-0.78	-0.16393	-0.05811	0.02	52.36	5.18	0.81	-0.15	0.36	0.309782	-0.26749	0.418197	0.239202	0.059746	-0.45289	27.92	8.76	24.86	17.66	0.05	0.34
Q6DEN2	DPYSL3 protein	DPYSL3	21.00	-0.99195	-0.54555	-1.0466	-1.27141	-1.03259	-1.23393	21.71	11.00	9.85	16.28	-1.02	0.26	-1.15874	-0.63427	-1.04081	-0.9917	-1.1075	-1.43281	25.43	2.41	15.88	13.34	-1.06	0.26
E7EX90	Dynactin subunit 1	DCTN1	2.00	0.301908	NA	NA	NA	NA	-0.27746	NA	NA	NA	28.02	0.01	0.41	0.226831	NA	NA	NA	NA	0.062738	NA	NA	NA	8.03	0.14	0.12
Q96FJ2	Dynein light chain 2, cytoplasmic	DYNLL2	3.00	-0.73677	-1.00533	-0.56391	-0.51636	-0.11557	-0.19216	13.12	2.33	3.75	24.76	-0.52	0.33	-0.31134	-0.8327	-0.86736	-0.48139	-0.55377	-0.48609	25.28	18.81	3.32	5.13	-0.59	0.22
Q59GU5	Dynein, cytoplasmic, intermediate polypeptide 2 variant (Fragment)	NA	1.00	NA	NA	0.050526	NA	0.29237	NA	NA	NA	NA	11.83	0.17	0.17	NA	NA	-0.161	NA	0.222777	NA	NA	NA	NA	18.70	0.03	0.27
B2ZZ89	Epididymis luminal protein 102	SPTBN1	26.83	-0.44039	-0.57324	-0.42612	-0.23125	-0.42897	-0.41994	6.51	9.54	0.44	6.26	-0.42	0.11	-0.51045	-0.62235	-0.50258	-0.4874	-0.57795	-0.68273	5.48	0.74	5.13	4.66	-0.56	0.08
V9HWE1	Epididymis luminal protein 113	VIM	93.50	0.075358	0.080658	0.428821	0.443495	-0.37976	-0.42696	0.26	0.72	2.31	28.29	0.04	0.38	0.607142	0.598488	1.214526	1.275097	0.497956	0.496259	0.42	2.97	0.08	29.74	0.78	0.36
V9HWK2	Epididymis luminal protein 114	VCL	11.00	1.083277	0.964739	0.711023	0.224974	0.601848	0.202839	5.81	23.60	19.43	24.29	0.63	0.37	0.967648	0.97129	0.83815	0.387008	0.918219	0.595096	0.18	21.93	15.77	11.95	0.78	0.24
V9HW98	Epididymis luminal protein 2	YWHAE	18.67	-0.21164	-0.2477	0.358618	0.147836	-0.43028	-0.534	1.77	10.31	5.08	26.76	-0.15	0.34	-0.29109	-0.3092	0.007824	-0.21016	-0.10372	-0.24045	0.89	10.66	6.70	7.06	-0.19	0.12
V9HWA6	Epididymis luminal protein 32	DSTN	3.50	0.607166	0.98291	-0.66493	0.007236	-0.70729	-0.03867	18.31	32.36	32.20	48.69	0.03	0.67	0.044235	1.754226	-0.46333	0.236634	-0.32934	0.077422	75.21	33.65	19.81	53.66	0.22	0.80
V9HWC0	Epididymis luminal protein 70	MSN	14.83	0.895309	0.600821	0.338635	0.331471	0.911882	0.149557	14.38	0.35	36.52	14.43	0.54	0.32	0.627709	0.695022	0.472645	0.588743	0.71857	0.314867	3.30	5.69	19.66	5.30	0.57	0.15
V9HWG7	Epididymis secretory protein Li 52	WDR1	7.00	NA	NA	-0.01921	-0.13258	0.597328	NA	NA	5.55	NA	32.36	0.15	0.39	NA	NA	0.034506	-0.03215	0.781995	NA	NA	3.27	NA	37.35	0.26	0.45
V9HW58	Epididymis secretory protein Li 77	DCTN2	4.20	0.239584	0.346322	0.111169	-0.64484	-0.46691	NA	5.23	36.23	NA	27.75	-0.08	0.44	-0.18515	-0.26882	-0.22729	-0.14481	-0.40645	NA	4.10	4.04	NA	7.98	-0.25	0.10
P58107	Epiplakin	EPPK1	3.50	NA	NA	1.666462	NA	NA	0.882184	NA	NA	NA	37.52	1.27	0.55	NA	NA	2.196365	NA	NA	0.914897	NA	NA	NA	58.98	1.56	0.91
E7EQR4	Ezrin	EZR	23.17	1.584306	1.908408	1.192397	1.184951	1.559935	1.022925	15.82	0.36	26.02	21.44	1.41	0.33	1.508392	1.967914	1.382341	1.497579	1.806084	1.332742	22.33	5.65	22.99	10.95	1.58	0.25
Q16658	Fascin	FSCN1	2.75	0.970167	NA	-0.15663	0.064324	-0.22225	NA	NA	10.81	NA	47.99	0.16	0.55	0.165243	NA	0.308862	-0.20056	0.502488	NA	NA	24.71	NA	16.09	0.19	0.30
B3KTA3	Fascin	NA	3.00	NA	0.114893	NA	NA	NA	-0.50183	NA	NA	NA	29.78	-0.19	0.44	NA	-0.33538	NA	NA	NA	0.021991	NA	NA	NA	17.43	-0.16	0.25

UniProt	Protein name	Gene name	Peptide mean	08/017 hESC-RPE to hRPE: Analysis 1.1	08/017 Analysis 1.2	08/017 Analysis 2.1	08/017 Analysis 2.2	08/017 Analysis 3.1	08/017 Analysis 3.2	08/017 Technical replicate CV 1	08/017 Technical replicate CV 2	08/017 Technical replicate CV 3	08/017 Biological CV	08/017 Fold change (mean)	08/017 StDev	08/023 hESC-RPE to hRPE: Analysis 1.1	08/023 Analysis 1.2	08/023 Analysis 2.1	08/023 Analysis 2.2	08/023 Analysis 3.1	08/023 Analysis 3.2	08/023 Technical replicate CV 1	08/023 Technical replicate CV 2	08/023 Technical replicate CV 3	08/023 Biological CV	08/023 Fold change (mean)	08/023 StDev
P35555	Fibrillin-1	FBN1	25.83	1.607747	0.988756	1.317947	1.315035	1.849868	2.22429	29.88	0.14	18.25	30.98	1.55	0.44	2.635805	2.18955	2.497953	2.681427	1.935242	2.457065	21.70	8.98	25.30	12.83	2.40	0.29
A0A087WYV8	Fibrillin-2	FBN2	2.33	NA	NA	1.394953	NA	0.369381	1.421472	NA	NA	49.40	19.75	1.06	0.60	NA	NA	2.219534	NA	0.574239	1.367344	NA	NA	37.92	55.43	1.39	0.82
P21333	Filamin-A	FLNA	38.83	1.279703	1.27822	1.027436	0.960179	0.79485	1.030971	0.07	3.30	11.55	13.57	1.06	0.19	1.270175	1.293218	1.450871	1.393843	1.132476	1.327719	1.13	2.79	9.55	6.91	1.31	0.11
O75369	Filamin-B	FLNB	9.67	0.744152	0.851052	0.808226	0.62111	0.276531	0.217864	5.24	9.16	2.88	19.38	0.59	0.27	1.025089	0.886578	0.87601	0.804938	0.521196	0.416215	6.78	3.48	5.14	16.89	0.76	0.24
Q14315	Filamin-C	FLNC	5.50	NA	NA	NA	-0.06815	NA	0.737786	NA	NA	NA	38.50	0.33	0.57	NA	NA	NA	0.450249	NA	0.636313	NA	NA	NA	9.11	0.54	0.13
Q5QTS3	FWP004	NA	2.20	0.230553	0.073099	NA	0.252676	0.288827	0.590042	7.71	NA	14.71	10.49	0.29	0.19	0.615604	-0.05457	NA	0.857415	0.04499	0.531346	32.27	NA	23.61	22.92	0.40	0.39
G3V4P8	Glia maturation factor beta (Fragment)	GMFB	1.00	1.044102	NA	0.758172	NA	NA	NA	NA	NA	NA	13.97	0.90	0.20	0.615461	NA	0.32208	NA	NA	NA	NA	NA	NA	14.33	0.47	0.21
P14136	Glial fibrillary acidic protein	GFAP	12.50	NA	NA	NA	-1.46451	NA	-0.50684	NA	NA	NA	45.29	-0.99	0.68	NA	NA	NA	-1.62929	NA	-0.09479	NA	NA	NA	68.84	-0.86	1.09
P04899	Guanine nucleotide-binding protein G(i) subunit alpha-2	GNAI2	6.80	0.539572	0.43619	0.323253	NA	-0.70068	0.159588	5.06	NA	40.96	23.59	0.15	0.50	0.32805	0.285916	0.015355	NA	-0.08189	0.981035	2.06	NA	49.86	18.15	0.31	0.42
B2R4R9	HCG26477	RPS28	2.00	-1.2535	-1.35359	-0.16642	0.402997	-0.38723	NA	4.90	27.55	NA	46.21	-0.55	0.75	-0.64241	-0.72315	0.777586	0.877471	0.2346	NA	3.96	4.89	NA	48.36	0.10	0.76
K7EP04	Heat shock protein beta-6	HSPB6	1.00	NA	NA	1.585581	NA	0.536273	NA	NA	NA	NA	49.28	1.06	0.74	NA	NA	0.886999	NA	-0.51513	NA	NA	NA	NA	63.78	0.19	0.99
Q7Z4W8	Heparin-binding protein HBp15	NA	1.33	-2.23022	-1.77544	-0.44116	0.784756	-0.71668	-1.56587	22.11	56.71	40.46	78.62	-0.99	1.10	0.085089	-1.8001	0.28953	0.464584	0.366677	0.207528	81.17	8.57	7.79	32.05	-0.06	0.86
P49773	Histidine triad nucleotide-binding protein 1	HINT1	2.33	0.513608	0.63496	0.498019	0.510908	0.609656	0.901908	5.94	0.63	14.28	9.42	0.61	0.15	0.564365	0.715162	0.550561	0.915295	0.397929	0.814022	7.38	17.78	20.25	4.64	0.66	0.19
P07305	Histone H1.0	H1F0	2.17	-2.02482	-2.14108	-0.44693	-0.69816	-1.0726	-1.12142	5.70	12.28	2.39	47.76	-1.25	0.69	-1.58474	-1.29022	-0.69564	-0.69477	-1.5761	-1.44209	14.39	0.04	6.56	33.18	-1.21	0.42
P05556	Integrin beta-1	ITGB1	2.33	0.149462	-0.28122	0.62839	0.613721	0.735134	0.260371	20.95	0.72	23.06	23.18	0.35	0.39	-0.25717	-0.68085	0.93993	0.767182	0.983781	0.313317	20.62	8.46	32.28	41.53	0.34	0.69
Q17R60	Interphotoreceptor matrix proteoglycan 1	IMPG1	4.80	-0.69379	-0.74267	-2.21543	-1.85193	-1.19271	NA	2.40	17.72	NA	42.04	-1.34	0.68	-0.92778	-1.02736	-2.11286	-1.89608	-0.84878	NA	4.88	10.61	NA	37.55	-1.36	0.59
H6VRG3	Keratin 1	KRT1	10.00	1.715608	1.313017	0.771898	0.149976	0.42978	0.164514	19.61	30.02	12.96	49.22	0.76	0.64	1.502371	0.944861	0.32127	-0.1727	0.436944	0.774393	26.99	23.98	16.46	40.04	0.63	0.58
P13645	Keratin, type I cytoskeletal 10	KRT10	7.25	1.740799	1.755394	1.072748	0.375217	NA	NA	0.72	33.54	NA	46.37	1.24	0.66	1.111899	1.732706	0.996453	0.323492	NA	NA	29.97	32.40	NA	36.26	1.04	0.58
F8VZY9	Keratin, type I cytoskeletal 18	KRT18	6.25	1.55097	1.488204	1.219056	NA	0.698563	NA	3.08	NA	NA	27.47	1.24	0.39	1.284368	1.255689	1.182774	NA	1.285281	NA	1.41	NA	NA	3.79	1.25	0.05
P08727	Keratin, type I cytoskeletal 19	KRT19	7.67	NA	NA	0.744948	0.760374	0.87501	NA	NA	0.76	NA	5.99	0.79	0.07	NA	NA	1.754672	1.446921	1.08121	NA	NA	15.03	NA	25.58	1.43	0.34
P05787	Keratin, type II cytoskeletal 8	KRT8	17.50	1.785737	1.388262	1.527923	1.812191	1.052638	1.506304	19.36	13.89	22.05	13.42	1.51	0.28	1.86948	1.38205	1.650753	1.886236	0.998759	1.810548	23.67	11.52	38.77	10.76	1.60	0.35
J9JID7	Lamin B2, isoform CRA_a	LMNB2	13.67	-0.50932	-0.53506	-0.10327	0.019772	-1.05943	-0.35669	1.26	6.03	33.78	23.67	-0.42	0.38	-0.68123	-0.40166	-0.19449	-0.00126	-0.59128	-0.18488	13.66	9.46	19.79	15.69	-0.34	0.26
P20700	Lamin-B1	LMNB1	10.67	-0.35836	-0.17389	0.392936	0.358453	-0.75509	0.954853	9.03	1.69	75.20	22.90	0.07	0.62	-0.46588	-0.07068	-0.23145	0.106735	-0.98376	-0.19245	19.25	16.50	37.84	16.50	-0.31	0.38
V9HWF2	Malate dehydrogenase	MDH1	8.83	-0.59177	-0.42545	-0.51272	-0.20123	-0.67167	-0.44262	8.14	15.21	11.20	7.53	-0.47	0.16	-0.75771	-0.56711	-0.45671	-0.32907	-0.72059	-0.46791	9.33	6.25	12.35	9.80	-0.55	0.17
A0A024R407	Microtubule-associated protein	MAP2	5.00	-1.06791	-0.55947	0.639152	0.50609	-0.70364	-0.26858	24.67	6.52	21.16	52.68	-0.24	0.68	-0.99694	-0.46899	1.229713	0.158072	-0.87244	-0.23641	25.59	50.24	30.68	61.45	-0.20	0.82
E7EVA0	Microtubule-associated protein	MAP4	2.83	-0.47445	-0.1628	-0.27929	-0.77819	0.270633	-1.40116	15.22	24.21	73.85	7.28	-0.47	0.57	-0.37897	-0.28941	0.057304	-1.17498	0.258305	-0.42216	4.39	56.98	32.75	14.40	-0.32	0.49
A0A024RAM4	Microtubule-associated protein 1B, isoform CRA_b	MAP1B	14.67	-0.24042	-0.32074	-0.79134	-0.53725	-0.14541	-0.4207	3.94	12.42	13.45	14.50	-0.41	0.23	-0.44068	-0.65599	-0.79532	-0.8095	-0.18978	-0.15478	10.53	0.69	1.72	22.24	-0.51	0.29
Q9H936	Mitochondrial glutamate carrier 1	SLC25A22	3.00	NA	-1.9095	-0.31446	-0.55883	NA	NA	NA	11.95	NA	66.71	-0.93	0.86	NA	-1.69078	-1.14105	-0.60453	NA	NA	NA	26.00	NA	40.17	-1.15	0.54
Q1HBJ4	Mitogen-activated protein kinase	MAPK1	2.75	NA	NA	0.147517	-0.26823	-1.00933	-0.71193	NA	20.24	14.52	38.58	-0.46	0.51	NA	NA	-0.25904	-0.13121	-0.91625	-0.41073	NA	6.26	24.53	21.77	-0.43	0.34
P25189	Myelin protein P0	MPZ	3.00	-0.6887	-1.00038	NA	NA	-2.52587	-1.89535	15.22	NA	30.42	61.34	-1.53	0.84	-0.70104	-1.13359	NA	NA	-2.66117	-1.93767	21.04	NA	34.74	61.86	-1.61	0.87
J3QRS3	Myosin regulatory light chain 12A	MYL12A	7.33	0.364712	0.582429	0.194135	0.322855	-0.05797	0.722278	10.65	6.30	37.34	7.53	0.35	0.28	0.701788	0.784989	0.803627	0.912616	0.740889	1.324303	4.08	5.34	28.21	11.30	0.88	0.23
A0A024R1N1	Myosin, heavy polypeptide 9, non-muscle, isoform CRA_a	MYH9	23.00	0.80772	1.391883	1.00278	0.681532	0.289513	0.159967	28.25	15.68	6.35	29.87	0.72	0.46	0.716398	1.418696	1.524849	1.114448	1.249635	0.980167	33.76	19.98	13.17	8.97	1.17	0.30
P35580	Myosin-10	MYH10	10.17	1.357816	1.470366	0.552529	0.574781	0.442283	0.37564	5.51	1.09	3.27	40.21	0.80	0.49	1.376116	1.211269	0.285798	1.163715	0.740394	0.386223	8.07	41.75	17.27	26.61	0.86	0.46
Q7Z406	Myosin-14	MYH14	5.50	NA	0.143703	NA	NA	0.929604	NA	NA	NA	NA	37.59	0.54	0.56	NA	0.33653	NA	NA	0.751368	NA	NA	NA	NA	20.19	0.54	0.29
O14745	Na(+)/H(+) exchange regulatory cofactor NHE-RF1	SLC9A3R1	8.67	1.385279	1.86852	1.280794	1.40678	1.46048	1.42337	23.47	6.17	1.82	10.85	1.47	0.20	1.407596	1.91543	1.841261	1.905157	1.435722	1.267986	24.64	3.13	8.21	17.71	1.63	0.29
Q09666	Neuroblast differentiation-associated protein AHNAK	AHNAK	21.50	-0.04722	-0.2145	-0.17597	-0.14069	-0.65617	-0.52056	8.19	1.73	6.64	16.80	-0.29	0.24	0.058403	-0.11892	0.66734	0.7961	-0.07927	-0.08045	8.68	6.31	0.06	33.74	0.21	0.41
Q9UNZ2	NSFL1 cofactor p47	NSFL1C	9.50	0.714109	1.293634	NA	NA	1.21656	1.434498	28.03	NA	10.66	14.50	1.16	0.31	0.953235	1.465967	NA	NA	0.800515	1.123844	24.87	NA	15.78	12.76	1.09	0.29
P06748	Nucleophosmin	NPM1	7.67	0.599781	0.343483	1.322935	0.611135	0.635892	0.643133	12.53	34.20	0.35	19.48	0.69	0.33	0.658437	0.706411	1.048766	0.292303	0.114252	0.503375	2.35	36.25	18.96	14.53	0.55	0.33
P15531	Nucleoside diphosphate kinase A	NME1	7.00	-0.33409	-1.45872	-0.21266	-0.67651	NA	-1.90537	52.49	22.54	NA	45.73	-0.92	0.74	-0.22916	-1.713	-0.051	-0.08337	NA	-0.9675	66.93	1.59	NA	35.02	-0.61	0.72
J3KQ32	Obg-like ATPase 1	OLA1	2.20	0.647309	0.757023	0.598417	0.742679	-0.01468	NA	5.37	7.06	NA	25.57	0.55	0.32	-0.19967	0.423387	-0.05572	0.645501	0.06608	NA	30.07	33.71	NA	9.82	0.18	0.35
Q02790	Peptidyl-prolyl cis-trans isomerase FKBP4	FKBP4	1.50	-0.34955	NA	NA	0.147122	NA	NA	NA	NA	NA	24.11	-0.10	0.35	-0.64686	NA	NA	0.67662	NA	NA	NA	NA	NA	60.67	0.01	0.94
P36871	Phosphoglucomutase-1	PGM1	5.50	0.178973	-0.05022	NA	NA	0.298634	-0.11162	11.21	NA	19.97	1.92	0.08	0.19	0.286436	0.096933	NA	NA	-0.02461	0.093966	9.27	NA	5.81	7.78	0.11	0.13
Q15149	Plectin	PLEC	25.83	0.608716	0.748574	0.569239	0.913077	0.521546	0.599028	6.85	16.77	3.80	6.62	0.66	0.15	0.418539	0.92492	0.79315	1.175829	0.677529	0.8457	24.57	18.65	8.23	11.26	0.81	0.25
Q96AQ6	Pre-B-cell leukemia transcription factor-interacting protein 1	PBXIP1	2.20	0.464237	0.430708	NA	0.530752	-0.33234	-0.1618	1.64	NA	8.35	26.77	0.19	0.40	0.345919	0.950117	NA	-0.00354	-0.31127	0.223203	29.19	NA	25.90	29.44	0.24	0.47
P02545	Prelamin-A/C	LMNA	20.50	0.108054	0.455838	0.610099	0.542927	-0.31607	-0.28296	16.96	3.29	1.62	29.08	0.19	0.41	0.028853	0.188148	0.289323	0.415668	-0.19664	-0.05199	7.80	6.19	7.08	16.48	0.11	0.23
P07737	Profilin-1	PFN1	10.00	0.757371	0.545318	0.405597	0.595168	1.437388	0.893868	10.37	9.28	26.33	26.22	0.77	0.37	0.731521	0.68738	1.107846	1.253985	1.554453	0.934521	2.16	7.16	29.93	19.82	1.04	0.33
P35080	Profilin-2	PFN2	3.60	-1.14243	-0.32122	NA	0.703877	-0.08619	-0.0817	39.20	NA	0.22	48.04	-0.19	0.66	-1.14462	-0.63257	NA	1.271338	-0.18554	-0.61099	24.84	NA	20.70	82.02	-0.26	0.92
Q07954	Prolow-density lipoprotein receptor-related protein 1	LRP1	2.50	0.663307	0.675935	-0.04332	0.42437	0.532895	0.159588	0.62	22.72	18.20	16.65	0.40	0.29	0.696389	0.197595	-0.02888	-0.30674	0.982944	0.239434	24.21	13.58	35.66	27.42	0.30	0.47
P49720	Proteasome subunit beta type-3	PSMB3	2.00	-0.67418	NA	-0.58205	-0.64319	NA	NA	NA	3.00	NA	3.03	-0.63	0.05	-0.51013	NA	-0.27273	-0.13647	NA	NA	NA	6.67	NA	15.00	-0.31	0.19
P11171	Protein 4.1	EPB41	2.25	-0.1897	NA	NA	-0.13576	-0.22683	-0.63157	NA	NA	19.71	9.99	-0.30	0.23	-1.25168	NA	NA	-0.75696	-0.80477	-0.81095	NA	NA	0.30	17.78	-0.91	0.23
Q5TZC3	Protein kinase C and casein kinase substrate in neurons 1, isoform CRA_a	PACSIN1	1.33	-0.91793	0.508962	NA	NA	NA	-0.83284	64.74	NA	NA	38.15	-0.41	0.80	-0.81554	-0.27787	NA	NA	NA	-1.38729	26.05	NA	NA	41.19	-0.83	0.55
S4R435	Protein RPS10-NUDT3 (Fragment)	RPS10-NUDT3	1.33	0.294865	-0.26265	0.245568	NA	NA	NA	26.99	NA	NA	9.92	0.09	0.31	0.047843	0.526424	0.091432	NA	NA	NA	23.24	NA	NA	10.54	0.22	0.26
P05109	Protein S100-A8	S100A8	1.33	-0.73218	-0.64801	-1.5924	-1.54839	-2.52185	-2.3978	4.12	2.16	6.08	58.53	-1.57	0.79	-0.49832	0.144159	-1.4136	-1.15237	-2.75298	-2.29615	30.98	12.77	22.21	74.79	-1.33	1.08
Q6MZZ3	Putative uncharacterized protein DKFZp686I0746	DKFZp686I0746	2.50	NA	-1.06762	-0.34141	NA	NA	NA	NA	NA	NA	34.86	-0.70	0.51	NA	-0.18953	-0.66162	NA	NA	NA	NA	NA	NA	22.93	-0.43	0.33
B0YJ88	Radixin	RDX	8.83	1.416955	1.04421	0.473412	0.924095	0.472116	-0.26858	18.17	21.91	35.53	37.00	0.68	0.59	1.898863	1.054807	0.92428	1.086201	-0.06838	0.209196	40.23	7.93	13.56	46.46	0.85	0.70
Q15907	Ras-related protein Rab-11B	RAB11B	6.50	0.185912	0.111031	0.180167	0.198569	NA	NA	3.67	0.90	NA	1.98	0.17	0.04	0.155439	0.237206	0.011092	0.058116	NA	NA	4.01	2.30	NA	7.94	0.12	0.10
P11233	Ras-related protein Ral-A	RALA	2.00	NA	-0.16919	-0.56195	NA	NA	NA	NA	NA	NA	19.13	-0.37	0.28	NA	-0.26273	-0.61088	NA	NA	NA	NA	NA	NA	16.98	-0.44	0.25
Q5VVD0	Ribosomal protein L11, isoform CRA_b	RPL11	1.50	0.39377	NA	0.366863	NA	0.845051	1.135344	NA	NA	14.18	26.18	0.69	0.37	0.596602	NA	0.20028	NA	0.226846	0.591123	NA	NA	17.76	13.63	0.40	0.22
E7EQV9	Ribosomal protein L15 (Fragment)	RPL15	1.50	NA	NA	1.004499	NA	0.774238	NA	NA	NA	NA	11.26	0.89	0.16	NA	NA	1.044601	NA	0.232841	NA	NA	NA	NA	38.77	0.64	0.57
A0A024RDH8	Ribosomal protein L34, isoform CRA_a	RPL34	1.40	0.644793	0.489354	0.799115	NA	1.01937	1.727623	7.61	NA	34.03	31.76	0.94	0.48	0.154849	0.674459	0.795339	NA	0.633289	0.913988	25.20	NA	13.71	13.41	0.63	0.29
A0A024R8P8	Ribosomal protein L38, isoform CRA_a	RPL38	1.25	-0.47663	-0.16791	NA	0.100181	0.113342	NA	15.07	NA	NA	15.96	-0.11	0.28	-0.29646	-0.00221	NA	0.424344	-0.34003	NA	14.37	NA	NA	28.73	-0.05	0.35
Q59GY2	Ribosomal protein L4 variant (Fragment)	NA	1.80	NA	0.164177	1.452257	0.544735	1.488051	0.977209	NA	43.07	24.78	35.52	0.93	0.57	NA	0.353704	1.118375	0.855046	0.605834	0.183975	NA	12.87	20.53	25.92	0.62	0.38
Q59GX9	Ribosomal protein L5 variant (Fragment)	NA	1.50	NA	0.489354	NA	0.916154	NA	NA	NA	NA	NA	20.77	0.70	0.30	NA	0.63044	NA	1.53933	NA	NA	NA	NA	NA	43.13	1.08	0.64
B3KTM6	Ribosomal protein L5, isoform CRA_b	RPL5	2.75	0.540925	NA	-0.02837	NA	0.515496	0.688651	NA	NA	8.48	22.35	0.43	0.31	0.536839	NA	1.071221	NA	0.363787	0.481307	NA	NA	5.76	25.18	0.61	0.31
Q8WVX7	Ribosomal protein S19 (Fragment)	NA	4.00	1.042478	0.342435	0.676282	0.557868	0.592608	0.562171	33.65	5.80	1.49	5.71	0.63	0.23	1.152781	0.440538	0.717604	0.43409	0.468371	0.603998	34.22	13.85	6.64	11.62	0.64	0.28
Q6IPX5	Ribosomal protein S2	RPS2	3.75	NA	NA	0.185304	-0.00938	0.236275	0.733627	NA	9.53	24.14	20.20	0.29	0.32	NA	NA	0.02906	0.114681	0.123962	0.528974	NA	4.20	19.72	13.10	0.20	0.22
Q53G25	Ribosomal protein S5 variant (Fragment)	NA	3.33	0.343925	0.285169	0.834921	0.460454	-0.03316	-0.05414	2.88	18.25	1.03	24.15	0.31	0.33	0.339352	0.157853	1.344874	0.400071	0.581581	0.847485	8.88	44.72	13.00	23.48	0.61	0.43
A0A024RCA7	Ribosomal protein, large, P2, isoform CRA_a	RPLP2	4.83	0.636966	0.39665	0.339055	0.533851	0.866831	0.756646	11.75	9.53	5.40	13.98	0.59	0.20	0.436137	0.536486	0.858391	0.516843	0.46403	0.798104	4.92	16.66	16.30	7.43	0.60	0.18
Q5TZA2	Rootletin	CROCC	10.80	0.262247	0.549085	-0.47097	-1.16827	NA	-0.36213	14.01	33.53	NA	43.24	-0.24	0.67	-0.00637	0.347483	-0.33585	-1.1202	NA	0.202141	17.26	37.52	NA	30.72	-0.18	0.58
Q6IAX2	RPL21 protein	RPL21	1.00	NA	-0.40847	NA	NA	1.163691	0.464972	NA	NA	33.59	58.30	0.41	0.79	NA	0.605167	NA	NA	0.691809	-0.19759	NA	NA	42.26	14.19	0.37	0.49
Q96IR1	RPS4X protein (Fragment)	RPS4X	1.80	0	NA	0.068078	-0.027	0.033644	0.645484	NA	4.66	29.55	15.01	0.14	0.28	0.326653	NA	0.337383	0.014563	0.23776	0.105951	NA	15.76	6.46	6.01	0.20	0.14
B5MCX3	Septin-2	SEPT2	3.83	-0.07039	-0.21605	0.144642	-0.31044	-0.36312	-0.00723	7.13	22.12	17.36	3.91	-0.14	0.19	-0.08646	-0.21131	-0.1025	-0.35563	-0.38118	0.049145	6.12	12.37	20.94	2.97	-0.18	0.17
E7EPK1	Septin-7	SEPT7	2.50	-0.47581	NA	NA	-0.3375	-0.0905	-0.0987	NA	NA	0.40	13.58	-0.25	0.19	-0.87498	NA	NA	-0.19622	-0.58166	-0.01431	NA	NA	27.45	23.74	-0.42	0.39
V9HW06	Serine hydroxymethyltransferase	SHMT2	1.33	NA	-1.79573	NA	0.26106	-0.7931	NA	NA	NA	NA	67.63	-0.78	1.03	NA	-2.40282	NA	0.276347	-0.73531	NA	NA	NA	NA	77.10	-0.95	1.35
Q15257	Serine/threonine-protein phosphatase 2A activator	PPP2R4	2.00	NA	-1.6915	-0.8196	-0.87543	NA	NA	NA	2.74	NA	40.24	-1.13	0.49	NA	-1.00243	-0.94611	-1.17669	NA	NA	NA	11.28	NA	2.66	-1.04	0.12
Q9BS10	Similar to ribosomal protein S8 (Fragment)	NA	1.50	NA	1.916326	NA	NA	NA	0.081465	NA	NA	NA	79.49	1.00	1.30	NA	2.733932	NA	NA	NA	0.465579	NA	NA	NA	92.80	1.60	1.60
Q13813	Spectrin alpha chain, non-erythrocytic 1	SPTAN1	53.83	-0.32691	-0.4504	-0.34477	-0.34217	-0.58168	-0.44763	6.05	0.13	6.57	6.02	-0.42	0.10	-0.40921	-0.41335	-0.34514	-0.27984	-0.64395	-0.41843	0.20	3.20	11.03	7.42	-0.42	0.12
O15020	Spectrin beta chain, non-erythrocytic 2	SPTBN2	7.17	0.689786	0.326325	0.678602	1.423683	0.228302	0.513301	17.72	35.73	13.92	27.85	0.64	0.42	0.787308	0.742157	1.53737	2.324298	-0.66314	0.60428	2.21	37.64	58.41	67.53	0.89	1.00
B5BU83	Stathmin	STMN1	2.80	-0.1597	-0.6708	0.482357	NA	-1.51624	-1.78068	24.79	NA	12.92	65.50	-0.73	0.94	-0.4756	-0.43434	-0.00787	NA	-1.51336	-1.53892	2.02	NA	1.25	47.13	-0.79	0.69
Q9UJZ1	Stomatin-like protein 2, mitochondrial	STOML2	5.33	NA	NA	-0.22098	-0.06318	0.549418	NA	NA	7.73	NA	33.16	0.09	0.41	NA	NA	-0.37502	-0.83764	0.726465	NA	NA	22.48	NA	60.30	-0.16	0.80
Q14683	Structural maintenance of chromosomes protein 1A	SMC1A	1.00	1.590398	NA	NA	1.520956	NA	NA	NA	NA	NA	3.40	1.56	0.05	1.219604	NA	NA	0.950977	NA	NA	NA	NA	NA	13.13	1.09	0.19
V9HWC9	Superoxide dismutase [Cu-Zn]	SOD1	11.50	0.258467	0.121539	0.578143	0.378871	0.575074	0.631229	6.71	9.75	2.75	14.23	0.42	0.21	0.207984	0.254489	0.36122	0.341927	0.327665	0.448422	2.28	0.95	5.92	5.64	0.32	0.08
A0A024R7Z5	Syndecan binding protein (Syntenin), isoform CRA_c	SDCBP	1.75	-0.38793	-0.17154	0.042055	-0.35122	NA	NA	10.59	19.16	NA	6.59	-0.22	0.20	-0.53373	0.718916	-0.12	0.262109	NA	NA	57.81	18.62	NA	6.90	0.08	0.53
O15061	Synemin	SYNM	4.67	0.59066	0.258122	0.400787	-0.34284	0.091204	0.537067	16.23	35.66	21.68	12.42	0.26	0.35	0.702326	0.721992	0.790519	-0.07218	0.648539	0.766863	0.96	41.07	5.80	11.11	0.59	0.33
Q9Y490	Talin-1	TLN1	12.67	0.72129	1.513624	0.949289	1.809034	0.997622	1.045696	37.89	40.93	2.36	15.03	1.17	0.41	0.899862	0.95615	0.565002	1.529835	1.094343	1.168056	2.76	45.60	3.61	7.85	1.04	0.32
P17987	T-complex protein 1 subunit alpha	TCP1	8.50	-0.18097	-0.30213	-0.04059	-0.07285	-0.03059	-0.03635	5.94	1.58	0.28	7.68	-0.11	0.11	-0.2118	0.024255	0.086435	-0.05137	-0.724	-0.62876	11.54	6.75	4.67	23.93	-0.25	0.35
P50991	T-complex protein 1 subunit delta	CCT4	7.17	0.487731	-0.41681	0.15872	0.487031	-0.52185	0.001555	42.94	16.02	25.38	19.36	0.03	0.43	0.578667	-0.61053	-0.04315	-0.12569	-0.55772	-0.19416	55.20	4.04	17.73	16.01	-0.16	0.43
Q71U36	Tubulin alpha-1A chain	TUBA1A	44.00	NA	NA	-1.11729	NA	-0.95114	-1.42563	NA	NA	23.05	2.53	-1.16	0.24	NA	NA	-1.43466	NA	-0.65954	-0.10857	NA	NA	26.68	50.45	-0.73	0.67
P68366	Tubulin alpha-4A chain	TUBA4A	38.00	-1.32715	-1	-0.75421	-0.2128	-1.78633	-1.73904	15.97	26.23	2.32	44.74	-1.14	0.61	-1.45713	-1.18026	-0.16298	-0.45833	-1.07534	-1.63473	13.53	14.43	27.08	44.07	-0.99	0.57
Q13885	Tubulin beta-2A chain	TUBB2A	44.67	-2.39853	-1.94854	-1.88467	-1.53993	-1.81408	-1.95273	21.88	16.82	6.79	15.55	-1.92	0.28	-2.43564	-2.43821	-2.15959	-1.91594	-1.60286	-2.12856	0.13	11.91	25.48	20.21	-2.11	0.32
Q13509	Tubulin beta-3 chain	TUBB3	31.50	NA	NA	NA	-0.61991	NA	-3.02394	NA	NA	NA	96.47	-1.82	1.70	NA	NA	NA	0.014563	NA	-1.02909	NA	NA	NA	49.03	-0.51	0.74
P04350	Tubulin beta-4A chain	TUBB4A	46.00	0.028457	0.195182	0.536354	0.677087	0.732444	0.651165	8.16	6.89	3.98	20.27	0.47	0.29	-0.14389	0.371698	0.160078	0.459638	0.392583	-0.06179	25.01	14.63	22.09	6.59	0.20	0.25
Q8IZ29	Tubulin, beta 2C	TUBB2C	54.83	-1.6651	-1.64149	-0.73776	-0.85478	-1.41213	-1.67265	1.16	5.73	12.73	34.40	-1.33	0.43	-1.57361	-1.81631	-1.14664	-0.91261	-1.43859	-1.77716	11.87	11.45	16.52	26.35	-1.44	0.36
E5RJD8	Tubulin-specific chaperone A	TBCA	2.33	1.576605	1.683756	0.658088	1.008809	0.233424	0.81549	5.25	17.11	28.15	40.67	1.00	0.55	0.673216	1.666914	0.703221	0.827439	0.185442	0.517802	46.87	6.08	16.22	31.04	0.76	0.50
B4DLN1	Uncharacterized protein	NA	2.40	-0.23045	0.489624	-0.60404	-0.2958	NA	0.383786	34.58	15.05	NA	27.54	-0.05	0.47	-0.05016	0.998677	-1.24218	-0.89608	NA	-0.59118	49.26	16.88	NA	60.94	-0.36	0.87
H3BN98	Uncharacterized protein (Fragment)	NA	5.83	-0.8454	-0.99924	-0.41265	-0.90129	0.248682	0.365254	7.53	23.72	5.71	47.42	-0.42	0.60	-0.92361	-0.83388	-0.62877	-0.58131	-0.19522	0.152153	4.40	2.33	16.94	31.87	-0.50	0.41

				08/017 hESC-RPE to hRPE												08/023 hESC-RPE to hRPE											
				Analysis						Technical replicate CV			Biological CV	Fold change (mean)	StDev	Analysis						Technical replicate CV			Biological CV	Fold change (mean)	StDev
UniProt	Protein name	Gene name	Peptide mean	1.1	1.2	2.1	2.2	3.1	3.2	1	2	3				1.1	1.2	2.1	2.2	3.1	3.2	1	2	3			
Q9UM54	Unconventional myosin-VI	MYO6	1.50	NA	NA	-0.63828	-1.10964	-0.96499	-1.16989	NA	22.90	10.03	10.23	-0.97	0.24	NA	NA	-0.7018	-0.72324	-0.47061	-1.6434	NA	1.05	54.51	11.18	-0.88	0.52
Q9P0L0	Vesicle-associated membrane protein-associated protein A	VAPA	2.67	0.310029	-0.13648	NA	-0.27619	NA	NA	21.71	NA	NA	18.53	-0.03	0.31	0.502449	-0.19684	NA	0.089762	NA	NA	33.62	NA	NA	5.14	0.13	0.35

Supplementary Table S7. The 83 proliferation and cell cycle proteins presented in Figure 4d. Differences in expression for both hESC-RPE lines compared to hRPE presented on log2 scale.
Mean number of peptides used for quantification, fold change value for each replicate analysis, technical replicate CV, biological replicate CV, mean fold change, and standard deviation shown for each protein. Note! CV values are calculated using normal scale, not the log2 scale shown here.
Differentially expressed proteins (>2-fold difference in expression i.e. values >1 and <-1 on log2 scale) marked with red color for upregulation and green color for downregulation.

				08/017 hESC-RPE to hRPE												08/023 hESC-RPE to hRPE											
				Analysis						Technical replicate CV						Analysis						Technical replicate CV					
UniProt	Protein name	Gene name	Peptide mean	1.1	1.2	2.1	2.2	3.1	3.2	1	2	3	Biological CV	Fold change (mean)	StDev	1.1	1.2	2.1	2.2	3.1	3.2	1	2	3	Biological CV	Fold change (mean)	StDev
P61981	14-3-3 protein gamma	YWHAG	8.67	-0.27002	-0.57918	0.147277	-0.10255	-1.99226	-2.10735	15.10	12.21	5.64	58.92	-0.82	0.98	-0.01542	-0.1718	0.166406	-0.04407	-0.27404	-0.74559	7.66	10.30	22.91	18.99	-0.18	0.31
P35998	26S protease regulatory subunit 7	PSMC2	2.67	0.382338	-0.523	1.035475	2.071931	0.280219	0.490949	42.97	48.72	10.31	63.42	0.62	0.87	-0.23431	0.04063	1.255168	1.070065	-0.00165	1.094868	13.43	9.06	51.30	41.19	0.54	0.67
P62195	26S protease regulatory subunit 8	PSMC5	2.33	0.38445	-0.12236	NA	NA	-0.22928	NA	24.59	NA	NA	18.64	0.01	0.33	0.535932	-0.08136	NA	NA	0.062322	NA	29.80	NA	NA	9.68	0.17	0.32
O00231	26S proteasome non-ATPase regulatory subunit 11	PSMD11	3.00	NA	NA	NA	0.408966	0.075238	NA	NA	NA	NA	16.28	0.24	0.24	NA	NA	NA	-0.57904	0.219761	NA	NA	NA	NA	38.18	-0.18	0.56
O00487	26S proteasome non-ATPase regulatory subunit 14	PSMD14	1.50	NA	NA	NA	0.098951	0.660863	NA	NA	NA	NA	27.20	0.38	0.40	NA	NA	NA	0.137572	1.448961	NA	NA	NA	NA	60.19	0.79	0.93
P23396	40S ribosomal protein S3	RPS3	5.50	0.368071	0.286259	0.250701	0.427314	0.435787	0.705996	4.01	8.65	13.21	9.97	0.41	0.16	0.309782	0.47424	0.295315	0.285072	0.533677	0.559221	8.05	0.50	1.25	9.00	0.41	0.13
P61160	Actin-related protein 2	ACTR2	1.50	1.017997	NA	NA	NA	-0.5041	NA	NA	NA	NA	68.37	0.26	1.08	1.141303	NA	NA	NA	0.04198	NA	NA	NA	NA	51.42	0.59	0.78
Q6FGX9	Adenylate kinase isoenzyme 1	AK1	5.67	-0.40738	-0.06226	-0.04141	-0.85812	-0.77936	-0.87628	16.84	38.99	4.75	20.51	-0.50	0.39	-0.2338	0.138164	-0.19652	-0.73663	-0.47176	-0.95874	18.13	26.17	23.64	23.46	-0.41	0.40
P36405	ADP-ribosylation factor-like protein 3	ARL3	2.67	0.166224	-0.13585	NA	NA	NA	-0.40415	14.75	NA	NA	20.79	-0.12	0.29	0.711589	-0.23238	NA	NA	NA	-0.36617	44.68	NA	NA	32.80	0.04	0.59
Q5TZZ9	Annexin	ANXA1	12.67	0.454789	0.563415	-0.19253	-0.46524	-0.71576	-0.50624	5.32	13.33	10.25	42.52	-0.14	0.53	0.193686	0.344362	0.56637	0.372852	0.310761	0.399269	7.38	9.47	4.34	7.06	0.36	0.12
Q5TZP7	APEX nuclease (Multifunctional DNA repair enzyme) 1	APEX1	5.80	-0.68237	-0.72939	0.529209	NA	-0.90827	-1.00737	2.30	NA	4.86	59.48	-0.56	0.62	-0.71572	-0.60851	1.016231	NA	-0.99566	-0.74165	5.25	NA	12.42	77.47	-0.41	0.81
A0A024R3E3	Apolipoprotein A-I, isoform CRA_a	APOA1	2.33	2.634717	2.317136	1.794767	2.021072	0.964121	0.421314	15.50	11.07	26.30	53.91	1.69	0.84	2.171774	2.231446	1.251878	1.297959	2.338172	1.711849	2.92	2.26	30.22	30.95	1.83	0.48
P02649	Apolipoprotein E	APOE	2.40	NA	0.463017	-0.40071	-0.26982	-0.76788	-0.13068	NA	6.41	30.73	36.02	-0.22	0.45	NA	0.988607	1.12512	-0.52104	-0.22391	0.423736	NA	72.94	31.22	29.63	0.36	0.72
A0A024RAI1	ARP3 actin-related protein 3 homolog (Yeast), isoform CRA_a	ACTR3	3.80	0.5114	0.212651	0.379959	0.579905	NA	0.387248	14.59	9.78	NA	4.29	0.41	0.14	-0.17497	0.825219	0.219099	0.381401	NA	1.021042	47.15	7.95	NA	28.40	0.45	0.48
Q12797	Aspartyl/asparaginyl beta-hydroxylase	ASPH	4.67	-0.57878	-0.62675	-0.11098	-0.38078	-0.92013	-1.06416	2.35	13.19	7.05	25.70	-0.61	0.35	-0.05403	-0.5632	-0.43466	-0.57904	-0.36831	-0.4915	24.70	7.07	6.03	7.77	-0.42	0.19
Q5SU16	Beta 5-tubulin	TUBB	51.33	0.363029	0.400538	0.546916	0.763949	-0.2626	-0.1428	1.84	10.62	5.87	28.61	0.28	0.40	0.079708	0.279032	0.698238	0.975697	0.239162	0.403484	9.75	13.56	8.05	25.25	0.45	0.33
V9HW88	Calreticulin, isoform CRA_b	CALR	20.17	0.842497	1.10416	0.762397	1.06445	0.940425	0.90047	12.79	14.75	1.96	2.35	0.94	0.13	0.906891	1.199803	0.707256	0.77664	0.828032	1.067036	14.31	3.40	11.69	11.01	0.91	0.19
P21964	Catechol O-methyltransferase	COMT	4.17	-0.00296	-0.03516	0.291553	0.388972	0.124156	-0.58928	1.58	4.77	34.27	19.24	0.03	0.34	0.54076	0.341408	0.393751	0.321676	-0.43663	-0.6281	9.76	3.53	9.37	32.82	0.09	0.49
Q6FHM9	CD59 antigen, complement regulatory protein, isoform CRA_b	CD59	2.00	0.87849	NA	NA	NA	NA	0.16464	NA	NA	NA	34.29	0.52	0.50	0.68866	NA	NA	NA	NA	0.942564	NA	NA	NA	12.41	0.82	0.18
P41208	Centrin-2	CETN2	1.25	-0.96596	-0.40696	-0.42826	NA	0.211498	NA	27.06	NA	NA	32.76	-0.40	0.48	0.234487	-0.09189	0.537021	NA	0.438166	NA	15.93	NA	NA	15.93	0.28	0.28
Q5SRT3	Chloride intracellular channel protein	CLIC1	2.33	0.201574	0.197998	0.170543	0.032458	0.856059	-0.08125	0.18	6.76	44.39	13.18	0.23	0.33	-0.03302	0.18078	-0.03743	0.131596	0.593553	0.303289	10.46	8.27	14.18	16.35	0.19	0.24
P10909	Clusterin	CLU	3.00	0.022462	-0.04884	0.232993	0.836901	-0.18857	NA	3.49	29.17	NA	28.71	0.17	0.40	0.253361	-0.55314	0.111013	0.807679	-0.01079	NA	38.53	33.50	NA	23.47	0.12	0.49
O75367	Core histone macro-H2A.1	H2AFY	6.67	-0.36798	-0.6498	0.234346	-0.10396	-0.27523	-0.39	13.77	16.51	5.62	21.20	-0.26	0.30	-0.30375	-0.38142	0.720371	-0.00835	-0.25936	-0.37114	3.81	34.98	5.48	31.17	-0.10	0.42
Q14204	Cytoplasmic dynein 1 heavy chain 1	DYNC1H1	19.83	-0.0077	0.604321	-0.38124	1.450071	0.06592	0.415921	29.55	79.38	17.07	21.84	0.36	0.64	-0.00791	0.922597	-0.80192	1.248047	0.365554	0.335884	44.09	86.40	1.45	7.69	0.34	0.72
P78527	DNA-dependent protein kinase catalytic subunit	PRKDC	6.50	-0.64218	0.24184	0.319222	0.138766	0.028582	-0.36686	42.02	8.83	19.26	15.67	-0.05	0.38	0.210069	-0.74403	-0.61591	-0.2649	0.19143	-0.38673	45.13	17.12	27.96	12.46	-0.27	0.40
P13639	Elongation factor 2	EEF2	13.67	0.923397	1.02176	0.157294	0.676922	0.698563	1.171119	4.82	25.20	22.96	19.56	0.77	0.36	1.022826	1.130535	0.191886	0.720622	0.88423	1.240613	5.28	25.63	17.38	22.00	0.87	0.38
V9HW22	Epididymis luminal protein 33	HSPA8	35.50	-0.11382	-0.19134	0.292852	0.243798	0.001188	0.037939	3.80	2.40	1.80	14.81	0.05	0.19	-0.14317	-0.14159	0.342182	0.431473	-0.16732	-0.16578	0.08	4.38	0.08	22.86	0.03	0.28
V9HW58	Epididymis secretory protein Li 77	DCTN2	4.20	0.239584	0.346322	0.111169	-0.64484	-0.46691	NA	5.23	36.23	NA	27.75	-0.08	0.44	-0.18515	-0.26882	-0.22729	-0.14481	-0.40645	NA	4.10	4.04	NA	7.98	-0.25	0.10
P63241	Eukaryotic translation initiation factor 5A-1	EIF5A	4.83	-0.60968	-1.78588	0.27872	-0.239	-1.22535	-0.63705	54.65	25.11	28.44	44.95	-0.70	0.73	-0.59232	-0.10273	0.372797	0.165066	-0.16918	-0.05955	23.77	10.16	5.37	21.53	-0.06	0.33
Q16658	Fascin	FSCN1	2.75	0.970167	NA	-0.15663	0.064324	-0.22225	NA	NA	10.81	NA	47.99	0.16	0.55	0.165243	NA	0.308862	-0.20056	0.502488	NA	NA	24.71	NA	16.09	0.19	0.30
O15540	Fatty acid-binding protein, brain	FABP7	2.00	-0.58674	-1.20179	NA	-0.44572	-0.46575	NA	29.70	NA	NA	15.44	-0.67	0.36	-0.87739	-1.37398	NA	-0.63751	-1.11869	NA	24.10	NA	NA	19.88	-1.00	0.32
Q9UK22	F-box only protein 2	FBXO2	1.25	1.623996	NA	0.96363	0.250462	NA	0.075711	NA	34.26	NA	55.43	0.73	0.71	1.447108	NA	0.011594	-0.52285	NA	-1.09205	NA	25.90	NA	89.54	-0.04	1.09
Q6ZVX7	F-box only protein 50	NCCRP1	2.33	1.517464	1.042644	1.845506	1.644994	0.370895	2.912346	23.06	9.81	99.96	28.60	1.56	0.85	2.369177	1.450086	1.052187	1.605343	0.454857	2.690151	43.58	26.78	91.87	22.82	1.60	0.83
P21333	Filamin-A	FLNA	38.83	1.279703	1.27822	1.027436	0.960179	0.79485	1.030971	0.07	3.30	11.55	13.57	1.06	0.19	1.270175	1.293218	1.450871	1.393843	1.132476	1.327719	1.13	2.79	9.55	6.91	1.31	0.11
P14136	Glial fibrillary acidic protein	GFAP	12.50	NA	NA	NA	-1.46451	NA	-0.50684	NA	NA	NA	45.29	-0.99	0.68	NA	NA	NA	-1.62929	NA	-0.09479	NA	NA	NA	68.84	-0.86	1.09
A8YXX4	Glutamine synthetase	PIG59	10.83	1.116885	-0.20791	-1.24968	-1.52848	-1.29362	-1.9128	60.72	13.62	29.89	89.62	-0.85	1.12	1.009174	-0.13552	-0.79225	-1.19969	-0.90343	-1.73095	53.34	19.84	39.48	72.76	-0.63	0.96
A0A024R2Z1	Guanine nucleotide binding protein (G protein), alpha transducing activity polypeptide 1, isoform CRA_a	GNAT1	25.83	-1.88085	-2.19917	-3.03732	-3.23807	-2.34241	-2.8922	15.54	9.82	26.63	37.65	-2.60	0.53	-1.92403	-2.42514	-2.50833	-2.54883	-2.55407	-3.19661	24.32	1.98	30.98	23.93	-2.53	0.41
A0A024R056	Guanine nucleotide binding protein (G protein), beta polypeptide 1, isoform CRA_a	GNB1	36.50	-1.24042	NA	-2.15752	NA	NA	NA	NA	NA	NA	43.49	-1.70	0.65	-1.27165	NA	-1.9301	NA	NA	NA	NA	NA	NA	31.72	-1.60	0.47
P04899	Guanine nucleotide-binding protein G(i) subunit alpha-2	GNAI2	6.80	0.539571	0.43619	0.323253	NA	-0.70068	0.159588	5.06	NA	40.96	23.59	0.15	0.50	0.32805	0.285916	0.015355	NA	-0.08189	0.981035	2.06	NA	49.86	18.15	0.31	0.42
X5D945	Harvey rat sarcoma viral oncoprotein isoform A (Fragment)	HRAS	1.00	NA	NA	-0.07061	NA	-0.54227	NA	NA	NA	NA	22.91	-0.31	0.33	NA	NA	0.651962	NA	-0.10884	NA	NA	NA	NA	36.45	0.27	0.54
Q5SSJ5	Heterochromatin protein 1-binding protein 3	HP1BP3	4.83	-1.51297	-1.2538	-1.23756	-2.00753	-1.14556	-1.33656	12.67	6.37	9.35	28.10	-1.57	0.41	-1.48079	-1.02198	-1.40353	-1.4881	-1.705	-1.35025	22.30	4.14	17.30	10.43	-1.41	0.22
A0A024RDR0	High-mobility group box 1, isoform CRA_a	HMGB1	4.00	-0.98375	-0.79309	-0.97087	NA	NA	-0.73268	9.33	NA	NA	8.45	-0.87	0.13	-0.53468	-0.71336	-1.28717	NA	NA	-1.16843	8.75	NA	NA	25.88	-0.93	0.36
Q5U071	High-mobility group box 2	NA	4.25	-0.95256	-1.12816	NA	NA	-0.49264	-1.43859	8.60	NA	44.77	7.26	-1.00	0.40	-0.77448	-1.0781	NA	NA	-0.58042	-1.23037	14.83	NA	31.33	2.41	-0.92	0.29
Q09028	Histone-binding protein RBBP4	RBBP4	1.00	NA	0.29943	NA	NA	NA	0.548418	NA	NA	NA	12.17	0.42	0.18	NA	0.192976	NA	NA	NA	0.138408	NA	NA	NA	2.67	0.17	0.04
P05556	Integrin beta-1	ITGB1	2.33	0.149462	-0.28122	0.62839	0.613721	0.735134	0.260371	20.95	0.72	23.06	23.18	0.35	0.39	-0.25717	-0.68085	0.93993	0.767182	0.983781	0.313317	20.62	8.46	32.28	41.53	0.34	0.69
L7RXH0	Integrin, alpha V	ITGAV	7.50	0.720813	0.426053	0.602438	0.157323	0.838343	0.658794	14.40	21.64	8.79	12.23	0.57	0.24	0.716264	0.766325	0.62317	0.033178	0.528359	0.463717	2.45	28.52	3.19	13.66	0.52	0.26
Q08722	Leukocyte surface antigen CD47	CD47	2.33	-1.16168	-1.18702	-1.03081	-1.11534	-1.15083	-1.21412	1.24	4.14	3.10	4.29	-1.14	0.06	-1.17943	-0.60683	-0.40554	-1.187	-0.85363	-0.93521	27.70	37.39	4.00	5.58	-0.86	0.31
I4AY87	Macrophage migration inhibitory factor (Fragment)	MIF	7.17	1.511884	1.464049	1.816903	1.642547	1.995871	2.328022	2.34	8.54	16.21	24.58	1.79	0.33	1.49306	1.608784	1.451427	1.373144	1.39628	1.740643	5.67	3.84	16.80	6.07	1.51	0.14
P49006	MARCKS-related protein	MARCKSL1	1.00	1.493755	1.169756	NA	2.014497	0.854514	-0.39056	15.81	NA	57.50	52.66	1.03	0.90	1.050596	1.169852	NA	1.839123	1.453649	1.322763	5.84	NA	6.41	25.95	1.37	0.30
Q1HBJ4	Mitogen-activated protein kinase	MAPK1	2.75	NA	NA	0.147517	-0.26823	-1.00933	-0.71193	NA	20.24	14.52	38.58	-0.46	0.51	NA	NA	-0.25904	-0.13121	-0.91625	-0.41073	NA	6.26	24.53	21.77	-0.43	0.34
A0A024R1N1	Myosin, heavy polypeptide 9, non-muscle, isoform CRA_a	MYH9	23.00	0.80772	1.391883	1.00278	0.681532	0.289513	0.159967	28.25	15.68	6.35	29.87	0.72	0.46	0.716398	1.418696	1.524849	1.114448	1.249635	0.980167	33.76	19.98	13.17	8.97	1.17	0.30
P35580	Myosin-10	MYH10	10.17	1.357816	1.470366	0.552529	0.574781	0.442283	0.37564	5.51	1.09	3.27	40.21	0.80	0.49	1.376116	1.211269	0.285798	1.163715	0.740394	0.386223	8.07	41.75	17.27	26.61	0.86	0.46
O14745	Na(+)/H(+) exchange regulatory cofactor NHE-RF1	SLC9A3R1	8.67	1.385279	1.86852	1.280794	1.40678	1.46048	1.42337	23.47	6.17	1.82	10.85	1.47	0.20	1.407596	1.91543	1.841261	1.905157	1.435722	1.267986	24.64	3.13	8.21	17.71	1.63	0.29
O43181	NADH dehydrogenase [ubiquinone] iron-sulfur protein 4, mitochondrial	NDUFS4	2.75	0.048772	0.893158	-1.67717	-1.17183	NA	NA	40.24	24.52	NA	82.77	-0.48	1.17	-0.30672	0.756576	-0.99369	-0.65251	NA	NA	49.88	16.64	NA	52.88	-0.30	0.76
P06748	Nucleophosmin	NPM1	7.67	0.599781	0.343483	1.322935	0.611135	0.635892	0.643133	12.53	34.20	0.35	19.48	0.69	0.33	0.658437	0.706411	1.048766	0.292303	0.114252	0.503375	2.35	36.25	18.96	14.53	0.55	0.33
P15531	Nucleoside diphosphate kinase A	NME1	7.00	-0.33409	-1.45872	-0.21266	-0.67651	NA	-1.90537	52.49	22.54	NA	45.73	-0.92	0.74	-0.22916	-1.713	-0.051	-0.08337	NA	-0.9675	66.93	1.59	NA	35.02	-0.61	0.72
Q13526	Peptidyl-prolyl cis-trans isomerase NIMA-interacting 1	PIN1	1.00	2.227067	NA	NA	NA	-0.33358	NA	NA	NA	NA	100.43	0.95	1.81	0.972458	NA	NA	NA	0.094827	NA	NA	NA	NA	41.74	0.53	0.62
Q06830	Peroxiredoxin-1	PRDX1	17.50	0.03462	0.066588	0.110187	0.35907	0.106996	0.038215	1.57	12.17	3.37	7.31	0.12	0.12	0.179438	0.234586	0.125468	0.141009	-0.30487	-0.3915	2.70	0.76	4.24	19.58	0.00	0.27
B1AKZ4	Phosphoprotein enriched in astrocytes 15, isoform CRA_a	PEA15	5.17	0.49599	-1.85905	-1.6478	-1.72275	-2.40767	-1.61308	95.18	3.67	37.99	68.77	-1.46	1.00	0.564513	-0.81398	-1.20437	-0.97672	-1.70163	-1.43281	62.85	11.13	13.14	59.44	-0.93	0.80
P36955	Pigment epithelium-derived factor	SERPINF1	3.17	0.815571	1.024376	0.644927	0.269614	0.412975	0.251851	10.22	18.29	7.89	22.27	0.57	0.31	0.730729	0.683406	0.197634	0.607249	0.361375	0.200093	2.32	19.94	7.90	15.37	0.46	0.24
P02545	Prelamin-A/C	LMNA	20.50	0.108054	0.455838	0.610099	0.542927	-0.31607	-0.28296	16.96	3.29	1.62	29.08	0.19	0.41	0.028853	0.188148	0.289323	0.415668	-0.19664	-0.05199	7.80	6.19	7.08	16.48	0.11	0.23
Q07954	Prolow-density lipoprotein receptor-related protein 1	LRP1	2.50	0.663307	0.675935	-0.04332	0.42437	0.532895	0.159588	0.62	22.72	18.20	16.65	0.40	0.29	0.696389	0.197595	-0.02888	-0.30674	0.982944	0.239434	24.21	13.58	35.66	27.42	0.30	0.47
A0A024R8G3	Prostaglandin D2 synthase 21kDa (Brain), isoform CRA_a	PTGDS	2.00	0.512781	NA	1.188913	NA	0.157125	NA	NA	NA	NA	37.52	0.62	0.52	0.394069	NA	1.093331	NA	0.637548	NA	NA	NA	NA	25.25	0.71	0.35
P49720	Proteasome subunit beta type-3	PSMB3	2.00	-0.67418	NA	-0.58205	-0.64319	NA	NA	NA	3.00	NA	3.03	-0.63	0.05	-0.51013	NA	-0.27273	-0.13647	NA	NA	NA	6.67	NA	15.00	-0.31	0.19
P28070	Proteasome subunit beta type-4	PSMB4	2.67	-0.30218	NA	NA	0.230153	-1.10802	NA	NA	NA	NA	43.45	-0.39	0.67	1.122103	NA	NA	-0.77887	-0.08299	NA	NA	NA	NA	67.69	0.09	0.96
V9HWH9	Protein S100	S100A11	2.17	0.718063	0.758146	0.794385	-0.32291	0.895218	0.611754	1.96	52.18	13.85	15.52	0.58	0.45	0.530784	0.841666	1.033147	-0.2349	0.495025	0.79317	15.18	58.44	14.56	5.69	0.58	0.45
P04271	Protein S100-B	S100B	2.40	-0.18611	NA	0.516666	-0.13518	-0.58482	-0.57426	NA	31.42	0.52	27.79	-0.19	0.45	2.362759	NA	1.477197	1.572883	0.486047	0.675179	NA	4.69	9.26	57.98	1.31	0.76
P61026	Ras-related protein Rab-10	RAB10	3.33	0.126744	-0.41605	-0.50217	-0.53343	-1.49994	-1.09492	26.29	1.53	19.72	37.77	-0.65	0.57	-0.26532	-0.34738	-1.2706	-0.7493	-1.10617	-0.3128	4.02	25.28	37.93	23.51	-0.68	0.44
P11233	Ras-related protein Ral-A	RALA	2.00	NA	-0.16919	-0.56195	NA	NA	NA	NA	NA	NA	19.13	-0.37	0.28	NA	-0.26273	-0.61088	NA	NA	NA	NA	NA	NA	16.98	-0.44	0.25
Q5TZA2	Rootletin	CROCC	10.80	0.262247	0.549085	-0.47097	-1.16827	NA	-0.36213	14.01	33.53	NA	43.24	-0.24	0.67	-0.00637	0.347483	-0.33585	-1.1202	NA	0.202141	17.26	37.52	NA	30.72	-0.18	0.58
B5MCX3	Septin-2	SEPT2	3.83	-0.07039	-0.21605	0.144642	-0.31044	-0.36312	-0.00723	7.13	22.12	17.36	3.91	-0.14	0.19	-0.08646	-0.21311	-0.1025	-0.53563	-0.38118	0.049145	6.12	12.37	20.94	2.97	-0.18	0.17
V9HW06	Serine hydroxymethyltransferase	SHMT2	1.33	NA	-1.79573	NA	0.26106	-0.7931	NA	NA	NA	NA	67.63	-0.78	1.03	NA	-2.40282	NA	0.276347	-0.73531	NA	NA	NA	NA	77.10	-0.95	1.35
Q15257	Serine/threonine-protein phosphatase 2A activator	PPP2R4	2.00	NA	-1.6915	-0.8196	-0.87543	NA	NA	NA	2.74	NA	40.24	-1.13	0.49	NA	-1.00243	-0.94611	-1.17669	NA	NA	NA	11.28	NA	2.66	-1.04	0.12
P62136	Serine/threonine-protein phosphatase PP1-alpha catalytic subunit	PPP1CA	3.20	0.190577	0.014731	0.765987	0.362765	NA	0.795598	8.61	19.64	NA	23.27	0.43	0.35	0.650781	0.342104	0.620704	-0.93608	NA	0.372397	15.07	69.67	NA	15.91	0.21	0.66
P63208	S-phase kinase-associated protein 1	SKP1	4.83	-0.14831	-0.21274	0.984276	0.980363	-0.19016	-0.07171	3.16	0.19	5.80	49.48	0.22	0.59	0.076803	-0.2835	0.936496	1.191935	-0.33376	0.411393	17.57	12.49	35.73	46.67	0.33	0.63
Q14683	Structural maintenance of chromosomes protein 1A	SMC1A	1.00	1.590398	NA	NA	1.520956	NA	NA	NA	NA	NA	3.40	1.56	0.05	1.219604	NA	NA	0.950977	NA	NA	NA	NA	NA	13.13	1.09	0.19
A0A0C4DFU2	Superoxide dismutase	SOD2	6.00	-0.1575	0.02017	-1.38021	-1.20002	-0.46267	-0.31196	8.70	8.47	7.38	39.00	-0.58	0.57	-0.02319	0.074507	-0.88548	-0.82105	0.283158	0.32242	4.79	3.16	1.92	37.16	-0.17	0.54
P30048	Thioredoxin-dependent peroxide reductase, mitochondrial	PRDX3	7.00	0.018969	0.552972	-0.3699	-0.44285	-0.01949	0.460563	25.88	3.57	23.31	25.02	0.03	0.41	0.257033	0.357665	-0.47102	-0.53706	-0.1736	0.363605	4.93	3.24	26.03	27.18	-0.03	0.41
Q71U36	Tubulin alpha-1A chain	TUBA1A	44.00	NA	NA	-1.11729	NA	-0.95114	-1.42563	NA	NA	23.05	2.53	-1.16	0.24	NA	NA	-1.43466	NA	-0.65954	-0.10857	NA	NA	26.68	50.45	-0.73	0.67
P68366	Tubulin alpha-4A chain	TUBA4A	38.00	-1.32715	-1	-0.75421	-0.2128	-1.78633	-1.73904	15.97	26.23	2.32	44.74	-1.14	0.61	-1.45713	-1.18026	-0.16298	-0.45833	-1.07534	-1.63473	13.53	14.43	27.08	44.07	-0.99	0.57
Q13509	Tubulin beta-3 chain	TUBB3	31.50	NA	NA	NA	-0.61991	NA	-3.02394	NA	NA	NA	96.47	-1.82	1.70	NA	NA	NA	0.014563	NA	-1.02909	NA	NA	NA	49.03	-0.51	0.74
P04350	Tubulin beta-4A chain	TUBB4A	46.00	0.028457	0.195182	0.536354	0.677087	0.732444	0.651165	8.16	6.89	3.98	20.27	0.47	0.29	-0.14389	0.371698	0.160078	0.459638	0.392583	-0.06179	25.01	14.63	22.09	6.59	0.20	0.25
P13010	X-ray repair cross-complementing protein 5	XRCC5	10.83	-0.45948	-0.18875	0.546371	0.330423	-0.53273	-0.66524	13.23	10.56	6.49	39.23	-0.16	0.49	-0.52016	-0.26802	0.286213	-0.00759	-0.74741	-0.75676	12.33	14.35	0.46	31.83	-0.34	0.42

Supplementary Table S8. Proteins involved in detection of light stimulus and selected RPE proteins shown in Figure 5. Differences in expression for both hESC-RPE lines compared to hRPE presented on log2 scale.
Mean number of peptides used for quantification, fold change value for each replicate analysis, technical replicate CV, biological replicate CV, mean fold change, and standard deviation shown for each protein. Note! CV values are calculated using normal scale, not the log2 scale shown here.
Differentially expressed proteins (>2-fold difference in expression i.e. values >1 and <-1 on log2 scale) marked with red color for upregulation and green color for downregulation.

Detection of light stimulus

UniProt	Protein name	Gene name	Peptide mean	08/017 hESC-RPE to hRPE												08/023 hESC-RPE to hRPE											
				Analysis						Technical replicate CV			Biological CV	Fold change (mean)	StDev	Analysis						Technical replicate CV			Biological CV	Fold change (mean)	StDev
				1.1	1.2	2.1	2.2	3.1	3.2	1	2	3				1.1	1.2	2.1	2.2	3.1	3.2	1	2	3			
P02489	Alpha-crystallin A chain	CRYAA	9.67	-1.10681	-1.04082	-2.43148	-2.1051	-1.53111	-1.62654	3.23	15.93	4.68	39.21	-1.64	0.55	-1.06636	-1.02826	-1.92145	-1.96239	-2.0942	-1.89325	1.87	2.01	9.83	39.66	-1.66	0.48
P02649	Apolipoprotein E	APOE	2.40	NA	0.463017	-0.40071	-0.26982	-0.76788	-0.13068	NA	6.41	30.73	36.02	-0.22	0.45	NA	0.988607	1.12512	-0.52104	-0.22391	0.423736	NA	72.94	31.22	29.63	0.36	0.72
P36575	Arrestin-C	ARR3	8.33	-0.7526	-0.91057	-0.66535	-0.49131	-0.73447	-1.23626	7.73	8.52	24.35	13.89	-0.80	0.25	-0.81477	-0.89608	-0.84173	-0.25252	-0.83433	-1.073	3.98	28.48	11.67	16.24	-0.79	0.28
A0A024R2Z1	Guanine nucleotide binding protein (G protein), alpha transducing activity polypeptide 1, isoform CRA_a	GNAT1	25.83	-1.88085	-2.19917	-3.03732	-3.23807	-2.34241	-2.8922	15.54	9.82	26.63	37.65	-2.60	0.53	-1.92403	-2.42514	-2.50833	-2.54883	-2.55407	-3.19661	24.32	1.98	30.98	23.93	-2.53	0.41
Q5T697	Guanine nucleotide binding protein (G protein), alpha transducing activity polypeptide 2	GNAT2	12.00	NA	-1.53809	-0.68951	NA	NA	NA	NA	NA	NA	40.43	-1.11	0.60	NA	-2.08697	-0.81879	NA	NA	NA	NA	NA	NA	58.44	-1.45	0.90
P63211	Guanine nucleotide-binding protein G(T) subunit gamma-T1	GNGT1	7.67	-2.13723	-2.4447	-2.92436	-3.24056	-2.59344	-2.40752	15.01	15.44	9.10	26.46	-2.62	0.40	-2.36508	-2.48063	-2.70551	-3.21895	-2.83175	-2.6281	5.66	24.90	9.97	18.24	-2.71	0.30
Q17R60	Interphotoreceptor matrix proteoglycan 1	IMPG1	4.80	-0.69379	-0.74267	-2.21543	-1.85193	-1.19271	NA	2.40	17.72	NA	42.04	-1.34	0.68	-0.92778	-1.02736	-2.11286	-1.89608	-0.84878	NA	4.88	10.61	NA	37.55	-1.36	0.59
P04181	Ornithine aminotransferase, mitochondrial	OAT	3.33	0.034428	0.40025	0.550178	1.888438	0.980367	0.555263	17.83	61.26	20.69	38.97	0.73	0.64	-0.22046	0.084517	0.718681	1.12038	0.546192	0.098772	14.89	19.56	21.76	35.20	0.39	0.49
P20941	Phosducin	PDC	5.00	-1.27287	-0.69365	-1.04769	-0.95804	-1.18253	NA	28.01	4.39	NA	8.17	-1.03	0.22	-1.64995	-0.68014	-0.87848	-0.98419	-0.3309	NA	45.82	5.18	NA	29.06	-0.90	0.49
Q07954	Prolow-density lipoprotein receptor-related protein 1	LRP1	2.50	0.663307	0.675935	-0.04332	0.42437	0.532895	0.159588	0.62	22.72	18.20	16.65	0.40	0.29	0.696389	0.197595	-0.02888	-0.30674	0.982944	0.239435	24.21	13.58	35.66	27.42	0.30	0.47
Q8IVA1	Purkinje cell protein 2 homolog	PCP2	1.33	-0.3274	-0.73982	NA	NA	NA	-0.11192	20.08	NA	NA	19.82	-0.39	0.32	-0.20068	-0.89526	NA	NA	NA	-0.28046	33.40	NA	NA	11.06	-0.46	0.38
Q6ZS82	Regulator of G-protein signaling 9-binding protein	RGS9BP	3.00	0.001181	-1.8708	-1.08882	-0.74026	NA	NA	80.73	17.00	NA	12.40	-0.92	0.78	0.195026	-1.18326	-1.10522	-1.00886	NA	NA	62.85	4.72	NA	34.61	-0.78	0.65
P12271	Retinaldehyde-binding protein 1	RLBP1	19.50	-0.85143	-0.83922	-1.04934	-0.91068	-1.32655	-1.45245	0.60	6.79	6.17	18.67	-1.07	0.26	-0.8373	-0.7701	-1.1841	-1.14454	-1.13222	-0.86295	3.29	1.94	13.16	12.52	-0.99	0.18
P78363	Retinal-specific ATP-binding cassette transporter	ABCA4	23.00	-0.59029	-0.29793	-1.20439	-1.87592	-0.25724	-2.21216	14.28	32.33	83.43	35.81	-1.07	0.83	-1.27589	-0.31303	-1.23204	-1.6753	0.170454	-0.69376	45.52	21.56	41.14	40.75	-0.84	0.69
Q16518	Retinoid isomerohydrolase	RPE65	24.67	-2.31095	-2.40194	-3.02432	-2.82179	-2.6966	-2.3266	4.46	9.91	18.04	19.32	-2.60	0.30	-2.48018	-2.37398	-3.01347	-2.94966	-2.64686	-2.2344	5.20	3.13	20.08	20.64	-2.62	0.31
Q8TC12	Retinol dehydrogenase 11	RDH11	4.00	-0.35634	-0.60292	-0.756	-0.72536	-0.10576	-0.1664	12.06	1.50	2.97	21.15	-0.45	0.28	-0.57655	-0.74256	-0.66522	-1.04641	-0.03792	-0.44967	8.13	18.58	20.05	22.52	-0.59	0.34
P10745	Retinol-binding protein 3	RBP3	23.67	-2.2667	-1.0768	-1.76093	-1.6656	-1.88047	-2.15628	55.22	4.67	13.48	15.74	-1.80	0.42	-2.44873	-1.2085	-1.94952	-1.54514	-1.85102	-2.31733	57.30	19.69	22.66	13.43	-1.89	0.47
O15537	Retinoschisin	RS1	3.80	-0.74697	-0.54167	-1.40597	-1.05166	NA	-0.52117	10.05	17.28	NA	23.90	-0.85	0.38	-0.3272	-1.25718	-0.19362	-1.32243	NA	-0.78895	44.07	52.67	NA	4.80	-0.78	0.52
Q15835	Rhodopsin kinase	GRK1	6.17	-0.7526	-0.71904	-1.15339	-0.83255	1.000629	-0.61048	1.64	15.66	71.67	55.45	-0.51	0.76	-1.13593	-0.82487	-0.90762	-1.18815	-0.82881	-0.56181	15.19	13.71	13.05	13.30	-0.91	0.23
P16499	Rod cGMP-specific 3',5'-cyclic phosphodiesterase subunit alpha	PDE6A	16.67	-1.19825	-1.05101	-1.76093	-1.48946	-0.80233	-1.02078	7.21	13.27	10.69	23.94	-1.22	0.35	-1.40282	-0.90307	-1.57236	-1.65291	-0.45239	-1.39741	24.25	3.95	44.73	25.63	-1.23	0.46
P35913	Rod cGMP-specific 3',5'-cyclic phosphodiesterase subunit beta	PDE6B	13.17	-1.48295	-1.20485	-1.79916	-1.8682	-2.15332	-1.80665	13.59	3.38	16.91	24.11	-1.72	0.33	-1.6153	-1.28323	-1.61591	-1.60376	-2.01434	-1.77057	16.20	0.60	11.92	15.33	-1.65	0.24
Q03395	Rod outer segment membrane protein 1	ROM1	6.83	-0.35609	-0.71779	-1.78174	-2.03104	-0.14047	-0.26858	17.64	12.19	6.28	50.66	-0.88	0.82	-0.5603	-0.89403	-1.90999	-2.04433	-1.56749	-0.69758	16.28	6.58	41.39	40.07	-1.28	0.64
P47804	RPE-retinal G protein-coupled receptor	RGR	6.83	-1.64711	-1.27523	-1.78265	-2.11992	-0.76124	-1.16513	18.13	16.46	19.67	33.94	-1.46	0.49	-1.55482	-1.41394	-1.64174	-2.03396	-0.59097	-0.81132	6.90	19.11	10.78	41.88	-1.34	0.54

Extracellular matrix

UniProt	Protein name	Gene name	Peptide mean	08/017 hESC-RPE to hRPE												08/023 hESC-RPE to hRPE											
				Analysis						Technical replicate CV			Biological CV	Fold change (mean)	StDev	Analysis						Technical replicate CV			Biological CV	Fold change (mean)	StDev
				1.1	1.2	2.1	2.2	3.1	3.2	1	2	3				1.1	1.2	2.1	2.2	3.1	3.2	1	2	3			
P09382	Galectin-1	LGALS1	6.83	0.763182	0.70359	0.594034	0.297339	0.751501	0.874264	2.92	14.49	6.01	12.70	0.66	0.20	0.638025	0.687653	1.158265	0.737521	1.134361	1.219714	2.43	20.48	4.18	17.59	0.93	0.27
Q08380	Galectin-3-binding protein	LGALS3BP	5.20	1.58896	1.811201	NA	0.254445	1.024396	1.362343	10.87	NA	16.49	45.92	1.21	0.61	1.008303	0.7056	NA	0.142839	0.583239	2.584213	14.78	NA	84.88	61.46	1.00	0.94
A0A024R8T0	Integrin beta	ITGB4	2.20	1.472631	1.594171	1.434225	2.570083	NA	1.935328	5.95	52.96	NA	19.63	1.80	0.47	1.325605	1.081709	0.957238	2.058359	NA	2.515708	11.93	51.49	NA	48.50	1.59	0.67
P05556	Integrin beta-1	ITGB1	2.33	0.149462	-0.28122	0.62839	0.613721	0.735134	0.260371	20.95	0.72	23.06	23.18	0.35	0.39	-0.25717	-0.68085	0.93993	0.767182	0.983781	0.313317	20.62	8.46	32.28	41.53	0.34	0.69
P26012	Integrin beta-8	ITGB8	2.75	NA	0.56944	-0.4347	1.024704	NA	-0.2138	NA	66.00	NA	26.88	0.24	0.68	NA	0.442323	-0.43877	0.742959	NA	-0.28724	NA	54.88	NA	24.64	0.11	0.57
L7RXH0	Integrin, alpha V	ITGAV	7.50	0.720813	0.426053	0.602438	0.157323	0.838343	0.658794	14.40	21.64	8.79	12.23	0.57	0.24	0.716264	0.766325	0.62317	0.033178	0.528812	0.463717	2.45	28.52	3.19	13.66	0.52	0.26
Q5NKV8	Intercellular adhesion molecule 1	ICAM1	2.83	1.480483	1.705221	1.145721	0.670808	0.612402	1.237177	10.99	23.07	30.15	27.82	1.14	0.43	1.687572	2.235523	1.899533	1.475243	0.779106	1.448814	26.54	20.65	32.25	27.83	1.59	0.49
A0A087WWD4	Neural cell adhesion molecule 1	NCAM1	9.33	-1.64157	-1.37608	-1.16463	-1.99172	-1.59852	-1.40415	12.98	39.46	9.51	0.79	-1.53	0.28	-1.60994	-1.32237	-1.1156	-1.61918	-1.89111	-1.69584	14.05	24.43	9.56	15.42	-1.54	0.28
Q9Y490	Talin-1	TLN1	12.67	0.72129	1.513624	0.949289	1.809034	0.997622	1.045696	37.89	40.93	2.36	15.03	1.17	0.41	0.899862	0.95615	0.565002	1.529835	1.094343	1.168056	2.76	45.60	3.61	7.85	1.04	0.32
D9ZGG2	Vitronectin	VTN	2.00	-0.06461	NA	-1.42221	-0.94073	NA	NA	NA	23.38	NA	51.31	-0.81	0.69	-0.07303	NA	-1.07559	-0.93318	NA	NA	NA	6.97	NA	44.05	-0.69	0.54

Pigmentation

UniProt	Protein name	Gene name	Peptide mean	08/017 hESC-RPE to hRPE												08/023 hESC-RPE to hRPE											
				Analysis						Technical replicate CV			Biological CV	Fold change (mean)	StDev	Analysis						Technical replicate CV			Biological CV	Fold change (mean)	StDev
				1.1	1.2	2.1	2.2	3.1	3.2	1	2	3				1.1	1.2	2.1	2.2	3.1	3.2	1	2	3			
P17643	5,6-dihydroxyindole-2-carboxylic acid oxidase	TYRP1	5.00	1.903488	1.885831	0.852643	1.555717	0.947329	1.357277	0.87	33.79	19.96	29.37	1.42	0.45	1.238022	1.159827	0.815897	0.686062	0.527665	1.128748	3.83	6.36	29.04	16.69	0.93	0.29
P36955	Pigment epithelium-derived factor	SERPINF1	3.17	0.815571	1.024376	0.644927	0.269614	0.412975	0.25185	10.22	18.29	7.89	22.27	0.57	0.31	0.730729	0.683406	0.197634	0.607249	0.361375	0.200093	2.32	19.94	7.90	15.37	0.46	0.24

Phagocytosis

UniProt	Protein name	Gene name	Peptide mean	08/017 hESC-RPE to hRPE												08/023 hESC-RPE to hRPE											
				Analysis						Technical replicate CV			Biological CV	Fold change (mean)	StDev	Analysis						Technical replicate CV			Biological CV	Fold change (mean)	StDev
				1.1	1.2	2.1	2.2	3.1	3.2	1	2	3				1.1	1.2	2.1	2.2	3.1	3.2	1	2	3			
V9HWI3	Cathepsin D (Lysosomal aspartyl peptidase), isoform CRA_a	CTSD	36.67	-0.49983	-0.59917	-1.03027	-1.03482	-0.13724	-0.14155	4.87	0.22	0.21	30.24	-0.57	0.40	-0.55998	-0.53164	-1.23085	-1.21309	-0.01267	0.074579	1.39	0.87	4.27	41.81	-0.58	0.56
P16070	CD44 antigen	CD44	1.00	0.313993	-0.16535	0.127275	0.434745	0.585545	0.433295	23.28	15.01	7.46	14.50	0.29	0.27	0.188695	-0.34794	0.382924	0.177392	0.259688	0.201769	26.00	10.06	2.84	12.18	0.14	0.25
E7EQR4	Ezrin	EZR	23.17	1.584306	1.908408	1.192397	1.184951	1.559935	1.022925	15.82	0.36	26.02	21.44	1.41	0.33	1.508392	1.967914	1.382341	1.497579	1.806084	1.332742	22.33	5.65	22.99	10.95	1.58	0.25
L7RXH0	Integrin, alpha V	ITGAV	7.50	0.720813	0.426053	0.602438	0.157323	0.838343	0.658794	14.40	21.64	8.79	12.23	0.57	0.24	0.716264	0.766325	0.62317	0.033178	0.528812	0.463717	2.45	28.52	3.19	13.66	0.52	0.26
F5GZN3	Lactadherin	MFGE8	2.00		0.526464		0.840144		-0.63551	NA	NA	NA	45.50	0.24	0.78		0.934752		0.925694		0.21271	NA	NA	NA	26.03	0.69	0.41
A0A024RDG6	Scavenger receptor class B, member 2, isoform CRA_a	SCARB2	9.17	0.411342	0.23943	0.003706	-0.14593	0.372624	-0.11667	8.42	7.33	23.75	13.66	0.13	0.25	0.051865	0.009444	-0.40621	-0.28967	0.547466	0.126494	2.08	5.71	20.49	23.85	0.01	0.34
H0YDL9	Tetraspanin (Fragment)	CD81	1.00	-0.21392	-0.06782	0.233219	0.121664	0.2488	NA	7.15	5.46	NA	13.77	0.06	0.20	-1.03684	-0.12179	-0.96673	-0.57303	0.565324	NA	43.40	19.18	NA	52.29	-0.43	0.66

Transporters and channel proteins

UniProt	Protein name	Gene name	Peptide mean	08/017 hESC-RPE to hRPE												08/023 hESC-RPE to hRPE											
				Analysis						Technical replicate CV			Biological CV	Fold change (mean)	StDev	Analysis						Technical replicate CV			Biological CV	Fold change (mean)	StDev
				1.1	1.2	2.1	2.2	3.1	3.2	1	2	3				1.1	1.2	2.1	2.2	3.1	3.2	1	2	3			
Q8N169	Amino acid transporter	SLC1A3	3.33	-1.35357	NA	NA	NA	-2.00792	-1.41957	NA	NA	28.44	16.12	-1.59	0.36	-1.6527	NA	NA	NA	-2.50395	-2.32375	NA	NA	8.82	36.34	-2.16	0.45
Q6JSD8	Aquaporin 1 splice variant 2 (Fragment)	AQP1	1.00	0.841618	2.084512	2.275493	2.145216	0.309504	NA	57.41	6.38	NA	57.29	1.53	0.90	0.604838	1.870517	1.804424	1.616907	0.343071	NA	58.34	9.18	NA	42.96	1.25	0.72
Q5SRT3	Chloride intracellular channel protein	CLIC1	2.33	0.201574	0.197998	0.170543	0.032458	0.856059	-0.08125	0.18	6.76	44.39	13.18	0.23	0.33	-0.03302	0.18078	-0.03743	0.131596	0.593553	0.303289	10.46	8.27	14.18	16.35	0.19	0.24
Q96NY7	Chloride intracellular channel protein 6	CLIC6	5.67	1.537946	1.458189	0.726324	0.776623	1.792673	0.45583	3.91	2.47	61.20	25.06	1.12	0.54	0.96944	0.98003	1.755946	1.744542	1.898277	0.686664	0.52	0.56	56.12	26.24	1.34	0.52
Q01650	Large neutral amino acids transporter small subunit 1	SLC7A5	1.25	NA	0.873917	NA	0.863226	3.253014	1.83875	NA	NA	64.25	80.25	1.71	1.13	NA	1.259375	NA	1.010685	3.038763	1.584927	NA	NA	65.78	59.14	1.72	0.91
P05023	Sodium/potassium-transporting ATPase subunit alpha-1	ATP1A1	33.17	-0.03971	0.187868	-0.12017	0.095007	0.193886	0.110032	11.13	10.53	4.11	5.58	0.07	0.13	0.025843	0.11108	-0.0338	0.067539	0.394944	0.168628	4.18	4.96	11.07	10.10	0.12	0.15
Q6LEU2	Sodium/potassium-transporting ATPase subunit beta (Fragment)	ATP1B1	6.67	-0.00513	0.199982	0.370764	0.093031	-0.16487	-0.01377	10.04	13.57	7.40	11.22	0.08	0.19	-0.13737	0.245409	0.546062	0.121656	-0.12589	-0.50602	18.65	20.65	18.52	22.21	0.02	0.36
A0A024QZT0	Voltage-dependent anion channel 2, isoform CRA_c	VDAC2	16.17	-0.97212	-1.2679	-2.18618	-2.04403	-0.75414	-0.76907	14.45	6.96	0.73	42.51	-1.33	0.64	-0.96836	-1.07068	-2.15733	-2.04641	-1.02945	-1.00182	5.01	5.43	1.35	37.01	-1.38	0.56
P21796	Voltage-dependent anion-selective channel protein 1	VDAC1	29.00	-1.46926	-1.38399	-2.06146	-2.12681	-1.14001	-1.03722	4.18	3.20	5.04	33.06	-1.54	0.46	-1.38325	-1.35695	-2.00838	-1.99421	-0.99278	-0.87351	1.29	0.69	5.84	35.46	-1.43	0.48
Q9Y277	Voltage-dependent anion-selective channel protein 3	VDAC3	7.67	-0.51945	-0.81664	-1.11844	-0.86099	-0.20878	-0.42639	14.51	12.58	10.65	23.18	-0.66	0.33	-0.68299	-0.19104	-1.10522	-0.88625	-0.56903	-0.13301	23.88	10.71	21.21	22.94	-0.59	0.38
